# Supplementary material for: Mechanistic study on the side arm effect in a palladium/Xu-Phos-catalyzed enantioselective alkoxyalkenylation of γ-hydroxyalkenes
Source: Nat Commun. 2023 Nov 22;14:7611. doi: 10.1038/s41467-023-43202-5 (PMC10665319; doi:10.1038/s41467-023-43202-5)
Supplement: Supplementary file 1 — Supplementary Information [file 41467_2023_43202_MOESM1_ESM.pdf]

Supplementary Information for

Mechanistic study on the side arm effect in a palladium/Xu-Phos-catalyzed enantioselective alkoxyalkenylation of γ-hydroxyalkenes

Shuai Zhu[1†], Zihao Ye[2†], Ming-Jie Chen[1†], Lei Wang[3], Yu-Zhuo Wang[1], Ke-Nan Zhang[1], Wen-Bo Li[1], Han-Ming Ding[1], Zhi-Ming Li[2*], and Junliang Zhang[1,2,3,4*]

[1]Shanghai Key Laboratory of Green Chemistry and Chemical Processes, School of Chemistry and Molecular Engineering, East China Normal University, 3663 N. Zhongshan Road, Shanghai 200062, P.R. China
[2]Department of Chemistry, Fudan University, 2005 Song hu Road, Shanghai 200438, P.R. China
[3]School of Chemistry & Chemical Engineering, Yangzhou University, Yangzhou 225002, China
[4]Henan Normal Univ, Sch Chem & Chem Engn, Xinxiang 453007, Henan, Peoples R China
*Corresponding author. Email: junliangzhang@fudan.edu.cn (J.Z.); zmli@fudan.edu.cn (Z.L.)
[†]These authors contributed equally to this work.

1. General Information: 2

2. General Procedure for the Synthesis of Xu-Phos 3

3. General procedure for preparation of substrates....................50

4. General Procedure and HPLC spectra 52

5. X-ray structure of Xu6 and 13. 126

6. Procedure of vibrational circular dichroism (VCD) experiment 126

7. Computational details 126

9. ^{1}H, ^{13}C, ^{31}P, ^{19}F NMR spectra....................138

9. References 309

1. General Information

Unless otherwise noted, all reactions were carried out under an argon atmosphere; materials obtained from commercial suppliers were used directly without further purification. The [±] D was recorded using PolAAr 3005 High Accuracy Polarimeter (λ = 589 nm, T = 20 ºC). ^{1}H NMR spectra, ^{13}C NMR spectra, and ^{31}P NMR spectra were recorded on a Bruker 400 MHz and 500 MHz spectrometer in $CDCl_3$ NMR. Experiments are reported in δ units, parts per million (ppm), and were referenced to $CDCl_3$ (δ 7.26 or 77.0 pp m) as the internal standard. The data is being reported as (s = singlet, d = doublet, dd = doublet of doublet, t = triplet, m = multiplet or unresolved, br = broad signal, coupling constant(s) in Hz, integration). Tetrahydrofuran (THF), toluene, hexane and ether were dried with sodium benzophenone and distilled before use; Reactions were monitored by thin layer chromatography (TLC) using silicycle pre-coated silica gel plates. Flash column chromatography was performed on silica gel 60 (particle size 300-400 mesh ASTM, purchased from Yantai, China) and eluted with petroleum ether/ethyl acetate.

2. General Procedure for the Synthesis of Xu-Phos

2.1 Synthesis of Xu3, Xu11

2.1.1 (*R*)-N-((*S*)-[1,1'-biphenyl]-4-yl(2-(dicyclohexylphosphanyl)-phenyl)-methyl)-N,2-dimethyl-propane-2-sulfinamide (Xu3)

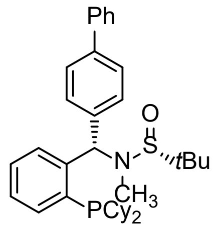

Xu3

To a solution of dicyclohexylphosphine borane (5 mmol) in dry PhMe/THF (2:1, 10 mL) was added *n*-BuLi (5 mmol, 2.4 M in hexane) dropwise under argon at -78 °C. The resulting solution at this temperature during 1 hour and 1,2-dibromobenzene (5 mmol) was added dropwise followed by *n*-BuLi (5 mmol, 2.4 M in hexane). After 10 minutes at -78 °C, (Rs)-sulfinyl imine (6 mmol) was added and the reaction mixture was warmed to room temperature overnight. The reaction mixture was cooled to 0 °C and added Methyl trifluoromethanesulfonate (7.5 mmol). The resulting solution was stirred at this temperature during 1 hour. Then, the reaction mixture was quenched by the addition of $NaHCO_3$ (aq.) and diluted with EtOAc. The organic layer was separated, and the aqueous layer was extracted twice with EtOAc. The combined organic layers were dried over Na_2SO_4, filtered, concentrated. The crude product was dealed with Et_2NH (20 mL) and the resulting solution was stirred under argon at 50 °C. After the reaction was complete (monitored by TLC), solvent was removed under reduced pressure. The crude product was then purified by flash column chromatography on silica gel (Petroleum ether: Acetone = 30:1) to afford the product **Xu3** as a white solid (1.3 g, 45% yield). Mp: 68.4-70.2 °C. $[\alpha]_D^{20}$ = 52.8 (*c* = 0.4, Chloroform). ^{1}H NMR (500 MHz, Chloroform-*d*) δ 7.84 – 7.74 (m, 1 H), 7.59 – 7.53 (m, 2 H), 7.54 – 7.45 (m, 3 H), 7.47 – 7.38 (m, 3 H), 7.35 – 7.29 (m, 2 H), 7.29 – 7.23 (m, 2 H), 6.94 (d, *J* = 10.0 Hz, 1 H), 2.64 (s, 3 H), 2.01 – 1.82 (m, 2 H), 1.83 – 1.72 (m, 1 H), 1.72 – 1.62 (m, 2 H), 1.62 – 1.57 (m, 1 H), 1.57 – 1.51 (m, 1 H), 1.51 – 1.39 (m, 4 H), 1.36 – 1.17 (m, 6 H), 1.15 – 1.06 (m, 10 H), 0.94 – 0.86 (m, 3 H), 0.65 – 0.51 (m, 1 H). ^{13}C NMR (125 MHz, Chloroform-*d*) δ 147.1 (d, *J* = 22.2 Hz), 140.7, 139.9, 139.1, 134.9 (d, *J* = 21.2 Hz), 133.1 (d, *J* = 3.2 Hz), 131.0, 128.7, 128.6, 128.0 (d, *J* = 4.9 Hz), 127.1, 126.9, 126.6, 69.8 (d, *J* = 31.8 Hz), 58.6, 34.7 (dd, *J* = 12.9, 10.8 Hz), 30.6 (d, *J* = 17.8

Hz), 30.3 – 29.8 (m), 29.5 (d, J = 10.0 Hz), 29.3 (d, J = 10.0 Hz), 27.0 (dd, J = 12.0, 9.7 Hz), 26.3, 26.1, 24.0. ^{31}P NMR (202 MHz, Chloroform-*d*) δ -16.85. HRMS (ESI) calculated for [$C_{36}H_{49}NOPS$] [M+H]$^+$: 574.3267 found: 574.3269.

2.1.2 (*R*)-N-((*S*)-[1,1'-biphenyl]-4-yl(2-(dicyclohexylphosphanyl)-4,5-dimethoxy-phenyl)-methyl)-N,2-dimethylpropane-2-sulfinamide (Xu11)

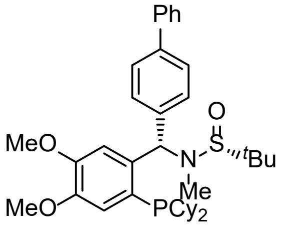

Xu11

To a solution of dicyclohexylphosphine borane (5 mmol) in dry PhMe/THF (2:1, 10 mL) was added *n*-BuLi (5 mmol, 2.4 M in hexane) dropwise under argon at -78 °C. The resulting solution at this temperature during 1 hour and 1,2-dibromo-4,5-dimethoxybenzene (5 mmol) was added dropwise followed by *n*-BuLi (5 mmol, 2.4 M in hexane). After 10 minutes at -78 °C, (Rs)-sulfinyl imine (6 mmol) was added and the reaction mixture was warmed to room temperature overnight. The reaction mixture was cooled to 0 °C and added Methyl trifluoromethanesulfonate (7.5 mmol). The resulting solution was stirred at this temperature during 1 hour. Then, the reaction mixture was quenched by the addition of $NaHCO_3$ (aq.) and diluted with EtOAc. The organic layer was separated, and the aqueous layer was extracted twice with EtOAc. The combined organic layers were dried over Na_2SO_4, filtered, concentrated. The crude product was dealed with Et_2NH (20 mL) and the resulting solution was stirred under argon at 50 °C. After the reaction was complete (monitored by TLC), solvent was removed under reduced pressure. The crude product was then purified by flash column chromatography on silica gel (Petroleum ether : Acetone = 30:1) to afford the product **Xu11** as a white solid (1.4 g, 44% yield). Mp: 75.6-78 °C. $[\alpha]_D^{20}$ = 112.7 (*c* = 0.4, Chloroform-*d*). ^{1}H NMR (500 MHz, Chloroform-*d*) δ 7.59 – 7.55 (m, 2H), 7.53 (d, J = 3.7 Hz, 1H), 7.51 – 7.47 (m, 2H), 7.43 (t, J = 7.6 Hz, 2H), 7.34 (t, J = 7.4 Hz, 1H), 7.29 (d, J = 2.6 Hz, 2H), 6.98 (d, J = 2.2 Hz, 1H), 6.83 (d, J = 10.3 Hz, 1H), 4.00 (s, 3H), 3.93 (s, 3H), 2.67 (s, 3H), 1.96 – 1.79 (m, 4H), 1.73 – 1.65 (m, 2H), 1.58 – 1.47 (m, 4H), 1.47 – 1.38 (m, 3H), 1.35 – 1.16 (m, 7H), 1.12 (s, 9H), 0.92 – 0.87 (m, 2H). ^{13}C NMR (125 MHz, Chloroform-*d*) δ 149.8, 147.2, 140.7, 140.1 (d, J

= 23.0 Hz), 139.9, 139.4, 131.3, 128.6, 127.1, 126.9, 126.4, 125.8 (d, J = 20.1 Hz), 115.1 (d, J = 2.9 Hz), 111.1 (d, J = 5.6 Hz), 69.9 (d, J = 33.1 Hz), 58.7, 56.0, 55.9, 35.0 (dd, J = 12.6, 8.3 Hz), 31.0 – 30.4 (m), 30.1 (d, J = 15.6 Hz), 229.4 (dd, J = 11.4, 9.4 Hz), 27.3-26.7 (m), 26.3, 26.1, 24.3. ^{31}P NMR (202 MHz, Chloroform-*d*) δ -17.3. HRMS (ESI) calculated for [$C_{38}H_{53}NO_3PS$] [M+H]$^+$: 634.3478 found: 634.3483.

2.2 Synthesis of Xu5, Xu6 and Xu7 [1]

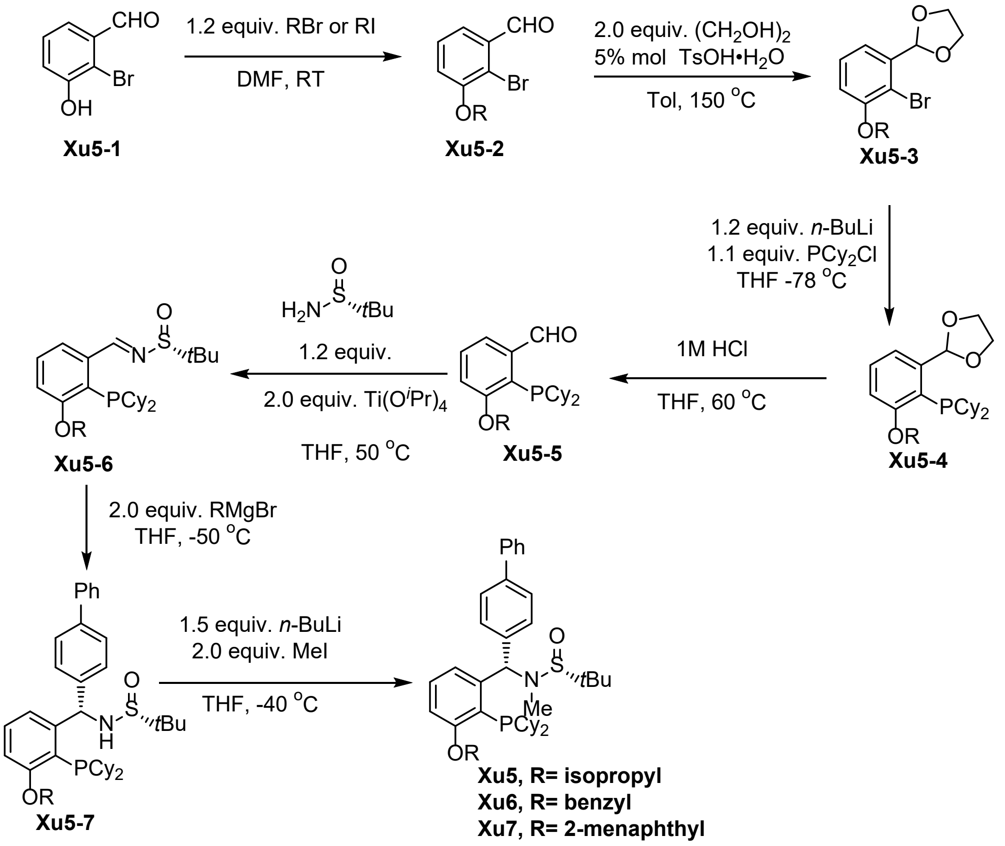

2.2.1 2-bromo-3-isopropoxybenzaldehyde (Xu5-2)

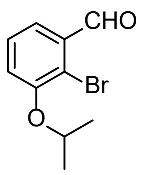

Xu5-2

Prepared from 2-bromo-3-hydroxybenzaldehyde **Xu5-1** (30 mmol) in DMF (50 mL) was added 2-iodopropane (36mmol, 1.2 equiv.) and K_2CO_3 (45 mmol, 1.5 equiv.). The resulting solution was stirred at room temperature overnight. The reaction mixture was quenched by the addition of H_2O

and diluted with EtOAc. The organic layer was separated, and the aqueous layer was extracted twice with EtOAc. The combined organic layers were dried over Na_2SO_4, filtered, concentrated. the crude product was then purified by flash column chromatography on silica gel (petroleum ether: ethyl acetate = 40: 1) to afford the product **Xu5-2** as a white liquid (5.5 g, 75% yield). ^{1}H NMR (500 MHz, Chloroform-*d*) δ 10.42 (s, 1 H), 7.49 (d, *J* = 7.7 Hz, 1 H), 7.32 (t, *J* = 7.9 Hz, 1 H), 7.13 (d, *J* = 8.1 Hz, 1 H), 4.65 – 4.53 (m, 1 H), 1.40 (d, *J* = 6.1 Hz, 6 H) ppm. ^{13}C NMR (125 MHz, Chloroform-*d*) δ 192.4, 154.9, 134.9, 128.0, 121.4, 120.4, 118.9, 72.6, 21.9. HRMS (ESI) calculated for $[C_{10}H_{12}BrO_2]$ $[M+H]^+$: 243.0015 found: 243.0017.

2.2.2 3-(benzyloxy)-2-bromobenzaldehyde (Xu6-2)

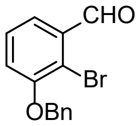

Xu6-2

To a solution of 2-bromo-3-hydroxybenzaldehyde **Xu5-1** (30 mmol) in DMF (50 mL) was added benzyl bromide (36mmol, 1.2 equiv.) and K_2CO_3 (45 mmol, 1.5 equiv.). The resulting solution was stirred at room temperature overnight. The reaction mixture was quenched by the addition of H_2O and diluted with EtOAc. The organic layer was separated, and the aqueous layer was extracted twice with EtOAc. The combined organic layers were dried over Na_2SO_4, filtered, concentrated. The crude product was then washed by petroleum ether with a little ethyl acetate to afford the product **Xu6-2** as a white solid (6.7 g, 77% yield). Mp: 64.0 – 64.5 °C. ^{1}H NMR (400 MHz, Chloroform-*d*) δ 10.43 (d, J = 0.8 Hz, 1H), 7.49 (ddd, J = 15.1, 7.9, 1.3 Hz, 3H), 7.43 – 7.36 (m, 2H), 7.36 – 7.29 (m, 2H), 7.14 (dd, J = 8.1, 1.5 Hz, 1H), 5.19 (s, 2H). ^{13}C NMR (100 MHz, Chloroform-*d*) δ 192.24, 155.39, 135.94, 134.92, 128.72, 128.28, 128.23, 127.06, 121.79, 118.79, 117.90, 71.29. HRMS (ESI) calculated for $[C_{14}H_{12}BrO_2]$ $[M+H]^+$: 291.0015 found: 291.0013.

2.2.3 2-bromo-3-(naphthalen-2-ylmethoxy)-benzaldehyde (Xu7-2)

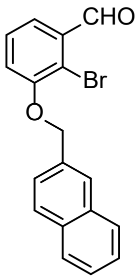

Xu7-2

Prepared from 2-bromo-3-hydroxybenzaldehyde **Xu5-1** (30 mmol) in DMF (50 mL) was added 2-(bromomethyl) naphthalene (36mmol, 1.2 equiv.) and K_2CO_3 (45 mmol, 1.5 equiv.). The resulting solution was stirred at room temperature overnight. The reaction mixture was quenched by the addition of H_2O and diluted with EtOAc. The organic layer was separated, and the aqueous layer was extracted twice with EtOAc. The combined organic layers were dried over Na_2SO_4, filtered, concentrated and washing by petroleum ether with a little ethyl acetate to afford the product **Xu7-2** as a white solid (8.7 g, 85% yield). Mp: 130.3 – 131.2 °C. ^{1}H NMR (500 MHz, Chloroform-*d*) δ 10.47 (s, 1 H), 8.00 – 7.80 (m, 4 H), 7.59 (dd, *J* = 8.5, 1.8 Hz, 1H), 7.56 – 7.47 (m, 3 H), 7.33 (t, *J* = 7.9 Hz, 1 H), 7.20 (dd, *J* = 8.1, 1.5 Hz, 1 H), 5.36 (s, 2 H). ^{13}C NMR (125 MHz, Chloroform-*d*) δ 192.2, 155.3, 134.8, 133.3, 133.2, 133.1, 128.5, 128.2, 127.9, 127.7, 126.3, 126.2, 126.0, 124.7, 121.8, 118.8, 117.8, 71.4. HRMS (ESI) calculated for $[C_{18}H_{14}BrO_2]$ $[M+H]^+$: 341.0172 found: 341.0175.

2.2.4 2-(2-bromo-3-isopropoxyphenyl)-1,3-dioxolane (Xu5-3)

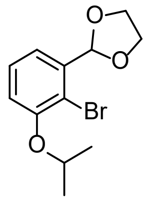

Xu5-3

Prepared from 2-bromo-3-isopropoxybenzaldehyde **Xu5-2** (20 mmol) in 50 mL toluene, was added ethylene glycol (40 mmol, 2.0 equiv.) and *p*-toluenesulfonic acid (1 mmol, 5% mol). The resulting solution was stirred 18 hours at 150 °C. The reaction mixture was washed by the addition of H_2O and the combined organic layers were dried over Na_2SO_4, filtered, concentrated, the crude product was then purified by flash column chromatography on silica gel (Petroleum ether: ethyl acetate = 20: 1) to afford the product **Xu5-3** as a white liquid (4.9 g, 85% yield). ^{1}H NMR (500 MHz, Chloroform-*d*) δ 7.26 (t, *J* = 7.9 Hz, 1 H), 7.20 (dd, *J* = 7.8, 1.7 Hz, 1 H), 6.92 (dd, *J* = 8.0, 1.7 Hz, 1 H), 6.16 (s, 1 H), 4.62 – 4.49 (m, 1 H), 4.17 – 4.10 (m, 2 H), 4.10 – 4.03 (m, 2 H), 1.38 (d, *J* = 6.1 Hz, 6 H). ^{13}C NMR (125 MHz, Chloroform-*d*) δ 154.6, 138.3, 127.7, 119.5, 116.2, 114.7, 102.7, 72.3, 65.3, 21.9. HRMS (ESI) calculated for $[C_{12}H_{16}BrO_3]$ $[M+H]^+$: 287.0277 found: 287.0279.

2.2.5 2-(3-(benzyloxy)-2-bromophenyl)-1,3-dioxolane (Xu6-3)

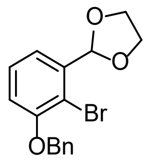

Xu6-3

To a solution of 3-(benzyloxy)-2-bromobenzaldehyde **Xu6-2** (20 mmol) in 50 mL toluene, was added ethylene glycol (40 mmol, 2.0 equiv.) and *p*-toluenesulfonic acid (1 mmol, 5% mol). The resulting solution was stirred 18 hours at 150 °C. The reaction mixture was washed by the addition of H_2O and the combined organic layers were dried over Na_2SO_4, filtered, concentrated. The crude product was then washed by petroleum ether with a little ethyl acetate to afford the product **Xu6-3** as a white solid (5.7 g, 85% yield). ^{1}H NMR (500 MHz, Chloroform-*d*) δ 7.51 (d, *J* = 6.9 Hz, 2 H), 7.45 – 7.39 (m, 2 H), 7.38 – 7.32 (m, 1 H), 7.32 – 7.24 (m, 2 H), 6.97 (dd, *J* = 7.2, 2.4 Hz, 1 H), 6.22 (s, 1 H), 5.19 (s, 1 H), 4.21 – 4.06 (m, 4 H). ^{13}C NMR (125 MHz, Chloroform-*d*) δ 154.9, 138.3, 136.3, 128.4, 127.8, 127.8, 126.9, 119.7, 114.3, 113.4, 102.6, 70.9, 65.3. HRMS (ESI) calculated for [$C_{16}H_{16}BrO_3$] $[M+H]^+$: 335.0277 found: 325.0279. Mp: 82.7 – 83.4 °C.

2.2.6 2-(2-bromo-3-(naphthalen-2-ylmethoxy)-phenyl)-1,3-dioxolane (Xu7-3)

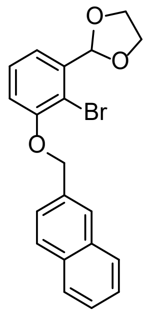

Xu7-3

Prepared from 2-bromo-3-(naphthalen-2-ylmethoxy)-benzaldehyde **Xu7-2** (20 mmol) in 50 mL toluene, was added ethylene glycol (40 mmol, 2.0 equiv.) and *p*-toluenesulfonic acid (1 mmol, 5% mol). The resulting solution was stirred 18 hours at 150 °C. The reaction mixture was washed by the addition of H_2O and the combined organic layers were dried over Na_2SO_4, filtered, concentrated, after washing by petroleum ether with a little ethyl acetate to afford the product **Xu7-3** as a white solid (6.9 g, 90% yield). Mp: 96.8 – 97.6 °C. ^{1}H NMR (500 MHz, Chloroform-*d*) δ 7.94 (s, 1 H), 7.92 – 7.84 (m, 3 H), 7.60 (dd, *J* = 8.4, 1.7 Hz, 1 H), 7.55 – 7.48 (m, 2 H), 7.32 – 7.22 (m, 2 H), 7.03 – 6.97 (m, 1 H), 6.24 (s, 1 H), 5.33 (s, 2 H), 4.22 – 4.14 (m, 2 H), 4.14 – 4.06 (m, 2 H). ^{13}C

NMR (125 MHz, Chloroform-*d*) δ 155.0, 138.4, 133.8, 133.1, 133.0, 128.3, 127.9, 127.6, 126.1, 126.0, 125.8, 124.7, 119.8, 114.4, 113.5, 102.6, 71.2, 65.4. HRMS (ESI) calculated for [$C_{20}H_{18}BrO_3$] $[M+H]^+$: 385.0434 found: 385.0438.

2.2.7 (2-(benzyloxy)-6-(1,3-dioxolan-2-yl)-phenyl)-dicyclohexylphosphane (Xu5-4)

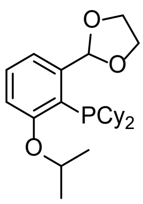

Xu5-4

Prepared from 2-(2-bromo-3-isopropoxyphenyl)-1,3-dioxolane **Xu5-3** (15 mmol) in 20 mL anhydrous THF, was added *n*-BuLi (18 mmol, 1.6 M in hexane) dropwise under argon at -78 °C, The resulting solution at this temperature during 1 hour, and dicyclohexylchlorophosphine (17 mmol, 1.1 equiv.) was added dropwise. The reaction mixture was warmed to room temperature overnight. The re action mixture was quenched by the addition of NH_4Cl (aq.) and diluted with EtOAc. The organic layer was separated, and the aqueous layer was extracted twice with EtOAc. The combined organic layers were dried over Na_2SO_4, filtered, concentrated, after washing by petroleum ether with a little ethyl acetate to afford the product **Xu5-4** as a white solid (4.5 g, 74% yield). Mp: 41.0-42.5 °C. ^{1}H NMR (500 MHz, Chloroform-*d*) δ 7.32 (t, *J* = 8.0 Hz, 1 H), 7.23 (dd, *J* = 7.8, 3.1 Hz, 1 H), 6.83 (d, *J* = 7.9 Hz, 1 H), 6.76 (d, *J* = 8.1 Hz, 1 H), 4.73 – 4.55 (m, 1 H), 4.16 – 4.08 (m, 2 H), 4.08 – 4.00 (m, 2 H), 2.54 – 2.38 (m, 2 H), 1.99 – 1.87 (m, 2 H), 1.86 – 1.72 (m, 2 H), 1.69 – 1.53 (m, 4 H), 1.3 8 (s, 3 H), 1.37 (s, 3 H), 1.33 – 1.23 (m, 6 H), 1.21 – 1.07 (m, 4 H), 1.02 – 0.91 (m, 2 H). ^{13}C NMR (125 MHz, Chloroform-*d*) δ 159.4 (d, *J* = 4.0 Hz), 146.5 (d, *J* = 21.5 Hz), 130.3, 123.6 (d, *J* = 30.3 Hz), 117.5 (d, *J* = 6.9 Hz), 110.7, 101.5 (d, *J* = 39.5 Hz), 68.3, 65.3, 34.7 (d, *J* = 9.7 H z), 32.8 (d, *J* = 24.3 Hz), 30.5 (d, *J* = 9.1 Hz), 27.3 (d, *J* = 8.7 Hz), 27.1 (d, *J* = 14.3 Hz), 26.3, 26.3, 21.7. ^{31}P NMR (202 MHz, Chloroform-*d*) δ -10.97. HRMS (ESI) calculated for [$C_{24}H_{38}O_3P$] $[M+H]^+$: 405.2553 found: 405.2556.

2.2.8 (2-(benzyloxy)-6-(1,3-dioxolan-2-yl)-phenyl)-dicyclohexylphosphane (Xu6-4)

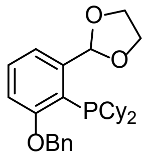

Xu6-4

To a solution of 2-(3-(benzyloxy)-2-bromophenyl)-1,3-dioxolane **Xu6-3** (15 mmol) in 20 mL anhydrous THF, was added *n*-BuLi (18 mmol, 1.6 M in hexane) dropwise under argon at -78 °C, The resulting solution at this temperature during 1 hour, and dicyclohexylchlorophosphine (17 mmol, 1.1 equiv.) was added dropwise. The reaction mixture was warmed to room temperature overnight. The re action mixture was quenched by the addition of NH_4Cl (aq.) and diluted with EtOAc. The organic layer was separated, and the aqueous layer was extracted twice with EtOAc. The combined organic layers were dried over Na_2SO_4, filtered, concentrated. The crude product was then washed by petroleum ether with a little ethyl acetate to afford the product **Xu6-4** as a white solid (6.0 g, 88% yield). Mp: 101.2-103.0 °C. ^{1}H NMR (500 MHz, Chloroform-*d*) δ 7.47 – 7.38 (m, 4 H), 7.40 – 7.29 (m, 3 H), 6.88 (d, *J* = 8.0 Hz, 1 H), 6.83 (d, *J* = 7.9 Hz, 1 H), 5.06 (s, 2 H), 4.19 – 4.08 (m, 2 H), 4.10 – 4.01 (m, 2 H), 2.41 – 2.28 (m, 2 H), 1.85 – 1.74 (m, 2 H), 1.73 – 1.65 (m, 2 H), 1.63 – 1.51 (m, 4 H), 1.35 – 1.18 (m, 4 H), 1.1 9 – 1.104 (m, 6 H), 1.03 – 0.92 (m, 2 H). ^{13}C NMR (125 MHz, Chloroform-*d*) δ 160.8 (d, *J* = 3.9 Hz), 146.3 (d, *J* = 21.3 Hz), 136.6, 130.5 (d, *J* = 1.5 Hz), 128.4, 128.0, 127.9, 123.9 (d, *J* = 31.3 Hz), 118.5 (d, *J* = 6.7 Hz), 111.1, 101.5 (d, *J* = 38.7 Hz), 70.4, 65.3, 34.1 (d, *J* = 9.7 H z), 32.6 (d, *J* = 23.6 Hz), 30.6 (d, *J* = 9.4 Hz), 27.1 (d, *J* = 8.9 Hz), 26.9 (d, *J* = 14.1 Hz), 26.2. ^{31}P NMR (202 MHz, Chloroform-*d*) δ -10.61. HRMS (ESI) calculated for [$C_{28}H_{38}O_3P$] $[M+H]^+$: 453.2553 found: 453.2556.

2.2.9 (2-(benzyloxy)-6-(1,3-dioxolan-2-yl)-phenyl)-dicyclohexylphosphane (Xu7-4)

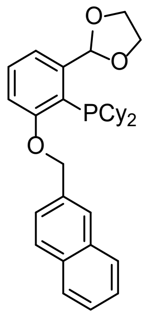

Xu7-4

Prepared from 2-(2-bromo-3-(naphthalen-2-ylmethoxy)phenyl)-1,3-dioxolane **Xu7-3** (15 mmol), in 20 mL anhydrous THF, was added *n*-BuLi (18 mmol, 1.6 M in hexane) dropwise under argon at -78 °C, The resulting solution at this temperature during 1 hour, and dicyclohexylchlorophosphine (17 mmol, 1.1 equiv.) was added dropwise. The reaction mixture was warmed to room temperature overnight. The re action mixture was quenched by the addition of NH_4Cl (aq.) and diluted with EtOAc. The organic layer was separated, and the aqueous layer was extracted twice with EtOAc. The combined organic layers were dried over Na_2SO_4, filtered, concentrated. The crude product was then purified by flash column chromatography on silica gel (petroleum ether: ethyl acetate = 20: 1) to afford the product **Xu7-4** as a white solid (5.3 g, 70% yield). Mp: 108.1-112.0 °C. ^{1}H NMR (500 MHz, Chloroform-*d*) δ 7.95 – 7.86 (m, 3 H), 7.86 – 7.82 (m, 1 H), 7.60 – 7.49 (m, 3 H), 7.41 – 7.31 (m, 2 H), 6.93 (dd, *J* = 7.6, 1.7 Hz, 1 H), 6.85 (d, *J* = 7.9 Hz, 1 H), 5.25 (s, 2 H), 4.18 – 4.11 (m, 2 H), 4.10 – 4.03 (m, 2 H), 2.47 – 2.35 (m, 2 H), 1.86 – 1.75 (m, 2 H), 1.71 – 1.55 (m, 6 H), 1.40 – 1.30 (m, 2 H), 1.29 – 1.18 (m, 2 H), 1.19 – 1.07 (m, 6 H), 1.07 – 0.95 (m, 2 H). ^{13}C NMR (125 MHz, Chloroform-*d*) δ 160.9 (d, *J* = 3.8 Hz), 146.4 (d, *J* = 21.3 Hz), 134.2, 133.2, 133.0, 130.6, 128.3, 127.8, 127.7, 126.5, 126.3, 126.1, 125.5, 124.0 (d, *J* = 31.3 Hz), 118.7 (d, *J* = 6.7 Hz), 111.3, 101.5 (d, *J* = 38.9 Hz), 70.5, 65.4 , 34.2 (d, *J* = 9.9 Hz), 32.6 (d, *J* = 23.7 Hz), 30.6 (d, *J* = 9.3 Hz), 27.2 (d, *J* = 8.8 Hz), 26.9 (d, *J* = 14.0 Hz), 26.3. ^{31}P NMR (202 MHz, Chloroform-*d*) δ -10.44. HRMS (ESI) calculated for $[C_{32}H_{40}O_3P]$ $[M+H]^+$: 503.2710 found: 503.2715.

2.2.10 2-(dicyclohexylphosphanyl)-3-isopropoxybenzaldehyde (Xu5-5)

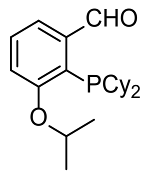

Xu5-5

Prepared from (2-(benzyloxy)-6-(1,3-dioxolan-2-yl)-phenyl)-dicyclohexylphosphane **Xu5-4** (2 mmol) in 2 mL THF, was added 3 mL HCl (1.0 M) under argon at 60 °C. The resulting solution was stirred 5 hours. The reaction mixture was quenched by the addition of $NaHCO_3$ (aq.) and diluted with EtOAc. The organic layer was separated, and the aqueous layer was extracted twice with EtOAc. The combined organic layers were dried over Na_2SO_4, filtered, concentrated and

purified by flash column chromatography on silica gel (petroleum ether: ethyl acetate = 20: 1) to afford the crude product **Xu5-5** as a yellow solid. Mp: 91.0-92.8 °C. ^{1}H NMR (500 MHz, Chloroform-*d*) δ 11.25 (dd, J = 9.1, 3.7 Hz, 1H), 7.48 (dt, J = 7.3, 3.4 Hz, 1H), 7.40 (td, J = 8.0, 3.6 Hz, 1H), 6.99 (dd, J = 8.3, 3.5 Hz, 1H), 4.68 (m, J = 5.8 Hz, 1H), 2.49 (dt, J = 11.5, 3.9 Hz, 2H), 1.93 (dt, J = 10.1, 4.5 Hz, 2H), 1.80 (d, J = 10.2 Hz, 2H), 1.65 – 1.57 (m, 4H), 1.42 (t, J = 4.9 Hz, 6H), 1.36 (dd, J = 9.6, 4.6 Hz, 1H), 1.29 (tt, J = 14.0, 6.5 Hz, 6H), 1.21 – 1.13 (m, 3H), 0.98 – 0.90 (m, 2H).^{13}C NMR (125 MHz, Chloroform-*d*) δ 194.8 (d, *J* =45.0 Hz), 160.1(d, *J* =3.6 Hz), 145.3 (d, *J* =17.3 Hz), 130.5, 127.7 (d, *J* =35.6 Hz), 119.0 (d, *J* =6.7 Hz), 115.1, 69.0, 34.4 (d, *J* =10.3 Hz), 32.5 (d, *J* =23.3 Hz) 30.7 (d, *J* =8.5 Hz), 27.2 (d, *J* =8.3 Hz), 27.0 (d, *J* =14.4Hz), 26.3, 21.8. ^{31}P NMR (202 MHz, $CDCl_3$) δ -16.79. HRMS (ESI) calculated for [$C_{22}H_{34}O_2P$] $[M+H]^+$: 361.2291 found: 361.2287.

2.2.11 3-(benzyloxy)-2-(dicyclohexylphosphanyl)-benzaldehyde (Xu6-5)

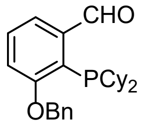

Xu6-5

To a solution of (2-(benzyloxy)-6-(1,3-dioxolan-2-yl)-phenyl)-dicyclohexylphosphane **Xu6-4** (2 mmol) in 2 mL THF, was added 3 mL HCl (1.0 M) under argon at 60 °C. The resulting solution was stirred 5 hours. The reaction mixture was quenched by the addition of $NaHCO_3$ (aq.) and diluted with EtOAc. The organic layer was separated, and the aqueous layer was extracted twice with EtOAc. The combined organic layers were dried over Na_2SO_4, filtered, concentrated and then was purified by flash column chromatography on silica gel (petroleum ether: ethyl acetate = 20: 1) to afford the crude product **Xu6-5** as a yellow solid. Mp: 85.2-87.2 °C. ^{1}H NMR (500 MHz, Chloroform-*d*) δ 11.27 (d, J = 8.9 Hz, 1H), 7.57 (dt, J = 7.4, 3.4 Hz, 1H), 7.50 – 7.38 (m, 6H), 7.14 (t, J = 6.1 Hz, 1H), 5.13 (d, J = 3.6 Hz, 2H), 2.39 (dp, J = 11.5, 3.9 Hz, 2H), 1.85 – 1.77 (m, 2H), 1.73 (dd, J = 9.1, 4.8 Hz, 2H), 1.66 – 1.57 (m, 4H), 1.37 – 1.31 (m, 2H), 1.26 – 1.05 (m, 8H), 0.98 (ddt, J = 12.1, 5.9, 3.3 Hz, 2H). ^{13}C NMR (125 MHz, Chloroform-*d*) δ 194.6 (d, J = 44.6 Hz), 161.6 (d, J = 3.6 Hz), 145.0 (d, J = 17.1 Hz), 136.2, 130.7, 128.7, 128.4, 128.1, 127.8, 119.9 (d, J = 6.6 Hz), 115.5, 70.9, 34.0 (d, J = 10.5 Hz), 32.3 (d, J = 22.8 Hz), 30.7 (d, J = 8.7 Hz), 27.0 (d, J =

8.7 Hz), 26.8 (d, J = 14.2 Hz), 26.3.^{31}P NMR (202 MHz, Chloroform-*d*) δ -16.41. HRMS (ESI) calculated for [$C_{26}H_{34}O_2P$] [M+H]$^+$: 409.2291 found: 409.2302.

2.2.12 2-(dicyclohexylphosphanyl)-3-(naphthalen-2-ylmethoxy)-benzaldehyde (Xu7-5)

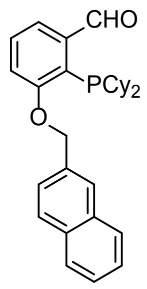

Xu7-5

Prepared from (2-(benzyloxy)-6-(1,3-dioxolan-2-yl)-phenyl)-dicyclohexylphosphane **Xu7-4** (2 mmol) in 2 mL THF, was added 3 mL HCl (1.0 M) under argon at 60 °C. The resulting solution was stirred 5 hours. The reaction mixture was quenched by the addition of $NaHCO_3$ (aq.) and diluted with EtOAc. The organic layer was separated, and the aqueous layer was extracted twice with EtOAc. The combined organic layers were dried over Na_2SO_4, filtered, concentrated and purified by flash column chromatography on silica gel (petroleum ether: ethyl acetate = 20: 1) to afford the crude product **Xu7-5** as a yellow solid. Mp: 107.1-110.2 °C. ^{1}H NMR (500 MHz, Chloroform-*d*) δ 11.27 (d, *J* = 8.7 Hz, 1H), 7.95 – 7.75 (m, 4H), 7.54 (ddd, *J* = 15.2, 6.9, 2.8 Hz, 4H), 7.41 (t, *J* = 8.0 Hz, 1H), 7.14 (d, *J* = 8.1 Hz, 1H), 5.27 (s, 2H), 2.42 (dtd, *J* = 11.6, 7.8, 3.6 Hz, 2H), 1.83 – 1.74 (m, 2H), 1.71 – 1.65 (m, 2H), 1.59 (t, *J* = 12.8 Hz, 4H), 1.39 – 1.32 (m, 2H), 1.24 – 1.06 (m, 8H), 1.01 – 0.92 (m, 2H). ^{13}C NMR (125 MHz, Chloroform-*d*) δ 194.6 (d, *J* = 44.8 Hz), 161.5 (d, *J* = 3.4 Hz), 145.0, 144.9, 133.7, 133.2, 133.1, 130.7, 128.5, 127.8, 127.7, 126.8, 126. 5, 126.3, 125.5, 119.9 (d, *J* = 6.5 Hz), 115.5, 70.9, 34.0 (d, *J* = 10.5 Hz), 32.3 (d, *J* = 23.0 Hz), 30.7 (d, *J* = 8.7 Hz), 27.0 (d, *J* = 8.6 Hz), 26.7 (d, *J* = 14.3 Hz), 26.2. ^{31}P NMR (202 MHz, Chloroform-*d*) δ -16.35. HRMS (ESI) calculated for [$C_{30}H_{36}O_2P$] [M+H]$^+$: 459.2447 found: 459.2455.

2.2.13 (*R*,*E*)-N-(2-(dicyclohexylphosphanyl)-3-isopropoxybenzylidene)-2-methylpropane-2-sulfinamide (Xu5-6)

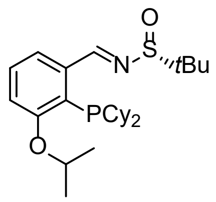

Xu5-6

Prepared from 2-(dicyclohexylphosphanyl)-3-isopropoxybenzaldehyde the crude product **Xu5-5** in 2 mL THF, was added (*R*)-2-methylpropane-2-sulfinamide (2.4 mmol, 1.2 equiv.) and titanium tetraisopropanolate (4 mmol, 2.0 equiv.) under argon at 50 °C. The resulting solution was stirred 8 hours. The reaction mixture was quenched by the addition of H_2O (aq.) and diluted with EtOAc. The solution was filtered and the residue was washed twice with EtOAc. The combined organic layers were dried over Na_2SO_4, filtered, concentratedand purified by flash column chromatography on silica gel (petroleum ether: ethyl acetate = 10: 1) to afford the crude product **Xu5-6** as a yellow solid. Mp: 102.1-104.2 °C. $[\alpha]_D^{20}$= -108.9 (*c* = 0.4, Chloroform). ^{1}H NMR (500 MHz, Chloroform-*d*) δ 9.86 (dd, *J* = 7.2, 1.9 Hz, 1H), 7.55 (dd, *J* = 7.6, 2.8 Hz, 1H), 7.35 (td, *J* = 8.0, 1.9 Hz, 1H), 6.89 (d, *J* = 8.0 Hz, 1H), 4.66 (pd, *J* = 6.1, 2.0 Hz, 1H), 2.52 – 2.38 (m, 2H), 1.93 – 1.82 (m, 2H), 1.78 – 1.75 (m, 2H), 1.59 (q, *J* = 10.7, 8.8 Hz, 4H), 1.40 (td, *J* = 4.9, 3.7, 2.0 Hz, 6H), 1.28 (dd, *J* = 9.3, 2.1 Hz, 15H), 1.19 – 1.08 (m, 4H), 0.97 – 0.89 (m, 2H). ^{13}C NMR (125 MHz, Chloroform-*d*) δ 165.0 (d, *J* = 41.2 Hz), 160.1 (d, *J* = 3.5 Hz), 143.8 (d, *J* = 21.8 Hz), 130.3,127.1 (d, *J* = 33.9 Hz), 119.6 (d, *J* = 6.3 Hz), 113.1, 68.8, 57.7, 34.6 (d, *J* = 11.5 Hz), 32.3 (dd, *J* = 23.5, 4.8 Hz), 30.5 (dd, *J* = 17.4, 8.7 Hz), 27.2 (dd, *J* = 8.4, 4.5 Hz), 27.0 (dd, *J* = 14.3, 7.8 Hz), 26.3, 22.7, 21.8 (d, *J* = 5.6 Hz). ^{31}P NMR (202 MHz, Chloroform-*d*) δ -12.13. HRMS (ESI) calculated for [$C_{26}H_{43}NO_2PS$] $[M+H]^+$: 464.2747 found: 464.2753.

2.2.14 (*R*, *E*)-N-(3-(benzyloxy)-2-(dicyclohexylphosphanyl)benzylidene)-2-methylpropane-2-sulfinamide (Xu6-6)

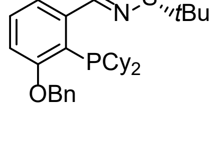

Xu6-6

To a solution of 3-(benzyloxy)-2-(dicyclohexylphosphanyl)-benzaldehyde the crude product **Xu6-5** in 2 mL THF, was added (*R*)-2-methylpropane-2-sulfinamide (2.4 mmol, 1.2 equiv.) and titanium tetraisopropanolate (4 mmol, 2.0 equiv.) under argon at 50 °C. The resulting solution was stirred 8 hours. The reaction mixture was quenched by the addition of H_2O (aq.) and diluted with EtOAc. The solution was filtered and the residue was washed twice with EtOAc. The combined organic layers were dried over Na_2SO_4, filtered, concentrated and then was purified by flash column chromatography on silica gel (petroleum ether: ethyl acetate = 10: 1) to afford the crude product **Xu6-6** as a yellow solid. Mp: 48.1-52.0 °C. $[\alpha]_D^{20}$= -110.0 (*c* = 0.4, Chloroform). ^{1}H NMR (400 MHz, Chloroform-*d*) δ 9.85 (d, J = 7.2 Hz, 1H), 7.63 (ddd, J = 7.8, 3.1, 1.0 Hz, 1H), 7.48 – 7.33 (m, 6H), 7.05 – 6.96 (m, 1H), 5.08 (s, 2H), 2.32 (tt, J = 7.7, 3.4 Hz, 2H), 1.78 – 1.66 (m, 4H), 1.56 (d, J = 8.6 Hz, 4H), 1.27 (s, 9H), 1.20 – 1.04 (m, 8H), 0.97 – 0.81 (m, 4H). ^{13}C NMR (100 MHz, Chloroform-*d*) δ 164.8 (d, *J* = 40.5 Hz), 161.6 (d, *J* = 3.4 Hz), 143.5 (d, *J* = 21.2 Hz), 136.4, 130.4, 128.6, 128.3,127.3 (d, *J* = 35.0 Hz), 120.5 (d, *J* = 6.2 Hz), 113.5, 70.7, 57.8, 34.1 (dd, *J* = 11.6, 5.3 Hz), 32.2, 32.0 (d, *J* = 1.9 Hz), 30.5 (dd, *J* = 13.1, 8.9 Hz), 29.7, 27.2 – 26.6 (m) 26.3, 22.7. ^{31}P NMR (162 MHz, Chloroform-*d*) δ -11.79. HRMS (ESI) calculated for [$C_{30}H_{43}NO_2PS$] [M+H]$^+$: 512.2747 found: 512.2757.

2.2.15 (*R*, *E*)-N-(2-(dicyclohexylphosphanyl)-3-(naphthalen-2-ylmethoxy)-benzylidenee)-2-methylpropane-2-sulfinamide (Xu7-6)

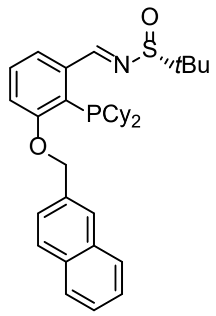

Xu7-6

Prepared from 2-(dicyclohexylphosphanyl)-3-(naphthalen-2-ylmethoxy)-benzaldehyde the crude product **Xu7-5** in 2 mL THF, was added (*R*)-2-methylpropane-2-sulfinamide (2.4 mmol, 1.2 equiv.) and titanium tetraisopropanolate (4 mmol, 2.0 equiv.) under argon at 50 °C. The resulting solution was stirred 8 hours. The reaction mixture was quenched by the addition of H_2O (aq.) and diluted

with EtOAc. The solution was filtered and the residue was washed twice with EtOAc. The combined organic layers were dried over Na_2SO_4, filtered, concentratedand purified by flash column chromatography on silica gel (petroleum ether: ethyl acetate = 10: 1) to afford the crude product **Xu7-6** as a yellow solid. Mp: 80-82.1 °C. $[\alpha]_D^{20}$ = -77.1 (*c* = 0.4, Chloroform). ^{1}H NMR (400 MHz, Chloroform-*d*) δ 9.86 (d, *J* = 7.2 Hz, 1H), 7.92 – 7.87 (m, 3H), 7.84 (dt, *J* = 6.2, 3.5 Hz, 1H), 7.64 (ddd, *J* = 7.8, 3.1, 1.0 Hz, 1H), 7.53 (ddd, *J* = 10.8, 7.4, 2.5 Hz, 3H), 7.37 (t, *J* = 8.0 Hz, 1H), 7.07 – 7.02 (m, 1H), 5.26 (s, 2H), 2.46 – 2.32 (m, 2H), 1.78 – 1.70 (m, 2H), 1.69 – 1.63 (m, 2H), 1.62 – 1.53 (m, 4H), 1.38 – 1.29 (m, 3H), 1.27 (s, 10H), 1.11 (q, *J* = 8.3, 7.4 Hz, 6H), 1.02 – 0.90 (m, 2H). ^{13}C NMR (100 MHz, Chloroform-*d*) δ 164.7 (d, *J* = 40.3 Hz), 161.6 (d, *J* = 3.3 Hz), 143.6, 143.4, 133.9, 133.3, 133.1, 130.5, 128.4, 127.8, 127.8, 126.7, 126.4, 126.2, 125.5, 120.6 (d, *J* = 6.1 Hz), 113.7, 70.7, 57.8, 34.2 (dd, *J* = 11.4, 5.0 Hz), 32.0 (d, *J* = 2.2 Hz), 30.6 (dd, *J* = 12.9, 8.9 Hz), 27.1 (dd, *J* = 8.6, 2.7 Hz), 26.8 (dd, *J* = 14.1, 4.4 Hz), 26.3, 22.7. ^{31}P NMR (162 MHz, Chloroform-*d*) δ -11.66. HRMS (ESI) calculated for $[C_{34}H_{45}NO_2PS]$ $[M+H]^+$: 562.2903 found: 562.2908.

2.2.16 (*R*)-N-((*S*)-[1,1'-biphenyl]-4-yl(3-(benzyloxy)-2-(dicyclohexylphosphanyl)-phenyl)-methyl)-2-methylpropane-2-sulfinamide (Xu5-7)

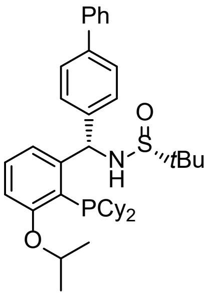

Xu5-7

Prepared from (*R*,*E*)-N-(2-(dicyclohexylphosphanyl)-3-isopropoxybenzylidene)-2- methylpropane-2-sulfinamide the crude product **Xu5-6** in 2 mL anhydrous THF, was added [1,1'-biphenyl]-4-ylmagnesium bromide (4 mmol, 2.0 equiv.) dropwise under argon at -50 °C. The reaction mixture was warmed to room temperature overnight and was quenched by the addition of NH_4Cl (aq.) and diluted with EtOAc. The organic layer was separated, and the aqueous layer was extracted twice with EtOAc. The combined organic layers were dried over Na_2SO_4, filtered,

concentrated.and purified by flash column chromatography on silica gel (petroleum ether: ethyl acetate = 5: 1) to afford the product **Xu5-7** as a white solid (507 mg, 41% yield). Mp: 182.3-185.0 °C. $[\alpha]_D^{20}$ = -99.7 (*c* = 0.4, Chloroform). ^{1}H NMR (500 MHz, Chloroform-*d*) δ 7.57 – 7.52 (m, 2 H), 7.51 – 7.47 (m, 4 H), 7.46 – 7.39 (m, 2 H), 7.38 – 7.29 (m, 2 H), 7.28 – 7.21 (m, 1 H), 7.01 (s, 1 H), 6.72 (d, *J* = 8.1 Hz, 1 H), 4.69 – 4.53 (m, 1 H), 4.08 (s, 1 H), 2.45 – 2.27 (m, 2 H), 1.94 – 1.83 (m, 2 H), 1.81 – 1.73 (m, 1 H), 1.72 – 1.66 (m, 1 H), 1.65 – 1.56 (m, 2 H), 1.55 – 1.47 (m, 1 H), 1.40 – 1.34 (m, 7 H), 1.30 – 1.22 (m, 13 H), 1.21 – 1.09 (m, 4 H)，1.03 – 0.89 (m, 4 H). ^{13}C NMR (125 MHz, Chloroform-*d*) δ 160.0 (d, *J* = 3.4 Hz), 151.3 (d, *J* = 23.7 Hz), 142.3, 141.0, 139.9, 130.1, 129.1, 129.1, 128.6, 127.1, 127.0, 126.9, 123.0 (d, *J* = 29.2 Hz), 119.8 (d, *J* = 6.7 Hz), 109.1, 68.4, 60.4 (d, *J* = 35.8 Hz), 55.8, 35.6 (d, *J* = 10.4 Hz), 34.8 (d, *J* = 11.2 Hz), 33.4 (d, *J* = 26.9 Hz), 32.5 (d, *J* = 23.8 Hz), 30.3 (d, *J* = 9.6 Hz), 30.0 (d, *J* = 6.9 Hz), 27.4 (dd, *J* = 8.2, 3.3 Hz), 27.1 (dd, *J* = 14.7, 9.9 Hz), 26.2 (d, *J* = 3.6 Hz), 22.7, 21.9, 21.8. ^{31}P NMR (202 MHz, Chloroform-*d*) δ -10.70. HRMS (ESI) calculated for [$C_{38}H_{53}NO_2PS$] $[M+H]^+$: 618.3529 found: 618.3526.

2.2.17 (*R*)-N-((*S*)-[1,1'-biphenyl]-4-yl(3-(benzyloxy)-2-(dicyclohexylphosphanyl)-phenyl)-methyl)-2-methylpropane-2-sulfinamide (Xu6-7)

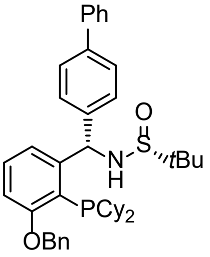

Xu6-7

To a solution of (*R*, *E*)-N-(3-(benzyloxy)-2-(dicyclohexylphosphanyl)-benzylidene)-2-methyl-propane-2-sulfinamide the crude product **Xu6-6** in 2 mL anhydrous THF, was added [1,1'-biphenyl]-4-ylmagnesium bromide (4 mmol, 2.0 equiv.) dropwise under argon at -50 °C. The reaction mixture was warmed to room temperature overnight and was quenched by the addition of NH_4Cl (aq.) and diluted with EtOAc. The organic layer was separated, and the aqueous layer was extracted twice with EtOAc. The combined organic layers were dried over Na_2SO_4, filtered, concentrated. The crude product was purified by flash column chromatography on silica gel

(petroleum ether: ethyl acetate = 5: 1) to afford the product **Xu6-7** as a white solid (573 mg, 43% yield). Mp: 94.1-96.0 °C. $[\alpha]_D^{20}$ = -101.7 (*c* = 0.4, Chloroform). ^{1}H NMR (500 MHz, Chloroform-*d*) δ 7.5 8 – 7.51 (m, 2 H), 7.49 (s, 4 H), 7.46 – 7.35 (m, 8 H), 7.36 – 7.29 (m, 2 H), 7.02 (s, 1 H), 6.84 (d, *J* = 8.1 Hz, 1 H), 5.10 – 4.99 (m, 2 H), 4.04 (s, 1 H), 2.36 – 2.11 (m, 2 H), 1.74 – 1.65 (m, 3 H), 1.64 – 1.53 (m, 3 H), 1.52 – 1.42 (m, 1 H), 1.38 – 1.31 (m, 1 H), 1.31 – 1.22 (m, 11 H), 1.21 – 1.88 (m, 10 H). ^{13}C NMR (125 MHz, Chloroform-*d*) δ 161.5 (d, *J* = 3.6 Hz), 151.2 (d, *J* = 23.5 H z), 142.2, 141.0, 140.1, 136.7, 130.3, 129.19, 129.17, 128.7, 128.5, 128.1, 128.0, 127.18, 127.11, 127.0, 123.5 (d, *J* = 30.3 Hz), 120.9 (d, *J* = 6.5 Hz), 109.6, 70.5, 60.4 (d, *J* = 35.5 Hz), 55.9, 35.1 (d, *J* = 10.4 Hz), 34.2 (d, *J* = 11.2 Hz), 33.2 (d, *J* = 26.4 Hz), 32.3 (d, *J* = 22.9 Hz), 30.5 (d, *J* = 10.0 Hz), 30.2 (d, *J* = 7.4 Hz), 27.3 (t, *J* = 8.5 Hz), 27.0 (dd, *J* = 14.2, 11.7 H z), 26.3, 22.8. ^{31}P NMR (202 MHz, Chloroform-*d*) δ -10.35. HRMS (ESI) calculated for [$C_{42}H_{53}NO_2PS$] $[M+H]^+$: 666.3529 found: 666.3526.

2.2.18 (*R*)-N-((*S*)-[1,1'-biphenyl]-4-yl(3-(benzyloxy)-2-(dicyclohexylphosphanyl)-phenyl)-methyl)-2-methylpropane-2-sulfinamide (Xu7-7)

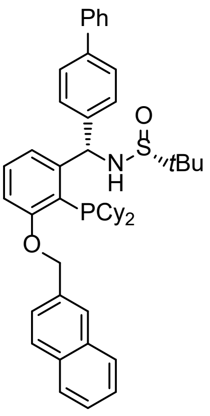

Xu7-7

Prepared from (*R*,*E*)-N-(2-(dicyclohexylphosphanyl)-3-(naphthalen-2-ylmethoxy)benzylidene)-2-methylpropane-2-sulfinamide the crude product **Xu7-6** in 2 mL anhydrous THF, was added [1,1'-biphenyl]-4-ylmagnesium bromide (4 mmol, 2.0 equiv.) dropwise under argon at -50 °C. The reaction mixture was warmed to room temperature overnight and was quenched by the addition of NH_4Cl (aq.) and diluted with EtOAc. The organic layer was separated, and the aqueous layer was extracted twice with EtOAc. The combined organic layers were dried over Na_2SO_4, filtered,

concentrated and purified by flash silica gel column chromatography (petroleum ether: ethyl acetate = 5: 1) to afford the product **Xu7-7** as a white solid (659 mg, 46% yield). Mp: 95.2-98.3 °C. $[\alpha]_D^{20}$ = -80.2 (*c* = 0.4, Chloroform). ^{1}H NMR (500 MHz, Chloroform-*d*) δ 7.93 – 7.87 (m, 3 H), 7.86 – 7.82 (m, 1 H), 7.57 – 7.56 (m, 1 H), 7.56 – 7.54 (m, 2 H), 7.54 – 7.53 (m, 1 H), 7.53 – 7.50 (m, 5 H), 7.46 – 7.37 (m, 3 H), 7.37 – 7.31 (m, 2 H), 7.06 (s, 1 H), 6.89 (d, *J* = 7.9 Hz, 1 H), 5.33 – 5.18 (m, 2 H), 4.06 (s, 1 H), 2.44 – 2.21 (m, 2 H), 1.81 – 1.63 (m, 4 H), 1.63 – 1.53 (m, 2 H), 1.53 – 1.44 (m, 1 H), 1.43 – 1.31 (m, 3 H), 1.31 – 1.25 (m, 10 H), 1.22 – 1.14 (m, 2 H), 1.12 – 0.95 (m, 6 H), 0.94 – 0.87 (m, 1 H). ^{13}C NMR (125 MHz, Chloroform-*d*) δ 161.5 (d, *J* = 3.3 Hz), 151.1 (d, *J* = 23.4 Hz), 142.1, 140.9, 140.0, 134.2, 133.2, 133.0, 130.3, 129.1, 129.1, 128.6, 128.3, 127.8, 127.7, 127.1, 127.03, 127.00, 126.5, 126.3, 126.1, 125.5, 123.5 (d, *J* = 30.2 Hz), 120.9 (d, *J* = 5.2 H z), 109.7, 70.5, 60.3 (d, *J* = 40.4 Hz), 55.9, 35.1 (d, *J* = 10.4 Hz), 34.2 (d, *J* = 11.1 Hz), 33.2 (d, *J* = 26.4 Hz), 32.3 (d, *J* = 22.9 Hz), 30.4 (d, *J* = 10.0 Hz), 30.2 (d, *J* = 7.4 Hz), 27.3 (t, *J* = 8.3 Hz), 26.9 (dd, *J* = 14.5, 12.3 Hz), 26.2, 22.8. ^{31}P NMR (202 MHz, Chloroform-*d*) δ -10.19. HR MS (ESI) calculated for [$C_{46}H_{55}NO_2PS$] $[M+H]^+$: 716.3686 found: 716.3689.

2.2.19 (*R*)-N-((*S*)-[1,1'-biphenyl]-4-yl(2-(dicyclohexylphosphanyl)-3-isopropoxyphenyl)-methyl)-N,2-dimethylpropane-2-sulfinamide (Xu5)

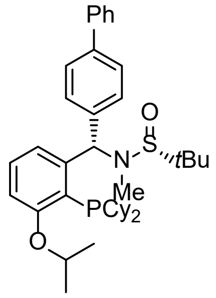

Xu5

Prepared from (*R*)-N-((*S*)-[1,1'-biphenyl]-4-yl(3-(benzyloxy)-2-(dicyclohexylphosphanyl)phenyl)-methyl)-2-methylpropane-2-sulfinamide **Xu5-7** (0.5 mmol) in 2 mL anhydrous THF, was added *n*-BuLi (0.75 mmol, 1.6 M in hexane) dropwise under argon at -40 °C. The resulting solution at this temperature during 1 hour and iodomethane (1 mmol, 2 equiv.) was added. After 1 hour, the reaction mixture moved to 0 °C and stirred 1 hour. The reaction mixture was the addition of NH_4Cl

(aq.) and diluted with EtOAc. The organic layer was separated, and the aqueous layer was extracted twice with EtOAc. The combined organic layers were dried over Na_2SO_4, filtered, concentrated and purified by flash column chromatography on silica gel (petroleum ether: ethyl acetate = 5: 1) to afford the product **Xu6** as a white solid (234 mg, 74% yield). Mp: 85.7-87.2 °C. $[\alpha]_D^{20}$ = -1.2 (*c* = 0.4, Chloroform). ^{1}H NMR (500 MHz, Chloroform-*d*) δ 7.60 – 7.52 (m, 2 H), 7.50 – 7.44 (m, 2 H), 7.43 – 7.36 (m, 4 H), 7.33 – 7.29 (m, 1 H), 7.29 – 7.23 (m, 2 H),7.15 (d, *J* = 11.7 Hz, 1 H), 6.72 (dd, *J* = 7.0, 2.4 Hz, 1 H), 4.69 – 4.55 (m, 1 H), 2.64 (s, 3 H), 2.55 – 2.41 (m, 1 H), 2.22 – 2.09 (m, 1 H), 1.90 – 1.71 (m, 3 H), 1.70 – 1.54 (m, 3 H), 1.43 – 1.33 (m, 7 H), 1.31 – 1.21 (m, 5 H), 1.20 – 0.99 (m, 13 H), 0.99 – 0.72 (m, 4 H). ^{13}C NMR (125 MHz, Chloroform-*d*) δ 160.0 (d, *J* = 3.6 Hz), 150.0 (d, *J* = 22.8 Hz), 140.9, 139.8, 139.2, 131.7, 130.2, 128.6, 127.0, 126.9, 126.3, 122.8 (d, *J* = 28.5 Hz), 119.5 (d, *J* = 5.4 Hz), 109.1, 70.5 (d, *J* = 40.5 Hz), 68.3, 58.6, 35.4 (d, *J* = 10.0 Hz), 34.4 (d, *J* = 11.2 Hz), 33.5 (d, *J* = 27.1 Hz), 32.3 (d, *J* = 23.1 Hz), 30.7 (d, *J* = 10.7 Hz), 30.3, 29.6 (d, *J* = 6.7 Hz), 27.3 (dd, *J* = 8.4, 3.4 Hz), 27.0 (dd, *J* = 14.5, 9.3 Hz), 26.4, 26.1, 24.1, 21.87, 21.83. ^{31}P NMR (202 MHz, Chloroform-*d*) δ -10.40. HRMS (ESI) calculated for $[C_{39}H_{55}NO_2PS]$ $[M+H]^+$: 632.3686 found: 632.3684.

2.2.20 ((*R*)-N-((*S*)-[1,1'-biphenyl]-4-yl(3-(benzyloxy)-2-(dicyclohexylphosphanyl)-phenyl)-methyl)-N,2-dimethylpropane-2-sulfinamide (Xu6)

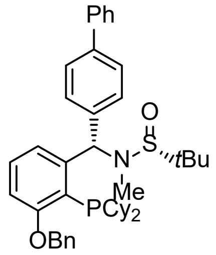

Xu6

To a solution of (*R*)-N-((*S*)-[1,1'-biphenyl]-4-yl(3-(benzyloxy)-2-(dicyclohexylphosphanyl)phenyl) methyl)-2-methylpropane-2-sulfinamide **Xu6-7** (0.5 mmol) in 2 mL anhydrous THF, was added *n*-BuLi (0.75 mmol, 1.6 M in hexane) dropwise under argon at -40 °C. The resulting solution at this temperature during 1 hour and iodomethane (1 mmol, 2 equiv.) was added. After 1 hour, the reaction mixture moved to 0 °C and stirred 1 hour. The reaction mixture was the addition of NH_4Cl

(aq.) and diluted with EtOAc. The organic layer was separated, and the aqueous layer was extracted twice with EtOAc. The combined organic layers were dried over Na_2SO_4, filtered, concentrated. The crude product was purified by flash column chromatography on silica gel (petroleum ether: ethyl acetate = 5: 1) to afford the product **Xu6** as a white solid (245 mg, 72% yield). Mp: 79.1-81.3 °C$[\alpha]_D^{20}$ = -26.1 (*c* = 0.4, Chloroform).. ^{1}H NMR (500 MHz, Chloroform-*d*) δ 7.57 – 7.52 (m, 2 H), 7.51 – 7.44 (m, 3 H), 7.43 (s, 1 H), 7.42 – 7.39 (m, 5 H), 7.39 – 7.34 (m, 1 H), 7.34-7.30 (m, 1 H), 7.29 – 7.23 (m, 3 H), 7.14 (d, *J* = 11.6 Hz, 1 H), 6.85 (d, *J* = 8.2, 1.0 Hz, 1 H), 5.03 (d, *J* = 2.3 Hz, 2 H), 2.64 (s, 3 H), 2.40 – 2.29 (m, 1 H), 2.07 – 1.95 (m, 1 H), 1.74 – 1.65 (m, 2 H), 1.64 – 1.56 (m, 3 H), 1.54 – 1.46 (m, 1 H), 1.41 – 1.34 (m, 1 H), 1.32 – 1.15 (m, 5 H), 1.14 – 1.10 (m, 12 H), 0.99 – 0.80 (m, 4 H), 0.79 – 0.70 (m, 1 H). ^{13}C NMR (125 MHz, Chloroform-*d*) δ 161.5 (d, *J* = 3.6 Hz), 149.9 (d, *J* = 22.5 Hz), 140.9, 139.9, 139.1, 136.7, 131.7, 130.4, 128.6, 128.4, 128.1, 128.0, 127.1, 127.0, 126.4, 123.3 (d, *J* = 29.6 Hz), 120.6 (d, *J* = 5.3 Hz), 109.6, 70.6 (d, *J* = 39.6 Hz), 70.4, 58.6, 34.9 (d, *J* = 10.0 Hz), 33.9 (d, *J* = 11.3 Hz), 33.2 (d, *J* = 26.4 Hz), 32.1 (d, *J* = 22.5 Hz), 30.8 (d, *J* = 11.3 Hz), 30.4, 29.8 (d, *J* = 7.5 Hz), 27.2 (dd, *J* = 8.5, 6.5 Hz), 26.9 (d, *J* = 2.9 Hz), 26.8 (d, *J* = 4.9 Hz), 26.4, 26.1, 24.1. ^{31}P NMR (202 MHz, Chloroform-*d*) δ -9.99. HRMS (ESI) calculated for $[C_{43}H_{55}NO_2PS]$ $[M+H]^+$: 680.3686 found: 680.3689.

2.2.21 ***(R)*-N-((*S*)-[1,1'-biphenyl]-4-yl(2-(dicyclohexylphosphanyl)-3-(naphthalen-2-ylmeth-oxy)-phenyl)-methyl)-N,2-dimethylpropane-2-sulfinamide** (**Xu7**)

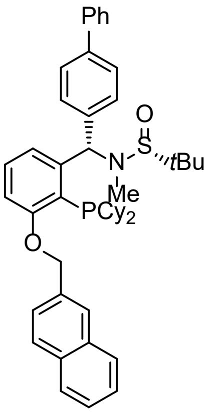

Xu7

Prepared from (*R*)-N-((*S*)-[1,1'-biphenyl]-4-yl(3-(benzyloxy)-2-(dicyclohexylphosphanyl)phenyl)-methyl)-2-methylpropane-2-sulfinamide **Xu7-7** (0.5 mmol) in 2 mL anhydrous THF, was added

n-BuLi (0.75 mmol, 1.6 M in hexane) dropwise under argon at -40 °C. The resulting solution at this temperature during 1 hour and iodomethane (1 mmol, 2 equiv.) was added. After 1 hour, the reaction mixture moved to 0 °C and stirred 1 hour. The reaction mixture was the addition of NH_4Cl (aq.) and diluted with EtOAc. The organic layer was separated, and the aqueous layer was extracted twice with EtOAc. The combined organic layers were dried over Na_2SO_4, filtered, concentrated and purified by flash column chromatography on silica gel (petroleum ether: ethyl acetate = 5: 1) to afford the product **Xu7** as a white solid (277 mg, 76% yield). Mp: 89.1-91.3 °C. $[\alpha]_D^{20}$ = -44.34 (*c* = 0.4, Chloroform). ^{1}H NMR (500 MHz, Chloroform-*d*) δ 7.95 – 7.85 (m, 3 H), 7.85 – 7.80 (m, 1 H), 7.61 – 7.52 (m, 3 H), 7.52 – 7.46 (m, 5 H), 7.46 – 7.39 (m, 3 H), 7.36 – 7.29 (m, 1 H), 7.30 – 7.24 (m, 2 H), 7.16 (d, *J* = 11.6 Hz, 1 H), 6.90 (d, *J* = 8.1, 1 H), 5.21 (d, *J* = 2.1 Hz, 2 H), 2.66 (s, 3 H), 2.51 – 2.33 (m, 1 H), 2.17 – 2.02 (m, 1 H), 1.74 – 1.54 (m, 5 H), 1.53 – 1.42 (m, 1 H), 1.41 – 1.32 (m, 2 H), 1.29 – 1.04 (m, 16 H), 0.97 – 0.84 (m, 4 H), 0.81 – 0.70 (m, 1 H). ^{13}C NMR (125MHz, Chloroform-*d*) δ 161.6 (d, *J* = 3.6 Hz), 149.9 (d, *J* = 22.7 Hz), 140.9, 139.9, 139.1, 134.2, 133.2, 133.0, 131.7, 130.5, 128.6, 128.2, 127.7, 127.7, 127.1, 127.0, 126.6, 126.4, 126.3, 126.1, 125.6, 123.3 (d, *J* = 29.5 Hz), 120.6 (d, *J* = 5.4 Hz), 109.7, 70.6 (d, *J* = 39.9 Hz), 70.5, 58.6, 35.0 (d, *J* = 10.0 Hz), 33.9 (d, *J* = 11.3 Hz), 33.2 (d, *J* = 26.5 Hz), 32.1 (d, *J* = 22.6 Hz), 30.8 (d, *J* = 11.2 Hz), 30.4, 29.8 (d, *J* = 7.3 Hz), 27.2 (dd, *J* = 8.6, 5.5 Hz), 26.8 (dd, *J* = 14.5, 3.2 Hz), 26.3, 26.1, 24.1. ^{31}P NMR (202 MHz, Chloroform-*d*) δ -10.19. HRMS (ESI) calculated for [$C_{47}H_{57}NO_2PS$] $[M+H]^+$: 730.3842 found: 730.3847.

2.3 Synthesis of Xu8, Xu9, (*R*, *R*$_S$)-Xu9

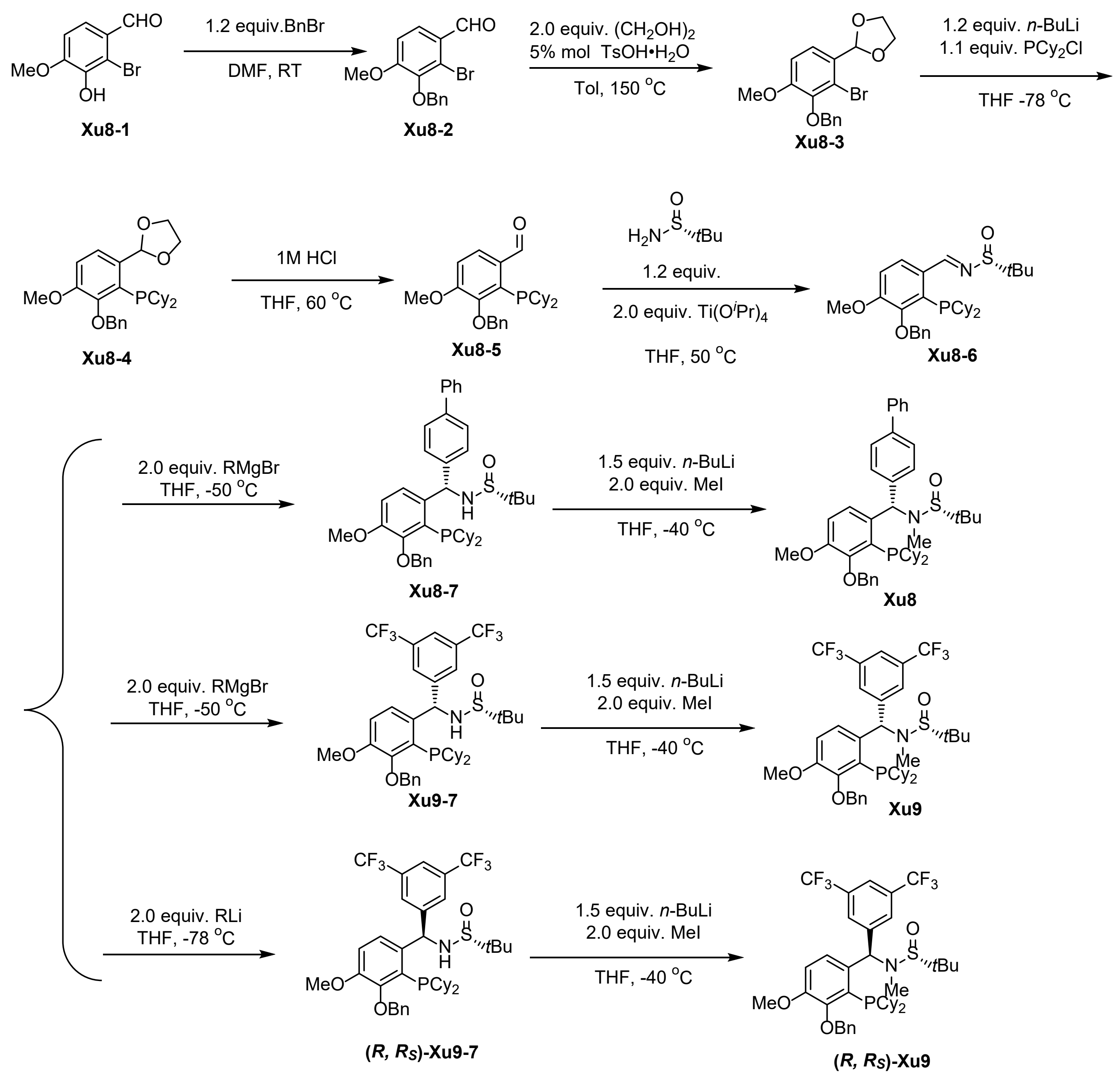

Prepared from 2-bromo-3-hydroxy-4-methoxybenzaldehyde, according to the preparation of **Xu5**

2.3.1 3-(benzyloxy)-2-bromo-4-methoxybenzaldehyde (Xu8-2)

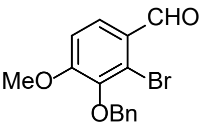

Xu8-2

Prepared from 2-bromo-3-hydroxy-4-methoxybenzaldehyde (30 mmol) in DMF (50 mL) was added benzyl bromide (36mmol, 1.2 equiv.) and K_2CO_3 (45 mmol, 1.5 equiv.). The resulting

solution was stirred at room temperature overnight. The reaction mixture was quenched by the addition of H_2O and diluted with EtOAc. The organic layer was separated, and the aqueous layer was extracted twice with EtOAc. The combined organic layers were dried over Na_2SO_4, filtered, concentrated. The crude product was then washed by petroleum ether with a little ethyl acetate to afford the product **Xu8-2** as a white solid (8.4 g, 87% yield). Mp: 72.2-74.5 °C. ^{1}H NMR (400 MHz, Chloroform-*d*) δ 10.25 (d, *J* = 0.9 Hz, 1H), 7.74 (d, *J* = 8.7 Hz, 1H), 7.57 – 7.48 (m, 2H), 7.43 – 7.28 (m, 3H), 6.96 (d, *J* = 8.7 Hz, 1H), 5.04 (s, 2H), 3.94 (s, 3H). ^{13}C NMR (100 MHz, Chloroform-*d*) δ 190.9, 158. 7, 145.0, 136.5, 128.4, 128.3, 128.2, 127.3, 126.5, 123.4, 110.9, 74.7, 56.2. HRMS (ESI) calculated for [$C_{15}H_{13}BrNaO_3$] $[M+Na]^+$: 342.9940 found: 342.9930.

2.3.2 3-(benzyloxy)-2-bromo-4-methoxybenzaldehyde (Xu8-3)

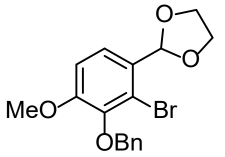

Xu8-3

Prepared from 3-(benzyloxy)-2-bromo-4-methoxybenzaldehyde **(Xu8-2)** (20 mmol) in 50 mL toluene, was added ethylene glycol (40 mmol, 2.0 equiv.) and *p*-toluenesulfonic acid (1 mmol, 5% mol). The resulting solution was stirred 18 hours at 150 °C. The reaction mixture was washed by the addition of H_2O and the combined organic layers were dried over Na_2SO_4, filtered, concentrated, The crude product was purified by flash column chromatography on silica gel (petroleum ether: ethyl acetate = 20: 1) to afford the product **Xu8-3** as a white liquid (3.9 g, 53% yield). Mp: 106.2-108.5 °C. ^{1}H NMR (400 MHz, Chloroform-*d*) δ 7.60 – 7.51 (m, 2 H), 7.44 – 7.28 (m, 4 H), 6.89 (d, *J* = 8.6 Hz, 1 H), 6.07 (s, 1 H), 5.00 (s, 1 H), 4.16 – 4.09 (m, 2 H), 4.09 – 4.01 (m, 2 H), 3.86 (s, 2 H). ^{13}C NMR (100 MHz, Chloroform-*d*) δ 154.3, 145.1, 137.0, 129.4, 128.3, 128.2, 127.9, 123.0, 119.1, 111.0, 102.6, 74.5, 65.3, 56.0. HRMS (ESI) calculated for [$C_{17}H_{17}BrNaO_4$] $[M+Na]^+$: 387.0202 found: 387.0205.

2.3.3 3-(benzyloxy)-2-bromo-4-methoxybenzaldehyde (Xu8-4)

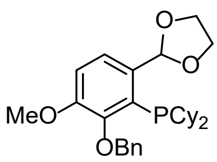

Xu8-4

Prepared from 3-(benzyloxy)-2-bromo-4-methoxybenzaldehyde **(Xu8-3)** (5 mmol) in 20 mL anhydrous THF, was added *n*-BuLi (18 mmol, 1.6 M in hexane) dropwise under argon at -78 °C, The resulting solution at this temperature during 1 hour, and dicyclohexylchlorophosphine (17 mmol, 1.1 equiv.) was added dropwise. The reaction mixture was warmed to room temperature overnight. The re action mixture was quenched by the addition of NH_4Cl (aq.) and diluted with EtOAc. The organic layer was separated, and the aqueous layer was extracted twice with EtOAc. The combined organic layers were dried over Na_2SO_4, filtered, concentrated. The crude product was purified by flash column chromatography on silica gel (petroleum ether: ethyl acetate = 20: 1) to afford the crude product **Xu8-4** as a white liquid. Mp: 96.2-98.8 °C. ^{1}H NMR (400 MHz, Chloroform-*d*) δ 7.46 (d, J = 7.5 Hz, 2H), 7.37 (td, J = 6.9, 6.5, 3.8 Hz, 2H), 7.33 – 7.29 (m, 1H), 7.26 (d, J = 3.1 Hz, 1H), 6.98 (d, J = 2.4 Hz, 1H), 6.59 (d, J = 7.3 Hz, 1H), 5.14 (s, 2H), 4.11 (q, J = 3.5, 2.6 Hz, 2H), 4.01 (dd, J = 4.6, 2.7 Hz, 2H), 3.88 (s, 3H), 1.94 – 1.84 (m, 4H), 1.78 (d, J = 12.9 Hz, 2H), 1.71 – 1.61 (m, 4H), 1.58 – 1.50 (m, 2H), 1.34 – 1.14 (m, 8H), 1.06 (td, J = 12.1, 11.6, 4.0 Hz, 2H).^{13}C NMR (100 MHz, Chloroform-*d*) δ 149.3, 149.2, 137.2 (d, *J* = 21.7 Hz), 136.8, 128.4, 127.9, 127.6, 126.5 (d, *J* = 23.1 Hz), 115.1, 110.8 (d, *J* = 6.7 Hz), 101.0 (d, *J* = 33.0 Hz), 70.7, 65.3, 56.2, 34.2 (d, *J* = 12.1 Hz), 30.5 (d, *J* = 17.7 Hz), 29.1 (d, *J* = 8.0 Hz), 27.1 (d, *J* = 9.0 Hz), 27.0 (d, *J* = 4.3 Hz), 26.3. ^{31}P NMR (162 MHz, Chloroform-*d*) δ -17.48. HRMS (ESI) calculated for [$C_{29}H_{40}O_4P$] $[M+Na]^+$: 483.2659 found: 483.2672.

2.3.4 3-(benzyloxy)-2-bromo-4-methoxybenzaldehyde (Xu8-5)

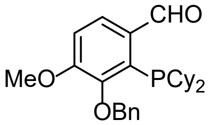

Xu8-5

Prepared from 3-(benzyloxy)-2-bromo-4-methoxybenzaldehyde the crude product **(Xu8-4)** in 2 mL THF, was added 3 mL HCl (1.0 M) under argon at 60 °C. The resulting solution was stirred 5 hours.

The reaction mixture was quenched by the addition of $NaHCO_3$ (aq.) and diluted with EtOAc. The organic layer was separated, and the aqueous layer was extracted twice with EtOAc. The combined organic layers were dried over Na_2SO_4, filtered, concentrated and purified by flash column chromatography on silica gel (petroleum ether: ethyl acetate = 20: 1) to afford the crude product **Xu8-5** as a yellow solid. Mp: 95.5-97.2 °C. ^1H NMR (400 MHz, Chloroform-*d*) δ 11.07 (d, *J* = 8.7 Hz, 1H), 7.78 (dd, *J* = 8.6, 3.2 Hz, 1H), 7.54 – 7.46 (m, 2H), 7.46 – 7.40 (m, 2H), 7.39 – 7.32 (m, 1H), 7.07 (d, *J* = 8.6 Hz, 1H), 5.20 (s, 2H), 3.92 (s, 3H), 2.36 (tdt, *J* = 11.4, 7.4, 3.3 Hz, 2H), 1.86 – 1.77 (m, 2H), 1.75 – 1.66 (m, 2H), 1.64 – 1.52 (m, 4H), 1.39 – 1.32 (m, 2H), 1.26 – 1.09 (m, 8H), 1.04 – 0.91 (m, 2H). ^{13}C NMR (100 MHz, Chloroform-*d*) δ 193.4 (d, *J* = 42.1 Hz), 156.0, 150.6, 138.0, 136.7, 133.4 (d, *J* = 37.7 Hz), 128.3, 127.7, 127.3, 124.4 (d, *J* = 6.2 Hz), 113.3, 73.9, 55.7, 34.5 (d, *J* = 11.6 Hz), 32.4 (d, *J* = 23.2 Hz), 30.8 (d, *J* = 9.7 Hz), 26.8 (d, *J* = 8.9 Hz), 26.6 (d, *J* = 14.0 Hz), 26.2 (d, *J* = 1.4 Hz). ^{31}P NMR (162 MHz, Chloroform-*d*) δ -13.94. HRMS (ESI) calculated for [$C_{27}H_{36}O_3P$] $[M+H]^+$: 439.2397 found: 439.2407.

2.3.5 (*R*, *E*)-N-(3-(benzyloxy)-2-(dicyclohexylphosphanyl)-4-methoxybenzylidene)-2-methylpropane-2-sulfinamide (Xu8-6)

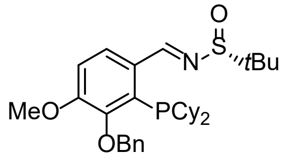

Xu8-6

Prepared from 3-(benzyloxy)-2-bromo-4-methoxybenzaldehyde the crude product **Xu8-5** in 2 mL THF, was added (*R*)-2-methylpropane-2-sulfinamide (2.4 mmol, 1.2 equiv.) and titanium tetraisopropanolate (4 mmol, 2.0 equiv.) under argon at 50 °C. The resulting solution was stirred 8 hours. The reaction mixture was quenched by the addition of H_2O (aq.) and diluted with EtOAc. The solution was filtered and the residue was washed twice with EtOAc. The combined organic layers were dried over Na_2SO_4, filtered, concentrated and purified by flash column chromatography on silica gel (petroleum ether: ethyl acetate = 10: 1) to afford the crude product **Xu8-6** as a yellow solid. Mp: 111.2-114.1 °C. $[\alpha]_D^{20}$ = -90.7 (*c* = 0.4, Chloroform). ^1H NMR (400 MHz, Chloroform-*d*) δ 9.65 (s, 1H), 7.87 (dd, *J* = 8.7, 2.8 Hz, 1H), 7.57 – 7.48 (m, 2H), 7.47 – 7.39 (m,

2H), 7.38 – 7.30 (m, 1H), 7.04 (d, J = 8.6 Hz, 1H), 5.17 (s, 2H), 3.90 (s, 3H), 2.33 (dddp, J = 14.9, 11.3, 7.7, 3.5, 3.0 Hz, 2H), 1.79 (d, J = 11.9 Hz, 2H), 1.69 (d, J = 7.6 Hz, 2H), 1.57 (d, J = 7.5 Hz, 4H), 1.38 – 1.31 (m, 2H), 1.26 (s, 9H), 1.25 – 1.06 (m, 8H), 1.03 – 0.90 (m, 2H). ^{13}C NMR (100 MHz, Chloroform-*d*) δ 164.1, 163.7, 154.5, 151.0, 138.0, 128.3, 127.6, 127.4, 124.3, 124.3, 113.5, 74.0, 57.5, 55.6, 34.8, 32.3 (d, J = 24.1 Hz), 30.7 (dd, J = 9.7, 7.1 Hz), 26.8 (d, J = 8.9 Hz), 26.6 (dd, J = 13.9, 4.4 Hz), 26.2, 22.6. ^{31}P NMR (162 MHz, Chloroform-*d*) δ -9.32. HRMS (ESI) calculated for [$C_{31}H_{45}NO_3PS$] [M+H]$^+$: 542.2852 found: 542.2865.

2.3.6 (*R*)-N-((*S*)-[1,1'-biphenyl]-4-yl(3-(benzyloxy)-2-(dicyclohexylphosphanyl)-4-methoxy-phenyl)-methyl)-2-methylpropane-2-sulfinamide (Xu8-7)

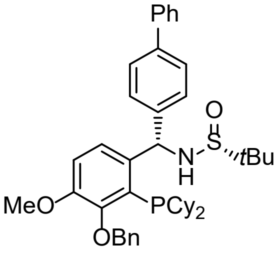

Xu8-7

Prepared from (*R*, *E*)-N-(3-(benzyloxy)-2-(dicyclohexylphosphanyl)-4-methoxybenzylidene)-2-methylpropane-2-sulfinamide the crude product **Xu8-6** in 2 mL anhydrous THF, was added [1,1'-biphenyl]-4-ylmagnesium bromide (4 mmol, 2.0 equiv.) dropwise under argon at -50 °C. The reaction mixture was warmed to room temperature overnight and was quenched by the addition of NH_4Cl (aq.) and diluted with EtOAc. The organic layer was separated, and the aqueous layer was extracted twice with EtOAc. The combined organic layers were dried over Na_2SO_4, filtered, concentrated and purified by flash column chromatography on silica gel (petroleum ether: ethyl acetate = 5: 1) to the product **Xu8-7** as a white solid (626 mg, 18% yield). Mp: 87.8-89.0 °C. $[\alpha]_D^{20}$ = -109.2 (*c* = 0.4, Chloroform). ^{1}H NMR (500 MHz, Chloroform-*d*) δ 7.60 – 7.52 (m, 2 H), 7.52 – 7.45 (m, 6 H), 7.45 – 7.36 (m, 5 H), 7.35 – 7.28 (m, 2 H), 7.06 (d, J = 8.6 Hz, 1 H), 6.93 (s, 1 H), 5.28 (d, J = 11.7 Hz, 1 H), 5.17 (d, J = 11.7 Hz, 1 H), 4.00 (s, 1 H), 3.86 (s, 3 H), 2.34 – 2.16 (m, 2 H), 1.79 – 1.53 (m, 6 H), 1.51 – 1.41 (m, 1 H), 1.38 – 0.87 (m, 22 H). ^{13}C NMR (125 MHz, Chloroform-*d*) δ 150.8 (d, J = 3.7 H z), 150.5, 142.4, 141.9 (d, J = 23.9 Hz), 140.9, 140.0, 138.5, 129.18, 129.17, 128.7 (d, J = 29.4 Hz), 128.6, 128.1, 127.3, 127.1, 127.03, 127.00, 126.9, 123.2 (d,

J = 6.5 Hz), 113.8, 73.3, 60.0 (d, J = 35.5 Hz), 55.8, 55.5, 35.2 (d, J = 10.7 Hz), 34.3 (d, J = 11.3 Hz), 33.1 (d, J = 25.9 Hz), 32.3 (d, J = 22.8 Hz), 30.4 (dd, J = 21.4, 9.2 Hz), 27.0 (dd, J = 8.6, 6.2 Hz), 26.8 (dd, J = 14.2, 8.0 Hz), 26.2, 22.7. ^{31}P NMR (202 MHz, Chloroform-*d*) δ -6.74. HRMS (ESI) calculated for [$C_{43}H_{54}NNaO_3PS$] $[M+Na]^+$: 718.3454 found: 718.3457.

2.3.7 (*R*)-N-((*S*)-(3-(benzyloxy)-2-(dicyclohexylphosphaneyl)-4-methoxyphenyl)(3,5-bis(trifle-oromethyl)phenyl)methyl)-2-methylpropane-2-sulfinamide (Xu9-7)

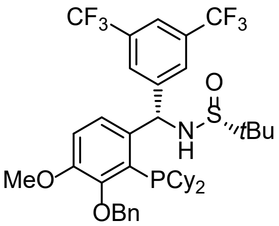

Xu9-7

Prepared from (*R*, *E*)-N-(3-(benzyloxy)-2-(dicyclohexylphosphanyl)-4-methoxybenzylidene)-2-methylpropane-2-sulfinamide the crude product **Xu8-6** in 2 mL anhydrous THF, was added (3,5-bis(trifluoromethyl)phenyl)magnesium bromide (10 mmol, 5.0 equiv.) dropwise under argon at -50 °C. The reaction mixture was warmed to room temperature overnight and was quenched by the addition of NH_4Cl (aq.) and diluted with EtOAc. The organic layer was separated, and the aqueous layer was extracted twice with EtOAc. The combined organic layers were dried over Na_2SO_4, filtered, concentrated and purified by flash column chromatography on silica gel (petroleum ether: ethyl acetate = 5: 1) to the product **Xu9-7** as a white solid (800 mg, 53% yield). Mp: 68.1-71.3 °C. $[\alpha]_D^{20}$ = -67.8 (*c* = 0.4, Chloroform). ^{1}H NMR (500 MHz, Chloroform-*d*) δ 7.93 (s, 2H), 7.71 (s, 1H), 7.45 (d, J = 8.1 Hz, 2H), 7.42 – 7.36 (m, 2H), 7.34 – 7.27 (m, 2H), 7.06 (dd, J = 8.6, 1.8 Hz, 1H), 6.86 (s, 1H), 5.28 (dd, J = 11.7, 1.7 Hz, 1H), 5.15 (d, J = 11.6 Hz, 1H), 3.87 (d, J = 1.8 Hz, 3H), 2.23 (d, J = 14.7 Hz, 2H), 1.80 – 1.47 (m, 10H), 1.40 – 1.33 (m, 1H), 1.27 (d, J = 1.9 Hz, 9H), 1.16 – 0.91 (m, 9H). ^{13}C NMR (125 MHz, Chloroform-*d*) δ 151.2, 151.0, 146.4,140.5 (d, J = 24.7 Hz), 138.3, 131.3 (q, J = 33.0 Hz), 128.9, 128.2, 127.5, 127.1, 124.4, 123.1, 122.2, 120.9, 114.2, 73.5, 56.2, 55.5, 35.3 (d, J = 10.0 Hz), 34.3 (d, J = 10.4 Hz), 33.1 (d, J = 25.9 Hz), 32.3 (d, J = 22.5 Hz), 30.6 (d, J = 9.3 Hz), 30.5 (d, J = 10.5 Hz), 27.0, 26.9, 26.9, 26.8 (d, J = 3.6 Hz), 26.7 (d, J = 4.5 Hz), 26.1 (d, J = 5.1 Hz) , 22.7. ^{31}P NMR (202 MHz, Chloroform-*d*) δ -5.95.

^{19}F NMR (376 MHz, Chloroform-*d*) δ -62.72. HRMS (ESI) calculated for [$C_{39}H_{49}F_6NO_3PS$] $[M+H]^+$: 756.3069 found: 756.3083.

2.3.8 (*R*)-N-((*R*)-(3-(benzyloxy)-2-(dicyclohexylphosphaneyl)-4-methoxyphenyl)(3,5bis(trifle-oromethyl)phenyl)methyl)-2-methylpropane-2-sulfinamide

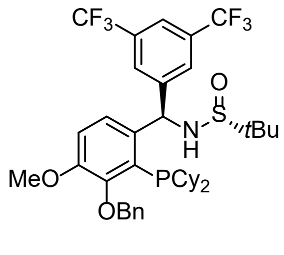

(*R*, *R*s)-Xu9-7

To a solution of 1-bromo-3,5-bis(trifluoromethyl)benzene (4 mmol) in dry THF (4 mL) was added *n*-BuLi (4 mmol, 2.4 M in hexane) dropwise under argon at -78 °C. The resulting solution at this temperature during 1 hour and the **Xu8-6** (2 mmol) in 2 mL anhydrous THF was added dropwise. Then the reaction mixture was warmed to room temperature overnight and was quenched by the addition of NH_4Cl (aq.) and diluted with EtOAc. The organic layer was separated, and the aqueous layer was extracted twice with EtOAc. The combined organic layers were dried over Na_2SO_4, filtered, concentrated and purified by flash column chromatography on silica gel (petroleum ether: ethyl acetate = 5: 1) to the product **(*R*, *R*s)-Xu9-7** as a white solid (720 mg, 48% yield). Mp: 79.2-81.1 °C. $[\alpha]_D^{20}$ = -3.5 (*c* = 0.4, Chloroform). ^1H NMR (400 MHz, Chloroform-*d*) δ 7.98 – 7.89 (m, 2H), 7.70 (s, 1H), 7.47 – 7.42 (m, 2H), 7.40 (t, *J* = 7.5 Hz, 2H), 7.37 – 7.29 (m, 1H), 7.27 – 7.23 (m, 1H), 7.04 (d, *J* = 8.5 Hz, 1H), 6.87 (dd, *J* = 9.1, 4.5 Hz, 1H), 5.25 (d, *J* = 11.6 Hz, 1H), 5.16 (d, *J* = 11.6 Hz, 1H), 4.24 (s, 1H), 3.84 (s, 3H), 2.32 – 2.16 (m, 2H), 1.75 – 1.49 (m, 8H), 1.42 (q, *J* = 3.5, 2.5 Hz, 1H), 1.27 (d, *J* = 8.3 Hz, 11H), 1.06 (ddd, *J* = 31.2, 15.6, 7.6 Hz, 9H). ^{13}C NMR (125 MHz, Chloroform-*d*) δ 151.3 (d, *J* = 3.5 Hz), 151.2, 146.1, 141.5 (d, *J* = 24.3 Hz), 138.3,131.2 (q, *J* = 33.2 Hz), 128.9, 128.3, 127.5, 127.1, 124.5,122.6 (d, *J* = 7.2 Hz), 122.3, 120.8, 114.7, 73.6, 59.7 (d, *J* = 31.4 Hz), 56.3, 55.6, 35.3 (d, *J* = 10.2 Hz), 34.7 (d, *J* = 10.0 Hz), 33.1 (d, *J* = 25.4 Hz), 32.4 (d, *J* = 22.5 Hz), 30.7 (d, *J* = 9.7 Hz), 30.6 (d, *J* = 7.2 Hz), 27.1, 27.0, 26.9 (d, *J* = 4.6 Hz), 26.8 (d, *J* = 3.4 Hz), 26.6, 26.1, 22.8. ^{31}P NMR (202 MHz, Chloroform-*d*) δ -6.70. ^{19}F NMR (376

MHz, Chloroform-*d*) δ -62.79. HRMS (ESI) calculated for [$C_{39}H_{49}F_6NO_3PS$] $[M+H]^+$: 756.3069 found: 756.3072.

2.3.9 (*R*)-N-((*S*)-[1,1'-biphenyl]-4-yl(3-(benzyloxy)-2-(dicyclohexylphosphanyl)-4-methoxyphenyl)-methyl)-N,2-dimethylpropane-2-sulfinamide (Xu8)

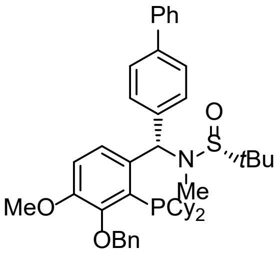

Xu8

Prepared from (*R*)-N-((*S*)-[1,1'-biphenyl]-4-yl(3-(benzyloxy)-2-(dicyclohexylphosphanyl)-4-methoxyphenyl)methyl)-2-methylpropane-2-sulfinamide **Xu8-7** (0.5 mmol) in 2 mL anhydrous THF, was added *n*-BuLi (0.75 mmol, 1.6 M in hexane) dropwise under argon at -40 °C. The resulting solution at this temperature during 1 hour and iodomethane (1 mmol, 2 equiv.) was added. After 1 hour, the reaction mixture moved to 0 °C and stirred 1 hour. The reaction mixture was the addition of NH_4Cl (aq.) and diluted with EtOAc. The organic layer was separated, and the aqueous layer was extracted twice with EtOAc. The combined organic layers were dried over Na_2SO_4, filtered, concentrated and purified by flash column chromatography on silica gel (petroleum ether: ethyl acetate = 5: 1) to the product **Xu8** as a white solid (245 mg, 69% yield). Mp: 86.0-87.2 °C. $[\alpha]_D^{20}$ = 11.3 (*c* = 0.4, Chloroform). ^{1}H NMR (500 MHz, Chloroform-*d*) δ 7.59 – 7.52 (m, 3 H), 7.50 – 7.44 (m, 4 H), 7.45 – 7.35 (m, 4 H), 7.35 – 7.28 (m, 2 H), 7.28 – 7.21 (m, 2 H), 7.09 (d, *J* = 8.6 Hz, 1 H), 7.03 (d, *J* = 11.4 Hz, 1 H), 5.21 (s, 2 H), 3.87 (s, 3 H), 2.64 (s, 3 H), 2.45 – 2.30 (m, 1 H), 2.13 – 1.97 (m, 1 H), 1.75 – 1.54 (m, 5 H), 1.54 – 1.44 (m, 1 H), 1.41 – 1.35 (m, 2 H), 1.24 – 1.14 (m, 4 H), 1.14 – 1.02 (m, 11 H), 0.99 – 0.69 (m, 6 H). ^{13}C NMR (125 MHz, Chloroform-*d*) δ 150.9 (d, *J* = 3.8 Hz), 150.4, 140.9, 140.4 (d, *J* = 22.9 Hz), 139.8, 139.4, 138.6, 131.7, 128.6, 128.6 (d, *J* = 30.9 Hz), 128.1, 127.3, 127.1, 127.02, 127.01, 126.3, 122.9 (d, *J* = 5.7 Hz), 113.9, 73.3, 70.5 (d, *J* = 39.9 Hz), 58.6, 55.5, 35.2 (d, *J* = 10.4 Hz), 34.0 (d, *J* = 11.6 Hz), 33.2 (d, *J* = 26.1 Hz), 32.2 (d, *J* = 22.3 Hz), 30.9 (d, *J* = 11.5 Hz), 30.5 , 29.9 (d, *J* = 8.0 Hz), 27.0 (t, *J* = 9.4 Hz), 26.8 (d, *J* = 2.9

Hz), 26.7 (d, J = 4.8 Hz), 26.3, 26.0, 24.1. ^{31}P NMR (202 MHz, Chloroform-*d*) δ -6.11. HRMS (ESI) calculated for [$C_{44}H_{56}NNaO_3PS$] $[M+Na]^+$: 732.3611 found: 732.3614.

2.3.10 (*R*)-N-((*S*)-(3-(benzyloxy)-2-(dicyclohexylphosphaneyl)-4-methoxyphenyl)(3,5-bis(tri-fleoromethyl)phenyl)methyl)-N,2-dimethylpropane-2-sulfinamide (Xu9)

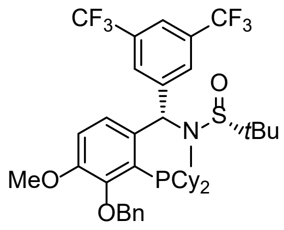

Xu9

Prepared from (*R*)-N-((*S*)-(3-(benzyloxy)-2-(dicyclohexylphosphaneyl)-4-methoxyphenyl)(3,5-bis-(trifle-oromethyl)phenyl)methyl)-2-methylpropane-2-sulfinamide **Xu9-7** (0.5 mmol) in 2 mL anhydrous THF, was added *n*-BuLi (0.75 mmol, 1.6 M in hexane) dropwise under argon at -40 °C. The resulting solution at this temperature during 1 hour and iodomethane (1 mmol, 2 equiv.) was added. After 1 hour, the reaction mixture moved to 0 °C and stirred 1 hour. The reaction mixture was the addition of NH_4Cl (aq.) and diluted with EtOAc. The organic layer was separated, and the aqueous layer was extracted twice with EtOAc. The combined organic layers were dried over Na_2SO_4, filtered, concentrated and purified by flash column chromatography on silica gel (petroleum ether: ethyl acetate = 5: 1) to the product **Xu9** as a white solid (230 mg, 60% yield). Mp: 146-148.5 °C. $[\alpha]_D^{20}$ = 45.9 (c = 0.4, Chloroform). ^{1}H NMR (500 MHz, Chloroform-*d*) δ 7.72 (s, 1H), 7.65 (s, 2H), 7.57 (dd, J = 8.6, 4.1 Hz, 1H), 7.45 (d, J = 7.6 Hz, 2H), 7.39 (t, J = 7.5 Hz, 2H), 7.31 (t, J = 7.4 Hz, 1H), 7.11 (d, J = 10.1 Hz, 2H), 5.22 (s, 2H), 3.88 (s, 3H), 2.65 (s, 3H), 2.32 (q, J = 10.8 Hz, 1H), 2.08 (q, J = 11.3 Hz, 1H), 1.68 (d, J = 9.1 Hz, 3H), 1.61 (d, J = 9.8 Hz, 3H), 1.52 – 1.39 (m, 3H), 1.36 (d, J = 12.5 Hz, 1H), 1.25 – 1.14 (m, 4H), 1.07 (s, 10H), 0.96 – 0.73 (m, 5H). ^{13}C NMR (125 MHz, Chloroform-*d*) δ 151.3 (d, J = 3.8 Hz), 151.1, 143.6, 138.4, 138.0 (d, J = 22.8 Hz), 131.6, 131.0 (q, J = 33.1 Hz), 128.5 (d, J = 30.7 Hz), 128.3, 127.5, 127.1, 124.4, 122.8 (d, J = 5.5 Hz), 122.2, 120.9, 114.3, 73.6, 70.2 (d, J = 41.2 Hz), 59.2, 55.5, 35.3 (d, J = 10.0 Hz), 33.8 (d, J = 10.8 Hz), 33.2 (d, J = 26.2 Hz), 32.2 (d, J = 21.8 Hz), 31.0, 30.9, 30.2 (d, J = 7.5 Hz), 27.0 (d, J = 9.5 Hz), 26.8 – 26.6 (m), 26.6, 26.3, 26.0, 24.1. ^{31}P NMR (202 MHz, Chloroform-*d*) δ -5.88.

^{19}F NMR (376 MHz, Chloroform-*d*) δ -62.83. HRMS (ESI) calculated for [$C_{40}H_{51}F_6NO_3PS$] $[M+H]^+$: 770.3226 found: 770.3246.

2.3.11 (*R*)-N-((*R*)-(3-(benzyloxy)-2-(dicyclohexylphosphaneyl)-4-methoxyphenyl)(3,5-bis(trifluoromethyl)phenyl)methyl)-N,2-dimethylpropane-2-sulfinamide ((*R*, *R_S*)-Xu9)

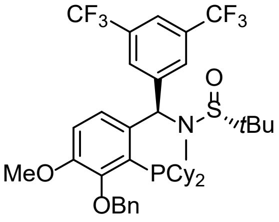

(*R*, *R_S*)-Xu9

Prepared from (*R*)-N-((*R*)-(3-(benzyloxy)-2-(dicyclohexylphosphaneyl)-4-methoxyphenyl)(3,5-bis-(trifle-oromethyl)phenyl)methyl)-2-methylpropane-2-sulfinamide **(*R*, *R_S*)-Xu9-7** (0.5 mmol) in 2 mL anhydrous THF, was added *n*-BuLi (0.75 mmol, 1.6 M in hexane) dropwise under argon at -40 °C. The resulting solution at this temperature during 1 hour and iodomethane (1 mmol, 2 equiv.) was added. After 1 hour, the reaction mixture moved to 0 °C and stirred 1 hour. The reaction mixture was the addition of NH_4Cl (aq.) and diluted with EtOAc. The organic layer was separated, and the aqueous layer was extracted twice with EtOAc. The combined organic layers were dried over Na_2SO_4, filtered, concentrated and purified by flash column chromatography on silica gel (petroleum ether: ethyl acetate = 5: 1) to the product **(*R*, *R_S*)-Xu9** as a white solid (238 mg, 62% yield). Mp: 67.5-70.1 °C. $[\alpha]_D^{20}$ = -23.4 (*c* = 0.4, Chloroform). ^1H NMR (400 MHz, Chloroform-*d*) δ 7.80 (s, 2H), 7.70 (s, 1H), 7.48 – 7.27 (m, 6H), 7.10 (d, *J* = 8.6 Hz, 1H), 6.93 (d, *J* = 9.9 Hz, 1H), 5.29 – 5.09 (m, 2H), 3.87 (s, 3H), 2.60 (s, 3H), 2.31 (dtt, *J* = 11.7, 8.5, 3.4 Hz, 1H), 2.11 – 1.95 (m, 1H), 1.71 – 1.57 (m, 5H), 1.52 (ddd, *J* = 12.1, 4.8, 2.4 Hz, 2H), 1.39 – 1.30 (m, 2H), 1.30 – 1.21 (m, 3H), 1.18 (s, 9H), 1.11 – 1.02 (m, 2H), 0.93 – 0.83 (m, 4H), 0.82 – 0.72 (m, 2H). ^{13}C NMR (100 MHz, Chloroform-*d*) δ 151.3 (d, *J* = 3.6 Hz), 150.9, 143.9, 138.4, 140.1 (d, *J* = 23.1 Hz), 131.3,131.7 – 130.6 (m), 128.7 (d, *J* = 32.3 Hz), 128.2, 127.5, 127.0, 124.7,121.9 (d, *J* = 6.2 Hz), 121.1 – 120.5 (m). 114.2,73.5, 58.9, 55.5, 35.6 (d, *J* = 10.6 Hz), 34.2 (d, *J* = 11.5 Hz), 33.1 (d, *J* = 26.7 Hz), 32.2 (d, *J* = 22.3 Hz), 31.0 (d, *J* = 11.0 Hz), 29.9 (d, *J* = 7.1 Hz), 26.9 (ddd, *J* = 21.0, 17.3,

8.4 Hz). ^{31}P NMR (162 MHz, Chloroform-*d*) δ -6.86. ^{19}F NMR (376 MHz, Chloroform-*d*) δ -62.76. HRMS (ESI) calculated for $[C_{40}H_{51}F_6NO_3PS]$ $[M+H]^+$: 770.3226 found: 770.3241.

2.4 Synthesis of Xu10

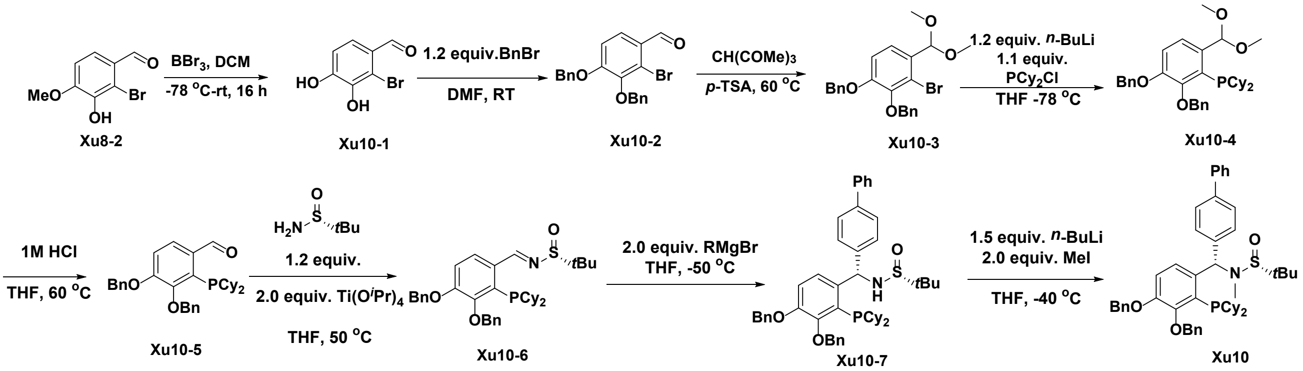

2.4.1 2-bromo-3,4-dihydroxybenzaldehyde (Xu10-1)

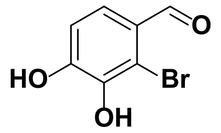

Xu10-1

To a solution of 2-bromo-3-hydroxy-4-methoxybenzaldehyde **Xu8-2** (3.45g, 15 mmol) in anhydrous CH_2Cl_2 (60 mL) at -78 °C was added BBr_3 (1M in CH_2Cl_2, 60 mL, 60 mmol). The mixture was warmed up to 25 °C and stirred for 16 h. Then the solution was cooled down to -78 °C and MeOH (35 mL) was added. The mixture was poured into water (100 mL) and extracted with EtOAc (3 x 100 mL). Combined organic fractions were washed with brine (100 mL), dried over MgSO4, filtered, and the solvent was evaporated. The residue was added to next step.

2.4.2 3,4-bis(benzyloxy)-2-bromobenzaldehyde (Xu10-2)

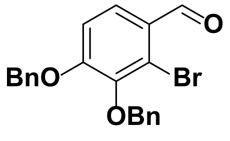

Xu10-2

Prepared from 2-bromo-3,4-dihydroxybenzaldehyde **Xu10-1** (10 mmol) in DMF (20 mL) was added benzyl bromide (30 mmol, 3 equiv.) and K_2CO_3 (30 mmol, 3 equiv.). The resulting solution was stirred at room temperature overnight. The reaction mixture was quenched by the addition of

H_2O and diluted with EtOAc. The organic layer was separated, and the aqueous layer was extracted twice with EtOAc. The combined organic layers were dried over Na_2SO_4, filtered, concentrated. The crude product was then washed by petroleum ether with a little ethyl acetate to afford the product **Xu10-2** as a white solid (1.5 g, 38% yield). Mp: 136.5-137.4 °C.^{1}H NMR (400 MHz, Chloroform-*d*) δ 10.24 (s, 1H), 7.70 (d, J = 8.6 Hz, 1H), 7.54 – 7.28 (m, 10H), 7.01 (d, J = 8.6 Hz, 1H), 5.18 (s, 2H), 5.04 (d, J = 1.7 Hz, 2H). ^{13}C NMR (100 MHz, Chloroform-*d*) δ 190.8, 157.7, 145.4, 136. 5, 135.4, 128.7, 128.6, 128.4, 128.3, 128.2, 127.5, 127.4, 126.4, 123.6, 112.3, 74.8, 71.1. HRMS (ESI) calculated for [$C_{21}H_{17}BrNaO_3$] [M+Na]$^+$: 419.0253 found: 419.0242.

2.4.3 (((3-bromo-4-(dimethoxymethyl)-1,2-phenylene)bis(oxy))bis(methylene))dibenzene (Xu10-3)

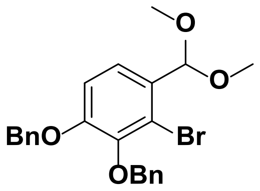

Xu10-3

Prepared from 3,4-bis(benzyloxy)-2-bromobenzaldehyde **(Xu10-2)** (3 mmol) in trimethoxymethane (30 mmol, 10 equiv.) and *p*-toluenesulfonic acid (0.15 mmol, 5% mol). The resulting solution was stirred 5 hours at 60 °C. The reaction mixture was washed by the addition of $NaHCO_3$ (aq.) and the combined organic layers concentrated. The crude product was purified by flash column chromatography on aluminum oxide (petroleum ether: ethyl acetate = 20: 1) to afford the product **Xu10-3** as a white liquid (1.1 g, 83% yield). ^{1}H NMR (400 MHz, Chloroform-*d*) δ 7.41 (dd, J = 7.3, 2.2 Hz, 2H), 7.38 – 7.32 (m, 2H), 7.31 – 7.21 (m, 7H), 6.88 (d, J = 8.6 Hz, 1H), 5.46 (s, 1H), 5.05 (s, 2H), 4.96 (s, 2H), 3.29 (s, 6H). ^{13}C NMR (100 MHz, Chloroform-*d*) δ 153.0, 145.7, 137.1, 136.5, 130.5, 129.9, 128.6, 128.6, 128.3, 128.2, 128.1, 127.5, 123.5, 119.4, 112.8, 103.1, 74.7, 71.2, 54.0. HRMS (ESI) calculated for [$C_{23}H_{23}BrNaO_4$] [M+Na]$^+$: 465.0672 found: 465.0682.

2.4.4 (2,3-bis(benzyloxy)-6-(dimethoxymethyl)phenyl)dicyclohexylphosphane (Xu10-4)

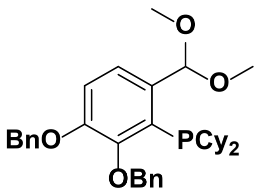

Xu10-4

Prepared from (((3-bromo-4-(dimethoxymethyl)-1,2-phenylene)bis(oxy))bis(methylene))dibenzene (**Xu10-3**) (2 mmol) in 4 mL anhydrous THF, was added *n*-BuLi (2.4 mmol, 1.6 M in hexane) dropwise under argon at -78 °C, The resulting solution at this temperature during 1 hour, and dicyclohexylchlorophosphine (2.6 mmol, 1.3 equiv.) was added dropwise. The reaction mixture was warmed to room temperature overnight. The re action mixture was quenched by the addition of $NaHCO_3$ (aq.) and diluted with EtOAc. The organic layer was separated, and the aqueous layer was extracted twice with EtOAc. The combined organic layers were concentrated. The crude product was purified by flash column chromatography on aluminum oxide (petroleum ether: ethyl acetate = 20: 1) to afford the crude product **Xu10-4** as a yellow liquid.^{1}H NMR (400 MHz, Chloroform-*d*) δ 7.43 – 7.27 (m, 12H), 7.09 (d, *J* = 8.6 Hz, 1H), 6.28 (d, *J* = 7.6 Hz, 1H), 5.25 (s, 2H), 5.09 (s, 2H), 3.36 (s, 6H), 2.35 (dtt, *J* = 11.7, 7.9, 3.7 Hz, 2H), 1.78 (d, *J* = 12.5 Hz, 2H), 1.68 (d, *J* = 11.1 Hz, 2H), 1.62 – 1.57 (m, 4H), 1.35 (d, *J* = 15.3 Hz, 3H), 1.29 (s, 1H), 1.25 (s, 1H), 1.13 (d, *J* = 10.1 Hz, 5H), 1.03 – 0.91 (m, 2H). ^{13}C NMR (100 MHz, Chloroform-*d*) δ 150.6, 138.5, 136.6, 128.5, 128.2, 128.0, 127.6, 127.4, 127.2, 121.4 (d, *J* = 7.1 Hz), 115.8, 102.5, 102.1, 73.5, 71.2, 53.9, 34.6 (d, *J* = 10.8 Hz), 32.8 (d, *J* = 23.7 Hz), 30.9 (d, *J* = 10.3 Hz), 27.0 (d, *J* = 8.9 Hz), 26.8 (d, *J* = 13.8 Hz), 26.3. ^{31}P NMR (162 MHz, Chloroform-*d*) δ -6.73. HRMS (ESI) calculated for [$C_{35}H_{46}O_4P$] $[M+H]^+$: 561.3128 found: 561.3128.

2.4.5 3,4-bis(benzyloxy)-2-(dicyclohexylphosphaneyl)benzaldehyde (Xu10-5)

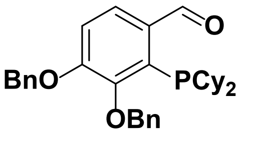

Xu10-5

Prepared from (2,3-bis(benzyloxy)-6-(dimethoxymethyl)phenyl)dicyclohexylphosphane **(Xu10-4)** in 2 mL THF, was added 1 mL HCl (1.0 M) under argon at 60 °C. The resulting solution was stirred 5 hours. The reaction mixture was quenched by the addition of $NaHCO_3$ (aq.) and diluted

with EtOAc. The organic layer was separated, and the aqueous layer was extracted twice with EtOAc. The combined organic layers were dried over Na_2SO_4, filtered, concentrated and purified by flash column chromatography on silica gel (petroleum ether: ethyl acetate = 20: 1) to afford the product **Xu10-5** as a yellow liquid. ^{1}H NMR (400 MHz, Chloroform-*d*) δ 11.06 (d, *J* = 8.7 Hz, 1H), 7.76 (dd, *J* = 8.6, 3.1 Hz, 1H), 7.46 – 7.28 (m, 10H), 7.15 (d, *J* = 8.6 Hz, 1H), 5.22 (s, 2H), 5.17 (s, 2H), 2.36 (tdt, *J* = 11.4, 7.4, 3.4 Hz, 2H), 1.85 – 1.78 (m, 2H), 1.71 (t, *J* = 5.2 Hz, 2H), 1.59 (d, *J* = 8.1 Hz, 4H), 1.34 (d, *J* = 3.4 Hz, 2H), 1.26 (s, 2H), 1.16 (d, *J* = 8.2 Hz, 6H), 1.04 – 0.92 (m, 2H). ^{13}C NMR (100 MHz, Chloroform-*d*) δ 193.41 (d, J = 41.3 Hz), 155.1, 151.0, 137.9, 135.8, 128.7, 128.4, 128.3, 127.7, 127.5, 124.3 (d, *J* = 6.0 Hz), 115.0, 73.9, 71.0, 34.6 (d, *J* = 11.0 Hz), 32.6, 32.4, 31.5, 31.0, 30.9, 30.2, 29.7, 26.9, 26.8, 26.8, 26.6, 26.2. ^{31}P NMR (162 MHz, Chloroform-*d*) δ -13.80. HRMS (ESI) calculated for $[C_{33}H_{40}O_3P]$ $[M+H]^+$: 515.2710 found: 515.2724.

2.4.6 (*R, E*)-N-(3,4-bis(benzyloxy)-2-(dicyclohexylphosphaneyl)benzylidene)-2-methylpropan-e-2-sulfinamide (Xu10-6)

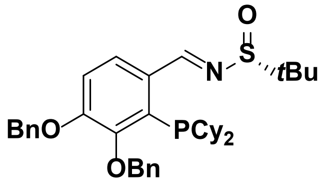

Xu10-6

Prepared from 3,4-bis(benzyloxy)-2-(dicyclohexylphosphaneyl)benzaldehyde **(Xu10-5)** in 2 mL THF, was added (*R*)-2-methylpropane-2-sulfinamide (2.4 mmol, 1.2 equiv.) and titanium tetraisopropanolate (4 mmol, 2.0 equiv.) under argon at 50 °C. The resulting solution was stirred 8 hours. The reaction mixture was quenched by the addition of H_2O (aq.) and diluted with EtOAc. The solution was filtered and the residue was washed twice with EtOAc. The combined organic layers were dried over Na_2SO_4, filtered, concentrated and purified by flash column chromatography on silica gel (petroleum ether: ethyl acetate = 10: 1) to afford the crude product **Xu10-6** as a yellow solid. Mp: 63.7-65.2 °C. $[\alpha]_D^{20}$ = -66.1 (*c* = 0.4, Chloroform). ^{1}H NMR (500 MHz, Chloroform-*d*) δ 9.66 (s, 1H), 7.86 (dd, *J* = 8.9, 2.6 Hz, 1H), 7.53 – 7.30 (m, 10H), 7.14 (dd, *J* = 8.7, 1.9 Hz, 1H), 5.31 – 5.09 (m, 4H), 2.42 – 2.24 (m, 2H), 1.83 (d, *J* = 12.8 Hz, 2H), 1.72 (s, 2H), 1.60 (d, *J* = 6.8 Hz, 4H), 1.38 (dd, *J* = 16.8, 7.0 Hz, 2H), 1.29 (d, *J* = 2.0 Hz, 11H), 1.18 (d, *J* = 7.9 Hz, 6H), 1.07 – 0.96 (m, 2H). ^{13}C NMR (125 MHz, Chloroform-*d*) δ 163.94 (d, *J* = 34.5 Hz), 153.76, 151.56, 138.00, 136.11, 134.97, 133.35 (d, *J* = 36.4 Hz), 128.67, 128.35, 128.28, 128.25, 127.68, 127.65,

124.30, 115.32, 74.08, 71.02, 57.64, 34.97 (d, J = 12.1 Hz), 32.45 (d, J = 23.4 Hz), 31.55, 30.77 (t, J = 10.2 Hz), 30.19, 29.73, 26.94 (d, J = 8.6 Hz), 26.79 (d, J = 6.4 Hz), 26.68 (d, J = 5.8 Hz), 26.28, 22.72. ^{31}P NMR (203 MHz, $CDCl_3$) δ -9.13. HRMS (ESI) calculated for [$C_{37}H_{49}NO_3PS$] $[M+H]^+$: 618.3165 found: 618.3179.

2.4.7 (*R*)-N-((*S*)-[1,1'-biphenyl]-4-yl(3,4-bis(benzyloxy)-2-(dicyclohexylphosphaneyl)phenyl) methyl)-2-methylpropane-2-sulfinamide (Xu10-7)

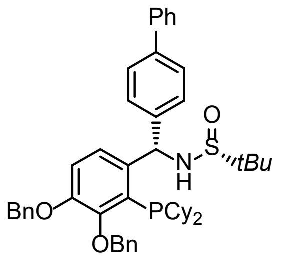

Xu10-7

Prepared from (R, E)-N-(3,4-bis(benzyloxy)-2-(dicyclohexylphosphaneyl)benzylidene)-2-Methyl-propane-2-sulfinamide **(Xu10-6)** in 1 mL anhydrous THF, was added [1,1'-biphenyl]-4-ylmagnesium bromide (1 mmol, 2.0 equiv.) dropwise under argon at -50 °C. The reaction mixture was warmed to room temperature overnight and was quenched by the addition of NH_4Cl (aq.) and diluted with EtOAc. The organic layer was separated, and the aqueous layer was extracted twice with EtOAc. The combined organic layers were dried over Na_2SO_4, filtered, concentrated and purified by flash column chromatography on silica gel (petroleum ether: ethyl acetate = 5: 1) to the product **Xu10-7** as a white solid (262 mg, 68% yield). Mp: 85-86.5 °C. $[\alpha]_D^{20}$ = -64.6 (c = 0.4, Chloroform).^{1}H NMR (400 MHz, Chloroform-d) δ 7.56 – 7.44 (m, 6H), 7.44 – 7.26 (m, 14H), 7.13 (d, J = 8.6 Hz, 1H), 6.93 (d, J = 9.4 Hz, 1H), 5.31 (d, J = 11.6 Hz, 1H), 5.20 (d, J = 11.6 Hz, 1H), 5.09 (s, 2H), 3.98 (s, 1H), 2.36 – 2.12 (m, 2H), 1.75 – 1.51 (m, 6H), 1.50 – 1.41 (m, 1H), 1.26 (s, 22H). ^{13}C NMR (100 MHz, Chloroform-d) δ 151.4, 149.7, 142.4, 141.0, 140.1, 138.4, 136.6, 129.2 (d, J = 2.2 Hz), 128.7, 128.5, 128.1, 128.0, 127.7, 127.3, 127.1, 127.1, 127.0, 123.2 (d, J = 4.2 Hz), 115.9, 73.4, 71.1, 55.9, 35.3 (d, J = 10.8 Hz), 34.4 (d, J = 11.6 Hz), 33.2 (d, J = 25.9 Hz), 32.3 (d, J = 22.7 Hz), 30.4 (dd, J = 18.4, 8.9 Hz), 27.7 – 26.4 (m), 26.2, 22.8.^{31}P NMR (162 MHz, Chloroform-d) δ -6.59. HRMS (ESI) calculated for [$C_{49}H_{59}NO_3PS$] $[M+H]^+$: 772.3948 found: 772.3967.

2.4.8 (*R*)-N-((*S*)-[1,1'-biphenyl]-4-yl(3,4-bis(benzyloxy)-2-(dicyclohexylphosphaneyl)phenyl)methyl)-N,2-dimethylpropane-2-sulfinamide (Xu10)

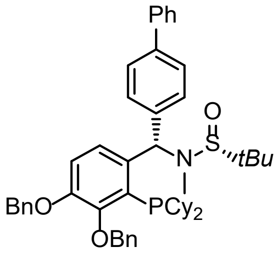

Xu10

Prepared from (R)-N-((S)-[1,1'-biphenyl]-4-yl(3,4-bis(benzyloxy)-2-(dicyclohexylphosphaneylphenyl)methyl)-2-methylpropane-2-sulfinamide (Xu10-7) in 1 mL anhydrous THF, was added *n*-BuLi (0.5 mmol, 1.6 M in hexane) dropwise under argon at -40 °C. The resulting solution at this temperature during 1 hour and iodomethane (0.6 mmol, 2 equiv.) was added. After 1 hour, the reaction mixture moved to 0 °C and stirred 1 hour. The reaction mixture was the addition of NH_4Cl (aq.) and diluted with EtOAc. The organic layer was separated, and the aqueous layer was extracted twice with EtOAc. The combined organic layers were dried over Na_2SO_4, filtered, concentrated and purified by flash column chromatography on silica gel (petroleum ether: ethyl acetate = 5: 1) to the product **Xu10** as a white solid (170 mg, 72% yield). Mp: 72-74.7 °C. $[\alpha]_D^{20}$ = 14.7 (*c* = 0.4, Chloroform).^{1}H NMR (400 MHz, Chloroform-*d*) δ 7.54 (ddd, *J* = 8.2, 5.3, 2.9 Hz, 3H), 7.46 (d, *J* = 8.2 Hz, 2H), 7.43 – 7.28 (m, 12H), 7.25 (dd, *J* = 8.4, 4.1 Hz, 2H), 7.16 (d, *J* = 8.6 Hz, 1H), 7.03 (d, *J* = 11.4 Hz, 1H), 5.25 (s, 2H), 5.10 (s, 2H), 2.64 (s, 3H), 2.35 (dqd, *J* = 11.3, 7.2, 4.8, 3.1 Hz, 1H), 2.06 (dtt, *J* = 11.7, 8.4, 3.4 Hz, 1H), 1.71 – 1.57 (m, 5H), 1.50 (dd, *J* = 10.8, 6.4 Hz, 1H), 1.41 – 1.34 (m, 2H), 1.21 (td, *J* = 15.5, 14.6, 5.7 Hz, 5H), 1.09 (s, 9H), 0.98 – 0.82 (m, 6H), 0.77 (tt, *J* = 12.3, 3.3 Hz, 1H). ^{13}C NMR (100 MHz, Chloroform-*d*) δ 151.6 (d, *J* = 3.7 Hz), 149.6, 141.2 (d, *J* = 23.0 Hz), 141.1, 139.9, 139.5, 138.5, 136.7, 131.8, 129.1, 128.8, 128.7, 128.5, 128.2, 128.0, 127.8, 127.4, 127.2, 127.1, 127.0, 126.5, 122.9 (d, *J* = 5.7 Hz), 116.2, 73.5, 71.3, 70.6 (d, *J* = 39.9 Hz), 58.7, 35.3 (d, *J* = 10.4 Hz), 34.7, 34.1(d, *J* = 11.7 Hz), 33.3(d, *J* = 26.3 Hz), 32.3 (d, *J* = 22.4 Hz), 31.0 (d, *J* = 11.6 Hz), 30.6, 30.0 (d, *J* = 8.0 Hz), 27.2 – 26.6 (m), 26.2, 25.3, 24.2. ^{31}P NMR (162 MHz, Chloroform-*d*) δ -5.94. HRMS (ESI) calculated for $[C_{50}H_{61}NO_3PS]$ $[M+H]^+$: 786.4104 found: 786.4118.

2.5 Synthesis of Xu12, Xu13

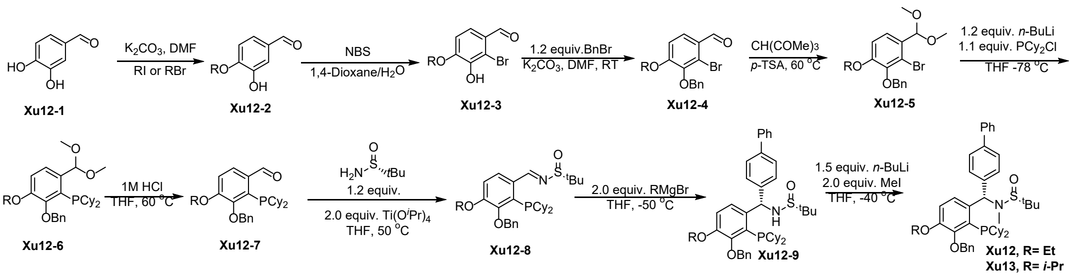

2.5.1 4-ethoxy-3-hydroxybenzaldehyde (Xu12-2)

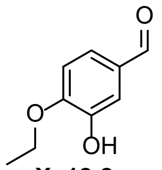

Xu12-2

Prepared from 3,4-dihydroxybenzaldehyde **Xu12-1** (30 mmol) in DMF (50 mL) was added iodoethane (33mmol, 1.1 equiv.) and K_2CO_3 (33 mmol, 1.1 equiv.). The resulting solution was stirred at 0 °C 48 h. The reaction mixture was quenched by the addition of H_2O and diluted with EtOAc. The organic layer was separated, and the aqueous layer was extracted twice with EtOAc. The combined organic layers were dried over Na_2SO_4, filtered, concentrated. the crude product was then purified by flash column chromatography on silica gel (petroleum ether: ethyl acetate = 5: 1) to afford the product **Xu12-2** as a white liquid (2.7 g, 55% yield). ^{1}H NMR (400 MHz, Chloroform-*d*) δ 9.84 (s, 1H), 7.44 (d, J = 1.9 Hz, 1H), 7.41 (dd, J = 8.2, 1.9 Hz, 1H), 6.95 (d, J = 8.2 Hz, 1H), 5.86 (s, 1H), 4.22 (q, J = 7.0 Hz, 2H), 1.50 (t, J = 7.0 Hz, 3H).^{13}C NMR (100 MHz, Chloroform-*d*) δ 191.0, 151.1, 146.2, 130.5, 124.5, 114.1, 110.8, 64.9, 14.6. HRMS (ESI) calculated for [$C_9H_{11}O_3$] [M+H]$^+$: 167.0704 found: 167.0703.

2.5.2 3-hydroxy-4-isopropoxybenzaldehyde (Xu13-2)

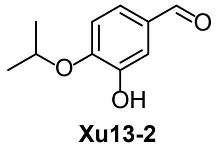

Xu13-2

Prepared from a stirred suspension of 3,4-dihydrobenzaldehyde **Xu12-1** (30 mmol), KI (3 mmol) and anhydrous potassium carbonate(30 mmol) in dry DMF (60 ml) was heated to 40°C and 2-bromopropane (39 mmol) added dropwise under nitrogen during 1.0 h. The mixture was stirred for a further 12 h and cooled to room temperature. The reaction mixture was quenched by the addition of H_2O and diluted with EtOAc. The organic layer was separated, and the aqueous layer was extracted twice with EtOAc. The combined organic layers were dried over Na_2SO_4, filtered,

concentrated. the crude product was then purified by flash column chromatography on silica gel (petroleum ether: ethyl acetate = 10: 1) to afford the product **Xu13-2** as a white liquid (2.8 g, 52% yield).^{1}H NMR (400 MHz, Chloroform-*d*) δ 9.82 (s, 1H), 7.95 – 7.27 (m, 2H), 6.96 (d, *J* = 8.3 Hz, 1H), 6.06 (s, 1H), 4.74 (h, *J* = 6.1 Hz, 1H), 1.42 (d, *J* = 6.1 Hz, 6H).^{13}C NMR (101 MHz, CDCl$_3$) δ 191.0, 150.1, 146.7, 130.1, 124.3, 114.2, 111.9, 71.9, 21.9.HRMS (ESI) calculated for [$C_{10}H_{12}NaO_3$] [M+Na]$^+$: 203.0679 found: 203.0678.

2.5.3 2-bromo-4-ethoxy-3-hydroxybenzaldehyde (Xu12-3)

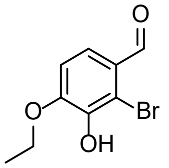

Xu12-3

Prepared from 4-ethoxy-3-hydroxybenzaldehyde (Xu12-2) (15 mmol) in 1,4-Dioxane/H_2O (v:v=1:1, 20 mL) was added NBS slowly (15.75 mmol, 1.05 equiv.). The resulting solution was stirred at room temperature overnight. The reaction mixture was diluted with EtOAc. The organic layer was separated, and the aqueous layer was extracted twice with EtOAc. The combined organic layers were dried over Na_2SO_4, filtered, concentrated. the crude product was then purified by flash column chromatography on silica gel (petroleum ether: ethyl acetate = 10: 1) to afford the product **Xu12-3** as a white liquid (2.4 g, 75% yield). ^{1}H NMR (400 MHz, Chloroform-*d*) δ 10.26 (s, 1H), 7.55 (d, *J* = 8.5 Hz, 1H), 6.90 (d, *J* = 8.5 Hz, 1H), 6.28 (s, 1H), 4.24 (q, *J* = 7.0 Hz, 2H), 1.51 (t, *J* = 7.0 Hz, 3H).^{13}C NMR (100 MHz, Chloroform-*d*) δ 191.0, 151.0, 143.3, 127.0, 122.6, 112.8, 109.8, 65.4, 14.6. HRMS (ESI) calculated for [$C_9H_9BrNaO_3$] [M+H]$^+$: 266.9634 found: 266.9627.

2.5.4 2-bromo-3-hydroxy-4-isopropoxybenzaldehyde (Xu13-3)

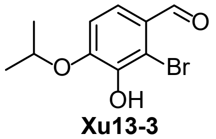

Xu13-3

Prepared from 3-hydroxy-4-isopropoxybenzaldehyde (Xu13-2) (15 mmol) in 1,4-Dioxane/H_2O (v:v=1:1, 20 mL) was added NBS slowly (15.75 mmol, 1.05 equiv.). The resulting solution was stirred at room temperature overnight. The reaction mixture was diluted with EtOAc. The organic layer was separated, and the aqueous layer was extracted twice with EtOAc. The combined organic layers were dried over Na_2SO_4, filtered, concentrated. the crude product was then purified by flash

column chromatography on silica gel (petroleum ether: ethyl acetate = 10: 1) to afford the product **Xu13-3** as a white liquid (3 g, 80% yield).^{1}H NMR (400 MHz, Chloroform-*d*) δ 10.26 (s, 1H), 7.55 (d, *J* = 8.6 Hz, 1H), 6.90 (d, *J* = 8.5 Hz, 1H), 6.17 (s, 1H), 4.74 (p, *J* = 6.0 Hz, 1H), 1.43 (d, *J* = 6.0 Hz, 8H).^{13}C NMR (100 MHz, $CDCl_3$) δ 191.0, 150.0, 143.9, 126.9, 122.4, 112.9, 110.9, 72.7, 22.0.

2.5.5 3-(benzyloxy)-2-bromo-4-ethoxybenzaldehyde (Xu12-4)

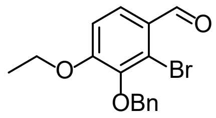

Xu12-4

To a solution of 2-bromo-4-ethoxy-3-hydroxybenzaldehyde (Xu12-3) (10 mmol) in DMF (20 mL) was added benzyl bromide (12mmol, 1.2 equiv.) and K_2CO_3 (15 mmol, 1.5 equiv.). The resulting solution was stirred at room temperature overnight. The reaction mixture was quenched by the addition of H_2O and diluted with EtOAc. The organic layer was separated, and the aqueous layer was extracted twice with EtOAc. The combined organic layers were dried over Na_2SO_4, filtered, concentrated. The crude product was then washed by petroleum ether with a little ethyl acetate to afford the product **Xu12-4** as a white solid (2 g, 60% yield). Mp: 59.7 – 60.5 °C.^{1}H NMR (400 MHz, Chloroform-*d*) δ 10.28 (s, 1H), 7.75 (d, *J* = 8.7 Hz, 1H), 7.67 – 7.51 (m, 2H), 7.50 – 7.31 (m, 4H), 6.97 (d, *J* = 8.7 Hz, 1H), 5.09 (s, 2H), 4.20 (q, *J* = 7.0 Hz, 2H), 1.53 (t, *J* = 7.0 Hz, 4H).^{13}C NMR (101 MHz, $CDCl_3$) δ 191.0, 158.1, 145.1, 136.7, 128.5, 128.4, 128.3, 127.2, 126.5, 123.5, 111.6, 74.7, 64.9, 14.6. HRMS (ESI) calculated for [$C_{16}H_{15}BrNaO_3$] $[M+Na]^+$: 357.0097 found: 357.0096.

2.5.6 3-(benzyloxy)-2-bromo-4-isopropoxybenzaldehyde (Xu13-4)

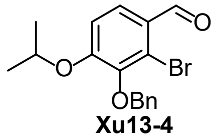

Xu13-4

To a solution of 2-bromo-3-hydroxy-4-isopropoxybenzaldehyde (Xu13-3) (10 mmol) in DMF (20 mL) was added benzyl bromide (12 mmol, 1.2 equiv.) and K_2CO_3 (15 mmol, 1.5 equiv.). The resulting solution was stirred at room temperature overnight. The reaction mixture was quenched by the addition of H_2O and diluted with EtOAc. The organic layer was separated, and the aqueous layer was extracted twice with EtOAc. The combined organic layers were dried over Na_2SO_4,

filtered, concentrated. The crude product was then washed by petroleum ether with a little ethyl acetate to afford the product **Xu13-4** as a white solid (2.6 g, 76% yield). Mp: 72.8 – 73.5 °C. ^{1}H NMR (400 MHz, Chloroform-*d*) δ 10.28 (s, 1H), 7.75 (d, *J* = 8.7 Hz, 1H), 7.58 (d, *J* = 7.0 Hz, 2H), 7.41 (dt, *J* = 12.1, 6.8 Hz, 3H), 6.98 (d, *J* = 8.7 Hz, 1H), 5.06 (s, 2H), 4.75 (p, *J* = 6.1 Hz, 1H), 1.45 (d, *J* = 6.0 Hz, 6H).^{13}C NMR (101 MHz, $CDCl_3$) δ 191.0, 157.2, 145.8, 136.8, 128.6, 128.4, 128.3, 127.0, 126.4, 123.8, 112.8, 74.7, 71.7, 21.9. HRMS (ESI) calculated for [$C_{17}H_{17}BrNaO_3$] $[M+Na]^+$: 371.0253 found: 371.0249.

2.5.7 2-(benzyloxy)-3-bromo-4-(dimethoxymethyl)-1-ethoxybenzene (Xu12-5)

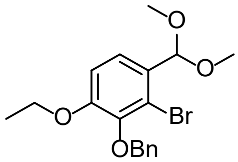

Xu12-5

Prepared from 3-(benzyloxy)-2-bromo-4-ethoxybenzaldehyde (Xu12-4) (3 mmol) in trimethoxymethane (30 mmol, 10 equiv.) and *p*-toluenesulfonic acid (0.15 mmol, 5% mol). The resulting solution was stirred 5 hours at 60 °C. The reaction mixture was washed by the addition of $NaHCO_3$ (aq.) and the combined organic layers concentrated. The crude product was purified by flash column chromatography on aluminum oxide (petroleum ether: ethyl acetate = 20: 1) to afford the product **Xu12-5** as a white liquid (1.0 g, 90% yield). ^{1}H NMR (400 MHz, Chloroform-*d*) δ 7.61 (d, *J* = 7.1 Hz, 1H), 7.49 – 7.31 (m, 2H), 6.93 (d, *J* = 8.6 Hz, 0H), 5.33 (d, *J* = 204.6 Hz, 1H), 4.12 (q, *J* = 7.0 Hz, 1H), 3.41 (s, 3H), 1.48 (t, *J* = 7.0 Hz, 1H).^{13}C NMR (101 MHz, $CDCl_3$) δ 153.1, 145.2, 137.1, 129.7, 129.6, 128.3, 128.1, 127.9, 123.3, 119.0, 111.7, 102.9, 74.4, 64.4, 53.7, 14.7.HRMS (ESI) calculated for [$C_{18}H_{21}BrNaO_4$] $[M+Na]^+$: 403.0515 found: 403.0518.

2.5.8 2-(benzyloxy)-3-bromo-4-(dimethoxymethyl)-1-isopropoxybenzene (Xu13-5)

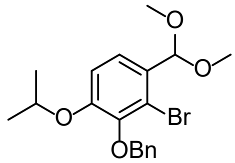

Xu13-5

Prepared from 3-(benzyloxy)-2-bromo-4-isopropoxybenzaldehyde (Xu13-4) (3 mmol) in trimethoxymethane (30 mmol, 10 equiv.) and *p*-toluenesulfonic acid (0.15 mmol, 5% mol). The resulting solution was stirred 5 hours at 60 °C. The reaction mixture was washed by the addition of

$NaHCO_3$ (aq.) and the combined organic layers concentrated. The crude product was purified by flash column chromatography on aluminum oxide (petroleum ether: ethyl acetate = 20: 1) to afford the product **Xu13-5** as a white liquid (1.0 g, 90% yield).^{1}H NMR (400 MHz, Chloroform-*d*) δ 7.56 (d, J = 7.4 Hz, 2H), 7.36 (t, J = 7.3 Hz, 2H), 7.31 (d, J = 7.9 Hz, 2H), 6.89 (d, J = 8.6 Hz, 1H), 5.53 (s, 1H), 5.01 (s, 2H), 4.57 (p, J = 6.1 Hz, 1H), 3.36 (s, 6H), 1.33 (d, J = 6.1 Hz, 6H).^{13}C NMR (101 MHz, $CDCl_3$) δ 151.9, 146.1, 137.2, 129.8, 128.4, 128.3, 128.2, 128.1, 127.8, 123.1, 119.2, 113.9, 103.0, 74.3, 71.3, 53.7, 21.9. HRMS (ESI) calculated for [$C_{19}H_{24}O_4Br$] $[M+H]^+$: 394.0780 found: 394.0781.

2.5.9(2-(benzyloxy)-6-(dimethoxymethyl)-3-ethoxyphenyl)dicyclohexylphosphane (Xu12-6)

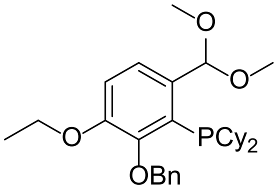

Xu12-6

Prepared from (((3-bromo-4-(dimethoxymethyl)-1,2-phenylene)bis(oxy))bis(methylene))dibenzene (**Xu12-5**) (2 mmol) in 4 mL anhydrous THF, was added *n*-BuLi (2.4 mmol, 1.6 M in hexane) dropwise under argon at -78 °C, The resulting solution at this temperature during 1 hour, and dicyclohexylchlorophosphine (2.6 mmol, 1.3 equiv.) was added dropwise. The reaction mixture was warmed to room temperature overnight. The re action mixture was quenched by the addition of $NaHCO_3$ (aq.) and diluted with EtOAc. The organic layer was separated, and the aqueous layer was extracted twice with EtOAc. The combined organic layers were concentrated. The crude product was added to next step.

2.5.10(2-(benzyloxy)-6-(dimethoxymethyl)-3-isopropoxyphenyl)dicyclohexylphosphane(Xu13-6)

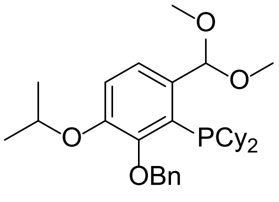

Xu13-6

Prepared from 2-(benzyloxy)-3-bromo-4-(dimethoxymethyl)-1-isopropoxybenzene (Xu13-5) (2 mmol) in 4 mL anhydrous THF, was added *n*-BuLi (2.4 mmol, 1.6 M in hexane) dropwise under

argon at -78 °C, The resulting solution at this temperature during 1 hour, and dicyclohexylchlorophosphine (2.6 mmol, 1.3 equiv.) was added dropwise. The reaction mixture was warmed to room temperature overnight. The re action mixture was quenched by the addition of $NaHCO_3$ (aq.) and diluted with EtOAc. The organic layer was separated, and the aqueous layer was extracted twice with EtOAc. The combined organic layers were concentrated. The crude product was added to next step.

2.5.11 3-(benzyloxy)-2-(dicyclohexylphosphaneyl)-4-ethoxybenzaldehyde (Xu12-7)

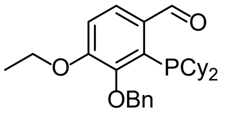

Xu12-7

Prepared from (2-(benzyloxy)-6-(dimethoxymethyl)-3-ethoxyphenyl)dicyclohexylphosphane (Xu12-6) in 2 mL THF, was added 1 mL HCl (1.0 M) under argon at 60 °C. The resulting solution was stirred 5 hours. The reaction mixture was quenched by the addition of $NaHCO_3$ (aq.) and diluted with EtOAc. The organic layer was separated, and the aqueous layer was extracted twice with EtOAc. The combined organic layers were dried over Na_2SO_4, filtered, concentrated and purified by flash column chromatography on silica gel (petroleum ether: ethyl acetate = 20: 1) to afford the product **Xu12-7** as a yellow liquid. ^1H NMR (400 MHz, Chloroform-*d*) δ 11.07 (d, *J* = 8.8 Hz, 1H), 7.76 (dd, *J* = 8.6, 3.2 Hz, 1H), 7.52 (d, *J* = 7.5 Hz, 2H), 7.42 (t, *J* = 7.5 Hz, 2H), 7.34 (t, *J* = 7.3 Hz, 1H), 7.04 (d, *J* = 8.6 Hz, 1H), 5.24 (s, 2H), 4.13 (q, *J* = 7.0 Hz, 2H), 2.37 (tdt, *J* = 11.5, 7.5, 3.4 Hz, 2H), 1.86 – 1.76 (m, 2H), 1.75 – 1.65 (m, 2H), 1.59 (d, *J* = 6.9 Hz, 4H), 1.40 (t, *J* = 7.0 Hz, 4H), 1.29 – 1.13 (m, 8H), 0.99 (tdd, *J* = 13.1, 6.0, 2.8 Hz, 2H), 0.86 (tt, *J* = 9.9, 5.6 Hz, 1H).^{13}C NMR (100 MHz, $CDCl_3$) δ 193.5, 193.1, 155.3, 150.6, 138.2, 136.4, 136.3, 133.5, 133.1, 128.2, 127.5, 127.2, 124.3, 124.2, 114.0, 73.7, 64.3, 34.5 (d, *J* = 11.6 Hz), 32.4 (d, *J* = 23.3 Hz), 30.8 (d, *J* = 9.7 Hz), 26.8 (d, *J* = 8.8 Hz), 26.6 (d, *J* = 14.1 Hz) 26.1, 14.5.^{31}P NMR (162 MHz, $CDCl_3$) δ -13.99. HRMS (ESI) calculated for [$C_{28}H_{38}O_3P$] $[M+H]^+$: 453.2553 found: 453.2556.

2.5.12 3-(benzyloxy)-2-(dicyclohexylphosphaneyl)-4-isopropoxybenzaldehyde (Xu13-7)

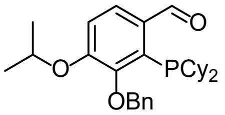

Xu13-7

Prepared from (2-(benzyloxy)-6-(dimethoxymethyl)-3-isopropoxyphenyl)dicyclohexylphosphane(Xu13-6) in 2 mL THF, was added 1 mL HCl (1.0 M) under argon at 60 °C. The resulting solution was stirred 5 hours. The reaction mixture was quenched by the addition of $NaHCO_3$ (aq.) and diluted with EtOAc. The organic layer was separated, and the aqueous layer was extracted twice with EtOAc. The combined organic layers were dried over Na_2SO_4, filtered, concentrated and purified by flash column chromatography on silica gel (petroleum ether: ethyl acetate = 20: 1) to afford the product **Xu13-7** as a yellow liquid.^{1}H NMR (400 MHz, Chloroform-*d*) δ 11.08 (d, *J* = 8.8 Hz, 1H), 7.77 (dt, *J* = 8.7, 2.4 Hz, 1H), 7.53 (d, *J* = 7.6 Hz, 2H), 7.49 – 7.41 (m, 2H), 7.41 – 7.32 (m, 1H), 7.07 (d, *J* = 8.6 Hz, 1H), 5.23 (s, 2H), 4.69 (hept, *J* = 6.3 Hz, 1H), 2.49 – 2.28 (m, 2H), 1.90 – 1.79 (m, 2H), 1.77 – 1.69 (m, 2H), 1.65 – 1.55 (m, 4H), 1.36 (dd, *J* = 6.2, 1.5 Hz, 7H), 1.32 – 1.10 (m, 9H), 1.07 – 0.95 (m, 2H).^{13}C NMR (101 MHz, $CDCl_3$) δ 193.18 (d, *J* = 42.2 Hz), 154.22, 151.42, 138.23, 136.22 (d, *J* = 15.3 Hz), 133.58 (d, *J* = 37.5 Hz), 128.15, 127.46, 127.07, 124.05 (d, *J* = 6.1 Hz), 115.49, 73.62, 71.08, 34.47 (d, *J* = 11.6 Hz), 32.38 (d, *J* = 23.5 Hz), 30.75 (d, *J* = 10.0 Hz), 26.74 (d, *J* = 9.0 Hz), 26.54 (d, *J* = 13.9 Hz), 26.11, 21.81. ^{31}P NMR (162 MHz, $CDCl_3$) δ -13.95. HRMS (ESI) calculated for [$C_{29}H_{40}O_3P$] $[M+H]^+$: 467.2710 found: 467.2715.

2.5.13(*R,E*)-*N*-(3-(benzyloxy)-2-(dicyclohexylphosphaneyl)-4-ethoxybenzylidene)-2-methylpropane-2-sulfinamide (Xu12-8)

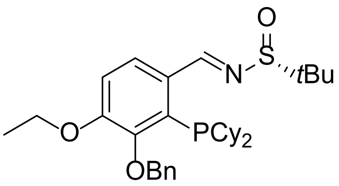

Xu12-8

Prepared from 3-(benzyloxy)-2-(dicyclohexylphosphaneyl)-4-ethoxybenzaldehyde (Xu12-7) in 2 mL THF, was added (*R*)-2-methylpropane-2-sulfinamide (1.2 mmol, 1.2 equiv.) and titanium tetraisopropanolate (2 mmol, 2.0 equiv.) under argon at 50 °C. The resulting solution was stirred 8 hours. The reaction mixture was quenched by the addition of H_2O (aq.) and diluted with EtOAc. The solution was filtered and the residue was washed twice with EtOAc. The combined organic layers were dried over Na_2SO_4, filtered, concentrated and purified by flash column chromatography on silica gel (petroleum ether: ethyl acetate = 10: 1) to afford the crude product **Xu12-8** as a yellow

solid. Mp: 56.6-57.5 °C. $[\alpha]_D^{20}$=-58.9(c = 0.4, Chloroform). ^{1}H NMR (400 MHz, Chloroform-d) δ 9.67 (s, 1H), 7.86 (dd, J = 8.6, 2.8 Hz, 1H), 7.55 (d, J = 7.5 Hz, 2H), 7.43 (t, J = 7.5 Hz, 2H), 7.35 (t, J = 7.3 Hz, 1H), 7.04 (d, J = 8.6 Hz, 1H), 5.23 (s, 2H), 4.20 – 4.07 (m, 2H), 2.40 – 2.26 (m, 2H), 1.87 – 1.77 (m, 2H), 1.71 (d, J = 7.4 Hz, 2H), 1.59 (d, J = 6.9 Hz, 4H), 1.42 (t, J = 7.0 Hz, 3H), 1.36 (d, J = 3.5 Hz, 2H), 1.28 (s, 9H), 1.17 (d, J = 7.1 Hz, 7H), 1.08 – 0.93 (m, 2H).^{13}C NMR (101 MHz, $CDCl_3$) δ 164.1, 163.7, 153.9, 151.1, 138.2, 134.2, 133.1, 132.7, 128.2, 127.5, 127.4, 124.2, 114.3, 73.8, 64.2, 57.5, 34.8 (t, J = 13.3 Hz), 32.3 (d, J = 23.7 Hz), 30.6 (dd, J = 9.7, 7.4 Hz), 27.1 – 26.3(m). 26.2, 22.6, 14.6. ^{31}P NMR (162 MHz, $CDCl_3$) δ -9.32. HRMS (ESI) calculated for [$C_{32}H_{46}NNaO_3PS$] $[M+Na]^+$: 578.2828 found: 578.2829.

2.5.14(*R*,*E*)-*N*-(3-(benzyloxy)-2-(dicyclohexylphosphaneyl)-4-isopropoxybenzylidene)-2-methylpropane-2-sulfinamide (Xu13-8)

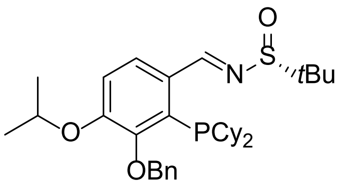

Xu13-8

Prepared from 3-(benzyloxy)-2-(dicyclohexylphosphaneyl)-4-isopropoxybenzaldehyde (Xu13-7) in 2 mL THF, was added (R)-2-methylpropane-2-sulfinamide (1.2 mmol, 1.2 equiv.) and titanium tetraisopropanolate (2 mmol, 2.0 equiv.) under argon at 50 °C. The resulting solution was stirred 8 hours. The reaction mixture was quenched by the addition of H_2O (aq.) and diluted with EtOAc. The solution was filtered and the residue was washed twice with EtOAc. The combined organic layers were dried over Na_2SO_4, filtered, concentrated and purified by flash column chromatography on silica gel (petroleum ether: ethyl acetate = 10: 1) to afford the crude product **Xu13-8** as a yellow solid. Mp: 73.8-74.5 °C. $[\alpha]_D^{20}$ = -79.9 (c = 0.4, Chloroform).^{1}H NMR (400 MHz, Chloroform-d) δ 9.64 (s, 1H), 7.82 (dd, J = 8.7, 2.8 Hz, 1H), 7.52 (d, J = 7.4 Hz, 2H), 7.41 (t, J = 7.5 Hz, 2H), 7.37 – 7.29 (m, 1H), 7.02 (d, J = 8.6 Hz, 1H), 5.18 (s, 2H), 4.63 (hept, J = 6.1 Hz, 1H), 2.31 (ddt, J = 14.8, 11.2, 8.3 Hz, 2H), 1.86 – 1.75 (m, 2H), 1.70 (d, J = 7.0 Hz, 2H), 1.57 (d, J = 7.1 Hz, 4H), 1.33 (d, J = 6.0 Hz, 8H), 1.26 (s, 9H), 1.25 – 1.05 (m, 8H), 1.05 – 0.91 (m, 2H).^{13}C NMR (101 MHz, $CDCl_3$) δ 163.83 (d, J = 34.9 Hz), 152.7, 152.0, 138.3, 134.3, 133.1 (d, J = 36.1 Hz), 128.1, 127.4, 127.2, 124.0, 116.1, 73.7, 71.1, 57.4, 35.1 – 34.4 (m), 32.3 (d, J = 23.8 Hz), 30.6 (dd, J = 9.8, 7.5 Hz),

26.8 (d, J = 8.6 Hz), 26.6 (dd, J = 13.9, 4.5 Hz), 26.1, 22.6, 21.9, 21.9. ^{31}P NMR (162 MHz, $CDCl_3$) δ -9.37. HRMS (ESI) calculated for [$C_{33}H_{49}NO_3PS$] [M+H]$^+$: 570.3165 found: 570.3161.

2.5.15(*R*)-*N*-((*S*)-[1,1'-biphenyl]-4-yl(3-(benzyloxy)-2-(dicyclohexylphosphaneyl)-4-ethoxyphenyl)methyl)-2-methylpropane-2-sulfinamide (Xu12-9)

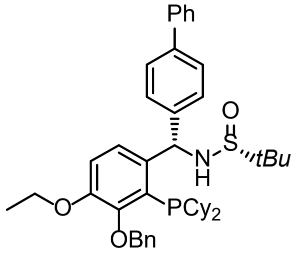

Xu12-9

Prepared from (*R*,*E*)-*N*-(3-(benzyloxy)-2-(dicyclohexylphosphaneyl)-4-ethoxybenzylidene)-2-methylpropane-2-sulfinamide (Xu12-8) in 1 mL anhydrous THF, was added [1,1'-biphenyl]-4-ylmagnesium bromide (1 mmol, 2.0 equiv.) dropwise under argon at -50 °C. The reaction mixture was warmed to room temperature overnight and was quenched by the addition of NH_4Cl (aq.) and diluted with EtOAc. The organic layer was separated, and the aqueous layer was extracted twice with EtOAc. The combined organic layers were dried over Na_2SO_4, filtered, concentrated and purified by flash column chromatography on silica gel (petroleum ether: ethyl acetate = 5: 1) to the product **Xu12-9** as a white solid (230 mg, 65% yield). Mp: 81.2-82.1 °C. $[\alpha]_D^{20}$ = -87.5 (c = 0.4, Chloroform).^{1}H NMR (400 MHz, Chloroform-*d*) δ 7.61 – 7.49 (m, 8H), 7.43 (p, J = 6.3, 5.5 Hz, 5H), 7.39 – 7.32 (m, 2H), 7.09 (d, J = 8.6 Hz, 1H), 7.02 – 6.90 (m, 1H), 5.37 (d, J = 11.8 Hz, 1H), 5.26 (d, J = 11.8 Hz, 1H), 4.10 (q, J = 7.0 Hz, 3H), 2.38 – 2.22 (m, 2H), 1.81 – 1.58 (m, 6H), 1.52 (d, J = 12.3 Hz, 1H), 1.39 (q, J = 6.7 Hz, 5H), 1.31 (s, 9H), 1.11 (dddd, J = 34.0, 23.7, 15.1, 5.0 Hz, 9H), 0.91 (dq, J = 8.8, 3.8 Hz, 2H).^{13}C NMR (101 MHz, $CDCl_3$) δ 151.0, 149.8, 142.5, 142.0, 141.8, 141.0, 140.0, 138.8, 129.2, 129.1, 128.7, 128.6, 128.4, 128.1, 127.2, 127.1, 127.0, 126.9, 126.9, 123.1, 123.1, 114.9, 73.2, 64.1, 60.1 (d, J = 34.3 Hz), 55.8, 35.3 (d, J = 10.8 Hz), 34.4 (d, J = 11.3 Hz), 33.1 (d, J = 25.7 Hz), 32.3 (d, J = 23.1 Hz), 30.5 (d, J = 10.6 Hz), 30.3 (d, J = 8.1 Hz),27.1, 27.0, 26.9 (d, J = 5.8 Hz), 26.8 (d, J = 6.2 Hz), 26.7 (d, J = 7.4 Hz), 26.2, 22.7, 14.8.^{31}P NMR (162 MHz, $CDCl_3$) δ -6.67. HRMS (ESI) calculated for [$C_{44}H_{57}NO_3PS$] [M+H]$^+$: 710.3791 found: 710.3795.

2.5.16(*R*)-*N*-((*S*)-[1,1'-biphenyl]-4-yl(3-(benzyloxy)-2-(dicyclohexylphosphaneyl)-4-isopropoxyphenyl)methyl)-2-methylpropane-2-sulfinamide (Xu13-9)

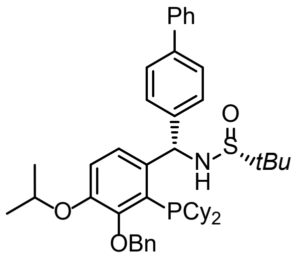

Xu13-9

Prepared from (*R*,*E*)-*N*-(3-(benzyloxy)-2-(dicyclohexylphosphaneyl)-4-isopropoxybenzylidene)-2-methylpropane-2-sulfinamide (Xu13-8) in 1 mL anhydrous THF, was added [1,1'-biphenyl]-4-ylmagnesium bromide (1 mmol, 2.0 equiv.) dropwise under argon at -50 °C. The reaction mixture was warmed to room temperature overnight and was quenched by the addition of NH_4Cl (aq.) and diluted with EtOAc. The organic layer was separated, and the aqueous layer was extracted twice with EtOAc. The combined organic layers were dried over Na_2SO_4, filtered, concentrated and purified by flash column chromatography on silica gel (petroleum ether: ethyl acetate = 5: 1) to the product **Xu13-9** as a white solid (198 mg, 55% yield). Mp: 73.8-74.5 °C. $[\alpha]_D^{20}$= -72.5 (*c* = 0.4, Chloroform).^{1}H NMR (400 MHz, Chloroform-*d*) δ 7.61 – 7.48 (m, 8H), 7.48 – 7.32 (m, 7H), 7.09 (d, *J* = 8.5 Hz, 1H), 7.02 – 6.85 (m, 1H), 5.35 (d, *J* = 11.8 Hz, 1H), 5.24 (d, *J* = 11.9 Hz, 1H), 4.58 (h, *J* = 6.1 Hz, 1H), 4.06 (s, 1H), 2.29 (p, *J* = 11.6 Hz, 2H), 1.81 – 1.58 (m, 6H), 1.39 (q, *J* = 6.5, 4.6 Hz, 2H), 1.32 (d, *J* = 6.1 Hz, 15H), 1.25 – 0.88 (m, 12H).^{13}C NMR (101 MHz, $CDCl_3$) δ 152.2, 148.6, 142.5, 142.2 (d, *J* = 23.7 Hz), 141.0, 140.0, 138.9, 129.2, 129.1, 128.6, 128.1, 127.2, 127.1, 127.0, 126.9, 126.8, 123.1, 123.1, 117.3, 73.2, 71.3, 60.2 (d, *J* = 35.4 Hz), 55.8, 35.3 (d, *J* = 10.6 Hz), 34.5 (d, *J* = 11.3 Hz), 33.1 (d, *J* = 25.9 Hz), 32.3 (d, *J* = 23.0 Hz), 30.5 (d, *J* = 10.3 Hz), 30.4 (d, *J* = 7.9 Hz), 27.1, 27.0 (d, *J* = 2.8 Hz) , 27.0, 26.9 (d, *J* = 5.9 Hz), 26.7 (d, *J* = 7.1 Hz), 26.2, 22.7, 22.1, 22.1. ^{31}P NMR (162 MHz, $CDCl_3$) δ -6.78. HRMS (ESI) calculated for [$C_{45}H_{59}NO_3PS$] $[M+H]^+$: 724.3948 found: 724.3945.

2.5.17(*R*)-*N*-((*S*)-[1,1'-biphenyl]-4-yl(3-(benzyloxy)-2-(dicyclohexylphosphaneyl)-4-ethoxyphenyl)methyl)-N,2-dimethylpropane-2-sulfinamide (Xu12)

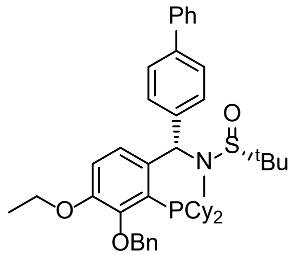

Xu12

Prepared from (*R*)-*N*-((*S*)-[1,1'-biphenyl]-4-yl(3-(benzyloxy)-2-(dicyclohexylphosphaneyl)-4-ethoxyphenyl)methyl)-2-methylpropane-2-sulfinamide (Xu12-9) in 1 mL anhydrous THF, was added *n*-BuLi (0.5 mmol, 1.6 M in hexane) dropwise under argon at -40 °C. The resulting solution at this temperature during 1 hour and iodomethane (0.6 mmol, 2 equiv.) was added. After 1 hour, the reaction mixture moved to 0 °C and stirred 1 hour. The reaction mixture was the addition of NH_4Cl (aq.) and diluted with EtOAc. The organic layer was separated, and the aqueous layer was extracted twice with EtOAc. The combined organic layers were dried over Na_2SO_4, filtered, concentrated and purified by flash column chromatography on silica gel (petroleum ether: ethyl acetate = 5: 1) to the product **Xu12** as a white solid (113 mg, 52% yield). Mp: 80.1-80.9 °C. $[\alpha]_D^{20}$ = 13.6 (*c* = 0.4, Chloroform).^{1}H NMR (400 MHz, Chloroform-*d*) δ 7.59 (d, *J* = 7.3 Hz, 3H), 7.51 (d, *J* = 7.9 Hz, 4H), 7.43 (q, *J* = 8.0 Hz, 4H), 7.37 – 7.28 (m, 4H), 7.10 (t, *J* = 10.6 Hz, 2H), 5.31 (s, 2H), 4.11 (q, *J* = 7.1 Hz, 2H), 2.69 (s, 3H), 2.48 – 2.35 (m, 1H), 2.18 – 2.07 (m, 1H), 1.76 – 1.64 (m, 4H), 1.57 – 1.51 (m, 1H), 1.48 – 1.42 (m, 2H), 1.39 (d, *J* = 6.9 Hz, 3H), 1.26 (dt, *J* = 22.5, 8.5 Hz, 4H), 1.14 (s, 9H), 1.02 – 0.86 (m, 9H).^{13}C NMR (101 MHz, $CDCl_3$) δ 151.1, 151.0, 149.7, 140.9, 140.5, 140.2, 139.8, 139.5, 138.8, 131.7, 128.7, 128.6, 128.4, 128.1, 127.2, 127.0, 126.9, 126.9, 126.3, 122.8, 122.8, 114.9, 73.3, 70.5 (d, *J* = 40.0 Hz), 64.1, 58.6, 35.2 (d, *J* = 10.5 Hz), 34.1 (d, *J* = 11.6 Hz), 33.2 (d, *J* = 26.2 Hz), 32.2 (d, *J* = 22.5 Hz), 30.9 (d, *J* = 11.6 Hz),30.5 , 29.9 (d, *J* = 7.9 Hz), 27.0 (d, *J* = 8.9 Hz), 26.8 (d, *J* = 10.8 Hz), 26.6 (d, *J* = 3.0 Hz), 26.3, 26.1, 24.1, 14.7. ^{31}P NMR (162 MHz, $CDCl_3$) δ -6.10. HRMS (ESI) calculated for [$C_{45}H_{59}NO_3PS$] $[M+H]^+$: 724.3948 found: 724.3946.

2.5.8(*R*)-*N*-((*S*)-[1,1'-biphenyl]-4-yl(3-(benzyloxy)-2-(dicyclohexylphosphaneyl)-4-isopropoxyphenyl)methyl)-N,2-dimethylpropane-2-sulfinamide(Xu13)

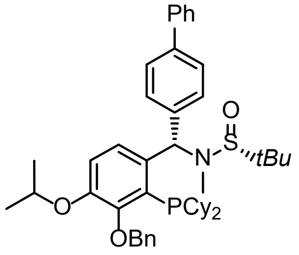

Xu13

Prepared from (R)-N-((S)-[1,1'-biphenyl]-4-yl(3-(benzyloxy)-2-(dicyclohexylphosphaneyl)-4-isopropoxyphenyl)methyl)-2-methylpropane-2-sulfinamide (Xu13-9) (Xu13-9) in 1 mL anhydrous THF, was added *n*-BuLi (0.5 mmol, 1.6 M in hexane) dropwise under argon at -40 °C. The resulting solution at this temperature during 1 hour and iodomethane (0.6 mmol, 2 equiv.) was added. After 1 hour, the reaction mixture moved to 0 °C and stirred 1 hour. The reaction mixture was the addition of NH_4Cl (aq.) and diluted with EtOAc. The organic layer was separated, and the aqueous layer was extracted twice with EtOAc. The combined organic layers were dried over Na_2SO_4, filtered, concentrated and purified by flash column chromatography on silica gel (petroleum ether: ethyl acetate = 5: 1) to the product **Xu13** as a white solid (118 mg, 60% yield). Mp: 82-82.7 °C. $[\alpha]_D^{20}$ = 10.1 (*c* = 0.4, Chloroform). ^{1}H NMR (400 MHz, Chloroform-*d*) δ 7.58 (d, *J* = 7.3 Hz, 2H), 7.54 (dd, *J* = 8.6, 4.1 Hz, 1H), 7.49 (t, *J* = 7.6 Hz, 4H), 7.45 – 7.40 (m, 3H), 7.39 (s, 1H), 7.34 (t, *J* = 7.3 Hz, 2H), 7.31 – 7.27 (m, 2H), 7.11 (d, *J* = 8.6 Hz, 1H), 7.05 (d, *J* = 11.5 Hz, 1H), 5.26 (s, 2H), 4.57 (p, *J* = 6.0 Hz, 1H), 2.67 (s, 3H), 2.38 (dtd, *J* = 11.5, 8.5, 3.8 Hz, 1H), 2.14 – 2.02 (m, 1H), 1.77 – 1.61 (m, 6H), 1.42 – 1.28 (m, 15H), 1.12 (s, 9H), 0.93 (ddt, *J* = 15.8, 10.1, 6.3 Hz, 5H).^{13}C NMR (101 MHz, $CDCl_3$) δ152.4 (d, *J* = 3.7 Hz),148.5, 141.0, 140.7 (d, *J* = 23.1 Hz), 139.8, 139.6, 138.9, 131.8, 128.7, 128.1, 127.2, 127.1, 127.1, 127.0, 126.9, 126.4, 122.9 (d, *J* = 5.8 Hz), 117.7, 73.3, 71.4, 70.5 (d, *J* = 40.2 Hz), 58.7, 35.3 (d, *J* = 10.3 Hz), 34.2 (d, *J* = 11.6 Hz), 33.2 (d, *J* = 26.2 Hz), 32.3 (d, *J* = 22.5 Hz), 31.5 (d, *J* = 7.2 Hz), 31.0 (d, *J* = 11.6 Hz), 30.6, 30.2 (d, *J* = 5.8 Hz), 23.0 (d, *J* = 8.0 Hz), 29.6 (d, *J* = 21.7 Hz), 27.0 (t, *J* = 9.1 Hz), 26.9 – 26.6 (m), 26.3 (d, *J* = 26.3 Hz), 24.2, 22.1, 22.0.^{31}P NMR (162 MHz, Chloroform-*d*) δ -6.25. HRMS (ESI) calculated for [$C_{46}H_{61}NO_3PS$] $[M+H]^+$: 738.4104 found: 738.4107.

3. General procedure for preparation of substrates

3.1 Synthesis of 1-(3-methylbut-3-en-1-yl)-cyclopentan-1-ol (1c)[2]

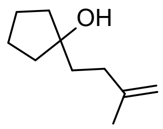

1c

To a solution of (3-methylbut-3-en-1-yl)-magnesium bromide (55 mmol, 1.1 equiv.) in dry Et_2O (30 mL), was added cyclopentanone (50 mmol, 1.0 equiv.) dropwise under argon at -30 °C. The reaction mixture was warmed to room temperature overnight. The reaction mixture was quenched by the addition of HCl (1 M) and diluted with EtOAc. The organic layer was separated and the aqueous layer was extracted twice with EtOAc. The combined organic layers were dried over Na_2SO_4, filtered, concentrated and purified by flash column chromatography on silica gel (petroleum ether: ethyl acetate = 20: 1) to the product **1c** as a white liquid (3.5 g, 45% yield). ^{1}H NMR (400 MHz, Chloroform-*d*) δ 4.69 (s, 2 H), 2.21 – 2.07 (m, 2 H), 1.83 – 1.76 (m, 2 H), 1.75 – 1.72 (m, 4 H), 1.70 (t, *J* = 3. 2 Hz, 1 H), 1.67 – 1.53 (m, 6 H). ^{13}C NMR (100 MHz, Chloroform-*d*) δ 146.5, 109.6, 82.4, 39.6, 39.3, 33.0, 23.7, 22.5. HRMS (ESI) calculated for [$C_{10}H_{18}ONa$] $[M+Na]^+$: 177.1250 found: 177.1252.

3.2 Synthesis of 1-(3-methylbut-3-en-1-yl)-cyclohexan-1-ol (1d)[2]

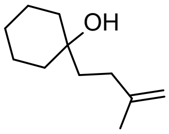

1d

To a solution of (3-methylbut-3-en-1-yl)-magnesium bromide (55 mmol, 1.1 equiv.) in dry Et_2O (30 mL), was added cyclohexanone (50 mmol, 1.0 equiv.) dropwise under argon at -30 °C. The reaction mixture was warmed to room temperature overnight. The reaction mixture was quenched by the addition of HCl (1 M) and diluted with EtOAc. The organic layer was separated and the aqueous layer was extracted twice with EtOAc. The combined organic layers were dried over Na_2SO_4, filtered, concentrated and purified by flash column chromatography on silica gel (petroleum ether: ethyl acetate = 20: 1) to the product **1d** as a white liquid (4.0 g, 48% yield). ^{1}H NMR (400 MHz, Chloroform-*d*) δ 4.69 (s, 2 H), 2.17 – 2.00 (m, 2 H), 1.73 (s, 3 H), 1.61 – 1.46 (m, 8 H), 1.46 – 1.37 (m, 3 H), 1.32 – 1.21 (m, 1 H). ^{13}C NMR (100 MHz, Chloroform-*d*) δ 146.5,

109.5, 71.3, 40.1, 37.3, 31.1, 25.8, 22.5, 22.1. HRMS (ESI) calculated for [$C_{11}H_{20}ONa$] $[M+Na]^+$: 191.1406 found: 191.1409.

Substrate **9a-9i**[3], **9j-9n**[4-5], **9o, 9p, 9r**[6], **9q**[7] were synthesized from reported procedure and the analytical data was consistent with the literature.

4. General Procedure and HPLC spectra

4.1 General Procedure for reactions of tertiary γ-hydroxyalkenes with alkenyl bromides

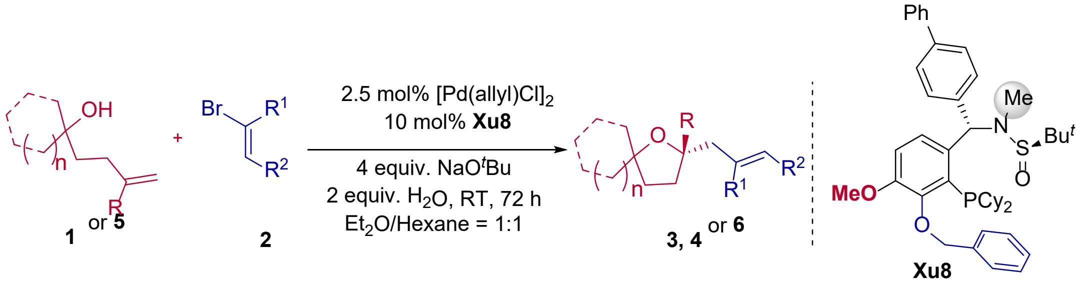

To a sealed tube was added $[Pd(allyl)Cl]_2$ (2.5 mol%), **Xu8** (10 mol%). The flask was evacuated and refilled with argon. Then tertiary γ-hydroxyalkenes (0.2 mmol), alkenyl halides (0.4 mmol), NaO^tBu (4.0 equiv.), H_2O (2.0 equiv.) and a mixed solution of Et_2O/Hexane (1: 1, 2 mL) was added to the tube, and stirred at room temperature for 72 hours. After the reaction was complete (monitored by TLC), solvent was removed under reduced pressure. The crude product was then purified by flash column chromatography on silica gel to afford the desired product.

4.1.1 (*S*, *E*)-2-(3-(4-methoxyphenyl)allyl)-1-oxaspiro[4.5]decane (3a)

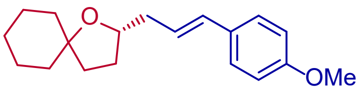

3a

Prepared according to typical procedure at RT for 72 hours by using $[Pd(allyl)Cl]_2$ (2.5 mol%), **Xu8** (10 mol%) from γ-hydroxyalkenes **1b** (0.2 mmol) and alkenyl bromide (0.4 mmol), after flash column chromatography on a silica gel (petroleum ether: ethyl acetate = 40: 1) give the product **3a** as a white liquid (42 mg, 74% yield) with 92% *ee*. $[\alpha]_D^{20}$ = -11.1 (*c* = 0.4, Chloroform). ^1H NMR (400 MHz, Chloroform-*d*) δ 7.34 – 7.26 (m, 2 H), 6.96 – 6.69 (m, 2 H), 6.38 (d, *J* = 15.6 Hz, 1 H), 6.17 – 5.99 (m, 1 H), 4.16 – 4.01 (m, 1 H), 3.8 0 (s, 3 H), 2.60 – 2.43 (m, 1 H), 2.43 – 2.20 (m, 1 H), 2.04 – 1.91 (m, 1 H), 1.82 – 1.61 (m, 5 H), 1.60 – 1.46 (m, 4 H), 1.45 – 1.31 (m, 4 H). ^{13}C NMR (100 MHz, Chloroform-*d*) δ 158.7, 131.1, 130.5, 127.0, 124.7, 113.8, 82.6, 77.5, 55.2, 39.8, 38.5,

37.6, 35.7, 30.6, 25.7, 24.1, 23.8. HRMS (ESI) calculated for [$C_{19}H_{26}NaO_2$] $[M+Na]^+$: 309.1825 found: 309.1828. Enantiomeric excess was determined by HPLC with a Chiralpak OJH column (hexanes: 2-propanol = 99: 1, 0.8 mL/min, 254 nm); major enantiomer tr = 15.62 min, minor enantiomer tr = 17.61 min.

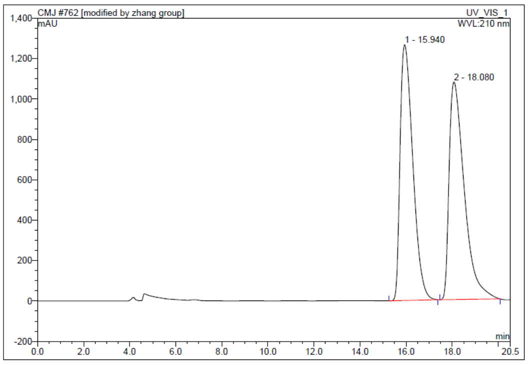

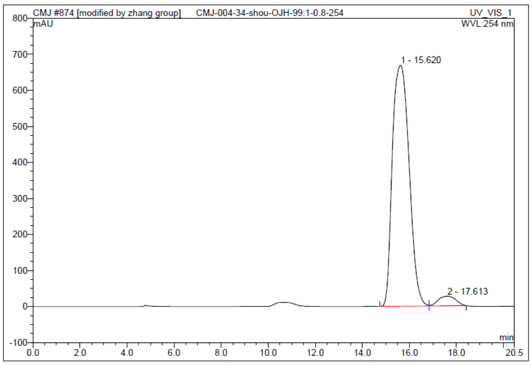

No.	Ret.Time min	Peak Name	Height mAU	Area mAU*min	Rel.Area %	Amount	Type
1	15.94	n.a.	1265.297	807.186	49.60	n.a.	BMB*
2	18.08	n.a.	1076.631	820.219	50.40	n.a.	BMB*
Total:			2341.928	1627.406	100.00	0.000	

No.	Ret.Time min	Peak Name	Height mAU	Area mAU*min	Rel.Area %	Amount	Type
1	15.62	n.a.	668.591	552.155	96.02	n.a.	BM *
2	17.61	n.a.	26.449	22.896	3.98	n.a.	MB*
Total:			695.040	575.051	100.00	0.000	

4.1.2 (*S*)-2-cinnamyl-1-oxaspiro[4.5]decane (3b)

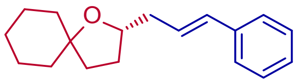

3b

Prepared according to typical procedure at RT for 72 hours by using [Pd(allyl)Cl]$_2$ (2.5 mol%), **Xu8** (10 mol%) from γ-hydroxyalkenes **1b** (0.2 mmol) and alkenyl bromide (0.4 mmol), after flash column chromatography on a silica gel (petroleum ether: ethyl acetate = 80: 1) give the product **3b** as a white liquid (31 mg, 61% yield) with 91% *ee*. $[\alpha]_D^{20}$ = -5.5 (*c* = 0.4, Chloroform).^{1}H NMR (500 MHz, Chloroform-*d*) δ 7.36 (d, *J* = 7.3 Hz, 2H), 7.30 (t, *J* = 7.7 Hz, 2H), 7.20 (t, *J* = 7.3 Hz, 1H), 6.44 (d, *J* = 15.8 Hz, 1H), 6.28 – 6.21 (m, 1H), 4.11 – 4.06 (m, 1H), 2.55 – 2.48 (m, 1H), 2.41 – 2.35 (m, 1H), 2.05 – 1.95 (m, 1H), 1.80 – 1.63 (m, 5H), 1.60 – 1.47 (m, 4H), 1.41 – 1.35 (m, 4H). ^{13}C NMR (125 MHz, Chloroform-*d*) δ 137.7, 131.8, 128.4, 126.9, 126.9, 126.0, 82.8, 77.4, 39.9, 38.5, 37.6, 35.7, 30.7, 25.7, 24.1, 23.8. HRMS (ESI) calculated for [$C_{18}H_{24}NaO$] $[M+Na]^+$: 279.1719 found: 279.1710. Enantiomeric excess was determined by HPLC with a Chiralpak OJH column (hexanes: 2-propanol = 99: 1, 0.6 mL/min, 254 nm); major enantiomer tr = 10.056 min, minor enantiomer tr = 11.487 min.

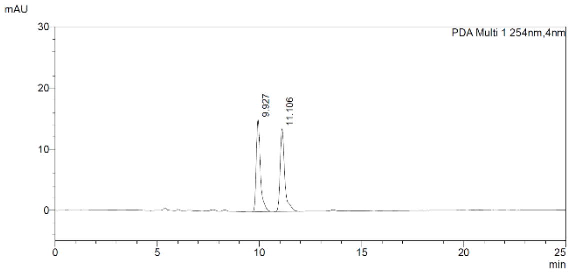

<Peak Table>

PDA Ch1 254nm

Peak#	Ret. Time	Height	Height%	Area	Area%
1	9.927	15076	52.676	211453	50.005
2	11.106	13544	47.324	211413	49.995
Total		28621	100.000	422866	100.000

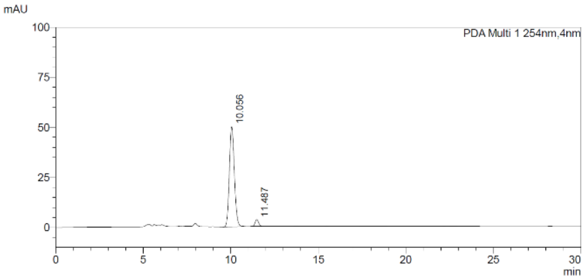

<Peak Table>

PDA Ch1 254nm

Peak#	Ret. Time	Height	Height%	Area	Area%
1	10.056	50075	93.709	992599	95.479
2	11.487	3362	6.291	46998	4.521
Total		53437	100.000	1039597	100.000

4.1.3 (*S*, *E*)-2-(3-(p-tolyl)-allyl)-1-oxaspiro[4.5]decane (3c)

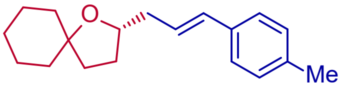

3c

Prepared according to typical procedure at RT for 72 hours by using $[Pd(allyl)Cl]_2$ (2.5 mol%), **Xu8** (10 mol%) from γ-hydroxyalkenes **1b** (0.2 mmol) and alkenyl bromide (0.4 mmol), after flash column chromatography on a silica gel (petroleum ether: ethyl acetate = 80: 1) give the product **3c** as a white liquid (26 mg, 51% yield) with 91% *ee*. $[\alpha]_D^{20}$ = -1.3 (*c* = 0.4, Chloroform). ^{1}H NMR (500 MHz, Chloroform-*d*) δ 7.27 (d, *J* = 9.5 Hz, 2H), 7.12 (d, *J* = 7.5 Hz, 2H), 6.42 (d, *J* = 16.0 Hz, 1H), 6.23 – 6.17 (m, 1H), 4.10 – 4.08 (m, 1H), 2.58 – 2.48 (m, 1H), 2.43 – 2.35 (m, 1H), 2.35 (s, 3H), 2.04 – 1.97 (m, 1H), 1.81 – 1.63 (m, 5H), 1.63 – 1.48 (m, 4H), 1.49 – 1.41 (m, 1H), 1.43 – 1.32 (m, 3H). ^{13}C NMR (125 MHz, Chloroform-d) δ 136.6, 134.9, 131.6, 129.1, 125.9, 125.8, 82.8, 77.5, 39.9, 38.5, 37.6, 35.7, 30.6, 25.7, 24.1, 23.8, 21.1. HRMS (ESI) calculated for $[C_{19}H_{26}NaO]$ $[M+Na]^+$: 293.1876 found: 293.1866. Enantiomeric excess was determined by HPLC with a Chiralpak OJH+OJ3 column (hexanes: 2-propanol = 99: 1, 0.3 mL/min, 254 nm); major enantiomer tr = 43.03 min, minor enantiomer tr = 51.09 min.

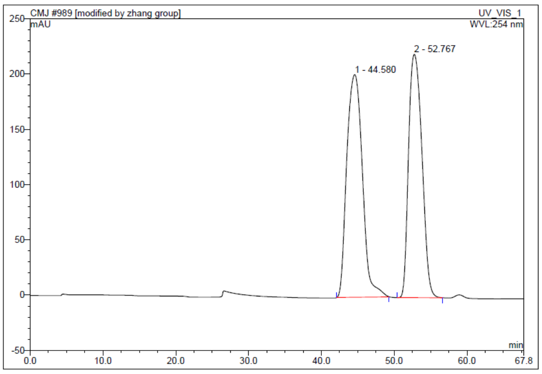

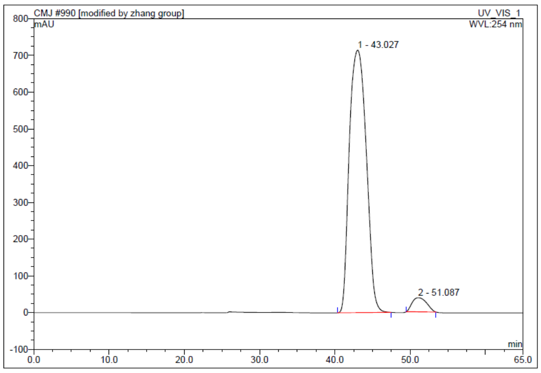

No.	Ret.Time min	Peak Name	Height mAU	Area mAU*min	Rel.Area %	Amount	Type
1	44.58	n.a.	201.460	501.693	51.77	n.a.	BM *
2	52.77	n.a.	220.085	467.392	48.23	n.a.	BMB*
Total:			421.545	969.085	100.00	0.000	

No.	Ret.Time min	Peak Name	Height mAU	Area mAU*min	Rel.Area %	Amount	Type
1	43.03	n.a.	714.243	1868.921	95.62	n.a.	BMB*
2	51.09	n.a.	38.309	85.587	4.38	n.a.	BMB*
Total:			752.552	1954.508	100.00	0.000	

4.1.4 (*S*, *E*)-2-(3-(m-tolyl)-allyl)-1-oxaspiro[4.5]decane (3d)

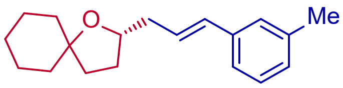

3d

Prepared according to typical procedure at RT for 72 hours by using [Pd(allyl)Cl]$_2$ (2.5 mol%), **Xu8** (10 mol%) from γ-hydroxyalkenes **1b** (0.2 mmol) and alkenyl bromide (0.4 mmol), after flash column chromatography on a silica gel (petroleum ether: ethyl acetate = 80: 1) give the product **3d** as a white liquid (40 mg, 78% yield) with 90% *ee*. $[\alpha]_D^{20}$ = -1.2 (*c* = 0.4, Chloroform). ^{1}H NMR (500 MHz, Chloroform-*d*) δ 7.23 – 7.12 (m, 3H), 7.02 (d, *J* = 7.5 Hz, 1H), 6.41 (d, *J* = 16 Hz, 1H), 6.28 – 6.18 (m, 1H), 4.12 – 4.03 (m, 1H), 2.57 – 2.48 (m, 1H), 2.42 – 2.34 (m, 1H), 2.34 (s, 3H), 2.04 – 1.95 (m, 1H), 1.80 – 1.61 (m, 5H), 1.62 – 1.46 (m, 4H), 1.48 – 1.31 (m, 4H). ^{13}C NMR (125 MHz, Chloroform-*d*) δ 137.9, 137.6, 131.8, 128.3, 127.6, 126.7, 126.6, 123.1, 82.7, 77.4, 39.9, 38.4, 37.6, 35.7, 30.6, 25.7, 24.1, 23.8, 21.3. HRMS (ESI) calculated for [$C_{19}H_{26}NaO$] [M+Na]$^+$: 293.1876 found: 293.1873. Enantiomeric excess was determined by HPLC with a Chiralpak OJH+OJ3 column (hexanes: 2-propanol = 99: 1, 0.3 mL/min, 254 nm); major enantiomer tr = 41.63 min, minor enantiomer tr = 49.69 min.

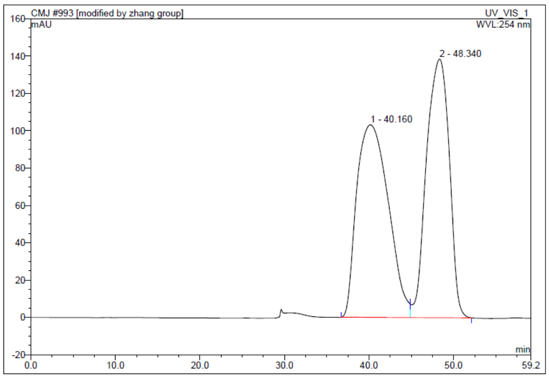

No.	Ret.Time min	Peak Name	Height mAU	Area mAU*min	Rel.Area %	Amount	Type
1	40.16	n.a.	103.156	448.941	50.82	n.a.	BM *
2	48.34	n.a.	138.496	434.444	49.18	n.a.	MB*
Total:			241.653	883.385	100.00	0.000	

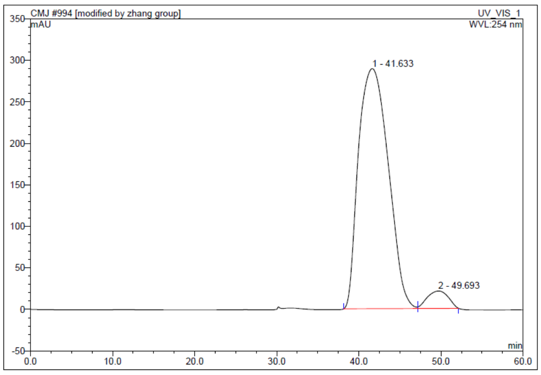

No.	Ret.Time min	Peak Name	Height mAU	Area mAU*min	Rel.Area %	Amount	Type
1	41.63	n.a.	289.180	1219.669	95.12	n.a.	BM *
2	49.69	n.a.	21.004	62.514	4.88	n.a.	MB*
Total:			310.184	1282.183	100.00	0.000	

4.1.5 (*S*, *E*)-2-(3-(2-methoxyphenyl)-allyl)-1-oxaspiro[4.5]decane (3e)

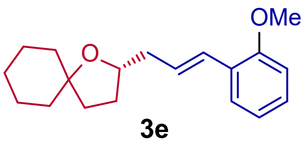

3e

Prepared according to typical procedure at RT for 72 hours by using $[Pd(allyl)Cl]_2$ (2.5 mol%), **Xu8** (10 mol%) from γ-hydroxyalkenes **1b** (0.2 mmol) and alkenyl bromide (0.4 mmol), after flash column chromatography on a silica gel (petroleum ether: ethyl acetate = 50: 1) give the product **3e** as a white liquid (44 mg, 77% yield) with 91% *ee*. $[\alpha]_D^{20}$ = -13.1 (*c* = 0.4, Chloroform) ^{1}H NMR (400 MHz, Chloroform-*d*) δ 7.42 (dd, *J* = 7.6, 1.6 Hz, 1H), 7.21 – 7.16 (m, 1H), 6.90 (t, *J* = 7.6 Hz, 1H), 6.85 (d, *J* = 8.4 Hz, 1H), 6.76 (d, *J* = 16.0 Hz, 1H), 6.26 – 6.18 (m, 1H), 4.11 – 4.05 (m, 1H), 3.84 (s, 3H), 2.58 – 2.52 (m, 1H), 2.43 – 2.36 (m, 1H), 2.05 – 1.95 (m, 1H), 1.77 – 1.63 (m, 5H), 1.60 – 1.47 (m, 4H), 1.45 – 1.31 (m, 4H). ^{13}C NMR (100 MHz, Chloroform-*d*) δ 156.3, 127.9, 127.6, 126.8, 126.4, 126.4, 120.6, 110.8, 82.7, 77.6, 55.5, 40.3, 38.5, 37.6, 35.8, 30.7, 25.7, 24.1, 23.8. HRMS (ESI) calculated for $[C_{19}H_{26}NaO_2]$ $[M+Na]^+$: 309.1825 found: 309.1816. Enantiomeric excess was determined by HPLC with a Chiralpak ODH+OD3 column (hexanes: 2-propanol = 99: 1, 0.5 mL/min, 254 nm); major enantiomer tr = 37.06 min, minor enantiomer tr = 33.72 min..

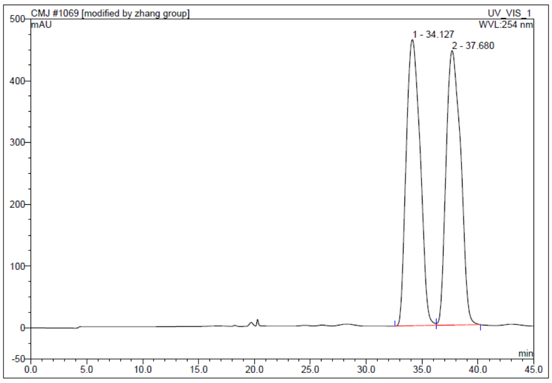

No.	Ret.Time min	Peak Name	Height mAU	Area mAU*min	Rel.Area %	Amount	Type
1	34.13	n.a.	462.964	669.110	50.00	n.a.	BM *
2	37.68	n.a.	443.814	669.042	50.00	n.a.	MB*
Total:			906.778	1338.152	100.00	0.000	

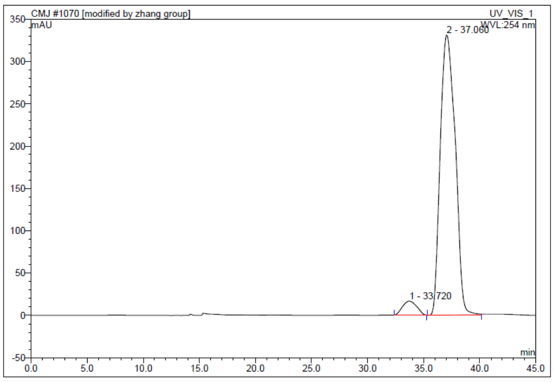

No.	Ret.Time min	Peak Name	Height mAU	Area mAU*min	Rel.Area %	Amount	Type
1	33.72	n.a.	16.415	24.126	4.50	n.a.	BMB*
2	37.06	n.a.	330.940	512.265	95.50	n.a.	BM *
Total:			347.355	536.390	100.00	0.000	

4.1.6 (*S*, *E*)-2-(3-(3,4-dimethoxyphenyl)-allyl)-1-oxaspiro[4.5]decane (3f)

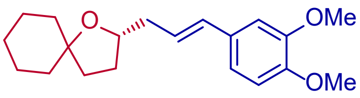

3f

Prepared according to typical procedure at RT for 72 hours by using [Pd(allyl)Cl]$_2$ (2.5 mol%), **Xu8** (10 mol%) from γ-hydroxyalkenes **1b** (0.2 mmol) and alkenyl bromide (0.4 mmol), after flash column chromatography on a silica gel (petroleum ether: ethyl acetate = 20: 1) give the product **3f** as a white liquid (40 mg, 66% yield) with 92% *ee*. $[\alpha]_D^{20}$ = -3.2 (*c* = 0.4, Chloroform).^{1}H NMR (500 MHz, Chloroform-*d*) δ 6.91 (d, *J* = 2.0 Hz, 1H), 6.87 (dd, *J* = 8.0, 2.0 Hz, 1H), 6.79 (d, *J* = 8.5 Hz, 1H), 6.36 (d, *J* = 15.5 Hz, 1H), 6.12 – 6.06 (m, 1H), 4.09 – 4.03 (m, 1H), 3.89 (s, 3H), 3.86 (s, 3H), 2.52 – 2.47 (m, 1H), 2.38 – 2.32 (m, 1H), 2.02 – 1.94 (m, 1H), 1.77 – 1.70 (m, 2H), 1.69 – 1.62 (m, 3H), 1.59 – 1.46 (m, 4H), 1.44 – 1.29 (m, 4H). ^{13}C NMR (125 MHz, Chloroform-*d*) δ 148.9, 148.3, 131.4, 130.9, 125.0, 118.9, 111.1, 108.5, 82.8, 77.5, 55.9, 55.7, 39.8, 38.5, 37.6, 35.7, 30.7, 25.7, 24.1, 23.8. HRMS (ESI) calculated for [$C_{20}H_{28}NaO_3$] [M+Na]$^+$: 339.1931 found: 339.1934. Enantiomeric excess was determined by HPLC with a Chiralpak OJH column (hexanes: 2-propanol = 97: 3, 0.8 mL/min, 254 nm); major enantiomer tr = 16.77 min, minor enantiomer tr = 20.39 min.

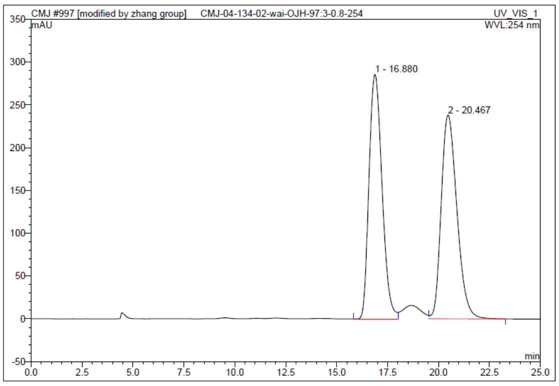

No.	Ret.Time min	Peak Name	Height mAU	Area mAU*min	Rel.Area %	Amount	Type
1	16.88	n.a.	285.677	211.606	49.83	n.a.	BM *
2	20.47	n.a.	237.953	213.029	50.17	n.a.	MB*
Total:			523.629	424.635	100.00	0.000	

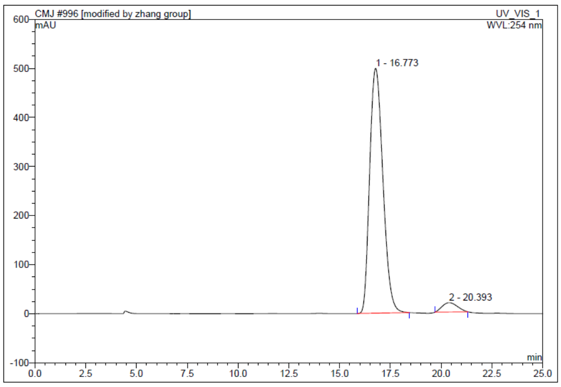

No.	Ret.Time min	Peak Name	Height mAU	Area mAU*min	Rel.Area %	Amount	Type
1	16.77	n.a.	499.175	376.740	95.85	n.a.	BMB*
2	20.39	n.a.	18.929	16.332	4.15	n.a.	BMB*
Total:			518.104	393.071	100.00	0.000	

4.1.7 (*S*, *E*)-2-(3-(3,4,5-trimethoxyphenyl)-allyl)-1-oxaspiro[4.5]decane (3g)

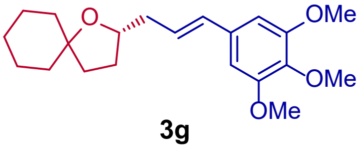

3g

Prepared according to typical procedure at RT for 72 hours by using [Pd(allyl)Cl]$_2$ (2.5 mol%), **Xu8** (10 mol%) from γ-hydroxyalkenes **1b** (0.2 mmol) and alkenyl bromide (0.4 mmol), after flash column chromatography on a silica gel (petroleum ether: ethyl acetate = 10: 1) give the product **3g** as a white liquid (28 mg, 42% yield) with 84% *ee*. $[\alpha]_D^{20}$ = -3.5 (*c* = 0.4, Chloroform). ^{1}H NMR (500 MHz, Chloroform-*d*) δ 6.57 (s, 2H), 6.35 (d, *J* = 16.0 Hz, 1H), 6.17 – 6.11 (m, 1H), 4.10 – 4.05 (m, 1H), 3.86 (s, 6H), 3.83 (s, 3H), 2.52 – 2.46 (m, 1H), 2.39 – 2.33 (m, 1H), 2.02 – 1.96 (m, 1H), 1.77 – 1.71 (m, 2H), 1.69 – 1.64 (m, 3H), 1.60 – 1.45 (m, 4H), 1.44 – 1.29 (m, 4H). ^{13}C NMR (126 MHz, Chloroform-*d*) δ 153.2, 137.3, 133.5, 131.6, 126.5, 103.0, 82.9, 77.4, 60.9, 56.0, 39.8, 38.5, 37.6, 35.7, 30.7, 25.7, 24.1, 23.8. HRMS (ESI) calculated for [$C_{21}H_{30}NaO_4$] [M+Na]$^+$: 369.2036 found: 369.2031. Enantiomeric excess was determined by HPLC with a Chiralpak ASH+ASH column (hexanes: 2-propanol = 90: 10, 0.3 mL/min, 254 nm); major enantiomer tr = 38.39 min, minor enantiomer tr = 36.41 min.

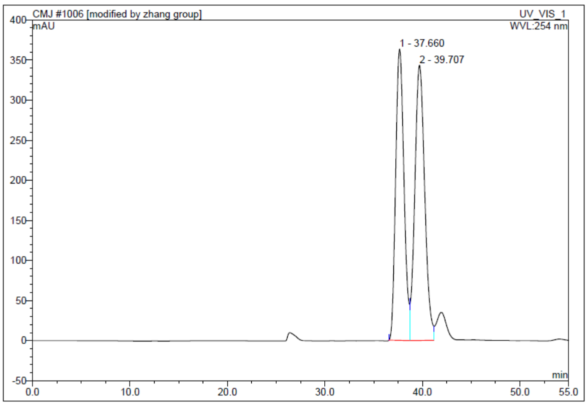

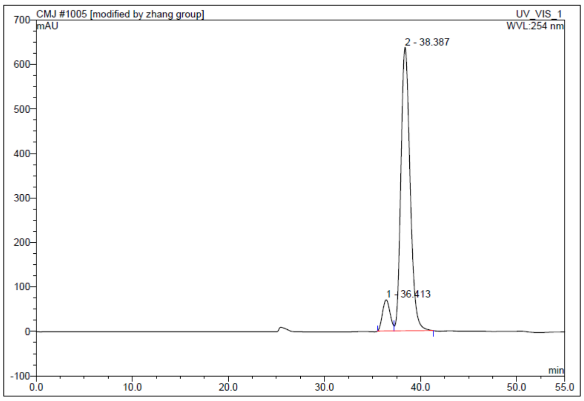

No.	Ret.Time min	Peak Name	Height mAU	Area mAU*min	Rel.Area %	Amount	Type
1	37.66	n.a.	363.371	358.189	46.97	n.a.	BM *
2	39.71	n.a.	343.531	404.409	53.03	n.a.	M *
Total:			706.902	762.598	100.00	0.000	

No.	Ret.Time min	Peak Name	Height mAU	Area mAU*min	Rel.Area %	Amount	Type
1	36.41	n.a.	70.018	63.364	8.26	n.a.	BM *
2	38.39	n.a.	636.766	704.101	91.74	n.a.	MB*
Total:			706.784	767.465	100.00	0.000	

4.1.8 (*S*, *E*)-2-(3-(4-fluorophenyl)-allyl)-1-oxaspiro[4.5]decane (3h)

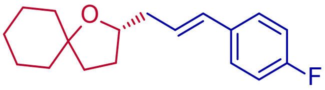

3h

Prepared according to typical procedure at RT for 72 hours by using $[Pd(allyl)Cl]_2$ (2.5 mol%), **Xu8** (10 mol%) from γ-hydroxyalkenes **1b** (0.2 mmol) and alkenyl bromide (0.4 mmol), after flash column chromatography on a silica gel (petroleum ether: ethyl acetate = 80: 1) give the product **3h** as a white liquid (35 mg, 64% yield) with 90% *ee*. $[\alpha]_D^{20}$ = -6.4 (*c* = 0.4, Chloroform). ^{1}H NMR (500 MHz, Chloroform-*d*) δ 7.31 – 7.28 (m, 2H), 6.99 – 6.96 (m, 2H), 6.39 (d, *J* = 16.0 Hz, 1H), 6.18 – 6.12 (m, 1H), 4.10 – 4.04 (m, 1H), 2.52 – 2.46 (m, 1H), 2.40 – 2.33 (m, 1H), 2.01 – 1.95 (m, 1H), 1.77 – 1.61 (m, 5H), 1.58 – 1.52 (m, 4H), 1.46 – 1.30 (m, 4H). ^{13}C NMR (125 MHz, Chloroform-*d*) δ 161.9 (d, *J* = 245.7 Hz), 133.8 (d, *J* = 3.2 Hz), 130.6, 127.4, 127.4, 126.7 (d, *J* = 2.1 Hz), 115.3, 115.2, 82.8, 77.4, 39.8, 38.5, 37.6, 35.7, 30.7, 25.7, 24.1, 23.8. ^{19}F NMR (376 MHz, Chloroform-*d*) δ -115.6. HRMS (ESI) calculated for $[C_{18}H_{23}NaFO]$ $[M+Na]^+$: 297.1625 found: 297.1617. Enantiomeric excess was determined by HPLC with a Chiralpak OJH+OJ3 column (hexanes: 2-propanol = 99: 1, 0.5 mL/min, 254 nm); major enantiomer tr = 23.13 min, minor enantiomer tr = 26.34 min.

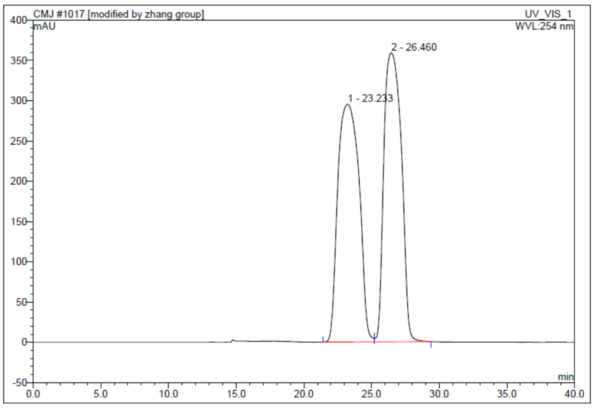

No.	Ret.Time min	Peak Name	Height mAU	Area mAU*min	Rel.Area %	Amount	Type
1	23.23	n.a.	295.112	538.078	50.01	n.a.	BM *
2	26.46	n.a.	358.484	537.810	49.99	n.a.	MB*
Total:			653.596	1075.888	100.00	0.000	

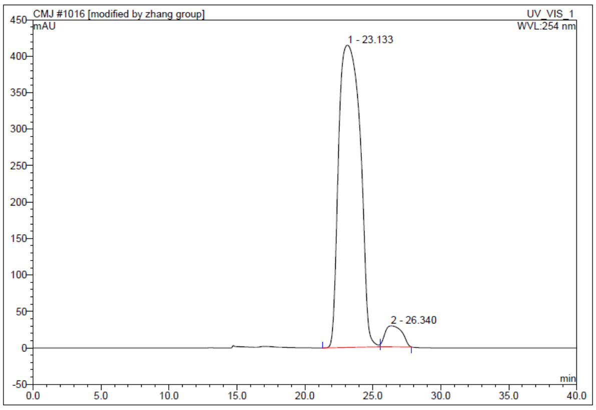

No.	Ret.Time min	Peak Name	Height mAU	Area mAU*min	Rel.Area %	Amount	Type
1	23.13	n.a.	414.757	761.724	94.75	n.a.	BM *
2	26.34	n.a.	28.895	42.212	5.25	n.a.	MB*
Total:			443.652	803.936	100.00	0.000	

4.1.9 (*S*, *E*)-2-(3-(3-fluorophenyl)-allyl)-1-oxaspiro[4.5]decane (3i)

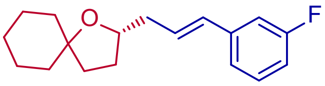

3i

Prepared according to typical procedure at RT for 72 hours by using [Pd(allyl)Cl]$_2$ (2.5 mol%), **Xu8** (10 mol%) from γ-hydroxyalkenes **1b** (0.2 mmol) and alkenyl bromide (0.4 mmol), after flash column chromatography on a silica gel (petroleum ether: ethyl acetate = 80: 1) give the product **3i** as a white liquid (26 mg, 48% yield) with 88% *ee*. $[\alpha]_D^{20}$ = -1.5 (*c* = 0.4, Chloroform). ^{1}H NMR (400 MHz, Chloroform-*d*) δ 7.27 – 7.21 (m, 1H), 7.10 (d, *J* = 7.6 Hz, 1H), 7.06 – 7.03 (m, 1H), 6.91 – 6.86 (m, 1H), 6.40 (d, *J* = 16 Hz, 1H), 6.29 – 6.21 (m, 1H), 4.11 – 3.03 (m, 1H), 2.54 – 2.47 (m, 1H), 2.42 – 2.35 (m, 1H), 2.04 – 1.94 (m, 1H), 1.80 – 1.59 (m, 5H), 1.62 – 1.44 (m, 4H), 1.46 – 1.29 (m, 4H). ^{13}C NMR (100 MHz, Chloroform-*d*) δ 163.1 (d, *J* = 244.7 Hz), 140.1 (d, *J* = 7.7 Hz), 130.8 (d, *J* = 2.6 Hz), 129.8 (d, *J* = 8.5 Hz), 128.5, 121.9 (d, *J* = 2.6 Hz), 113.7 (d, *J* = 21.4 Hz), 112.4 (d, *J* = 21.7 Hz), 82.9, 77.2, 39.8, 38.5, 37.6, 35.8, 30.7, 25.7, 24.1, 23.8. ^{19}F NMR (376 MHz, Chloroform-*d*) δ -113.9. HRMS (ESI) calculated for [$C_{18}H_{23}NaFO$] [M+Na]$^+$: 297.1625 found: 297.1615. Enantiomeric excess was determined by HPLC with a Chiralpak OJH+OJH+OJ3 column (hexanes: 2-propanol = 99: 1, 0.6 mL/min, 254 nm); major enantiomer tr = 8.841 min, minor enantiomer tr = 9.368 min.

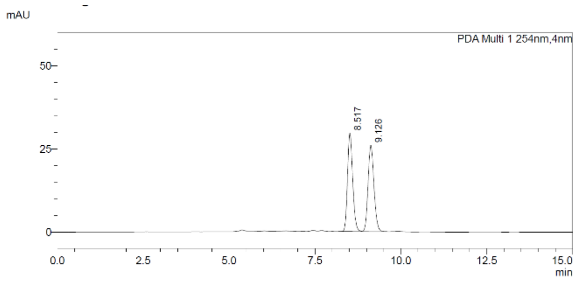

<Peak Table>

PDA Ch1 254nm

Peak#	Ret. Time	Height	Height%	Area	Area%
1	8.517	29605	53.258	312115	50.098
2	9.126	25984	46.742	310895	49.902
Total		55589	100.000	623010	100.000

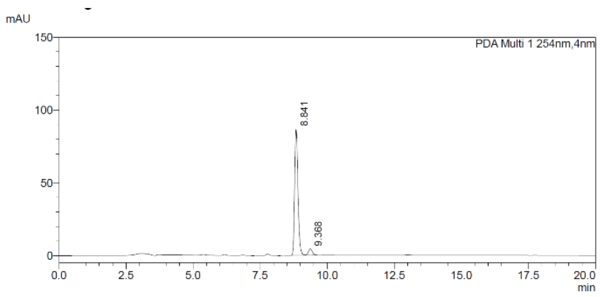

<Peak Table>

PDA Ch1 254nm

Peak#	Ret. Time	Height	Height%	Area	Area%
1	8.841	86923	95.144	747094	94.178
2	9.368	4436	4.856	46185	5.822
Total		91359	100.000	793279	100.000

4.1.10 (*S*, *E*)-2-(3-(thiophen-2-yl)-allyl)-1-oxaspiro[4.5]decane (3j)

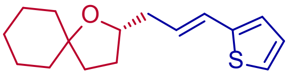

3j

Prepared according to typical procedure at RT for 72 hours by using $[Pd(allyl)Cl]_2$ (2.5 mol%), **Xu8** (10 mol%) from γ-hydroxyalkenes **1b** (0.2 mmol) and alkenyl bromide (0.4 mmol), after flash column chromatography on a silica gel (petroleum ether: ethyl acetate = 80: 1) give the product **3j** as a white liquid (21 mg, 41% yield) with 90% *ee*. $[\alpha]_D^{20}$ = -1.3 (*c* = 0.4, Chloroform). ^{1}H NMR (400 MHz, Chloroform-*d*) δ 7.09 (d, *J* = 5.2 Hz, 1H), 6.93 (dd, *J* = 4.8, 3.2 Hz, 1H), 6.87 (d, *J* = 3.6 Hz, 1H), 6.56 (d, *J* = 15.6 Hz, 1H), 6.10 – 6.03 (m, 1H), 4.10 – 4.03 (m, 1H), 2.51 – 2.44 (m, 1H), 2.38 – 2.30 (m, 1H), 2.02 – 1.94 (m, 1H), 1.78 – 1.60 (m, 5H), 1.61 – 1.43 (m, 4H), 1.46 – 1.28 (m, 4H). ^{13}C NMR (100 MHz, Chloroform-*d*) δ 142.9, 127.1, 126.9, 125.0, 124.4, 123.2, 82.8, 77.3, 39.7, 38.5, 37.6, 35.8, 30.7, 25.7, 24.1, 23.8. HRMS (ESI) calculated for $[C_{16}H_{22}NaSO]$ $[M+Na]^+$: 285.1284 found: 285.1279. Enantiomeric excess was determined by HPLC with a Chiralpak OJH column (hexanes: 2-propanol = 99: 1, 0.5 mL/min, 254 nm); major enantiomer tr = 12.91 min, minor enantiomer tr = 15.65 min.

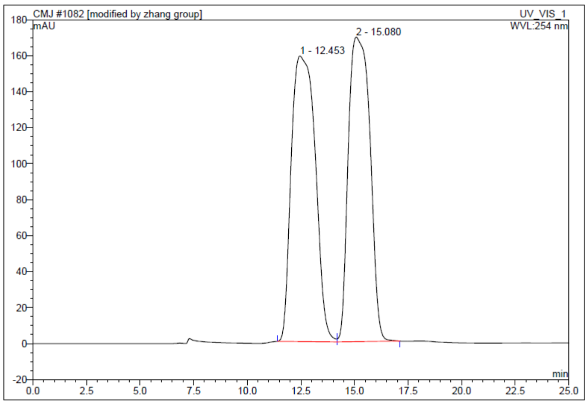

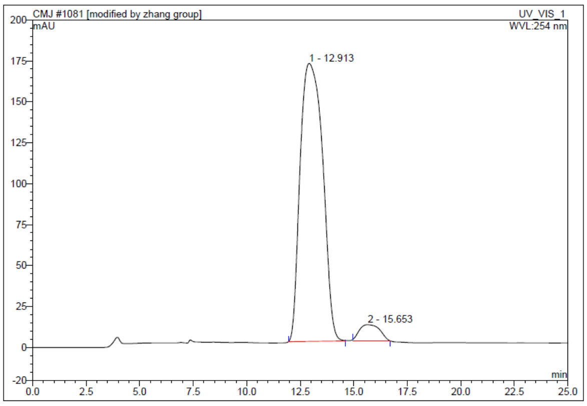

No.	Ret.Time min	Peak Name	Height mAU	Area mAU*min	Rel.Area %	Amount	Type
1	12.45	n.a.	158.759	205.988	50.96	n.a.	BM *
2	15.08	n.a.	169.302	198.194	49.04	n.a.	MB*
Total:			328.061	404.181	100.00	0.000	

No.	Ret.Time min	Peak Name	Height mAU	Area mAU*min	Rel.Area %	Amount	Type
1	12.91	n.a.	169.709	207.284	94.98	n.a.	BM *
2	15.65	n.a.	9.869	10.965	5.02	n.a.	MB*
Total:			179.578	218.249	100.00	0.000	

4.1.11 (*S*, *E*)-2-(3-(naphthalen-1-yl)-allyl)-1-oxaspiro[4.5]decane (3k)

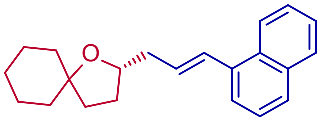

3k

Prepared according to typical procedure at RT for 72 hours by using $[Pd(allyl)Cl]_2$ (2.5 mol%), **Xu8** (10 mol%) from γ-hydroxyalkenes **1b** (0.2 mmol) and alkenyl bromide (0.4 mmol), after flash column chromatography on a silica gel (petroleum ether: ethyl acetate = 80: 1) give the product **3k** as a white liquid (40 mg, 65% yield) with 93% *ee*. $[\alpha]_D^{20}$ = -8.3 (*c* = 0.4, Chloroform).^{1}H NMR (500 MHz, Chloroform-*d*) δ 8.15 (d, *J* = 8.0 Hz, 1H), 7.85 (d, *J* = 7.5 Hz, 1H), 7.76 (d, *J* = 8.5 Hz, 1H), 7.58 (d, *J* = 7.0 Hz, 1H), 7.54 – 7.47 (m, 2H), 7.45 (t, *J* = 7.5 Hz, 1H), 7.20 (d, *J* = 15.5 Hz, 1H), 6.30 – 6.24 (m, 1H), 4.19 – 4.14 (m, 1H), 2.70 – 2.64 (m, 1H), 2.55 – 2.49 (m, 1H), 2.08 – 2.01 (m, 1H), 1.82 – 1.68 (m, 5H), 1.66 – 1.50 (m, 4H), 1.49 – 1.34 (m, 4H). ^{13}C NMR (125 MHz, Chloroform-*d*) δ 135.5, 133.6, 131.1, 130.2, 129.0, 128.4, 127.3, 125.8, 125.6, 125.6, 123.9, 123.6, 82.8, 77.4, 40.2, 38.5, 37.6, 35.8, 30.7, 25.7, 24.1, 23.8. HRMS (ESI) calculated for $[C_{22}H_{26}NaO]$ $[M+Na]^+$: 329.1876 found: 329.1875. Enantiomeric excess was determined by HPLC with a Chiralpak OJH column (hexanes: 2-propanol = 99: 1, 0.5 mL/min, 210 nm); major enantiomer tr = 19.69 min, minor enantiomer tr = 23.88 min.

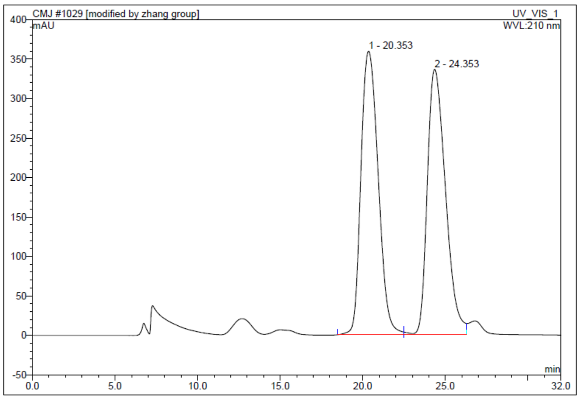

No.	Ret.Time min	Peak Name	Height mAU	Area mAU*min	Rel.Area %	Amount	Type
1	20.35	n.a.	359.090	434.804	50.33	n.a.	BM *
2	24.35	n.a.	336.199	429.149	49.67	n.a.	M *
Total:			695.289	863.954	100.00	0.000	

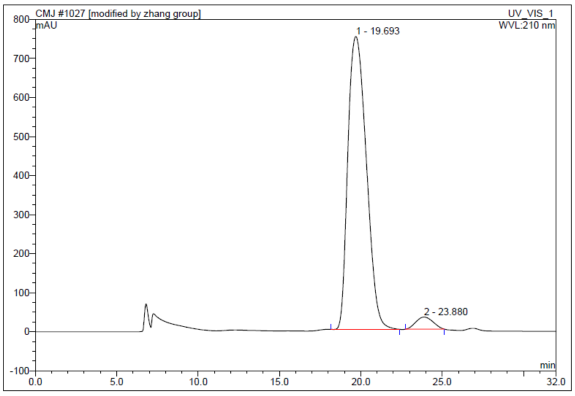

No.	Ret.Time min	Peak Name	Height mAU	Area mAU*min	Rel.Area %	Amount	Type
1	19.69	n.a.	750.029	987.366	96.30	n.a.	BMB*
2	23.88	n.a.	31.042	37.950	3.70	n.a.	BMB*
Total:			781.071	1025.316	100.00	0.000	

4.1.12 (*S*, *E*)-2-(3-(naphthalen-2-yl)-allyl)-1-oxaspiro[4.5]decane (3l)

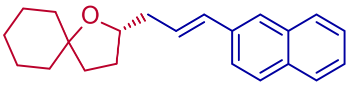

3l

Prepared according to typical procedure at RT for 72 hours by using $[Pd(allyl)Cl]_2$ (2.5 mol%), **Xu8** (10 mol%) from γ-hydroxyalkenes **1b** (0.2 mmol) and alkenyl bromide (0.4 mmol), after flash column chromatography on a silica gel (petroleum ether: ethyl acetate = 80: 1) give the product **3l** as a white liquid (34 mg, 56% yield) with 91% *ee*. $[\alpha]_D^{20}$ = -5.4 (*c* = 0.4, Chloroform). ^{1}H NMR (500 MHz, Chloroform-*d*) δ 7.80 – 7.63 (m, 3H), 7.69 (s, 1H), 7.60 (dd, *J* = 8.5, 1.5 Hz, 1H), 7.47 – 7.40 (m, 2H), 6.61 (d, *J* = 16.0 Hz, 1H), 6.41 – 6.35 (m, 1H), 4.15 – 4.10 (m, 1H), 2.62 – 2.56 (m, 1H), 2.48 – 2.42 (m, 1H), 2.06 – 1.98 (m, 1H), 1.82 – 1.65 (m, 5H), 1.65 – 1.49 (m, 4H), 1.49 – 1.31 (m, 4H). ^{13}C NMR (125 MHz, Chloroform-*d*) δ 135.1, 133.6, 132.7, 131.9, 128.0, 127.8, 127.6, 127.4, 126.1, 125.5, 125.5, 123.5, 82.8, 77.4, 40.0, 38.5, 37.6, 35.7, 30.8, 25.7, 24.2, 23.8. HRMS (ESI) calculated for $[C_{22}H_{26}NaO]$ $[M+Na]^+$: 329.1876 found: 329.1869. Enantiomeric excess was determined by HPLC with a Chiralpak OJH column (hexanes: 2-propanol = 99: 1, 0.5 mL/min, 210 nm); major enantiomer tr = 33.72 min, minor enantiomer tr = 39.15 min.

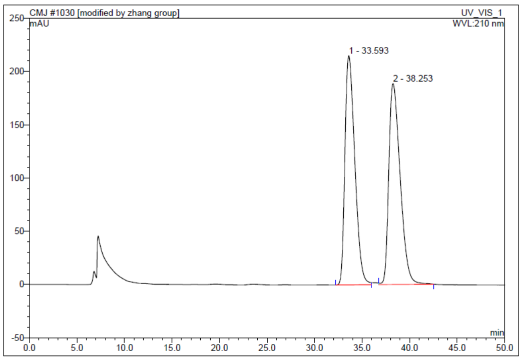

No.	Ret.Time min	Peak Name	Height mAU	Area mAU*min	Rel.Area %	Amount	Type
1	33.59	n.a.	215.210	258.937	49.82	n.a.	BM *
2	38.25	n.a.	188.558	260.833	50.18	n.a.	MB*
Total:			403.768	519.770	100.00	0.000	

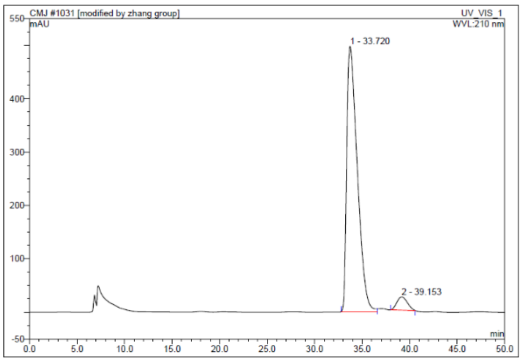

No.	Ret.Time min	Peak Name	Height mAU	Area mAU*min	Rel.Area %	Amount	Type
1	33.72	n.a.	496.638	640.731	95.31	n.a.	BM *
2	39.15	n.a.	24.851	31.534	4.69	n.a.	BMB*
Total:			521.489	672.264	100.00	0.000	

4.1.13 (*S*)-2-((2*E*,4*E*)-5-phenylpenta-2,4-dien-1-yl)-1-oxaspiro[4.5]decane (3m)

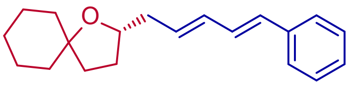

3m

Prepared according to typical procedure at RT for 72 hours by using $[Pd(allyl)Cl]_2$ (2.5 mol%), **Xu8** (10 mol%) from γ-hydroxyalkenes **1b** (0.2 mmol) and alkenyl bromide (0.4 mmol), after flash column chromatography on a silica gel (petroleum ether: ethyl acetate = 80: 1) give the product **3m** as a white liquid (47 mg, 83% yield) with 92% *ee*. $[\alpha]_D^{20}$ = 10.4 (*c* = 0.4, Chloroform). ^{1}H NMR (500 MHz, Chloroform-*d*) δ 7.38 (d, *J* = 7.3 Hz, 1 H), 7.33 – 7.27 (m, 2 H), 7.20 (t, *J* = 7.3 Hz, 1 H), 6.77 (dd, *J* = 15.6, 10.4 Hz, 1 H), 6.46 (d, *J* = 15.6 Hz, 1 H), 6.26 (dd, *J* = 15.2, 10.4 Hz, 1 H), 5.90 – 5.77 (m, 1 H), 4.10 – 3.98 (m, 1 H), 2.54 – 2.41 (m, 1 H), 2.38 – 2.23 (m, 1 H), 2.07 – 1.90 (m, 1 H), 1.77 – 1.65 (m, 4 H), 1.64 – 1.47 (m, 5 H), 1.48 – 1.31 (m, 4 H). ^{13}C NMR (125 MHz, Chloroform-*d*) δ 137.5, 132.4, 131.5, 130.4, 129.2, 128.5, 127.1, 126.1, 82.7, 77.3, 39.8, 38.5, 37.5, 35.6, 30.7, 25.6, 24.1, 23.8. HRMS (ESI) calculated for $[C_{20}H_{26}NaO]$ $[M+Na]^+$: 305.1876 found: 305.1878. Enantiomeric excess was determined by HPLC with a Chiralpak OJH column (hexanes: 2-propanol = 99: 1, 0.5 mL/min, 254 nm); major enantiomer tr = 20.67 min, minor enantiomer tr = 24.06 min.

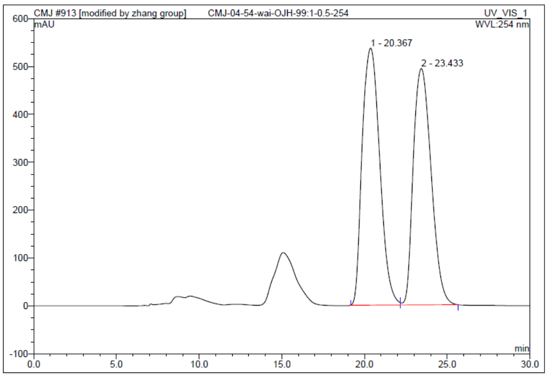

No.	Ret.Time min	Peak Name	Height mAU	Area mAU*min	Rel.Area %	Amount	Type
1	20.37	n.a.	536.876	661.609	51.68	n.a.	BM *
2	23.43	n.a.	493.854	618.488	48.32	n.a.	MB*
Total:			1030.730	1280.097	100.00	0.000	

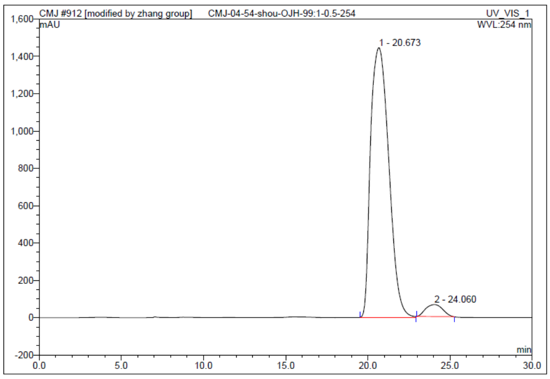

No.	Ret.Time min	Peak Name	Height mAU	Area mAU*min	Rel.Area %	Amount	Type
1	20.67	n.a.	1444.133	1854.477	96.00	n.a.	BM *
2	24.06	n.a.	63.956	77.235	4.00	n.a.	BMB*
Total:			1508.089	1931.712	100.00	0.000	

4.1.14 (*S*)-2-(cyclohex-1-en-1-ylmethyl)-1-oxaspiro[4.5]decane (3n)

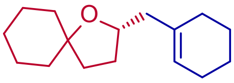

3n

Prepared according to typical procedure at RT for 96 hours by using $[Pd(allyl)Cl]_2$ (5 mol%), **Xu8** (20 mol%) from γ-hydroxyalkenes **1b** (0.2 mmol) and alkenyl bromide (0.4 mmol), after flash column chromatography on a silica gel (petroleum ether: ethyl acetate = 80: 1) give the product **3n** as a white liquid (28 mg, 60% yield) with 90% *ee*. $[\alpha]_D^{20}$ = -5.8 (*c* = 0.4, Chloroform). ^{1}H NMR (500 MHz, Chloroform-*d*) δ 5.42 (t, *J* = 3.5 Hz, 1 H), 4.10 – 3.91 (m, 1 H), 2.30 (dd, *J* = 13.4, 5.5 Hz, 1 H), 2.03 – 1.87 (m, 6 H), 1.74 – 1.61 (m, 4 H), 1.61 – 1.43 (m, 9 H), 1.42 – 1.28 (m, 4 H). ^{13}C NMR (125 MHz, Chloroform-*d*) δ 135.2, 122.7, 82.4, 76.7, 45.1, 38.6, 37.6, 35.6, 31.1, 29.0, 25.7, 25.2, 24.1, 23.8, 22.9, 22.4. HRMS (ESI) calculated for $[C_{16}H_{26}NaO]$ $[M+Na]^+$: 257.1876 found: 257.1878. Enantiomeric excess was determined by HPLC with a Chiralpak OJH+OJH+OJ3 column (hexanes: 2-propanol = 99: 1, 0.5 mL/min, 210 nm); major enantiomer tr = 23.13 min, minor enantiomer tr = 22.36 min.

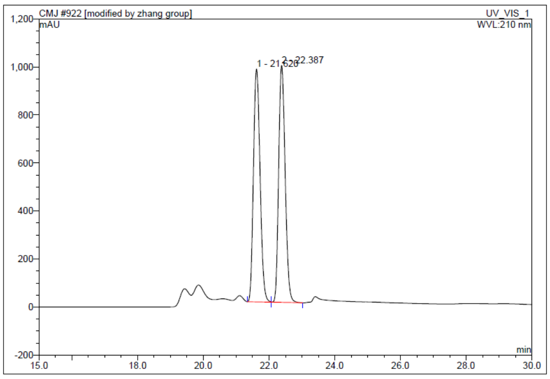

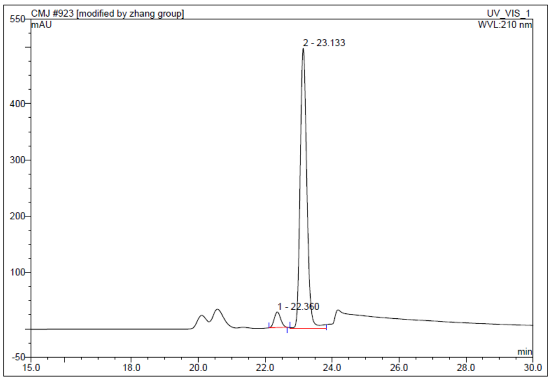

No.	Ret.Time min	Peak Name	Height mAU	Area mAU*min	Rel.Area %	Amount	Type
1	21.62	n.a.	970.096	226.119	50.00	n.a.	BM *
2	22.39	n.a.	987.383	226.124	50.00	n.a.	MB*
Total:			1957.479	452.243	100.00	0.000	

No.	Ret.Time min	Peak Name	Height mAU	Area mAU*min	Rel.Area %	Amount	Type
1	22.36	n.a.	27.638	6.157	5.09	n.a.	BMB*
2	23.13	n.a.	496.817	114.717	94.91	n.a.	M *
Total:			524.455	120.874	100.00	0.000	

4.1.15 Synthesis of (*S*, *E*)-2-(3-phenylbut-2-en-1-yl)-1-oxaspiro[4.5]decane (3o)

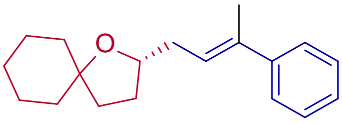

3o

Prepared according to typical procedure at RT for 72 hours by using $[Pd(allyl)Cl]_2$ (2.5 mol%), **Xu8** (10 mol%) from γ-hydroxyalkenes (0.2 mmol) and alkenyl bromide (0.4 mmol), after flash column chromatography on a silica gel (petroleum ether: ethyl acetate = 50: 1) give the product **3o** as a white liquid (34 mg, 63% yield) with 87% *ee*. $[\alpha]_D^{20}$ = -2.5 (*c* = 0.4, Chloroform).^{1}H NMR (400 MHz, Chloroform-*d*) δ 7.41 – 7.34 (m, 2H), 7.33 – 7.27 (m, 2H), 7.24 – 7.18 (m, 1H), 5.80 (tq, *J* = 7.3, 1.4 Hz, 1H), 4.07 (tt, *J* = 7.5, 5.4 Hz, 1H), 2.56 (dddd, *J* = 14.5, 7.2, 5.2, 1.0 Hz, 1H), 2.35 (dtd, *J* = 14.6, 7.4, 1.0 Hz, 1H), 2.08 – 2.01 (m, 3H), 2.02 – 1.93 (m, 1H), 1.79 – 1.61 (m, 5H), 1.53 (tq, *J* = 6.3, 3.7 Hz, 4H), 1.45 – 1.28 (m, 4H). ^{13}C NMR (100 MHz, Chloroform-*d*) δ 143.9, 136.3, 128.1, 126.6, 125.7, 124.3, 82.7, 77.6, 38.6, 37.7, 35.9, 35.7, 30.9, 25.7, 24.2, 23.9, 16.1. HRMS (ESI) calculated for $[C_{19}H_{26}NaO]$ $[M+Na]^+$: 293.1876 found: 293.1868. Enantiomeric excess was determined by HPLC with a Chiralpak OJH-OJH column (hexanes: 2-propanol = 99: 1, 0.6 mL/min, 254 nm); major enantiomer tr = 16.400 min, minor enantiomer tr = 16.989.

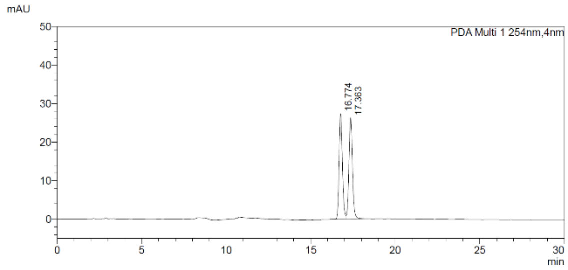

<Peak Table>
PDA Ch1 254nm

Peak#	Ret. Time	Height	Height%	Area	Area%
1	16.774	27340	51.036	370033	49.417
2	17.363	26230	48.964	378770	50.583
Total		53570	100.000	748803	100.000

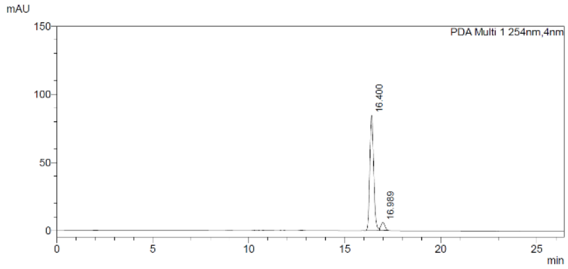

<Peak Table>
PDA Ch1 254nm

Peak#	Ret. Time	Height	Height%	Area	Area%
1	16.400	84703	93.596	1185985	93.439
2	16.989	5796	6.404	83276	6.561
Total		90499	100.000	1269261	100.000

4.1.16 (*S*, *E*)-10-(3-(4-methoxyphenyl)-allyl)-1,4,9-trioxadispiro[4.2.48.25]tetradecane (4a)

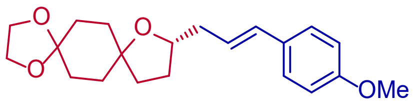

4a

Prepared according to typical procedure at RT for 72 hours by using $[Pd(allyl)Cl]_2$ (2.5 mol%), **Xu8** (10 mol%) from γ-hydroxyalkenes (0.2 mmol) and alkenyl bromide (0.4 mmol), after flash column chromatography on a silica gel (petroleum ether: ethyl acetate = 10: 1) give the product **4a** as a white liquid (43 mg, 63% yield) with 88% *ee*. $[\alpha]_D^{20}$ = -9.6 (*c* = 0.4, Chloroform). ^{1}H NMR (400 MHz, Chloroform-*d*) δ 7.29 – 7.25 (m, 2H), 6.85 – 6.81 (m, 2H), 6.37 (d, *J* = 15.6 Hz, 1H), 6.12 – 6.04 (m, 1H), 4.08 – 4.01 (m, 1H), 3.97 – 3.90 (m, 4H), 3.79 (s, 3H), 2.50 – 2.43 (m, 1H), 2.38 – 2.31 (m, 1H), 2.01 – 1.96 (m, 1H), 1.93 – 1.84 (m, 2H), 1.80 – 1.70 (m, 4H), 1.70 – 1.64 (m, 2H), 1.61 – 1.52 (m, 3H). ^{13}C NMR (100 MHz, Chloroform-*d*) δ 158.7, 131.2, 130.5, 127.0, 124.6, 113.9, 108.7, 81.0, 77.9, 64.2, 64.1, 55.2, 39.8, 36.2, 35.5, 34.3, 31.8, 31.8, 30.5. HRMS (ESI) calculated for $[C_{21}H_{28}NaO_4]$ $[M+Na]^+$: 367.1880 found: 367.1873. Enantiomeric excess was determined by HPLC with a Chiralpak ADH column (hexanes: 2-propanol = 60: 40, 0.5 mL/min, 254 nm); major enantiomer tr = 11.45 min, minor enantiomer tr = 10.69 min.

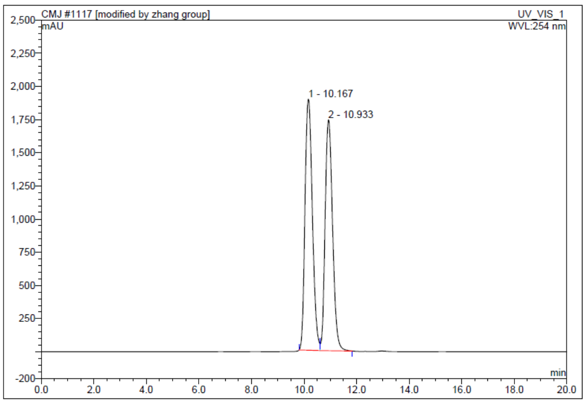

No.	Ret.Time min	Peak Name	Height mAU	Area mAU*min	Rel.Area %	Amount	Type
1	10.17	n.a.	1892.219	615.732	51.87	n.a.	BM *
2	10.93	n.a.	1739.297	571.296	48.13	n.a.	MB*
Total:			3631.517	1187.028	100.00	0.000	

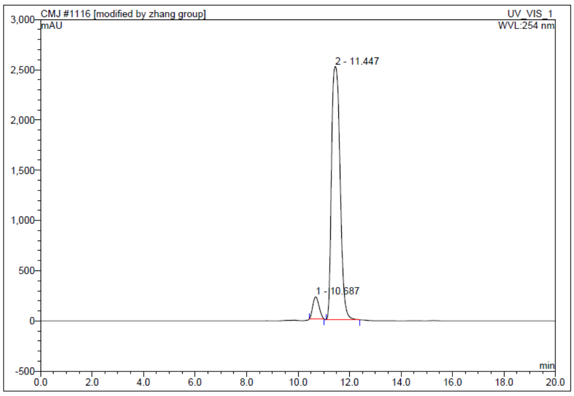

No.	Ret.Time min	Peak Name	Height mAU	Area mAU*min	Rel.Area %	Amount	Type
1	10.69	n.a.	222.057	62.811	6.09	n.a.	BMB*
2	11.45	n.a.	2521.153	968.983	93.91	n.a.	BMB*
Total:			2743.210	1031.794	100.00	0.000	

4.1.17 (*S*, *E*)-8,8-difluoro-2-(3-(4-methoxyphenyl)-allyl)-1-oxaspiro[4.5]decane (4b)

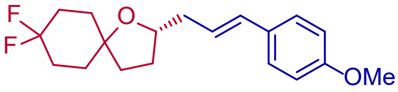

4b

Prepared according to typical procedure at RT for 72 hours by using $[Pd(allyl)Cl]_2$ (2.5 mol%), **Xu8** (10 mol%) from γ-hydroxyalkenes (0.2 mmol) and alkenyl bromide (0.4 mmol), after flash column chromatography on a silica gel (petroleum ether: ethyl acetate = 80: 1) give the product **4b** as a white liquid (38 mg, 60% yield) with 87% *ee*. $[\alpha]_D^{20}$ = -5.4 (*c* = 0.4, Chloroform). ^{1}H NMR (500 MHz, Chloroform-*d*) δ 7.29 (d, *J* = 9.0 Hz, 2H), 6.85 (d, *J* = 9.0 Hz, 2H), 6.39 (d, *J* = 15.5 Hz, 1H), 6.10 – 6.04 (m, 1H), 4.09 – 4.04 (m, 1H), 3.80 (s, 3H), 2.50 – 2.45 (m, 1H), 2.39 – 2.33 (m, 1H), 2.23 – 2.05 (m, 2H), 2.04 – 1.99 (m, 1H), 1.96 – 1.86 (m, 2H), 1.82 – 1.59 (m, 7H). ^{13}C NMR (125 MHz, Chloroform-*d*) δ 158.8, 131.4, 130.4, 127.1, 124.3, 123.6 (t, *J* = 240.1 Hz), 113.9, 80.0, 78.2, 55.2, 39.6, 36.4 (d, *J* = 2.1 Hz), 34.3 (dd, *J* = 7.8, 2.0 Hz), 33.0 (dd, *J* = 8.0, 2.0 Hz), 30.9 (d, *J* = 24.5 Hz), 30.7 (d, *J* = 24.1 Hz), 30.5. ^{19}F NMR (376 MHz, Chloroform-*d*) δ -93.8 (d, *J* = 235.0 Hz), -102.5 (d, *J* = 234.9 Hz). HRMS (ESI) calculated for $[C_{19}H_{24}NaF_2O_2]$ $[M+Na]^+$: 345.1637 found: 345.1627. Enantiomeric excess was determined by HPLC with a Chiralpak OJH+OJ3 column (hexanes: 2-propanol = 90: 10, 0.5 mL/min, 254 nm); major enantiomer tr = 50.19 min, minor enantiomer tr = 45.05 min.

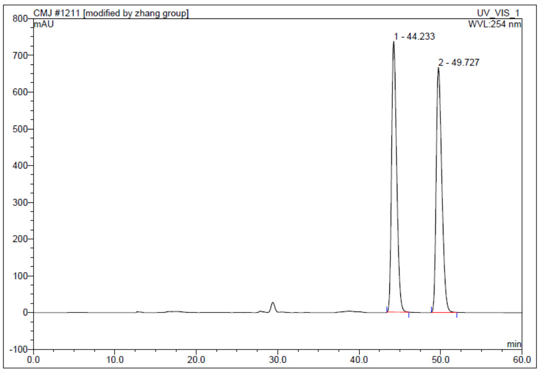

No.	Ret.Time min	Peak Name	Height mAU	Area mAU*min	Rel.Area %	Amount	Type
1	44.23	n.a.	736.491	531.933	49.87	n.a.	BMB*
2	49.73	n.a.	666.416	534.668	50.13	n.a.	BMB*
Total:			1402.907	1066.601	100.00	0.000	

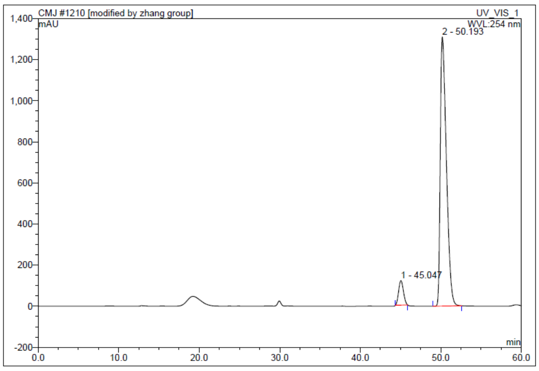

No.	Ret.Time min	Peak Name	Height mAU	Area mAU*min	Rel.Area %	Amount	Type
1	45.05	n.a.	119.288	82.333	6.68	n.a.	BMB*
2	50.19	n.a.	1309.592	1150.666	93.32	n.a.	BMB*
Total:			1428.880	1232.999	100.00	0.000	

4.1.18 (*S*, *E*)-2-(3-(4-methoxyphenyl)-allyl)-1,8-dioxaspiro[4.5]decane (4c)

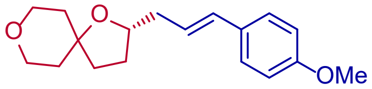

4c

Prepared according to typical procedure at RT for 72 hours by using $[Pd(allyl)Cl]_2$ (2.5 mol%), **Xu8** (10 mol%) from γ-hydroxyalkenes (0.2 mmol), NaO^tBu (0.4 mmol), alkenyl bromide (0.4 mmol) and no additive H_2O, after flash column chromatography on a silica gel (petroleum ether: ethyl acetate = 10: 1) give the product **4c** as a white liquid (42 mg, 75% yield) with 85% *ee*. $[\alpha]_D^{20}$ = -7.8 (*c* = 0.4, Chloroform). ^1H NMR (400 MHz, Chloroform-*d*) δ 7.27 (d, *J* = 8.8 Hz, 2H), 6.84 (d, *J* = 8.8 Hz, 2H), 6.38 (d, *J* = 16.0 Hz, 1H), 6.11 – 6.03 (m, 1H), 4.11 – 4.04 (m, 1H), 3.87 – 3.81 (m, 2H), 3.79 (s, 3H), 3.66 – 3.59 (m, 2H), 2.53 – 2.46 (m, 1H), 2.39 – 2.32 (m, 1H), 2.06 – 1.98 (m, 1H), 1.85 – 1.76 (m, 1H), 1.75 – 1.69 (m, 2H), 1.68 – 1.59 (m, 4H). ^{13}C NMR (100 MHz, Chloroform-*d*) δ 158.8, 131.4, 130.4, 127.1, 124.3, 113.9, 79.4, 77.9, 65.5, 65.4, 55.2, 39.7, 38.7, 37.6, 36.5, 30.3. HRMS (ESI) calculated for $[C_{18}H_{24}NaO_3]$ $[M+Na]^+$: 311.1618 found: 311.1611. Enantiomeric excess was determined by HPLC with a Chiralpak IC+IC column (hexanes: 2-propanol = 60: 40, 0.5 mL/min, 254 nm); major enantiomer tr = 27.05 min, minor enantiomer tr = 28.22 min.

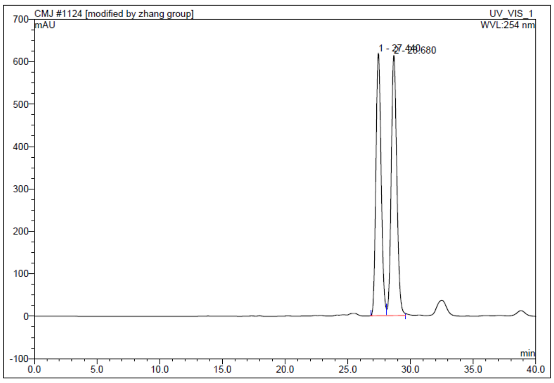

No.	Ret.Time min	Peak Name	Height mAU	Area mAU*min	Rel.Area %	Amount	Type
1	27.44	n.a.	618.687	299.629	48.57	n.a.	BM *
2	28.68	n.a.	613.191	317.211	51.43	n.a.	M *
Total:			1231.878	616.840	100.00	0.000	

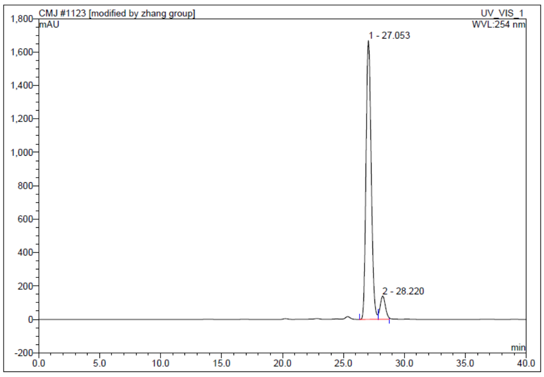

No.	Ret.Time min	Peak Name	Height mAU	Area mAU*min	Rel.Area %	Amount	Type
1	27.05	n.a.	1668.984	824.067	92.25	n.a.	BM *
2	28.22	n.a.	138.669	69.224	7.75	n.a.	M *
Total:			1807.654	893.291	100.00	0.000	

4.1.19 (*S*, *E*)-2-(3-(4-methoxyphenyl)-allyl)-1-oxaspiro[4.6]undecane (4d)

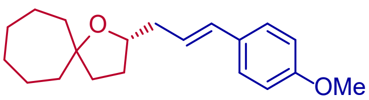

4d

Prepared according to typical procedure at RT for 72 hours by using $[Pd(allyl)Cl]_2$ (2.5 mol%), **Xu8** (10 mol%) from γ-hydroxyalkenes (0.2 mmol) and alkenyl bromide (0.4 mmol), after flash column chromatography on a silica gel (petroleum ether: ethyl acetate = 80: 1) give the product **4d** as a white liquid (32 mg, 54% yield) with 88% *ee*. $[\alpha]_D^{20}$ = -5.5 (*c* = 0.4, Chloroform). ^1H NMR (400

MHz, Chloroform-*d*) δ 7.27 (d, *J* = 8.8 Hz, 2H), 6.83 (d, *J* = 8.8 Hz, 2H), 6.36 (d, *J* = 16 Hz, 1H), 6.11 – 6.03 (m, 1H), 4.03 – 3.97 (m, 1H), 3.79 (s, 3H), 2.53 – 2.46 (m, 1H), 2.37 – 2.29 (m, 1H), 1.99 – 1.92 (m, 1H), 1.82 – 1.50 (m, 13H), 1.49 – 1.35 (m, 2H). ^{13}C NMR (100 MHz, Chloroform-*d*) δ 158.7, 131.1, 130.6, 127.1, 124.7, 113.9, 86.4, 77.7, 55.2, 41.6, 40.6, 39.7, 37.9, 30.5, 29.5, 29.4, 23.2, 22.9. HRMS (ESI) calculated for [$C_{20}H_{28}NaO_2$] [M+Na]$^+$: 323.1982 found: 323.1978. Enantiomeric excess was determined by HPLC with a Chiralpak OJH+OJH column (hexanes: 2-propanol = 99: 1, 0.5 mL/min, 254 nm); major enantiomer tr = 46.11 min, minor enantiomer tr = 48.99 min.

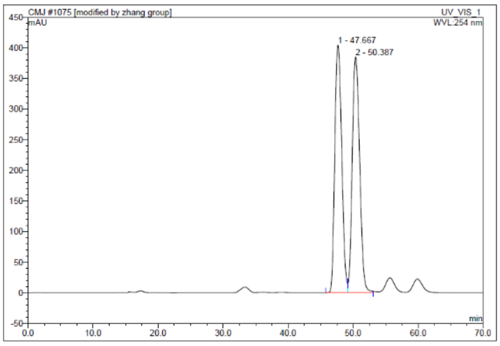

No.	Ret.Time min	Peak Name	Height mAU	Area mAU*min	Rel.Area %	Amount	Type
1	47.67	n.a.	404.595	499.529	49.54	n.a.	BM *
2	50.39	n.a.	385.167	508.828	50.46	n.a.	M *
Total:			789.763	1008.357	100.00	0.000	

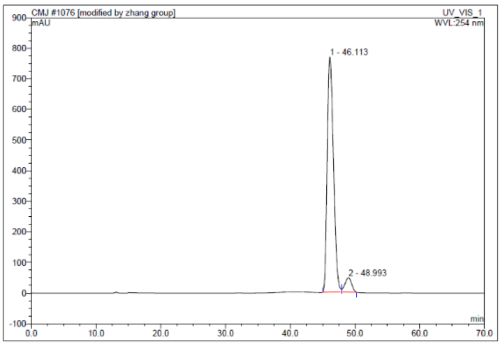

No.	Ret.Time min	Peak Name	Height mAU	Area mAU*min	Rel.Area %	Amount	Type
1	46.11	n.a.	769.025	867.489	93.96	n.a.	BM *
2	48.99	n.a.	46.420	55.798	6.04	n.a.	MB*
Total:			815.445	923.287	100.00	0.000	

4.1.20 (*S*, *E*)-2-(3-(4-methoxyphenyl)-allyl)-1-oxaspiro[4.4]nonane (4e)

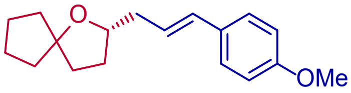

4e

Prepared according to typical procedure at RT for 72 hours by using [Pd(allyl)Cl]$_2$ (2.5 mol%), **Xu8** (10 mol%) from γ-hydroxyalkenes (0.2 mmol) and alkenyl bromide (0.4 mmol), after flash column chromatography on a silica gel (petroleum ether: ethyl acetate = 80: 1) give the product **4e** as a white liquid (43 mg, 79% yield) with 93% *ee*. $[\alpha]_D^{20}$= -11.1 (*c* = 0.4, Chloroform). ^{1}H NMR (400 MHz, Chloroform-*d*) δ 7.27 (d, *J* = 8.8 Hz, 2H), 6.83 (d, *J* = 8.8 Hz, 2H), 6.37 (d, *J* = 16.0 Hz, 1H), 6.10 – 6.03 (m, 1H), 4.06 – 3.99 (m, 1H), 3.78 (s, 3H), 2.52 – 2.46 (m, 1H), 2.37 – 2.30 (m, 1H), 2.03 – 1.95 (m, 1H), 1.89 – 1.73 (m, 6H), 1.56 – 1.64 (m, 1H), 1.63 – 1.45 (m, 4H). ^{13}C NMR (100 MHz, Chloroform-*d*) δ 158.7, 131.2, 130.5, 127.1, 124.6, 113.9, 91.2, 77.9, 55.2, 39.9, 39.1, 38.3, 36.5, 31.1, 24.0. HRMS (ESI) calculated for [$C_{18}H_{24}NaO_2$] [M+Na]$^+$: 295.1669 found: 295.1668.

Enantiomeric excess was determined by HPLC with a Chiralpak OJH column (hexanes: 2-propanol = 99: 1, 0.5 mL/min, 254 nm); major enantiomer tr = 27.22 min, minor enantiomer tr = 30.29 min.

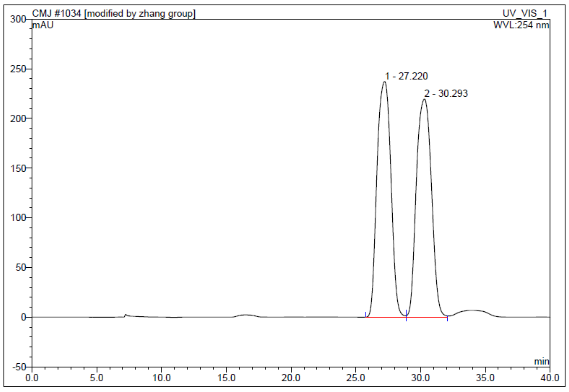

No.	Ret.Time min	Peak Name	Height mAU	Area mAU*min	Rel.Area %	Amount	Type
1	27.22	n.a.	237.244	294.787	49.94	n.a.	BM *
2	30.29	n.a.	219.533	295.518	50.06	n.a.	M *
Total:			456.777	590.305	100.00	0.000	

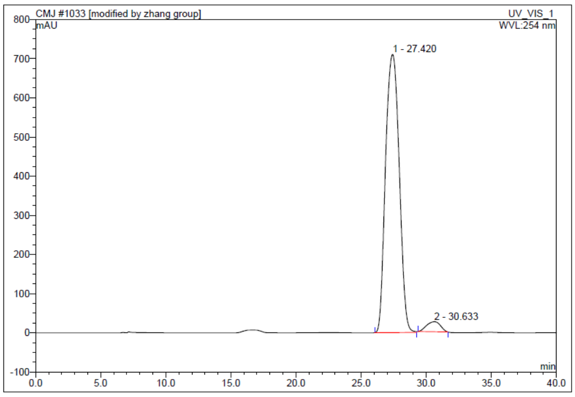

No.	Ret.Time min	Peak Name	Height mAU	Area mAU*min	Rel.Area %	Amount	Type
1	27.42	n.a.	710.108	864.106	96.49	n.a.	BM *
2	30.63	n.a.	25.582	31.478	3.51	n.a.	BMB*
Total:			735.689	895.584	100.00	0.000	

4.1.21 (*S*, *E*)-5-(3-(4-methoxyphenyl)-allyl)-2,2-dimethyltetrahydrofuran (4f)

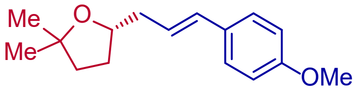

4f

Prepared according to typical procedure at RT for 72 hours by using $[Pd(allyl)Cl]_2$ (2.5 mol%), **Xu8** (10 mol%) from γ-hydroxyalkenes (0.2 mmol), NaO^tBu (0.4 mmol), alkenyl bromide (0.4 mmol) and no additive H_2O, after flash column chromatography on a silica gel (petroleum ether: ethyl acetate = 80: 1) give the product **4f** as a white liquid (43 mg, 87% yield) with 91% *ee*. $[\alpha]_D^{20}$ = -12.7 (*c* = 0.4, Chloroform). ^1H NMR (400 MHz, Chloroform-*d*) δ 7.28 (d, *J* = 8.8 Hz, 2H), 6.83 (d, *J* = 8.8 Hz, 2H), 6.38 (d, *J* = 16.0 Hz, 1H), 6.12 – 6.04 (m, 1H), 4.11 – 4.05 (m, 1H), 3.80 (s, 3H), 2.53 – 2.46 (m, 1H), 2.39 – 2.31 (m, 1H), 2.04 – 1.98 (m, 1H), 1.72 – 1.69 (m, 1H), 1.67 – 1.65 (m, 2H), 1.27 (s, 3H), 1.24 (s, 3H). ^{13}C NMR (100 MHz, Chloroform-*d*) δ 158.8, 131.3, 130.6, 127.1, 124.5, 113.9, 80.7, 78.2, 55.3, 39.9, 38.5, 31.2, 29.2, 28.1. HRMS (ESI) calculated for $[C_{16}H_{22}NaO_2]$ $[M+Na]^+$: 269.1512 found: 269.1507. Enantiomeric excess was determined by HPLC with a Chiralpak OJH+OJ3 column (hexanes: 2-propanol = 99: 1, 0.5 mL/min, 254 nm); major enantiomer tr = 56.59 min, minor enantiomer tr = 60.39 min.

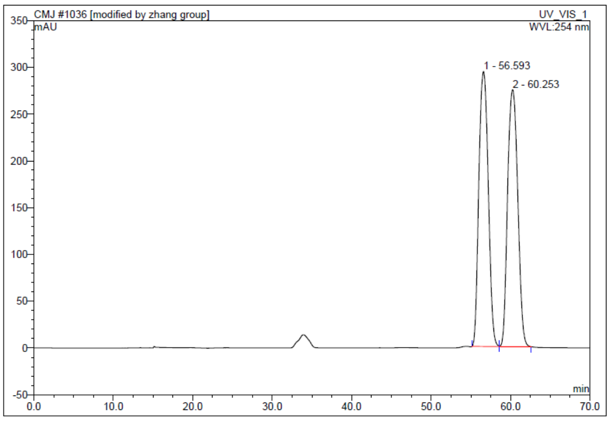

No.	Ret.Time min	Peak Name	Height mAU	Area mAU*min	Rel.Area %	Amount	Type
1	56.59	n.a.	294.033	403.581	49.78	n.a.	BM *
2	60.25	n.a.	274.703	407.222	50.22	n.a.	MB*
Total:			568.736	810.803	100.00	0.000	

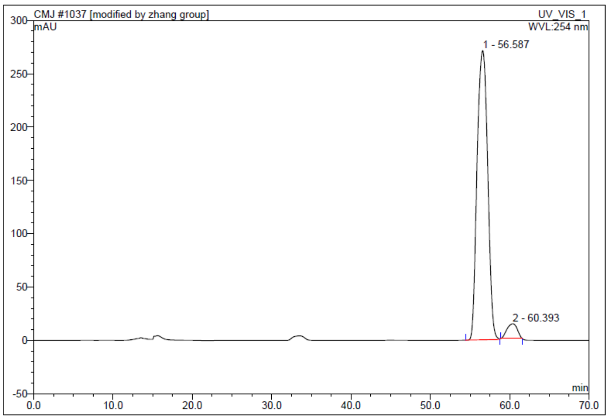

No.	Ret.Time min	Peak Name	Height mAU	Area mAU*min	Rel.Area %	Amount	Type
1	56.59	n.a.	271.243	420.597	95.29	n.a.	BM *
2	60.39	n.a.	13.821	20.796	4.71	n.a.	BMB*
Total:			285.065	441.393	100.00	0.000	

4.1.22 (*S*, *E*)-5-(3-(4-fluorophenyl)-allyl)-2,2-dimethyltetrahydrofuran (4g)

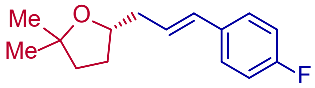

4g

Prepared according to typical procedure at RT for 72 hours by using [Pd(allyl)Cl]$_2$ (2.5 mol%), **Xu8** (10 mol%) from γ-hydroxyalkenes (0.2 mmol), NaO^{t}Bu (0.4 mmol), alkenyl bromide (0.4 mmol) and no additive H_2O, after flash column chromatography on a silica gel (petroleum ether: ethyl acetate = 80: 1) give the product **4g** as a white liquid (20 mg, 43% yield) with 89% *ee*. $[\alpha]_D^{20}$= -8.4 (*c* = 0.4, Chloroform). ^{1}H NMR (400 MHz, Chloroform-*d*) δ 7.32 – 7.28 (m, 2H), 6.99 – 6.95 (m, 2H), 6.39 (d, *J* = 16.0 Hz, 1H), 6.18 – 6.10 (m, 1H), 4.11 – 4.05 (m, 1H), 2.53 – 2.46 (m, 1H), 2.40 – 2.33 (m, 1H), 2.06 – 1.98 (m, 1H), 1.78 – 1.63 (m, 3H), 1.27 (s, 3H), 1.24 (s, 3H). ^{13}C NMR (100 MHz, Chloroform-*d*) δ 161.9 (d, *J* = 244.3 Hz), 133.8 (d, *J* = 3.3 Hz), 130.7, 127.4 (d, *J* = 7.8 Hz), 126.5 (d, *J* = 2.3 Hz), 115.3 (d, *J* = 21.2 Hz), 80.8, 78.0, 39.8, 38.4, 31.2, 29.2, 28.1. ^{19}F NMR (376 MHz, Chloroform-*d*) δ -115.6. HRMS (ESI) calculated for [$C_{15}H_{19}NaFO$] [M+Na]$^+$: 257.1312 found: 257.1303. Enantiomeric excess was determined by HPLC with a Chiralpak ASH column (hexanes: 2-propanol = 99.5: 0.5, 0.6 mL/min, 254 nm); major enantiomer tr = 15.387 min, minor enantiomer tr = 14.391 min.

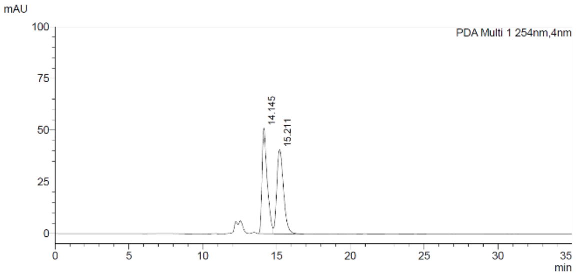

<Peak Table>

PDA Ch1 254nm

Peak#	Ret. Time	Height	Height%	Area	Area%
1	14.145	51006	55.673	1240202	49.025
2	15.211	40611	44.327	1289554	50.975
Total		91617	100.000	2529756	100.000

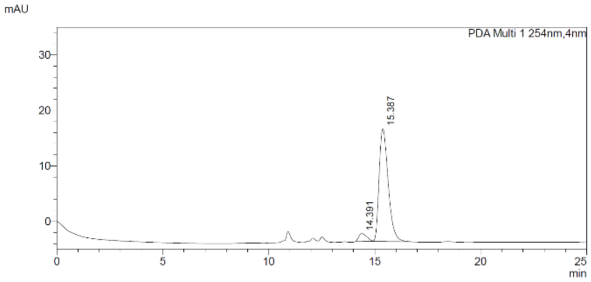

<Peak Table>

PDA Ch1 254nm

Peak#	Ret. Time	Height	Height%	Area	Area%
1	14.391	1390	6.403	36183	5.642
2	15.387	20325	93.597	605183	94.358
Total		21716	100.000	641367	100.000

4.1.23 (*S*, *E*)-5-(3-(2-methoxyphenyl)-allyl)-2,2-dimethyltetrahydrofuran (4h)

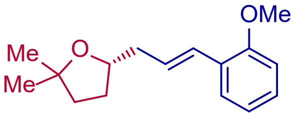

4h

Prepared according to typical procedure at RT for 72 hours by using $[Pd(allyl)Cl]_2$ (2.5 mol%), **Xu8** (10 mol%) from γ-hydroxyalkenes (0.2 mmol), NaO^tBu (0.4 mmol), alkenyl bromide (0.4 mmol) and no additive H_2O, after flash column chromatography on a silica gel (petroleum ether: ethyl acetate = 80: 1) give the product **4h** as a white liquid (34 mg, 68% yield) with 91% *ee*. $[\alpha]_D^{20}$ = -16.3 (*c* = 0.4, Chloroform).^{1}H NMR (400 MHz, Chloroform-*d*) δ 7.43 (dd, *J* = 7.6, 1.6 Hz, 1H), 7.21 – 7.17 (m, 1H), 6.91 (t, *J* = 7.6 Hz, 1H), 6.85 (d, *J* = 8.0 Hz, 1H), 6.77 (d, *J* = 16.0 Hz, 1H), 6.26 – 6.18 (m, 1H), 4.14 – 4.07 (m, 1H), 3.84 (s, 3H), 2.59 – 2.52 (m, 1H), 2.44 – 2.37 (m, 1H), 2.05 – 1.99 (m, 1H), 1.77 – 1.69 (m, 3H), 1.28 (s, 3H), 1.25 (s, 3H). ^{13}C NMR (100 MHz, Chloroform-*d*) δ 156.3, 127.9, 127.3, 126.7, 126.5, 126.4, 120.6, 110.8, 80.6, 78.2, 55.4, 40.2, 38.4, 31.1, 29.1, 28.1. HRMS (ESI) calculated for $[C_{16}H_{22}NaO_2]$ $[M+Na]^+$: 269.1512 found: 269.1508. Enantiomeric excess was determined by HPLC with a Chiralpak ODH+OD3 column (hexanes: 2-propanol = 99: 1, 0.5 mL/min, 254 nm); major enantiomer tr = 43.01 min, minor enantiomer tr = 38.70 min.

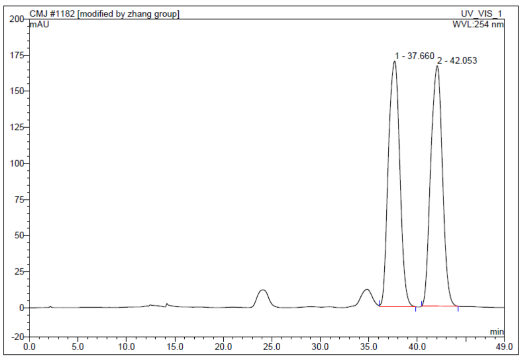

No.	Ret.Time min	Peak Name	Height mAU	Area mAU*min	Rel.Area %	Amount	Type
1	37.66	n.a.	170.118	233.404	50.16	n.a.	MB*
2	42.05	n.a.	166.414	231.920	49.84	n.a.	BMB*
Total:			336.532	465.323	100.00	0.000	

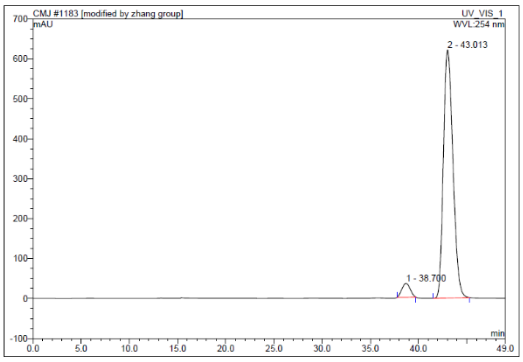

No.	Ret.Time min	Peak Name	Height mAU	Area mAU*min	Rel.Area %	Amount	Type
1	38.70	n.a.	34.788	34.652	4.65	n.a.	BMB*
2	43.01	n.a.	621.030	710.111	95.35	n.a.	BMB*
Total:			655.819	744.763	100.00	0.000	

4.1.24 (*S*)-5-cinnamyl-2,2-dimethyltetrahydrofuran (4i)

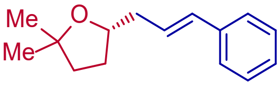

4i

Prepared according to typical procedure at RT for 72 hours by using [Pd(allyl)Cl]$_2$ (2.5 mol%), **Xu8** (10 mol%) from γ-hydroxyalkenes (0.2 mmol), NaO^{t}Bu (0.4 mmol), alkenyl bromide (0.4 mmol) and no additive H_2O, after flash column chromatography on a silica gel (petroleum ether: ethyl acetate = 80: 1) give the product **4i** as a white liquid (21 mg, 47% yield) with 90% *ee*. $[\alpha]_D^{20}$ = -14.3 (c = 0.4, Chloroform).^{1}H NMR (400 MHz, Chloroform-*d*) δ 7.37 – 7.35 (m, 2H), 7.31 – 7.26 (m, 2H), 7.22 – 7.18 (m, 1H), 6.44 (d, J = 16.0 Hz, 1H), 6.27 – 6.20 (m, 1H), 4.13 – 4.07 (m, 1H), 2.56 – 2.49 (m, 1H), 2.42 – 2.35 (m, 1H), 2.06 – 1.98 (m, 1H), 1.77 – 1.73 (m, 2H), 1.72 – 1.68 (m, 1H), 1.28 (s, 3H), 1.25 (s, 3H). ^{13}C NMR (100 MHz, Chloroform-*d*) δ 137.7, 131.9, 128.4, 126.9, 126.7, 126.0, 80.7, 78.1, 39.8, 38.4, 31.2, 29.2, 28.1. HRMS (ESI) calculated for [$C_{15}H_{20}NaO$] [M+Na]$^+$: 239.1406 found: 239.1398. Enantiomeric excess was determined by HPLC with a Chiralpak ODH+ODH+OD3 column (hexanes: 2-propanol = 99: 1, 0.5 mL/min, 254 nm); major enantiomer tr = 30.51 min, minor enantiomer tr = 26.75 min.

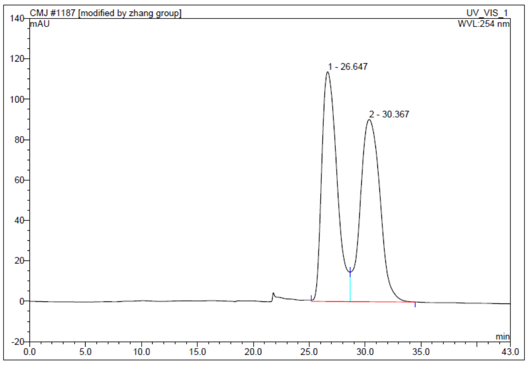

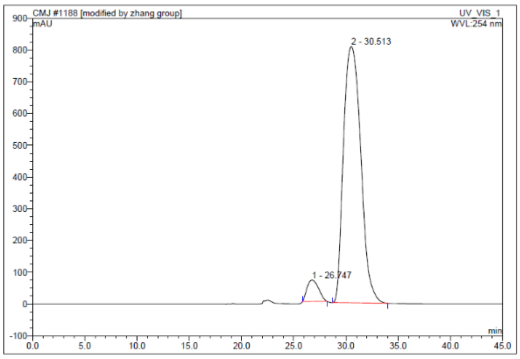

No.	Ret.Time min	Peak Name	Height mAU	Area mAU*min	Rel.Area %	Amount	Type
1	26.65	n.a.	113.645	179.576	49.33	n.a.	M *
2	30.37	n.a.	90.180	184.443	50.67	n.a.	MB*
Total:			203.825	364.019	100.00	0.000	

No.	Ret.Time min	Peak Name	Height mAU	Area mAU*min	Rel.Area %	Amount	Type
1	26.75	n.a.	67.322	86.004	5.25	n.a.	BMB*
2	30.51	n.a.	807.107	1551.586	94.75	n.a.	BMB*
Total:			874.430	1637.590	100.00	0.000	

4.1.25 (*S*, *E*)-2,2-dimethyl-5-(3-(naphthalen-1-yl)-allyl)tetrahydrofuran (4j)

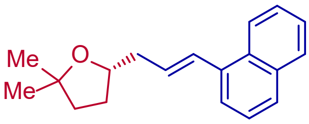

4j

Prepared according to typical procedure at RT for 72 hours by using $[Pd(allyl)Cl]_2$ (2.5 mol%), **Xu8** (10 mol%) from γ-hydroxyalkenes (0.2 mmol), NaO^{t}Bu (0.4 mmol), alkenyl bromide (0.4 mmol) and no additive H_2O, after flash column chromatography on a silica gel (petroleum ether: ethyl acetate = 80: 1) give the product **4j** as a white liquid (39 mg, 73% yield) with 89% *ee*. $[\alpha]_D^{20}$ = -15.7 (*c* = 0.4, Chloroform).^{1}H NMR (400 MHz, Chloroform-*d*) δ 8.15 (d, *J* = 7.6 Hz, 1H), 7.87 – 7.84 (m, 1H), 7.77 (d, *J* = 8.0 Hz, 1H), 7.60 (d, *J* = 7.2 Hz, 1H), 7.55 – 7.49 (m, 2H), 7.48 – 7.43 (m, 1H), 7.21 (d, *J* = 15.6 Hz, 1H), 6.35 – 6.20 (m, 1H), 4.27 – 4.12 (m, 1H), 2.74 – 2.62 (m, 1H), 2.60 – 2.47 (m, 1H), 2.15 – 2.05 (m, 1H), 1.86 – 1.74 (m, 4H), 1.33 (s, 4H), 1.29 (s, 4H). ^{13}C NMR (100 MHz, Chloroform-*d*) δ 135.4, 133.6, 131.1, 129.9, 129.2, 128.4, 127.3, 125.8, 125.6, 123.9, 123.6, 80.7, 78.0, 40.1, 38.5, 31.2, 29.2, 28.1. ^{13}C NMR (100 MHz, Chloroform-*d*) δ 137.7, 131.9, 128.4, 126.9, 126.7, 126.0, 80.7, 78.1, 39.8, 38.4, 31.2, 29.2, 28.1. HRMS (ESI) calculated for $[C_{19}H_{22}NaO]$ $[M+Na]^+$: 289.1563 found: 289.1559. Enantiomeric excess was determined by HPLC with a Chiralpak ODH+ODH column (hexanes: 2-propanol = 99: 1, 0.8 mL/min, 210 nm); major enantiomer tr = 20.29 min, minor enantiomer tr = 23.36 min.

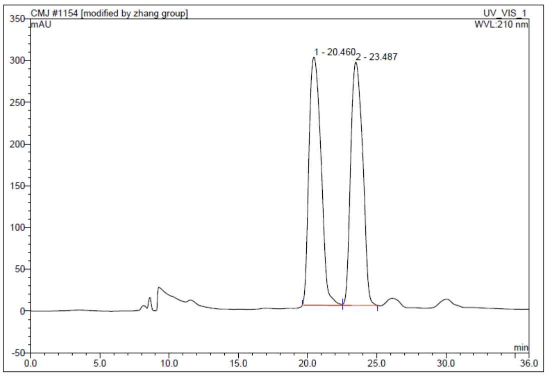

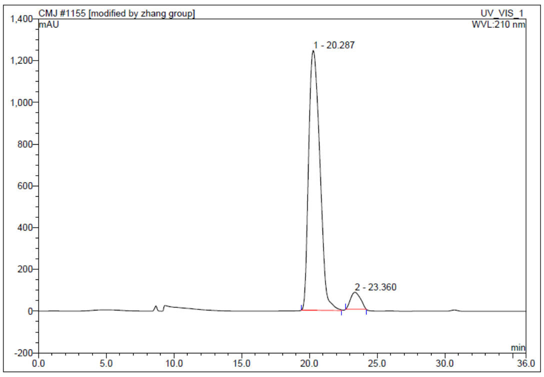

No.	Ret.Time min	Peak Name	Height mAU	Area mAU*min	Rel.Area %	Amount	Type
1	20.46	n.a.	296.784	299.000	50.72	n.a.	BM *
2	23.49	n.a.	291.250	290.503	49.28	n.a.	MB*
Total:			588.034	589.503	100.00	0.000	

No.	Ret.Time min	Peak Name	Height mAU	Area mAU*min	Rel.Area %	Amount	Type
1	20.29	n.a.	1244.059	1194.477	94.56	n.a.	BM *
2	23.36	n.a.	80.944	68.668	5.44	n.a.	BMB*
Total:			1325.003	1263.145	100.00	0.000	

4.1.26 (*S*, *E*)-2,2-dimethyl-5-(3-(naphthalen-2-yl)-allyl)tetrahydrofuran (4k)

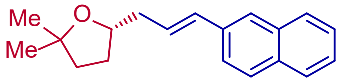

4k

Prepared according to typical procedure at RT for 72 hours by using $[Pd(allyl)Cl]_2$ (2.5 mol%), **Xu8** (10 mol%) from γ-hydroxyalkenes (0.2 mmol), NaO^{t}Bu (0.4 mmol), alkenyl bromide (0.4 mmol) and no additive H_2O, after flash column chromatography on a silica gel (petroleum ether: ethyl acetate = 80: 1) give the product **4k** as a white liquid (38 mg, 71% yield) with 87% *ee*. $[\alpha]_D^{20}$ = -13.7 (*c* = 0.4, Chloroform). ^{1}H NMR (400 MHz, Chloroform-*d*) δ 7.781 – 7.768 (m, 3H), 7.71 (s, 1H), 7.61 (d, *J* = 8.4 Hz, 1H), 7.48 – 7.41 (m, 2H), 6.63 (d, *J* = 15.6 Hz, 1H), 6.43 – 6.36 (m, 1H), 4.19 – 4.13 (m, 1H), 2.64 – 2.57 (m, 1H), 2.50 – 2.43 (m, 1H), 2.13 – 2.03 (m, 1H), 1.80 – 1.72 (m, 3H), 1.32 (s, 3H), 1.29 (s, 3H). ^{13}C NMR (100 MHz, Chloroform-*d*) δ 135.1, 133.6, 132.7, 132.0, 128.0, 127.8, 127.6, 127.2, 126.1, 125.5, 125.5, 123.6, 80.7, 78.1, 40.0, 38.4, 31.2, 29.2, 28.1. HRMS (ESI) calculated for $[C_{19}H_{22}NaO]$ $[M+Na]^+$: 289.1563 found: 289.1556. Enantiomeric excess was determined by HPLC with a Chiralpak OJH column (hexanes: 2-propanol = 99: 1, 0.5 mL/min, 254 nm); major enantiomer tr = 35.54 min, minor enantiomer tr = 40.26 min.

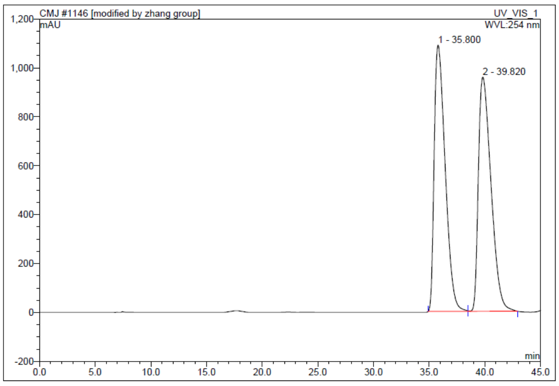

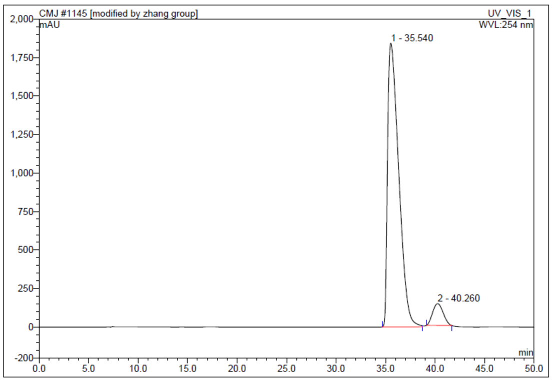

No.	Ret.Time min	Peak Name	Height mAU	Area mAU*min	Rel.Area %	Amount	Type
1	35.80	n.a.	1088.639	1245.067	49.79	n.a.	BM *
2	39.82	n.a.	957.640	1255.733	50.21	n.a.	MB*
Total:			2046.279	2500.800	100.00	0.000	

No.	Ret.Time min	Peak Name	Height mAU	Area mAU*min	Rel.Area %	Amount	Type
1	35.54	n.a.	1840.915	2403.351	93.25	n.a.	BM *
2	40.26	n.a.	140.914	173.832	6.75	n.a.	BMB*
Total:			1981.829	2577.183	100.00	0.000	

4.1.27 (*S*)-2,2-dimethyl-5-((2*E*, 4*E*)-5-phenylpenta-2,4-dien-1-yl)tetrahydrofuran (4l)

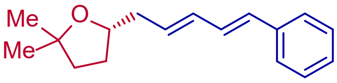

4l

Prepared according to typical procedure at RT for 72 hours by using $[Pd(allyl)Cl]_2$ (2.5 mol%), **Xu8** (10 mol%) from γ-hydroxyalkenes (0.2 mmol), NaO^{t}Bu (0.4 mmol), alkenyl bromide (0.4 mmol) and no additive H_2O, after flash column chromatography on a silica gel (petroleum ether: ethyl acetate = 80: 1) give the product **4l** as a white liquid (27 mg, 56% yield) with 83% *ee*. $[\alpha]_D^{20}$ = -8.9 (*c* = 0.4, Chloroform). ^{1}H NMR (400 MHz, Chloroform-*d*) δ 7.39 – 7.37 (m, 2H), 7.31 – 7.26 (m, 2H), 7.22 – 7.18 (m, 1H), 6.76 (dd, *J* = 16.0, 10.0 Hz, 1H), 6.46 (d, *J* = 15.6 Hz, 1H), 6.26 (dd, *J* = 15.2, 10.4 Hz, 1H), 5.87 – 5.79 (m, 1H), 4.09 – 4.02 (m, 1H), 2.49 – 2.43 (m, 1H), 2.35 – 2.28 (m, 1H), 2.06 – 1.98 (m, 1H), 1.76 – 1.64 (m, 3H), 1.27 (s, 3H), 1.24 (s, 3H). ^{13}C NMR (100 MHz, Chloroform-*d*) δ 137.5, 132.6, 131.4, 130.5, 129.3, 128.5, 127.1, 126.1, 80.7, 78.0, 39.8, 38.4, 31.3, 29.2, 28.1. HRMS (ESI) calculated for $[C_{17}H_{22}NaO]$ $[M+Na]^+$: 265.1563 found: 265.1558. Enantiomeric excess was determined by HPLC with a Chiralpak OJH column (hexanes: 2-propanol = 99: 1, 0.5 mL/min, 254 nm); major enantiomer tr = 19.39 min, minor enantiomer tr = 25.35 min.

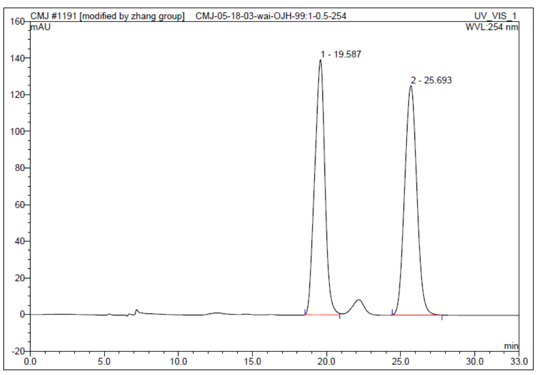

No.	Ret.Time min	Peak Name	Height mAU	Area mAU*min	Rel.Area %	Amount	Type
1	19.59	n.a.	139.275	111.190	48.31	n.a.	BM *
2	25.69	n.a.	125.180	118.970	51.69	n.a.	BMB*
Total:			264.455	230.160	100.00	0.000	

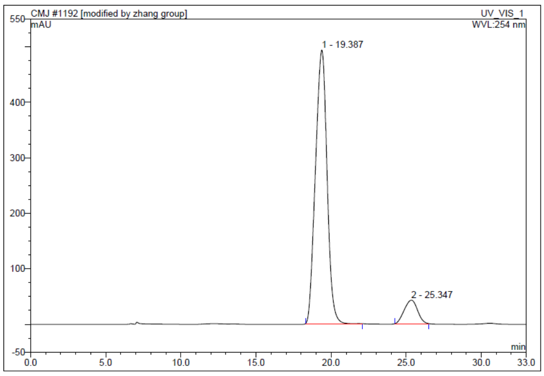

No.	Ret.Time min	Peak Name	Height mAU	Area mAU*min	Rel.Area %	Amount	Type
1	19.39	n.a.	492.752	440.381	91.06	n.a.	BMB*
2	25.35	n.a.	42.328	43.261	8.94	n.a.	BMB*
Total:			535.080	483.643	100.00	0.000	

4.1.29 Synthesis of (5*S*)-2-butyl-5-((*E*)-3-(4-methoxyphenyl)allyl)-2-methyltetrahydrofuran (4m)

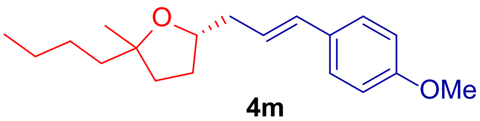

4m

Prepared according to typical procedure at RT for 72 hours by using $[Pd(allyl)Cl]_2$ (2.5 mol%), **Xu8** (10 mol%) from γ-hydroxyalkenes (0.2 mmol) and alkenyl bromide (0.4 mmol), after flash column chromatography on a silica gel (petroleum ether: ethyl acetate = 50: 1) give the product **4m** as a white liquid (32 mg, 56% yield, dr : 1.1:1) with 84% *ee* (major)，93% *ee* (minor). $[\alpha]_D^{20}$ = -11.2 (*c* = 0.4, Chloroform).^{1}H NMR (400 MHz, Chloroform-*d*) δ 7.27 (d, *J* = 8.7 Hz, 2H), 6.86 – 6.80 (m, 2H), 6.37 (ddt, *J* = 15.8, 2.8, 1.4 Hz, 1H), 6.08 (dt, *J* = 15.9, 7.2 Hz, 1H), 4.05 (dqd, *J* = 22.6, 6.9, 5.3 Hz, 1H), 3.79 (s, 3H), 2.50 (ddddd, *J* = 13.9, 12.5, 6.9, 5.2, 1.5 Hz, 1H), 2.34 (dtdd, *J* = 14.0, 7.1, 4.3, 1.4 Hz, 1H), 2.06 – 1.90 (m, 1H), 1.82 – 1.73 (m, 1H), 1.70 – 1.61 (m, 2H), 1.50 (qd, *J* = 5.8, 2.1 Hz, 1H), 1.36 – 1.29 (m, 5H), 1.19 (d, *J* = 7.8 Hz, 3H), 0.91 – 0.87 (m, 3H). ^{13}C NMR (100 MHz, Chloroform-*d*) δ 158.8, 131.2, 130.6, 127.1, 124.6, 113.9, 83.1, 78.5, 55.3, 41.5, 39.8, 36.9, 31.2, 27.1, 24.4, 22.7, 14.1. HRMS (ESI) calculated for $[C_{19}H_{26}NaO_2]$ $[M+Na]^+$: 309.1825 found: 309.1820. Enantiomeric excess was determined by HPLC with a Chiralpak ADH-ADH-ADH column (hexanes: 2-propanol = 99: 1, 0.6 mL/min, 254 nm); Minor [tr (minor) = 24.408 min, tr (major) = 31.454 min; 84% ee] major [tr (minor) = 29.766 min, tr (major) = 25.831 min; 90% ee].

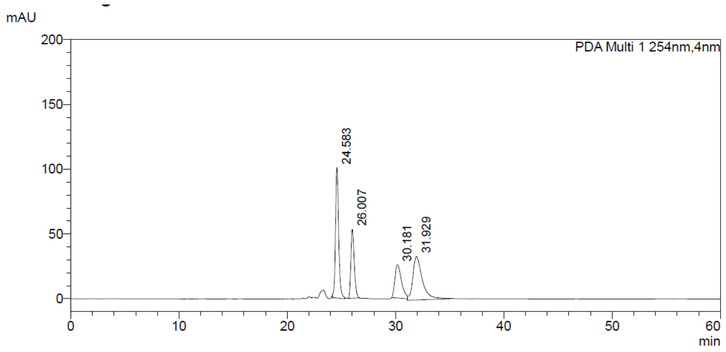

<Peak Table>

PDA Ch1 254nm

Peak#	Ret. Time	Height	Height%	Area	Area%
1	24.583	100810	47.256	2031825	32.232
2	26.007	53356	25.011	1149676	18.238
3	30.181	25737	12.065	1080062	17.134
4	31.929	33425	15.668	2042199	32.397
Total		213329	100.000	6303763	100.000

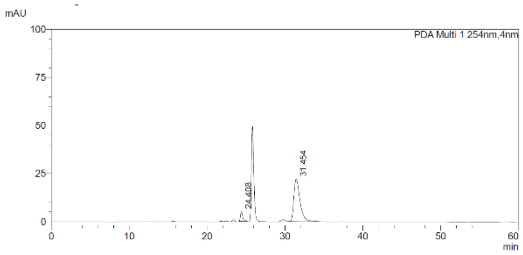

<Peak Table>

PDA Ch1 254nm

Peak#	Ret. Time	Height	Height%	Area	Area%
1	24.408	5288	19.274	105439	8.001
2	31.454	22147	80.726	1212358	91.999
Total		27434	100.000	1317797	100.000

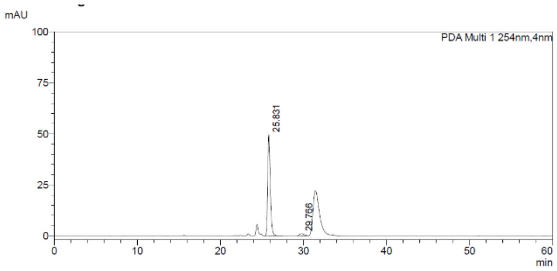

<Peak Table>

PDA Ch1 254nm

Peak#	Ret. Time	Height	Height%	Area	Area%
1	25.831	49645	98.000	1073331	96.584
2	29.766	1013	2.000	37964	3.416
Total		50658	100.000	1111295	100.000

4.1.30 Synthesis of (5*S*)-2-(but-3-en-1-yl)-5-((*E*)-3-(4-methoxyphenyl)allyl)-2-methyltetrahydrofuran (4n)

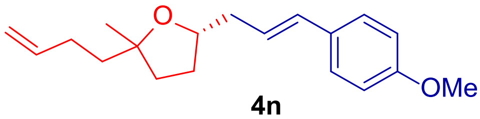

4n

Prepared according to typical procedure at RT for 72 hours by using [Pd(allyl)Cl]$_2$ (2.5 mol%), **Xu6** (10 mol%) from γ-hydroxyalkenes (0.2 mmol) and alkenyl bromide (0.4 mmol), after flash column chromatography on a silica gel (petroleum ether: ethyl acetate = 50: 1) give the product **4n** as a white liquid (27 mg, 52% yield, dr : 2:1) with 82% *ee* (major)，80% *ee* (minor). $[\alpha]_D^{20}$ = -7.1 (*c* = 0.4, Chloroform).^{1}H NMR (400 MHz, Chloroform-*d*) δ 7.28 (d, *J* = 8.5 Hz, 2H), 6.83 (dd, *J* = 8.6, 1.5 Hz, 2H), 6.38 (dd, *J* = 15.9, 2.8 Hz, 1H), 6.17 – 6.00 (m, 1H), 5.85 (ddt, *J* = 16.8, 10.2, 6.5 Hz, 1H), 5.09 – 4.79 (m, 2H), 4.17 – 3.93 (m, 1H), 3.80 (s, 3H), 2.56 – 2.44 (m, 1H), 2.36 (dtd, *J* = 14.1, 7.1, 1.3 Hz, 1H), 2.23 – 2.06 (m, 2H), 2.06 – 1.94 (m, 1H), 1.86 – 1.73 (m, 1H), 1.67 (dddd, *J* = 20.1, 10.1, 7.4, 5.4 Hz, 4H), 1.26 – 1.20 (m, 3H). ^{13}C NMR (100 MHz, Chloroform-*d*) δ 158.8, 139.1, 131.3, 130.5, 127.1, 124.5, 114.0, 113.9, 82.8, 78.6, 55.3, 40.4, 39.8, 37.0, 31.2, 29.0,

27.1.RMS (ESI) calculated for [$C_{19}H_{26}NaO_2$] [M+Na]$^+$: 309.1825 found: 309.1820. Enantiomeric excess was determined by HPLC with a Chiralpak OJH-OJH-OJ3 column (hexanes: 2-propanol = 99.5: 0.5, 0.6 mL/min, 254 nm); Minor [tr (minor) = 86.695 min, tr (major) = 81.929 min; 80% ee] major [tr (minor) = 90.829 min, tr (major) = 84.875 min; 92% ee]

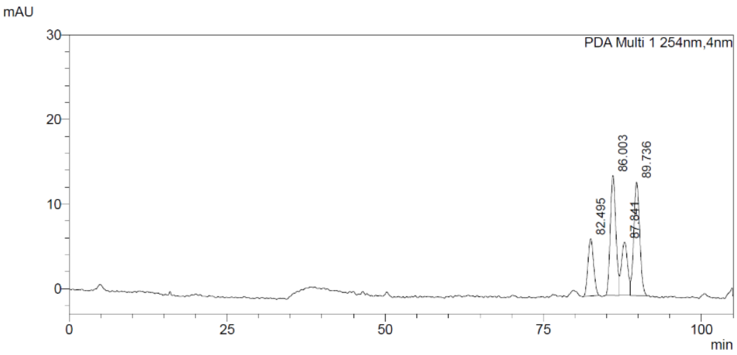

<Peak Table>

PDA Ch1 254nm

Peak#	Ret. Time	Height	Height%	Area	Area%
1	82.495	6753	16.614	421079	16.146
2	86.003	14167	34.854	874184	33.520
3	87.841	6320	15.550	429223	16.458
4	89.736	13406	32.982	883432	33.875
Total		40646	100.000	2607918	100.000

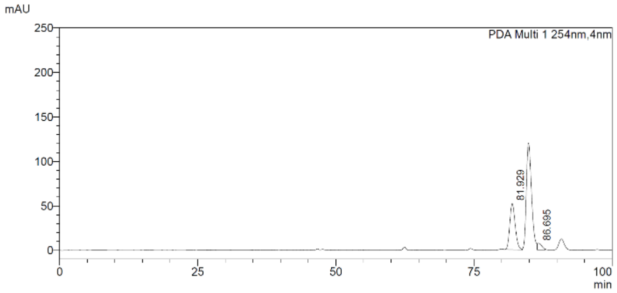

<Peak Table>

PDA Ch1 254nm

Peak#	Ret. Time	Height	Height%	Area	Area%
1	81.929	51645	87.926	3507763	90.103
2	86.695	7092	12.074	385309	9.897
Total		58737	100.000	3893072	100.000

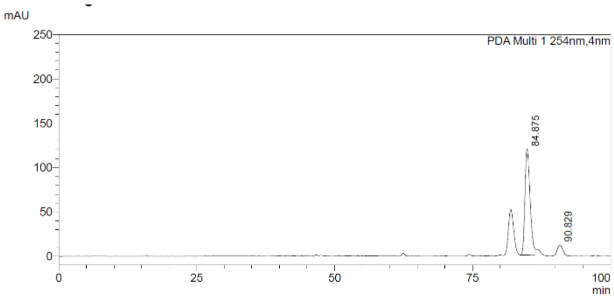

<Peak Table>

PDA Ch1 254nm

Peak#	Ret. Time	Height	Height%	Area	Area%
1	84.875	119152	90.954	7727271	90.951
2	90.829	11850	9.046	768775	9.049
Total		131003	100.000	8496045	100.000

4.1.31 Synthesis of (2*R*)-2-cinnamyl-5-methyltetrahydrofuran (4o)

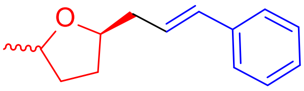

4o

Prepared according to typical procedure at -20 °C for 72 hours by using [Pd(allyl)Cl]$_2$ (2.5 mol%), **(*R*, *R*$_S$)-Xu9** (10 mol%) from γ-hydroxyalkenes (0.5 mmol) and alkenyl bromide (1 mmol), after flash column chromatography on a silica gel (petroleum ether: ethyl acetate = 50: 1) give the product **4o** as a white liquid (21 mg, 21% yield, dr = 1.1:1, 94% ee(major), 80% ee (minor). $[\alpha]_D^{20}$ = 4.3 (*c* = 0.4, Chloroform).^{1}H NMR (400 MHz, Chloroform-*d*) δ 7.39 – 7.33 (m, 2H), 7.29 (dd, *J* = 8.5, 6.7 Hz, 2H), 7.23 – 7.16 (m, 1H), 6.48 – 6.40 (m, 1H), 6.23 (dtd, *J* = 15.8, 7.1, 5.4 Hz, 1H),

4.14 (ddt, J = 8.1, 5.8, 3.4 Hz, 1H), 3.97 (dqd, J = 12.5, 6.3, 2.7 Hz, 1H), 2.52 (ddddd, J = 14.2, 10.2, 7.2, 5.8, 1.4 Hz, 1H), 2.46 – 2.31 (m, 1H), 2.05 (dddd, J = 12.7, 11.3, 5.6, 2.6 Hz, 1H), 2.00 – 1.91 (m, 1H), 1.68 – 1.61 (m, 1H), 1.46 (ddddd, J = 11.8, 9.8, 8.4, 4.9, 3.5 Hz, 1H), 1.24 (dd, J = 13.0, 6.0 Hz, 3H). ^{13}C NMR (100 MHz, Chloroform-*d*) δ 137.6, 132.0, 128.5, 127.0, 126.8, 126.1, 78.9, 75.5, 39.7, 33.9, 31.8, 21.4. HRMS (ESI) calculated for [$C_{14}H_{19}O$] [M+H]$^+$: 203.1430 found: 203.1435. Enantiomeric excess was determined by HPLC with a Chiralpak OD-H column (hexanes: 2-propanol = 95: 5, 0.6 mL/min, 254 nm); Minor [tr (minor) = 7.887 min, tr (major) = 8.362 min; 80% ee] major [tr (minor) = 10.327 min, tr (major) = 7.119 min; 94% ee]

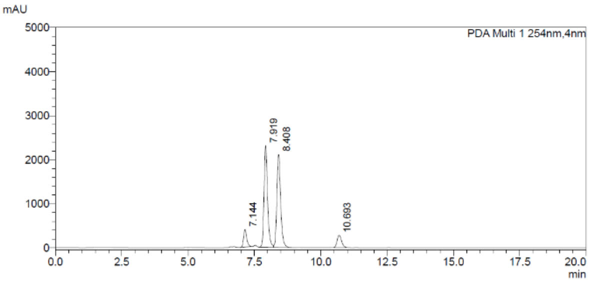

<Peak Table>

PDA Ch1 254nm

Peak#	Ret. Time	Height	Height%	Area	Area%
1	7.144	398617	7.818	3318975	6.840
2	7.919	2310089	45.308	21087685	43.460
3	8.408	2114753	41.477	20906902	43.088
4	10.693	275171	5.397	3208193	6.612
Total		5098630	100.000	48521755	100.000

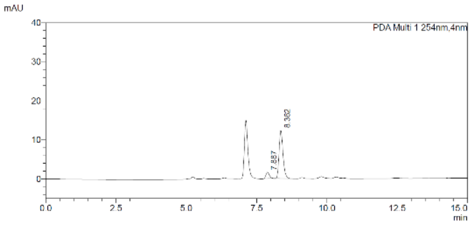

<Peak Table>

PDA Ch1 254nm

Peak#	Ret. Time	Height	Height%	Area	Area%
1	7.887	1587	11.408	13350	10.134
2	8.362	12321	88.592	118386	89.866
Total		13908	100.000	131736	100.000

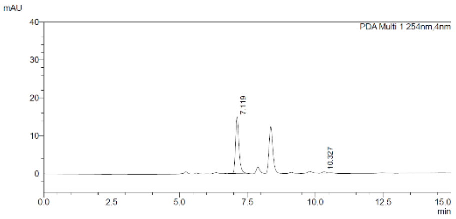

<Peak Table>

PDA Ch1 254nm

Peak#	Ret. Time	Height	Height%	Area	Area%
1	7.119	14973	97.453	132553	96.947
2	10.327	391	2.547	4174	3.053
Total		15364	100.000	136727	100.000

4.1.32 (*S*, *E*)-2-(3-(4-methoxyphenyl)-allyl)-2-methyl-1-oxaspiro[4.5]decane (6a)

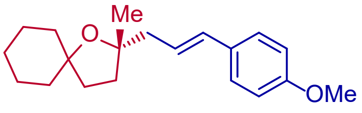

6a

Prepared according to typical procedure at RT for 72 hours by using [Pd(allyl)Cl]$_2$ (2.5 mol%), **Xu8** (10 mol%) from γ-hydroxyalkenes (0.2 mmol) and alkenyl bromide (0.4 mmol), after flash column chromatography on a silica gel (petroleum ether: ethyl acetate = 80: 1) give the product **6a** as a white liquid (38 mg, 63% yield) with 88% *ee*. $[\alpha]_D^{20}$= -6.1 (c = 0.4, Chloroform). ^{1}H NMR (400 MHz, Chloroform-*d*) δ 7.28 (d, J = 8.8 Hz, 2H), 6.84 (d, J = 8.8 Hz, 2H), 6.33 (d, J = 15.6 Hz, 1H),

6.15 – 6.08 (m, 1H), 3.80 (s, 3H), 2.38 – 2.35 (m, 2H), 1.97 – 1.92 (m, 1H), 1.85 – 1.76 (m, 2H), 1.72 – 1.64 (m, 5H), 1.56 – 1.51 (m, 4H), 1.34 – 1.28 (m, 2H), 1.24 (s, 3H). ^{13}C NMR (100 MHz, Chloroform-*d*) δ 158.7, 131.6, 130.7, 127.1, 125.1, 113.9, 83.4, 82.4, 55.3, 46.0, 39.4, 38.8, 35.7, 28.4, 25.7, 24.2. HRMS (ESI) calculated for [$C_{20}H_{28}NaO_2$] [M+Na]$^+$: 323.1982 found: 323.1972. Enantiomeric excess was determined by HPLC with a Chiralpak ADH+ADH column (hexanes: 2-propanol = 99: 1, 0.5 mL/min, 254 nm); major enantiomer tr = 24.63 min, minor enantiomer tr = 19.59 min.

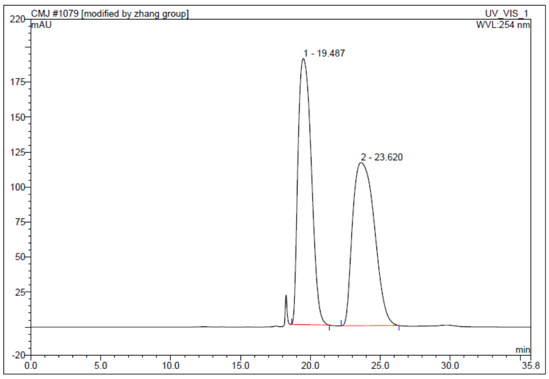

No.	Ret.Time min	Peak Name	Height mAU	Area mAU*min	Rel.Area %	Amount	Type
1	19.49	n.a.	190.105	209.880	49.81	n.a.	BMB*
2	23.62	n.a.	116.605	211.479	50.19	n.a.	BMB*
Total:			306.709	421.359	100.00	0.000	

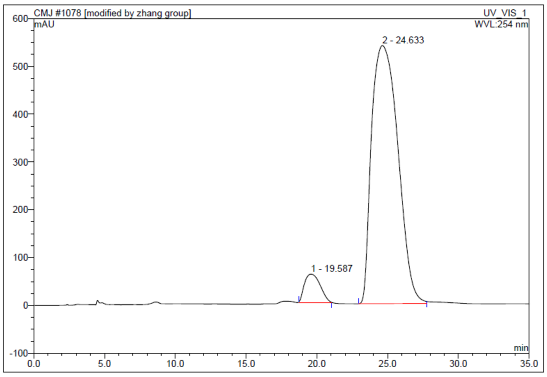

No.	Ret.Time min	Peak Name	Height mAU	Area mAU*min	Rel.Area %	Amount	Type
1	19.59	n.a.	59.535	76.526	6.16	n.a.	MB*
2	24.63	n.a.	539.830	1166.426	93.84	n.a.	BM *
Total:			599.365	1242.952	100.00	0.000	

4.1.33 (*S*)-2-cinnamyl-2-methyl-1-oxaspiro[4.4]nonane (6b)

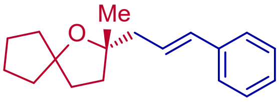

6b

Prepared according to typical procedure at RT for 72 hours by using [Pd(allyl)Cl]$_2$ (2.5 mol%), **Xu8** (10 mol%) from γ-hydroxyalkenes (0.2 mmol) and alkenyl bromide (0.4 mmol), after flash column chromatography on a silica gel (petroleum ether: ethyl acetate = 80: 1) give the product **6b** as a white liquid (47 mg, 91% yield) with 90% *ee*. $[\alpha]_D^{20}$ = -4.1 (*c* = 0.4, Chloroform). ^{1}H NMR (400 MHz, Chloroform-*d*) δ 7.38 – 7.36 (m, 2H), 7.32 – 7.28 (m, 2H), 7.23 – 7.17 (m, 1H), 6.41 (d, *J* = 16.0 Hz, 1H), 6.30 – 6.22 (m, 1H), 2.42 – 2.39 (m, 2H), 1.97 – 1.87 (m, 3H), 1.84 – 1.72 (m, 5H), 1.64 – 1.53 (m, 4H), 1.26 (s, 3H). ^{13}C NMR (100 MHz, Chloroform-*d*) δ 137.7, 132.3, 128.4, 127.2, 126.9, 126.0, 91.5, 82.6, 45.9, 39.8, 39.4, 37.2, 36.6, 28.0, 23.9, 23.8. HRMS (ESI) calculated for [$C_{18}H_{24}NaO$] [M+Na]$^+$: 279.1719 found: 279.1717. Enantiomeric excess was determined by HPLC

with a Chiralpak IA+IA column (hexanes: 2-propanol = 99: 1, 0.3 mL/min, 254 nm); major enantiomer tr = 29.01 min, minor enantiomer tr = 28.07 min.

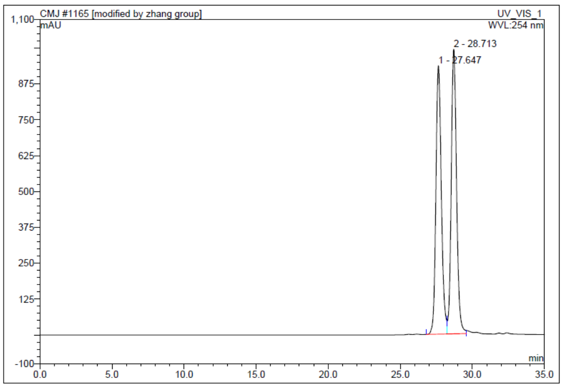

No.	Ret.Time min	Peak Name	Height mAU	Area mAU*min	Rel.Area %	Amount	Type
1	27.65	n.a.	935.553	410.474	49.22	n.a.	BM *
2	28.71	n.a.	992.006	423.508	50.78	n.a.	M *
Total:			1927.559	833.982	100.00	0.000	

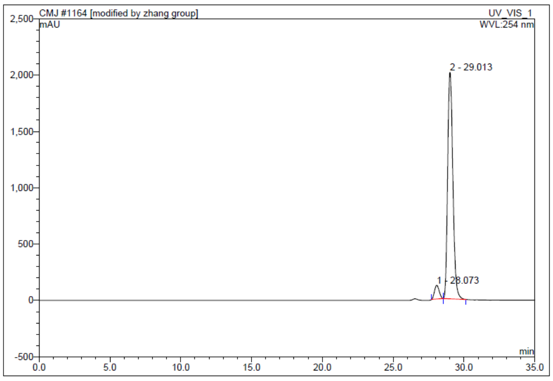

No.	Ret.Time min	Peak Name	Height mAU	Area mAU*min	Rel.Area %	Amount	Type
1	28.07	n.a.	121.916	46.445	5.14	n.a.	BMb*
2	29.01	n.a.	2010.599	857.131	94.86	n.a.	BMB*
Total:			2132.515	903.576	100.00	0.000	

4.1.34 (*S*, *E*)-2-(3-(4-methoxyphenyl)-allyl)-2-methyl-1-oxaspiro[4.4]nonane (6c)

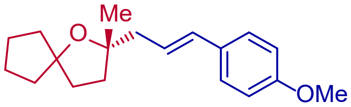

6c

Prepared according to typical procedure at RT for 72 hours by using [Pd(allyl)Cl]$_2$ (2.5 mol%), **Xu8** (10 mol%) from γ-hydroxyalkenes (0.2 mmol) and alkenyl bromide (0.4 mmol), after flash column chromatography on a silica gel (petroleum ether: ethyl acetate = 80: 1) give the product **6c** as a white liquid (51 mg, 90% yield) with 90% *ee*. $[\alpha]_D^{20}$ = -5.2 (*c* = 0.4, Chloroform). ^{1}H NMR (400 MHz, Chloroform-*d*) δ 7.29 (d, *J* = 8.8 Hz, 2H), 6.84 (d, *J* = 8.8 Hz, 2H), 6.35 (d, *J* = 15.6 Hz, 1H), 6.14 – 6.07 (m, 1H), 3.79 (s, 3H), 2.40 – 2.37 (m, 2H), 1.98 – 1.86 (m, 3H), 1.83 – 1.70 (m, 5H), 1.63 – 1.54 (m, 4H), 1.25 (s, 3H). ^{13}C NMR (100 MHz, Chloroform-*d*) δ 158.7, 131.6, 130.6, 127.0, 124.9, 113.9, 91.4, 82.7, 55.2, 45.8, 39.7, 39.4, 37.2, 36.5, 27.9, 23.8, 23.8. HRMS (ESI) calculated for [$C_{19}H_{26}NaO_2$] $[M+Na]^+$: 309.1825 found: 309.1832. Enantiomeric excess was determined by HPLC with a Chiralpak IC+IC column (hexanes: 2-propanol = 99: 1, 0.5 mL/min, 254 nm); major enantiomer tr = 20.47 min, minor enantiomer tr = 25.47 min.

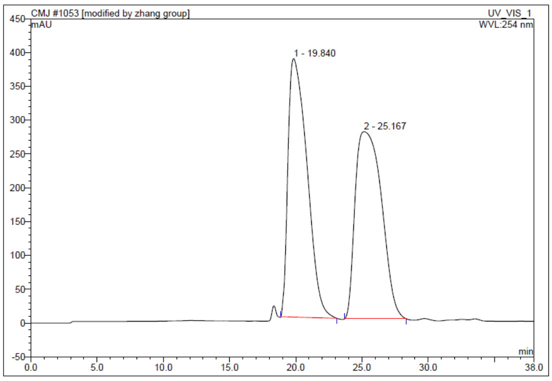

No.	Ret.Time min	Peak Name	Height mAU	Area mAU*min	Rel.Area %	Amount	Type
1	19.84	n.a.	382.411	636.893	49.85	n.a.	BMB*
2	25.17	n.a.	276.698	640.812	50.15	n.a.	BMB*
Total:			659.109	1277.704	100.00	0.000	

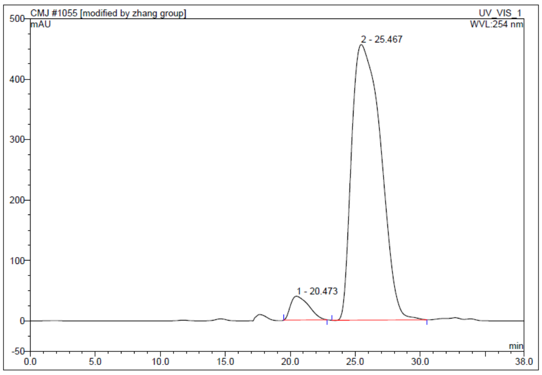

No.	Ret.Time min	Peak Name	Height mAU	Area mAU*min	Rel.Area %	Amount	Type
1	20.47	n.a.	39.276	63.688	5.10	n.a.	BMB*
2	25.47	n.a.	455.705	1186.216	94.90	n.a.	BMB*
Total:			494.981	1249.904	100.00	0.000	

4.1.35 (*S*, *E*)-2-(3-(2-methoxyphenyl)-allyl)-2-methyl-1-oxaspiro[4.4]nonane (6d)

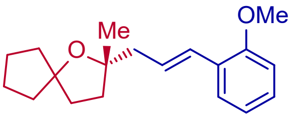

6d

Prepared according to typical procedure at RT for 72 hours by using $[Pd(allyl)Cl]_2$ (2.5 mol%), **Xu8** (10 mol%) from γ-hydroxyalkenes (0.2 mmol) and alkenyl bromide (0.4 mmol), after flash column chromatography on a silica gel (petroleum ether: ethyl acetate = 80: 1) give the product **6d** as a white liquid (59 mg, 91% yield) with 91% *ee*. $[\alpha]_D^{20}$= -9.7 (*c* = 0.4, Chloroform). ^{1}H NMR (400 MHz, Chloroform-*d*) δ 7.45 (dd, *J* = 7.6, 2.0 Hz, 1H), 7.21 – 7.17 (m, 1H), 6.92 (t, *J* = 7.6 Hz, 1H), 6.86 (d, *J* = 8.4 Hz, 1H), 6.74 (d, *J* = 16.0 Hz, 1H), 6.29 – 6.22 (m, 1H), 3.84 (s, 3H), 2.49 – 2.38 (m, 2H), 2.01 – 1.94 (m, 1H), 1.92 – 1.88 (m, 2H), 1.84 – 1.69 (m, 5H), 1.64 – 1.53 (m, 4H), 1.27 (s, 3H). ^{13}C NMR (100 MHz, Chloroform-*d*) δ 156.3, 127.8, 127.7, 126.8, 126.8, 126.4, 120.6, 110.8, 91.4, 82.7, 55.4, 46.3, 39.8, 39.4, 37.2, 36.4, 28.0, 23.9, 23.8. HRMS (ESI) calculated for $[C_{19}H_{26}NaO_2]$ $[M+Na]^+$: 309.1825 found: 309.1817. Enantiomeric excess was determined by HPLC with a Chiralpak OJH+OJ3 column (hexanes: 2-propanol = 99: 1, 0.5 mL/min, 254 nm); major enantiomer tr = 41.54 min, minor enantiomer tr = 24.25 min.

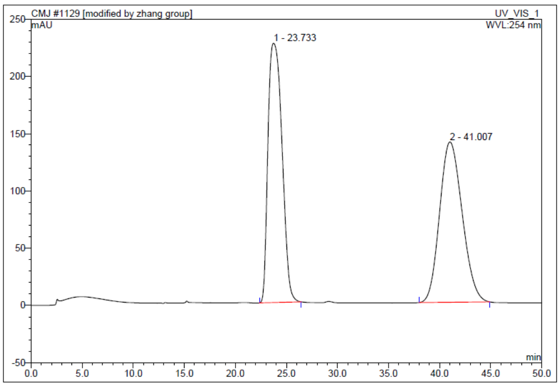

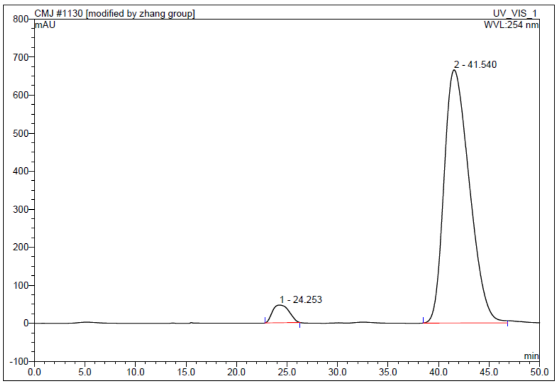

No.	Ret.Time min	Peak Name	Height mAU	Area mAU*min	Rel.Area %	Amount	Type
1	23.73	n.a.	226.529	360.978	50.17	n.a.	BMB*
2	41.01	n.a.	140.060	358.594	49.83	n.a.	BMB*
Total:			366.589	719.573	100.00	0.000	

No.	Ret.Time min	Peak Name	Height mAU	Area mAU*min	Rel.Area %	Amount	Type
1	24.25	n.a.	46.364	88.746	4.40	n.a.	BMB*
2	41.54	n.a.	665.365	1929.304	95.60	n.a.	BM *
Total:			711.729	2018.050	100.00	0.000	

4.1.36 (*S*, *E*)-2-(3-(4-fluorophenyl)-allyl)-2-methyl-1-oxaspiro[4.4]nonane (6e)

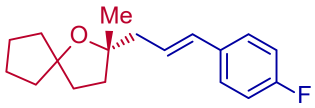

6e

Prepared according to typical procedure at RT for 72 hours by using $[Pd(allyl)Cl]_2$ (2.5 mol%), **Xu8** (10 mol%) from γ-hydroxyalkenes (0.2 mmol) and alkenyl bromide (0.4 mmol), after flash column chromatography on a silica gel (petroleum ether: ethyl acetate = 80: 1) give the product **6e** as a white liquid (54 mg, 93% yield) with 90% *ee*. $[\alpha]_D^{20}$ = -1.5 (*c* = 0.4, Chloroform). ^{1}H NMR (400 MHz, Chloroform-*d*) δ 7.32 – 7.29 (m, 2H), 7.00 – 6.95 (m, 2H), 6.36 (d, *J* = 15.6 Hz, 1H), 6.20 – 6.12 (m, 1H), 2.38 (d, *J* = 7.2 Hz, 2H), 1.96 – 1.90 (m, 2H), 1.87 – 1.85 (m, 1H), 1.83 – 1.71 (m, 5H), 1.63 – 1.54 (m, 4H), 1.24 (s, 3H). ^{13}C NMR (100 MHz, Chloroform-*d*) δ 161.9 (d, *J* = 244.2 Hz), 133.9 (d, *J* = 3.3 Hz), 131.1, 127.4 (d, *J* = 7.8 Hz), 126.9 (d, *J* = 2.2 Hz), 115.3 (d, *J* = 21.4 Hz), 91.5, 82.5, 45.8, 39.7, 39.4, 37.2, 36.6, 27.9, 23.8, 23.8. ^{19}F NMR (376 MHz, Chloroform-*d*) δ -115.6. HRMS (ESI) calculated for $[C_{18}H_{23}NaFO]$ $[M+Na]^+$: 297.1625 found: 297.1613. Enantiomeric excess was determined by HPLC with a Chiralpak OJH column (hexanes: 2-propanol = 99: 1, 0.5 mL/min, 254 nm); major enantiomer tr = 13.48 min, minor enantiomer tr = 8.69 min.

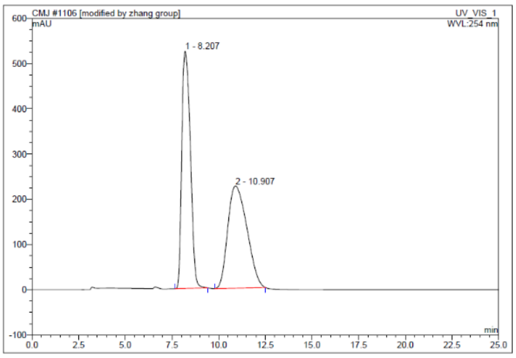

No.	Ret.Time min	Peak Name	Height mAU	Area mAU*min	Rel.Area %	Amount	Type
1	8.21	n.a.	524.517	278.913	50.91	n.a.	BMB*
2	10.91	n.a.	226.044	268.966	49.09	n.a.	BMB*
Total:			750.560	547.879	100.00	0.000	

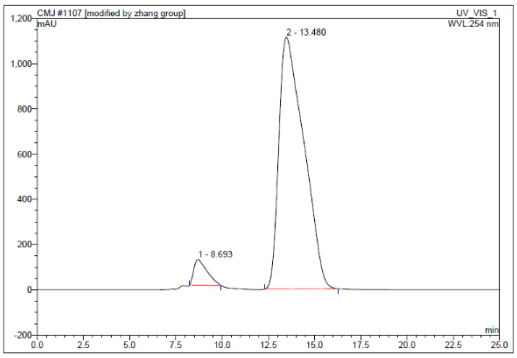

No.	Ret.Time min	Peak Name	Height mAU	Area mAU*min	Rel.Area %	Amount	Type
1	8.69	n.a.	113.657	93.937	5.11	n.a.	BMB*
2	13.48	n.a.	1113.249	1744.306	94.89	n.a.	BMB*
Total:			1226.907	1838.243	100.00	0.000	

4.1.37 (*S*, *E*)-2-methyl-2-(3-(naphthalen-1-yl)-allyl)-1-oxaspiro[4.4]nonane (6f)

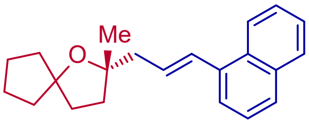

6f

Prepared according to typical procedure at RT for 72 hours by using $[Pd(allyl)Cl]_2$ (2.5 mol%), **Xu8** (10 mol%) from γ-hydroxyalkenes (0.2 mmol) and alkenyl bromide (0.4 mmol), after flash column chromatography on a silica gel (petroleum ether: ethyl acetate = 80: 1) give the product **6f** as a white liquid (59 mg, 95% yield) with 91% *ee*. $[\alpha]_D^{20}$= -12.2 (*c* = 0.4, Chloroform). ^{1}H NMR (400 MHz, Chloroform-*d*) δ 8.16 (d, *J* = 7.6 Hz, 1H), 7.86 (dd, *J* = 7.6, 2.4 Hz, 1H), 7.77 (d, *J* = 8.0 Hz, 1H), 7.60 (d, *J* = 7.2 Hz, 1H), 7.55 – 7.44 (m, 3H), 7.18 (d, *J* = 15.6 Hz, 1H), 6.33 – 6.26 (m, 1H), 2.56 (d, *J* = 7.2 Hz, 2H), 2.08 – 1.99 (m, 1H), 1.96 – 1.92 (m, 2H), 1.87 – 1.76 (m, 5H), 1.67 – 1.55 (m, 4H), 1.34 (s, 3H). ^{13}C NMR (100 MHz, Chloroform-*d*) δ 135.5, 133.6, 131.1, 130.4, 129.5, 128.4, 127.3, 125.8, 125.6, 125.6, 123.9, 123.5, 91.5, 82.6, 46.3, 39.8, 39.5, 37.3, 36.6, 28.0, 23.9, 23.8. HRMS (ESI) calculated for $[C_{22}H_{26}NaO]$ $[M+Na]^+$: 329.1876 found: 329.1878. Enantiomeric excess was determined by HPLC with a Chiralpak OJH+OJ3 column (hexanes: 2-propanol = 99: 1, 0.5 mL/min, 210 nm); major enantiomer tr = 29.83 min, minor enantiomer tr = 26.73 min.

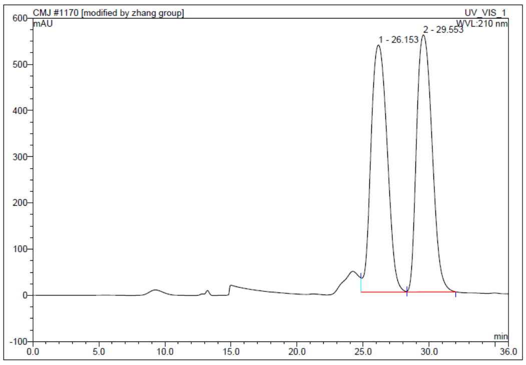

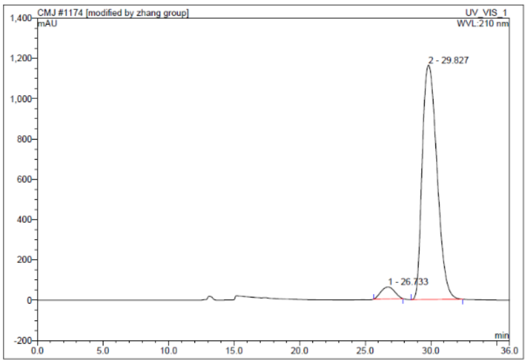

No.	Ret.Time min	Peak Name	Height mAU	Area mAU*min	Rel.Area %	Amount	Type
1	26.15	n.a.	534.916	761.249	50.16	n.a.	M *
2	29.55	n.a.	556.323	756.381	49.84	n.a.	MB*
Total:			1091.240	1517.631	100.00	0.000	

No.	Ret.Time min	Peak Name	Height mAU	Area mAU*min	Rel.Area %	Amount	Type
1	26.73	n.a.	59.608	72.137	4.54	n.a.	BMB*
2	29.83	n.a.	1163.639	1516.278	95.46	n.a.	BMB*
Total:			1223.247	1588.416	100.00	0.000	

4.1.38 (*S*, *E*)-2-methyl-2-(3-(naphthalen-2-yl)-allyl)-1-oxaspiro[4.4]nonane (6g)

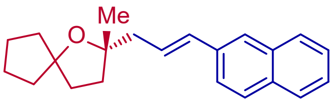

6g

Prepared according to typical procedure at RT for 72 hours by using [Pd(allyl)Cl]$_2$ (2.5 mol%), **Xu8** (10 mol%) from γ-hydroxyalkenes (0.2 mmol) and alkenyl bromide (0.4 mmol), after flash column chromatography on a silica gel (petroleum ether: ethyl acetate = 80: 1) give the product **6g** as a white liquid (58 mg, 95% yield) with 87% *ee*. $[\alpha]_D^{20}$= -7.8 (*c* = 0.4, Chloroform). ^{1}H NMR (400 MHz, Chloroform-*d*) δ 7.81 – 7.73 (m, 3H), 7.71 (s, 1H), 7.62 (d, *J* = 8.8 Hz, 1H), 7.48 – 7.41 (m, 2H), 6.59 (d, *J* = 16.0 Hz, 1H), 6.44 – 6.37 (m, 1H), 2.48 (d, *J* = 7.2 Hz, 2H), 2.07 – 1.98 (m, 1H), 1.94 – 1.90 (m, 2H), 1.87 – 1.76 (m, 5H), 1.66 – 1.55 (m, 4H), 1.31 (s, 3H). ^{13}C NMR (100 MHz, Chloroform-*d*) δ 135.2, 133.6, 132.7, 132.4, 128.0, 127.8, 127.6, 127.6, 126.1, 125.5, 125.4, 123.6, 91.5, 82.7, 46.0, 39.7, 39.4, 37.2, 36.6, 28.0, 23.9, 23.8. HRMS (ESI) calculated for [$C_{22}H_{26}NaO$] $[M+Na]^+$: 329.1876 found: 329.1872. Enantiomeric excess was determined by HPLC with a Chiralpak OJH+OJH column (hexanes: 2-propanol = 99: 1, 0.5 mL/min, 254 nm); major enantiomer tr = 50.95 min, minor enantiomer tr = 47.92 min.

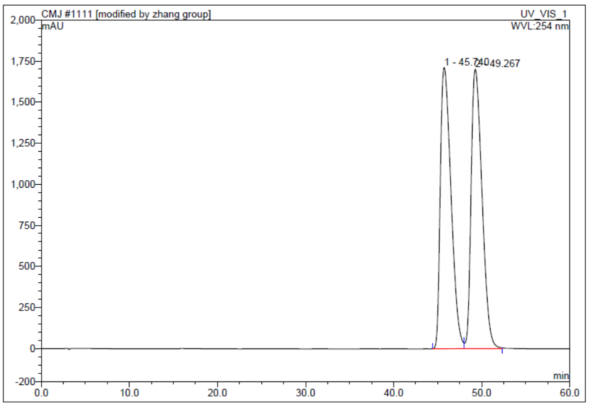

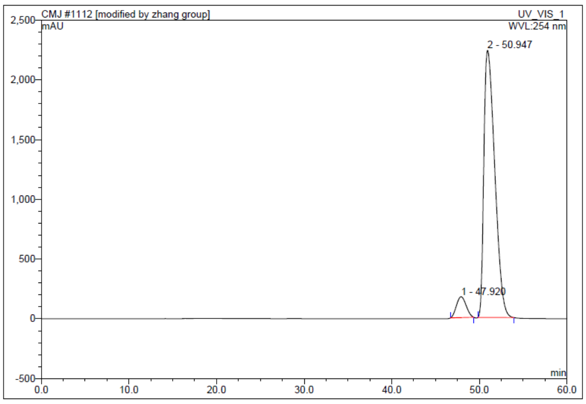

No.	Ret.Time min	Peak Name	Height mAU	Area mAU*min	Rel.Area %	Amount	Type
1	45.74	n.a.	1711.146	2405.939	49.63	n.a.	BM *
2	49.27	n.a.	1700.048	2441.483	50.37	n.a.	M *
Total:			3411.194	4847.422	100.00	0.000	

No.	Ret.Time min	Peak Name	Height mAU	Area mAU*min	Rel.Area %	Amount	Type
1	47.92	n.a.	175.122	223.399	6.49	n.a.	BMB*
2	50.95	n.a.	2235.016	3219.556	93.51	n.a.	BMB*
Total:			2410.137	3442.955	100.00	0.000	

4.1.39 (*S*)-2-methyl-2-((2*E*, 4*E*)-5-phenylpenta-2,4-dien-1-yl)-1-oxaspiro[4.4]nonane (6h)

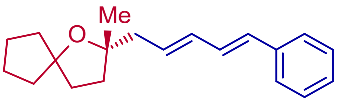

6h

Prepared according to typical procedure at RT for 72 hours by using $[Pd(allyl)Cl]_2$ (2.5 mol%), **Xu8** (10 mol%) from γ-hydroxyalkenes (0.2 mmol) and alkenyl bromide (0.4 mmol), after flash column chromatography on a silica gel (petroleum ether: ethyl acetate = 80: 1) give the product **6h** as a white liquid (57 mg, 96% yield) with 92% *ee*. $[\alpha]_D^{20}$ = -13.1 (*c* = 0.4, Chloroform). ^{1}H NMR (400 MHz, Chloroform-*d*) δ 7.40 – 7.38 (m, 2H), 7.33 – 7.29 (m, 2H), 7.23 – 7.18 (m, 1H), 6.79 (dd, *J* = 15.6, 10.4 Hz, 1H), 6.47 (d, *J* = 15.6 Hz, 1H), 6.24 (dd, *J* = 15.2, 10.4 Hz, 1H), 5.90 – 5.82 (m, 1H), 2.35 (d, *J* = 7.6 Hz, 2H), 1.93 – 1.87 (m, 3H), 1.81 – 1.71 (m, 5H), 1.64 – 1.50 (m, 4H), 1.24 (s, 3H). ^{13}C NMR (100 MHz, Chloroform-*d*) δ 137.5, 133.0, 131.8, 130.4, 129.3, 128.5, 127.1, 126.1, 91.4, 82.6, 45.8, 39.7, 39.4, 37.2, 36.7, 27.8, 23.9, 23.8. HRMS (ESI) calculated for $[C_{20}H_{26}NaO]$ $[M+Na]^+$: 305.1876 found: 305.1874. Enantiomeric excess was determined by HPLC with a Chiralpak OJH+OJ3 column (hexanes: 2-propanol = 99: 1, 0.5 mL/min, 254 nm); major enantiomer tr = 30.16 min, minor enantiomer tr = 28.13 min.

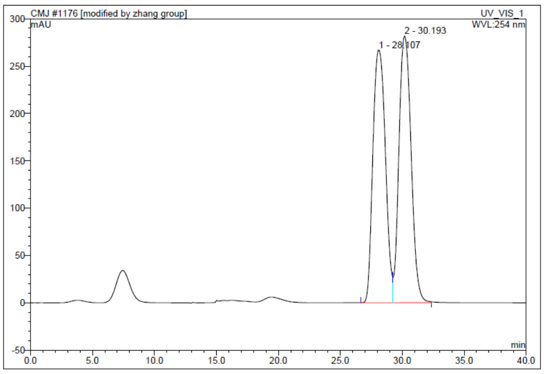

No.	Ret.Time min	Peak Name	Height mAU	Area mAU*min	Rel.Area %	Amount	Type
1	28.11	n.a.	267.061	312.245	49.30	n.a.	BM *
2	30.19	n.a.	281.624	321.071	50.70	n.a.	M *
Total:			548.684	633.316	100.00	0.000	

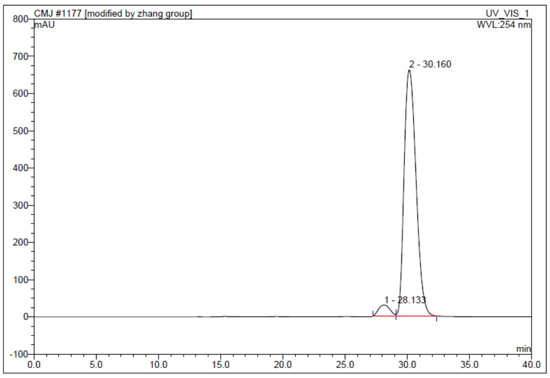

No.	Ret.Time min	Peak Name	Height mAU	Area mAU*min	Rel.Area %	Amount	Type
1	28.13	n.a.	29.427	31.134	4.07	n.a.	BM *
2	30.16	n.a.	661.171	733.290	95.93	n.a.	MB*
Total:			690.599	764.424	100.00	0.000	

4.2 General Procedure for reactions of 4-penten-1-ol with alkenyl bromides

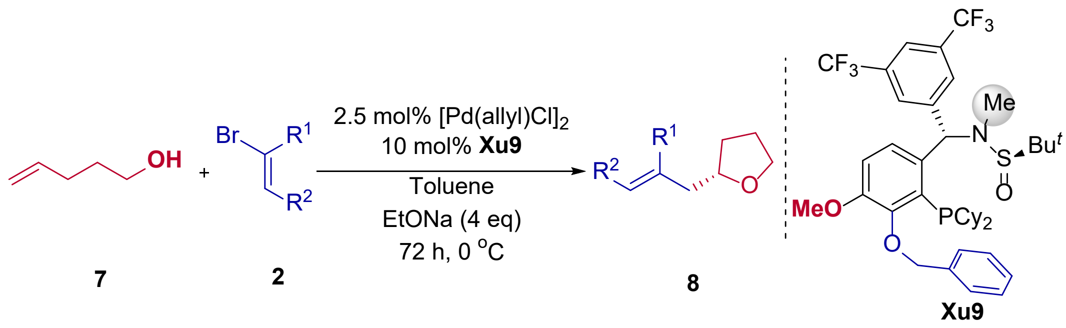

To a sealed tube was added $[Pd(allyl)Cl]_2$ (2.5 mol%), **Xu9** (10 mol%). The flask was evacuated and refilled with argon. Then γ-hydroxyalkenes (0.5 mmol), alkenyl halides (1 mmol), EtONa (4.0 equiv.), and solution of toluene (3.5 mL) was added to the tube, and stirred at 0 °C for 72 hours. After the reaction was complete (monitored by TLC), solvent was removed under reduced pressure. The crude product was then purified by flash column chromatography on silica gel to afford the desired product.

4.2.1 Synthesis of (*S*, *E*)-2-(3-(4-methoxyphenyl)allyl)tetrahydrofuran (8a)

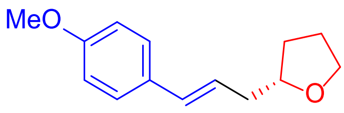

8a

Prepared according to typical procedure at 0 °C for 72 hours by using $[Pd(allyl)Cl]_2$ (2.5 mol%), **Xu9** (10 mol%) from γ-hydroxyalkenes (0.5 mmol) and alkenyl bromide (1 mmol), after flash column chromatography on a silica gel (petroleum ether: ethyl acetate = 50: 1) give the product **8a** as a white liquid (74 mg, 68% yield) with 92% ee. $[\alpha]_D^{20}$ = -2.4 (*c* = 0.4, Chloroform).^{1}H NMR (400 MHz, Chloroform-*d*) δ 7.35 – 7.23 (m, 2H), 6.87 – 6.79 (m, 2H), 6.40 (d, *J* = 15.8 Hz, 1H), 6.09 (dt,

J = 15.7, 7.1 Hz, 1H), 4.02 – 3.84 (m, 2H), 3.79 (s, 3H), 3.74 (q, J = 7.4 Hz, 1H), 2.53 – 2.42 (m, 1H), 2.43 – 2.31 (m, 1H), 1.99 (tdd, J = 11.4, 7.0, 4.8 Hz, 1H), 1.89 (ddd, J = 12.3, 7.8, 6.2 Hz, 2H), 1.56 (dq, J = 11.7, 7.8 Hz, 1H). ^{13}C NMR (100 MHz, Chloroform-*d*) δ 158.8, 131.3, 130.4, 127.2, 124. 6, 113.9, 78.9, 68.0, 55.3, 39.3, 30.9, 25.8. HRMS (ESI) calculated for [$C_{14}H_{18}NaO_2$] $[M+Na]^+$: 241.1199 found: 241.1199. Enantiomeric excess was determined by HPLC with a Chiralpak OD-H column (hexanes: 2-propanol = 97: 3, 0.6 mL/min, 254 nm); major enantiomer tr = 15.290 min, minor enantiomer tr = 13.051 min.

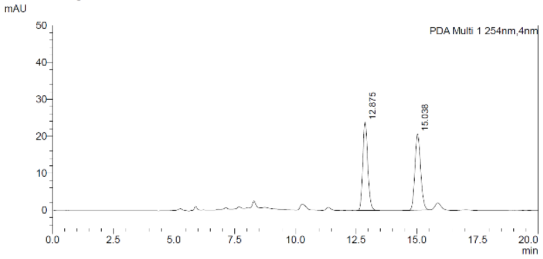

<Peak Table>
PDA Ch1 254nm

Peak#	Ret. Time	Height	Height%	Area	Area%
1	12.875	23959	53.696	341864	50.130
2	15.038	20660	46.304	340093	49.870
Total		44619	100.000	681957	100.000

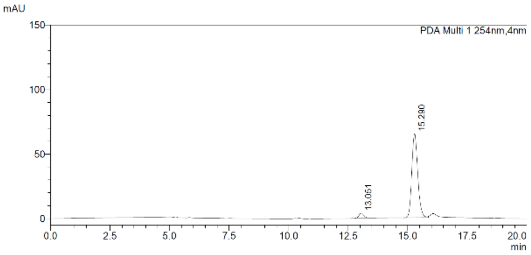

<Peak Table>
PDA Ch1 254nm

Peak#	Ret. Time	Height	Height%	Area	Area%
1	13.051	3559	5.198	48367	4.113
2	15.290	64906	94.802	1127608	95.887
Total		68465	100.000	1175975	100.000

4.2.2 Synthesis of (*S*)-2-cinnamyltetrahydrofuran (8b)

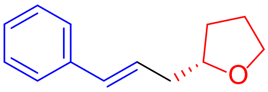

8b

Prepared according to typical procedure at 0 °C for 72 hours by using $[Pd(allyl)Cl]_2$ (2.5 mol%), **Xu9** (10 mol%) from γ-hydroxyalkenes (0.5 mmol) and alkenyl bromide (1 mmol), after flash column chromatography on a silica gel (petroleum ether: ethyl acetate = 50: 1) give the product **8b** as a white liquid (69 mg, 73% yield) with 92% ee. $[\alpha]_D^{20}$ = 1.3 (c = 0.4, Chloroform). ^{1}H NMR (400 MHz, Chloroform-*d*)δ 7.35 (d, J = 7.2 Hz, 2H), 7.28 (t, J = 7.5 Hz, 2H), 7.19 (t, J = 7.2 Hz, 1H), 6.45 (d, J = 15.8 Hz, 1H), 6.24 (dt, J = 15.8, 7.1 Hz, 1H), 4.05 – 3.85 (m, 2H), 3.82 – 3.68 (m, 1H), 2.57 – 2.44 (m, 1H), 2.45 – 2.32 (m, 1H), 1.98 (tdd, J = 9.3, 7.7, 4.3 Hz, 1H), 1.89 (ddd, J = 12.1, 7.9, 6.2 Hz, 2H), 1.63 – 1.46 (m, 1H). ^{13}C NMR (101 MHz, Chloroform-*d*)δ 137.5, 131.9, 128.4, 127.0, 126.7, 126.0, 78.7, 67.9, 39.2, 30.8, 25.7. HRMS (ESI) calculated for [$C_{13}H_{17}O$] $[M+H]^+$: 189.1274 found: 189.1278. Enantiomeric excess was determined by HPLC with a Chiralpak OD-H column (hexanes: 2-propanol = 95: 5, 0.6 mL/min, 254 nm); major enantiomer tr = 12.572 min, minor enantiomer tr = 10.400 min.

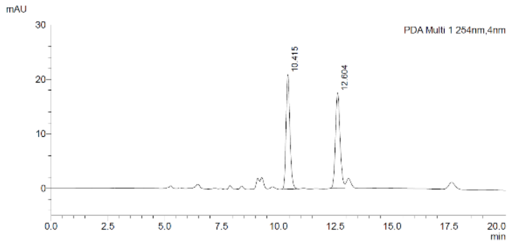

<Peak Table>
PDA Ch1 254nm

Peak#	Ret. Time	Height	Height%	Area	Area%
1	10.415	20994	54.535	247505	50.055
2	12.604	17503	45.465	246962	49.945
Total		38497	100.000	494466	100.000

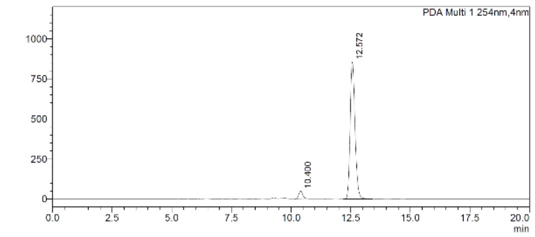

<Peak Table>
PDA Ch1 254nm

Peak#	Ret. Time	Height	Height%	Area	Area%
1	10.400	47743	5.284	529798	4.163
2	12.572	855751	94.716	12195728	95.837
Total		903495	100.000	12725526	100.000

4.2.3 Synthesis of (*S*, *E*)-2-(3-(p-tolyl)allyl)tetrahydrofuran (8c)

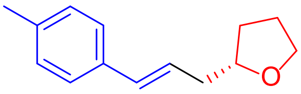

8c

Prepared according to typical procedure at 0 ºC for 72 hours by using [Pd(allyl)Cl]$_2$ (2.5 mol%), **Xu9** (10 mol%) from γ-hydroxyalkenes (0.5 mmol) and alkenyl bromide (1 mmol), after flash column chromatography on a silica gel (petroleum ether: ethyl acetate = 50: 1) give the product **8c** as a white liquid (65 mg, 64% yield) with 92% ee. $[\alpha]_D^{20}$ = -3.8 (*c* = 0.4, Chloroform). ^{1}H NMR (400 MHz, Chloroform-*d*) δ 7.24 (d, *J* = 7.9 Hz, 2H), 7.09 (d, *J* = 7.9 Hz, 2H), 6.41 (d, *J* = 15.8 Hz, 1H), 6.17 (dt, *J* = 15.8, 7.1 Hz, 1H), 4.00 – 3.84 (m, 2H), 3.79 – 3.69 (m, 1H), 2.54 – 2.43 (m, 1H), 2.45 – 2.33 (m, 1H), 2.31 (s, 3H), 2.06 – 1.93 (m, 1H), 1.93 – 1.79 (m, 2H), 1.55 (dq, *J* = 11.7, 7.8 Hz, 1H). ^{13}C NMR (100 MHz, Chloroform-*d*) δ 136.6, 134.7, 131.7, 129.1, 125.9, 125.6, 78.8, 67.9, 39.2, 30.7, 25.7, 21.0. HRMS (ESI) calculated for [$C_{22}H_{26}NaO$] [M+Na]$^+$: 225.1250 found: 225.1245. Enantiomeric excess was determined by HPLC with a Chiralpak OD-H column (hexanes: 2-propanol = 95: 5, 0.6 mL/min, 254 nm); major enantiomer tr = 13.659 min, minor enantiomer tr = 9.569 min.

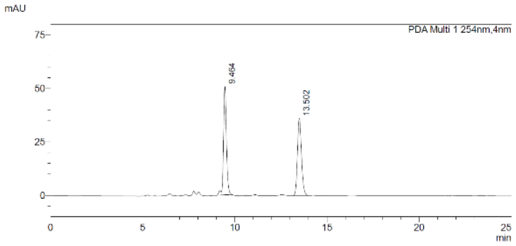

<Peak Table>
PDA Ch1 254nm

Peak#	Ret. Time	Height	Height%	Area	Area%
1	9.464	50684	58.462	550763	50.195
2	13.502	36011	41.538	546479	49.805
Total		86695	100.000	1097242	100.000

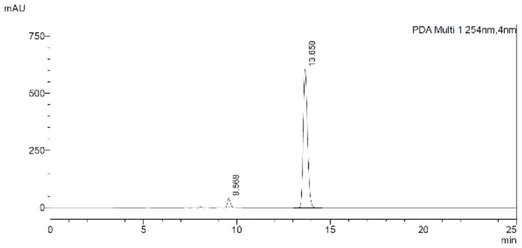

<Peak Table>
PDA Ch1 254nm

Peak#	Ret. Time	Height	Height%	Area	Area%
1	9.569	39713	6.153	410947	4.106
2	13.659	605713	93.847	9598708	95.894
Total		645425	100.000	10009655	100.000

4.2.4 Synthesis of (*S*, *E*)-2-(3-(4-isopropylphenyl)allyl)tetrahydrofuran (8d)

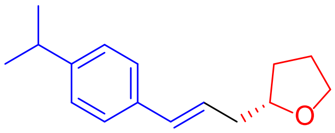

8d

Prepared according to typical procedure at 0 °C for 72 hours by using [Pd(allyl)Cl]$_2$ (2.5 mol%), **Xu9** (10 mol%) from γ-hydroxyalkenes (0.5 mmol) and alkenyl bromide (1 mmol), after flash column chromatography on a silica gel (petroleum ether: ethyl acetate = 50: 1) give the product **8d** as a white liquid (62 mg, 54% yield) with 94% ee. $[\alpha]_D^{20}$ = -5.9 (*c* = 0.4, Chloroform). ^{1}H NMR (400 MHz, Chloroform-*d*) δ 7.29 (d, *J* = 8.0 Hz, 2H), 7.15 (d, *J* = 8.0 Hz, 2H), 6.42 (d, *J* = 15.8 Hz, 1H), 6.18 (dt, *J* = 15.8, 7.1 Hz, 1H), 4.01 – 3.82 (m, 2H), 3.82 – 3.67 (m, 1H), 2.87 (p, *J* = 6.9 Hz, 1H), 2.58 – 2.42 (m, 1H), 2.43 – 2.32 (m, 1H), 2.02 – 1.77 (m, 3H), 1.61 – 1.47 (m, 1H), 1.23 (d, *J* = 6.9 Hz, 6H). ^{13}C NMR (100 MHz, Chloroform-*d*) δ 147.8, 135.2, 131.8, 126.6, 126.1, 125.8, 78.9, 68.0, 39.3, 33.9, 30.8, 25.8, 24.0. HRMS (ESI) calculated for [$C_{16}H_{22}NaO$] [M+Na]$^+$: 253.1563 found: 253.1565. Enantiomeric excess was determined by HPLC with a Chiralpak OD-H column (hexanes: 2-propanol = 95: 5, 0.6 mL/min, 254 nm); major enantiomer tr = 9.225 min, minor enantiomer tr = 7.976 min.

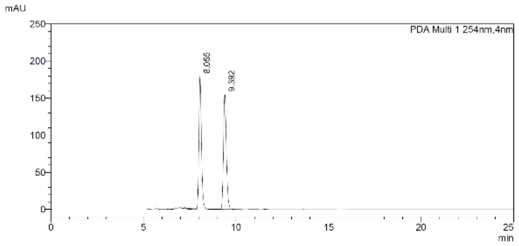

<Peak Table>
PDA Ch1 254nm

Peak#	Ret. Time	Height	Height%	Area	Area%
1	8.055	179306	53.561	1665650	49.220
2	9.392	155465	46.439	1718466	50.780
Total		334771	100.000	3384116	100.000

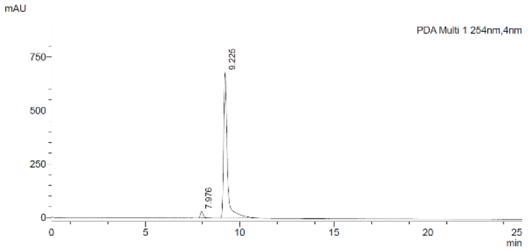

<Peak Table>
PDA Ch1 254nm

Peak#	Ret. Time	Height	Height%	Area	Area%
1	7.976	30472	4.290	307962	3.214
2	9.225	679861	95.710	9274605	96.786
Total		710333	100.000	9582567	100.000

4.2.5 Synthesis of (*S*, *E*)-2-(3-(4-(tert-butyl)phenyl)allyl)tetrahydrofuran (8e)

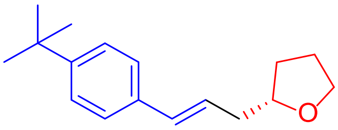

8e

Prepared according to typical procedure at 0 °C for 72 hours by using [Pd(allyl)Cl]$_2$ (2.5 mol%), **Xu9** (10 mol%) from γ-hydroxyalkenes (0.5 mmol) and alkenyl bromide (1 mmol), after flash column chromatography on a silica gel (petroleum ether: ethyl acetate = 50: 1) give the product **8e** as a white liquid (82 mg, 67% yield) with 93% ee. $[\alpha]_D^{20}$ = -7.5 (*c* = 0.4, Chloroform). ^{1}H NMR (400 MHz, Chloroform-*d*) δ 7.36 – 7.23 (m, 4H), 6.43 (d, *J* = 15.8 Hz, 1H), 6.19 (dt, *J* = 15.7, 7.1 Hz,

1H), 4.03 – 3.82 (m, 2H), 3.73 (td, *J* = 7.9, 6.6 Hz, 1H), 2.56 – 2.31 (m, 2H), 2.02 – 1.77 (m, 3H), 1.62 – 1.48 (m, 1H), 1.30 (s, 9H). ^{13}C NMR (100 MHz, Chloroform-*d*) δ 150.1, 134.8, 131.7, 125.9, 125.8, 125.4, 78.9, 68.0, 39.3, 34. 6, 31.4, 30.8, 25.8. HRMS (ESI) calculated for [$C_{17}H_{24}NaO$] [M+Na]$^+$: 267.1719 found: 267.1726. Enantiomeric excess was determined by HPLC with a Chiralpak OD-H column (hexanes: 2-propanol = 95: 5, 0.6 mL/min, 254 nm); major enantiomer tr = 7.851 min, minor enantiomer tr = 7.371 min..

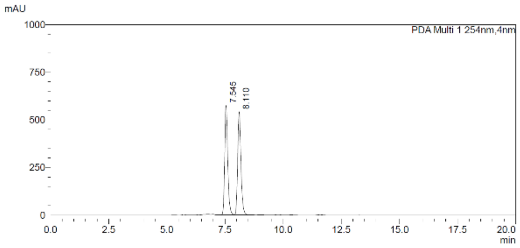

<Peak Table>

PDA Ch1 254nm

Peak#	Ret. Time	Height	Height%	Area	Area%
1	7.545	575694	51.498	5301871	50.000
2	8.110	542194	48.502	5301814	50.000
Total		1117888	100.000	10603685	100.000

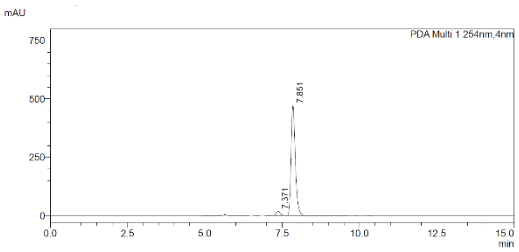

<Peak Table>

PDA Ch1 254nm

Peak#	Ret. Time	Height	Height%	Area	Area%
1	7.371	18870	3.851	165540	3.567
2	7.851	471087	96.149	4475553	96.433
Total		489957	100.000	4641092	100.000

4.2.6 Synthesis of (*S*, *E*)-2-(3-([1,1'-biphenyl]-4-yl)allyl)tetrahydrofuran (8f)

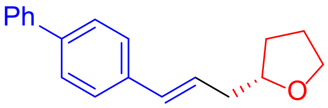

8f

Prepared according to typical procedure at 0 °C for 72 hours by using [Pd(allyl)Cl]$_2$ (2.5 mol%), **Xu9** (10 mol%) from γ-hydroxyalkenes (0.5 mmol) and alkenyl bromide (1 mmol), after flash column chromatography on a silica gel (petroleum ether: ethyl acetate = 50: 1) give the product **8f** as a white solid (70 mg, 53% yield) with 92% ee. Mp: 79.5 – 83.1 °C. $[\alpha]_D^{20}$ = 0.9 (*c* = 0.4, Chloroform).^{1}H NMR (400 MHz, Chloroform-*d*) δ 7.62 – 7.55 (m, 2H), 7.55 – 7.49 (m, 2H), 7.41 (dt, *J* = 7.8, 3.6 Hz, 4H), 7.36 – 7.26 (m, 1H), 6.54 – 6.43 (m, 1H), 6.28 (dt, *J* = 15.8, 7.1 Hz, 1H), 4.04 – 3.84 (m, 2H), 3.74 (td, *J* = 7.8, 6.3 Hz, 1H), 2.51 (dtd, *J* = 14.8, 6.6, 1.4 Hz, 1H), 2.46 – 2.36 (m, 1H), 1.98 (dddd, *J* = 11.3, 8.3, 6.3, 4.8 Hz, 1H), 1.92 – 1.81 (m, 2H), 1.56 (ddt, *J* = 11.5, 8.4, 7.4 Hz, 1H). ^{13}C NMR (100 MHz, Chloroform-*d*) δ 140.8, 139.8, 136.7, 131.5, 128.8, 127.2, 127.2, 127.0, 126.9, 126.6, 78.8, 68.1, 39.4, 30.9, 25.8. HRMS (ESI) calculated for [$C_{19}H_{20}NaO$] [M+Na]$^+$: 287.1406 found: 287.1401. Enantiomeric excess was determined by HPLC with a Chiralpak OD-H column (hexanes: 2-propanol = 95: 5, 0.6 mL/min, 254 nm); major enantiomer tr = 12.804 min, minor enantiomer tr = 11.448 min.

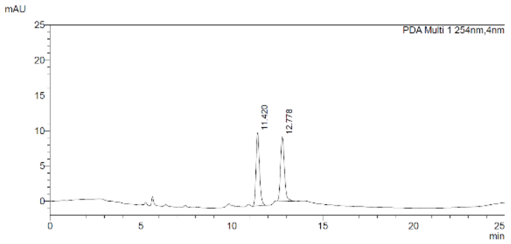

<Peak Table>
PDA Ch1 254nm

Peak#	Ret. Time	Height	Height%	Area	Area%
1	11.420	10350	53.281	137788	50.737
2	12.778	9076	46.719	133784	49.263
Total		19426	100.000	271573	100.000

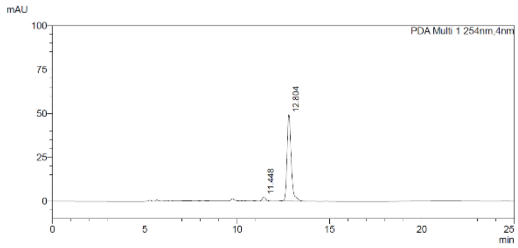

<Peak Table>
PDA Ch1 254nm

Peak#	Ret. Time	Height	Height%	Area	Area%
1	11.448	2358	4.573	30827	3.872
2	12.804	49212	95.427	765287	96.128
Total		51570	100.000	796114	100.000

4.2.7 Synthesis of (*S*, *E*)-2-(3-(2,4-dimethylphenyl)allyl)tetrahydrofuran (8g)

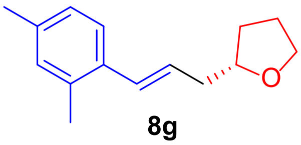

8g

Prepared according to typical procedure at 0 °C for 72 hours by using $[Pd(allyl)Cl]_2$ (2.5 mol%), **Xu9** (10 mol%) from γ-hydroxyalkenes (0.5 mmol) and alkenyl bromide (1 mmol), after flash column chromatography on a silica gel (petroleum ether: ethyl acetate = 50: 1) give the product **8g** as a white liquid (62 mg, 57% yield) with 93% ee. $[\alpha]_D^{20}$ = -7.6 (*c* = 0.4, Chloroform).^{1}H NMR (400 MHz, Chloroform-*d*) δ 7.32 (d, *J* = 7.6 Hz, 1H), 6.94 (d, *J* = 7.7 Hz, 2H), 6.61 (d, *J* = 15.7 Hz, 1H), 6.05 (dt, *J* = 15.7, 7.2 Hz, 1H), 4.02 – 3.83 (m, 2H), 3.74 (td, *J* = 7.9, 6.5 Hz, 1H), 2.52 (dddd, *J* = 13.2, 7.2, 5.9, 1.4 Hz, 1H), 2.46 – 2.35 (m, 1H), 2.29 (d, *J* = 5.1 Hz, 6H), 2.08 – 1.82 (m, 3H), 1.63 – 1.47 (m, 1H). ^{13}C NMR (100 MHz, Chloroform-*d*) δ 136.5, 134.7, 133.7, 130.8, 129.5, 127.0, 126.6, 125.3, 78.8, 67.9, 39.4, 30.7, 25.7, 21.0, 19.7. HRMS (ESI) calculated for $[C_{15}H_{20}NaO]$ $[M+Na]^+$: 239.1406 found: 239.1409. Enantiomeric excess was determined by HPLC with a Chiralpak OD-H column (hexanes: 2-propanol = 95: 5, 0.6 mL/min, 254 nm); major enantiomer tr = 20.085 min, minor enantiomer tr = 10.095 min.

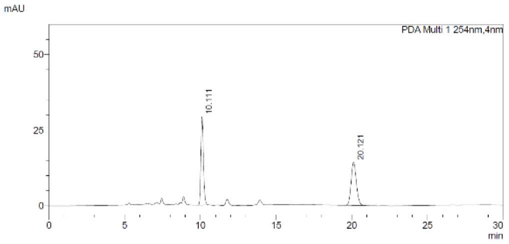

<Peak Table>
PDA Ch1 254nm

Peak#	Ret. Time	Height	Height%	Area	Area%
1	10.111	29278	67.233	340671	49.942
2	20.121	14269	32.767	341459	50.058
Total		43547	100.000	682129	100.000

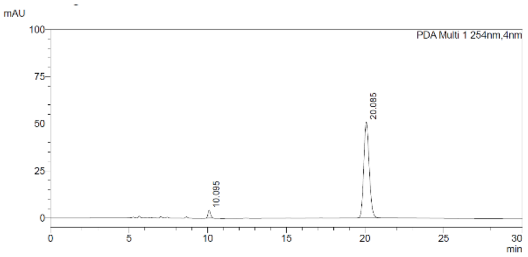

<Peak Table>
PDA Ch1 254nm

Peak#	Ret. Time	Height	Height%	Area	Area%
1	10.095	4190	7.603	45663	3.610
2	20.085	50923	92.397	1219312	96.390
Total		55113	100.000	1264975	100.000

4.2.8 Synthesis of (*S*, *E*)-2-(3-(4-(benzyloxy)phenyl)allyl)tetrahydrofuran (8h)

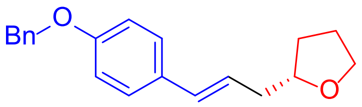

8h

Prepared according to typical procedure at 0 °C for 72 hours by using $[Pd(allyl)Cl]_2$ (2.5 mol%), **Xu9** (10 mol%) from γ-hydroxyalkenes (0.5 mmol) and alkenyl bromide (1 mmol), after flash column chromatography on a silica gel (petroleum ether: ethyl acetate = 50: 1) give the product **8h** as a white solid (83 mg, 56% yield) with 91% ee. Mp: 40.0 – 41.5 °C. $[\alpha]_D^{20}$ = -2.4 (c = 0.4, Chloroform). ^{1}H NMR (400 MHz, Chloroform-d) δ 7.44 – 7.24 (m, 7H), 6.89 (d, J = 8.5 Hz, 2H), 6.39 (d, J = 15.8 Hz, 1H), 6.09 (dt, J = 15.6, 7.1 Hz, 1H), 5.03 (s, 2H), 3.99 – 3.83 (m, 2H), 3.81 – 3.67 (m, 1H), 2.52 – 2.42 (m, 1H), 2.42 – 2.31 (m, 1H), 2.04 – 1.80 (m, 3H), 1.54 (dq, J = 11.7, 7.8 Hz, 1H). ^{13}C NMR (100 MHz, Chloroform-d) δ 158.0, 137.0, 131.3, 130.7, 128.6, 128.0, 127.5, 127.2, 124.7, 114.9, 79.0, 70.0, 68.0, 39.3, 30.9, 25.8. HRMS (ESI) calculated for $[C_{20}H_{22}NaO_2]$ $[M+Na]^+$: 317.1512 found: 317.1509. Enantiomeric excess was determined by HPLC with a Chiralpak OD-H column (hexanes: 2-propanol = 95: 5, 0.6 mL/min, 254 nm); major enantiomer tr = 21.576 min, minor enantiomer tr = 17.993 min.

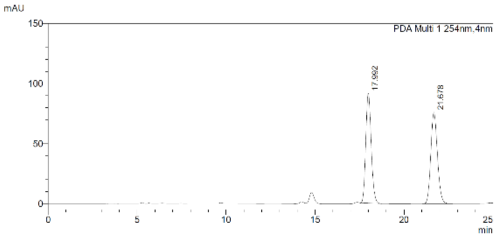

<Peak Table>

PDA Ch1 254nm

Peak#	Ret. Time	Height	Height%	Area	Area%
1	17.992	91554	54.606	1944284	49.671
2	21.678	76109	45.394	1970032	50.329
Total		167664	100.000	3914316	100.000

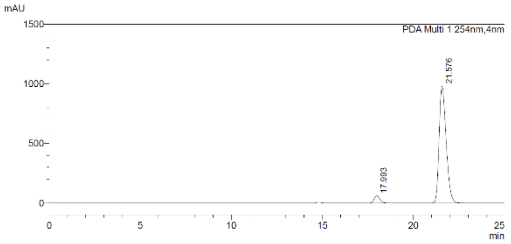

<Peak Table>

PDA Ch1 254nm

Peak#	Ret. Time	Height	Height%	Area	Area%
1	17.993	61617	5.899	1281764	4.656
2	21.576	982939	94.101	26247948	95.344
Total		1044557	100.000	27529713	100.000

4.2.9 Synthesis of (*S*, *E*)-2-(3-(2-methoxyphenyl)allyl)tetrahydrofuran (8i)

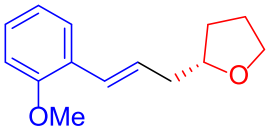

8i

Prepared according to typical procedure at 0 °C for 72 hours by using $[Pd(allyl)Cl]_2$ (2.5 mol%), **Xu9** (10 mol%) from γ-hydroxyalkenes (0.5 mmol) and alkenyl bromide (1 mmol), after flash column chromatography on a silica gel (petroleum ether: ethyl acetate = 50: 1) give the product **8i** as a white liquid (70 mg, 64% yield) with 92% ee. $[\alpha]_D^{20}$ = -4.5 (c = 0.4, Chloroform). ^{1}H NMR (400 MHz, Chloroform-d) δ 7.43 (dd, J = 7.6, 1.7 Hz, 1H), 7.18 (ddd, J = 8.1, 7.4, 1.7 Hz, 1H), 6.90 (td,

J = 7.5, 1.0 Hz, 1H), 6.84 (dd, J = 8.2, 1.0 Hz, 1H), 6.78 (dd, J = 16.0, 1.6 Hz, 1H), 6.22 (dt, J = 15.9, 7.2 Hz, 1H), 4.01 – 3.86 (m, 2H), 3.83 (s, 3H), 3.74 (td, J = 7.9, 6.4 Hz, 1H), 2.53 (dddd, J = 14.6, 7.3, 6.1, 1.5 Hz, 1H), 2.41 (dddd, J = 13.9, 7.5, 6.5, 1.5 Hz, 1H), 2.07 – 1.79 (m, 3H), 1.63 – 1.54 (m, 1H). ^{13}C NMR (100 MHz, Chloroform-*d*) δ 156.3, 128.1, 127.4, 126.6, 126.5, 120.6, 110.7, 79.0, 68.0, 55.5, 39.7, 30.8, 25.8. HRMS (ESI) calculated for [$C_{14}H_{18}NaO_2$] [M+Na]$^+$: 241.1199 found: 241.1205. Enantiomeric excess was determined by HPLC with a Chiralpak ODH column (hexanes: 2-propanol = 95: 5, 0.6 mL/min, 254 nm); major enantiomer tr = 26.598 min, minor enantiomer tr = 21.757 min.

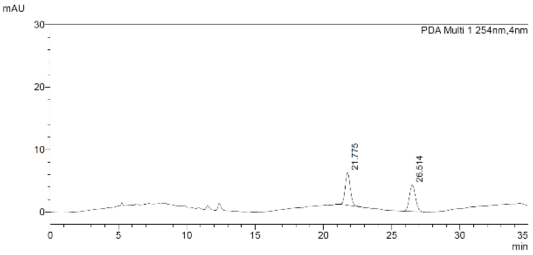

<Peak Table>
PDA Ch1 254nm

Peak#	Ret. Time	Height	Height%	Area	Area%
1	21.775	5229	55.025	137274	51.007
2	26.514	4274	44.975	131852	48.993
Total		9503	100.000	269126	100.000

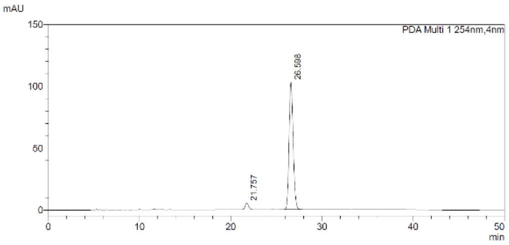

<Peak Table>
PDA Ch1 254nm

Peak#	Ret. Time	Height	Height%	Area	Area%
1	21.757	5427	5.005	137850	4.092
2	26.598	103014	94.995	3231245	95.908
Total		108441	100.000	3369094	100.000

4.2.10 Synthesis of (*S*,*E*)-5-(3-(tetrahydrofuran-2-yl)prop-1-en-1-yl)benzo[d][1,3]dioxole (8j)

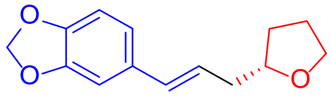

8j

Prepared according to typical procedure at 0 °C for 72 hours by using [Pd(allyl)Cl]$_2$ (2.5 mol%), **Xu9** (10 mol%) from γ-hydroxyalkenes (0.5 mmol) and alkenyl bromide (1 mmol), after flash column chromatography on a silica gel (petroleum ether: ethyl acetate = 50: 1) give the product **8j** as a white liquid (54 mg, 56% yield) with 94% ee. $[\alpha]_D^{20}$ = 0.1 (c = 0.4, Chloroform). ^{1}H NMR (400 MHz, Chloroform-*d*) δ 6.90 (d, J = 1.7 Hz, 1H), 6.82 – 6.67 (m, 2H), 6.35 (dt, J = 15.8, 1.4 Hz, 1H), 6.13 – 5.99 (m, 1H), 5.92 (s, 2H), 4.00 – 3.83 (m, 2H), 3.74 (td, J = 7.9, 6.5 Hz, 1H), 2.53 – 2.30 (m, 2H), 2.04 – 1.78 (m, 3H), 1.54 (ddt, J = 11.7, 8.4, 7.4 Hz, 1H). ^{13}C NMR (100 MHz, Chloroform-*d*) δ 147.8, 146.6, 132.0, 131.4, 124.9, 120.4, 108.1, 105.4, 100.9, 78.8, 67.9, 39.0, 30.8, 25.7. HRMS (ESI) calculated for [$C_{14}H_{16}NaO_3$] [M+Na]$^+$: 255.0992 found: 255.0989. Enantiomeric excess was determined by HPLC with a Chiralpak OD-H column (hexanes: 2-propanol = 95: 5, 0.6 mL/min, 254 nm); major enantiomer tr = 12.594 min, minor enantiomer tr = 11.869 min.

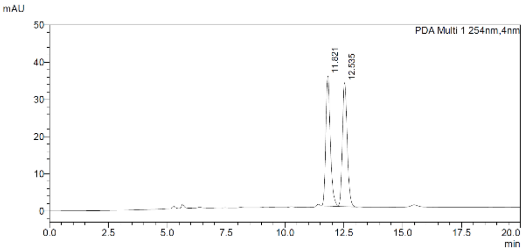

<Peak Table>
PDA Ch1 254nm

Peak#	Ret. Time	Height	Height%	Area	Area%
1	11.821	35024	51.244	473897	50.156
2	12.535	33324	48.756	470946	49.844
Total		68348	100.000	944843	100.000

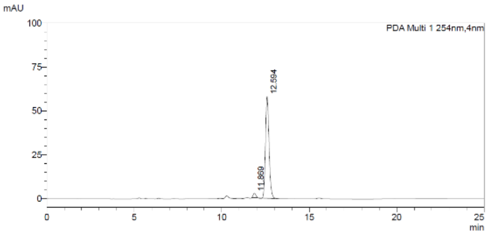

<Peak Table>
PDA Ch1 254nm

Peak#	Ret. Time	Height	Height%	Area	Area%
1	11.869	2512	4.152	27651	3.247
2	12.594	57995	95.848	823913	96.753
Total		60508	100.000	851564	100.000

4.2.11 Synthesis of (*S*, *E*)-2-(3-(3,4-dimethoxyphenyl)allyl)tetrahydrofuran (8k)

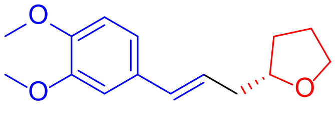

8k

Prepared according to typical procedure at 0 °C for 72 hours by using $[Pd(allyl)Cl]_2$ (2.5 mol%), **Xu9** (10 mol%) from γ-hydroxyalkenes (0.5 mmol) and alkenyl bromide (1 mmol), after flash column chromatography on a silica gel (petroleum ether: ethyl acetate = 50: 1) give the product **8k** as a white liquid (54 mg, 44% yield) with 91% ee. $[\alpha]_D^{20}$= 1.9 (*c* = 0.4, Chloroform).^{1}H NMR (400 MHz, Chloroform-*d*) δ 6.93 (d, *J* = 2.0 Hz, 1H), 6.88 (dd, *J* = 8.3, 2.0 Hz, 1H), 6.79 (d, *J* = 8.2 Hz, 1H), 6.39 (dt, *J* = 15.9, 1.4 Hz, 1H), 6.11 (dt, *J* = 15.8, 7.2 Hz, 1H), 3.99 – 3.90 (m, 2H), 3.89 (s, 3H), 3.86 (s, 3H), 3.75 (td, *J* = 7.9, 6.4 Hz, 1H), 2.55 – 2.30 (m, 2H), 2.00 (dddd, *J* = 11.3, 8.4, 6.3, 4.9 Hz, 1H), 1.95 – 1.84 (m, 2H), 1.57 (ddt, *J* = 11.6, 8.4, 7.4 Hz, 1H). ^{13}C NMR (100 MHz, Chloroform-*d*) δ 148.9, 148.4, 131.5, 130.7, 124.9, 119.1, 111.1, 108.5, 78.9, 68.0, 55.9, 55.8, 39.21, 30.9, 25.7. HRMS (ESI) calculated for $[C_{15}H_{20}NaO_3]$ $[M+Na]^+$: 271.1305 found: 271.1313. Enantiomeric excess was determined by HPLC with a Chiralpak OD-H column (hexanes: 2-propanol = 95: 5, 0.6 mL/min, 254 nm); major enantiomer tr = 21.788 min, minor enantiomer tr = 19.809 min.

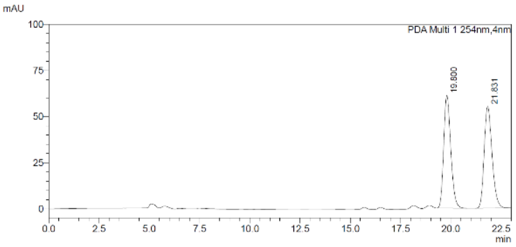

<Peak Table>
PDA Ch1 254nm

Peak#	Ret. Time	Height	Height%	Area	Area%
1	19.800	61010	52.511	1490991	49.757
2	21.831	55174	47.489	1505550	50.243
Total		116184	100.000	2996542	100.000

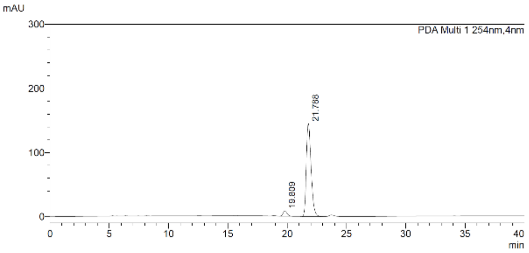

<Peak Table>
PDA Ch1 254nm

Peak#	Ret. Time	Height	Height%	Area	Area%
1	19.809	8113	5.302	191973	4.536
2	21.788	144910	94.698	4040662	95.464
Total		153023	100.000	4232635	100.000

4.2.12 Synthesis of (*S*, *E*)-2-(3-(3,4,5-trimethoxyphenyl)allyl)tetrahydrofuran (8l)

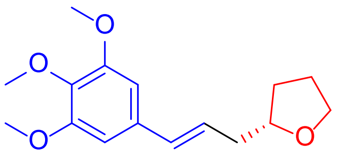

8l

Prepared according to typical procedure at 0 °C for 72 hours by using [Pd(allyl)Cl]$_2$ (2.5 mol%), **Xu9** (10 mol%) from γ-hydroxyalkenes (0.5 mmol) and alkenyl bromide (1 mmol), after flash column chromatography on a silica gel (petroleum ether: ethyl acetate = 50: 1) give the product **8l** as a white liquid (52 mg, 37% yield) with 92% ee. $[\alpha]_D^{20}$ = 3.6 (c = 0.4, Chloroform). ^{1}H NMR (400 MHz, Chloroform-d) δ 6.59 (s, 2H), 6.38 (dt, J = 15.8, 1.4 Hz, 1H), 6.17 (dt, J = 15.8, 7.1 Hz, 1H), 4.00 – 3.89 (m, 2H), 3.87 (s, 6H), 3.83 (s, 3H), 3.76 (td, J = 7.9, 6.4 Hz, 1H), 2.56 – 2.31 (m, 2H), 2.07 – 1.82 (m, 3H), 1.57 (ddt, J = 11.7, 8.5, 7.5 Hz, 1H). ^{13}C NMR (100 MHz, Chloroform-d) δ 153.2, 137.4, 133.4, 131.8, 126.4, 103.1, 78.8, 68.0, 60.9, 56.0, 39.2, 30.9, 25.7. HRMS (ESI) calculated for [$C_{16}H_{22}NaO_4$] [M+Na]$^+$: 301.1410 found: 301.1415. Enantiomeric excess was determined by HPLC with a Chiralpak OD-H column (hexanes: 2-propanol = 95: 5, 0.6 mL/min, 254 nm); major enantiomer tr =21.027 min, minor enantiomer tr = 19.968 min.

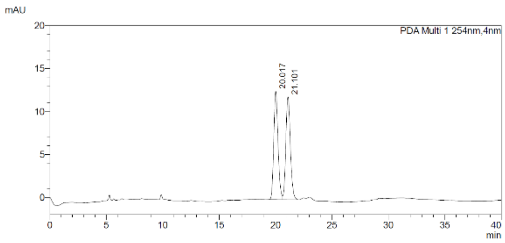

<Peak Table>

PDA Ch1 254nm

Peak#	Ret. Time	Height	Height%	Area	Area%
1	20.017	12630	51.358	330939	49.807
2	21.101	11963	48.642	333501	50.193
Total		24593	100.000	664439	100.000

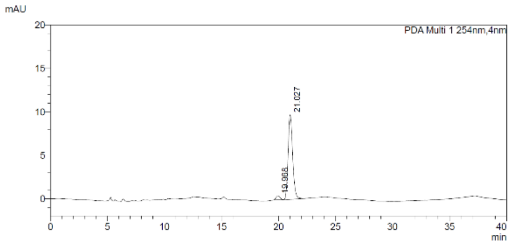

<Peak Table>

PDA Ch1 254nm

Peak#	Ret. Time	Height	Height%	Area	Area%
1	19.968	477	4.672	11163	3.966
2	21.027	9732	95.328	270334	96.034
Total		10209	100.000	281497	100.000

4.2.13 Synthesis of (*S*, *E*)-N,N-dimethyl-4-(3-(tetrahydrofuran-2-yl)prop-1-en-1-yl)aniline (8m)

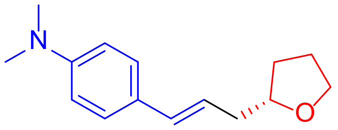

8m

Prepared according to typical procedure at 0 °C for 72 hours by using [Pd(allyl)Cl]$_2$ (2.5 mol%), **Xu9** (10 mol%) from γ-hydroxyalkenes (0.5 mmol) and alkenyl bromide (1 mmol), after flash column chromatography on a silica gel (petroleum ether: ethyl acetate = 50: 1) give the product **8m** as a white liquid (60 mg, 52% yield) with 91% ee. $[\alpha]_D^{20}$ = -6.7 (c = 0.4, Chloroform). ^{1}H NMR (400 MHz, Chloroform-d) δ 7.31 – 7.16 (m, 2H), 6.76 – 6.60 (m, 2H), 6.42 – 6.29 (m, 1H), 6.01 (dt,

J = 15.8, 7.2 Hz, 1H), 4.07 – 3.80 (m, 2H), 3.73 (td, J = 7.9, 6.4 Hz, 1H), 2.92 (s, 6H), 2.47 (dddd, J = 14.5, 7.3, 6.0, 1.5 Hz, 1H), 2.35 (dddd, J = 13.9, 7.6, 6.5, 1.4 Hz, 1H), 2.03 – 1.93 (m, 1H), 1.93 – 1.78 (m, 2H), 1.55 (ddt, J = 11.7, 8.6, 7.5 Hz, 1H).^{13}C NMR (100 MHz, Chloroform-*d*) δ 149.8, 131.8, 127.0, 126.4, 122.4, 112.6, 79.2, 68.0, 40.6, 39.3, 30.8, 25.8. HRMS (ESI) calculated for [$C_{15}H_{22}NO$] [M+Na]$^+$: 232.1696 found: 232.1698. Enantiomeric excess was determined by HPLC with a Chiralpak OD-H column (hexanes: 2-propanol = 95: 5, 0.6 mL/min, 254 nm); major enantiomer tr = 17.316 min, minor enantiomer tr = 14.020 min.（Note: The mixture of 8m and ent-8m with different concentration to gain the HPLC time, all HPLC time difference are less than one minute.）

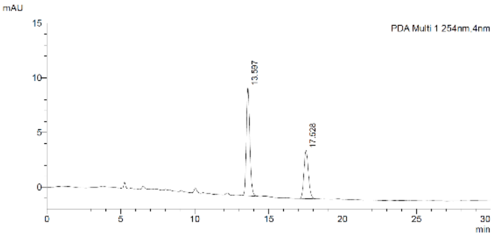

<Peak Table>
PDA Ch1 254nm

Peak#	Ret. Time	Height	Height%	Area	Area%
1	13.597	9889	69.079	161482	63.476
2	17.528	4427	30.921	92918	36.524
Total		14316	100.000	254399	100.000

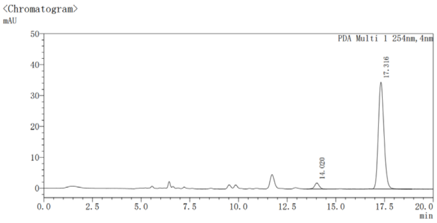

<Peak Table>
PDA Ch1 254nm

No.	Ret.Time(min)	Height(mAU)	Height%	Area(mAU*min)	Area%
1	14.020	1939	5.315	32398	4.456
2	17.316	34550	94.685	694591	95.544
Total		36489	100.000	726988	100.000

4.2.14 Synthesis of (*S*, *E*)-2-(3-(4-(trifluoromethoxy)phenyl)allyl)tetrahydrofuran (8n)

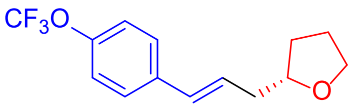

8n

Prepared according to typical procedure at 0 °C for 72 hours by using [Pd(allyl)Cl]$_2$ (2.5 mol%), **Xu9** (10 mol%) from γ-hydroxyalkenes (0.5 mmol) and alkenyl bromide (1 mmol), after flash column chromatography on a silica gel (petroleum ether: ethyl acetate = 50: 1) give the product **8n** as a white liquid (67 mg, 49% yield) with 92% ee. $[\alpha]_D^{20}$ = 4.1 (c = 0.4, Chloroform). ^{1}H NMR (400 MHz, Chloroform-*d*) δ 7.39 – 7.31 (m, 2H), 7.13 (d, J = 8.3 Hz, 2H), 6.44 (d, J = 15.9 Hz, 1H), 6.23 (dt, J = 15.8, 7.1 Hz, 1H), 4.02 – 3.83 (m, 2H), 3.82 – 3.68 (m, 1H), 2.53 – 2.32 (m, 2H), 1.95 (dddd, J = 36.0, 14.7, 7.2, 4.4 Hz, 3H), 1.55 (dq, J = 11.6, 7.8 Hz, 1H). ^{13}C NMR (100 MHz, Chloroform-*d*) δ 148.1 (d, J = 1.9 Hz), 136.4, 130.5, 128.1, 127.2, 121.0, 120.5 (d, J = 256.8 Hz). 78.6, 68.0, 39.2, 30.9, 25.7. ^{19}F NMR (376 MHz, Chloroform-*d*) δ -57.91. HRMS (ESI) calculated for [$C_{14}H_{15}F_3NaO_2$] [M+Na]$^+$: 295.0916 found: 295.0909. Enantiomeric excess was determined by

HPLC with a Chiralpak AD-H column (hexanes: 2-propanol = 99: 1, 0.6 mL/min, 254nm); major enantiomer tr = 10.873 min, minor enantiomer tr = 10.042 min.

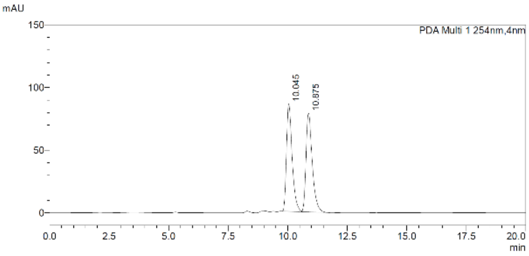

<Peak Table>

PDA Ch1 254nm

Peak#	Ret. Time	Height	Height%	Area	Area%
1	10.045	85789	52.098	1418330	49.535
2	10.875	78879	47.902	1444936	50.465
Total		164669	100.000	2863266	100.000

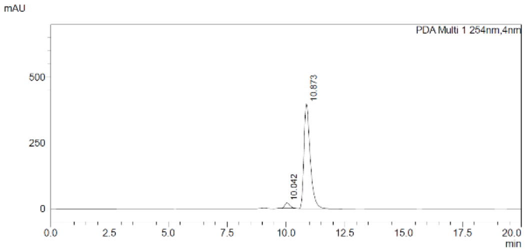

<Peak Table>

PDA Ch1 254nm

Peak#	Ret. Time	Height	Height%	Area	Area%
1	10.042	20344	4.868	311600	4.056
2	10.873	397567	95.132	7371144	95.944
Total		417911	100.000	7682745	100.000

4.2.15 Synthesis of (*S*, *E*)-2-(3-(4-fluorophenyl)allyl)tetrahydrofuran (8o)

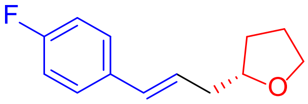

8o

Prepared according to typical procedure at 0 °C for 72 hours by using $[Pd(allyl)Cl]_2$ (2.5 mol%), **Xu9** (10 mol%) from γ-hydroxyalkenes (0.5 mmol) and alkenyl bromide (1 mmol), after flash column chromatography on a silica gel (petroleum ether: ethyl acetate = 50: 1) give the product **8o** as a white liquid (52 mg, 50% yield) with 91% ee. $[\alpha]_D^{20}$ = 3.3 (*c* = 0.4, Chloroform). ^{1}H NMR (400 MHz, Chloroform-*d*) δ 7.35 – 7.27 (m, 2H), 7.07 – 6.91 (m, 2H), 6.48 – 6.35 (m, 1H), 6.15 (dt, *J* = 15.8, 7.1 Hz, 1H), 4.06 – 3.84 (m, 2H), 3.75 (td, *J* = 7.9, 6.4 Hz, 1H), 2.55 – 2.24 (m, 2H), 2.00 (dddd, *J* = 11.3, 8.4, 6.3, 4.9 Hz, 1H), 1.96 – 1.80 (m, 2H), 1.56 (ddt, *J* = 11.6, 8.4, 7.4 Hz, 1H). ^{13}C NMR (100 MHz, Chloroform-*d*) δ 162.0 (d, *J* = 245.8 Hz), 133.7 (d, *J* = 3.3 Hz), 130.7, 127.5 (d, *J* = 7.8 Hz), 126.6 (d, *J* = 2.3 Hz), 115.3 (d, *J* = 21.5 Hz), 78.8, 68.0, 39.2, 30.9, 25.7. ^{19}F NMR (376 MHz, Chloroform-*d*) δ -115.44. HRMS (ESI) calculated for $[C_{13}H_{15}FNaO]$ $[M+Na]^+$: 229.0999 found: 229.0999. Enantiomeric excess was determined by HPLC with a Chiralpak AD-H column (hexanes: 2-propanol = 99: 1, 0.6 mL/min, 254 nm); major enantiomer tr = 12.318 min, minor enantiomer tr = 11.652 min.

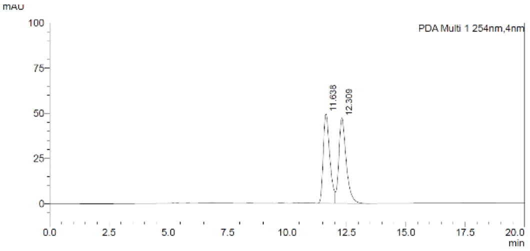

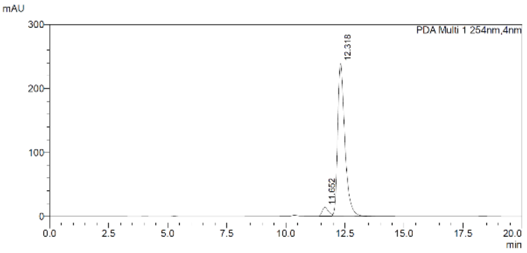

<Peak Table>
PDA Ch1 254nm

Peak#	Ret. Time	Height	Height%	Area	Area%
1	11.638	49440	51.142	952266	48.197
2	12.309	47232	48.858	1023516	51.803
Total		96672	100.000	1975782	100.000

<Peak Table>
PDA Ch1 254nm

Peak#	Ret. Time	Height	Height%	Area	Area%
1	11.652	13674	5.412	249462	4.684
2	12.318	238961	94.588	5076296	95.316
Total		252635	100.000	5325758	100.000

4.2.16 Synthesis of (*S*, *E*)-2-(3-(4-(trifluoromethyl)phenyl)allyl)tetrahydrofuran (8p)

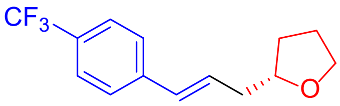

8p

Prepared according to typical procedure at 0 °C for 72 hours by using $[Pd(allyl)Cl]_2$ (2.5 mol%), **Xu9** (10 mol%) from γ-hydroxyalkenes (0.5 mmol) and alkenyl bromide (1 mmol), after flash column chromatography on a silica gel (petroleum ether: ethyl acetate = 50: 1) give the product **8p** as a white liquid (74 mg, 58% yield) with 91% ee. $[\alpha]_D^{20}$=3.4 (*c* = 0.4, Chloroform). ^{1}H NMR (400 MHz, Chloroform-*d*) δ 7.35 (d, *J* = 8.7 Hz, 2H), 7.19 – 7.07 (m, 2H), 6.44 (dd, *J* = 16.0, 1.6 Hz, 1H), 6.23 (dt, *J* = 15.9, 7.1 Hz, 1H), 4.05 – 3.85 (m, 2H), 3.75 (td, *J* = 7.8, 6.4 Hz, 1H), 2.54 – 2.34 (m, 2H), 2.07 – 1.80 (m, 3H), 1.62 – 1.44 (m, 1H). ^{13}C NMR (100 MHz, Chloroform-*d*) δ 148.13, 148.11, 136.38, 130.47, 128.06, 127.22, 121.77, 120.97, 119.21, 78.64, 67.98, 39.14, 30.91, 25.70. ^{19}F NMR (376 MHz, CDCl3) δ -57.92. HRMS (ESI) calculated for $[C_{14}H_{16}F_3O]$ $[M+H]^+$: 257.1148 found: 257.1145. Enantiomeric excess was determined by HPLC with a Chiralpak AD-H column (hexanes: 2-propanol = 99: 1, 0.6 mL/min, 254 nm); major enantiomer tr = 10.956 min, minor enantiomer tr = 10.078 min.

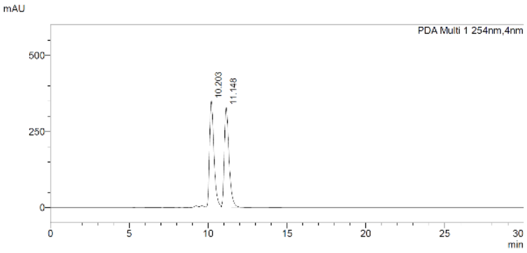

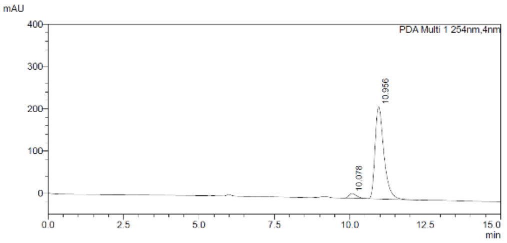

<Peak Table>
PDA Ch1 254nm

Peak#	Ret. Time	Height	Height%	Area	Area%
1	10.203	349182	51.530	6748061	49.813
2	11.148	328451	48.470	6798604	50.187
Total		677633	100.000	13546665	100.000

<Peak Table>
PDA Ch1 254nm

Peak#	Ret. Time	Height	Height%	Area	Area%
1	10.078	11204	4.867	206445	4.424
2	10.956	218974	95.133	4459941	95.576
Total		230178	100.000	4666385	100.000

4.2.17 Synthesis of (*S*, *E*)-2-(3-(thiophen-2-yl)allyl)tetrahydrofuran (8q)

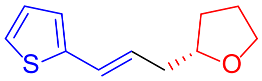

8q

Prepared according to typical procedure at 0 °C for 72 hours by using [Pd(allyl)Cl]$_2$ (2.5 mol%), **Xu9** (10 mol%) from γ-hydroxyalkenes (0.5 mmol) and alkenyl bromide (1 mmol), after flash column chromatography on a silica gel (petroleum ether: ethyl acetate = 50: 1) give the product **8q** as a white liquid (64 mg, 65% yield) with 91% ee. $[\alpha]_D^{20}$ = 5.3 (*c* = 0.4, Chloroform). ^{1}H NMR (400 MHz, Chloroform-*d*) δ 7.09 (dt, *J* = 5.1, 0.8 Hz, 1H), 6.97 – 6.84 (m, 2H), 6.58 (dd, *J* = 15.7, 1.6 Hz, 1H), 6.07 (dt, *J* = 15.7, 7.2 Hz, 1H), 4.01 – 3.84 (m, 2H), 3.74 (td, *J* = 7.8, 6.4 Hz, 1H), 2.54 – 2.26 (m, 2H), 2.00 (dddd, *J* = 11.4, 8.4, 6.4, 4.9 Hz, 1H), 1.94 – 1.81 (m, 2H), 1.55 (ddt, *J* = 11.7, 8.4, 7.4 Hz, 1H). ^{13}C NMR (100 MHz, Chloroform-*d*) δ 142.8, 127.2, 126.7, 125.2, 124.7, 123.4, 78.6, 68.0, 39.1, 30.9, 25.8. HRMS (ESI) calculated for [$C_{11}H_{15}OS$] $[M+H]^+$:195.0838 found: 195.837. Enantiomeric excess was determined by HPLC with a Chiralpak OD-H column (hexanes: 2-propanol = 95: 5, 0.6 mL/min, 254 nm); major enantiomer tr = 15.378 min, minor enantiomer tr = 11.923 min.

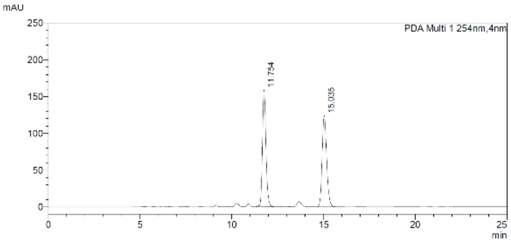

<Peak Table>
PDA Ch1 254nm

Peak#	Ret. Time	Height	Height%	Area	Area%
1	11.754	158840	56.135	2136142	50.154
2	15.035	124119	43.865	2122985	49.846
Total		282959	100.000	4259127	100.000

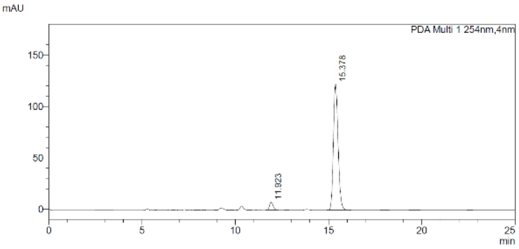

<Peak Table>
PDA Ch1 254nm

Peak#	Ret. Time	Height	Height%	Area	Area%
1	11.923	7758	5.955	102316	4.570
2	15.378	122514	94.045	2136413	95.430
Total		130271	100.000	2238729	100.000

4.2.18 Synthesis of (*S*, *E*)-2-(3-(naphthalen-1-yl)allyl)tetrahydrofuran (8r)

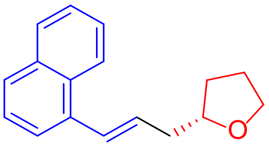

8r

Prepared according to typical procedure at 0 °C for 72 hours by using [Pd(allyl)Cl]$_2$ (2.5 mol%), **Xu9** (10 mol%) from γ-hydroxyalkenes (0.5 mmol) and alkenyl bromide (1 mmol), after flash column chromatography on a silica gel (petroleum ether: ethyl acetate = 50: 1) give the product **8r** as a white liquid (79 mg, 66% yield) with 92% ee. $[\alpha]_D^{20}$ = -4.0 (*c* = 0.4, Chloroform). ^{1}H NMR (400 MHz, Chloroform-*d*) δ 8.17 – 8.06 (m, 1H), 7.83 (dd, *J* = 8.0, 1.6 Hz, 1H), 7.74 (d, *J* = 8.2 Hz, 1H),

7.57 (dt, J = 7.1, 0.9 Hz, 1H), 7.53 – 7.39 (m, 3H), 7.19 (d, J = 15.6 Hz, 1H), 6.25 (dt, J = 15.5, 7.1 Hz, 1H), 4.03 (dq, J = 7.7, 6.3 Hz, 1H), 3.93 (ddd, J = 8.3, 7.2, 6.2 Hz, 1H), 3.77 (td, J = 7.9, 6.4 Hz, 1H), 2.58 (ddddd, J = 44.9, 14.0, 7.6, 6.3, 1.5 Hz, 2H), 2.04 (dddd, J = 11.6, 8.5, 6.5, 4.9 Hz, 1H), 1.99 – 1.81 (m, 2H), 1.69 – 1.61 (m, 1H). ^{13}C NMR (100 MHz, Chloroform-*d*) δ 135.4, 133.6, 131.1, 130.1, 129.2, 128.5, 127.5, 125.9, 125.7, 123.9, 123.7, 78.8, 68.1, 39.6, 30.9, 25.8. HRMS (ESI) calculated for [$C_{17}H_{18}NaO$] $[M+Na]^+$: 261.1250 found: 261.1256. Enantiomeric excess was determined by HPLC with a Chiralpak OD-H column (hexanes: 2-propanol = 95: 5, 0.6 mL/min, 254 nm); major enantiomer tr = 16.388 min, minor enantiomer tr = 15.385 min.

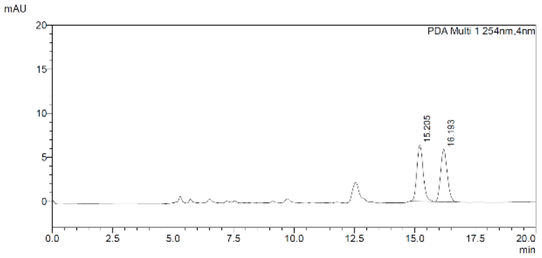

<Peak Table>
PDA Ch1 254nm

Peak#	Ret. Time	Height	Height%	Area	Area%
1	15.205	6399	51.745	115888	50.176
2	16.193	5967	48.255	115076	49.824
Total		12366	100.000	230964	100.000

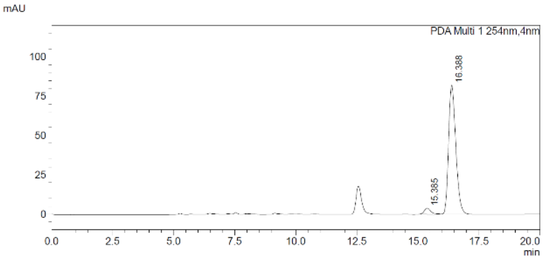

<Peak Table>
PDA Ch1 254nm

Peak#	Ret. Time	Height	Height%	Area	Area%
1	15.385	3810	4.439	72662	4.124
2	16.388	82021	95.561	1689255	95.876
Total		85831	100.000	1761918	100.000

4.2.19 Synthesis of (*S*, *E*)-2-(3-(naphthalen-2-yl)allyl)tetrahydrofuran (8s)

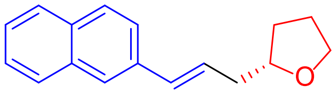

8s

Prepared according to typical procedure at 0 °C for 72 hours by using [Pd(allyl)Cl]$_2$ (2.5 mol%), **Xu9** (10 mol%) from γ-hydroxyalkenes (0.5 mmol) and alkenyl bromide (1 mmol), after flash column chromatography on a silica gel (petroleum ether: ethyl acetate = 50: 1) give the product **8s** as a yellow solid (69 mg, 58% yield) with 90% ee. Mp: 31.2– 34.3 °C. $[\alpha]_D^{20}$ = 0.9 (c = 0.4, Chloroform). ^{1}H NMR (400 MHz, Chloroform-*d*)δ 7.75 (t, J = 8.3 Hz, 3H), 7.67 (d, J = 1.6 Hz, 1H), 7.58 (dd, J = 8.6, 1.7 Hz, 1H), 7.40 (pd, J = 7.0, 1.4 Hz, 2H), 6.59 (d, J = 15.8 Hz, 1H), 6.35 (dt, J = 15.9, 7.1 Hz, 1H), 4.07 – 3.85 (m, 2H), 3.81 – 3.69 (m, 1H), 2.48 (dq, J = 30.7, 7.2 Hz, 2H), 2.05 – 1.93 (m, 1H), 1.93 – 1.79 (m, 2H), 1.56 (dq, J = 11.7, 7.8 Hz, 1H). ^{13}C NMR (100 MHz, Chloroform-*d*) δ 134.9, 133.5, 132.6, 131.9, 127.9, 127.8, 127.5, 127.2, 126.0, 125.6, 125.5, 123.5, 78.7, 67.9, 39.3, 30.8, 25.6. HRMS (ESI) calculated for [$C_{17}H_{18}NaO$] $[M+Na]^+$: 261.1250 found: 261.1255. Enantiomeric excess was determined by HPLC with a Chiralpak AD-H column (hexanes:

2-propanol = 95: 5, 0.6 mL/min, 254 nm); major enantiomer tr = 11.337 min, minor enantiomer tr = 10.624 min.

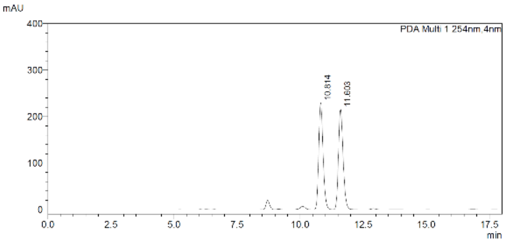

<Peak Table>
PDA Ch1 254nm

Peak#	Ret. Time	Height	Height%	Area	Area%
1	10.814	229931	51.566	2769786	49.873
2	11.603	215963	48.434	2783912	50.127
Total		445894	100.000	5553697	100.000

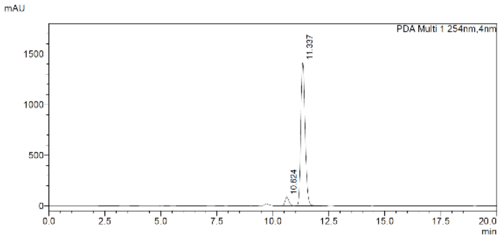

<Peak Table>
PDA Ch1 254nm

Peak#	Ret. Time	Height	Height%	Area	Area%
1	10.624	86975	5.776	1036500	5.191
2	11.337	1418805	94.224	18932652	94.809
Total		1505781	100.000	19969152	100.000

4.2.20 Synthesis of (*S*)-2-((2*E*,4*E*)-5-phenylpenta-2,4-dien-1-yl)tetrahydrofuran (8t)

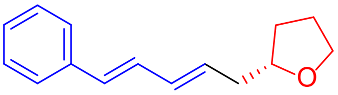

8t

Prepared according to typical procedure at 0 ºC for 72 hours by using $[Pd(allyl)Cl]_2$ (2.5 mol%), **Xu9** (10 mol%) from γ-hydroxyalkenes (0.5 mmol) and alkenyl bromide (1 mmol), after flash column chromatography on a silica gel (petroleum ether: ethyl acetate = 50: 1) give the product **8t** as a white liquid (78 mg, 73% yield) with 91% ee. $[\alpha]_D^{20}$ = 4.4 (*c* = 0.4, Chloroform).^{1}H NMR (400 MHz, Chloroform-*d*) δ 7.41 – 7.34 (m, 2H), 7.29 (dd, *J* = 8.5, 6.8 Hz, 2H), 7.23 – 7.15 (m, 1H), 6.76 (dd, *J* = 15.6, 10.4 Hz, 1H), 6.46 (d, *J* = 15.7 Hz, 1H), 6.36 – 6.19 (m, 1H), 5.83 (dt, *J* = 14.9, 7.3 Hz, 1H), 4.01 – 3.83 (m, 2H), 3.74 (td, *J* = 7.9, 6.5 Hz, 1H), 2.51 – 2.27 (m, 2H), 2.06 – 1.77 (m, 3H), 1.53 (ddt, *J* = 11.8, 8.5, 7.5 Hz, 1H). ^{13}C NMR (100 MHz, Chloroform-*d*) δ 137.6, 132.6, 131.4, 130.7, 129.2, 128.6, 127.2, 126.2, 78.8, 68.0, 39.1, 30.9, 25.7. HRMS (ESI) calculated for $[C_{15}H_{18}NaO]$ $[M+Na]^+$: 237.1250 found: 237.1249. Enantiomeric excess was determined by HPLC with a Chiralpak OD-H column (hexanes: 2-propanol = 95: 5, 0.6 mL/min, 254 nm); major enantiomer tr =9.507 min, minor enantiomer tr = 10.029 min.

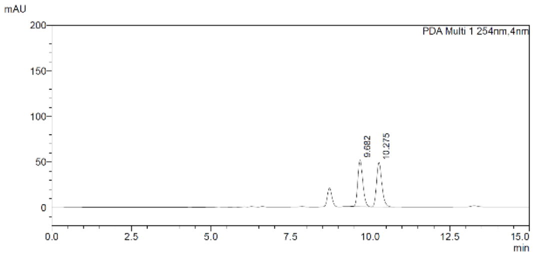

<Peak Table>
PDA Ch1 254nm

Peak#	Ret. Time	Height	Height%	Area	Area%
1	9.682	51207	51.164	568802	49.875
2	10.275	48877	48.836	571648	50.125
Total		100085	100.000	1140450	100.000

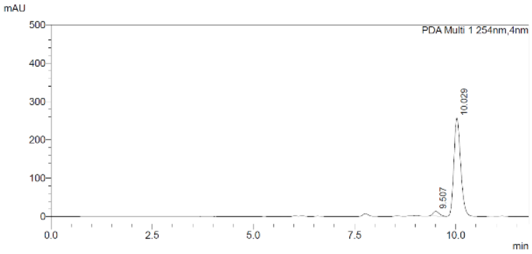

<Peak Table>
PDA Ch1 254nm

Peak#	Ret. Time	Height	Height%	Area	Area%
1	9.507	12468	4.628	133356	4.277
2	10.029	256942	95.372	2984693	95.723
Total		269410	100.000	3118049	100.000

4.2.21 Synthesis of (*S*)-2-(2-phenylallyl)tetrahydrofuran (8u)

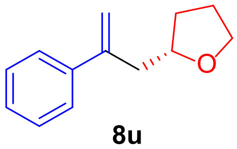

8u

Prepared according to typical procedure at 0 °C for 72 hours by using $[Pd(allyl)Cl]_2$ (2.5 mol%), **Xu9** (10 mol%) from γ-hydroxyalkenes (0.5 mmol) and alkenyl bromide (1 mmol), after flash column chromatography on a silica gel (petroleum ether: ethyl acetate = 50: 1) give the product **8u** as a white liquid (32 mg, 34% yield) with 87% *ee*. $[\alpha]_D^{20}$ = -1.4 (*c* = 0.4, Chloroform). ^{1}H NMR (400 MHz, Chloroform-*d*) δ 7.45 – 7.38 (m, 2H), 7.32 (ddd, *J* = 8.1, 7.1, 1.1 Hz, 2H), 7.29 – 7.26 (m, 1H), 5.34 (d, *J* = 1.5 Hz, 1H), 5.15 (q, *J* = 1.3 Hz, 1H), 4.00 – 3.84 (m, 2H), 3.69 (td, *J* = 8.2, 6.2 Hz, 1H), 2.88 (ddd, *J* = 14.3, 6.5, 1.3 Hz, 1H), 2.60 (ddd, *J* = 14.3, 7.0, 1.2 Hz, 1H), 1.97 – 1.73 (m, 3H), 1.58 – 1.43 (m, 1H). ^{13}C NMR (100 MHz, Chloroform-*d*) δ 145.7, 141.1, 128.5, 128.3, 127.4, 126.2, 126.1, 114.2, 77.6, 67.8, 41.6, 31.1, 25.6. HRMS (ESI) calculated for $[C_{13}H_{17}O]$ $[M+H]^+$: 189.1274 found: 189.1278. Enantiomeric excess was determined by HPLC with a Chiralpak AD-H column (hexanes: 2-propanol = 95: 5, 0.6 mL/min, 254 nm); major enantiomer tr = 7.345 min, minor enantiomer tr = 7.893 min.

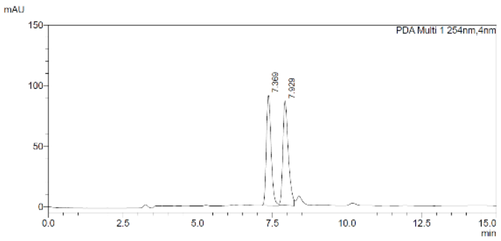

<Peak Table>

PDA Ch1 254nm

Peak#	Ret. Time	Height	Height%	Area	Area%
1	7.369	91287	51.415	1010353	48.224
2	7.929	86261	48.585	1084779	51.776
Total		177548	100.000	2095132	100.000

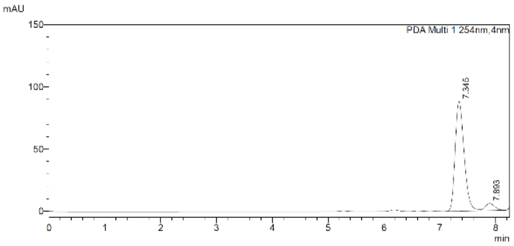

<Peak Table>

PDA Ch1 254nm

Peak#	Ret. Time	Height	Height%	Area	Area%
1	7.345	88434	93.855	989897	93.596
2	7.893	5790	6.145	67731	6.404
Total		94224	100.000	1057628	100.000

4.2.22 Synthesis of (*S*)-2-((1H-inden-2-yl)methyl)tetrahydrofuran (8v)

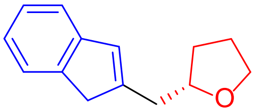

8v

Prepared according to typical procedure at 0 °C for 72 hours by using $[Pd(allyl)Cl]_2$ (2.5 mol%), **Xu9** (10 mol%) from γ-hydroxyalkenes (0.5 mmol) and alkenyl bromide (1 mmol), after flash column chromatography on a silica gel (petroleum ether: ethyl acetate = 50: 1) give the product **8v** as a white liquid (82 mg, 82% yield) with 95% *ee*. $[\alpha]_D^{20}$ = -3.7 (*c* = 0.4, Chloroform). ^{1}H NMR (400 MHz, Chloroform-*d*)δ 7.38 (dq, *J* = 7.4, 0.9 Hz, 1H), 7.28 (dt, *J* = 7.5, 0.9 Hz, 1H), 7.21 (td, *J*

= 7.4, 1.0 Hz, 1H), 7.10 (td, *J* = 7.4, 1.3 Hz, 1H), 6.61 – 6.57 (m, 1H), 4.11 (dq, *J* = 7.6, 6.4 Hz, 1H), 3.91 (ddd, *J* = 8.3, 7.1, 6.2 Hz, 1H), 3.75 (td, *J* = 7.9, 6.5 Hz, 1H), 3.42 – 3.34 (m, 2H), 2.78 (ddd, *J* = 14.8, 6.6, 1.3 Hz, 1H), 2.66 (ddd, *J* = 14.8, 6.2, 1.1 Hz, 1H), 2.02 (dddd, *J* = 11.6, 8.4, 6.4, 5.0 Hz, 1H), 1.96 – 1.83 (m, 2H), 1.59 – 1.51 (m, 1H). ^{13}C NMR (100 MHz, Chloroform-*d*) δ 147.1, 145.4, 143.3, 128.0, 126.2, 123.8, 123.4, 120.1, 78.6, 67.9, 41.7, 37.4, 31.3, 25.7. HRMS (ESI) calculated for [$C_{14}H_{17}O$] [M+H]$^+$: 201.1274 found: 201.1278. Enantiomeric excess was determined by HPLC with a Chiralpak OD-H column (hexanes: 2-propanol = 95: 5, 0.6 mL/min, 254 nm); major enantiomer tr = 10.973 min, minor enantiomer tr = 10.363 min.

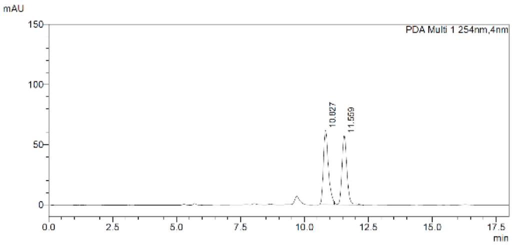

<Peak Table>
PDA Ch1 254nm

Peak#	Ret. Time	Height	Height%	Area	Area%
1	10.827	61939	51.817	833252	51.710
2	11.559	57596	48.183	778144	48.290
Total		119535	100.000	1611396	100.000

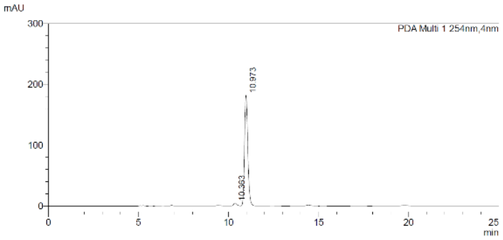

<Peak Table>
PDA Ch1 254nm

Peak#	Ret. Time	Height	Height%	Area	Area%
1	10.363	4796	2.567	57272	2.428
2	10.973	182044	97.433	2301977	97.572
Total		186840	100.000	2359248	100.000

4.2.23 Synthesis of (*S*)-2-((*R*, *E*)-3-(4-methoxyphenyl)allyl-1-d)tetrahydrofuran (8-D)

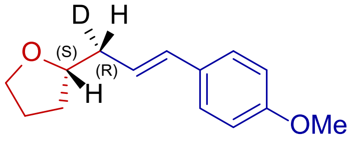

8-D

Prepared according to typical procedure at 0 ºC for 36 hours by using [Pd(allyl)Cl]$_2$ (2.5 mol%), **Xu9** (10 mol%) from D-γ-hydroxyalkenes (0.5 mmol) and alkenyl bromide (1 mmol), after flash column chromatography on a silica gel (petroleum ether: ethyl acetate = 50: 1) give the product **8-D** as a white liquid (68 mg, 62% yield) with 92% *ee*, >20:1 dr. $[\alpha]_D^{20}$ = -2.5 (*c* = 0.4, Chloroform).^{1}H NMR (400 MHz, Chloroform-*d*) δ 7.31 – 7.26 (m, 2H), 6.89 – 6.77 (m, 2H), 6.39 (dd, *J* = 15.9, 1.4 Hz, 1H), 6.08 (dd, *J* = 15.8, 7.0 Hz, 1H), 3.98 – 3.85 (m, 2H), 3.78 (s, 3H), 3.74 (td, *J* = 7.9, 6.4 Hz, 1H), 2.45 (td, *J* = 6.7, 1.7 Hz, 1H), 1.98 (dddd, *J* = 11.4, 8.6, 6.4, 4.9 Hz, 1H), 1.92 – 1.79 (m, 2H), 1.55 (ddt, *J* = 11.7, 8.5, 7.5 Hz, 1H). ^{13}C NMR (100 MHz, Chloroform-*d*) δ 158.7, 131.2, 130.3, 127.1, 124.4, 113.8, 78.8, 67.9, 55.2, 39.1-38.4 (m), 30.7, 25.7.RMS (ESI) calculated for [$C_{14}H_{17}DNaO_2$] [M+Na]$^+$: 242.1262 found: 242.1258. Enantiomeric excess was determined by HPLC with a Chiralpak OD-H column (hexanes: 2-propanol = 99: 1, 0.6 mL/min, 254 nm); major enantiomer tr = 27.808 min, minor enantiomer tr = 21.379 min.

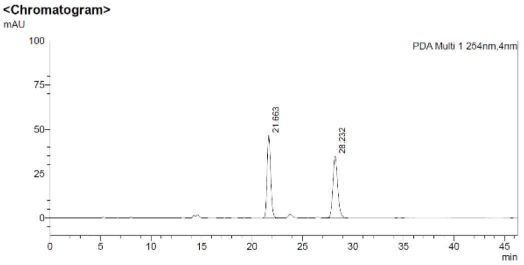

<Peak Table>
PDA Ch1 254nm

Peak#	Ret. Time	Height	Height%	Area	Area%
1	21.663	46657	57.187	1151447	49.928
2	28.232	34929	42.813	1154755	50.072
Total		81586	100.000	2306202	100.000

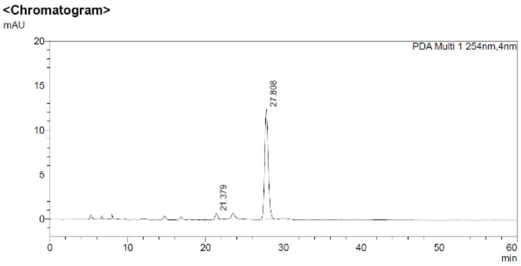

<Peak Table>
PDA Ch1 254nm

Peak#	Ret. Time	Height	Height%	Area	Area%
1	21.379	681	5.216	15769	3.804
2	27.808	12371	94.784	398785	96.196
Total		13051	100.000	414554	100.000

4.3 General procedure for reactions of primary /secondary γ-hydroxy alkenes with 2b

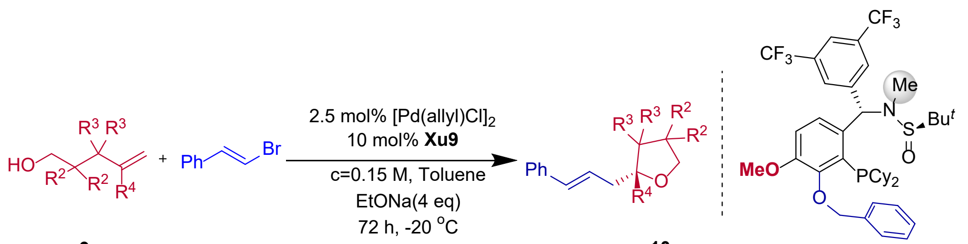

9 **2b** **10** **Xu9**

To a sealed tube was added [Pd(allyl)Cl]$_2$ (2.5 mol%), **Xu9** (10 mol%). The flask was evacuated and refilled with argon. Then γ-hydroxyalkenes (0.5 mmol), alkenyl halides (1 mmol), EtONa (4.0 equiv.), and solution of toluene (3.5 mL) was added to the tube, and stirred at -20 °C for 72 hours. After the reaction was complete (monitored by TLC), solvent was removed under reduced pressure. The crude product was then purified by flash column chromatography on silica gel to afford the desired product.

4.3.1 Synthesis of (*R*)-3,3-dibenzyl-2-cinnamyltetrahydrofuran (10a)

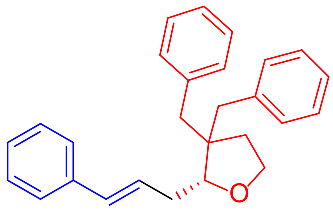

10a

Prepared according to typical procedure at -20 °C for 72 hours by using [Pd(allyl)Cl]$_2$ (2.5 mol%), **Xu9** (10 mol%) from γ-hydroxyalkenes **9a** (0.5 mmol) and alkenyl bromide (1 mmol), after flash column chromatography on a silica gel (petroleum ether: ethyl acetate = 50: 1) give the product **10a** as a white liquid (116 mg, 63% yield) with 93% *ee*. $[\alpha]^{20}_{D}$ = 59.5 (*c* = 0.4, Chloroform).^{1}H NMR (400 MHz, Chloroform-*d*) δ 7.39 – 7.31 (m, 4H), 7.30 – 7.16 (m, 9H), 7.05 – 7.00 (m, 2H), 6.50 (d, *J* = 15.8 Hz, 1H), 6.32 (dt, *J* = 15.8, 6.9 Hz, 1H), 4.00 – 3.89 (m, 1H), 3.67 (ddt, *J* = 9.7, 6.7, 3.2

Hz, 2H), 2.88 – 2.73 (m, 3H), 2.68 – 2.60 (m, 1H), 2.56 – 2.42 (m, 2H), 1.83 (ddd, *J* = 12.8, 7.4, 3.2 Hz, 1H), 1.67 – 1.57 (m, 1H).^{13}C NMR (100 MHz, Chloroform-*d*) δ 138.7, 137.6, 137.4, 131.6, 131.0, 130.9, 128.5, 128.2, 128.1, 128.0, 127.1, 126.4, 126.2, 82.8, 65.1, 48.0, 39.9, 38.7, 33.1, 31.7. HRMS (ESI) calculated for [$C_{27}H_{28}NaO$] $[M+Na]^+$: 391.2032 found: 391.2022. Enantiomeric excess was determined by HPLC with a Chiralpak AD-H column (hexanes: 2-propanol = 99: 1, 0.6 mL/min, 254 nm); major enantiomer tr = 13.311 min, minor enantiomer tr = 11.545 min.

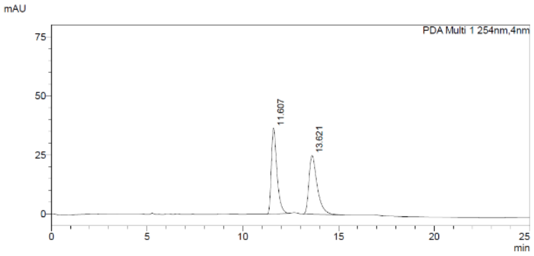

<Peak Table>

PDA Ch1 254nm

Peak#	Ret. Time	Height	Height%	Area	Area%
1	11.607	36318	59.387	741970	49.824
2	13.621	24837	40.613	747222	50.176
Total		61155	100.000	1489192	100.000

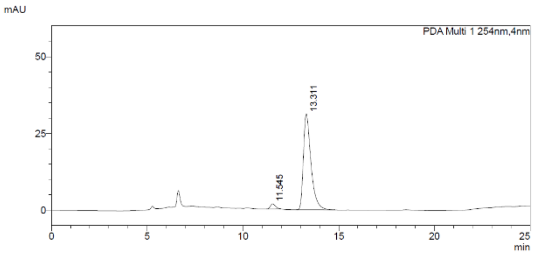

<Peak Table>

PDA Ch1 254nm

Peak#	Ret. Time	Height	Height%	Area	Area%
1	11.545	1679	5.105	31366	3.526
2	13.311	31207	94.895	858270	96.474
Total		32885	100.000	889636	100.000

4.3.2 Synthesis of (*R*)-2-cinnamyl-3,3-bis(4-methylbenzyl)tetrahydrofuran (10b)

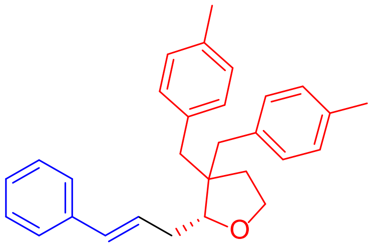

10b

Prepared according to typical procedure at -20 °C for 72 hours by using $[Pd(allyl)Cl]_2$ (2.5 mol%), **Xu9** (10 mol%) from γ-hydroxyalkenes (0.5 mmol) and alkenyl bromide (1 mmol), after flash column chromatography on a silica gel (petroleum ether: ethyl acetate = 50: 1) give the product **10b** as a white liquid (116 mg, 63% yield) with 92% *ee*. $[\alpha]_D^{20}$ = 41.1 (*c* = 0.4, Chloroform). ^{1}H NMR (400 MHz, Chloroform-*d*) δ 7.40 – 7.35 (m, 2H), 7.28 (dd, *J* = 8.4, 6.8 Hz, 2H), 7.21 – 7.09 (m, 5H), 7.05 (d, *J* = 7.8 Hz, 2H), 6.97 – 6.86 (m, 2H), 6.50 (d, *J* = 15.7 Hz, 1H), 6.32 (dt, *J* = 15.8, 6.9 Hz, 1H), 3.99 – 3.87 (m, 1H), 3.66 (ddd, *J* = 9.4, 8.3, 3.1 Hz, 2H), 2.84 – 2.68 (m, 3H), 2.63 – 2.56 (m, 1H), 2.48 (dddt, *J* = 13.2, 6.7, 3.0, 1.4 Hz, 2H), 2.36 (s, 3H), 2.30 (s, 3H), 1.82 (ddd, *J* = 12.7, 7.4, 3.1 Hz, 1H), 1.66 – 1.57 (m, 1H). ^{13}C NMR (100 MHz, Chloroform-*d*) δ 137.7, 135.8, 135.5, 134.3, 131.4, 130.8, 130.8, 128.9, 128.8, 128.5, 128.1, 127.0, 126.2, 82.8, 65.1, 48.0, 39.4, 38.2, 33.1, 31.6, 21.1, 21.1, 21.0. HRMS (ESI) calculated for [$C_{29}H_{32}NaO$] $[M+Na]^+$: 419.2345 found: 419.2354. Enantiomeric excess was determined by HPLC with a Chiralpak AS-H column (hexanes:

2-propanol = 99: 1, 0.6 mL/min, 254 nm); major enantiomer tr = 9.629 min, minor enantiomer tr = 7.807 min.

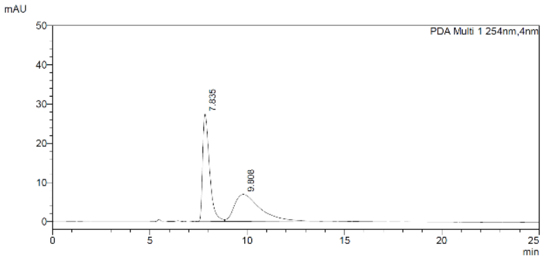

<Peak Table>

PDA Ch1 254nm

Peak#	Ret. Time	Height	Height%	Area	Area%
1	7.835	27493	79.952	679075	51.772
2	9.808	6894	20.048	632587	48.228
Total		34386	100.000	1311661	100.000

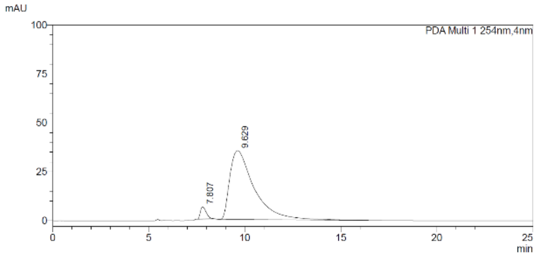

<Peak Table>

PDA Ch1 254nm

Peak#	Ret. Time	Height	Height%	Area	Area%
1	7.807	6290	15.199	130915	4.035
2	9.629	35092	84.801	3113322	95.965
Total		41381	100.000	3244237	100.000

4.3.3 Synthesis of (*R*)-2-cinnamyl-3,3-bis(4-fluorobenzyl)tetrahydrofuran (10c)

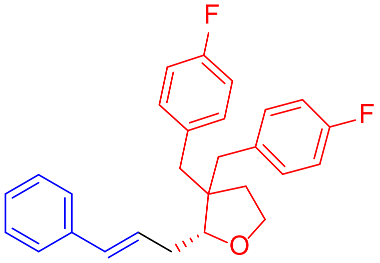

10c

Prepared according to typical procedure at -20 ºC for 72 hours by using $[Pd(allyl)Cl]_2$ (2.5 mol%), **Xu9** (10 mol%) from γ-hydroxyalkenes (0.5 mmol) and alkenyl bromide (1 mmol), after flash column chromatography on a silica gel (petroleum ether: ethyl acetate = 50: 1) give the product **10c** as a white liquid (140 mg, 69% yield) with 93% *ee*. $[\alpha]_D^{20}$ = 57.1 (*c* = 0.4, Chloroform). ^{1}H NMR (400 MHz, Chloroform-*d*) δ 7.40 – 7.33 (m, 2H), 7.29 (dd, *J* = 8.4, 6.8 Hz, 2H), 7.24 – 7.12 (m, 3H), 7.08 – 6.89 (m, 6H), 6.51 (d, *J* = 15.9 Hz, 1H), 6.31 (dt, *J* = 15.8, 6.9 Hz, 1H), 3.92 (td, *J* = 8.7, 7.5 Hz, 1H), 3.73 – 3.59 (m, 2H), 2.82 (d, *J* = 13.7 Hz, 1H), 2.79 – 2.67 (m, 2H), 2.59 (d, *J* = 13.7 Hz, 1H), 2.54 – 2.44 (m, 2H), 1.79 (ddd, *J* = 12.8, 7.5, 3.3 Hz, 1H), 1.65 – 1.53 (m, 1H). ^{13}C NMR (100 MHz, Chloroform-*d*) δ 161.72 (d, *J* = 244.9 Hz), 161.64 (d, *J* = 245.1 Hz), 137.4, 134.1 (d, *J* = 3.3 Hz), 132.8 (d, *J* = 3.4 Hz), 132.8, 132.8, 132.2, 132.1, 131.7, 128.5, 127.6, 127.2, 126.2, 115.1 (d, J = 10.5 Hz), 114.9 (d, J = 10.4 Hz), 82.7, 65.0, 47.9, 39.0, 37.9, 33.1, 31.5. ^{19}F NMR (376 MHz, CDCl3) δ -116.37, -116.57. HRMS (ESI) calculated for $[C_{27}H_{26}F_2NaO]$ $[M+Na]^+$: 427.1844 found: 427.1848. Enantiomeric excess was determined by HPLC with a Chiralpak AD-H column (hexanes: 2-propanol = 95: 5, 0.6 mL/min, 254 nm); major enantiomer tr = 15.830 min, minor enantiomer tr = 13.675 min.

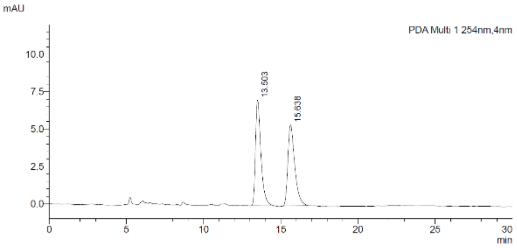

<Peak Table>
PDA Ch1 254nm

Peak#	Ret. Time	Height	Height%	Area	Area%
1	13.503	7083	56.547	173276	50.752
2	15.638	5443	43.453	168139	49.248
Total		12526	100.000	341415	100.000

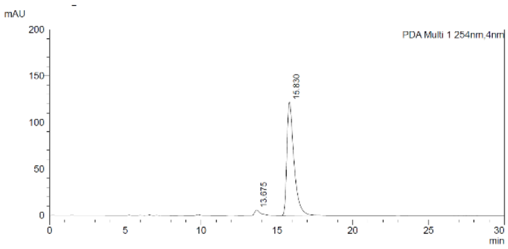

<Peak Table>
PDA Ch1 254nm

Peak#	Ret. Time	Height	Height%	Area	Area%
1	13.675	5587	4.375	137217	3.359
2	15.830	122118	95.625	3947530	96.641
Total		127705	100.000	4084747	100.000

4.3.4 Synthesis of (*R*)-3,3-bis(4-chlorobenzyl)-2-cinnamyltetrahydrofuran (10d)

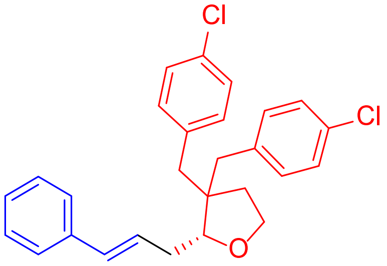

10d

Prepared according to typical procedure at -20 °C for 72 hours by using [Pd(allyl)Cl]$_2$ (2.5 mol%), **Xu9** (10 mol%) from γ-hydroxyalkenes (0.5 mmol) and alkenyl bromide (1 mmol), after flash column chromatography on a silica gel (petroleum ether: ethyl acetate = 50: 1) give the product **10d** as a yellow solid (170 mg, 78% yield) with 94% *ee*. Mp: 86.0-89.1 °C. $[\alpha]_D^{20}$ = 41.0 (*c* = 0.4, Chloroform).^{1}H NMR (400 MHz, Chloroform-*d*) δ 7.40 – 7.34 (m, 2H), 7.33 – 7.26 (m, 4H), 7.25 – 7.17 (m, 3H), 7.16 – 7.09 (m, 2H), 6.97 – 6.91 (m, 2H), 6.51 (d, *J* = 15.7 Hz, 1H), 6.30 (dt, *J* = 15.8, 6.9 Hz, 1H), 3.91 (td, *J* = 8.7, 7.5 Hz, 1H), 3.71 – 3.57 (m, 2H), 2.82 (d, *J* = 13.5 Hz, 1H), 2.78 – 2.66 (m, 2H), 2.63 – 2.55 (m, 1H), 2.54 – 2.39 (m, 2H), 1.78 (ddd, *J* = 12.9, 7.5, 3.3 Hz, 1H), 1.64 – 1.56 (m, 1H). ^{13}C NMR (100 MHz, Chloroform-*d*) δ 137.4, 136.8, 135.5, 132.4, 132.1, 132.1, 131.7, 128.5, 128.4, 128.3, 127.4, 127.2, 126.2, 82.6, 64.9, 47.9, 39.2, 38.1, 33.0, 31.5. HRMS (ESI) calculated for [$C_{27}H_{26}Cl_2NaO$] $[M+Na]^+$: 459.1253 found: 459.1263. Enantiomeric excess was determined by HPLC with a Chiralpak AD-H column (hexanes: 2-propanol = 95: 5, 0.6 mL/min, 254 nm); major enantiomer tr = 15.804 min, minor enantiomer tr = 14.077 min.

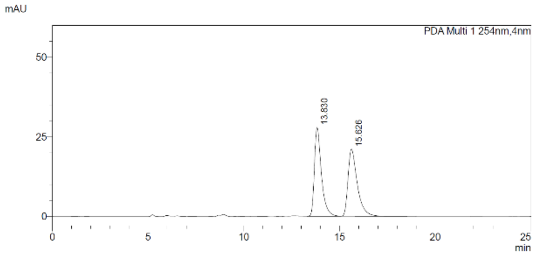

<Peak Table>
PDA Ch1 254nm

Peak#	Ret. Time	Height	Height%	Area	Area%
1	13.830	27864	56.930	706017	49.857
2	15.626	21080	43.070	710054	50.143
Total		48943	100.000	1416071	100.000

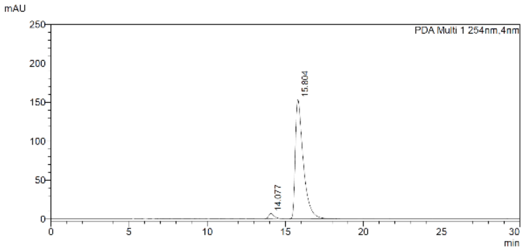

<Peak Table>
PDA Ch1 254nm

Peak#	Ret. Time	Height	Height%	Area	Area%
1	14.077	6560	4.099	154553	2.763
2	15.804	153468	95.901	5440036	97.237
Total		160028	100.000	5594589	100.000

4.3.5 Synthesis of (*R*)-2-cinnamyl-3,3-bis(4-(trifluoromethyl)benzyl)tetrahydrofuran (10e)

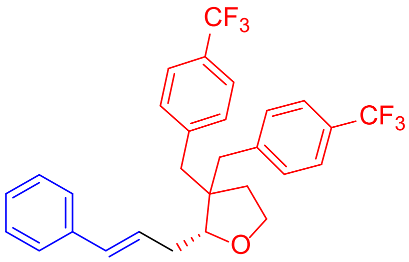

10e

Prepared according to typical procedure at -20 °C for 72 hours by using [Pd(allyl)Cl]$_2$ (2.5 mol%), **Xu9** (10 mol%) from γ-hydroxyalkenes (0.5 mmol) and alkenyl bromide (1 mmol), after flash column chromatography on a silica gel (petroleum ether: ethyl acetate = 50: 1) give the product **10e** as a white liquid (192 mg, 76% yield) with 93% *ee*. $[\alpha]_D^{20}$ = 41.5 (*c* = 0.4, Chloroform). ^{1}H NMR (400 MHz, Chloroform-*d*) δ 7.61 (d, *J* = 8.0 Hz, 2H), 7.52 (d, *J* = 7.9 Hz, 2H), 7.43 – 7.36 (m, 2H), 7.31 (td, *J* = 8.0, 6.2 Hz, 4H), 7.24 – 7.19 (m, 1H), 7.14 (d, *J* = 7.9 Hz, 2H), 6.61 – 6.43 (m, 1H), 6.32 (dt, *J* = 15.8, 6.9 Hz, 1H), 3.95 (td, *J* = 8.6, 7.5 Hz, 1H), 3.77 – 3.56 (m, 2H), 2.94 (d, *J* = 13.5 Hz, 1H), 2.84 (s, 2H), 2.71 (d, *J* = 13.5 Hz, 1H), 2.61 – 2.46 (m, 2H), 1.82 (ddd, *J* = 12.9, 7.5, 3.3 Hz, 1H), 1.70 – 1.59 (m, 1H). ^{13}C NMR (100 MHz, Chloroform-*d*) δ 142.6 (d, *J* = 1.5 Hz), 141.3 (d, *J* = 1.4 Hz), 137.4, 131.9, 131.2, 131.2, 128.9 (q, *J* = 32.3 Hz), 128.6, 127.3, 126.2,125.2 (q, *J* = 3.8 Hz), 125.1 (q, *J* = 3.8 Hz), 82.7, 64.9, 48.1, 39.7, 38.7, 33.1, 31.6. ^{19}F NMR (376 MHz, Chloroform-*d*) δ -62.27, -62.32. HRMS (ESI) calculated for [$C_{29}H_{26}F_6NaO$] [M+Na]$^+$: 527.1780 found: 527.1783. Enantiomeric excess was determined by HPLC with a Chiralpak AS-H column (hexanes: 2-propanol = 99: 1, 0.6 mL/min, 254 nm); major enantiomer tr = 10.681 min, minor enantiomer tr = 8.541 min.

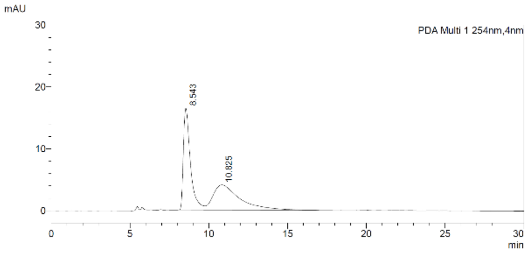

<Peak Table>
PDA Ch1 254nm

Peak#	Ret. Time	Height	Height%	Area	Area%
1	8.543	16420	80.220	503635	50.737
2	10.825	4049	19.780	488996	49.263
Total		20469	100.000	992631	100.000

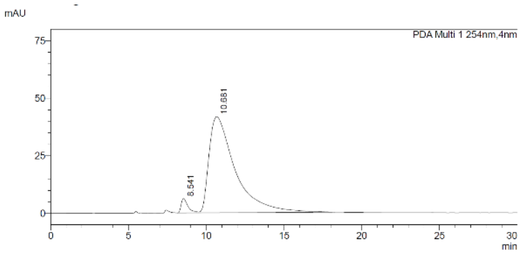

<Peak Table>
PDA Ch1 254nm

Peak#	Ret. Time	Height	Height%	Area	Area%
1	8.541	6211	12.964	185678	3.652
2	10.681	41698	87.036	4898546	96.348
Total		47908	100.000	5084224	100.000

4.3.6 Synthesis of (*R*)-2-cinnamyl-3,3-bis(4-methoxybenzyl)tetrahydrofuran (10f)

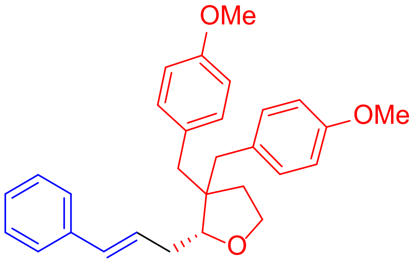

10f

Prepared according to typical procedure at -20 °C for 72 hours by using $[Pd(allyl)Cl]_2$ (2.5 mol%), **Xu9** (10 mol%) from γ-hydroxyalkenes (0.5 mmol) and alkenyl bromide (1 mmol), after flash column chromatography on a silica gel (petroleum ether: ethyl acetate = 50: 1) give the product **10f** as a white liquid (136 mg, 63% yield) with 90% *ee*. $[\alpha]_D^{20}$ = 45.9 (*c* = 0.4, Chloroform).^{1}H NMR (400 MHz, Chloroform-*d*) δ 7.31 – 7.25 (m, 2H), 7.18 (t, *J* = 7.6 Hz, 2H), 7.12 – 7.07 (m, 1H), 7.05 – 7.00 (m, 2H), 6.87 – 6.81 (m, 2H), 6.79 – 6.75 (m, 2H), 6.69 (dq, *J* = 9.6, 3.1, 2.7 Hz, 2H), 6.40 (d, *J* = 15.8 Hz, 1H), 6.23 (dt, *J* = 15.8, 6.9 Hz, 1H), 3.89 – 3.78 (m, 1H), 3.69 (s, 3H), 3.64 (s, 3H), 3.60 – 3.52 (m, 2H), 2.72 – 2.55 (m, 3H), 2.46 (d, *J* = 13.6 Hz, 1H), 2.42 – 2.31 (m, 2H), 1.70 (ddt, *J* = 14.8, 7.4, 3.7 Hz, 1H), 1.50 (dt, *J* = 11.9, 9.5 Hz, 1H). ^{13}C NMR (100 MHz, Chloroform-*d*) δ 158.3, 158.2, 137.7, 131.9, 131.8, 131.5, 130.6, 129.4, 128.5, 128.1, 127.1, 126.2, 113.7, 113.5, 82.8, 65.1, 55.3, 55.2, 55.2, 48.1, 39.0, 37.7, 33.1, 31.7. HRMS (ESI) calculated for $[C_{29}H_{32}NaO_3]$ $[M+Na]^+$: 451.2244 found: 451.2256. Enantiomeric excess was determined by HPLC with a Chiralpak AD-H column (hexanes: 2-propanol = 95: 5, 0.6 mL/min, 254 nm); major enantiomer tr = 22.581 min, minor enantiomer tr = 24.506 min.

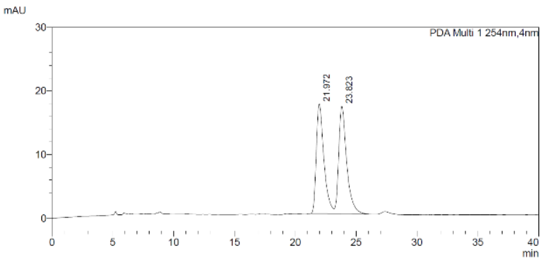

<Peak Table>

PDA Ch1 254nm

Peak#	Ret. Time	Height	Height%	Area	Area%
1	21.972	17214	50.456	746236	49.101
2	23.823	16903	49.544	773568	50.899
Total		34117	100.000	1519804	100.000

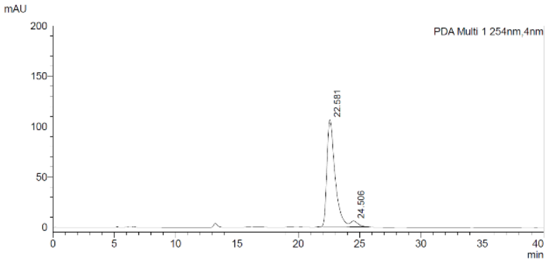

<Peak Table>

PDA Ch1 254nm

Peak#	Ret. Time	Height	Height%	Area	Area%
1	22.581	106664	94.602	4941485	94.967
2	24.506	6086	5.398	261889	5.033
Total		112750	100.000	5203374	100.000

4.3.7 Synthesis of (*R*)-2-cinnamyl-3,3-bis(3-methoxybenzyl)tetrahydrofuran (10g)

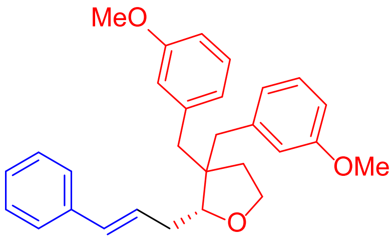

10g

Prepared according to typical procedure at -20 °C for 72 hours by using [Pd(allyl)Cl]$_2$ (2.5 mol%), **Xu9** (10 mol%) from γ-hydroxyalkenes (0.5 mmol) and alkenyl bromide (1 mmol), after flash column chromatography on a silica gel (petroleum ether: ethyl acetate = 50: 1) give the product **10g** as a white liquid (137 mg, 64% yield) with 90% *ee*. $[\alpha]_D^{20}$ = 56.4 (*c* = 0.4, Chloroform).^{1}H NMR (400 MHz, Chloroform-*d*) δ 7.39 – 7.31 (m, 2H), 7.25 (dt, *J* = 9.0, 7.6 Hz, 3H), 7.20 – 7.09 (m, 2H), 6.84 – 6.76 (m, 3H), 6.73 (ddd, *J* = 8.3, 2.6, 0.9 Hz, 1H), 6.63 (dt, *J* = 7.6, 1.2 Hz, 1H), 6.58 (dd, *J* = 2.6, 1.6 Hz, 1H), 6.49 (d, *J* = 15.9 Hz, 1H), 6.33 (dt, *J* = 15.8, 6.9 Hz, 1H), 3.94 (td, *J* = 8.7, 7.4 Hz, 1H), 3.78 (s, 3H), 3.72 – 3.63 (m, 5H), 2.86 – 2.71 (m, 3H), 2.60 (d, *J* = 13.4 Hz, 1H), 2.54 – 2.37 (m, 2H), 1.85 (ddd, *J* = 12.7, 7.4, 3.2 Hz, 1H), 1.73 – 1.59 (m, 1H). ^{13}C NMR (100 MHz, Chloroform-*d*) δ 159.5, 159.4, 140.3, 139.0, 137.6, 131.6, 129.2, 129.0, 128.5, 128.0, 127.1, 126.2, 123.5, 123.4, 117.2, 116.8, 111.8, 111.3, 83.0, 65.1, 55.2, 55.2, 48.0, 40.1, 38.8, 33.2, 32.0. HRMS (ESI) calculated for [$C_{29}H_{32}NaO_3$] [M+Na]$^+$: 451.2244 found: 451.2254. Enantiomeric excess was determined by HPLC with a Chiralpak AD-H column (hexanes: 2-propanol = 90: 10, 0.6 mL/min, 254 nm); major enantiomer tr = 13.800 min, minor enantiomer tr = 15.075 min..

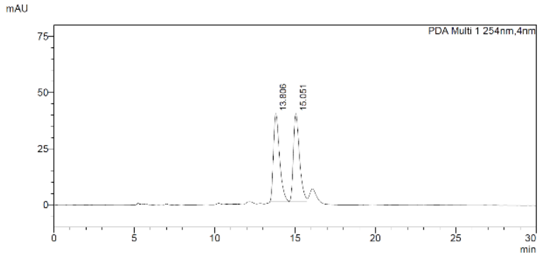

<Peak Table>

PDA Ch1 254nm

Peak#	Ret. Time	Height	Height%	Area	Area%
1	13.806	39336	49.966	1040769	50.028
2	15.051	39390	50.034	1039590	49.972
Total		78726	100.000	2080359	100.000

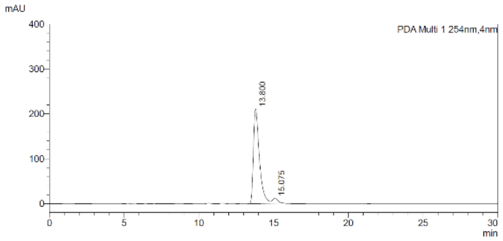

<Peak Table>

PDA Ch1 254nm

Peak#	Ret. Time	Height	Height%	Area	Area%
1	13.800	210524	94.878	5817507	95.059
2	15.075	11366	5.122	302387	4.941
Total		221890	100.000	6119893	100.000

4.3.8 Synthesis of (*R*)-2-cinnamyl-3,3-bis(2-methoxybenzyl)tetrahydrofuran (10h)

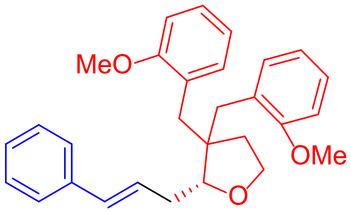

10h

Prepared according to typical procedure at -20 °C for 72 hours by using [Pd(allyl)Cl]$_2$ (2.5 mol%), **Xu9** (10 mol%) from γ-hydroxyalkenes (0.5 mmol) and alkenyl bromide (1 mmol), after flash column chromatography on a silica gel (petroleum ether: ethyl acetate = 50: 1) give the product **10h** as a white liquid (133 mg, 62% yield) with 90% *ee*. $[\alpha]_D^{20}$ =61.6 (c = 0.4, Chloroform).^{1}H NMR (400 MHz, Chloroform-*d*) δ 7.40 – 7.33 (m, 2H), 7.31 – 7.22 (m, 3H), 7.22 – 7.14 (m, 3H), 6.96 – 6.86 (m, 3H), 6.81 (ddd, J = 8.3, 4.3, 1.1 Hz, 2H), 6.53 (d, J = 15.9 Hz, 1H), 6.34 (dt, J = 15.8, 6.7 Hz, 1H), 4.04 (dt, J = 9.4, 7.4 Hz, 1H), 3.80 (s, 3H), 3.75 (s, 3H), 3.64 (ddd, J = 10.0, 7.7, 2.4 Hz, 1H), 3.52 (dd, J = 10.2, 1.6 Hz, 1H), 2.98 (dd, J = 15.0, 13.4 Hz, 2H), 2.80 (ddt, J = 14.8, 6.6, 1.7 Hz, 1H), 2.74 – 2.59 (m, 2H), 2.47 (dddd, J = 15.0, 10.2, 6.8, 1.4 Hz, 1H), 1.76 (ddd, J = 12.6, 7.2, 2.4 Hz, 1H), 1.67 – 1.60 (m, 1H). ^{13}C NMR (100 MHz, Chloroform-*d*) δ 158.4, 158.2, 137.9, 133.2, 133.0, 130.9, 129.2, 128.4, 127.6, 127.5, 127.5, 126.8, 126.2, 126.1, 120.1, 119.9, 110.5, 110.1, 82.9, 65.5, 55.1, 49.0, 33.4, 32.9, 32.2, 31.3. HRMS (ESI) calculated for [$C_{29}H_{32}NaO_3$] [M+Na]$^+$: 451.2244 found: 451.2255. Enantiomeric excess was determined by HPLC with a Chiralpak OD-H column (hexanes: 2-propanol = 97: 3, 0.6 mL/min, 254 nm); major enantiomer tr = 29.578 min, minor enantiomer tr = 33.929 min.

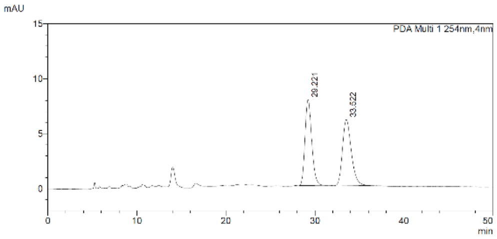

<Peak Table>
PDA Ch1 254nm

Peak#	Ret. Time	Height	Height%	Area	Area%
1	29.221	7816	56.628	408769	49.825
2	33.522	5987	43.372	411633	50.175
Total		13803	100.000	820402	100.000

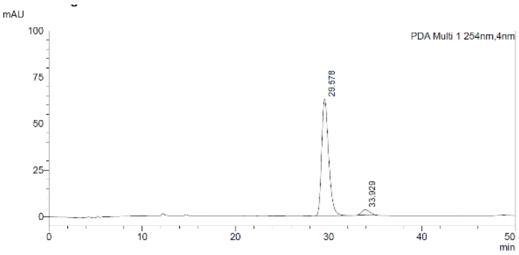

<Peak Table>
PDA Ch1 254nm

Peak#	Ret. Time	Height	Height%	Area	Area%
1	29.578	62662	95.659	3272914	94.933
2	33.929	2843	4.341	174688	5.067
Total		65506	100.000	3447603	100.000

4.3.9 Synthesis of (*R*)-3,3-diallyl-2-cinnamyltetrahydrofuran (10i)

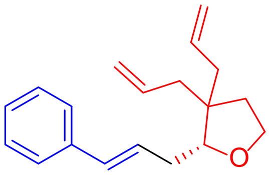

10i

Prepared according to typical procedure at -20 ºC for 72 hours by using $[Pd(allyl)Cl]_2$ (2.5 mol%), **Xu9** (10 mol%) from γ-hydroxyalkenes (0.5 mmol) and alkenyl bromide (1 mmol), after flash column chromatography on a silica gel (petroleum ether: ethyl acetate = 50: 1) give the product **10i** as a white liquid (95 mg, 71% yield) with 90% *ee*. $[\alpha]_D^{20}$ = 15.4 (*c* = 0.4, Chloroform). ^{1}H NMR (400 MHz, Chloroform-*d*) δ 7.37 – 7.33 (m, 2H), 7.30 – 7.25 (m, 2H), 7.22 – 7.11 (m, 1H), 6.52 – 6.38 (m, 1H), 6.29 (dt, *J* = 15.8, 6.9 Hz, 1H), 5.84 (tddd, *J* = 17.6, 14.4, 9.3, 7.2 Hz, 2H), 5.16 – 5.04 (m, 4H), 3.89 (td, *J* = 8.3, 6.5 Hz, 1H), 3.81 – 3.70 (m, 1H), 3.65 (dd, *J* = 8.1, 4.9 Hz, 1H), 2.44 – 2.32 (m, 2H), 2.24 – 2.12 (m, 4H), 1.93 – 1.82 (m, 1H), 1.81 – 1.72 (m, 1H). ^{13}C NMR (100MHz, Chloroform-*d*) δ 137.6, 135.1, 134.5, 131.4, 128.4, 128.0, 127.0, 126.1, 126.1, 118.0, 117.7, 85.2, 65.5, 46.7, 40.9, 37.7, 35.5, 34.1. HRMS (ESI) calculated for $[C_{19}H_{24}NaO]$ $[M+Na]^+$: 291.1719 found: 291.1717. Enantiomeric excess was determined by HPLC with a Chiralpak OD-H column (hexanes: 2-propanol = 95: 5, 0.6 mL/min, 254 nm); major enantiomer tr = 8.483 min, minor enantiomer tr = 16.925 min.

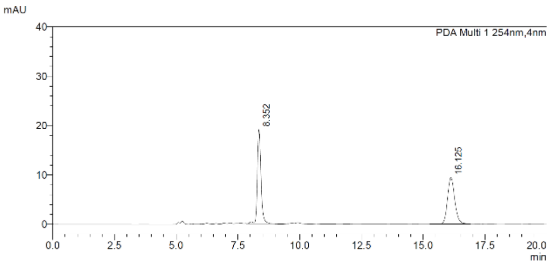

<Peak Table>
PDA Ch1 254nm

Peak#	Ret. Time	Height	Height%	Area	Area%
1	8.352	19102	66.971	189661	50.072
2	16.125	9421	33.029	189118	49.928
Total		28523	100.000	378779	100.000

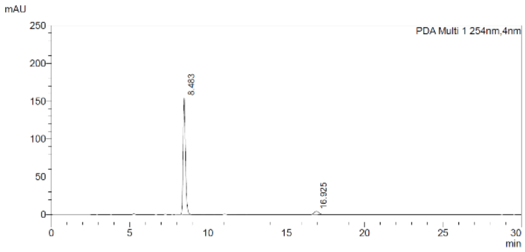

<Peak Table>
PDA Ch1 254nm

Peak#	Ret. Time	Height	Height%	Area	Area%
1	8.483	153914	97.435	1616107	95.094
2	16.925	4051	2.565	83379	4.906
Total		157966	100.000	1699486	100.000

4.3.10 Synthesis of (*R*)-2-cinnamyl-3,3-diphenyltetrahydrofuran (10j)

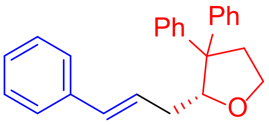

10j

Prepared according to typical procedure at -20 ºC for 72 hours by using $[Pd(allyl)Cl]_2$ (2.5 mol%), **Xu9** (10 mol%) from γ-hydroxyalkenes (0.5 mmol) and alkenyl bromide (1 mmol), after flash column chromatography on a silica gel (petroleum ether: ethyl acetate = 50: 1) give the product **10j** as a white liquid (119 mg, 71% yield) with 91% *ee*. $[\alpha]_D^{20}$ = 110.4 (*c* = 0.4, Chloroform). ^{1}H NMR (400 MHz, Chloroform-*d*) δ 7.32 – 7.25 (m, 9H), 7.23 – 7.13 (m, 4H), 7.11 – 7.06 (m, 2H), 6.28 – 6.18 (m, 2H), 4.68 (dd, *J* = 9.6, 2.8 Hz, 1H), 4.19 (td, *J* = 8.7, 3.3 Hz, 1H), 3.82 (q, *J* = 8.4 Hz, 1H), 2.94 (dt, *J* = 12.4, 8.8 Hz, 1H), 2.30 (ddd, *J* = 12.3, 7.9, 3.4 Hz, 1H), 2.22 – 2.10 (m, 1H), 1.85 (ddd, *J* = 14.2, 9.6, 4.9 Hz, 1H). ^{13}C NMR (100 MHz, Chloroform-*d*) δ 147.5, 145.0, 137.7, 131.5, 128.8, 128.6, 128.5, 128.3, 128.2, 128.1, 128.1, 127.9, 127.8, 127.0, 126.5, 126.3, 126.1, 84.1, 66.0, 58.2, 39.2, 37.1. HRMS (ESI) calculated for $[C_{25}H_{24}NaO]$ $[M+Na]^+$: 363.1719 found: 363.1712. Enantiomeric excess was determined by HPLC with a Chiralpak OD-H column (hexanes: 2-propanol = 95: 5, 0.6 mL/min, 254 nm); major enantiomer tr = 10.471 min, minor enantiomer tr = 18.737 min.

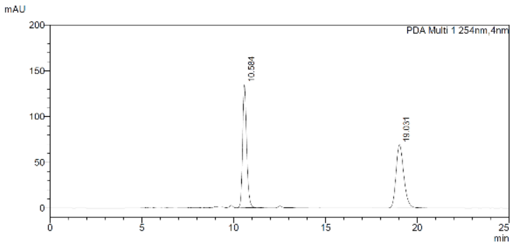

<Peak Table>

PDA Ch1 254nm

Peak#	Ret. Time	Height	Height%	Area	Area%
1	10.584	134501	65.990	2072740	50.559
2	19.031	69318	34.010	2026878	49.441
Total		203819	100.000	4099618	100.000

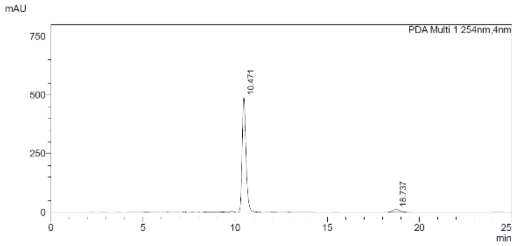

<Peak Table>

PDA Ch1 254nm

Peak#	Ret. Time	Height	Height%	Area	Area%
1	10.471	489109	97.569	7129223	95.618
2	18.737	12189	2.431	326737	4.382
Total		501298	100.000	7455960	100.000

4.3.11 Synthesis of (*R*)-2-cinnamyl-3,3-dimethyltetrahydrofuran (10k)

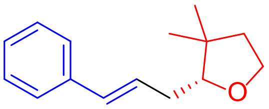

10k

Prepared according to typical procedure at -20 ºC for 72 hours by using $[Pd(allyl)Cl]_2$ (2.5 mol%), **Xu9** (10 mol%) from γ-hydroxyalkenes (0.5 mmol) and alkenyl bromide (1 mmol), after flash column chromatography on a silica gel (petroleum ether: ethyl acetate = 50: 1) give the product **10k**

as a white liquid (72 mg, 67% yield) with 90% *ee*. $[\alpha]_D^{20}$ = 23.8 (*c* = 0.4, Chloroform). ^{1}H NMR (400 MHz, Chloroform-*d*) δ 7.37 – 7.32 (m, 2H), 7.31 – 7.23 (m, 2H), 7.21 – 7.14 (m, 1H), 6.47 (dt, *J* = 15.9, 1.5 Hz, 1H), 6.29 (dt, *J* = 15.8, 7.0 Hz, 1H), 3.90 (q, *J* = 8.1 Hz, 1H), 3.80 (td, *J* = 8.7, 4.6 Hz, 1H), 3.46 (dd, *J* = 7.6, 5.3 Hz, 1H), 2.32 (tt, *J* = 7.0, 1.3 Hz, 2H), 1.84 – 1.68 (m, 2H), 1.07 (s, 3H), 0.97 (s, 3H). ^{13}C NMR (100 MHz, Chloroform-*d*) δ 137.7, 131.3, 128.4, 128.1, 126.9, 126.1, 86.6, 65.4, 41.4, 40.5, 34.0, 25.5, 21.7. HRMS (ESI) calculated for [$C_{15}H_{20}NaO$] [M+Na]$^+$: 239.1406 found: 239.1409. Enantiomeric excess was determined by HPLC with a Chiralpak OD-H column (hexanes: 2-propanol = 95: 5, 0.6 mL/min, 254 nm); major enantiomer tr = 8.520 min, minor enantiomer tr = 13.746 min.

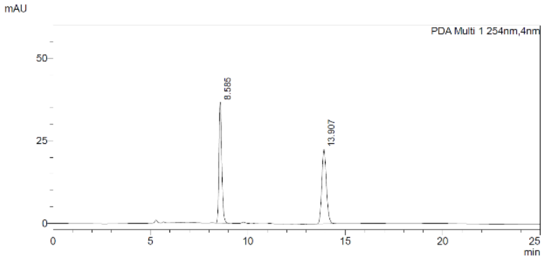

<Peak Table>
PDA Ch1 254nm

Peak#	Ret. Time	Height	Height%	Area	Area%
1	8.585	36606	61.864	368848	49.963
2	13.907	22566	38.136	369388	50.037
Total		59171	100.000	738236	100.000

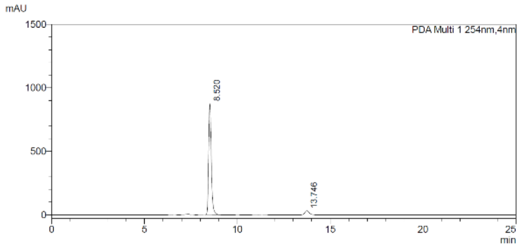

<Peak Table>
PDA Ch1 254nm

Peak#	Ret. Time	Height	Height%	Area	Area%
1	8.520	874754	96.683	8651106	95.126
2	13.746	30012	3.317	443225	4.874
Total		904765	100.000	9094331	100.000

4.3.12 Synthesis of (*R*)-2-cinnamyl-3,3-diethyltetrahydrofuran (10l)

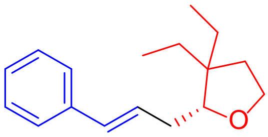

10l

Prepared according to typical procedure at -20 °C for 72 hours by using $[Pd(allyl)Cl]_2$ (2.5 mol%), **Xu9** (10 mol%) from γ-hydroxyalkenes (0.5 mmol) and alkenyl bromide (1 mmol), after flash column chromatography on a silica gel (petroleum ether: ethyl acetate = 50: 1) give the product **10l** as a white liquid (84 mg, 69% yield) with 88% *ee*. $[\alpha]_D^{20}$ = 34.9 (*c* = 0.4, Chloroform). ^{1}H NMR (400 MHz, Chloroform-*d*) δ 7.39 – 7.31 (m, 2H), 7.26 (t, *J* = 7.7 Hz, 2H), 7.19 – 7.12 (m, 1H), 6.44 (d, *J* = 15.9 Hz, 1H), 6.32 (dt, *J* = 15.8, 6.8 Hz, 1H), 3.87 (td, *J* = 8.3, 5.8 Hz, 1H), 3.73 (td, *J* = 8.4, 6.6 Hz, 1H), 3.62 (dd, *J* = 7.7, 5.3 Hz, 1H), 2.33 (td, *J* = 7.1, 6.6, 1.5 Hz, 2H), 1.73 (dddd, *J* = 33.5, 12.4, 8.4, 6.1 Hz, 2H), 1.50 – 1.34 (m, 4H), 0.88 (dt, *J* = 9.2, 7.5 Hz, 6H). ^{13}C NMR (100 MHz, Chloroform-*d*) δ 137.7, 131.2, 128.5, 128.4, 126.9, 126.1, 85.7, 65.6, 47.1, 36.1, 34.7, 28.3, 24.5, 9.1, 9.0. HRMS (ESI) calculated for [$C_{17}H_{24}NaO$] [M+Na]$^+$: 267.1719 found: 267.1721.

Enantiomeric excess was determined by HPLC with a Chiralpak OD-H column (hexanes: 2-propanol = 95: 5, 0.6 mL/min, 254 nm); major enantiomer tr = 8.175 min, minor enantiomer tr = 12.139 min..

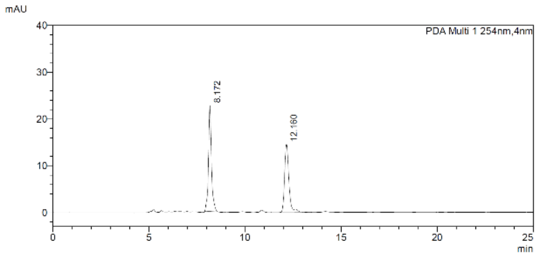

<Peak Table>
PDA Ch1 254nm

Peak#	Ret. Time	Height	Height%	Area	Area%
1	8.172	22706	60.773	248963	51.955
2	12.160	14656	39.227	230231	48.045
Total		37361	100.000	479194	100.000

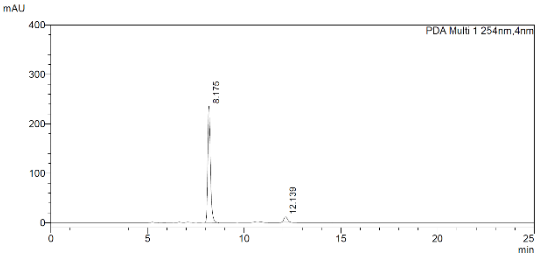

<Peak Table>
PDA Ch1 254nm

Peak#	Ret. Time	Height	Height%	Area	Area%
1	8.175	236057	95.566	2319162	94.136
2	12.139	10953	4.434	144471	5.864
Total		247011	100.000	2463633	100.000

4.3.13 Synthesis of (*R*)-1-cinnamyl-2-oxaspiro[4.4]nonane (10m)

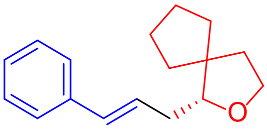

10m

Prepared according to typical procedure at -20 °C for 72 hours by using $[Pd(allyl)Cl]_2$ (2.5 mol%), **Xu9** (10 mol%) from γ-hydroxyalkenes (0.5 mmol) and alkenyl bromide (1 mmol), after flash column chromatography on a silica gel (petroleum ether: ethyl acetate = 50: 1) give the product **10m** as a white liquid (79 mg, 65% yield) with 85% *ee*. $[\alpha]_D^{20}$ = 31.3 (*c* = 0.4, Chloroform). ^1H NMR (400 MHz, Chloroform-*d*) δ 7.37 – 7.32 (m, 2H), 7.26 (dd, *J* = 8.4, 6.8 Hz, 2H), 7.20 – 7.12 (m, 1H), 6.55 – 6.39 (m, 1H), 6.30 (dt, *J* = 15.8, 6.9 Hz, 1H), 3.84 (dtd, *J* = 27.5, 8.2, 6.4 Hz, 2H), 3.70 (dd, *J* = 7.3, 5.5 Hz, 1H), 2.33 (td, *J* = 6.3, 5.6, 1.5 Hz, 2H), 1.79 (qdd, *J* = 12.0, 7.8, 6.4 Hz, 2H), 1.64 (ddddd, *J* = 8.5, 7.2, 4.9, 3.7, 2.0 Hz, 5H), 1.59 – 1.52 (m, 2H), 1.38 (tq, *J* = 5.5, 2.4 Hz, 1H). ^{13}C NMR (100 MHz, Chloroform-*d*) δ 137.7, 131.4, 128.4, 128.1, 126.9, 126.1, 84.9, 66.0, 52.6, 39.8, 36.5, 35.2, 32.2, 24.9, 24.0. HRMS (ESI) calculated for $[C_{17}H_{22}NaO]$ $[M+Na]^+$: 265.1563 found: 265.1566. Enantiomeric excess was determined by HPLC with a Chiralpak OD-H column (hexanes: 2-propanol = 95: 5, 0.6 mL/min, 254 nm); major enantiomer tr = 9.612 min, minor enantiomer tr = 16.531 min.

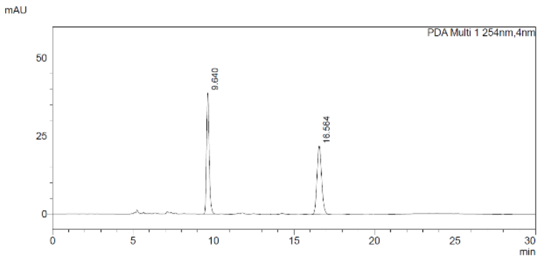

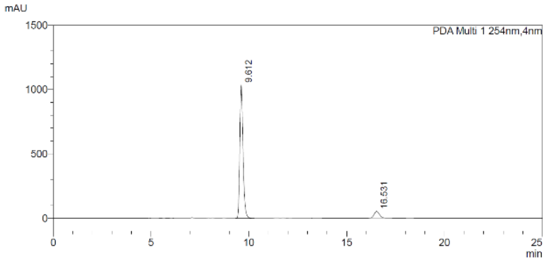

<Peak Table>
PDA Ch1 254nm

Peak#	Ret. Time	Height	Height%	Area	Area%
1	9.640	38913	63.852	444762	50.178
2	16.564	22030	36.148	441605	49.822
Total		60943	100.000	886367	100.000

<Peak Table>
PDA Ch1 254nm

Peak#	Ret. Time	Height	Height%	Area	Area%
1	9.612	1034020	95.316	11820743	92.597
2	16.531	50814	4.684	945084	7.403
Total		1084834	100.000	12765827	100.000

4.3.14 Synthesis of (*R*)-1-cinnamyl-2-oxaspiro[4.5]decane (10n)

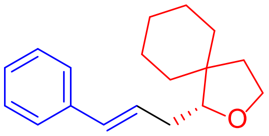

10n

Prepared according to typical procedure at -20 ºC for 72 hours by using [Pd(allyl)Cl]$_2$ (2.5 mol%), **Xu9** (10 mol%) from γ-hydroxyalkenes (0.5 mmol) and alkenyl bromide (1 mmol), after flash column chromatography on a silica gel (petroleum ether: ethyl acetate = 50: 1) give the product **10n** as a white liquid (86 mg, 67% yield) with 87% *ee*. $[\alpha]_D^{20}$ = 33.7 (*c* = 0.4, Chloroform).^{1}H NMR (400 MHz, Chloroform-*d*) δ 7.40 – 7.31 (m, 2H), 7.26 (dd, J = 8.5, 6.8 Hz, 2H), 7.21 – 7.11 (m, 1H), 6.51 – 6.41 (m, 1H), 6.31 (dt, *J* = 15.8, 6.9 Hz, 1H), 3.90 (td, *J* = 8.3, 6.5 Hz, 1H), 3.76 (td, *J* = 8.7, 5.9 Hz, 1H), 3.44 (dd, *J* = 7.2, 5.8 Hz, 1H), 2.32 (td, *J* = 6.3, 5.7, 1.4 Hz, 2H), 1.95 (ddd, *J* = 12.4, 8.2, 5.9 Hz, 1H), 1.70 – 1.57 (m, 4H), 1.50 – 1.41 (m, 1H), 1.40 – 1.32 (m, 4H), 1.31 – 1.13 (m, 2H). ^{13}C NMR (100 MHz, Chloroform-*d*) δ 137.7, 131.3, 128.4, 126.9, 126.1, 87.3, 65.7, 44.8, 35.7, 35.4, 33.9, 30.3, 26.5, 24.0, 23.0. HRMS (ESI) calculated for [$C_{18}H_{24}NaO$] [M+Na]$^+$: 279.1719 found: 279.1720. Enantiomeric excess was determined by HPLC with a Chiralpak OD-H column (hexanes: 2-propanol = 95: 5, 0.6 mL/min, 254 nm); major enantiomer tr = 9.748 min, minor enantiomer tr = 21.555 min.

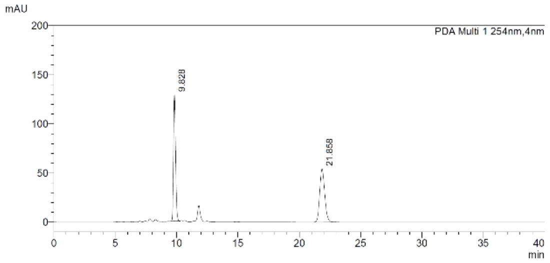

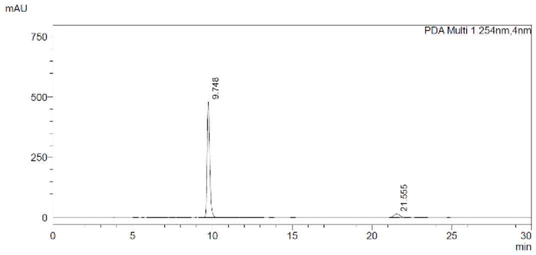

<Peak Table>
PDA Ch1 254nm

Peak#	Ret. Time	Height	Height%	Area	Area%
1	9.828	129058	70.296	1524000	49.767
2	21.858	54535	29.704	1538260	50.233
Total		183593	100.000	3062260	100.000

<Peak Table>
PDA Ch1 254nm

Peak#	Ret. Time	Height	Height%	Area	Area%
1	9.748	480932	96.987	5579582	93.556
2	21.555	14943	3.013	384333	6.444
Total		495875	100.000	5963916	100.000

4.3.15 Synthesis of (*R*)-2-cinnamyl-4,4-dimethyltetrahydrofuran (10o)

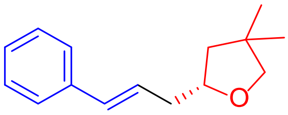

10o

Prepared according to typical procedure at 0 ºC for 72 hours by using $[Pd(allyl)Cl]_2$ (2.5 mol%), **Xu9** (10 mol%) from γ-hydroxyalkenes (0.5 mmol) and alkenyl bromide (1 mmol), after flash column chromatography on a silica gel (petroleum ether: ethyl acetate = 50: 1) give the product **10o** as a white liquid (78 mg, 72% yield) with 87% *ee*. $[\alpha]_D^{20}$ = -4.3 (*c* = 0.4, Chloroform). ^{1}H NMR (400 MHz, Chloroform-*d*) δ 7.37 – 7.32 (m, 2H), 7.28 (dd, *J* = 8.5, 6.8 Hz, 2H), 7.22 – 7.16 (m, 1H), 6.45 (dt, *J* = 15.9, 1.4 Hz, 1H), 6.22 (dt, *J* = 15.8, 7.1 Hz, 1H), 4.13 (dq, *J* = 9.0, 6.4 Hz, 1H), 3.55 (d, *J* = 8.1 Hz, 1H), 3.47 (d, *J* = 8.1 Hz, 1H), 2.53 (dtd, *J* = 13.6, 6.7, 1.4 Hz, 1H), 2.41 (dddd, *J* = 14.0, 7.4, 6.2, 1.4 Hz, 1H), 1.80 (dd, J = 12.2, 6.5 Hz, 1H), 1.44 (dd, *J* = 12.3, 9.0 Hz, 1H), 1.10 (d, *J* = 3.6 Hz, 6H). ^{13}C NMR (100 MHz, Chloroform-*d*) δ 137.6, 132.0, 128.5, 127.0, 126.7, 126.1, 80.2, 78.9, 46.4, 39.8, 39.7, 26.9, 26.6. HRMS (ESI) calculated for $[C_{15}H_{20}NaO]$ $[M+Na]^+$: 239.1406 found: 239.1412. Enantiomeric excess was determined by HPLC with a Chiralpak OD-H column (hexanes: 2-propanol = 95: 5, 0.6 mL/min, 254 nm); major enantiomer tr = 7.962 min, minor enantiomer tr = 8.298 min.

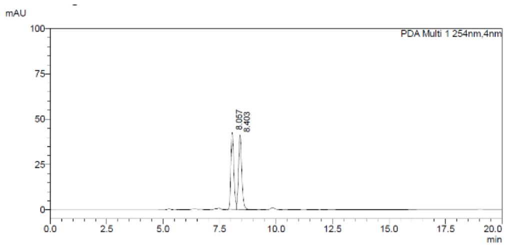

<Peak Table>

PDA Ch1 254nm

Peak#	Ret. Time	Height	Height%	Area	Area%
1	8.057	42410	50.693	383037	48.545
2	8.403	41249	49.307	406000	51.455
Total		83659	100.000	789036	100.000

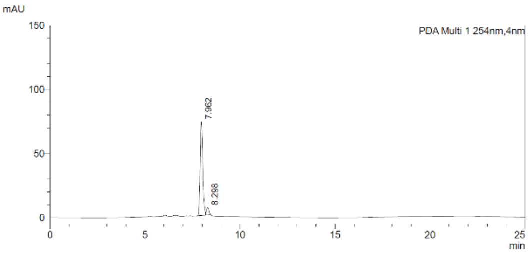

<Peak Table>

PDA Ch1 254nm

Peak#	Ret. Time	Height	Height%	Area	Area%
1	7.962	73064	92.824	692291	93.452
2	8.298	5648	7.176	48511	6.548
Total		78712	100.000	740802	100.000

4.3.16 Synthesis of (*R*)-7-cinnamyl-6-oxaspiro[3.4]octane (10p)

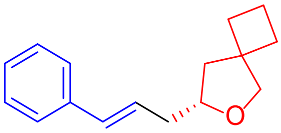

10p

Prepared according to typical procedure at 0 ºC for 72 hours by using $[Pd(allyl)Cl]_2$ (2.5 mol%), **Xu9** (10 mol%) from γ-hydroxyalkenes (0.5 mmol) and alkenyl bromide (1 mmol), after flash column chromatography on a silica gel (petroleum ether: ethyl acetate = 50: 1) give the product **10p**

as a white liquid (84 mg, 74% yield) with 82% *ee*. $[\alpha]_D^{20}$ = -7.9 (*c* = 0.4, Chloroform). ^{1}H NMR (400 MHz, Chloroform-*d*) δ 7.37 – 7.32 (m, 2H), 7.28 (dd, *J* = 8.5, 6.8 Hz, 2H), 7.22 – 7.16 (m, 1H), 6.44 (dt, *J* = 15.9, 1.4 Hz, 1H), 6.21 (dt, *J* = 15.9, 7.2 Hz, 1H), 4.01 (dq, *J* = 8.4, 6.2 Hz, 1H), 3.80 (d, *J* = 8.4 Hz, 1H), 3.73 (d, *J* = 8.4 Hz, 1H), 2.49 (dddd, *J* = 13.5, 7.3, 6.3, 1.4 Hz, 1H), 2.39 (dddd, *J* = 14.0, 7.4, 6.2, 1.4 Hz, 1H), 2.12 – 1.95 (m, 5H), 1.91 – 1.79 (m, 2H), 1.69 – 1.60 (m, 1H).^{13}C NMR (100 MHz, Chloroform-*d*) δ 137.6, 132.0, 128.5, 127.0, 126.7, 126.1, 79.1, 78.4, 46.2, 44.8, 39.5, 33.1, 31.5, 16.5. HRMS (ESI) calculated for $[C_{16}H_{20}NaO]$ $[M+Na]^+$: 251.1406 found: 251.1412. Enantiomeric excess was determined by HPLC with a Chiralpak OD-H column (hexanes: 2-propanol = 95: 5, 0.6 mL/min, 254 nm); major enantiomer tr = 9.276 min, minor enantiomer tr = 8.883 min.

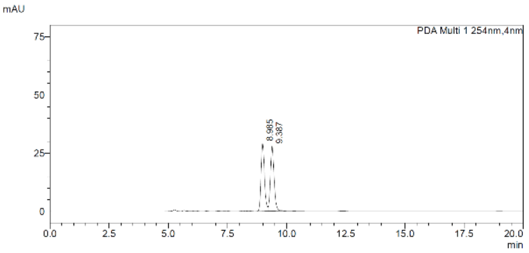

<Peak Table>
PDA Ch1 254nm

Peak#	Ret. Time	Height	Height%	Area	Area%
1	8.985	29084	50.994	295513	49.421
2	9.387	27950	49.006	302442	50.579
Total		57034	100.000	597955	100.000

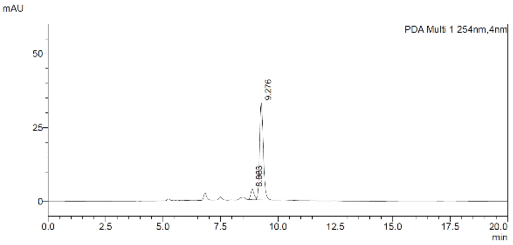

<Peak Table>
PDA Ch1 254nm

Peak#	Ret. Time	Height	Height%	Area	Area%
1	8.883	3582	9.832	36586	9.100
2	9.276	32851	90.168	365454	90.900
Total		36433	100.000	402040	100.000

4.3.17 Synthesis of (*S*)-2-cinnamyl-2-methyltetrahydrofuran (10q)

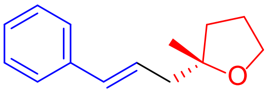

10q

Prepared according to typical procedure at 0 °C for 72 hours by using $[Pd(allyl)Cl]_2$ (2.5 mol%), **Xu9** (10 mol%) from γ-hydroxyalkenes (0.5 mmol) and alkenyl bromide (1 mmol), after flash column chromatography on a silica gel (petroleum ether: ethyl acetate = 50: 1) give the product **10q** as a white liquid (70 mg, 69% yield) with 81% *ee*. $[\alpha]_D^{20}$ = 7.0 (*c* = 0.4, Chloroform). ^{1}H NMR (400 MHz, Chloroform-*d*) δ 7.39 – 7.33 (m, 2H), 7.29 (dd, *J* = 8.5, 6.8 Hz, 2H), 7.22 – 7.16 (m, 1H), 6.51 – 6.37 (m, 1H), 6.25 (dt, *J* = 15.8, 7.3 Hz, 1H), 3.93 – 3.76 (m, 2H), 2.41 (dd, *J* = 7.4, 1.2 Hz, 2H), 1.99 – 1.79 (m, 3H), 1.65 (ddd, *J* = 11.6, 5.6, 2.6 Hz, 1H), 1.23 (s, 3H). ^{13}C NMR (100 MHz, Chloroform-*d*) δ 137.7, 132.5, 128.5, 127.0, 126.8, 126.1, 82.6, 67.5, 44.9, 36.3, 26.3, 26.2. HRMS (ESI) calculated for [C14H18NaO] $[M+Na]^+$: 225.1250 found: 225.1255. Enantiomeric excess was

determined by HPLC with a Chiralpak OD-H column (hexanes: 2-propanol = 99: 1, 0.5 mL/min, 254 nm); major enantiomer tr = 13.055 min, minor enantiomer tr = 17.859 min.

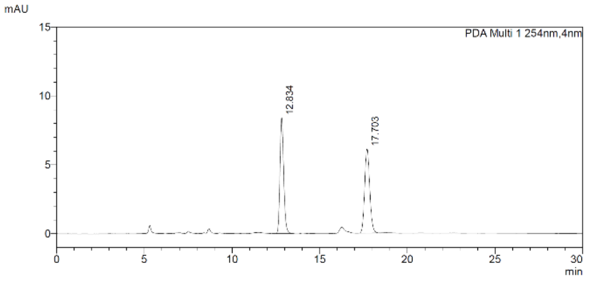

<Peak Table>

PDA Ch1 254nm

Peak#	Ret. Time	Height	Height%	Area	Area%
1	12.834	8430	57.949	124878	50.497
2	17.703	6117	42.051	122418	49.503
Total		14547	100.000	247297	100.000

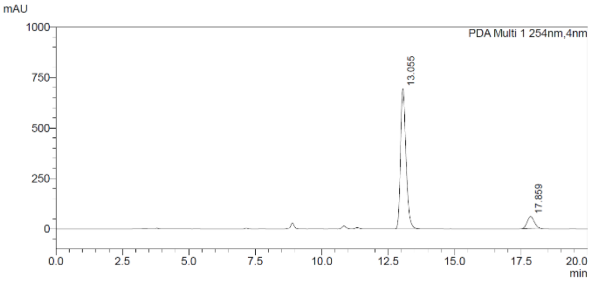

<Peak Table>

PDA Ch1 254nm

Peak#	Ret. Time	Height	Height%	Area	Area%
1	13.055	694608	92.226	9920559	90.364
2	17.859	58553	7.774	1057932	9.636
Total		753162	100.000	10978491	100.000

4.4 (*S*, *Z*)-2-(3-phenylallyl)-1-oxaspiro[4.5]decane (*Z*-3b)

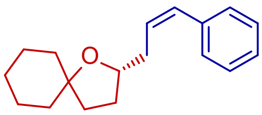

Z-3b

Prepared according to typical procedure at RT for 72 hours by using $[Pd(allyl)Cl]_2$ (5 mol%), **Xu2** (20 mol%) from γ-hydroxyalkenes (0.2 mmol) and alkenyl bromide (0.4 mmol), after flash column chromatography on a silica gel (petroleum ether: ethyl acetate = 80: 1) give the product ***Z*-3b** as a white liquid (29 mg, 57% yield) with 88% *ee*. $[\alpha]_D^{20}$= -34.9 (*c* = 0.4, Chloroform).^{1}H NMR (400 MHz, Chloroform-*d*)δ 7.34 – 7.28 (m, 4H), 7.21 (ddd, J = 6.7, 5.7, 2.6 Hz, 1H), 6.50 (dt, J = 11.7, 1.7 Hz, 1H), 5.73 (dt, J = 11.8, 7.2 Hz, 1H), 4.05 (dq, J = 12.4, 6.2 Hz, 1H), 2.73 – 2.59 (m, 1H), 2.49 (dtd, J = 8.6, 6.9, 1.8 Hz, 1H), 2.04 – 1.92 (m, 1H), 1.75 – 1.62 (m, 4H), 1.57 – 1.42 (m, 5H), 1.42 – 1.23 (m, 4H). ^{13}C NMR (100 MHz, Chloroform-*d*) δ 137.6, 130.3, 128.8, 128.7, 128.1, 126.5, 100.0, 82.8, 38.5, 37.6, 35.8, 35.3, 31.0, 25.7, 24.2, 23.8. HRMS (ESI) calculated for $[C_{18}H_{24}NaO]$ $[M+Na]^+$: 279.1720 found: 279.1719. Enantiomeric excess was determined by HPLC with a Chiralpak ADH+ADH column (hexanes: 2-propanol = 99: 1, 0.6 mL/min, 254 nm); major enantiomer tr = 13.126. min, minor enantiomer tr = 12.777 min.

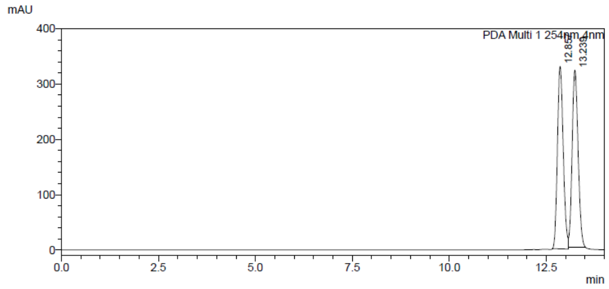

<Peak Table>
PDA Ch1 254nm

Peak#	Ret. Time	Height	Height%	Area	Area%
1	12.857	330006	50.706	3461776	50.357
2	13.239	320813	49.294	3412760	49.643
Total		650819	100.000	6874536	100.000

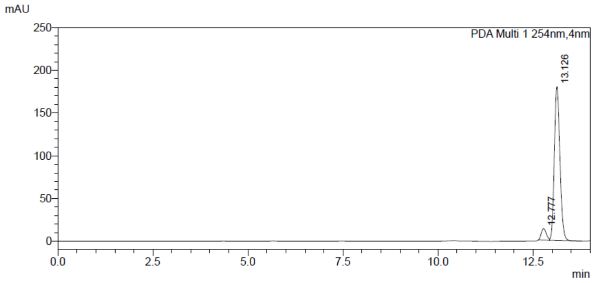

<Peak Table>
PDA Ch1 254nm

Peak#	Ret. Time	Height	Height%	Area	Area%
1	12.777	13371	6.919	123072	6.266
2	13.126	179866	93.081	1841196	93.734
Total		193237	100.000	1964269	100.000

4.5 Gram-scale synthesis of 6c:

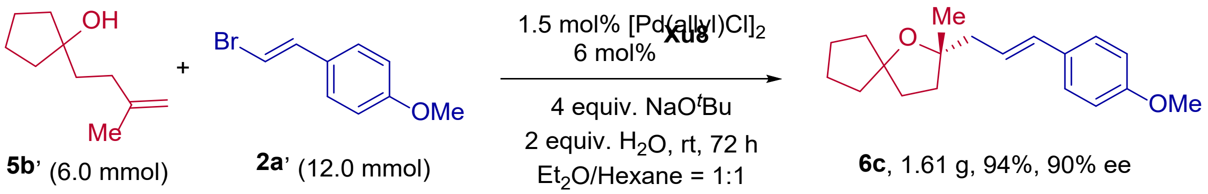

To a 100 mL tube was added [Pd(allyl)Cl]$_2$ (0.09 mmol, 32.9 mg, 1.5 mol%), **Xu8** (0.36 mmol, 234.8 mg, 6 mol%). The flask was evacuated and refilled with argon. Then tertiary γ-hydroxyalkenes **5b** (6 mmol), alkenyl halides **2a** (12 mmol), NaO^{t}Bu (24 mmol, 2.3g, 4.0 equiv.), H_2O (12 mmol, 324 mg, 2.0 equiv.) and a mixed solution of Et_2O/Hexane (1: 1, 60 mL) was added to the tube, and stirred at room temperature for 72 hours. After the reaction was complete (monitored by TLC), solvent was removed under reduced pressure. The crude product was then purified by flash column chromatography on silica gel to afford the desired product **6c** (1.61g, 94%, 90% ee).

4.6 Procedure for synthetic applications

4.6.1 (*R*)-2-(3-(4-methoxyphenyl)propyl)-2-methyl-1-oxaspiro[4.4]nonane (11)

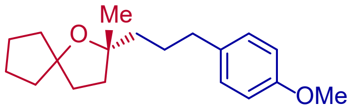

11

To a solution of **(*S*)-5c** (0.1 mmol, 52.2 mg) in MeOH (1 mL) was added 10% Pd/C (1 mol%, 1 mg) at room temperature. The reaction flask was evacuated twice under reduced pressure, and a H_2

balloon was placed on the top. After stirring at room temperature for 3.5 h, the mixture was concentrated and the residue was purified by column chromatography on a silica gel column(hexanes/EtOAc=80/1) to afford the desired product **11** (25.5mg, 89% yield) with 90% *ee*. $[\alpha]_D^{20}$= -9.3 (*c* = 0.4, Chloroform). ^{1}H NMR (400 MHz, Chloroform-*d*) δ 7.09 (d, J = 8.6 Hz, 2H), 6.84 – 6.75 (m, 2H), 3.78 (s, 3H), 2.54 (t, J = 7.4 Hz, 2H), 1.88 – 1.56 (m, 12H), 1.50 (ddd, J = 13.5, 8.1, 3.9 Hz, 4H), 1.17 (s, 3H). ^{13}C NMR (100 MHz, Chloroform-*d*) δ 157.7, 134.8, 129.3, 113.7, 91.0, 82.6, 55.3, 42.1, 39.8, 39.6, 37.4, 37.0, 35.6, 27.5, 27.0, 24.0, 23.9. HRMS (ESI) calculated for [$C_{19}H_{28}NaO_2$] [M+Na]$^+$: 311.1982 found: 311.1977. Enantiomeric excess was determined by HPLC with a Chiralpak ADH+ ADH column (hexanes: 2-propanol = 99: 1, 0.6 mL/min, 254 nm); major enantiomer tr = 14.356 min, minor enantiomer tr = 13.818 min.

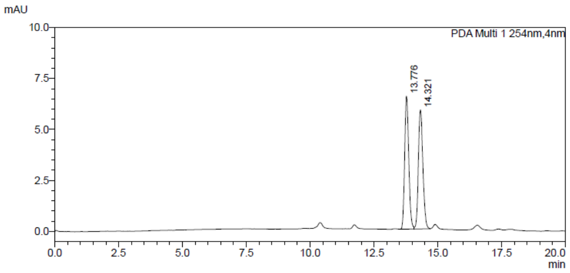

<Peak Table>
PDA Ch1 254nm

Peak#	Ret. Time	Height	Height%	Area	Area%
1	13.776	6521	52.773	72667	50.212
2	14.321	5836	47.227	72053	49.788
Total		12357	100.000	144720	100.000

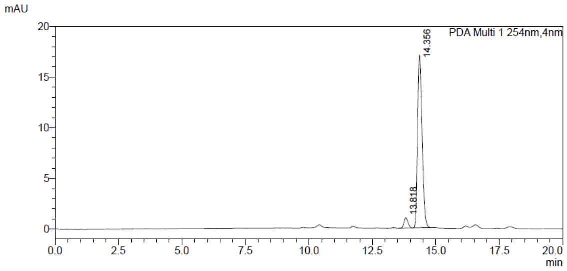

<Peak Table>
PDA Ch1 254nm

Peak#	Ret. Time	Height	Height%	Area	Area%
1	13.818	1023	5.654	11318	5.038
2	14.356	17068	94.346	213343	94.962
Total		18090	100.000	224661	100.000

4.6.2 1-((*S, E*)-3-(4-methoxyphenyl)-1-((*S*)-2-methyl-1-oxaspiro[4.4]nonan-2-yl)allyl)-1H-pyrazole (12)

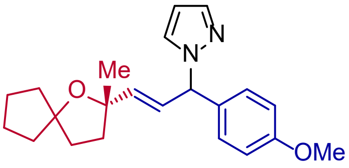

12

To a solution of 1, 4-dioxane (1.0 mL) was added ***(R)*-5c** (0.2 mmol), (0.24 mmol) and DDQ (0.28 mmol). The reaction mixture was stirred at room temperature for 8 h and then the solvent was removed under vacuum. The residue was purified by column chromatography on a silica gel column(hexanes/EtOAc=80/1) to afford the desired product **12** as a white liquid (62 mg, 88% yield) with 1.2:1 dr. $[\alpha]_D^{20}$= -12.6 (*c* = 0.4, Chloroform). ^{1}H NMR (400 MHz, Chloroform-*d*) δ 7.56 – 7.47 (m, 1H), 7.38 (dd, J = 4.3, 2.3 Hz, 1H), 7.09 (dt, J = 4.5, 2.2 Hz, 2H), 6.85 (dd, J = 8.7, 1.0 Hz, 2H),

6.25 (dd, J = 4.1, 2.1 Hz, 1H), 6.17 (ddd, J = 15.3, 6.7, 1.2 Hz, 1H), 5.99 (d, J = 6.6 Hz, 1H), 5.56 (ddd, J = 15.3, 4.8, 1.3 Hz, 1H), 3.78 (d, J = 0.7 Hz, 3H), 1.96 – 1.67 (m, 8H), 1.66 – 1.48 (m, 4H), 1.31 (d, J = 0.8 Hz, 3H). ^{13}C NMR (100 MHz, Chloroform-*d*) δ 159.2, 141.3, 141.3, 139.2, 132.0, 131.9, 128.7, 128.6, 128.1, 128.0, 125.4, 125.3, 114.0, 105.4, 105.3, 92.2, 92.1, 81.9, 81.9, 66.5, 66.4, 55.3, 39.6, 39.0, 38.9, 38.5, 38.4, 36.7, 28. 4, 28.1, 23.9. HRMS (ESI) calculated for [$C_{22}H_{28}N_2NaO_2$] $[M+Na]^+$: 375.2043 found: 375.2038.

4.6.3 (*S, E*)-4-(3-(2-methyl-1-oxaspiro[4.4]nonan-2-yl)prop-1-en-1-yl)phenol (13) [8-9]

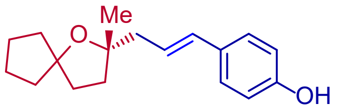

13

$C_{12}H_{25}SH$ (204 mg, 1 mmol) was added to a stirred suspension of sodium hydride (60% dispersion in mineral oil) (44 mg, 1.1 mmol) in dry diethyl ether (2 mL) over 10 min at a temperature around 5 °C. The solvent was removed in a vacuum, and the obtained residue was added NMP (1 mL). Then **(*R*)-5c** (143 mg, 0.5 mmol) was added with vigorous stirring at room temperature. The mixture was allowed to heat to 130 °C and kept at that temperature for 18 h. The solvent was neutralized to pH < 7 by using a diluted hydrochloric acid solution and extracted with ethyl acetate (3 × 15 mL). The combined organic layer was washed with brine, dried over anhydrous Na_2SO_4 and filtered. The filtrate was concentrated, after flash column chromatography on a silica gel (petroleum ether: ethyl acetate = 5: 1) give the product **13** as a yellow solid (108.4 mg, 80% yield, 90% ee). m.p. 137-147 °C. $[\alpha]_D^{20}$= 0.57 (*c* = 0.4, Chloroform). ^{1}H NMR (400 MHz, Chloroform-*d*) δ 7.25 (s, 1H), 7.13 (d, J = 8.6 Hz, 2H), 6.78 (t, J = 5.6 Hz, 2H), 6.30 (d, J = 15.8 Hz, 1H), 6.11 – 5.97 (m, 1H), 2.44 – 2.31 (m, 2H), 1.97 – 1.81 (m, 5H), 1.79 – 1.67 (m, 3H), 1.58 (dddd, J = 14.4, 10.4, 9.8, 5.4 Hz, 4H), 1.27 (s, 3H). ^{13}C NMR (100 MHz, Chloroform-*d*) δ 155.43, 132.12, 129.92, 127.23, 123.88, 115.52, 92.21, 83.58, 45.67, 39.54, 39.36, 37.16, 36.79, 27.48, 23.69.RMS (ESI) calculated for [$C_{18}H_{24}NaO_2$] $[M+Na]^+$: 295.1662 found: 295.1669. $[\alpha]_D^{20}$ = 0.57 (*c* = 0.4, Chloroform). Enantiomeric excess was determined by HPLC with a Chiralpak ADH column (hexanes: 2-propanol = 99: 10, 0.5 mL/min, 254 nm); major enantiomer tr = 12.151 min, minor enantiomer tr = 14.004 min.

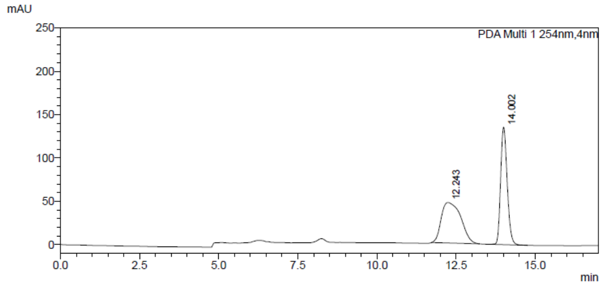

<Peak Table>

PDA Ch1 254nm

Peak#	Ret. Time	Height	Height%	Area	Area%
1	12.243	46705	25.629	2009709	50.680
2	14.002	135528	74.371	1955797	49.320
Total		182233	100.000	3965507	100.000

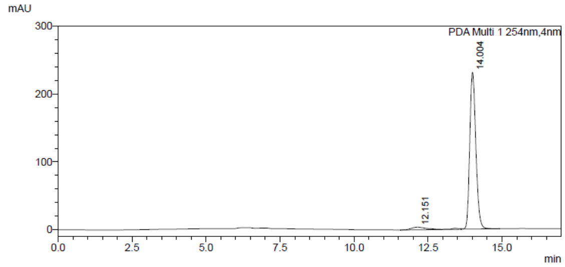

<Peak Table>

PDA Ch1 254nm

Peak#	Ret. Time	Height	Height%	Area	Area%
1	12.151	3746	1.595	169043	4.853
2	14.004	231146	98.405	3314183	95.147
Total		234893	100.000	3483227	100.000

5. X-ray structure of Xu6 and 13.

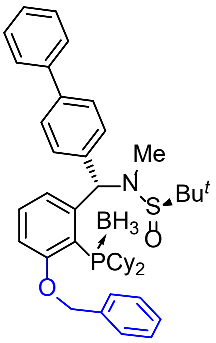

Xu**6**

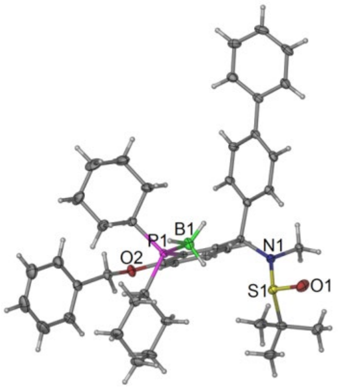

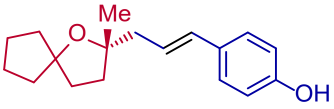

13

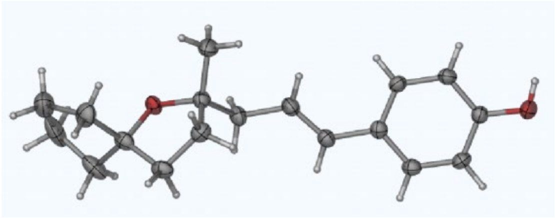

6. Procedure of vibrational circular dichroism (VCD) experiment

VCD and IR measurements of **8-D** were performed on a BioTools ChiralIR-2X FT-VCD spectrometer, equipped with a single photoelastic modulation and a mercury cadmium tellurium detector. 15 mg of sample was dissolved in 300μL $CDCl_3$ and placed in a BaF_2 cell with a pathlength of 75μm. Data were acquired at a resolution of 4 cm-1 for 15 h.

7. Computational details

General calculation procedure

Density functional theory (DFT) calculations were carried out using Gaussian 09 E.01 software package.[10] All structure optimizations and frequency calculations were performed using the PBE0 functional[11] with D3BJ dispersion correction (PBE0-D3BJ) and combined basis set.[12-13] That is SDD basis set[14] for Palladium and 6-31G* basis set for all other elements.[15-16] Intrinsic reaction coordinate calculations were performed to ensure every TS is connected with corresponding equilibrium structure[17-18]. Single point energies were further calculated using PBE0-D3BJ functional and def2-TZVP basis set[19] for higher accuracy. Truhlar and coworkers' SMD solvation model[20] was employed to consider the solvent effect of the mixed solvent of Et_2O and Hexane. The solvent is defined by 7 parameters. As it is not on the solvent list of Gaussian 09 software, the parameters used in calculation are the average value of Et_2O and Hexane: eps (3.06095), epsinf (1.36375), HBondAcidity(0.00), HBondBasicity(0.205), SurfaceTensionAtInterface(24.855), CarbonAromaticity(0.000), ElectronegativeHalogenicity(0.000). 3D structures and isosurfaces are visualized by VMD 1.9.3[21] and CYLview v0.1b[22].

VCD calculation procedure

VCD spectra of different conformers may differ considerably. In order to compare with the experimental spectrum, conformation search and Boltzmann weighted spectrums are essential. First, systematic conformation search of both configuration (*S* and *R*) of **8-D** (**8-D-*S*** and **8-D-*R***) was performed with **CREST** program developed by Prof. Grimme.[23] (detailed input: "crest xtbopt.xyz --T 4 --v3 --ewin 6 --rthr 0.35 --ethr 0.2 --chrg 0 --noreftopo --temp 298.15 --gfn 2 --shake 1 --hmass 1") Then, **isostat** component of **Molclus** program developed by Tian Lu et al.[24] is used to remove the duplicated conformers (detailed input: "isostat crest_conformers.xyz -nt 4 -Nout 60 -Eout 5 -Edis 0.3 -Gdis 0.3 -T 298.15"). 23 and 28 low-energy conformations of **8-D-*S*** and **8-D-*R*** were achieved, respectively.

Then, optimization and free energy calculations of the structures were carried out using the Gaussian 09 E.01 software package.[10] All structure optimizations, frequency and VCD spectrum calculations (invoked by "freq=vcd" keyword) were performed using B3LYP functional[25-27] with D3BJ dispersion correction developed by Grimme et al. And 6-31G* basis set was chosen to match the frequency correction factor (0.9614) provided by Multiwfn.[28] The solvent was changed to chloroform (specified by "scrf=(smd,solvent=chloroform)" in the command line of Gaussian input file) to match the solvent ($CDCl_3$) used in the VCD experiment. Single point energies were further calculated at B3LYP-D3BJ/def2-TZVP level for higher accuracy.

After obtaining the free energy of all conformations, their VCD spectrum were calculated according to Boltzmann distribution with Multiwfn.[28] FWHM was set as 8.0 cm^{-1}, while frequency correction factor is 0.9614.

Experimental and simulated (with the same procedure stated above) IR spectrum of **8-D-*SR*** and **8-D-*SS*** are provided is **Supplementary Figure1**.

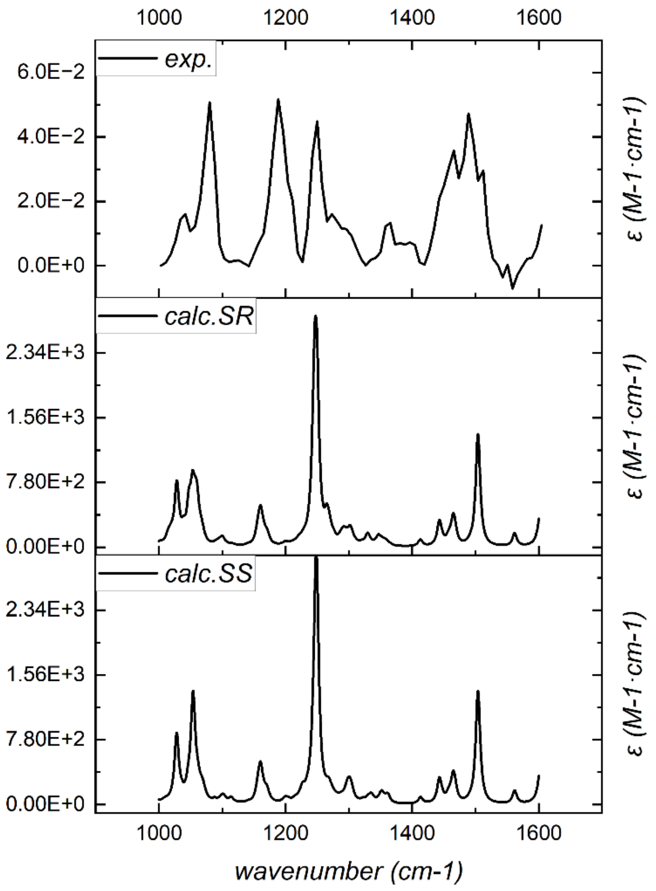

Supplementary Figure1. Experimental and simulated IR spectrum of 8-D-*SR and 8-D-SS*

Considering that free energy profile of conformations may be sensitive to the computational parameters, which will influence the Boltzmann distribution result and consequently result in differences in simulated spectrums, we slightly altered the calculation parameters and reperformed the calculation. The Boltzmann distribution profile are provided in **Supplementary Table1** and spectrums are provided in **Supplementary Figure2**. We observed that while there are variations in the Boltzmann distribution of different conformers and the spectra, the overall trend remains consistent across various calculation levels. Therefore, we believe that this result lends support to our initial conclusion.

Supplementary Table1. Boltzmann distribution profile of 8-D-*S* conformers under different computational parameters

structure index	standard*	no-disp-scrf**	no-disp-scrf-dp***
0	4.80%	2.40%	2.51%
1	4.62%	4.93%	4.89%
2	6.96%	6.78%	6.90%

3	14.23%	14.60%	10.50%
4	11.03%	4.25%	4.69%
5	10.99%	4.25%	4.69%
6	4.74%	1.90%	1.96%
7	5.39%	6.17%	6.26%
8	0.77%	0.35%	0.26%
9	2.83%	3.99%	4.04%
10	0.44%	0.22%	0.20%
11	7.11%	12.98%	13.46%
12	8.69%	0.38%	0.43%
13	3.83%	7.70%	8.19%
14	3.63%	4.01%	4.23%
15	1.42%	3.90%	4.18%
16	1.55%	4.65%	4.73%
17	3.58%	10.66%	11.87%
18	0.52%	0.18%	0.18%
19	1.55%	4.69%	4.74%
20	0.71%	0.33%	0.33%
21	0.31%	0.34%	0.38%
22	0.31%	0.34%	0.38%

* standard parameter

** standard parameter without dispersion correction and solvent model

*** standard parameter without dispersion correction and solvent model and change basis from 6-31G* to 6-31G** in optimization process

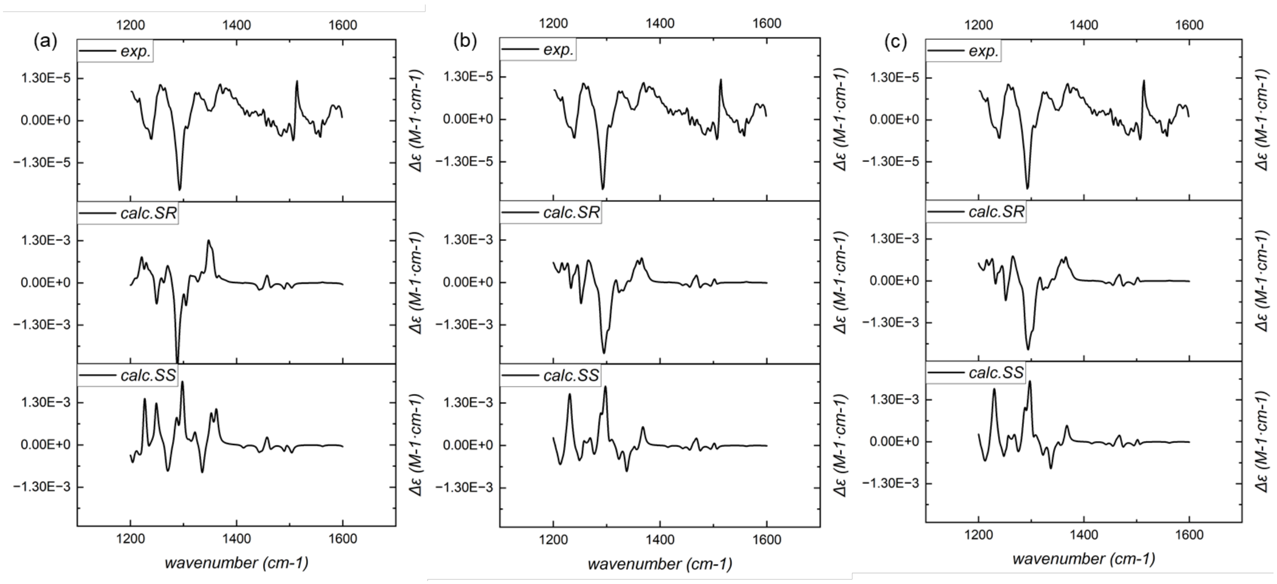

Supplementary Figure2. VCD spectrum of 8-D-*SR* and 8-D-*SS* under different computational parameters. (a) standard parameter; (b) standard parameter without dispersion correction and solvent model; (c) standard parameter without dispersion correction and solvent model and change basis from 6-31G* to 6-31G** in optimization process.

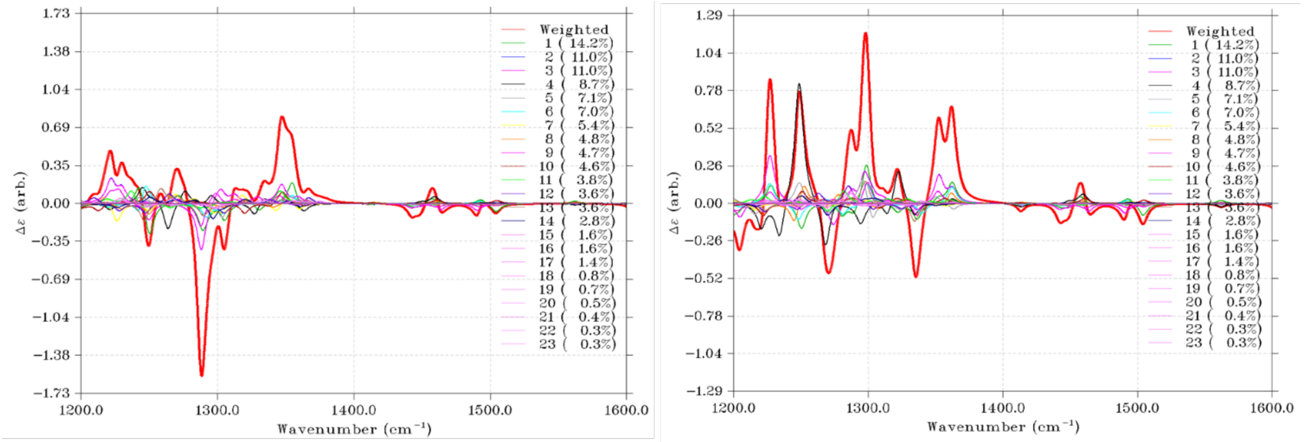

Supplementary Figure3. Overlayed and weighted 8-D-*SR* (left) and 8-D-*SS* (right) VCD spectra of all conformers under standard calculation parameters

In order to be certain that we have considered enough conformers, clustering threshold was tightened in **isostat** (modified input: “isostat crest_conformers.xyz -nt 4 -Nout 60 -Eout 5 -Edis 0.2 -Gdis 0.2 -T 298.15”), and 38 and 42 low-energy conformations of 8-D-S and 8-D-R were achieved. VCD calculation procedure were applied to these conformers and the comparison between the 8-D-SS and 8-D-SR VCD spectra of different number of conformers are provided in **Supplementary Figure4**. From which we may conclude that the conformers we have taken into consideration is basically sufficient and the spectra is reliable.

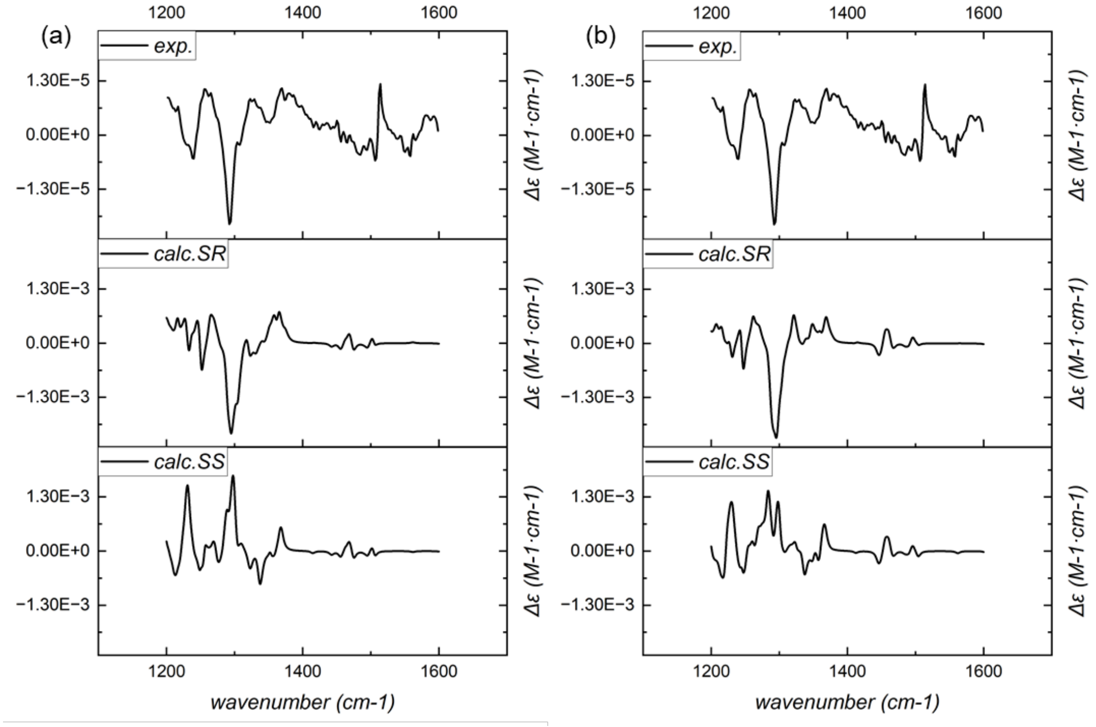

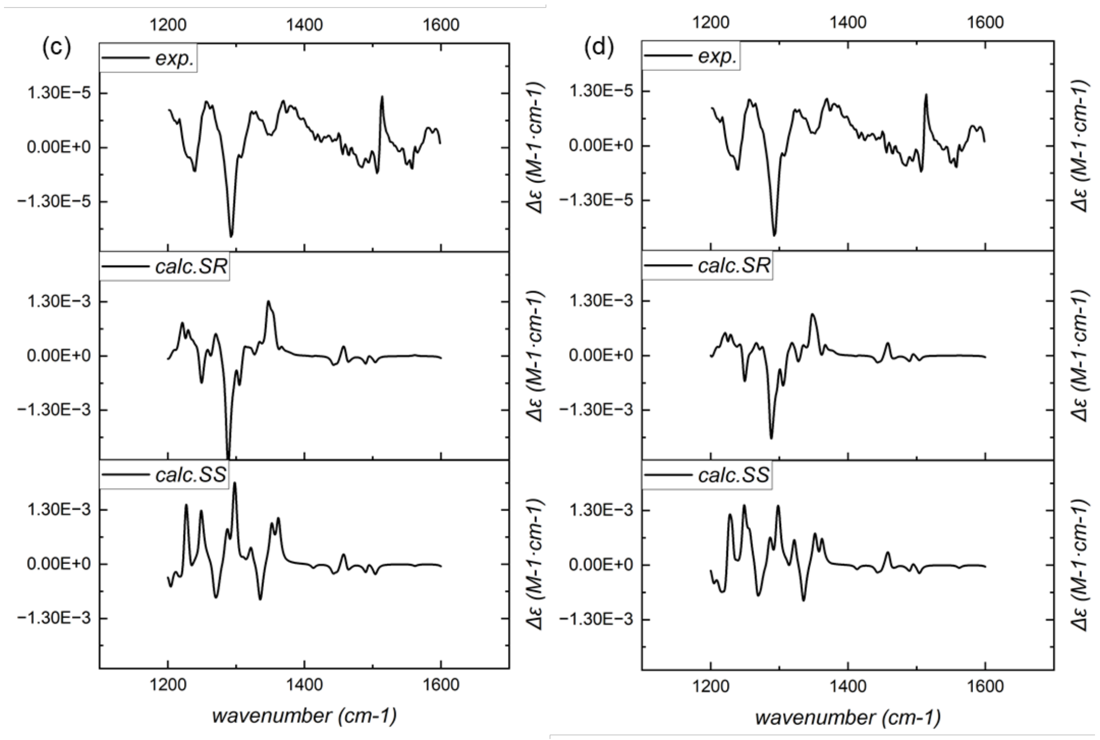

Supplementary Figure4. Comparison between VCD spectrum calculated with different number of conformers. (a) standard parameter without dispersion correction and solvent model, 23 conformers; (b) standard parameter without dispersion correction and solvent model, 38 conformers; (c) standard parameter, 23 conformers; (d) standard parameter, 38 conformers.

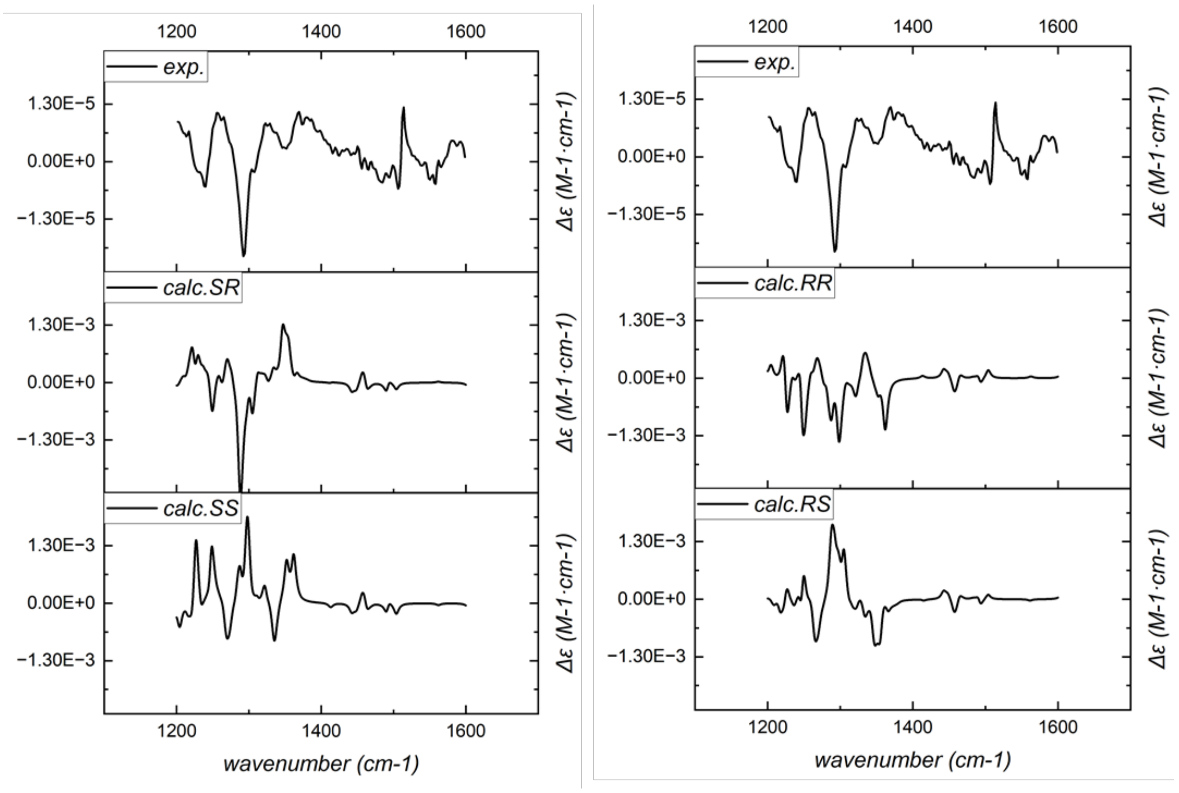

Supplementary Figure5. Comparison between 8-D-S (left) and 8-D-R (right) VCD spectrum.

Transition state calculations

Since the conformations of the TSs are quite complicated, in order to prove that the TS core structures found in **Xu8** are representative in this reaction other than a result by chance, **Xu1, Xu2, Xu3, Xu6, Xu8, Xu9** were taken as templates to calculate the free energy difference between major and minor TSs based on the core TS conformation found in **Xu8**. The calculated *ee*% are in good accordance with the experimental results (**Supplementary Table2**). TS structures are provided in related .xlsx file along with the SI, named as **Xu1-1a-2a-major** and **Xu1-1a-2a-minor** etc.

Supplementary Table2. Calculated and experimental *ee*%

Entry	Ligand and reactants	Calc. ΔΔG (kcal/mol)	Calc. *ee*%	Exp. *ee*%
1	**Xu1** + **1a** + **2a**	-0.02	-2	2
2	**Xu2** + **1a** + **2a**	0.87	63	85
3	**Xu3** + **1a** + **2a**	1.42	83	86
4	**Xu6** + **1a** + **2a**	0.98	68	82
5	**Xu8** + **1a** + **2a**	2.23	95	92
6	**Xu9** + **1a** + **2a**	1.81	91	92

Energy difference decomposition calculations

In order to analysis the key stereo-controlling factors and investigate the CH-π interaction quantitively, energy difference decomposition calculations were carried out. Firstly, from **Supplementary Table3**, we can tell that metal-involved interactions are quite similar between TSs of **Xu6** and **Xu8**. In other words, these interactions do not contribute much to the ΔΔE between them. Therefore, Pd atoms are removed from the TS structures in the later analysis, to eliminate the interaction difference involving metal centers. And weak interactions between **Xu-Phos** and substrates are focused.

Supplementary Table3. Calculated ΔΔG and ΔE of structure without Pd (kcal/mol)

Entry	Ligand and reactants	Calc. ΔΔG	no Pd ΔE	Metal-involved interaction
1	**Xu6** + **1a** + **2a**	0.98	7.86	6.88
2	**Xu8** + **1a** + **2a**	2.23	9.35	7.12

Secondly, to investigate interaction between **Xu-Phos** and substrates, no Pd TS structures were split into two dimers: (a) **Xu-Phos**-alcohol dimer (referenced as dimer1 later) and (b) **Xu-Phos**-vinyl substrate dimer (referenced as dimer2 later). ΔE_{dimer1} and ΔE_{dimer2} are provided in **Supplementary Table4**.

Supplementary Table4. Calculated ΔE_{dimer1} and ΔE_{dimer2} (kcal/mol)

Entry	Ligand and reactants	ΔE_{dimer1}	ΔE_{dimer2}
1	**Xu6** + **1a** + **2a**	2.53	3.80
2	**Xu8** + **1a** + **2a**	1.84	8.20

From **Supplementary Table4**, it is clear that the energy difference of dimer2 is the main contributor of the total energy gap. So, we further separate the dimer into (a) **Xu-Phos** and (b) vinyl substrate. More analysis results (**Fig 7b**) are provided in the manuscript.

Analysis of different conformation of Xu6 and Xu8 TSs

We proposed that the side-arm conformation of **Xu6** and **Xu8** are critical to the enantioselectivity. To certify that current conformations are not a result by chance, we replaced H adjacent to the side-arm in **Xu6** TSs to OMe to generate new conformation of **Xu8** TSs and also generated new conformation of **Xu6** TSs with similar procedure. The energy difference profile of the TSs is provided in **Supplementary Table5**, the structures are shown in **Supplementary Figure6**, and coordinates are provided in related .xlsx file along with the SI.

Supplementary Table5. Energy difference between conformations of Xu6 and Xu8 TSs (kcal/mol)

Entry	TS name	$\Delta E_{Xu\text{-}phos}$	ΔE_{other}	$\Delta E_{int\text{-}sidearm}$	$\Delta E_{int\text{-}all}$	ΔE_{all}	ΔG_{all}
1	**Xu6-major**	2.51	1.55	-0.04	-3.08	0.98	0.66
2	**Xu6-minor**	-1.50	0.06	3.67	3.83	2.39	4.07
3	**Xu8-major**	0.36	-0.08	0.02	-0.39	-0.10	1.42
4	**Xu8-minor**	1.90	-0.28	-3.03	-0.50	1.12	2.08

1) All energy differences are calculated as the new TSs' energy subtract the original TSs' energy

2) $E_{Xu\text{-}phos}$ refers to the single point energy of **Xu-phos** segment in the TS structures

3) E_{other} refers to the sum of single point energy of all segments except for **Xu-phos** (i.e. vinyl substrate and alcohol substrate) in the TS structures

4) $E_{int\text{-}sidearm}$ refers to the interaction energy between side-arm and vinyl substrate in the TS structures

5) $E_{int\text{-}all}$ refers to the sum of interaction energy between all segments in the TS structures

6) E_{all} refers to the single point energy of the TS structures

7) G_{all} refers to the free energy of the TS structures

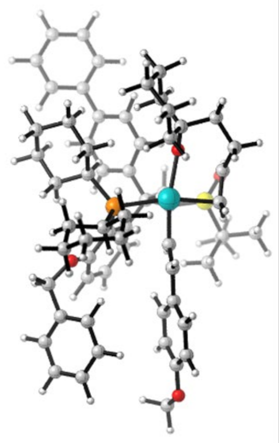
Xu6-major

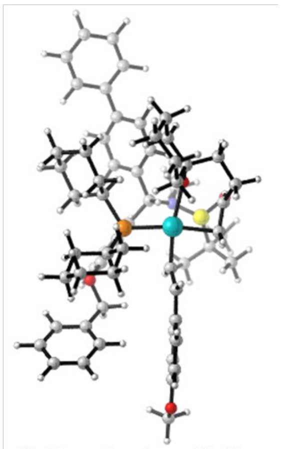
Xu6-major-fromXu8

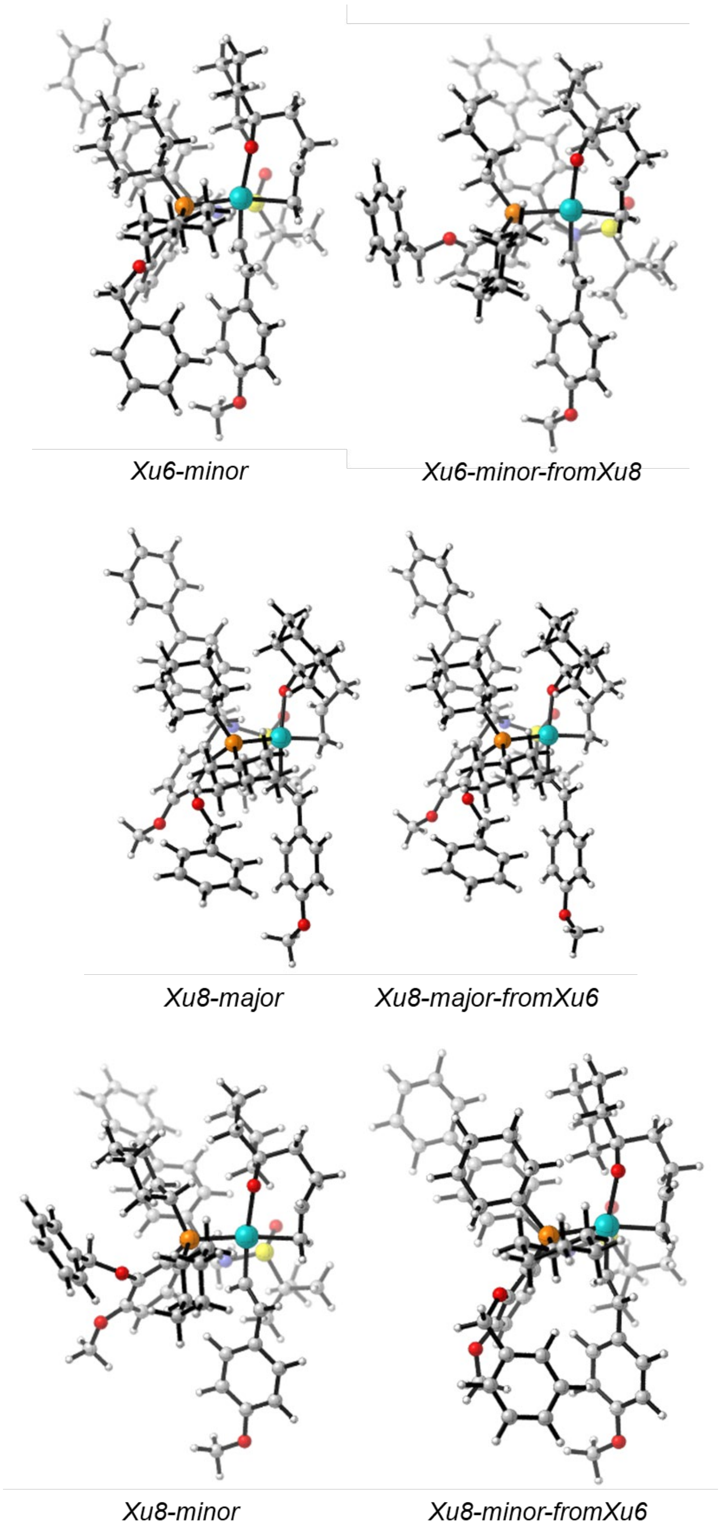

Supplementary Figure6. Structures of different structures of Xu6 and Xu8 TSs.

Referring to **Supplementary Table5**, we can make the following observations: 1) None of the new TS structures exhibit lower free energy compared to their corresponding original TS structures. This reinforces our analysis, which is primarily based on the original TS structures; 2) Focusing on the **Xu6**-major TSs, a noteworthy increase in energy, denoted by $\Delta E_{Xu\text{-}phos}$, suggests that the introduction of the OMe group does indeed compel the side-arm to adopt a different position (even though both positions are downward), resulting in an overall higher energy for the entire ligand (+2.51 kcal/mol); 3) Concerning the **Xu6**-minor TSs, it is notable that the primary contributor to

the increase in energy (+3.67 kcal/mol) is the loss of interaction between the side-arm and the vinyl substrate. This underscores the significance of this interaction in the overall energetics of the system; 4) In the case of **Xu8**-major TSs, after the H was substituted with OMe, side-arm was optimized spontaneously to a conformation similar to the original **Xu8**-major TS. As a result, the single-point energies of these structures turned out to be very similar.; 5) Concerning **Xu8**-minor TSs, forcing the side-arm to take the downward conformation indeed make the ligand energy higher (+1.90 kcal/mol), which is the main contributor of the overall energy rise, and although interaction between the side-arm and vinyl substrate is stronger, it is masked by the loss of other interactions.

Analysis of the effect of OR groups ortho to the side-arm

We originally proposed that the main reason of OMe promoting the side-arm effect is its steric influence. To verify this proposal, OMe group was replaced with OEt, OiPr and OtBu groups respectively to generate new ligands **Xu12**, **Xu13** and **Xu14**. Difference of free energies and *ee*% values were calculated according to the standard procedure and provided in **Supplementary Table6**.

Supplementary Table6. Calculated and experimental *ee*% of ligands with different OR groups

Entry	Ligand and reactants	Calc. ΔΔG (kcal/mol)	Calc. *ee*%	Exp. *ee%*
1	**Xu12** + **1a** + **2a**	2.16	94	90
2	**Xu13** + **1a** + **2a**	0.99	68	85
3	**Xu14** + **1a** + **2a**	1.08	72	/*

* Multiple attempts to synthesize **Xu14** all failed, so experimental *ee%* value of **Xu14** is not available.

The high calc. and exp. *ee%* value of **Xu12** supports that the steric effect of OR groups are beneficial to improvement of *ee%*. However, we also found that when the OR group gets bulkier, the *ee%* value starts to drop. From visual inspection of the TSs of **Xu13** and **Xu14**, we suspect that as the OR groups might have weak interaction with vinyl substrate (**Supplementary Figure7**). To verify this, interaction energy between OR groups and vinyl substrates are calculated and provided in **Supplementary Table7**.

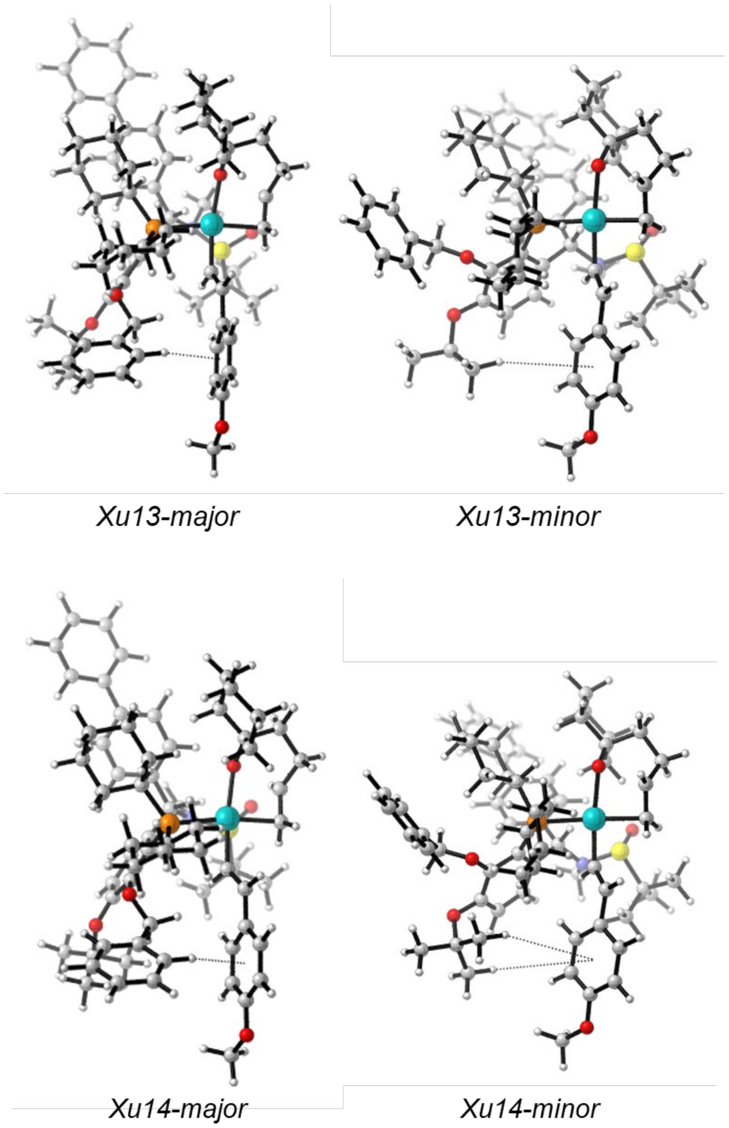

Supplementary Figure7. Structure of Xu13 and Xu14 TSs

Supplementary Table7. Weak interaction energy between different OR groups and vinyl substrate (kcal/mol)

Entry	Ligand and reactants	Eint	ΔEint	Calc. ΔΔG
1	**Xu8-major**	-0.01	/	/
2	**Xu8-minor**	-0.13	-0.12	2.23
3	**Xu12-major**	-0.03	/	/
4	**Xu12-minor**	-0.17	-0.14	2.16
5	**Xu13-major**	-0.07	/	/
6	**Xu13-minor**	-0.77	-0.70	0.99
7	**Xu14-major**	-0.56	/	/
8	**Xu14-minor**	-1.48	-0.91	1.08

From **Supplementary Table7**, it is clear that when the OR group is OMe or OEt, the interaction between OR group and vinyl substrate is quite weak and negligible, but when it comes to OiPr and OtBu, the interaction become significant. More importantly, the interactions in the minor TSs are

stronger than those in the major TSs due to the conformation difference. And ΔEint accounts for the major part of the decrease of the ΔΔG and thus the decrease of *ee%*.

In conclusion, we posit that the enhancement in ee% achieved by introducing the OMe group into the ortho position of the side-arm is primarily attributed to its steric effect. Nonetheless, the introduction of bulkier substitution groups is likely to lead to weak interactions with the vinyl substrate, particularly in the minor transition state, consequently resulting in a decrease in ee%.

Visualization of weak interaction

We calculated weak interactions between two fragments: 1. Side-arm (OBn group, atom index 77-91 in **Xu8-ts**) 2. Vinyl substrate (atom index 108-126 in **Xu8-ts**). Weak interaction is calculated with IGM method by Multiwfn.[28] Isovalue of the isosurface shown in **Fig 7** is 0.01.

Quantitative calculation of weak interaction

The interaction energy values are defined as the single point energy gap between the complex and components of the complex (**Eq 1**).[29]

$$E_{int} = E_{complex} - E_{component1} - E_{component2} \quad \textbf{(Eq 1)}$$

To illustrate the calculation procedure, we will use the example of calculating the interaction energy between the side-arm and the vinyl substrate in Xu8-ts. Firstly, we isolate the side-arm (OBn group, atom index 77-91) and the vinyl substrate (atom index 108-126) from the TS structure. Next, we introduce a hydrogen atom at each break point of the covalent bonds within these isolated molecules. The positions of these added hydrogen atoms are then optimized using standard optimization parameters (as outlined in the General Calculation Procedure), with all other atoms held strictly constrained. Finally, we perform a single-point energy calculation using standard parameters (as specified in the **General Calculation Procedure**). This calculation yields the energy of the complex as well as the individual energies of its constituent parts.

9. ^{1}H, ^{13}C, ^{31}P, ^{19}F NMR spectra

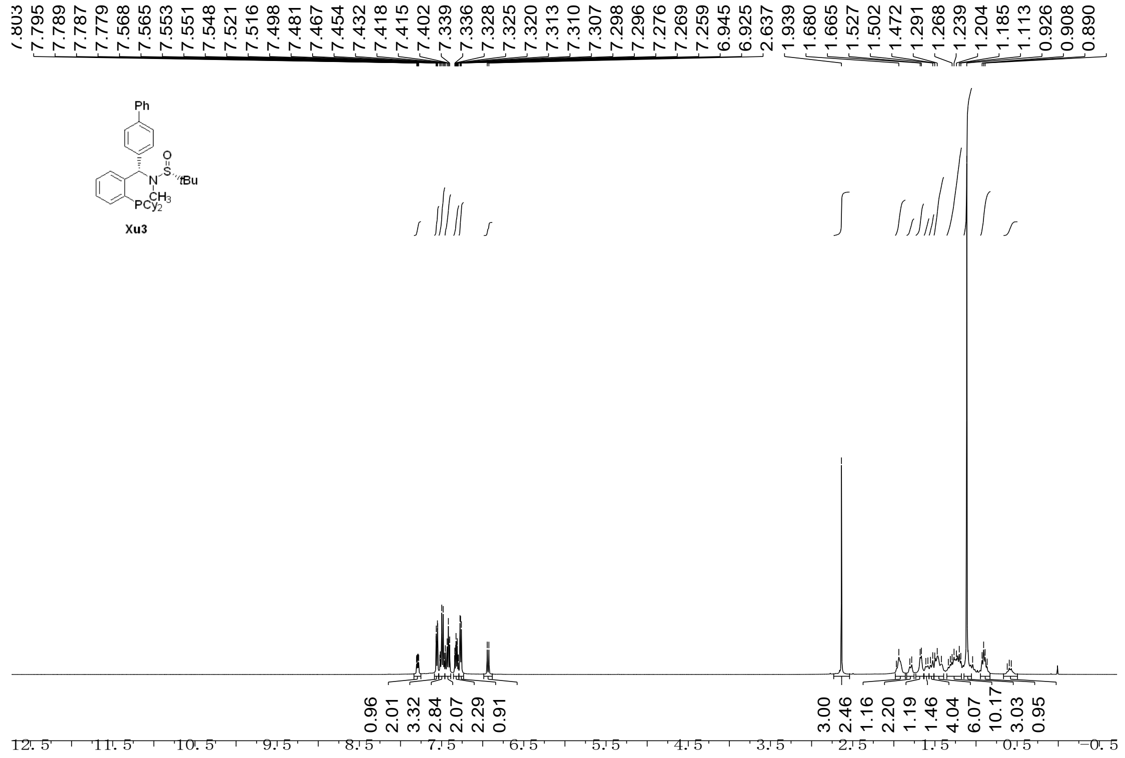

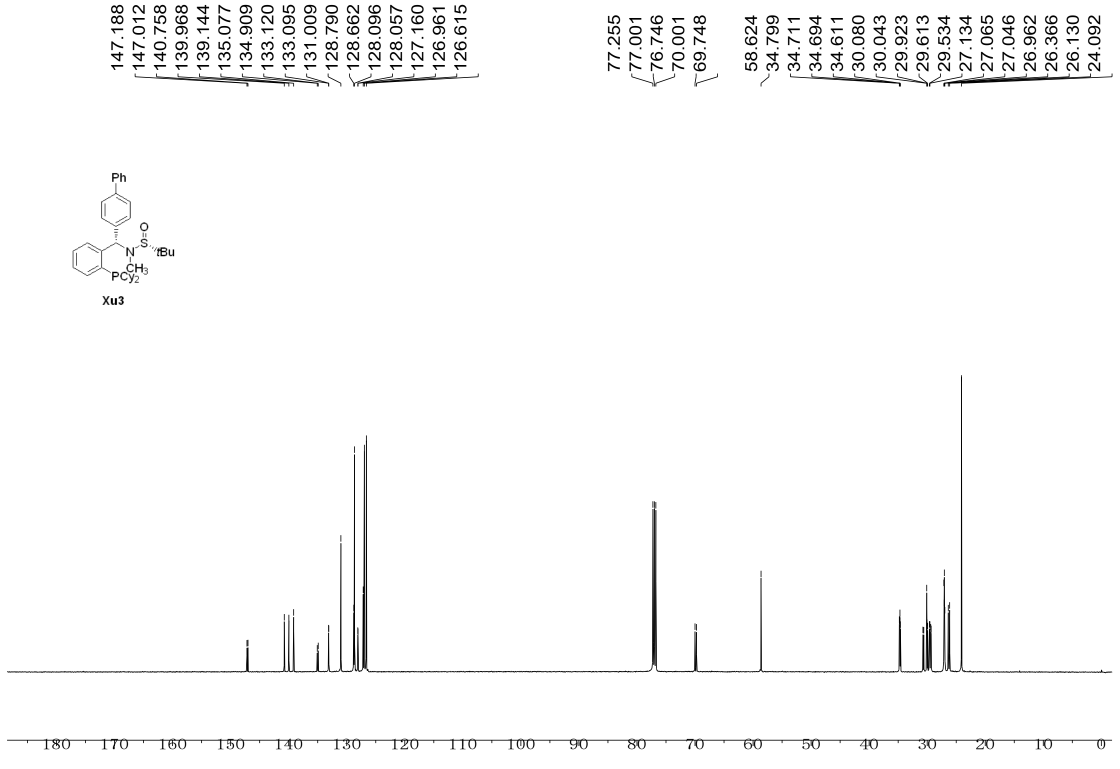

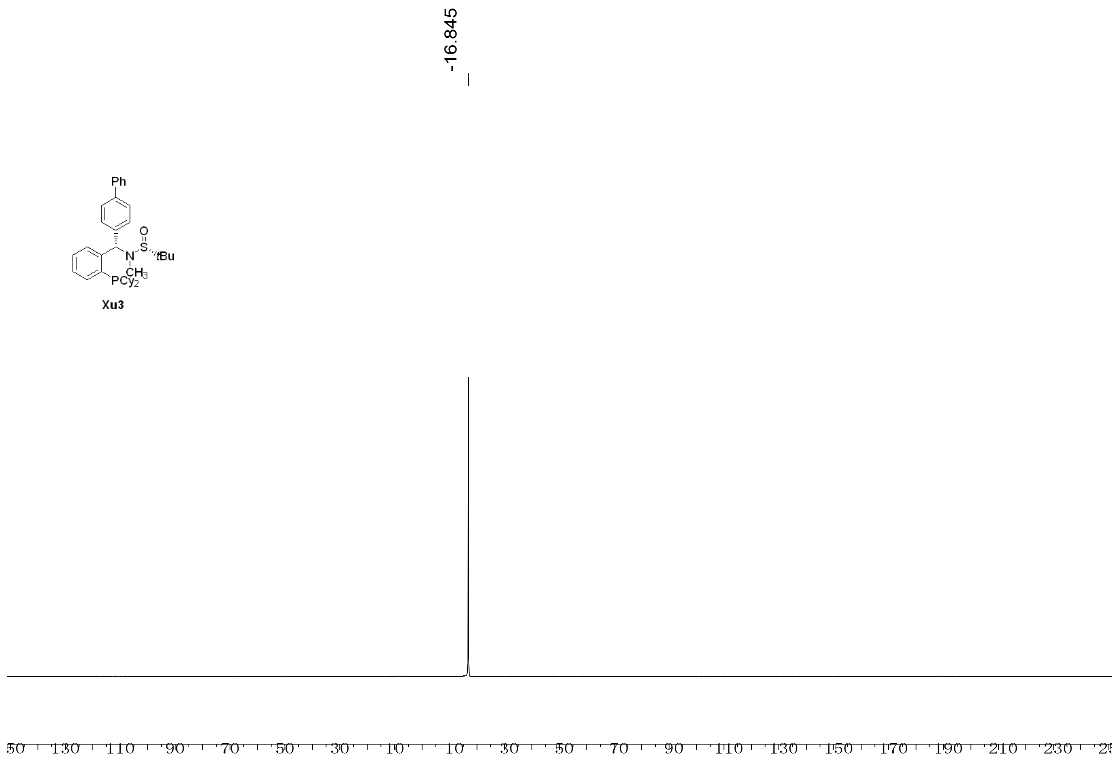
-16.845
Ph
O
S
tBu
N
CH3
PCy2
Xu3
50 130 110 90 70 50 30 10 -10 -30 -50 -70 -90 -110 -130 -150 -170 -190 -210 -230

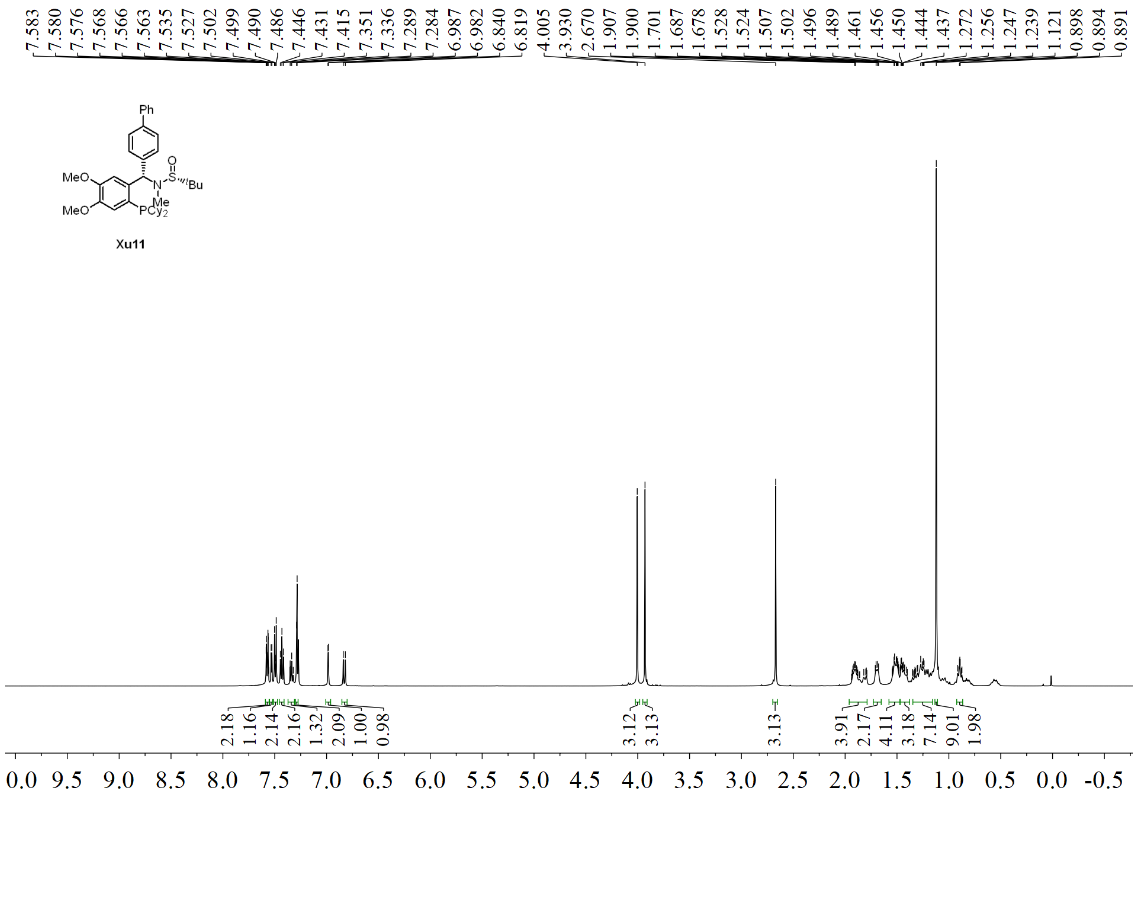
7.583
7.580
7.576
7.568
7.566
7.563
7.535
7.527
7.502
7.499
7.490
7.486
7.446
7.431
7.415
7.351
7.336
7.289
7.284
6.987
6.982
6.840
6.819
4.005
3.930
2.670
1.907
1.900
1.701
1.687
1.678
1.528
1.524
1.507
1.502
1.496
1.489
1.461
1.456
1.450
1.444
1.437
1.272
1.256
1.247
1.239
1.121
0.898
0.894
0.891
Ph
MeO
MeO
O
S
tBu
N
Me
PCy2
Xu11
2.18
1.16
2.14
2.16
1.32
2.09
1.00
0.98
3.12
3.13
3.13
3.91
2.17
4.11
3.18
7.14
9.01
1.98
0.0 9.5 9.0 8.5 8.0 7.5 7.0 6.5 6.0 5.5 5.0 4.5 4.0 3.5 3.0 2.5 2.0 1.5 1.0 0.5 0.0 -0.5

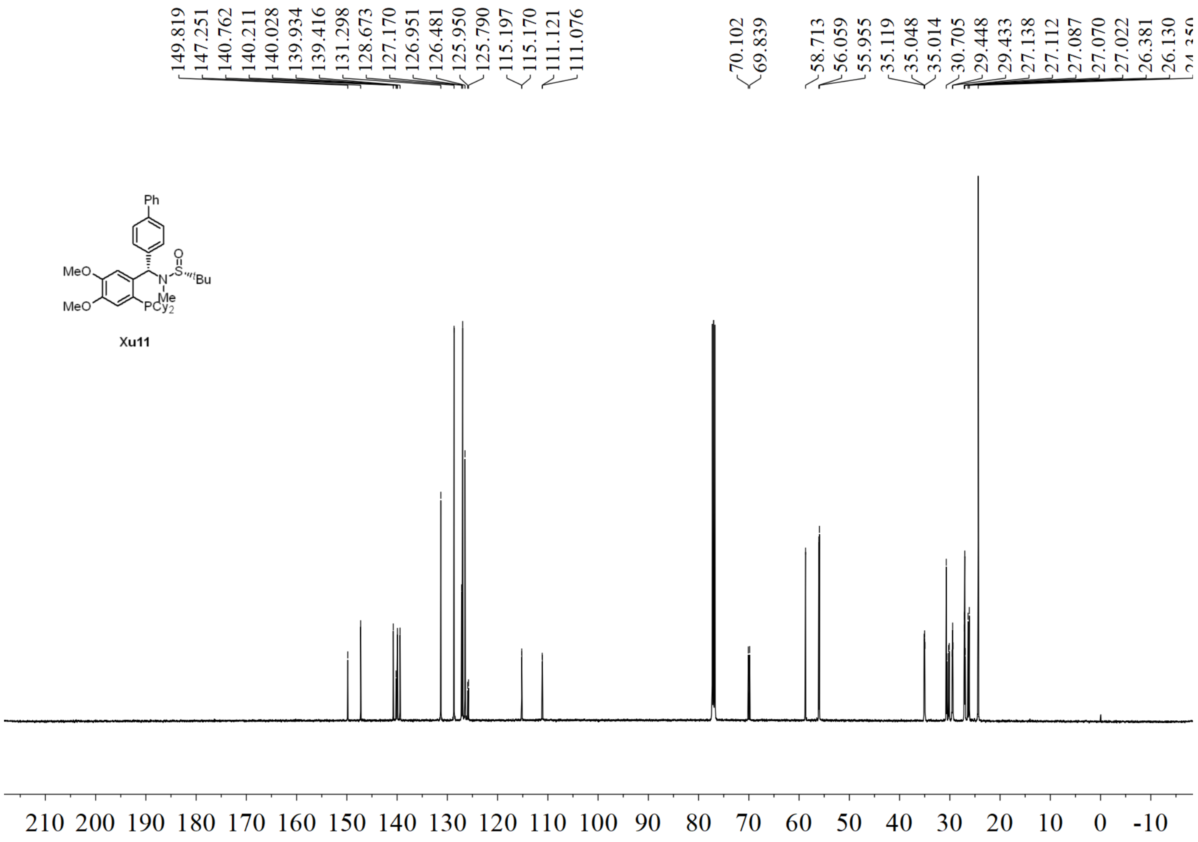

149.819
147.251
140.762
140.211
140.028
139.934
139.416
131.298
128.673
127.170
126.951
126.481
125.950
125.790
115.197
115.170
111.121
111.076
70.102
69.839
58.713
56.059
55.955
35.119
35.048
35.014
30.705
29.448
29.433
27.138
27.112
27.087
27.070
27.022
26.381
26.130
Ph
MeO
MeO
N
S
O
tBu
Me
PCy2
Xu11
210 200 190 180 170 160 150 140 130 120 110 100 90 80 70 60 50 40 30 20 10 0 -10

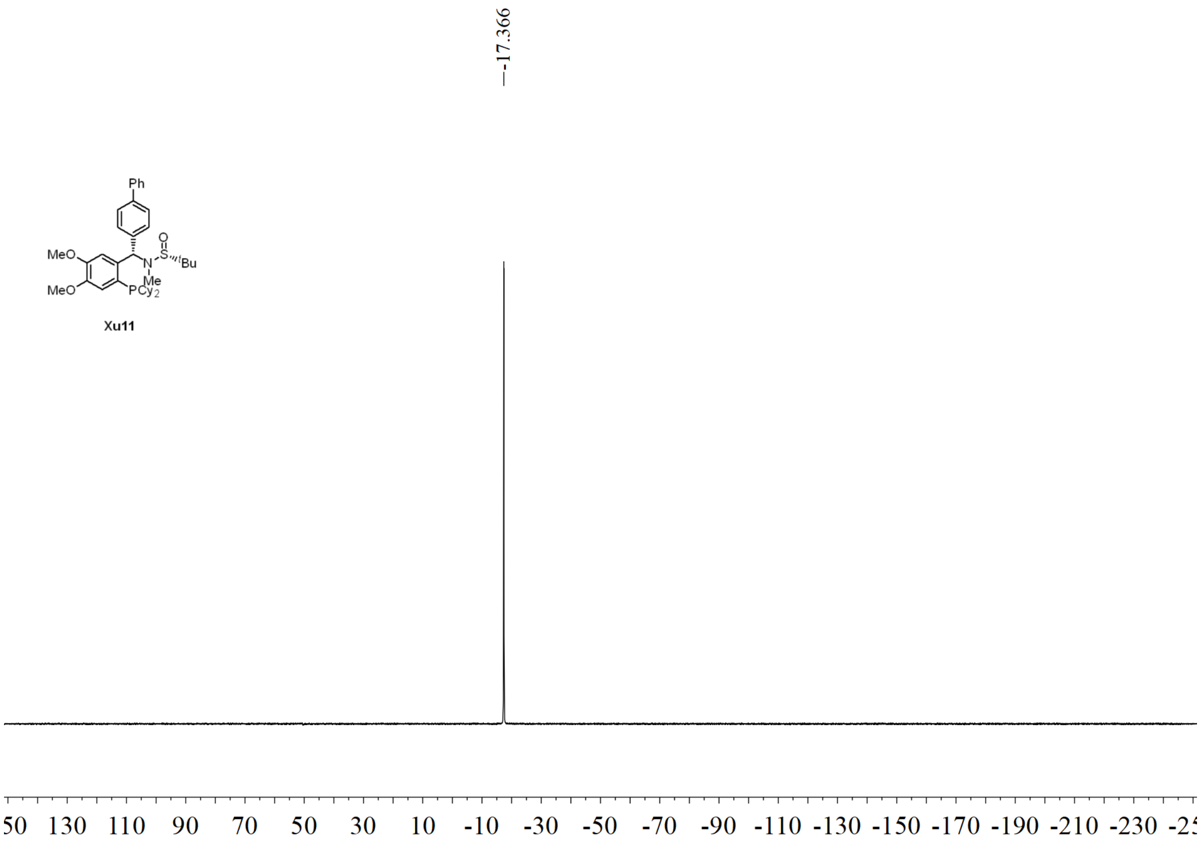

-17.366
Ph
MeO
MeO
N
S
O
tBu
Me
PCy2
Xu11
130 110 90 70 50 30 10 -10 -30 -50 -70 -90 -110 -130 -150 -170 -190 -210 -230

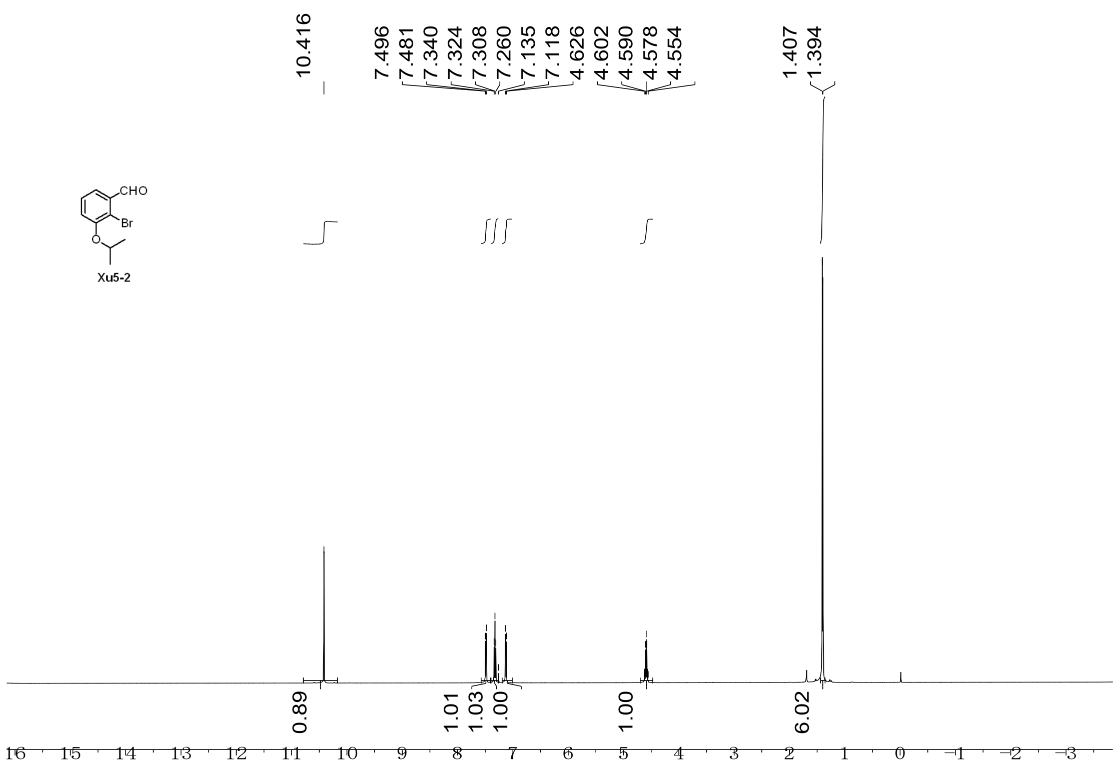

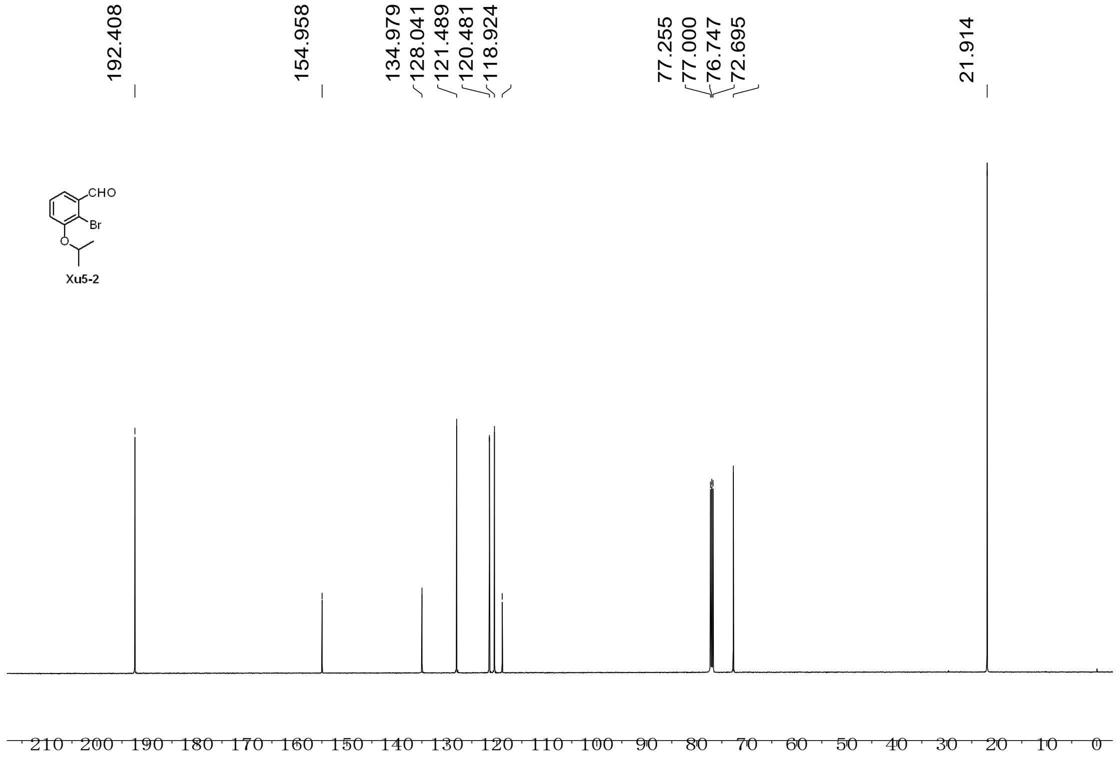

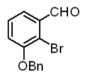
CHO
Br
OBn

Xu6-2

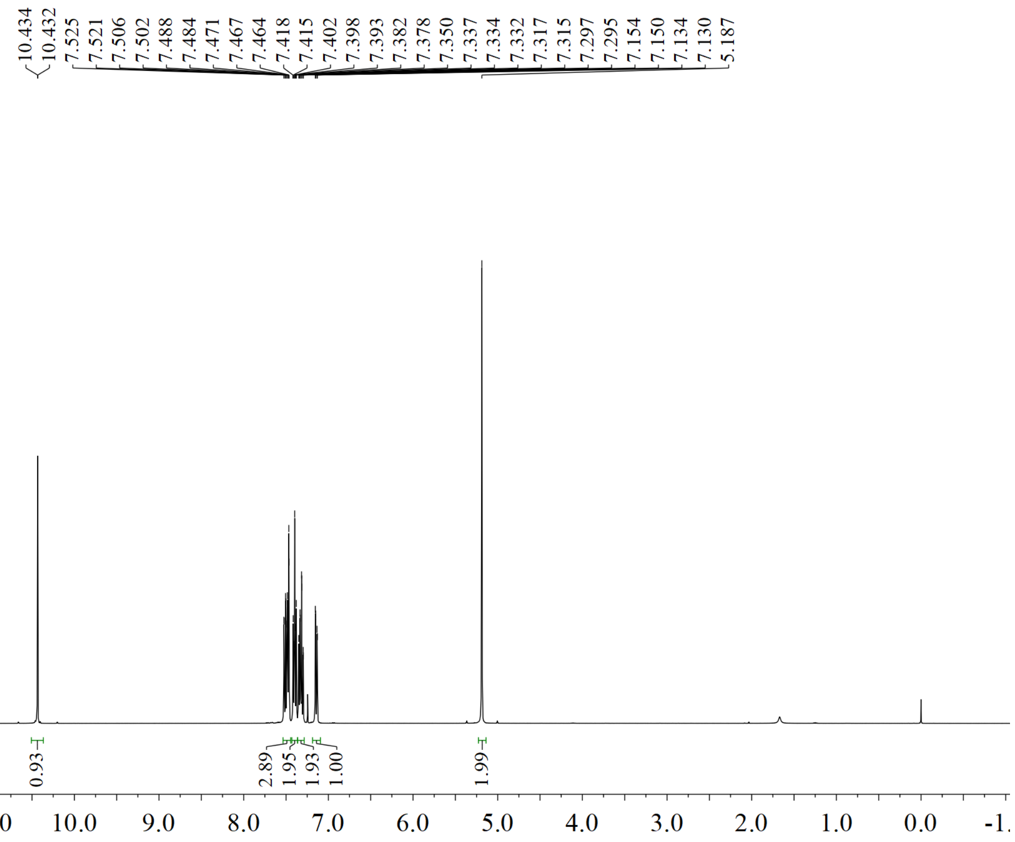
10.434
10.432
7.525
7.521
7.506
7.502
7.488
7.484
7.471
7.467
7.464
7.418
7.415
7.402
7.398
7.393
7.382
7.378
7.350
7.337
7.334
7.332
7.317
7.315
7.297
7.295
7.154
7.150
7.134
7.130
5.187
0.93
2.89
1.95
1.93
1.00
1.99
12.0 11.0 10.0 9.0 8.0 7.0 6.0 5.0 4.0 3.0 2.0 1.0 0.0 -1.

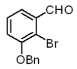
CHO
Br
OBn

Xu6-2

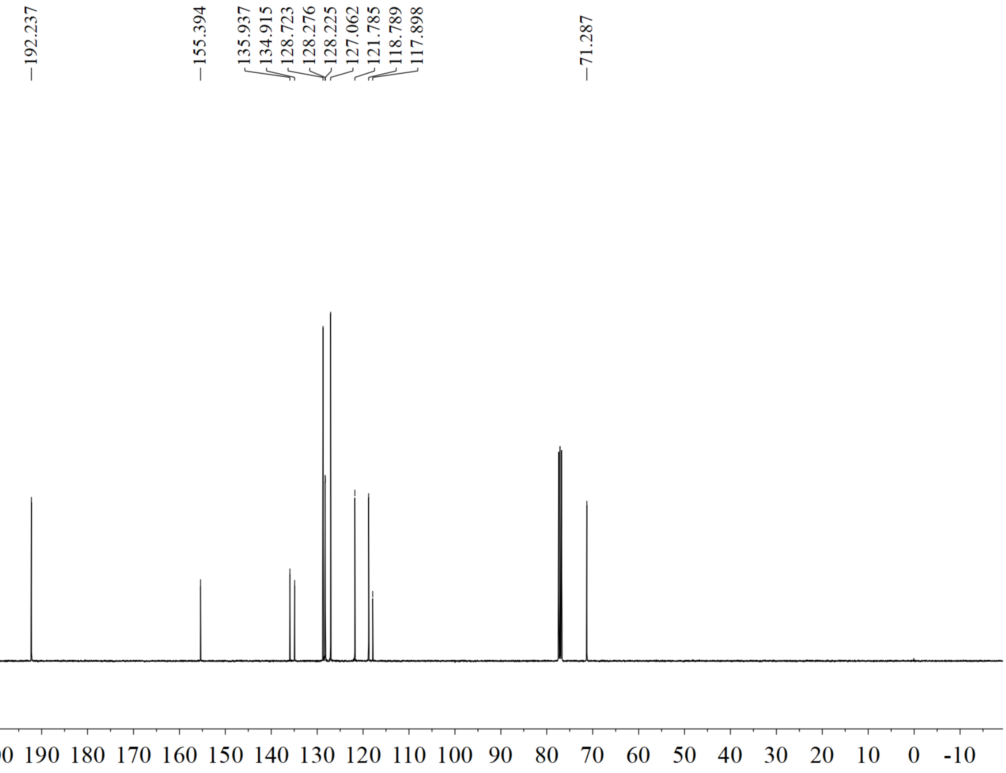
192.237
155.394
135.937
134.915
128.723
128.276
128.225
127.062
121.785
118.789
117.898
71.287
210 200 190 180 170 160 150 140 130 120 110 100 90 80 70 60 50 40 30 20 10 0 -10

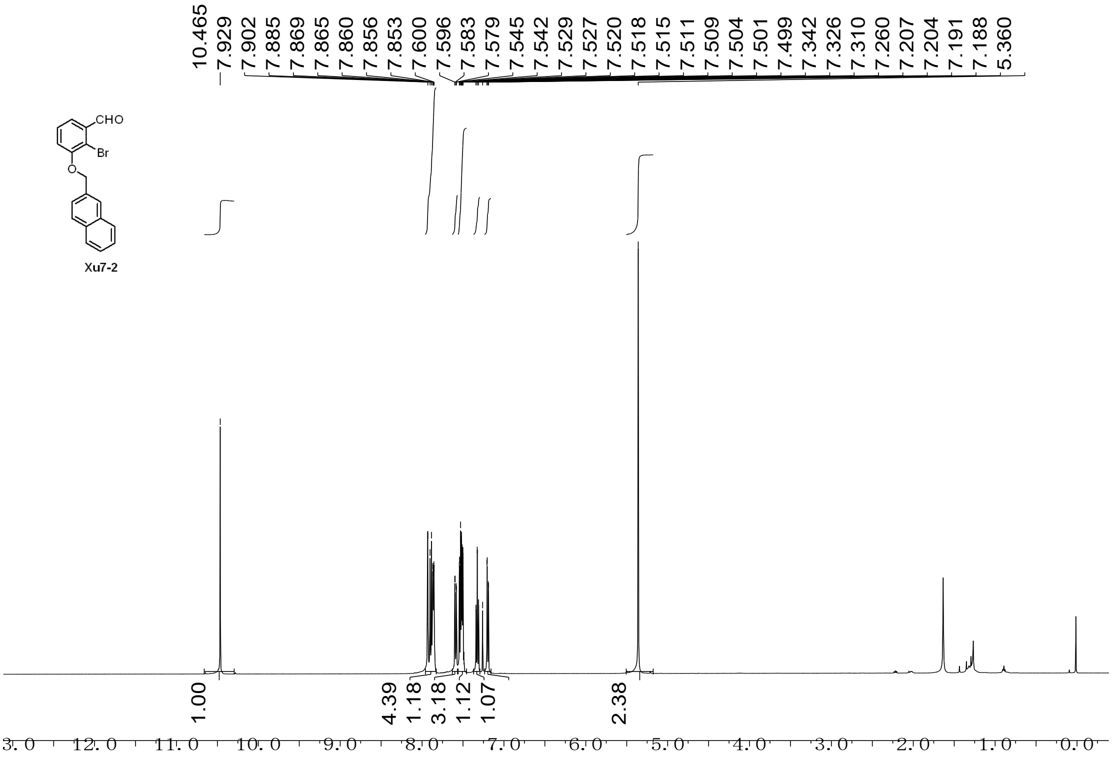
10.465
7.929
7.902
7.885
7.869
7.865
7.860
7.856
7.853
7.600
7.596
7.583
7.579
7.545
7.542
7.529
7.527
7.520
7.518
7.515
7.511
7.509
7.504
7.501
7.499
7.342
7.326
7.310
7.260
7.207
7.204
7.191
7.188
5.360
CHO
Br
O
Xu7-2
1.00
4.39
1.18
3.18
1.12
1.07
2.38
12.0
11.0
10.0
9.0
8.0
7.0
6.0
5.0
4.0
3.0
2.0
1.0
0.0

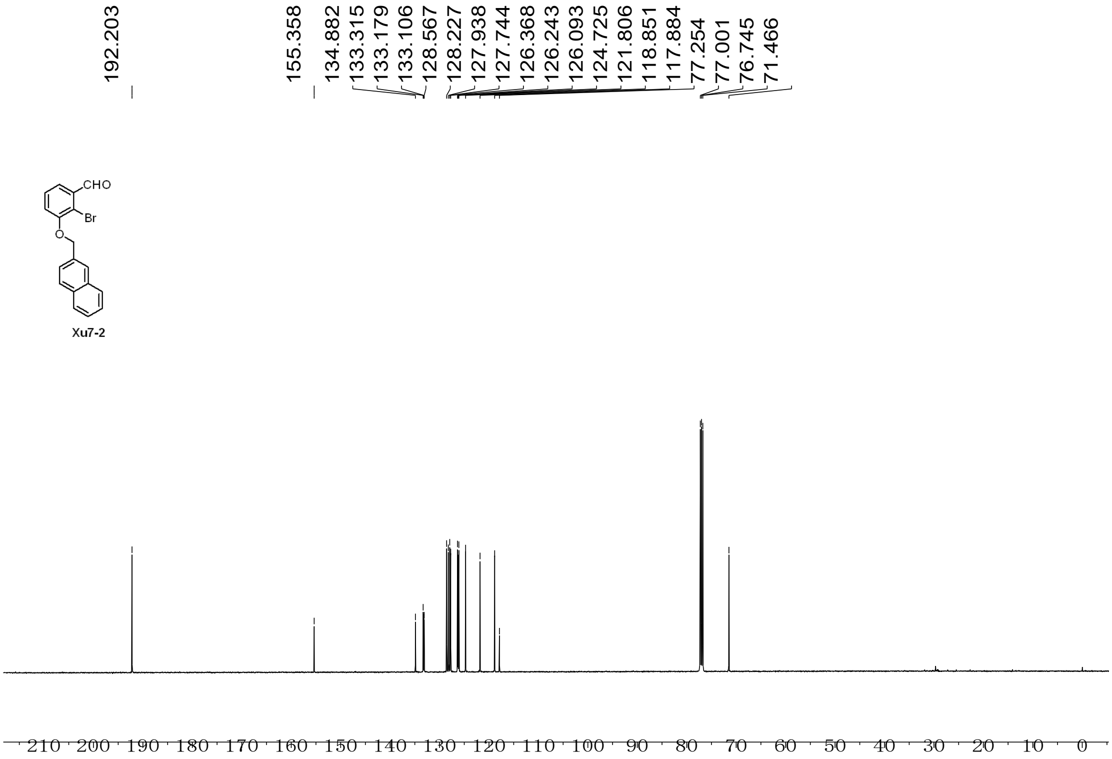
192.203
155.358
134.882
133.315
133.179
133.106
128.567
128.227
127.938
127.744
126.368
126.243
126.093
124.725
121.806
118.851
117.884
77.254
77.001
76.745
71.466
CHO
Br
O
Xu7-2
210
200
190
180
170
160
150
140
130
120
110
100
90
80
70
60
50
40
30
20
10
0

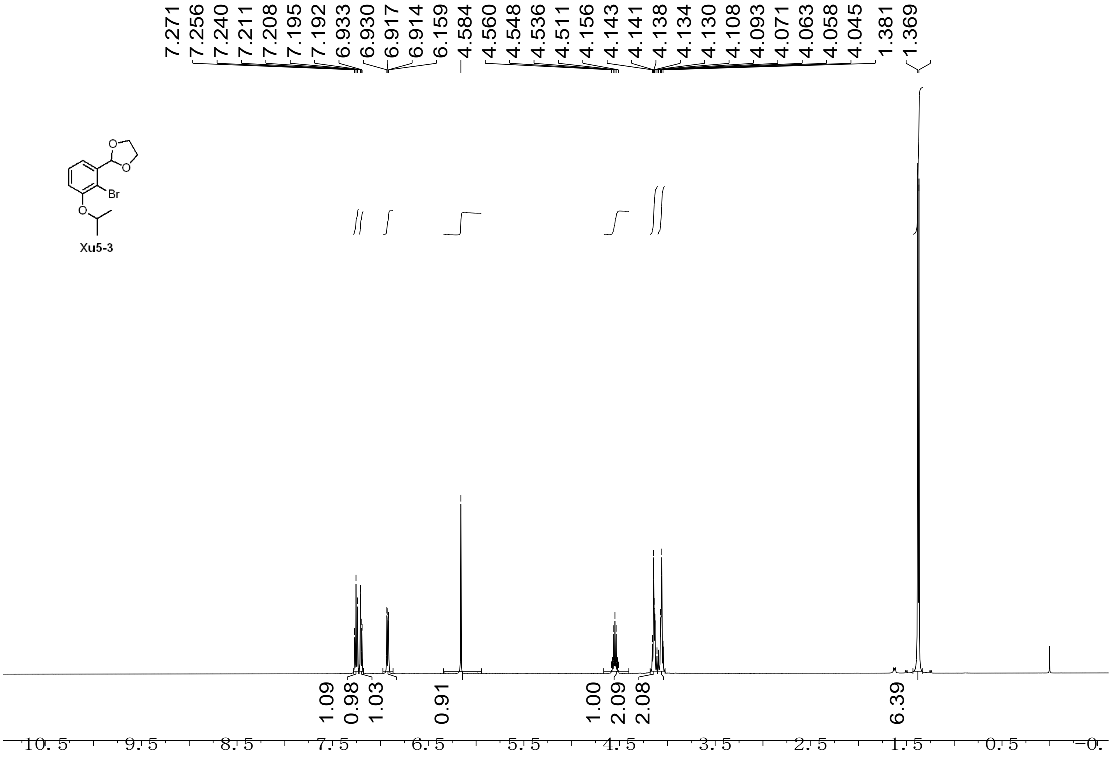
7.271
7.256
7.240
7.211
7.208
7.195
7.192
6.933
6.930
6.917
6.914
6.159
4.584
4.560
4.548
4.536
4.511
4.156
4.143
4.141
4.138
4.134
4.130
4.108
4.093
4.071
4.063
4.058
4.045
1.381
1.369
Br
Xu5-3
1.09
0.98
1.03
0.91
1.00
2.09
2.08
6.39

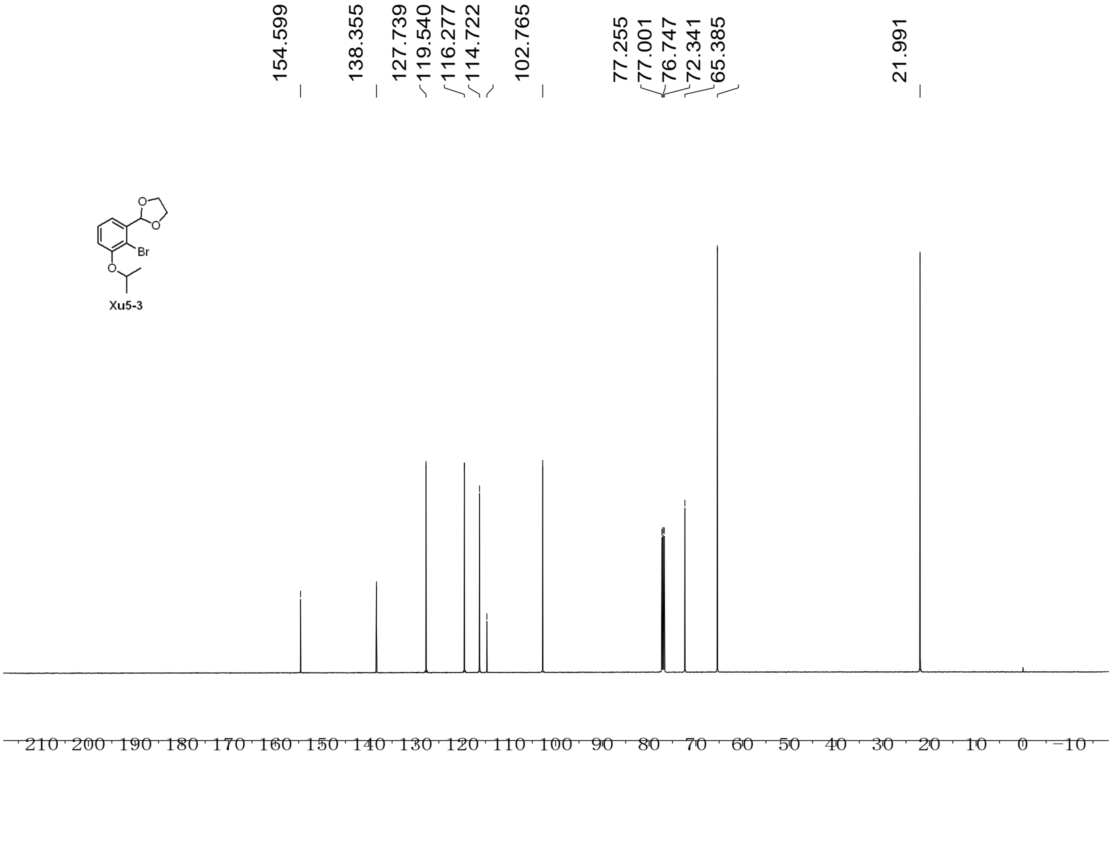
154.599
138.355
127.739
119.540
116.277
114.722
102.765
77.255
77.001
76.747
72.341
65.385
21.991
Br
Xu5-3

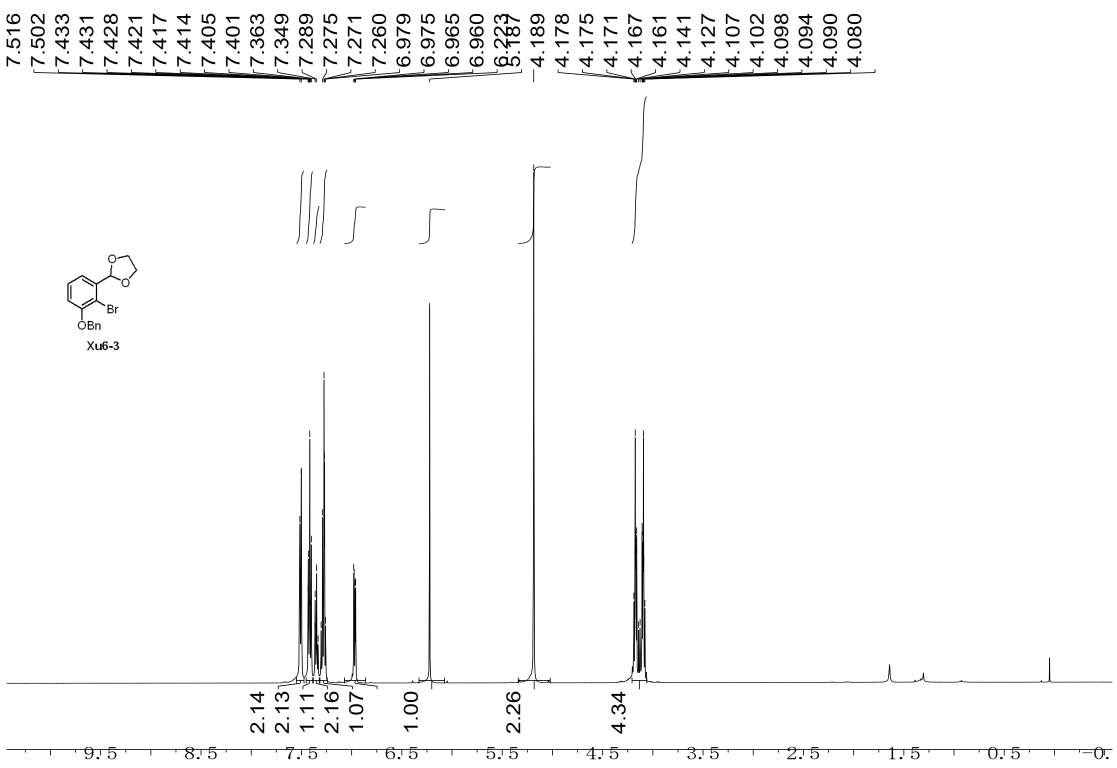
7.516
7.502
7.433
7.431
7.428
7.421
7.417
7.414
7.405
7.401
7.363
7.349
7.289
7.275
7.271
7.260
6.979
6.975
6.965
6.960
6.223
5.187
4.189
4.178
4.175
4.171
4.167
4.161
4.141
4.127
4.107
4.102
4.098
4.094
4.090
4.080
Br
OBn
Xu6-3
2.14
2.13
1.11
2.16
1.07
1.00
2.26
4.34

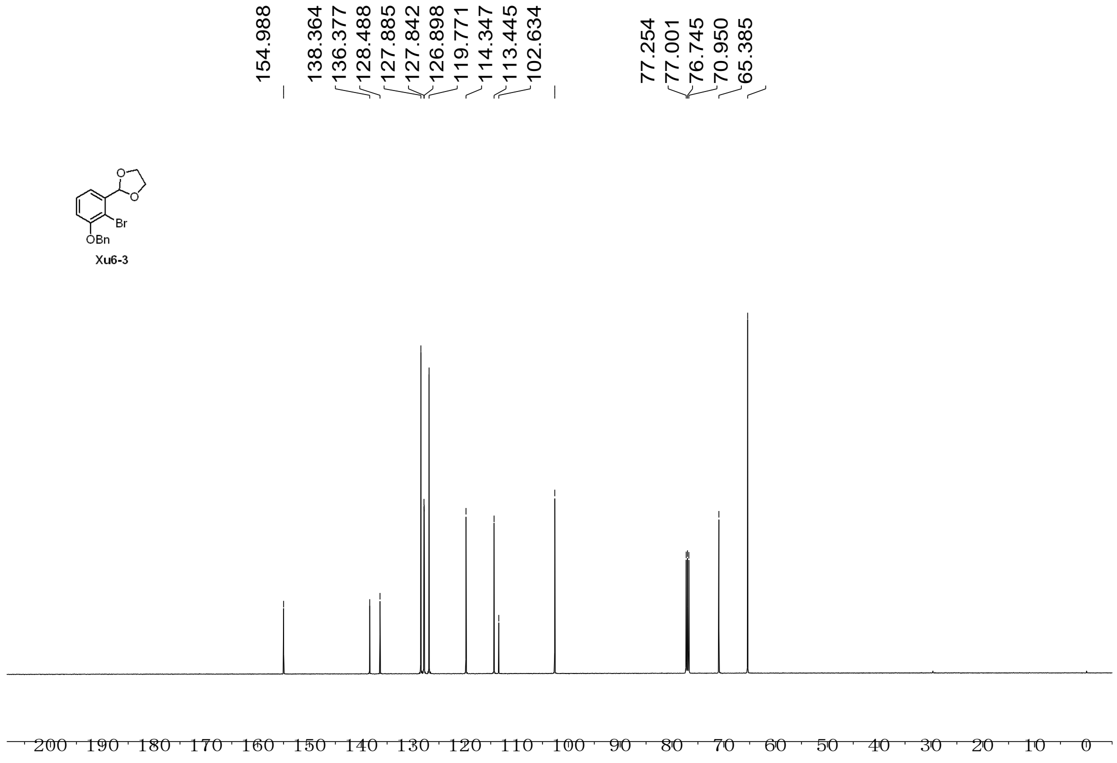
154.988
138.364
136.377
128.488
127.885
127.842
126.898
119.771
114.347
113.445
102.634
77.254
77.001
76.745
70.950
65.385
Br
OBn
Xu6-3
200 190 180 170 160 150 140 130 120 110 100 90 80 70 60 50 40 30 20 10 0

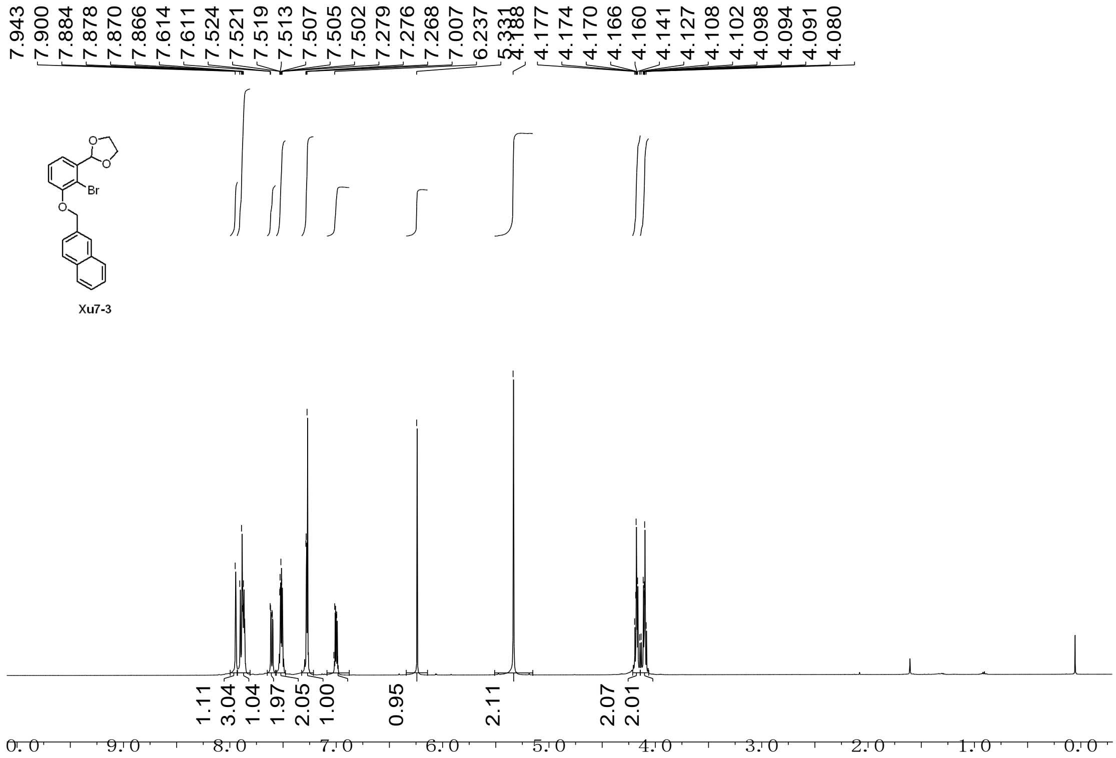
7.943
7.900
7.884
7.878
7.870
7.866
7.614
7.611
7.524
7.521
7.519
7.513
7.507
7.505
7.502
7.279
7.276
7.268
7.007
6.237
5.331
4.188
4.177
4.174
4.170
4.166
4.160
4.141
4.127
4.108
4.102
4.098
4.094
4.091
4.080
Br
O
Xu7-3
1.11
3.04
1.04
1.97
2.05
1.00
0.95
2.11
2.07
2.01
9.0
8.0
7.0
6.0
5.0
4.0
3.0
2.0
1.0
0.0

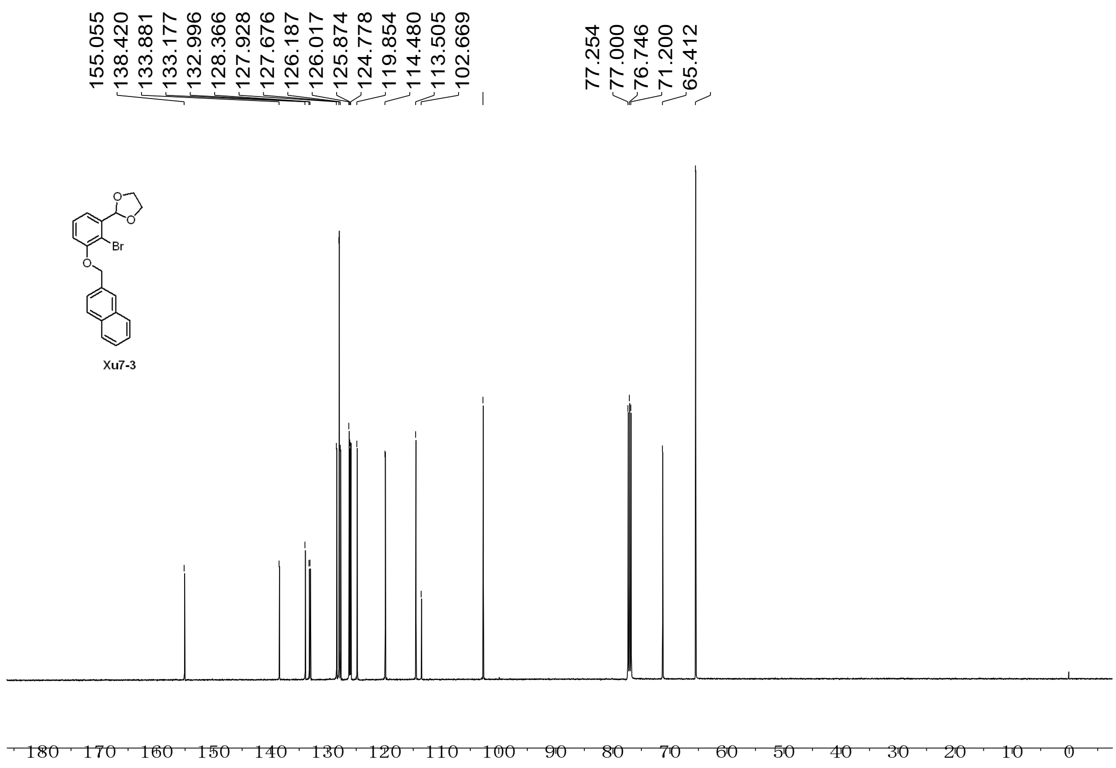
155.055
138.420
133.881
133.177
132.996
128.366
127.928
127.676
126.187
126.017
125.874
124.778
119.854
114.480
113.505
102.669
77.254
77.000
76.746
71.200
65.412
Br
O
Xu7-3
180
170
160
150
140
130
120
110
100
90
80
70
60
50
40
30
20
10
0

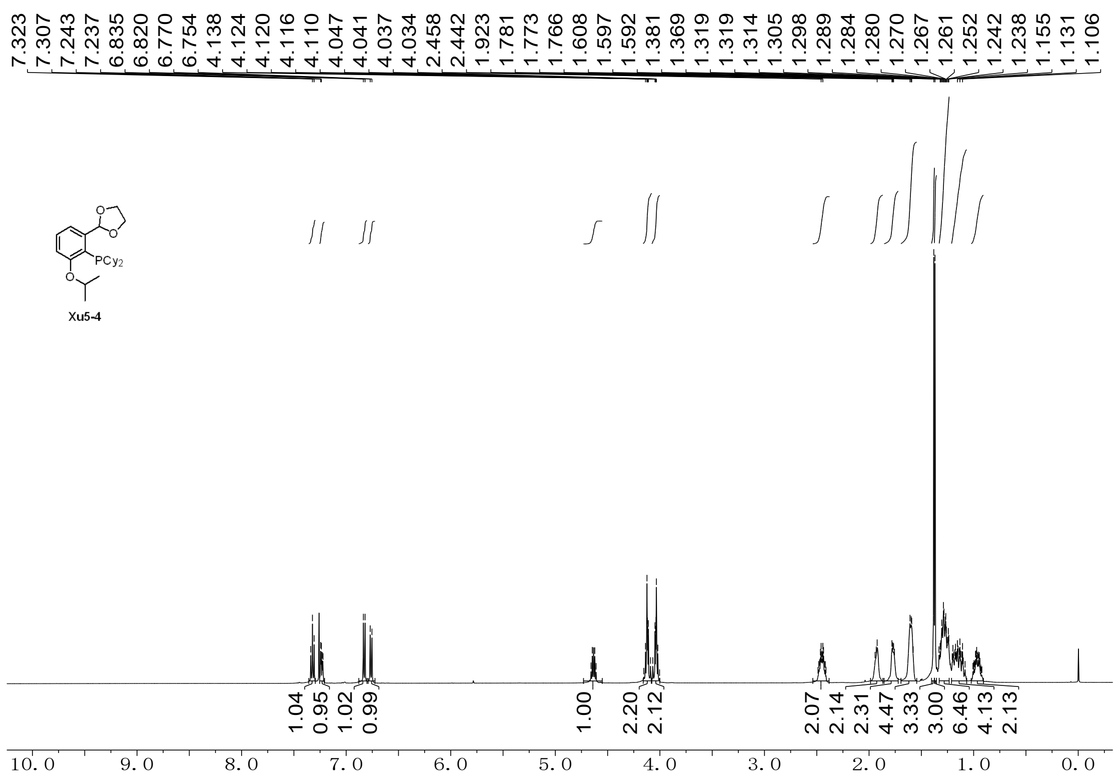
7.323
7.307
7.243
7.237
6.835
6.820
6.770
6.754
4.138
4.124
4.120
4.116
4.110
4.047
4.041
4.037
4.034
2.458
2.442
1.923
1.781
1.773
1.766
1.608
1.597
1.592
1.381
1.369
1.319
1.319
1.314
1.305
1.298
1.289
1.284
1.280
1.270
1.267
1.261
1.252
1.242
1.238
1.155
1.131
1.106
PCy2
Xu5-4
1.04
0.95
1.02
0.99
1.00
2.20
2.12
2.07
2.14
2.31
4.47
3.33
3.00
6.46
4.13
2.13
10.0
9.0
8.0
7.0
6.0
5.0
4.0
3.0
2.0
1.0
0.0

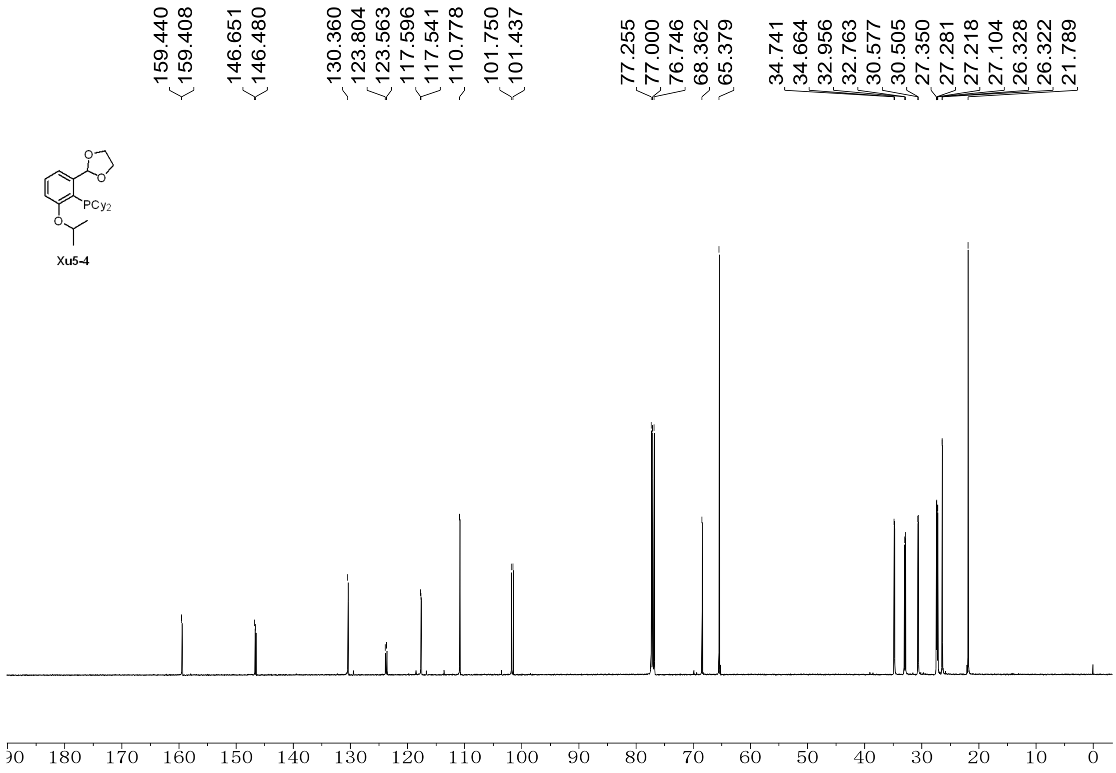
159.440
159.408
146.651
146.480
130.360
123.804
123.563
117.596
117.541
110.778
101.750
101.437
77.255
77.000
76.746
68.362
65.379
34.741
34.664
32.956
32.763
30.577
30.505
27.350
27.281
27.218
27.104
26.328
26.322
21.789
PCy2
Xu5-4
180
170
160
150
140
130
120
110
100
90
80
70
60
50
40
30
20
10
0

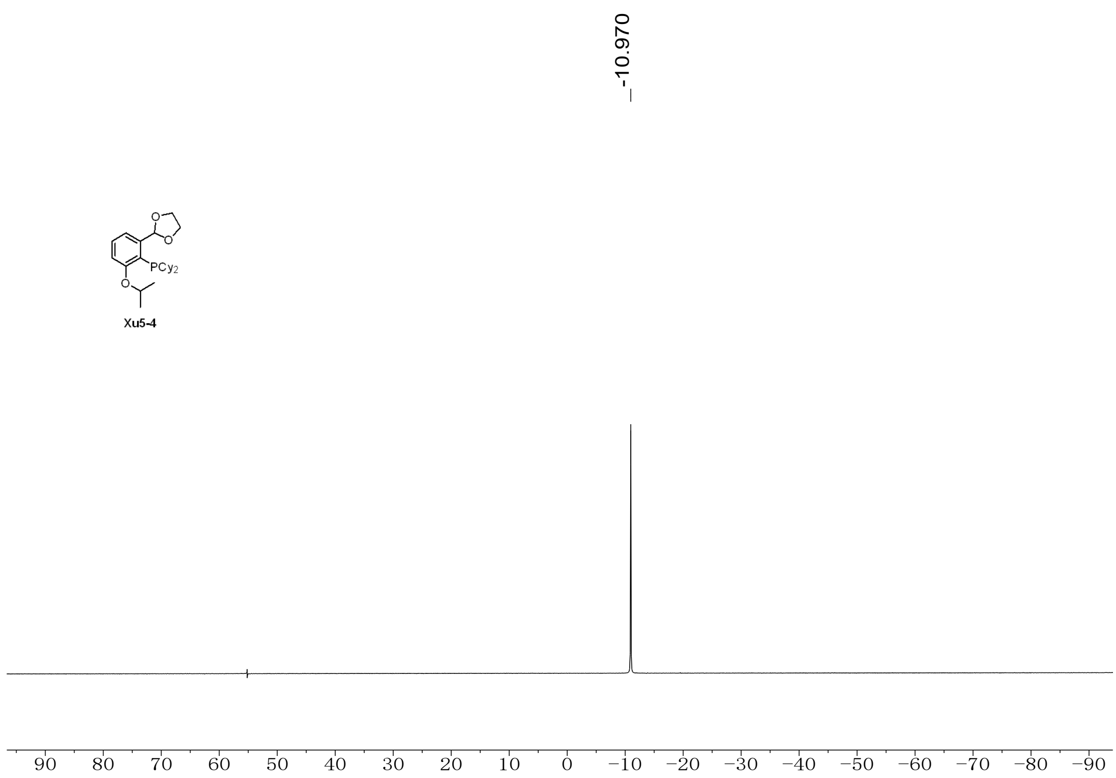

-10.970
PCy2
Xu5-4
90 80 70 60 50 40 30 20 10 0 −10 −20 −30 −40 −50 −60 −70 −80 −90

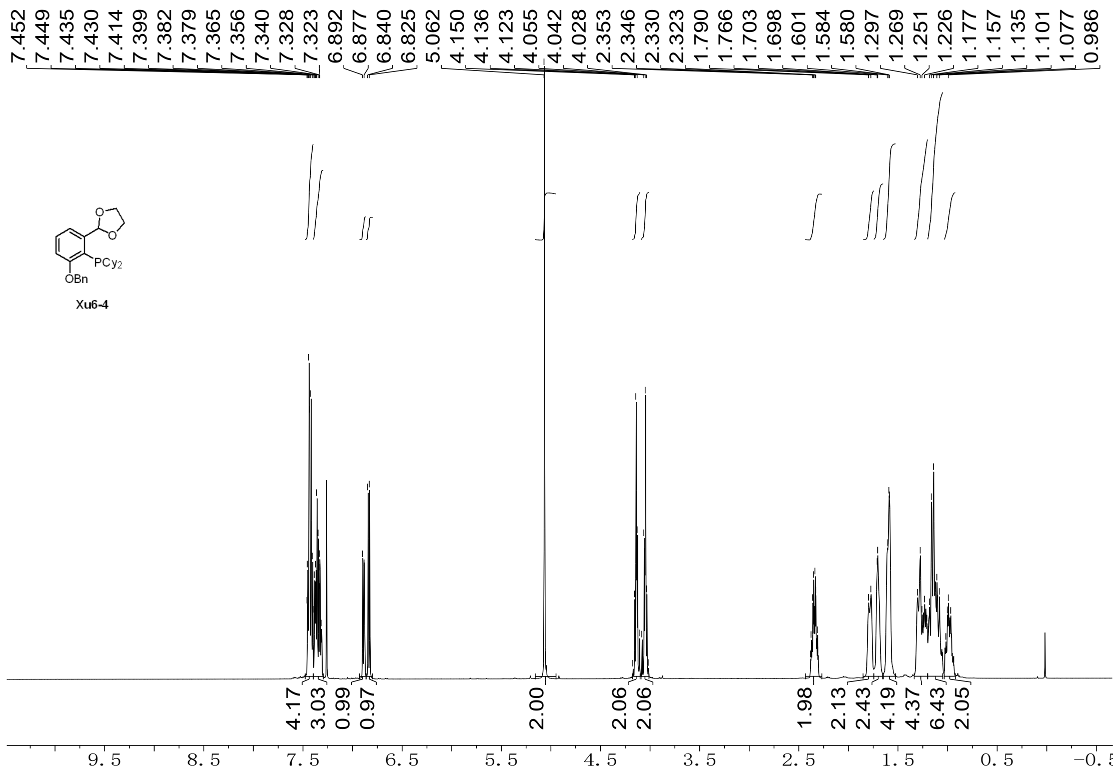

7.452 7.449 7.435 7.430 7.414 7.399 7.382 7.379 7.365 7.356 7.340 7.328 7.323 6.892 6.877 6.840 6.825 5.062 4.150 4.136 4.123 4.055 4.042 4.028 2.353 2.346 2.330 2.323 1.790 1.766 1.703 1.698 1.601 1.584 1.580 1.297 1.269 1.251 1.226 1.177 1.157 1.135 1.101 1.077 0.986
PCy2
OBn
Xu6-4
4.17 3.03 0.99 0.97 2.00 2.06 2.06 1.98 2.13 2.43 4.19 4.37 6.43 2.05
9.5 8.5 7.5 6.5 5.5 4.5 3.5 2.5 1.5 0.5 −0.5

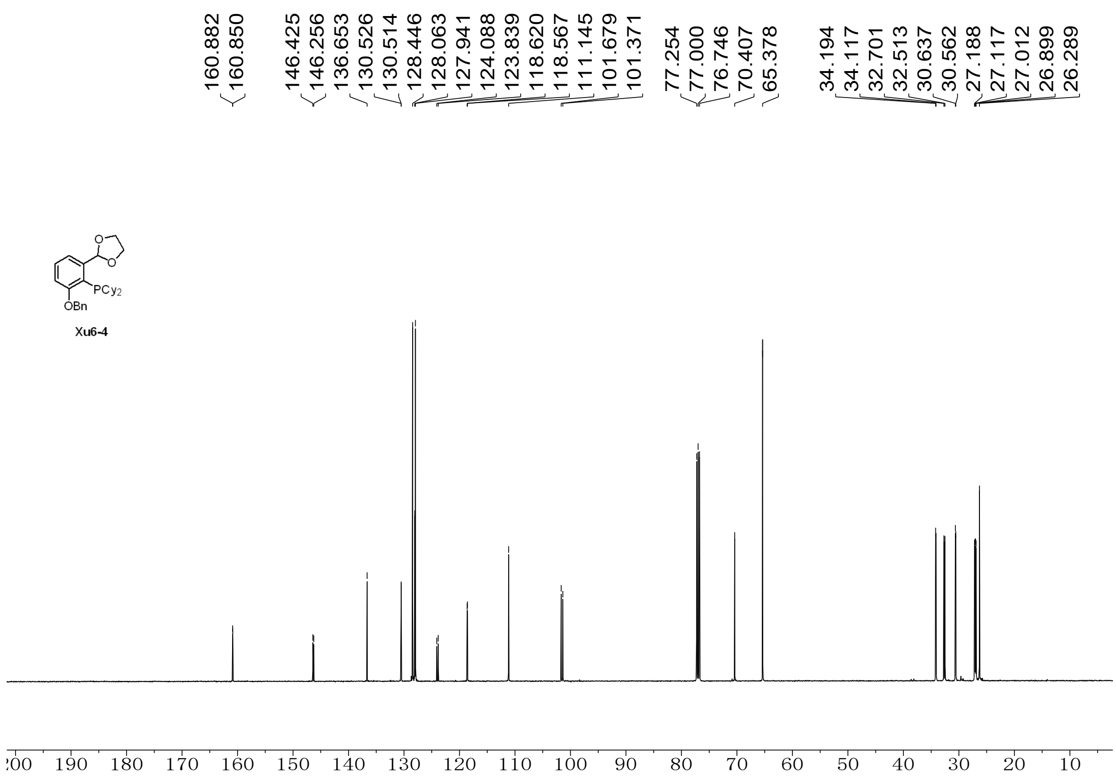
160.882
160.850
146.425
146.256
136.653
130.526
130.514
128.446
128.063
127.941
124.088
123.839
118.620
118.567
111.145
101.679
101.371
77.254
77.000
76.746
70.407
65.378
34.194
34.117
32.701
32.513
30.637
30.562
27.188
27.117
27.012
26.899
26.289
PCy2
OBn
Xu6-4
190 180 170 160 150 140 130 120 110 100 90 80 70 60 50 40 30 20 10

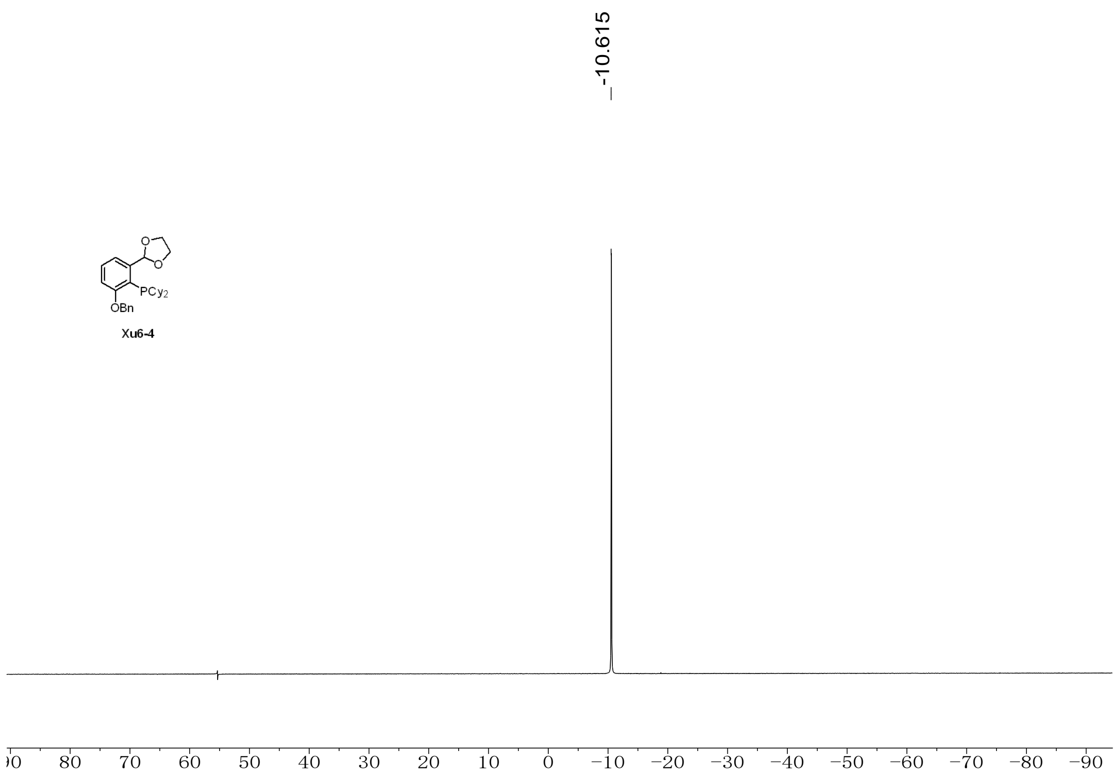
-10.615
PCy2
OBn
Xu6-4
80 70 60 50 40 30 20 10 0 −10 −20 −30 −40 −50 −60 −70 −80 −90

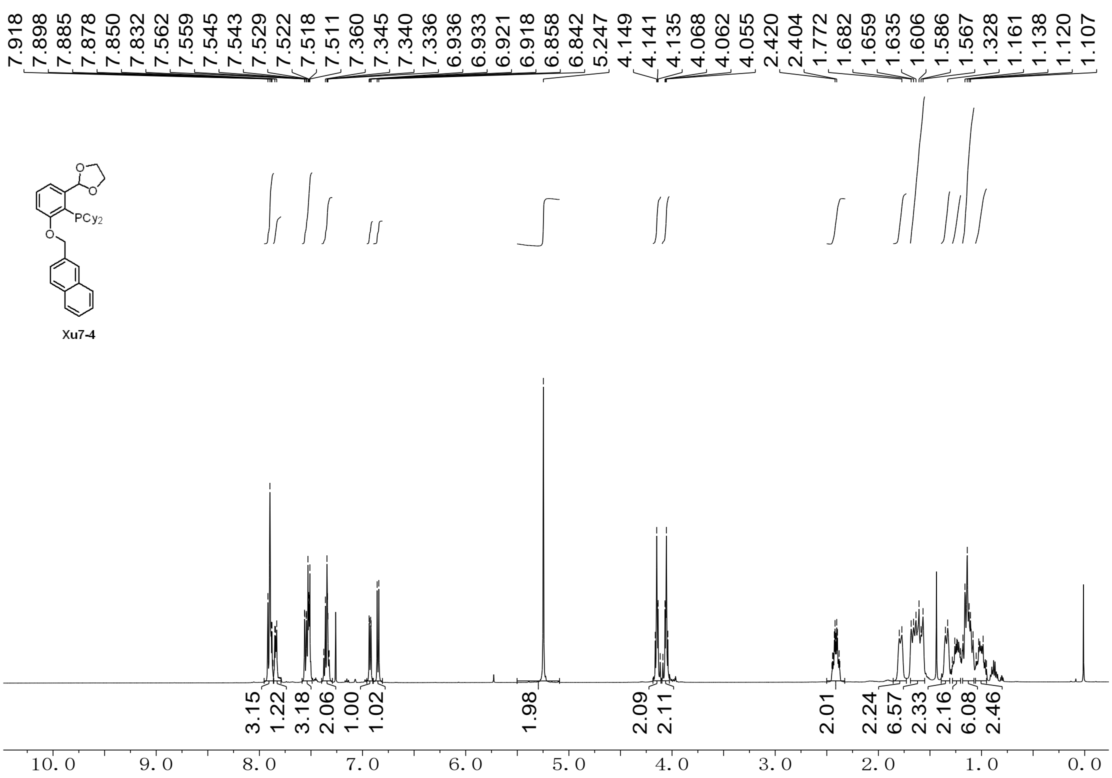
7.918 7.898 7.885 7.878 7.850 7.832 7.562 7.559 7.545 7.543 7.529 7.522 7.518 7.511 7.360 7.345 7.340 7.336 6.936 6.933 6.921 6.918 6.858 6.842 5.247 4.149 4.141 4.135 4.068 4.062 4.055 2.420 2.404 1.772 1.682 1.659 1.635 1.606 1.586 1.567 1.328 1.161 1.138 1.120 1.107
PCy2
Xu7-4
3.15 1.22 3.18 2.06 1.00 1.02 1.98 2.09 2.11 2.01 2.24 6.57 2.33 2.16 6.08 2.46
10.0 9.0 8.0 7.0 6.0 5.0 4.0 3.0 2.0 1.0 0.0

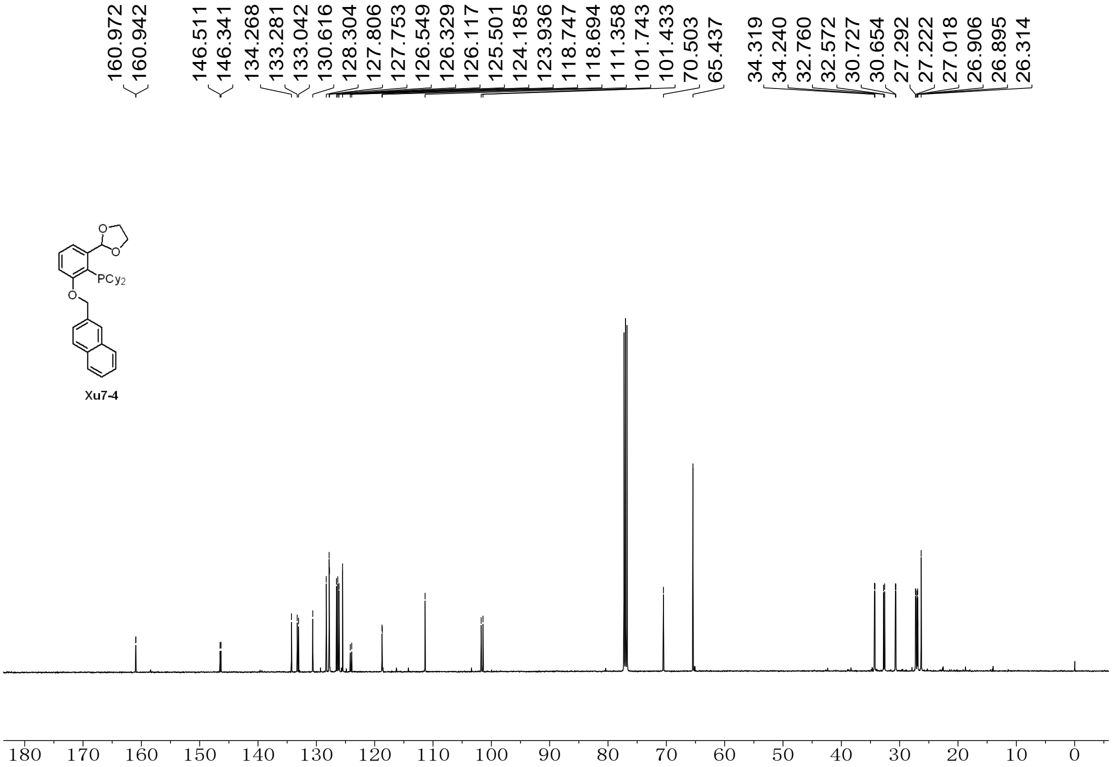
160.972 160.942 146.511 146.341 134.268 133.281 133.042 130.616 128.304 127.806 127.753 126.549 126.329 126.117 125.501 124.185 123.936 118.747 118.694 111.358 101.743 101.433 70.503 65.437 34.319 34.240 32.760 32.572 30.727 30.654 27.292 27.222 27.018 26.906 26.895 26.314
PCy2
Xu7-4
180 170 160 150 140 130 120 110 100 90 80 70 60 50 40 30 20 10 0

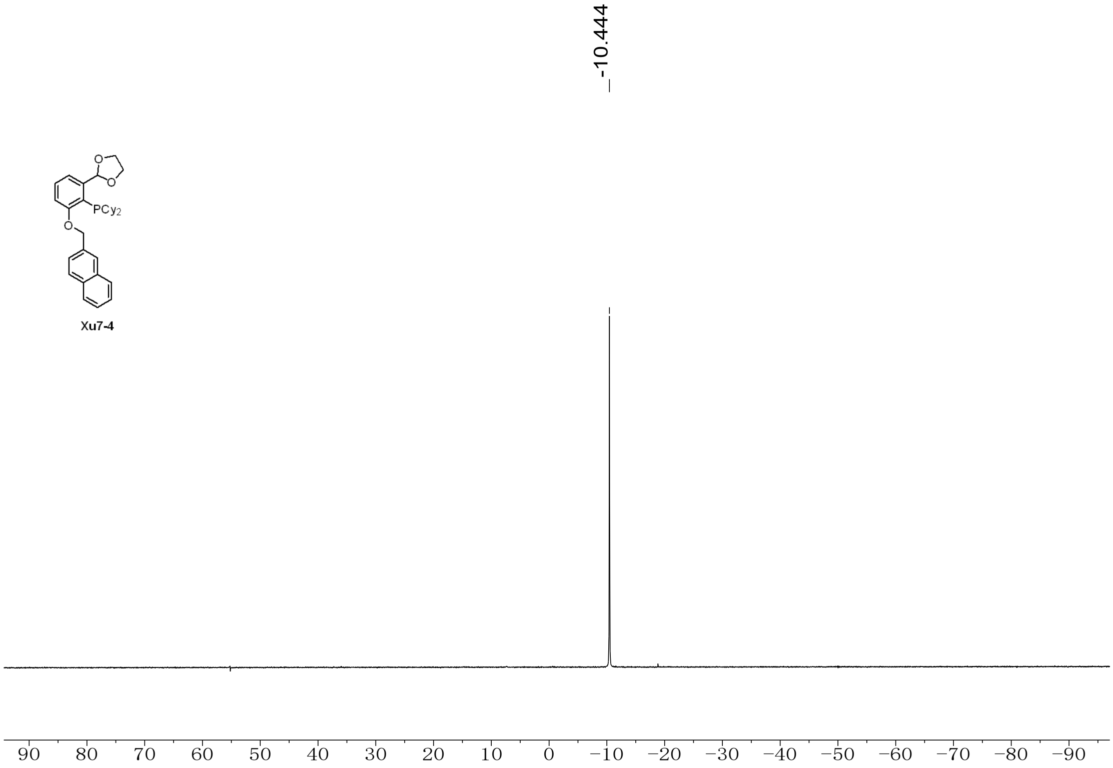
-10.444
PCy2
Xu7-4
90 80 70 60 50 40 30 20 10 0 −10 −20 −30 −40 −50 −60 −70 −80 −90

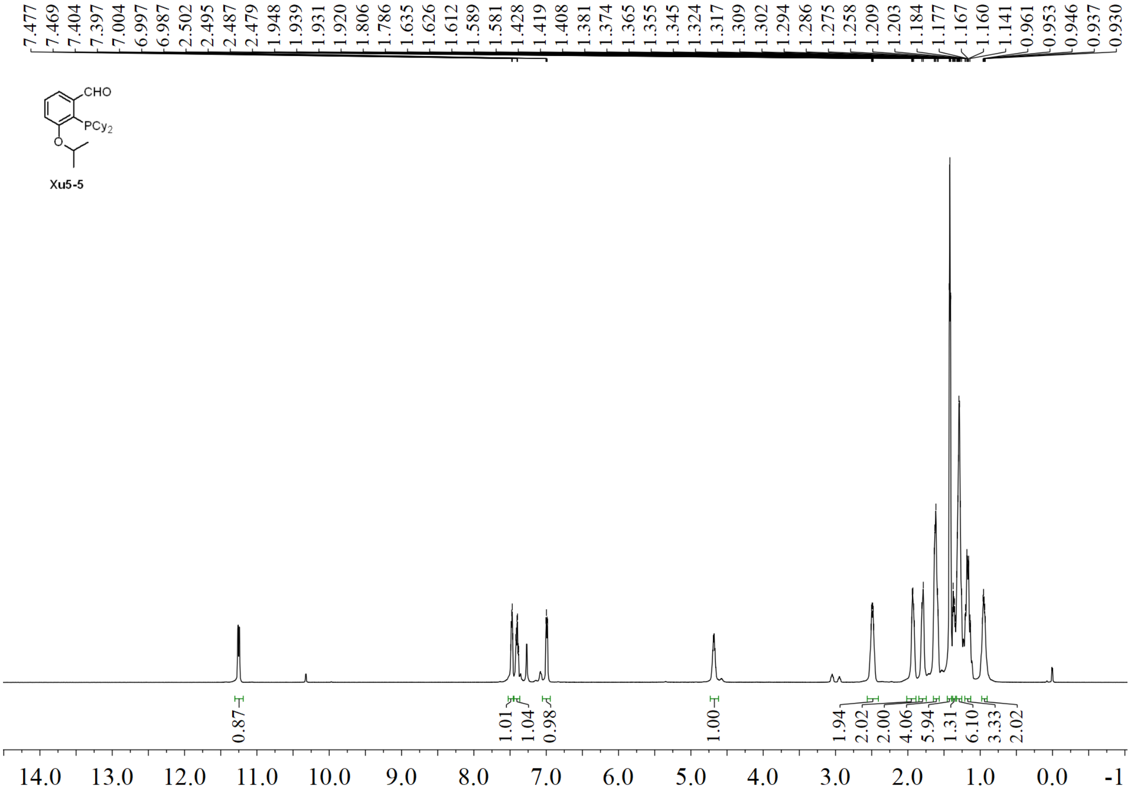

7.477
7.469
7.404
7.397
7.004
6.997
6.987
2.502
2.495
2.487
2.479
1.948
1.939
1.931
1.920
1.806
1.786
1.635
1.626
1.612
1.589
1.581
1.428
1.419
1.408
1.381
1.374
1.365
1.355
1.345
1.324
1.317
1.309
1.302
1.294
1.286
1.275
1.258
1.209
1.203
1.184
1.177
1.167
1.160
1.141
0.961
0.953
0.946
0.937
0.930
CHO
PCy2
O
Xu5-5
0.87
1.01
1.04
0.98
1.00
1.94
2.02
2.00
4.06
5.94
1.31
6.10
3.33
2.02
14.0 13.0 12.0 11.0 10.0 9.0 8.0 7.0 6.0 5.0 4.0 3.0 2.0 1.0 0.0 -1

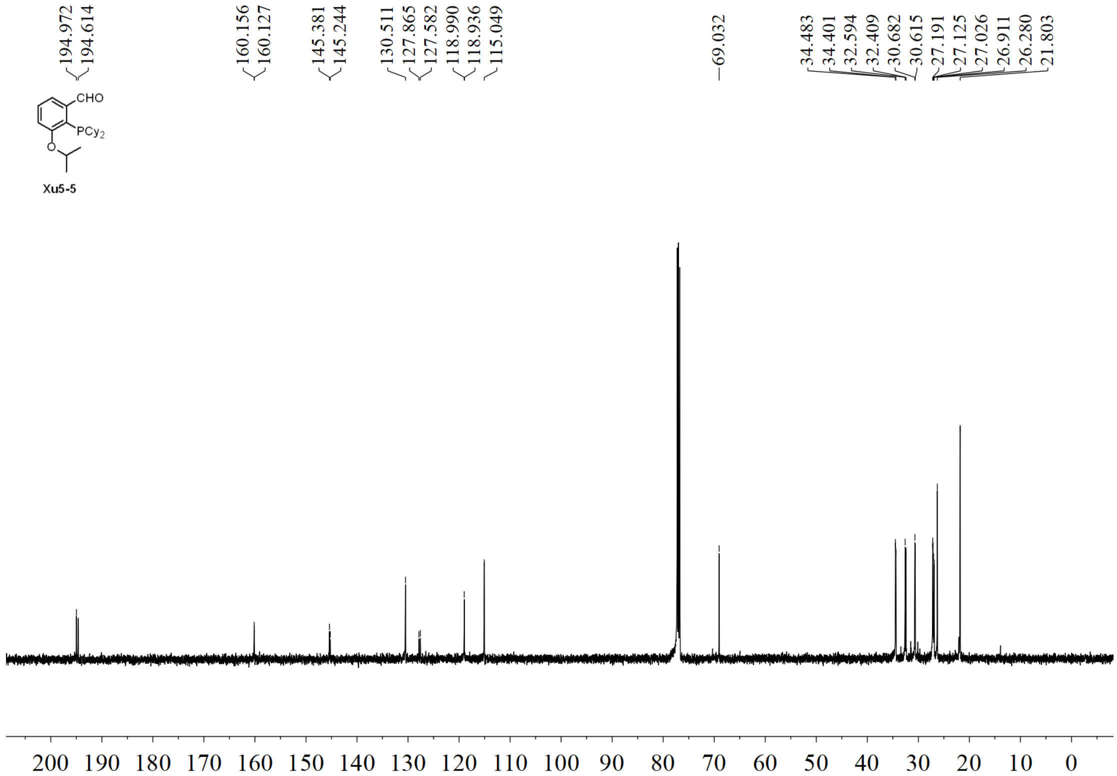

194.972
194.614
160.156
160.127
145.381
145.244
130.511
127.865
127.582
118.990
118.936
115.049
69.032
34.483
34.401
32.594
32.409
30.682
30.615
27.191
27.125
27.026
26.911
26.280
21.803
CHO
PCy2
O
Xu5-5
200 190 180 170 160 150 140 130 120 110 100 90 80 70 60 50 40 30 20 10 0

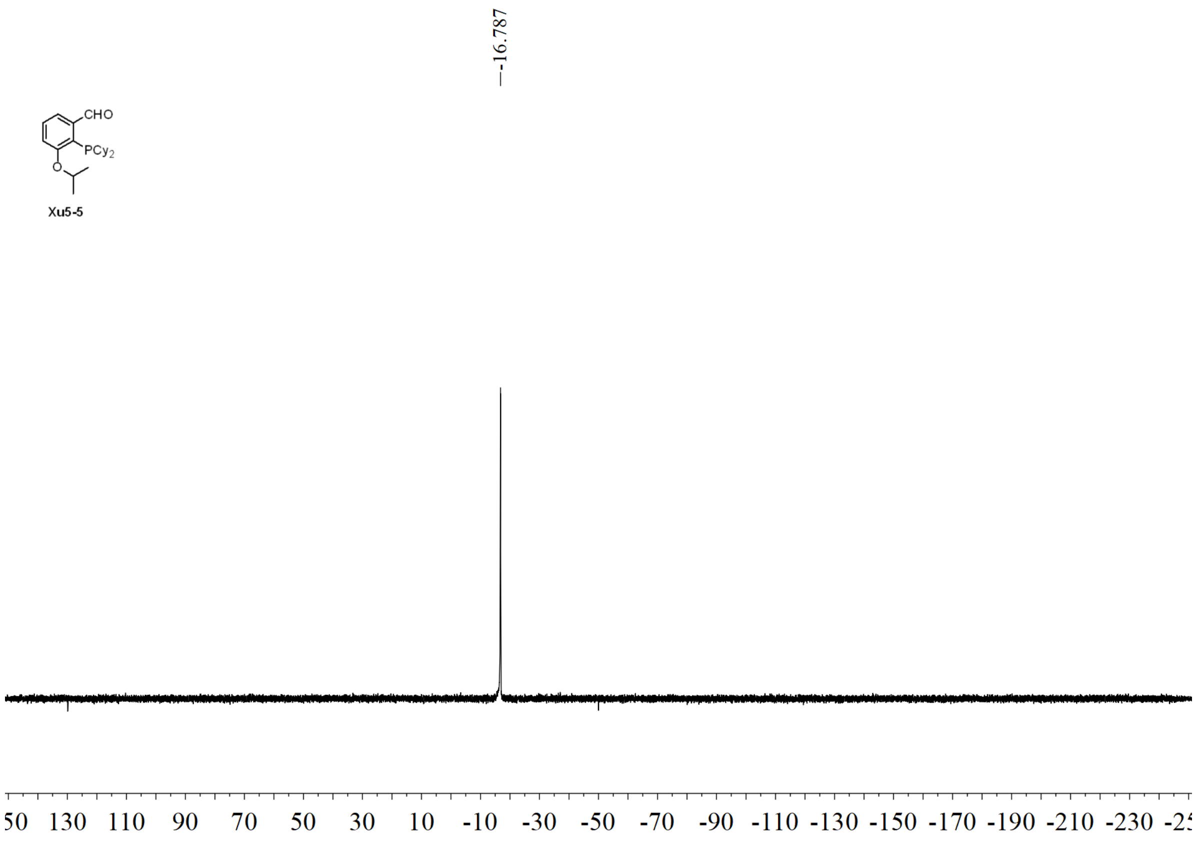
-16.787
CHO
PCy2
Xu5-5
50 130 110 90 70 50 30 10 -10 -30 -50 -70 -90 -110 -130 -150 -170 -190 -210 -230

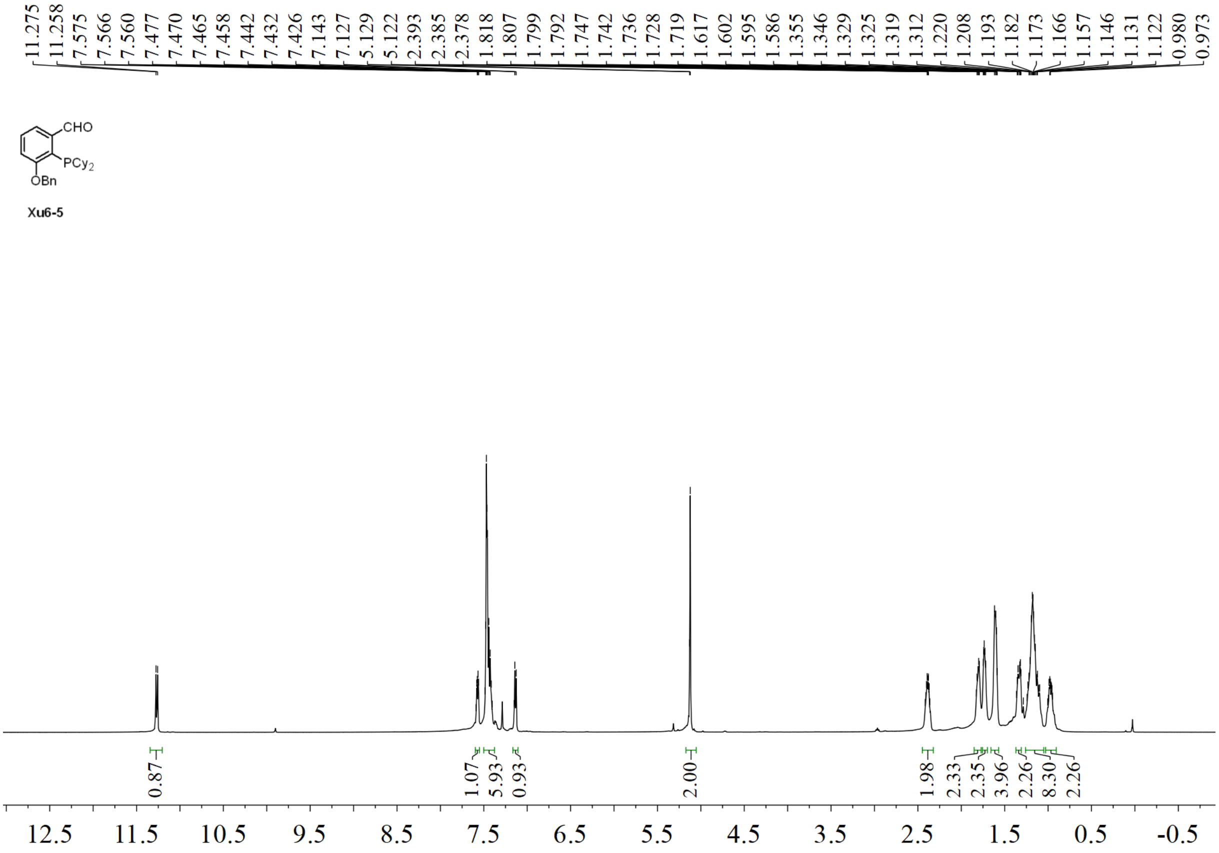
11.275
11.258
7.575
7.566
7.560
7.477
7.470
7.465
7.458
7.442
7.432
7.426
7.143
7.127
5.129
5.122
2.393
2.385
2.378
1.818
1.807
1.799
1.792
1.747
1.742
1.736
1.728
1.719
1.617
1.602
1.595
1.586
1.355
1.346
1.329
1.325
1.319
1.312
1.220
1.208
1.193
1.182
1.173
1.166
1.157
1.146
1.131
1.122
0.980
0.973
CHO
PCy2
OBn
Xu6-5
0.87
1.07
5.93
0.93
2.00
1.98
2.33
2.35
3.96
2.26
8.30
2.26
12.5 11.5 10.5 9.5 8.5 7.5 6.5 5.5 4.5 3.5 2.5 1.5 0.5 -0.5

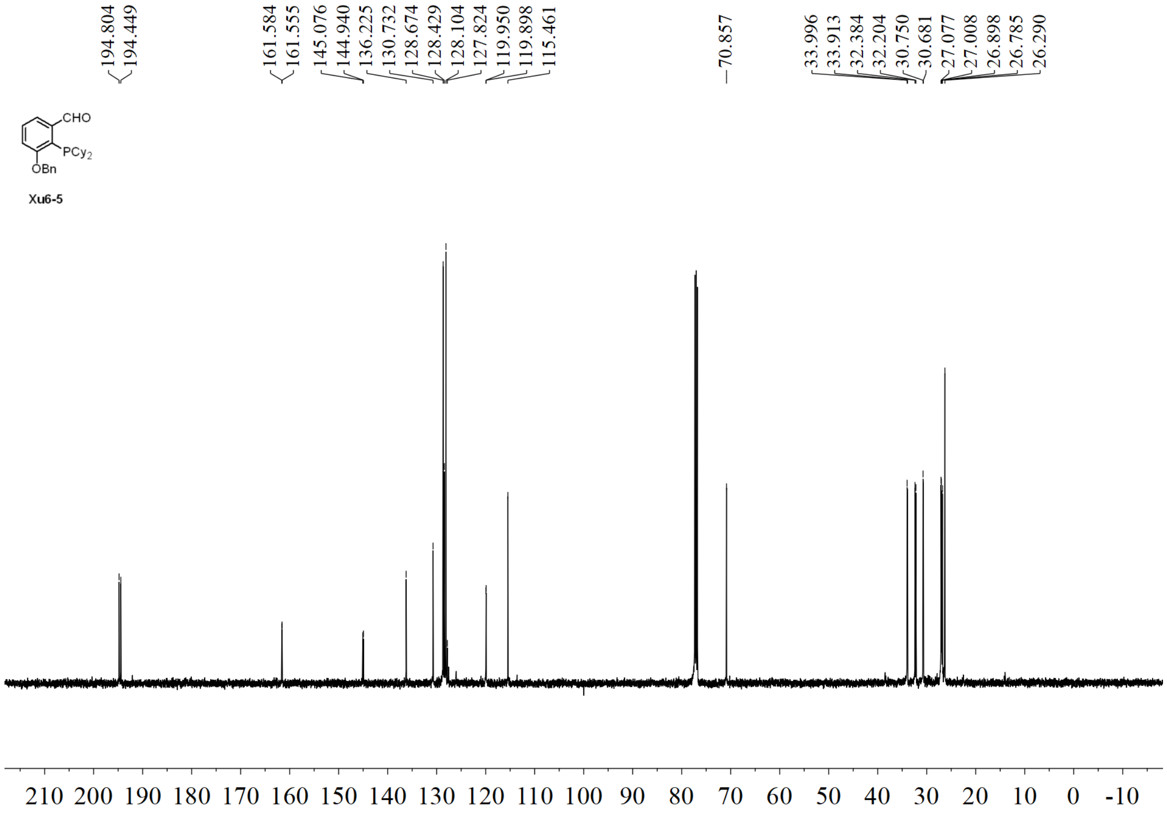
194.804
194.449
161.584
161.555
145.076
144.940
136.225
130.732
128.674
128.429
128.104
127.824
119.950
119.898
115.461
70.857
33.996
33.913
32.384
32.204
30.750
30.681
27.077
27.008
26.898
26.785
26.290
CHO
PCy2
OBn
Xu6-5
210 200 190 180 170 160 150 140 130 120 110 100 90 80 70 60 50 40 30 20 10 0 -10

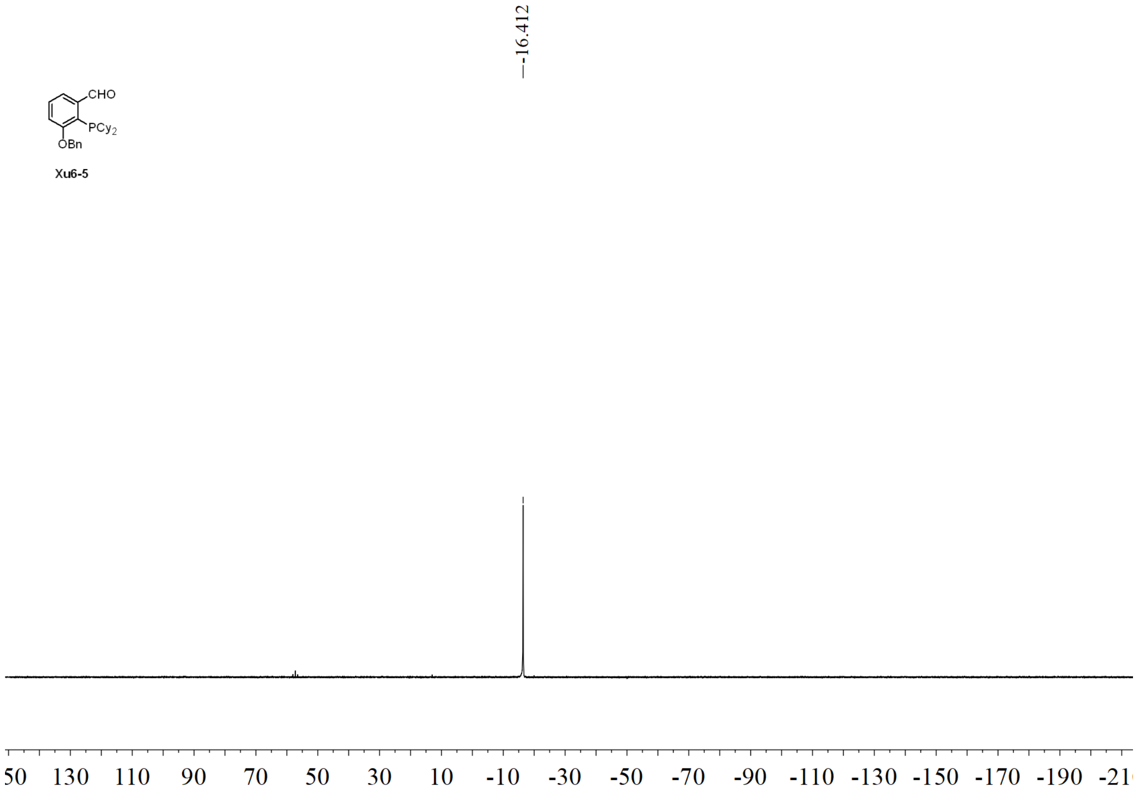
-16.412
CHO
PCy2
OBn
Xu6-5
130 110 90 70 50 30 10 -10 -30 -50 -70 -90 -110 -130 -150 -170 -190

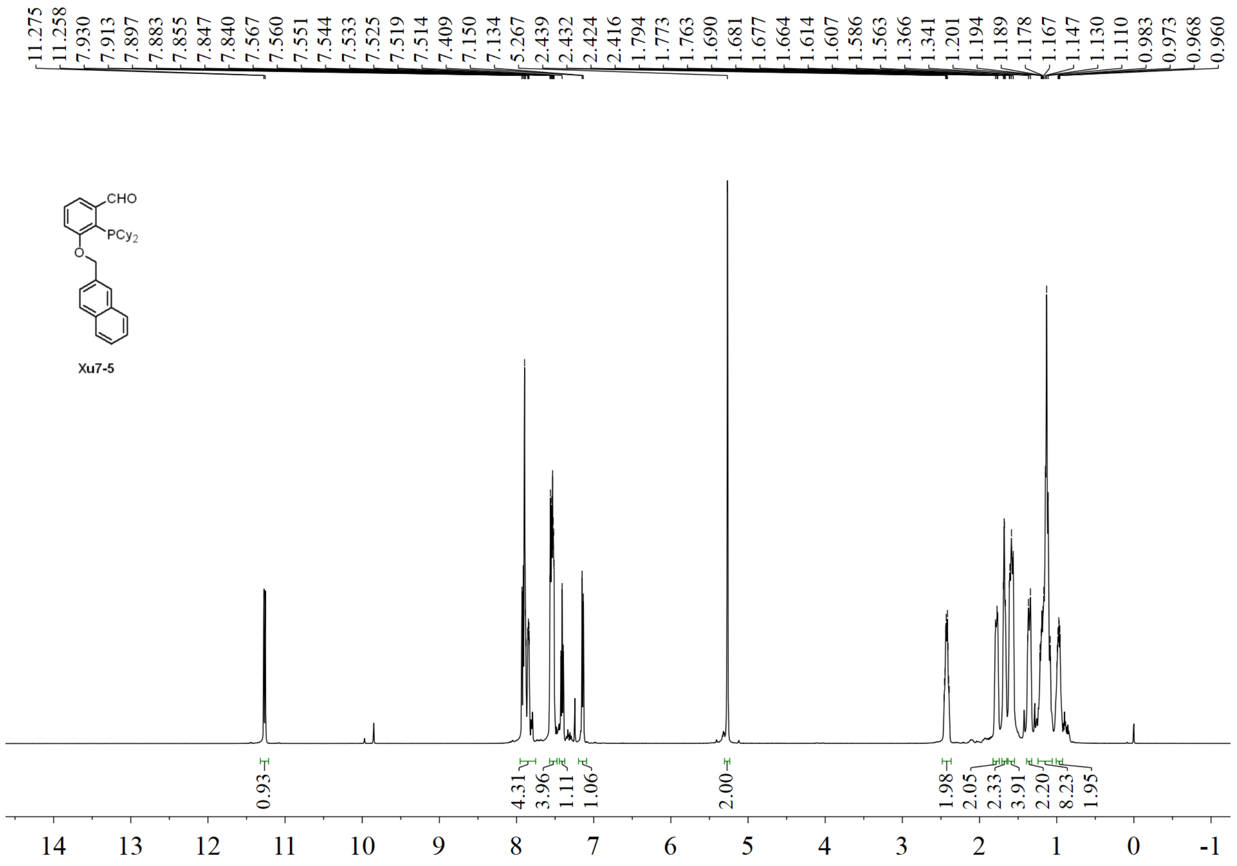
11.275
11.258
7.930
7.913
7.897
7.883
7.855
7.847
7.840
7.567
7.560
7.551
7.544
7.533
7.525
7.519
7.514
7.409
7.150
7.134
5.267
2.439
2.432
2.424
2.416
1.794
1.773
1.763
1.690
1.681
1.677
1.664
1.614
1.607
1.586
1.563
1.366
1.341
1.201
1.194
1.189
1.178
1.167
1.147
1.130
1.110
0.983
0.973
0.968
0.960
CHO
PCy2
O
Xu7-5
0.93
4.31
3.96
1.11
1.06
2.00
1.98
2.05
2.33
3.91
2.20
8.23
1.95
14
13
12
11
10
9
8
7
6
5
4
3
2
1
0
-1

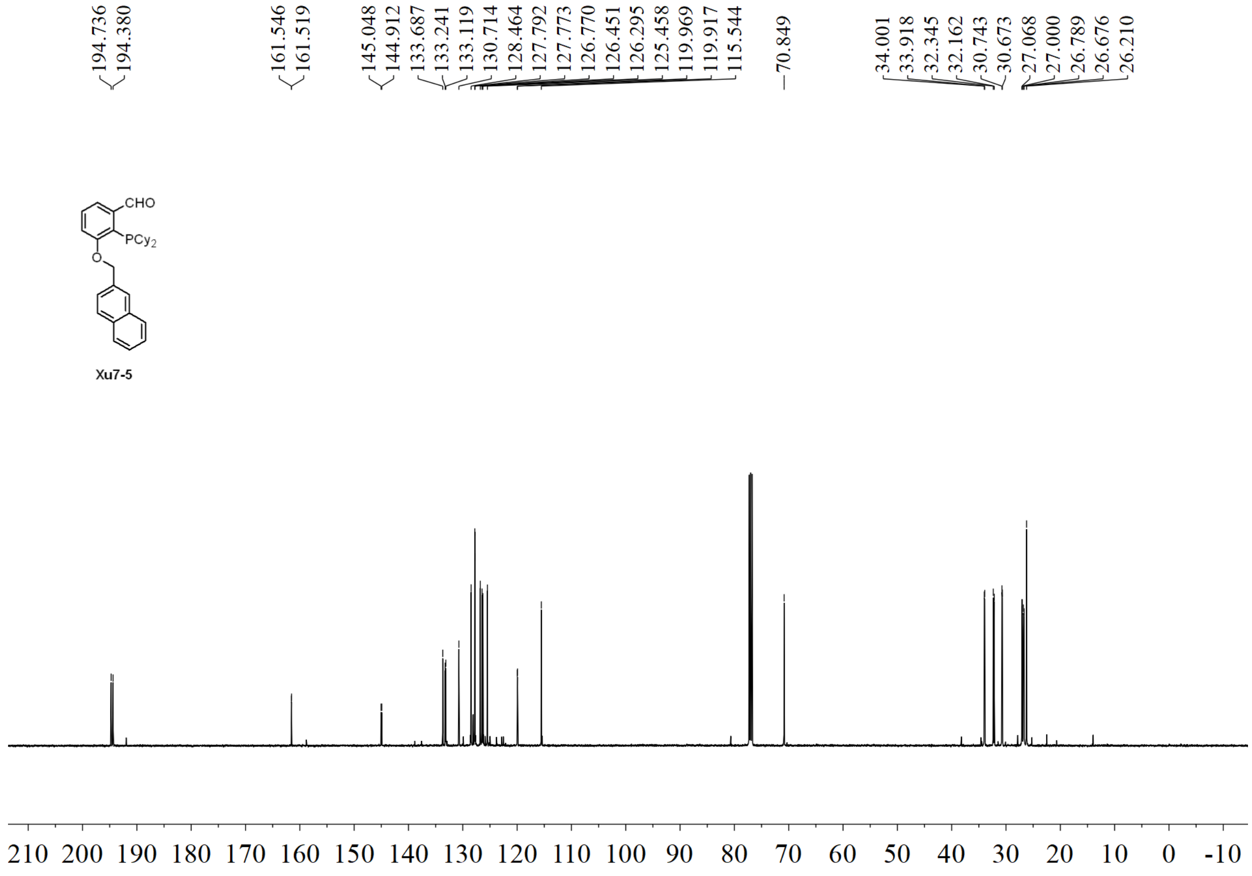
194.736
194.380
161.546
161.519
145.048
144.912
133.687
133.241
133.119
130.714
128.464
127.792
127.773
126.770
126.451
126.295
125.458
119.969
119.917
115.544
70.849
34.001
33.918
32.345
32.162
30.743
30.673
27.068
27.000
26.789
26.676
26.210
CHO
PCy2
O
Xu7-5
210
200
190
180
170
160
150
140
130
120
110
100
90
80
70
60
50
40
30
20
10
0
-10

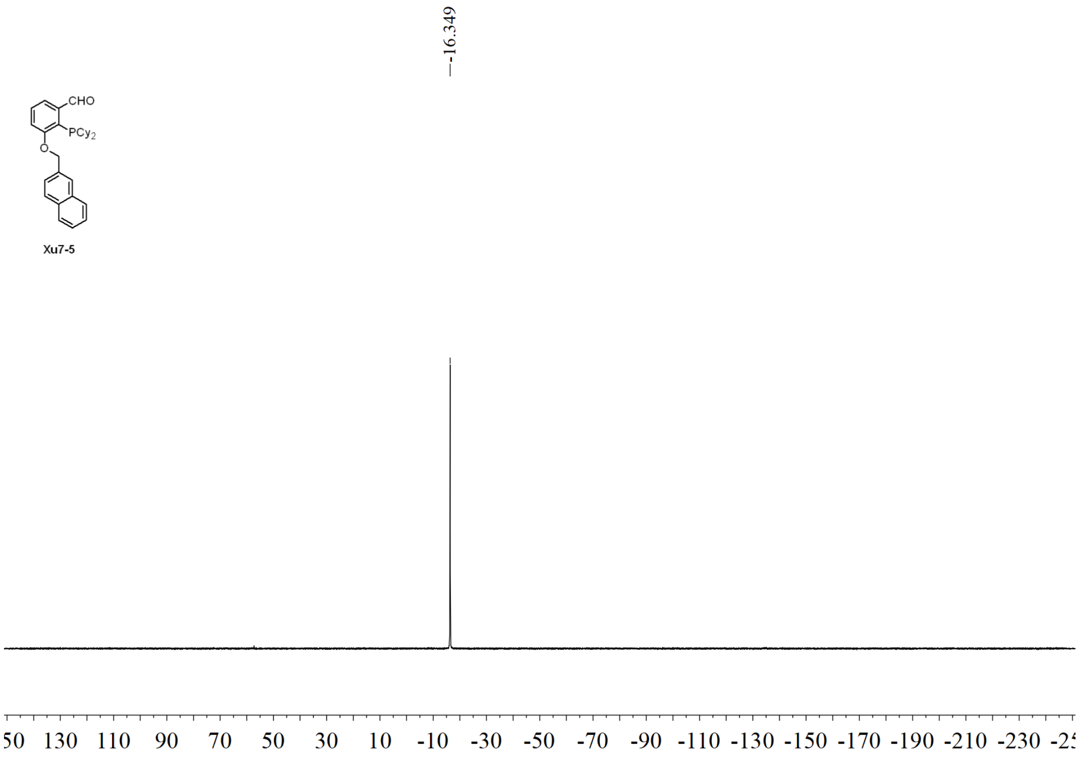
CHO
PCy2
O
Xu7-5
-16.349
50 130 110 90 70 50 30 10 -10 -30 -50 -70 -90 -110 -130 -150 -170 -190 -210 -230 -2

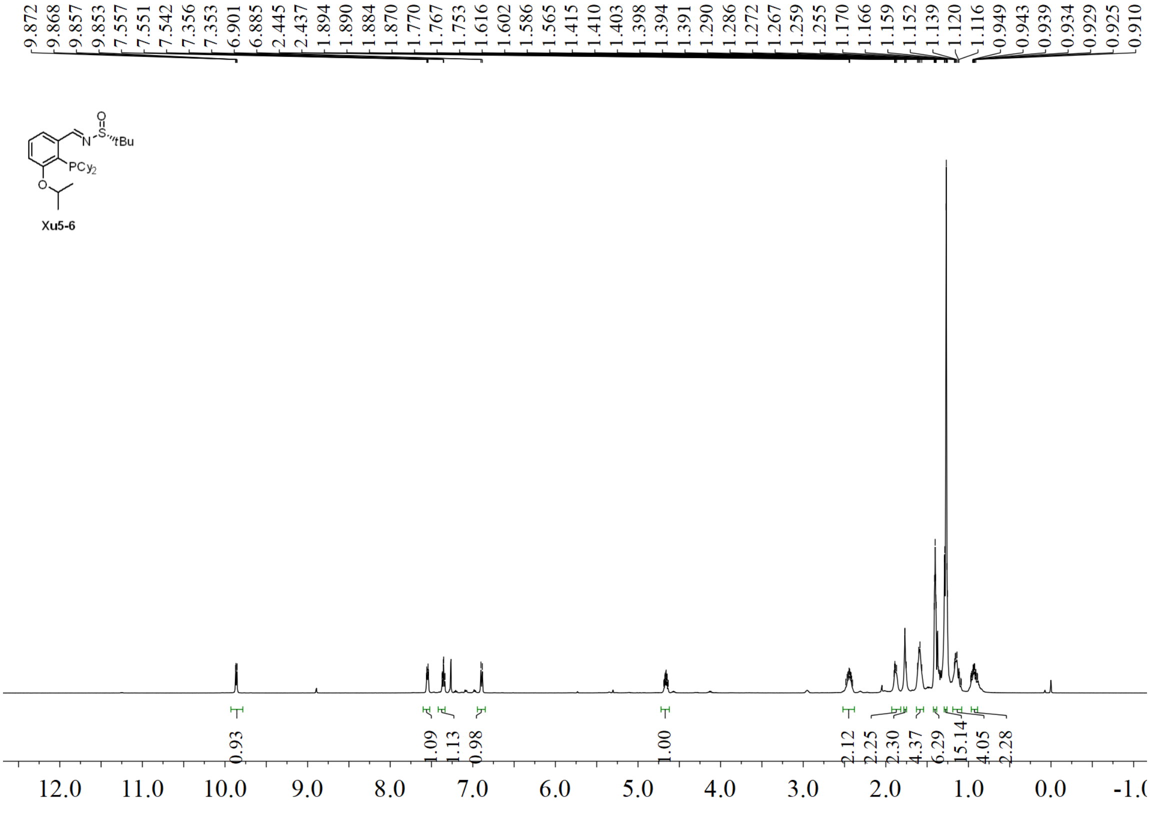
9.872
9.868
9.857
9.853
7.557
7.551
7.542
7.356
7.353
6.901
6.885
2.445
2.437
1.894
1.890
1.884
1.870
1.770
1.767
1.753
1.616
1.602
1.586
1.565
1.415
1.410
1.403
1.398
1.394
1.391
1.290
1.286
1.272
1.267
1.259
1.255
1.170
1.166
1.159
1.152
1.139
1.120
1.116
0.949
0.943
0.939
0.934
0.929
0.925
0.910
O
N
S
tBu
PCy2
O
Xu5-6
0.93
1.09
1.13
0.98
1.00
2.12
2.25
2.30
4.37
6.29
15.14
4.05
2.28
12.0 11.0 10.0 9.0 8.0 7.0 6.0 5.0 4.0 3.0 2.0 1.0 0.0 -1.

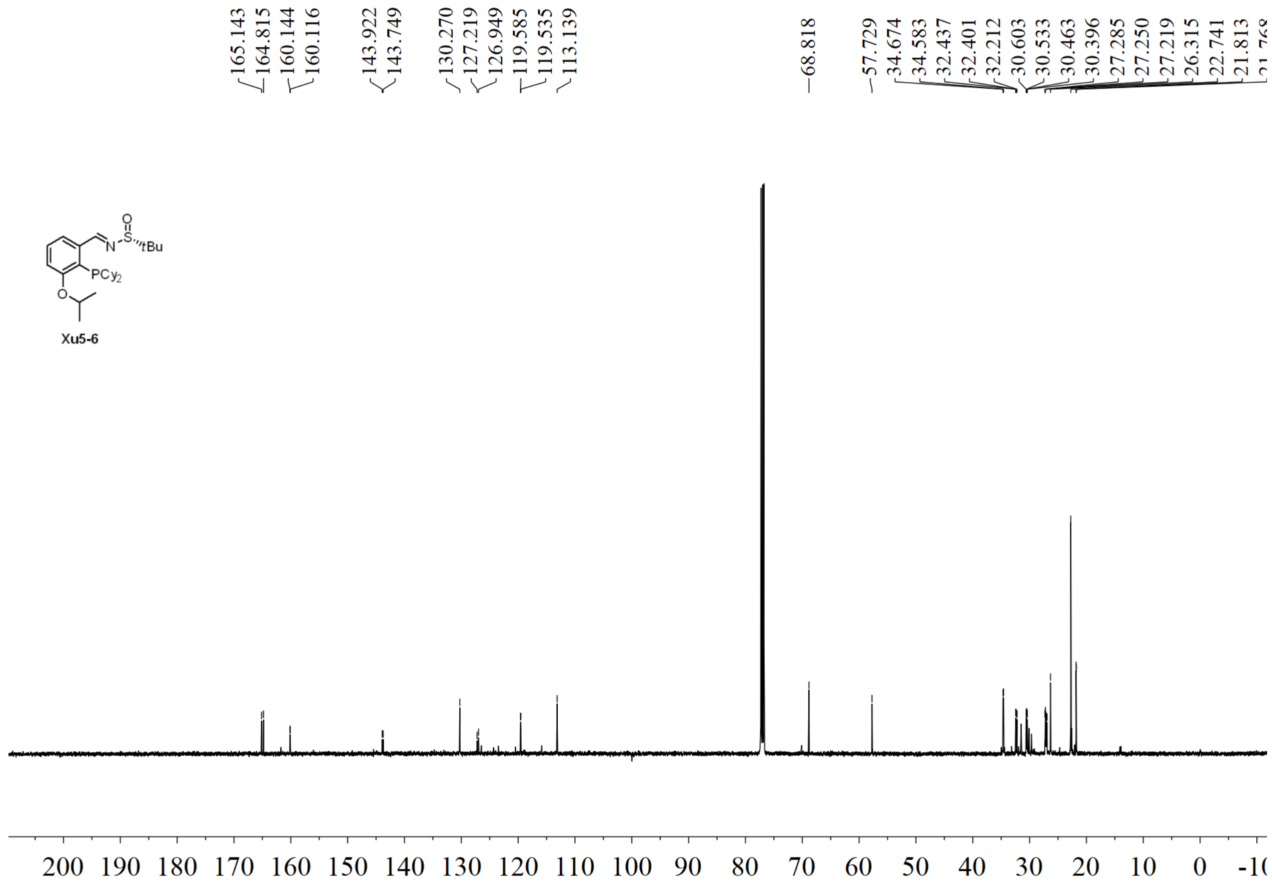
165.143
164.815
160.144
160.116
143.922
143.749
130.270
127.219
126.949
119.585
119.535
113.139
68.818
57.729
34.674
34.583
32.437
32.401
32.212
30.603
30.533
30.463
30.396
27.285
27.250
27.219
26.315
22.741
21.813
O
S
N
tBu
PCy2
O
Xu5-6
200 190 180 170 160 150 140 130 120 110 100 90 80 70 60 50 40 30 20 10 0

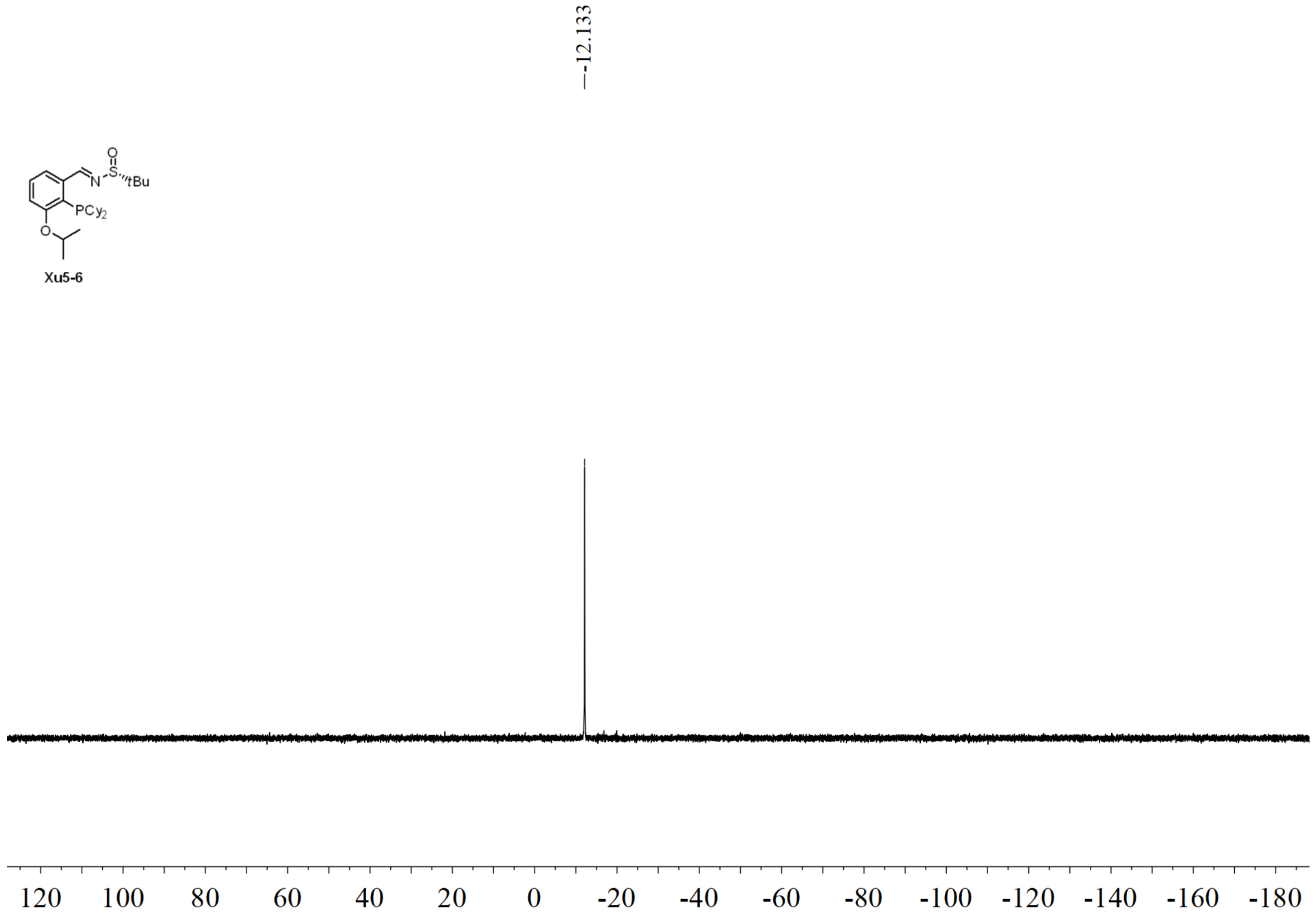
-12.133
O
S
N
tBu
PCy2
O
Xu5-6
120 100 80 60 40 20 0 -20 -40 -60 -80 -100 -120 -140 -160 -180

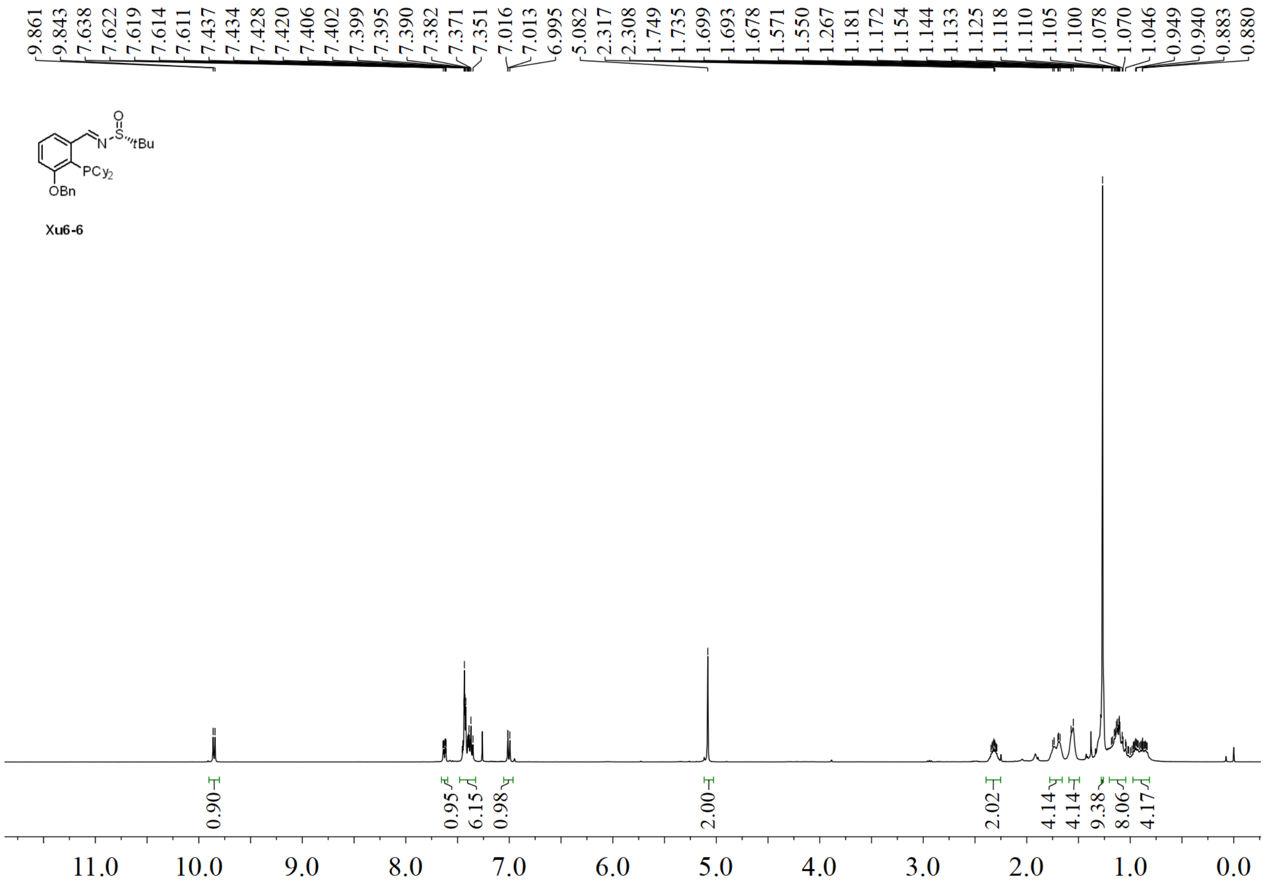
9.861 9.843 7.638 7.622 7.619 7.614 7.611 7.437 7.434 7.428 7.420 7.406 7.402 7.399 7.395 7.390 7.382 7.371 7.351 7.016 7.013 6.995 5.082 2.317 2.308 1.749 1.735 1.699 1.693 1.678 1.571 1.550 1.267 1.181 1.172 1.154 1.144 1.133 1.125 1.118 1.110 1.105 1.100 1.078 1.070 1.046 0.949 0.940 0.883 0.880
O
S
N
tBu
PCy2
OBn
Xu6-6
0.90 0.95 6.15 0.98 2.00 2.02 4.14 4.14 9.38 8.06 4.17
11.0 10.0 9.0 8.0 7.0 6.0 5.0 4.0 3.0 2.0 1.0 0.0

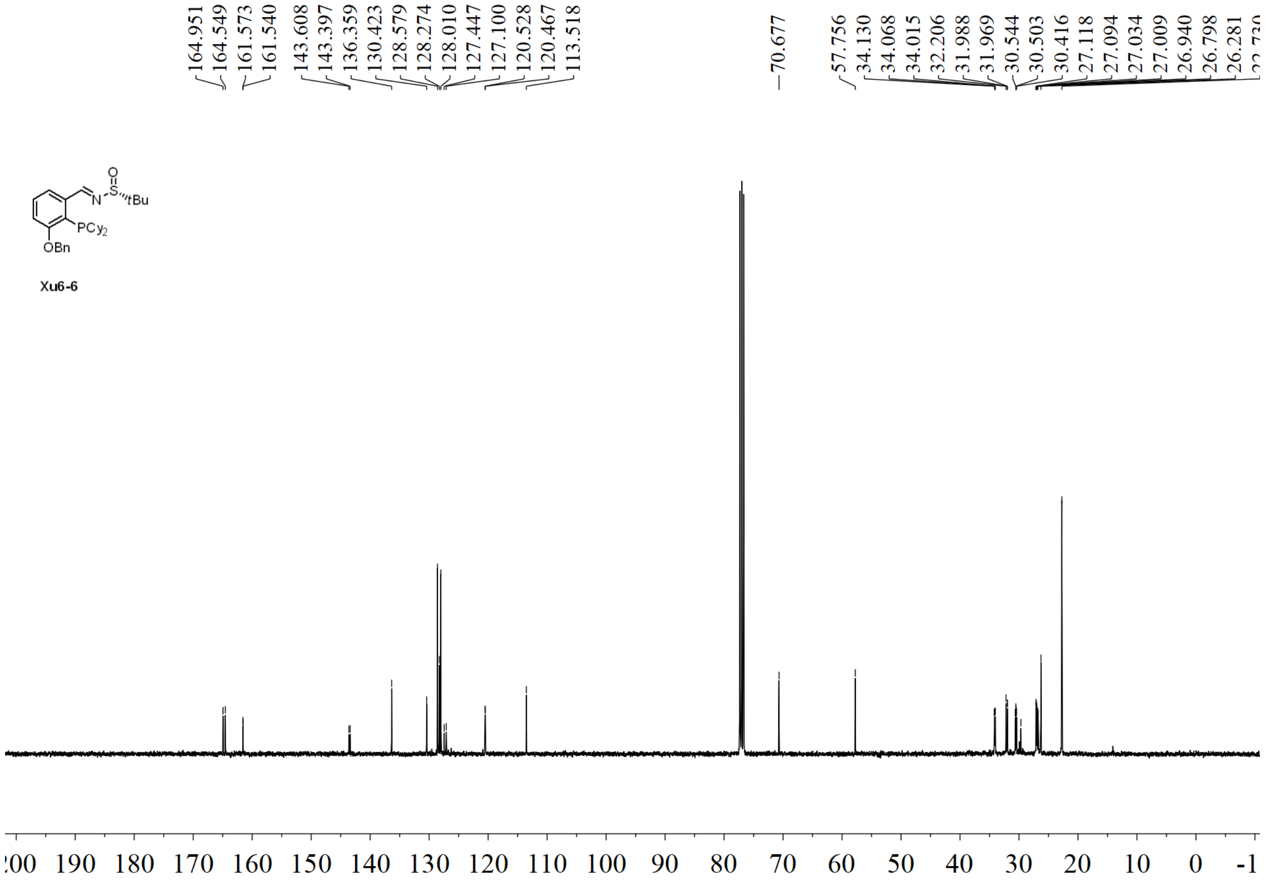
164.951 164.549 161.573 161.540 143.608 143.397 136.359 130.423 128.579 128.274 128.010 127.447 127.100 120.528 120.467 113.518
70.677
57.756 34.130 34.068 34.015 32.206 31.988 31.969 30.544 30.503 30.416 27.118 27.094 27.034 27.009 26.940 26.798 26.281
O
S
N
tBu
PCy2
OBn
Xu6-6
200 190 180 170 160 150 140 130 120 110 100 90 80 70 60 50 40 30 20 10 0 -1

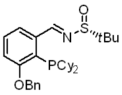

tBu
PCy2
OBn

Xu6-6

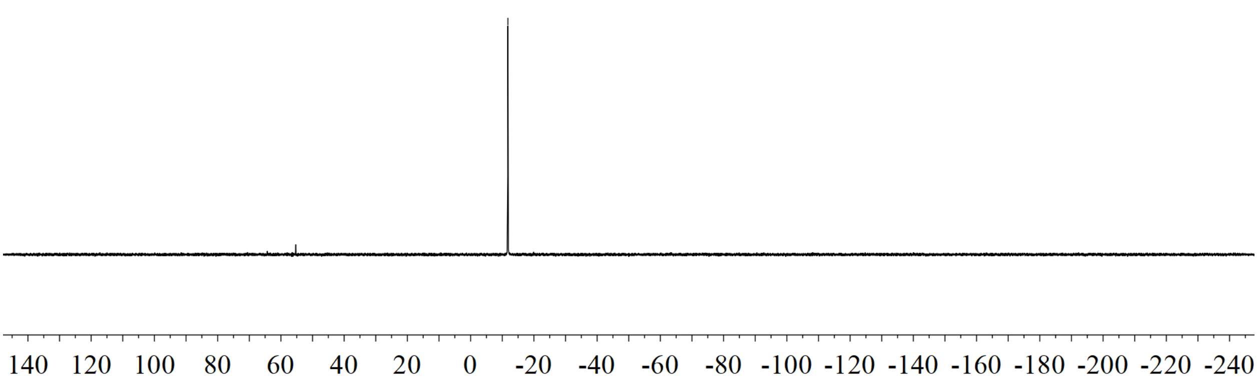

-11.789
140 120 100 80 60 40 20 0 -20 -40 -60 -80 -100 -120 -140 -160 -180 -200 -220 -240

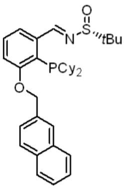

tBu
PCy2

Xu7-6

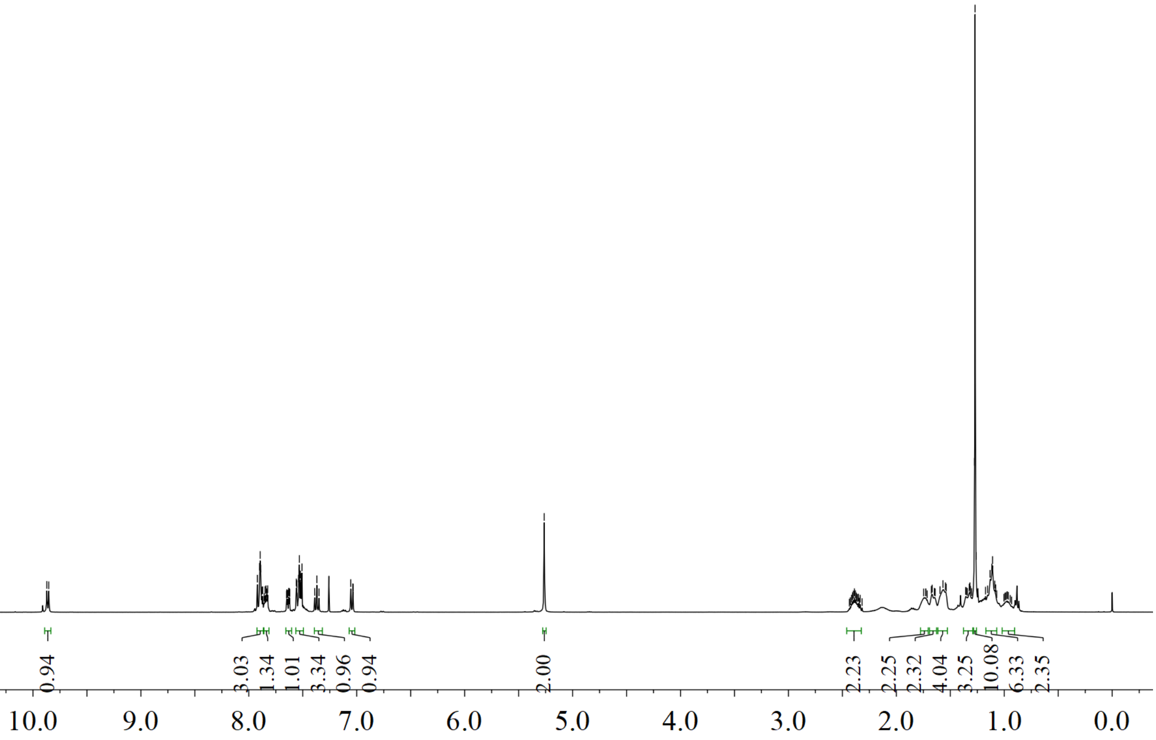

9.872 9.854 7.921 7.900 7.895 7.890 7.880 7.871 7.849 7.841 7.825 7.629 7.560 7.555 7.539 7.535 7.531 7.523 7.515 7.508 7.390 7.370 7.056 7.036 7.034 5.263 1.676 1.668 1.648 1.595 1.568 1.546 1.538 1.358 1.352 1.342 1.324 1.319 1.313 1.302 1.276 1.271 1.262 1.174 1.155 1.131 1.115 1.109 1.088 1.080
0.94 3.03 1.34 1.01 3.34 0.96 0.94 2.00 2.23 2.25 2.32 4.04 3.25 10.08 6.33 2.35
11.0 10.0 9.0 8.0 7.0 6.0 5.0 4.0 3.0 2.0 1.0 0.0

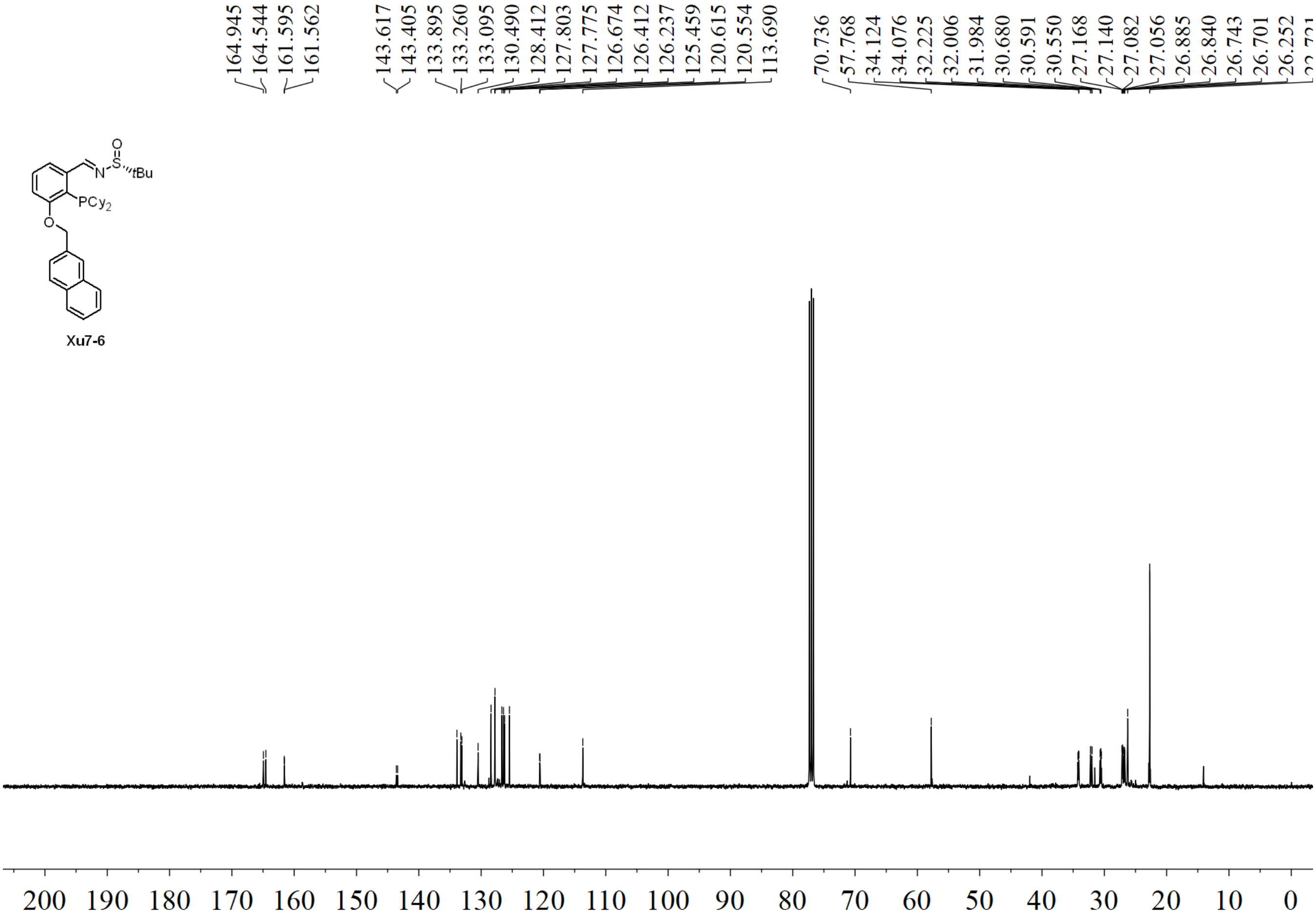
164.945
164.544
161.595
161.562
143.617
143.405
133.895
133.260
133.095
130.490
128.412
127.803
127.775
126.674
126.412
126.237
125.459
120.615
120.554
113.690
70.736
57.768
34.124
34.076
32.225
32.006
31.984
30.680
30.591
30.550
27.168
27.140
27.082
27.056
26.885
26.840
26.743
26.701
26.252
N
S
O
tBu
PCy2
O
Xu7-6
200 190 180 170 160 150 140 130 120 110 100 90 80 70 60 50 40 30 20 10 0

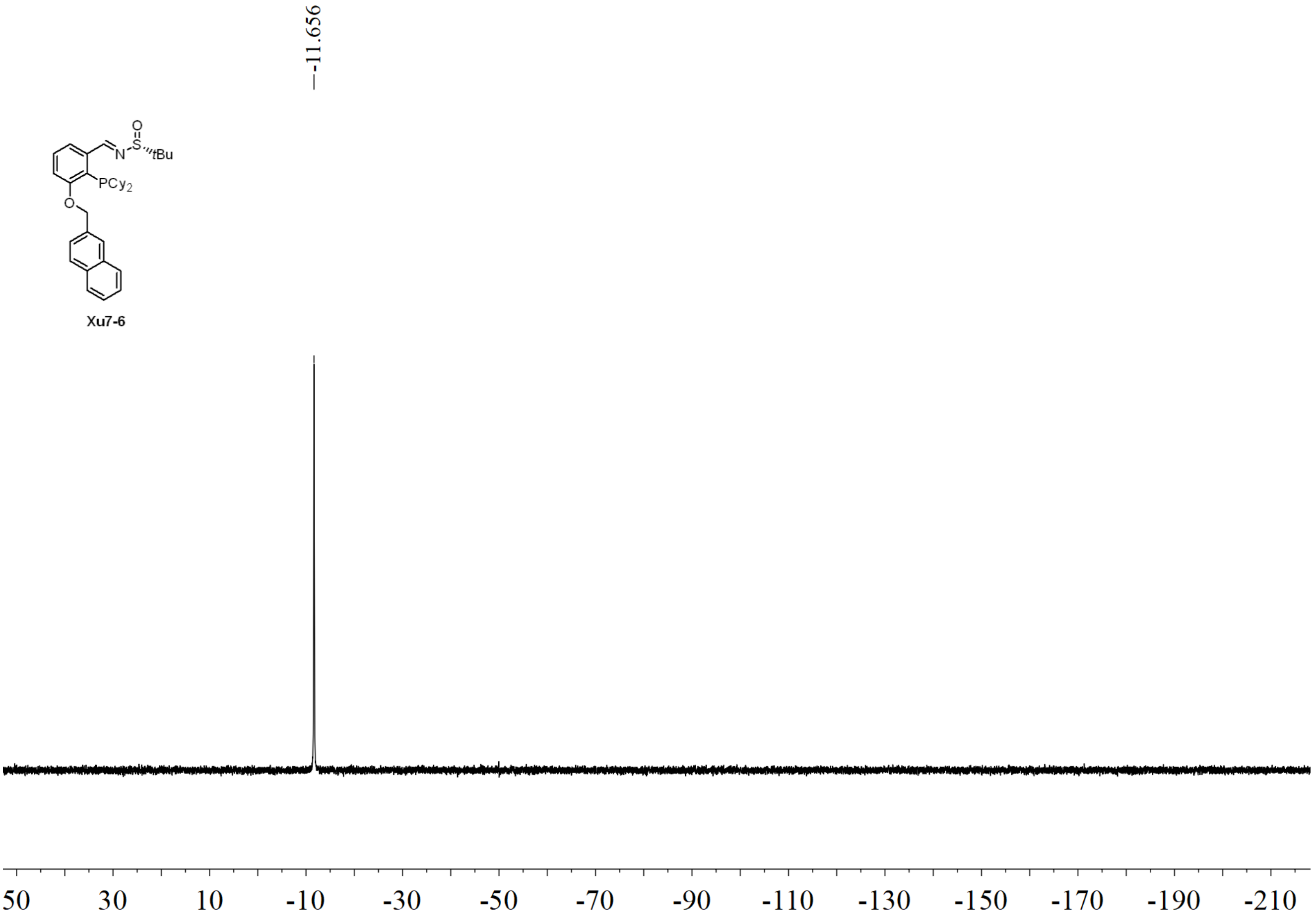
-11.656
N
S
O
tBu
PCy2
O
Xu7-6
50 30 10 -10 -30 -50 -70 -90 -110 -130 -150 -170 -190 -210

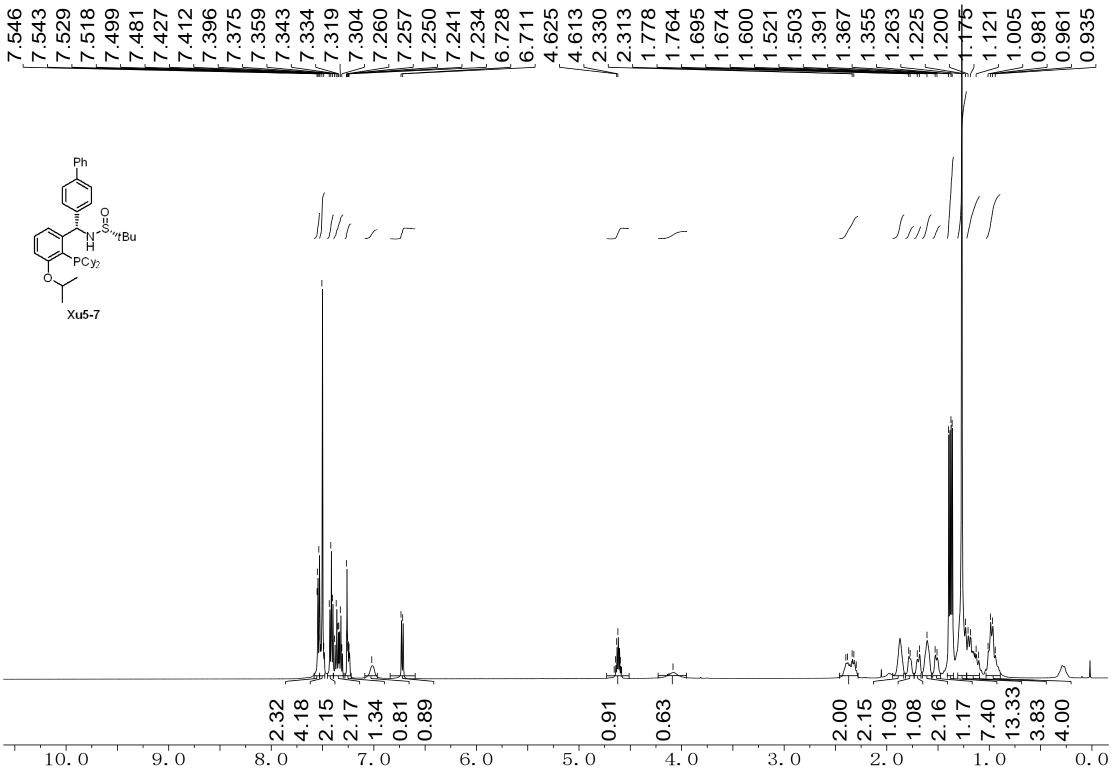
7.546 7.543 7.529 7.518 7.499 7.481 7.427 7.412 7.396 7.375 7.359 7.343 7.334 7.319 7.304 7.260 7.257 7.250 7.241 7.234 6.728 6.711 4.625 4.613 2.330 2.313 1.778 1.764 1.695 1.674 1.600 1.521 1.503 1.391 1.367 1.355 1.263 1.225 1.200 1.175 1.121 1.005 0.981 0.961 0.935
Ph
tBu
PCy2
Xu5-7
2.32 4.18 2.15 2.17 1.34 0.81 0.89 0.91 0.63 2.00 2.15 1.09 1.08 2.16 1.17 7.40 13.33 3.83 4.00
10.0 9.0 8.0 7.0 6.0 5.0 4.0 3.0 2.0 1.0 0.0

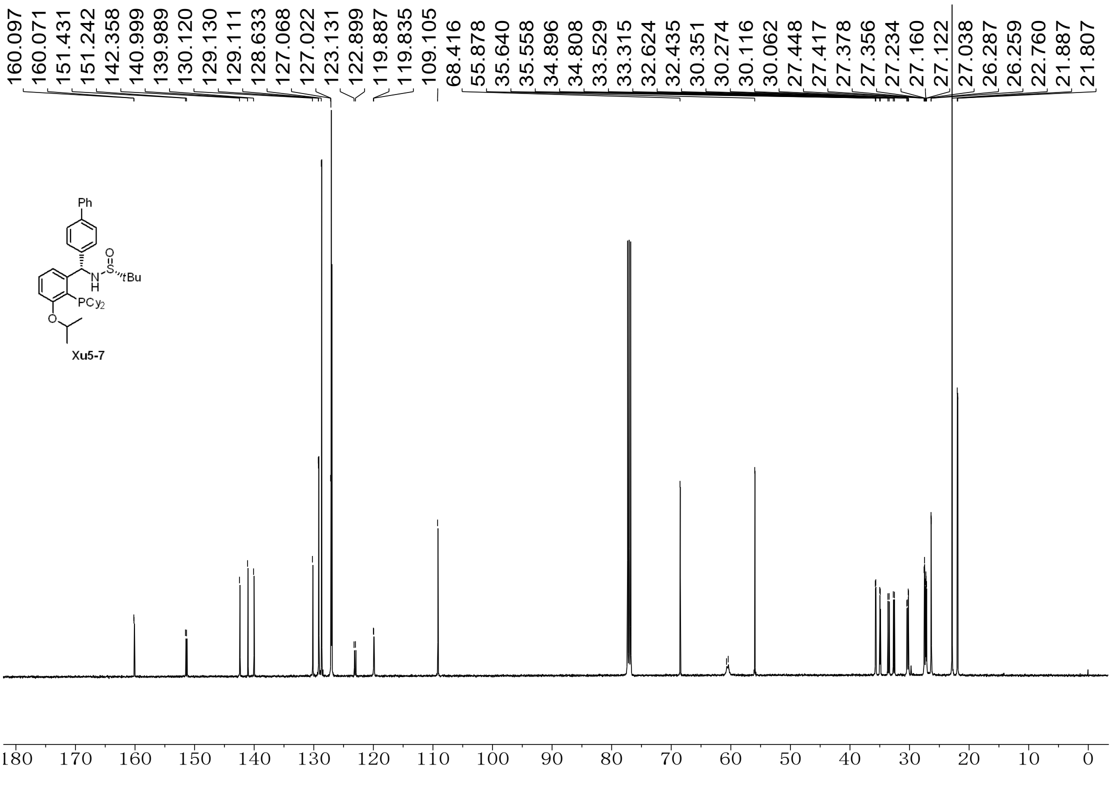
160.097 160.071 151.431 151.242 142.358 140.999 139.989 130.120 129.130 129.111 128.633 127.068 127.022 123.131 122.899 119.887 119.835 109.105 68.416 55.878 35.640 35.558 34.896 34.808 33.529 33.315 32.624 32.435 30.351 30.274 30.116 30.062 27.448 27.417 27.378 27.356 27.234 27.160 27.122 27.038 26.287 26.259 22.760 21.887 21.807
Ph
tBu
PCy2
Xu5-7
180 170 160 150 140 130 120 110 100 90 80 70 60 50 40 30 20 10 0

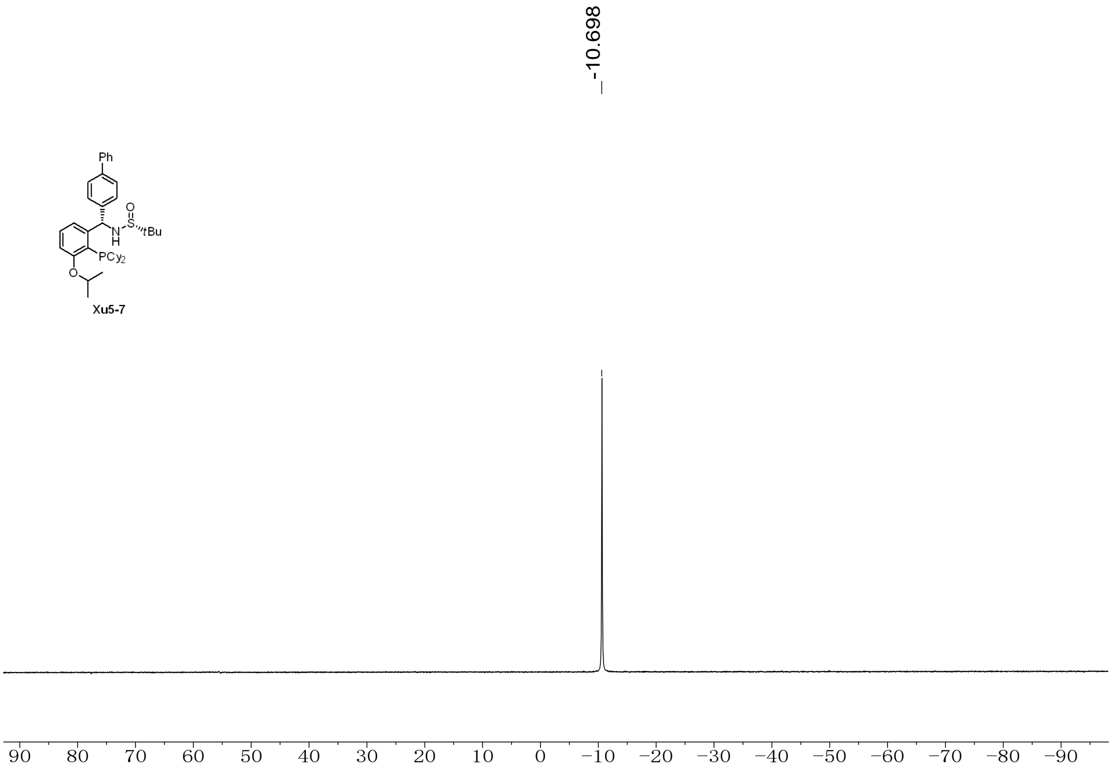

-10.698
Ph
O
S
N
H
tBu
PCy2
O
Xu5-7
90 80 70 60 50 40 30 20 10 0 −10 −20 −30 −40 −50 −60 −70 −80 −90

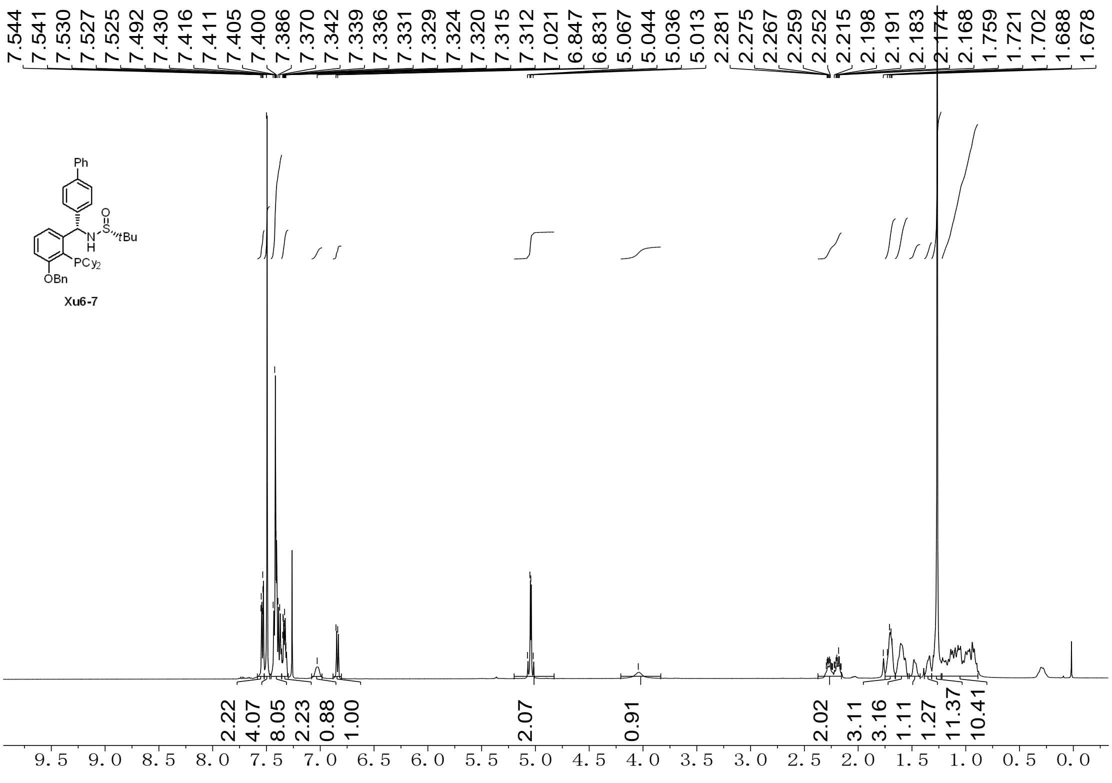

7.544 7.541 7.530 7.527 7.525 7.492 7.430 7.416 7.411 7.405 7.400 7.386 7.370 7.342 7.339 7.336 7.331 7.329 7.324 7.320 7.315 7.312 7.021 6.847 6.831 5.067 5.044 5.036 5.013 2.281 2.275 2.267 2.259 2.252 2.215 2.198 2.191 2.183 2.174 2.168 1.759 1.721 1.702 1.688 1.678
Ph
O
S
N
H
tBu
PCy2
OBn
Xu6-7
2.22 4.07 8.05 2.23 0.88 1.00 2.07 0.91 2.02 3.11 3.16 1.11 1.27 11.37 10.41
9.5 9.0 8.5 8.0 7.5 7.0 6.5 6.0 5.5 5.0 4.5 4.0 3.5 3.0 2.5 2.0 1.5 1.0 0.5 0.0

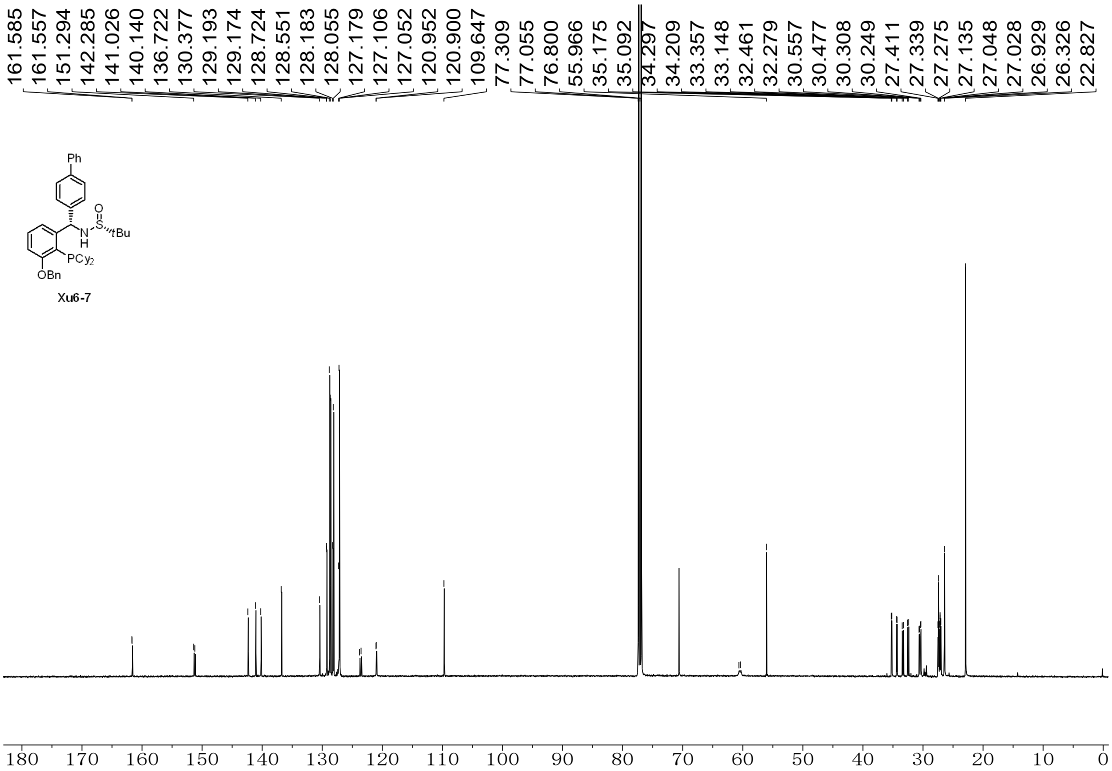
161.585
161.557
151.294
142.285
141.026
140.140
136.722
130.377
129.193
129.174
128.724
128.551
128.183
128.055
127.179
127.106
127.052
120.952
120.900
109.647
77.309
77.055
76.800
55.966
35.175
35.092
34.297
34.209
33.357
33.148
32.461
32.279
30.557
30.477
30.308
30.249
27.411
27.339
27.275
27.135
27.048
27.028
26.929
26.326
22.827
Ph
O
S
N
H
tBu
PCy2
OBn
Xu6-7
180 170 160 150 140 130 120 110 100 90 80 70 60 50 40 30 20 10 0

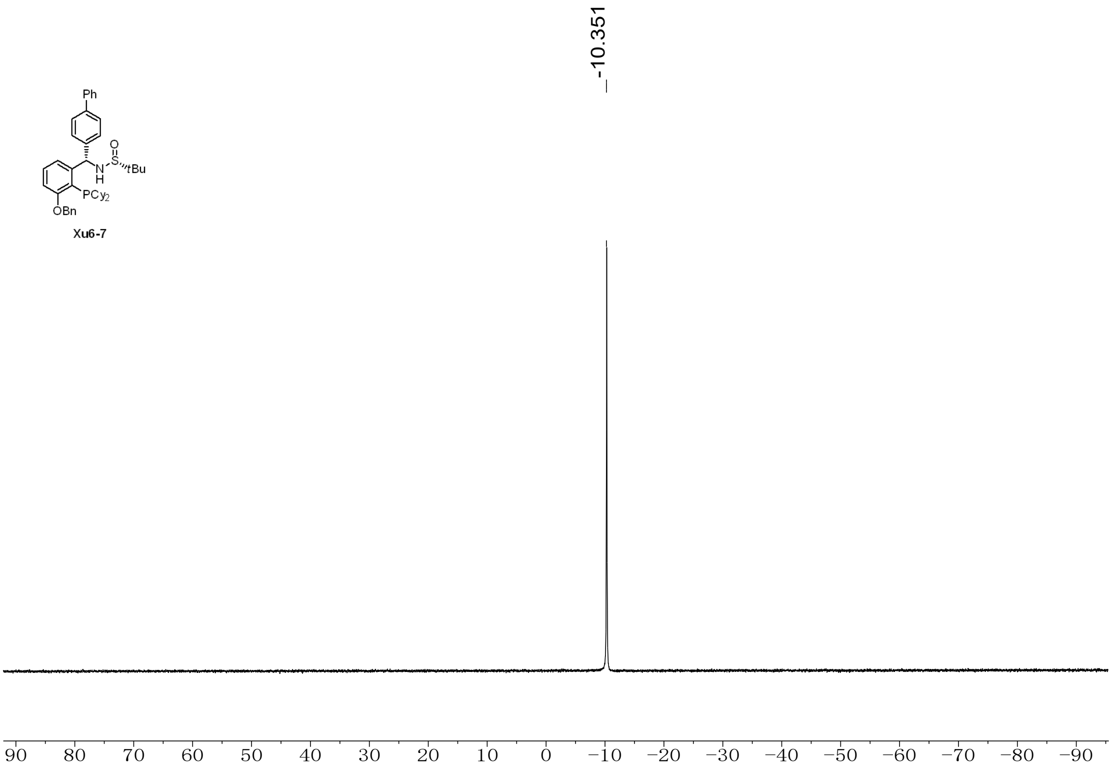
-10.351
Ph
O
S
N
H
tBu
PCy2
OBn
Xu6-7
90 80 70 60 50 40 30 20 10 0 −10 −20 −30 −40 −50 −60 −70 −80 −90

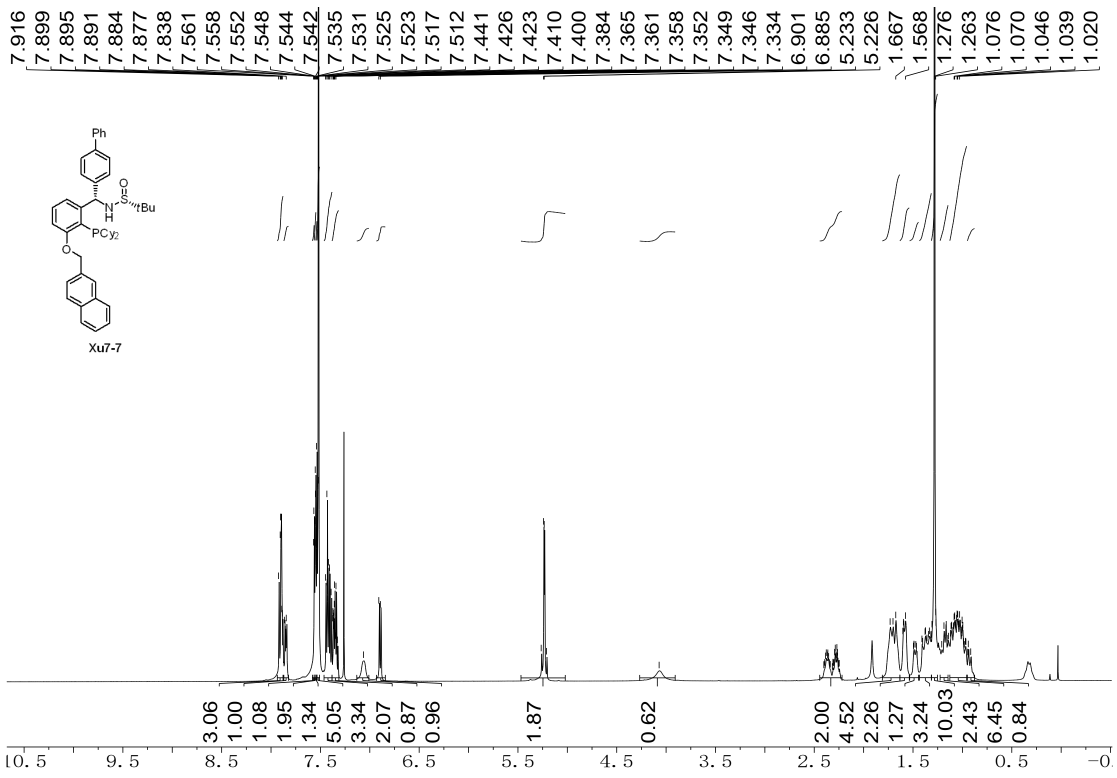
7.916
7.899
7.895
7.891
7.884
7.877
7.838
7.561
7.558
7.552
7.548
7.544
7.542
7.535
7.531
7.525
7.523
7.517
7.512
7.441
7.426
7.423
7.410
7.400
7.384
7.365
7.361
7.358
7.352
7.349
7.346
7.334
6.901
6.885
5.233
5.226
1.667
1.568
1.276
1.263
1.076
1.070
1.046
1.039
1.020
Ph
tBu
PCy2
Xu7-7
3.06
1.00
1.08
1.95
1.34
5.05
3.34
2.07
0.87
0.96
1.87
0.62
2.00
4.52
2.26
1.27
3.24
10.03
2.43
6.45
0.84

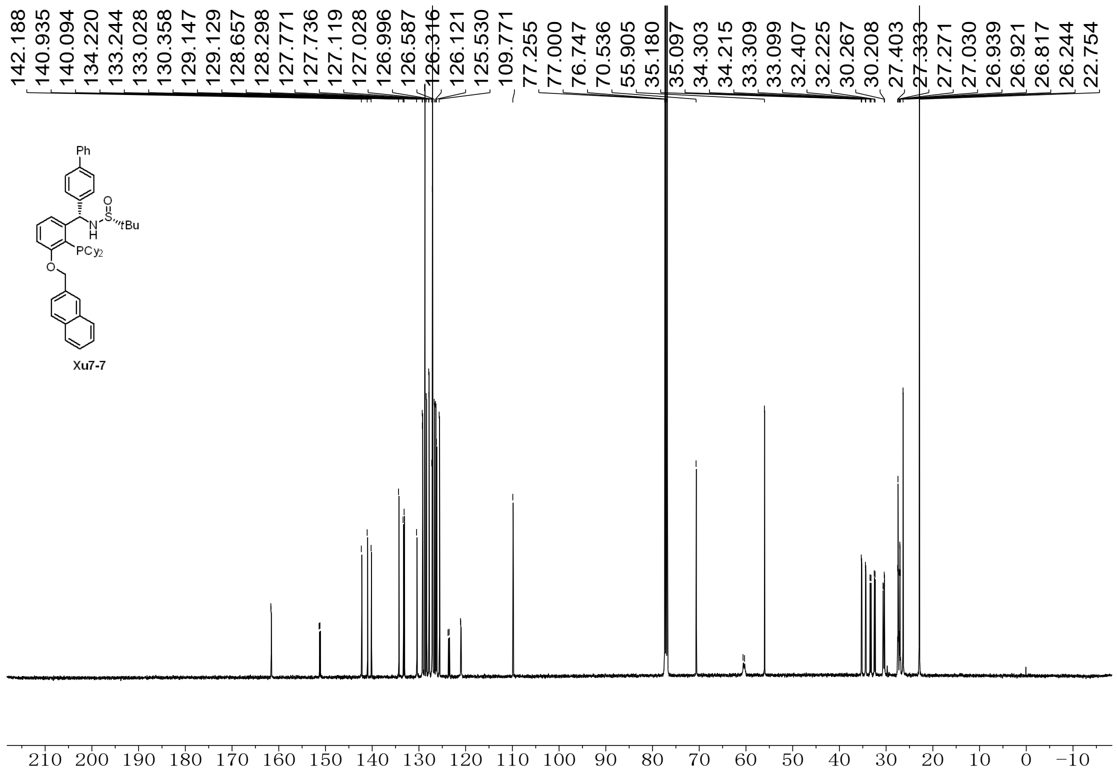
142.188
140.935
140.094
134.220
133.244
133.028
130.358
129.147
129.129
128.657
128.298
127.771
127.736
127.119
127.028
126.996
126.587
126.316
126.121
125.530
109.771
77.255
77.000
76.747
70.536
55.905
35.180
35.097
34.303
34.215
33.309
33.099
32.407
32.225
30.267
30.208
27.403
27.333
27.271
27.030
26.939
26.921
26.817
26.244
22.754
Ph
tBu
PCy2
Xu7-7

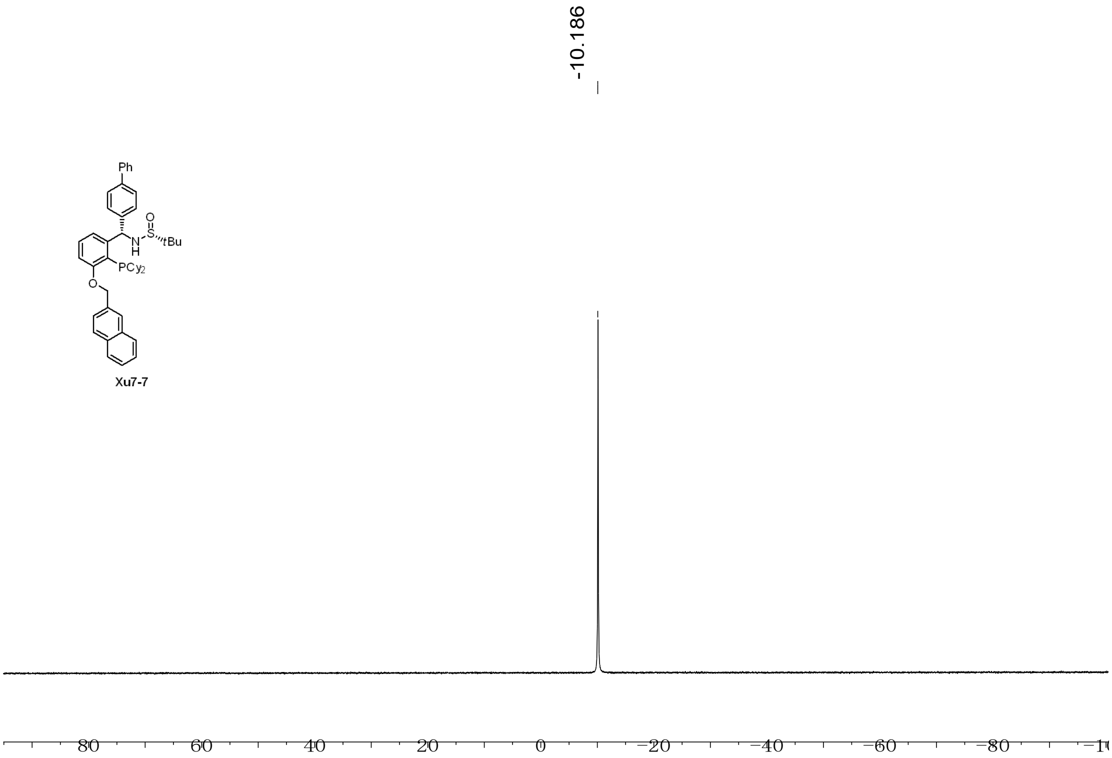
-10.186
Ph
O
S
N
H
tBu
PCy2
O
Xu7-7
80
60
40
20
0
-20
-40
-60
-80

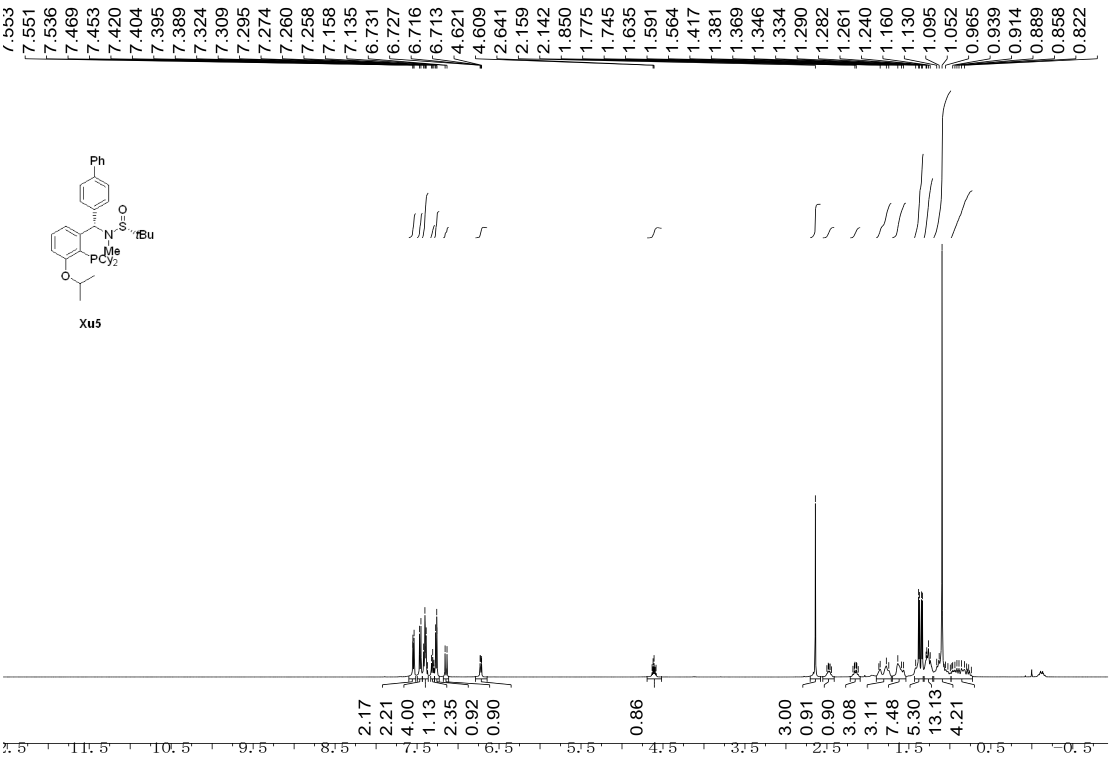
7.551
7.536
7.469
7.453
7.420
7.404
7.395
7.389
7.324
7.309
7.295
7.274
7.260
7.258
7.158
7.135
6.731
6.727
6.716
6.713
4.621
4.609
2.641
2.159
2.142
1.850
1.775
1.745
1.635
1.591
1.564
1.417
1.381
1.369
1.346
1.334
1.290
1.282
1.261
1.240
1.160
1.130
1.095
1.052
0.965
0.939
0.914
0.889
0.858
0.822
Ph
O
S
N
Me
tBu
PCy2
O
Xu5
2.17
2.21
4.00
1.13
2.35
0.92
0.90
0.86
3.00
0.91
0.90
3.08
3.11
7.48
5.30
13.13
4.21
11.5
10.5
9.5
8.5
7.5
6.5
5.5
4.5
3.5
2.5
1.5
0.5
-0.5

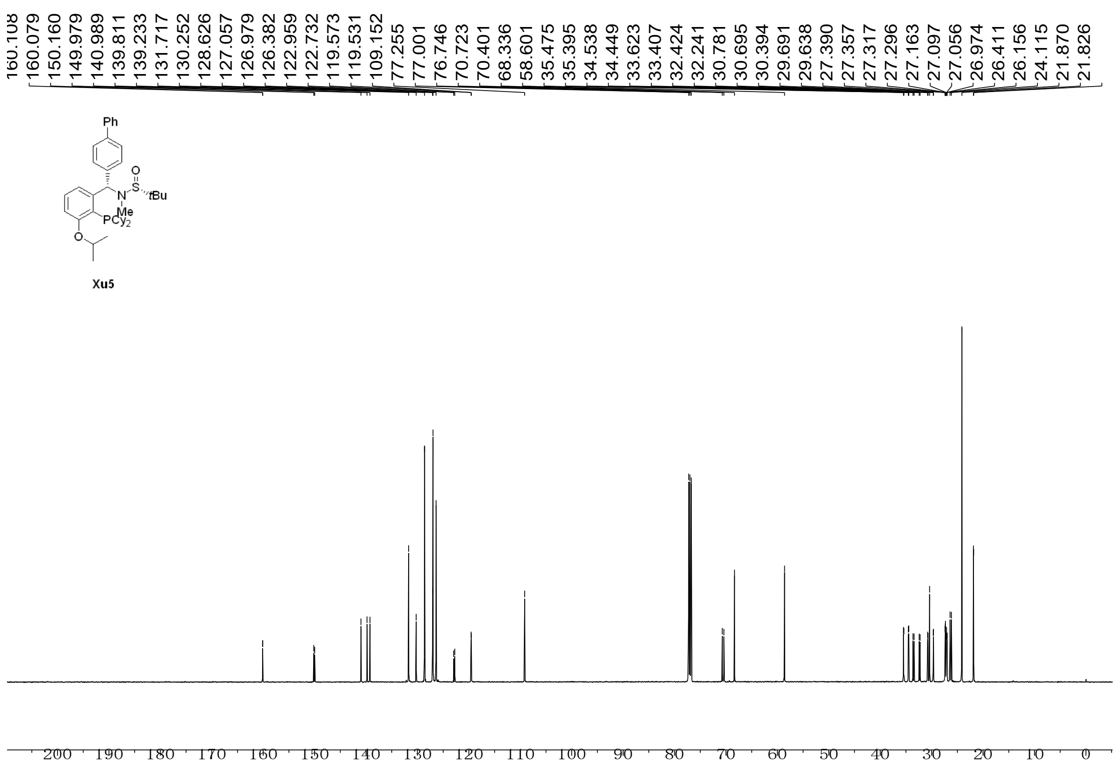
160.108
160.079
150.160
149.979
140.989
139.811
139.233
131.717
130.252
128.626
127.057
126.979
126.382
122.959
122.732
119.573
119.531
109.152
77.255
77.001
76.746
70.723
70.401
68.336
58.601
35.475
35.395
34.538
34.449
33.623
33.407
32.424
32.241
30.781
30.695
30.394
29.691
29.638
27.390
27.357
27.317
27.296
27.163
27.097
27.056
26.974
26.411
26.156
24.115
21.870
21.826
Ph
O
S
N
tBu
Me
PCy2
O
Xu5
200 190 180 170 160 150 140 130 120 110 100 90 80 70 60 50 40 30 20 10 0

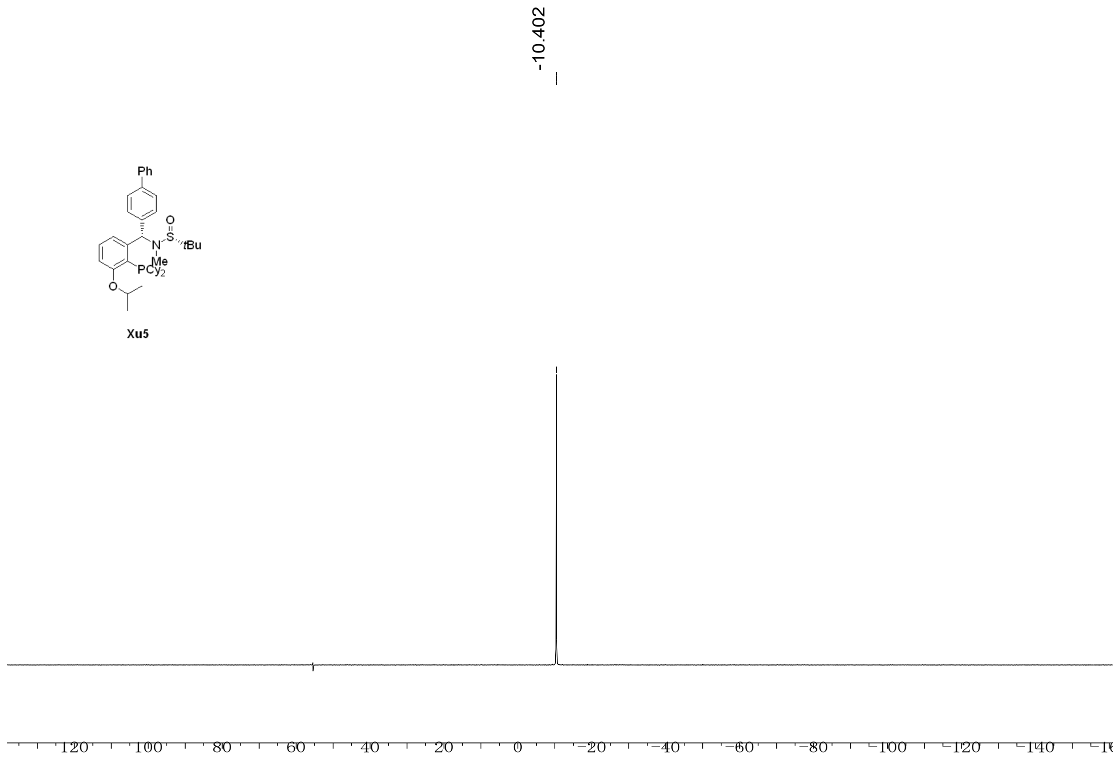
-10.402
Ph
O
S
N
tBu
Me
PCy2
O
Xu5
120 100 80 60 40 20 0 −20 −40 −60 −80 −100 −120 −140

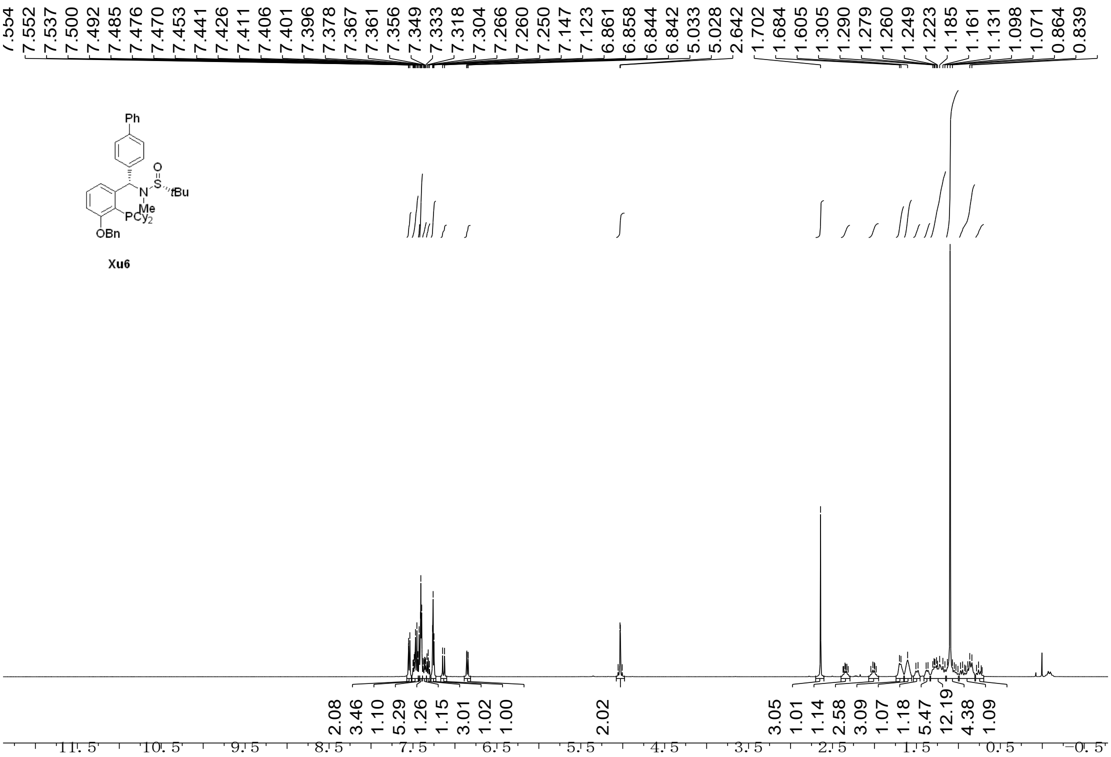
7.554
7.552
7.537
7.500
7.492
7.485
7.476
7.470
7.453
7.441
7.426
7.411
7.406
7.401
7.396
7.378
7.367
7.361
7.356
7.349
7.333
7.318
7.304
7.266
7.260
7.250
7.147
7.123
6.861
6.858
6.844
6.842
5.033
5.028
2.642
1.702
1.684
1.605
1.305
1.290
1.279
1.260
1.249
1.223
1.185
1.161
1.131
1.098
1.071
0.864
0.839
Ph
O
N
S
tBu
Me
PCy2
OBn
Xu6
2.08
3.46
1.10
5.29
1.26
1.15
3.01
1.02
1.00
2.02
3.05
1.01
1.14
2.58
3.09
1.07
1.18
5.47
12.19
4.38
1.09
11.5
10.5
9.5
8.5
7.5
6.5
5.5
4.5
3.5
2.5
1.5
0.5
-0.5

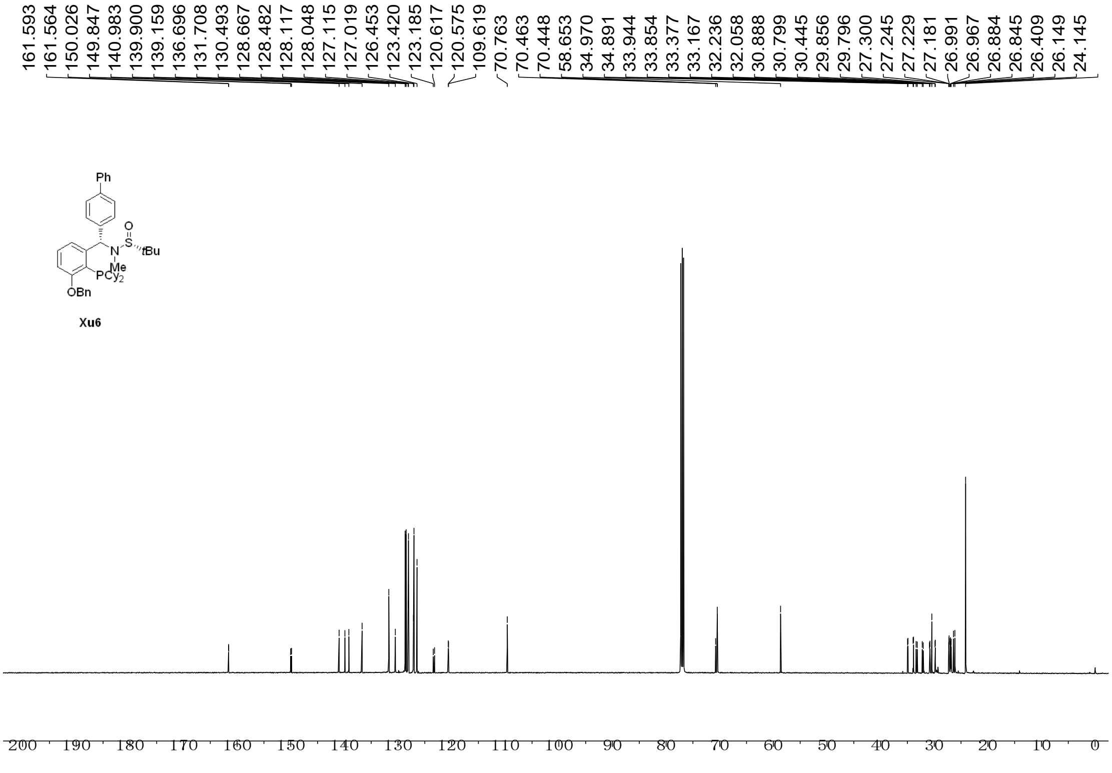
161.593
161.564
150.026
149.847
140.983
139.900
139.159
136.696
131.708
130.493
128.667
128.482
128.117
128.048
127.115
127.019
126.453
123.420
123.185
120.617
120.575
109.619
70.763
70.463
70.448
58.653
34.970
34.891
33.944
33.854
33.377
33.167
32.236
32.058
30.888
30.799
30.445
29.856
29.796
27.300
27.245
27.229
27.181
26.991
26.967
26.884
26.845
26.409
26.149
24.145
Ph
O
N
S
tBu
Me
PCy2
OBn
Xu6
200
190
180
170
160
150
140
130
120
110
100
90
80
70
60
50
40
30
20
10
0

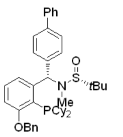
Ph
O
S
N
tBu
Me
PCy2
OBn

Xu6

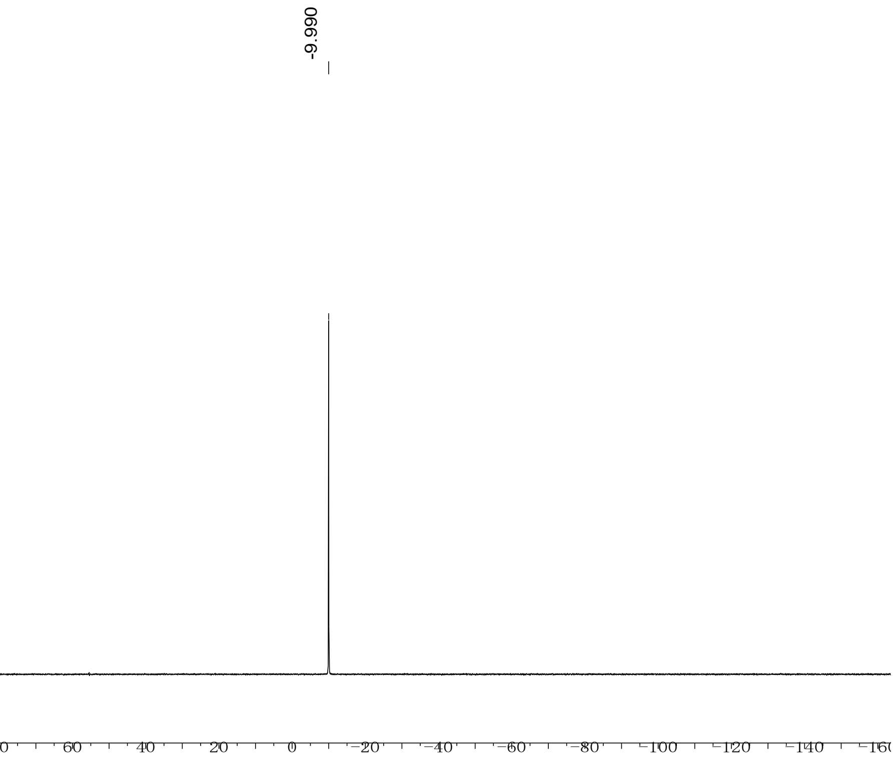
-9.990
120 100 80 60 40 20 0 −20 −40 −60 −80 −100 −120 −140

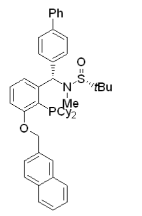
Ph
O
S
N
tBu
Me
PCy2
O

Xu7

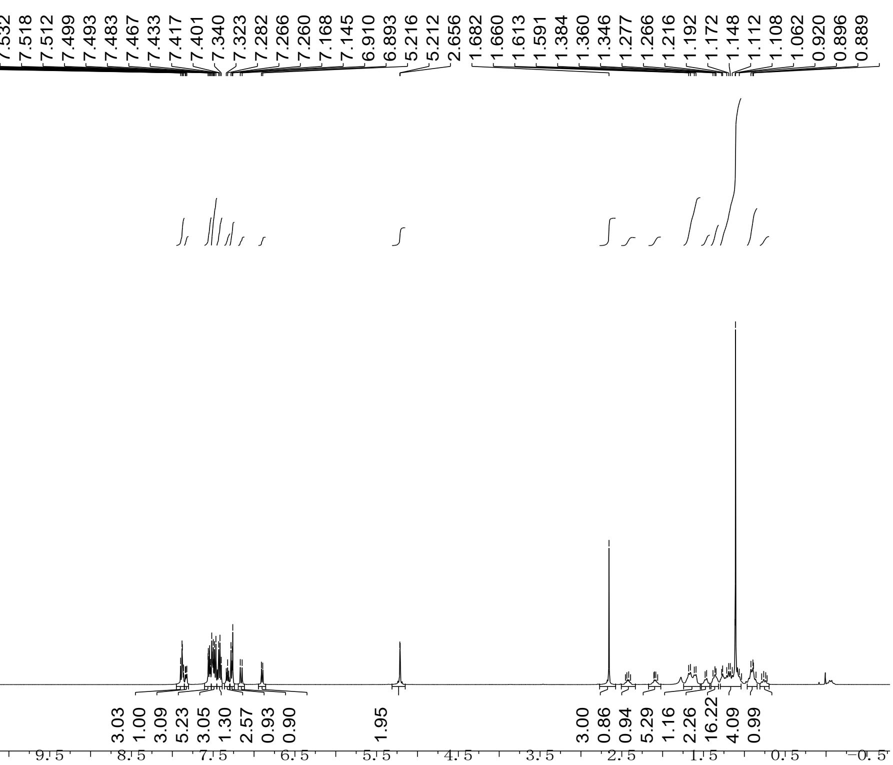
7.881 7.877 7.863 7.842 7.835 7.823 7.562 7.560 7.544 7.532 7.518 7.512 7.499 7.493 7.483 7.467 7.433 7.417 7.401 7.340 7.323 7.282 7.266 7.260 7.168 7.145 6.910 6.893 5.216 5.212 2.656 1.682 1.660 1.613 1.591 1.384 1.360 1.346 1.277 1.266 1.216 1.192 1.172 1.148 1.112 1.108 1.062 0.920 0.896 0.889
3.03 1.00 3.09 5.23 3.05 1.30 2.57 0.93 0.90
1.95
3.00 0.86 0.94 5.29 1.16 2.26 16.22 4.09 0.99
12.5 11.5 10.5 9.5 8.5 7.5 6.5 5.5 4.5 3.5 2.5 1.5 0.5 −0.5

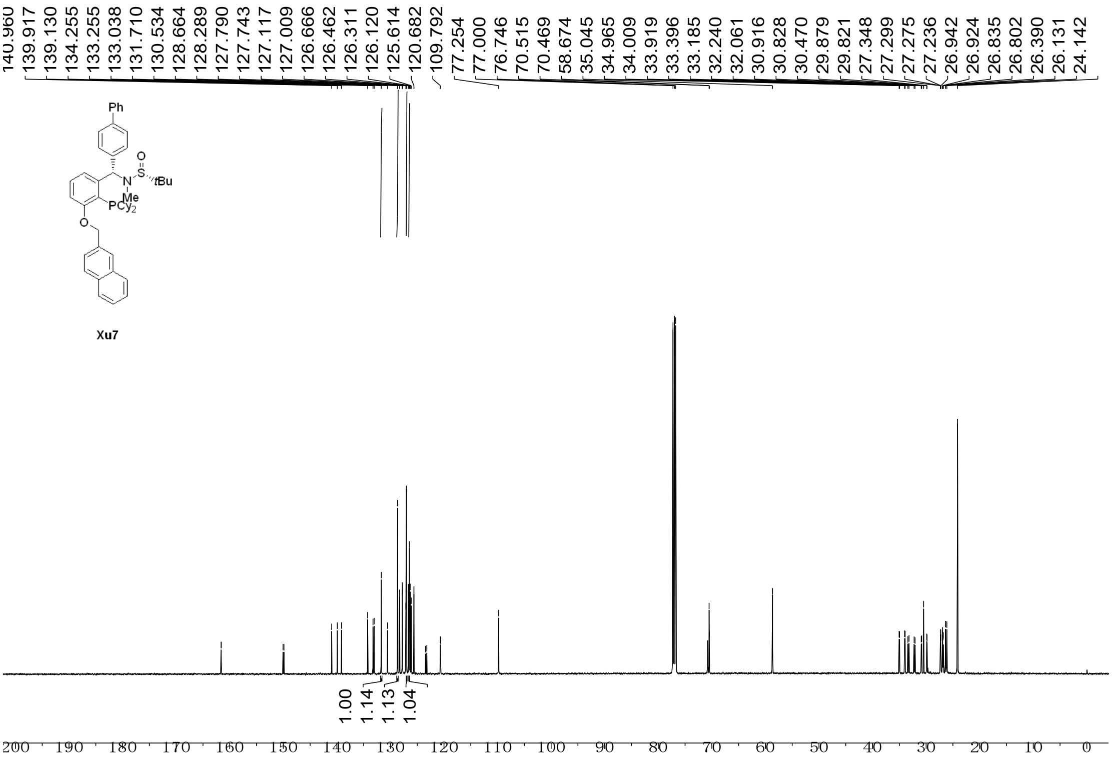
140.960
139.917
139.130
134.255
133.255
133.038
131.710
130.534
128.664
128.289
127.790
127.743
127.117
127.009
126.666
126.462
126.311
126.120
125.614
120.682
109.792
77.254
77.000
76.746
70.515
70.469
58.674
35.045
34.965
34.009
33.919
33.396
33.185
32.240
32.061
30.916
30.828
30.470
29.879
29.821
27.348
27.299
27.275
27.236
26.942
26.924
26.835
26.802
26.390
26.131
24.142
Ph
O
S
N
tBu
Me
PCy2
Xu7
1.00
1.14
1.13
1.04
200 190 180 170 160 150 140 130 120 110 100 90 80 70 60 50 40 30 20 10 0

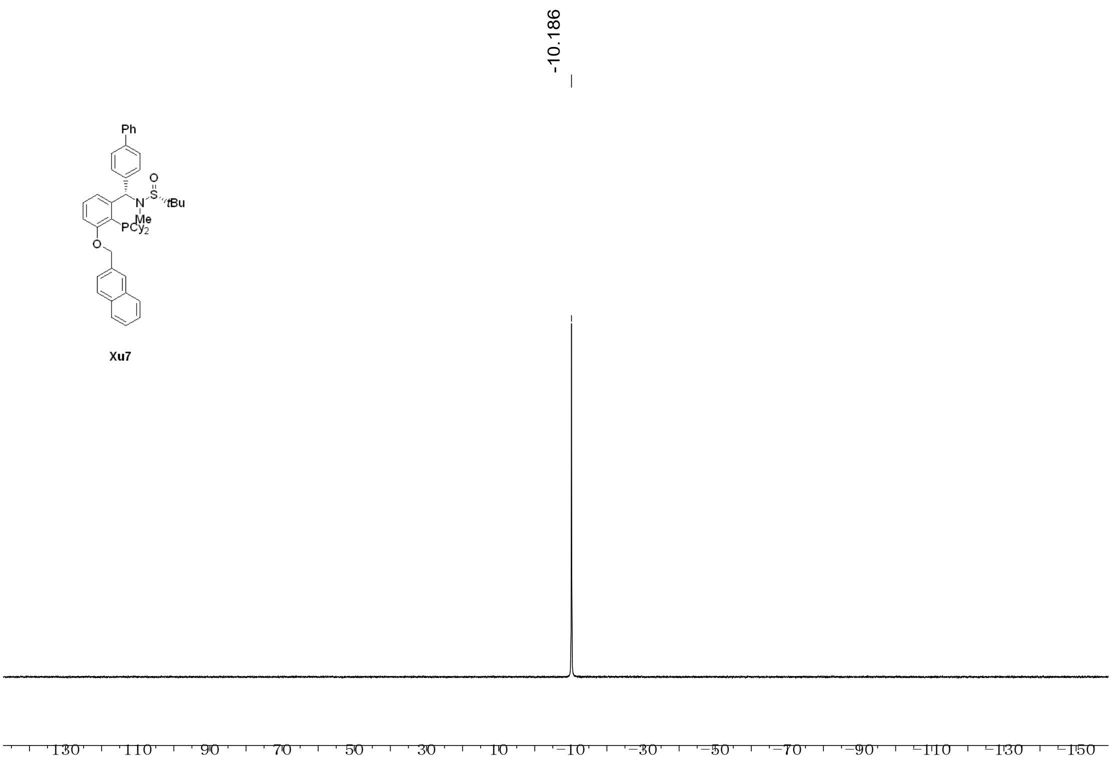
-10.186
Ph
O
S
N
tBu
Me
PCy2
Xu7
130 110 90 70 50 30 10 -10 -30 -50 -70 -90 -110 -130 -150

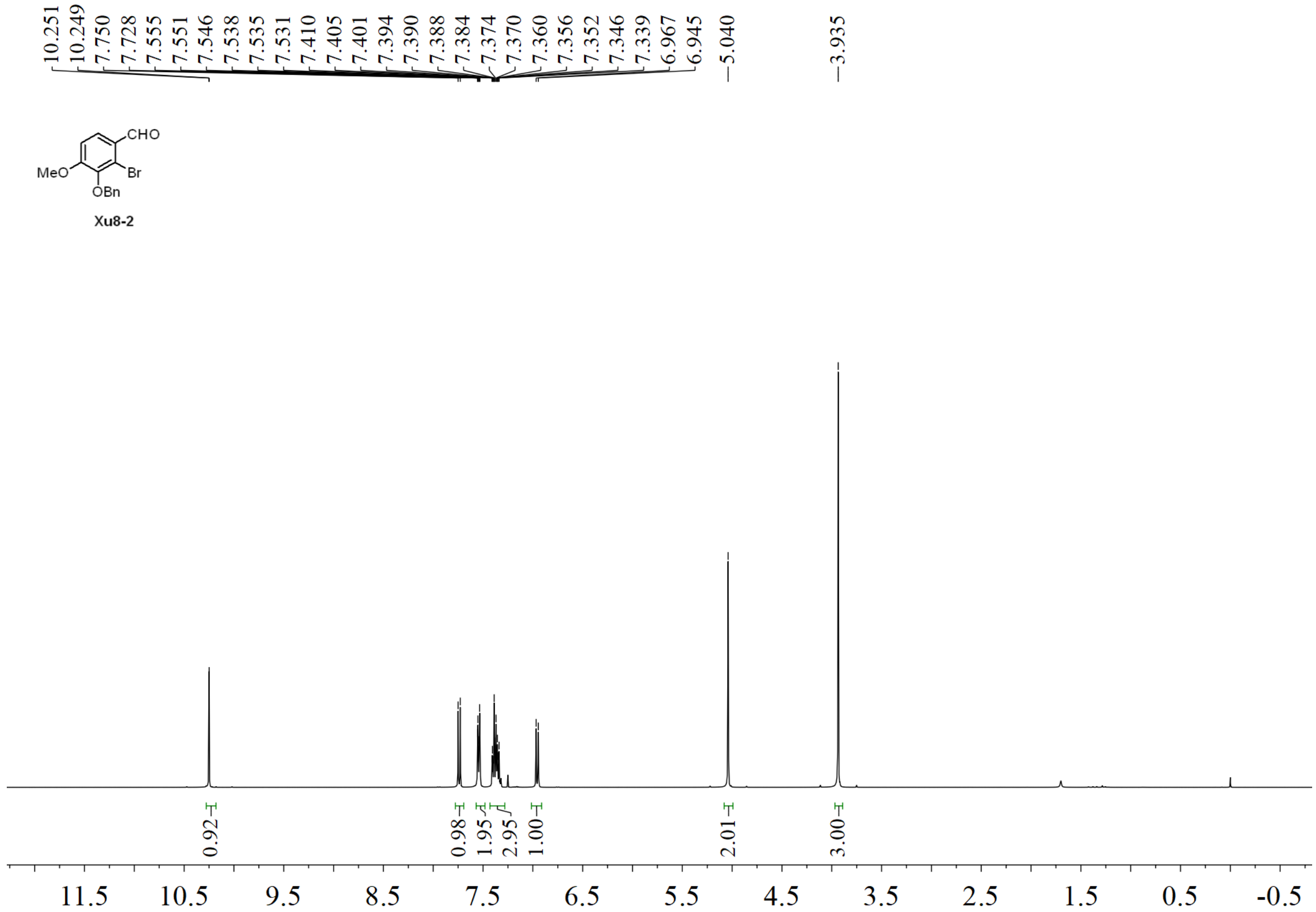
10.251
10.249
7.750
7.728
7.555
7.551
7.546
7.538
7.535
7.531
7.410
7.405
7.401
7.394
7.390
7.388
7.384
7.374
7.370
7.360
7.356
7.352
7.346
7.339
6.967
6.945
5.040
3.935
CHO
MeO
Br
OBn
Xu8-2
0.92
0.98
1.95
2.95
1.00
2.01
3.00
11.5
10.5
9.5
8.5
7.5
6.5
5.5
4.5
3.5
2.5
1.5
0.5
-0.5

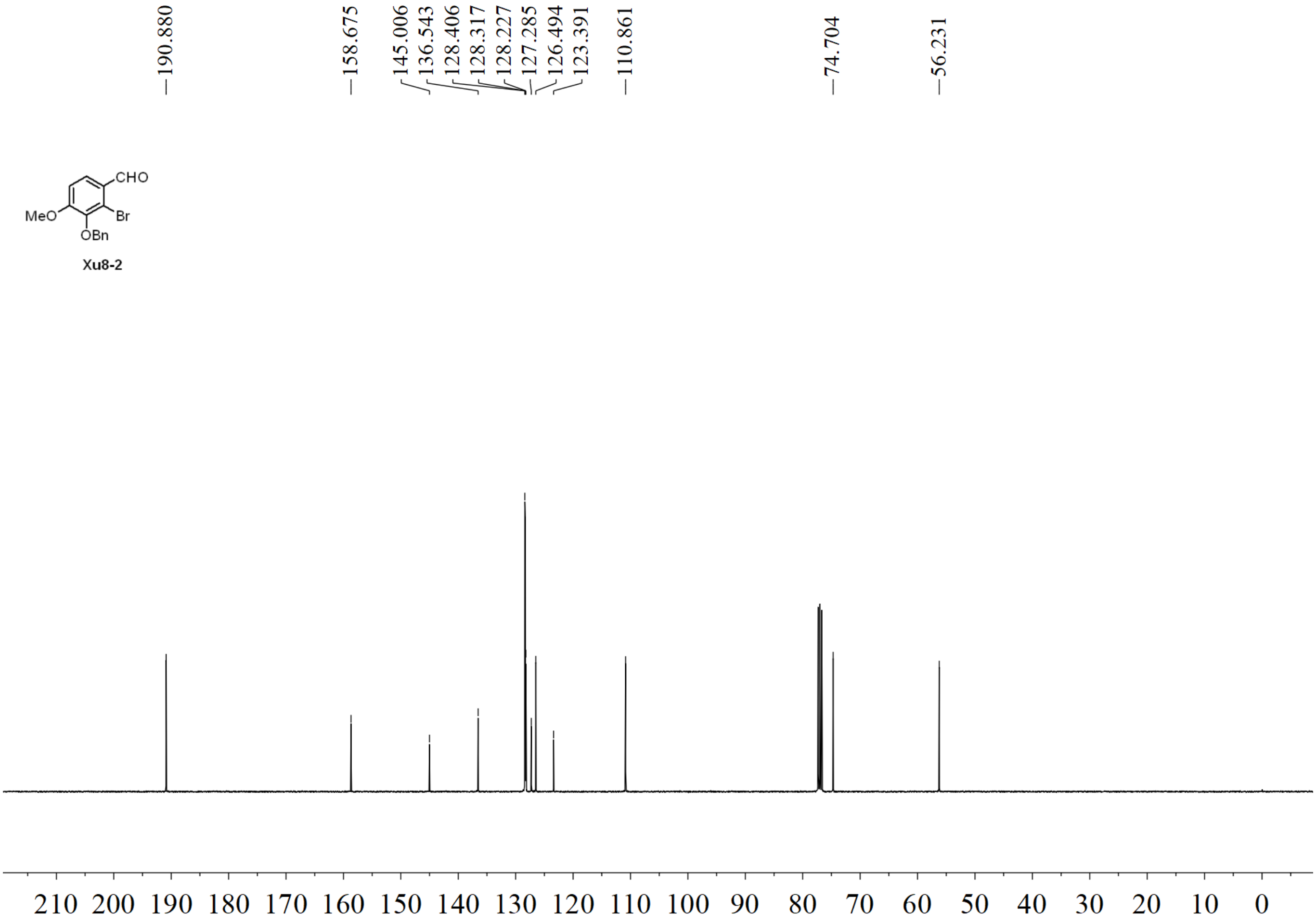
190.880
158.675
145.006
136.543
128.406
128.317
128.227
127.285
126.494
123.391
110.861
74.704
56.231
CHO
MeO
Br
OBn
Xu8-2
210 200 190 180 170 160 150 140 130 120 110 100 90 80 70 60 50 40 30 20 10 0

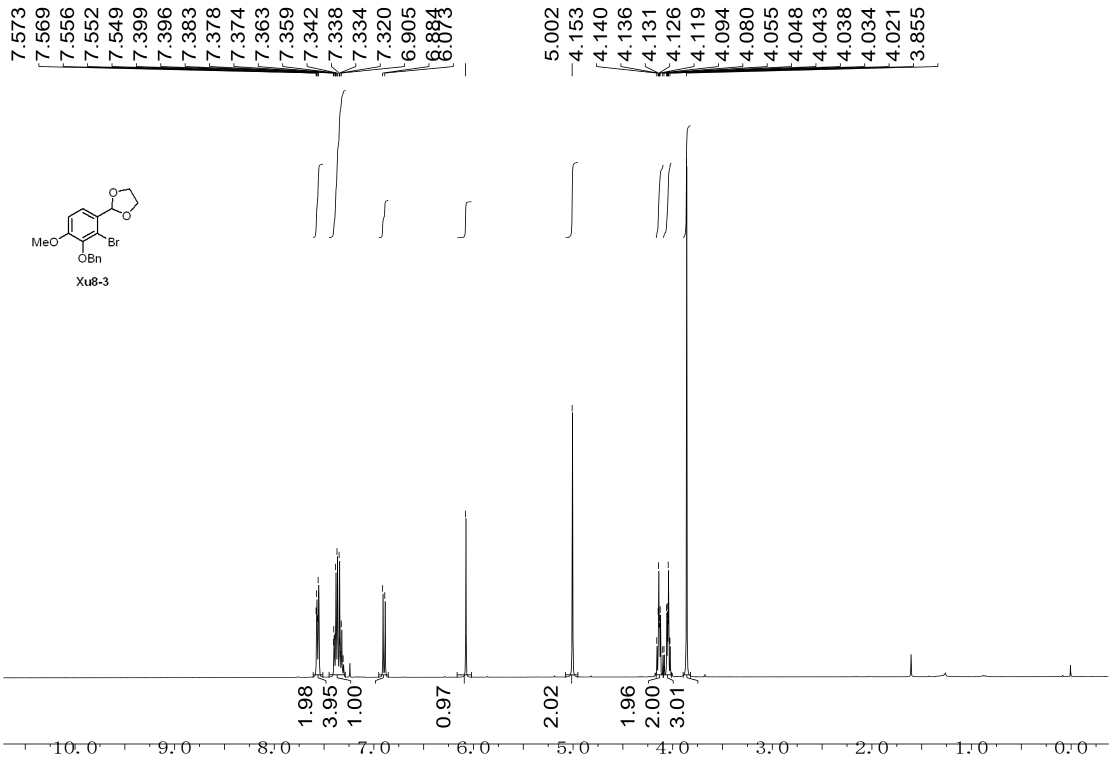
7.573
7.569
7.556
7.552
7.549
7.399
7.396
7.383
7.378
7.374
7.363
7.359
7.342
7.338
7.334
7.320
6.905
5.002
4.153
4.140
4.136
4.131
4.126
4.119
4.094
4.080
4.055
4.048
4.043
4.038
4.034
4.021
3.855
MeO
Br
OBn
Xu8-3
1.98
3.95
1.00
0.97
2.02
1.96
2.00
3.01
10.0 9.0 8.0 7.0 6.0 5.0 4.0 3.0 2.0 1.0 0.0

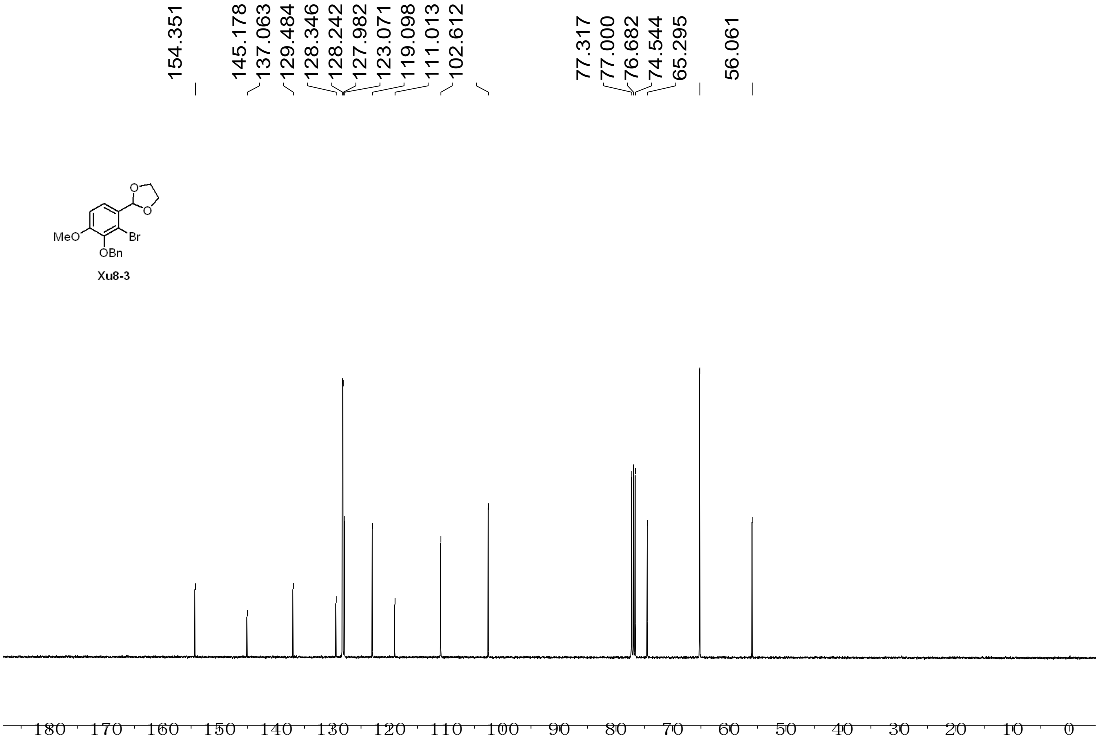
154.351
145.178
137.063
129.484
128.346
128.242
127.982
123.071
119.098
111.013
102.612
77.317
77.000
76.682
74.544
65.295
56.061
MeO
Br
OBn
Xu8-3
180 170 160 150 140 130 120 110 100 90 80 70 60 50 40 30 20 10 0

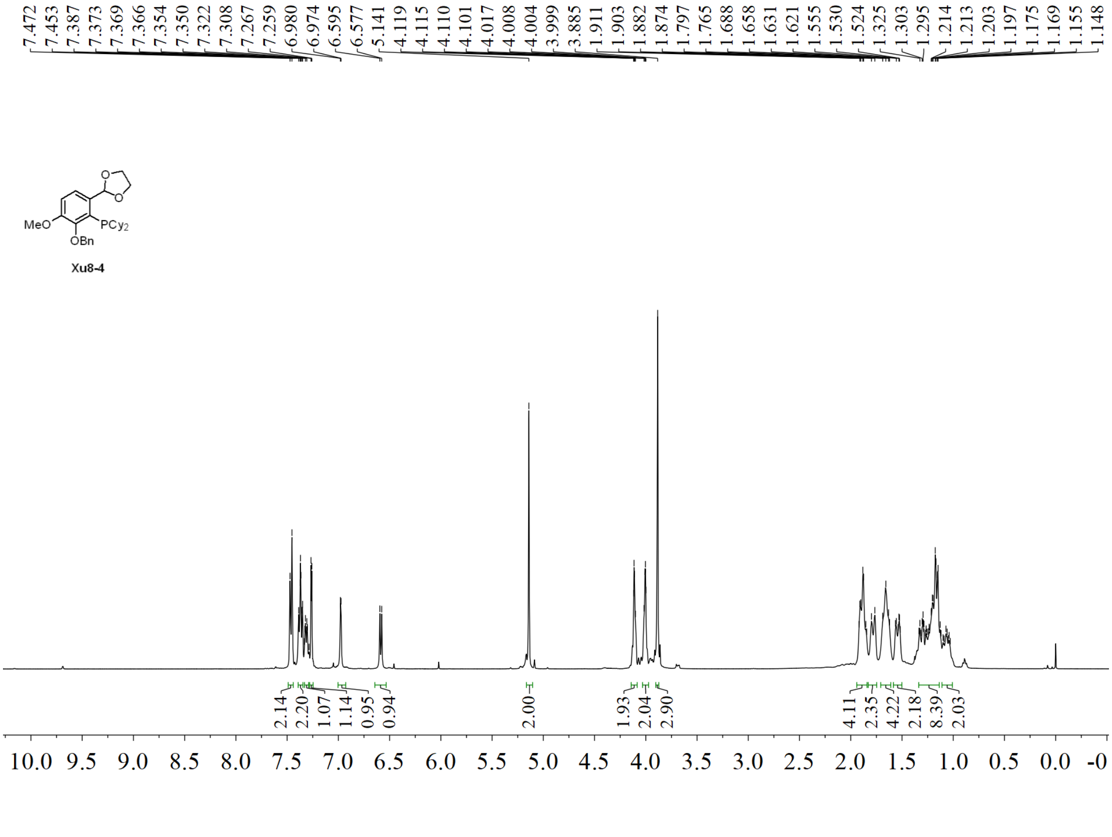
7.472
7.453
7.387
7.373
7.369
7.366
7.354
7.350
7.322
7.308
7.267
7.259
6.980
6.974
6.595
6.577
5.141
4.119
4.115
4.110
4.101
4.017
4.008
4.004
3.999
3.885
1.911
1.903
1.882
1.874
1.797
1.765
1.688
1.658
1.631
1.621
1.555
1.530
1.524
1.325
1.303
1.295
1.214
1.213
1.203
1.197
1.175
1.169
1.155
1.148
MeO
PCy2
OBn
Xu8-4
2.14
2.20
1.07
1.14
0.95
0.94
2.00
1.93
2.04
2.90
4.11
2.35
4.22
2.18
8.39
2.03
10.0 9.5 9.0 8.5 8.0 7.5 7.0 6.5 6.0 5.5 5.0 4.5 4.0 3.5 3.0 2.5 2.0 1.5 1.0 0.5 0.0 -0

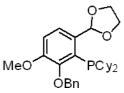

MeO
PCy2
OBn

Xu8-4

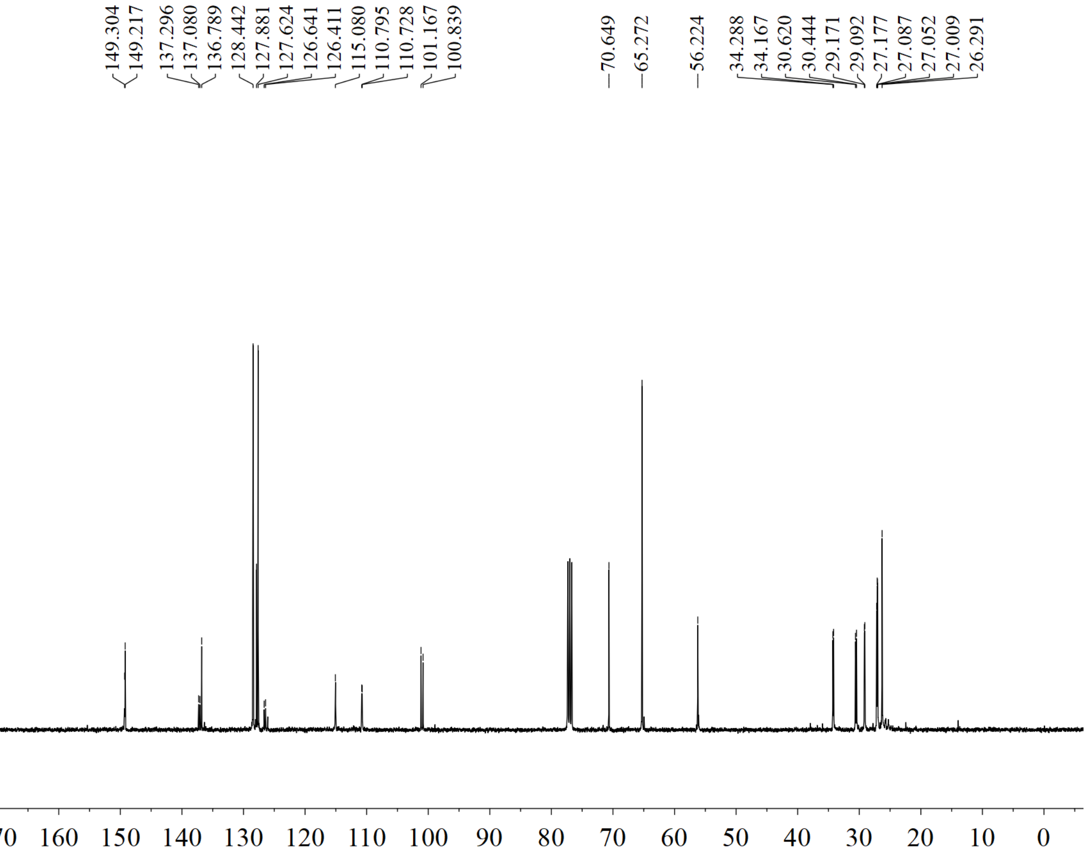

149.304
149.217
137.296
137.080
136.789
128.442
127.881
127.624
126.641
126.411
115.080
110.795
110.728
101.167
100.839
70.649
65.272
56.224
34.288
34.167
30.620
30.444
29.171
29.092
27.177
27.087
27.052
27.009
26.291
90 180 170 160 150 140 130 120 110 100 90 80 70 60 50 40 30 20 10 0

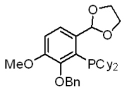

MeO
PCy2
OBn

Xu8-4

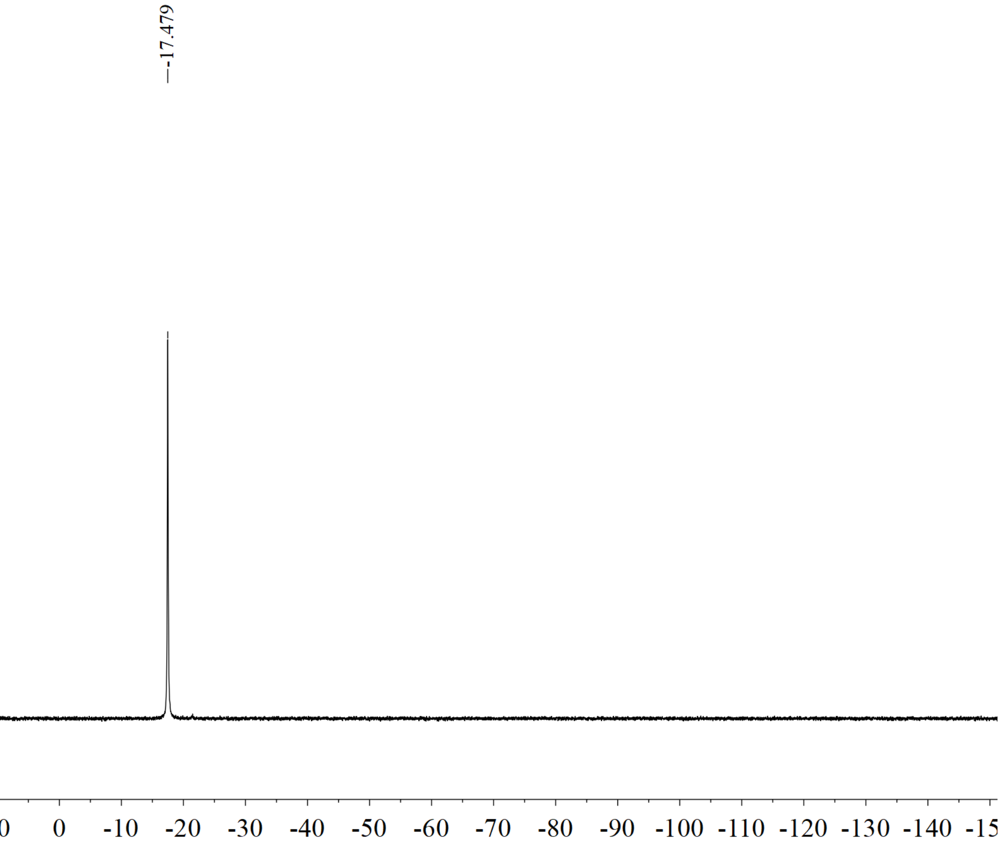

-17.479
40 30 20 10 0 -10 -20 -30 -40 -50 -60 -70 -80 -90 -100 -110 -120 -130 -140 -15

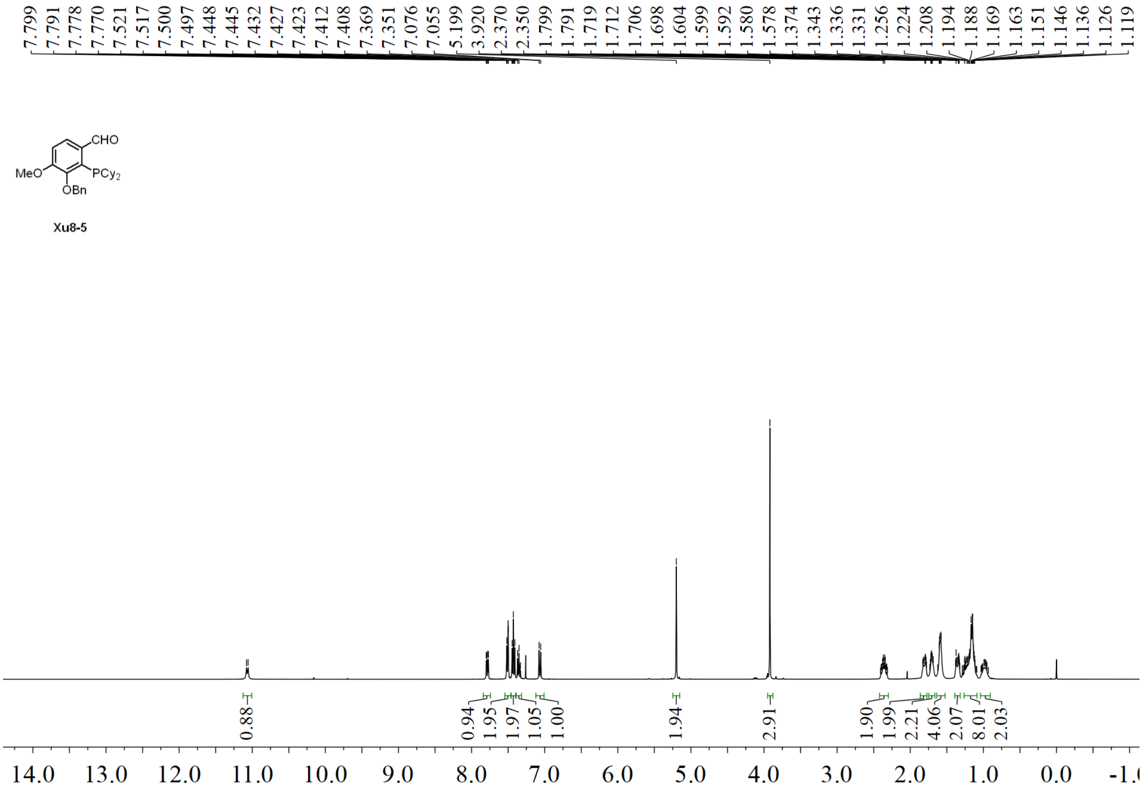

CHO
MeO
PCy2
OBn
Xu8-5
7.799 7.791 7.778 7.770 7.521 7.517 7.500 7.497 7.448 7.445 7.432 7.427 7.423 7.412 7.408 7.369 7.351 7.076 7.055 5.199 3.920 2.370 2.350 1.799 1.791 1.719 1.712 1.706 1.698 1.604 1.599 1.592 1.580 1.578 1.374 1.343 1.336 1.331 1.256 1.224 1.208 1.194 1.188 1.169 1.163 1.151 1.146 1.136 1.126 1.119
0.88 0.94 1.95 1.97 1.05 1.00 1.94 2.91 1.90 1.99 2.21 4.06 2.07 8.01 2.03
14.0 13.0 12.0 11.0 10.0 9.0 8.0 7.0 6.0 5.0 4.0 3.0 2.0 1.0 0.0

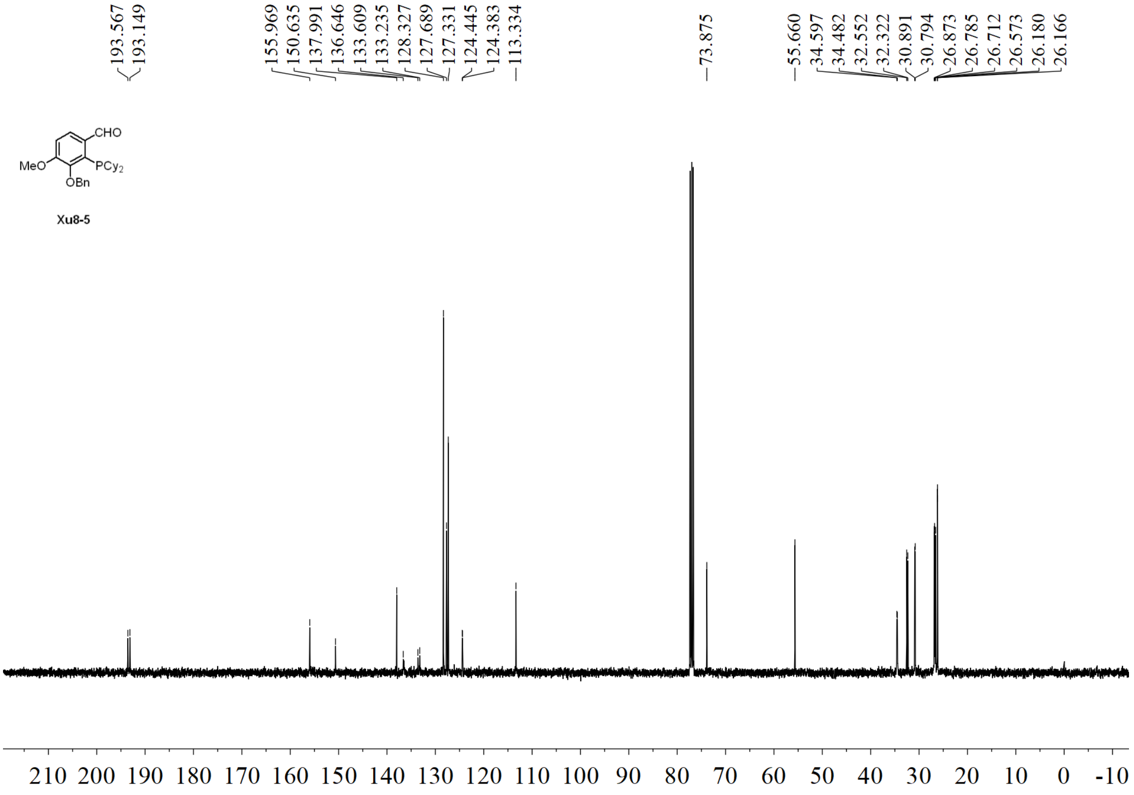

193.567 193.149 155.969 150.635 137.991 136.646 133.609 133.235 128.327 127.689 127.331 124.445 124.383 113.334 73.875 55.660 34.597 34.482 32.552 32.322 30.891 30.794 26.873 26.785 26.712 26.573 26.180 26.166
CHO
MeO
PCy2
OBn
Xu8-5
210 200 190 180 170 160 150 140 130 120 110 100 90 80 70 60 50 40 30 20 10 0 -10

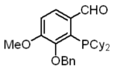
CHO
MeO
PCy2
OBn

Xu8-5

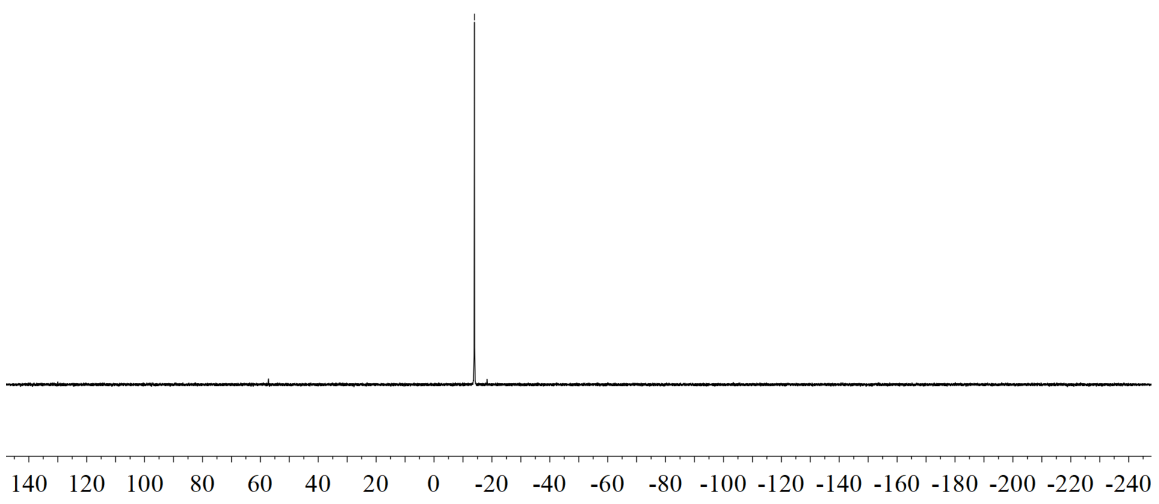
13.943
140 120 100 80 60 40 20 0 -20 -40 -60 -80 -100 -120 -140 -160 -180 -200 -220 -240

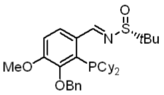
O
N
S
tBu
MeO
PCy2
OBn

Xu8-6

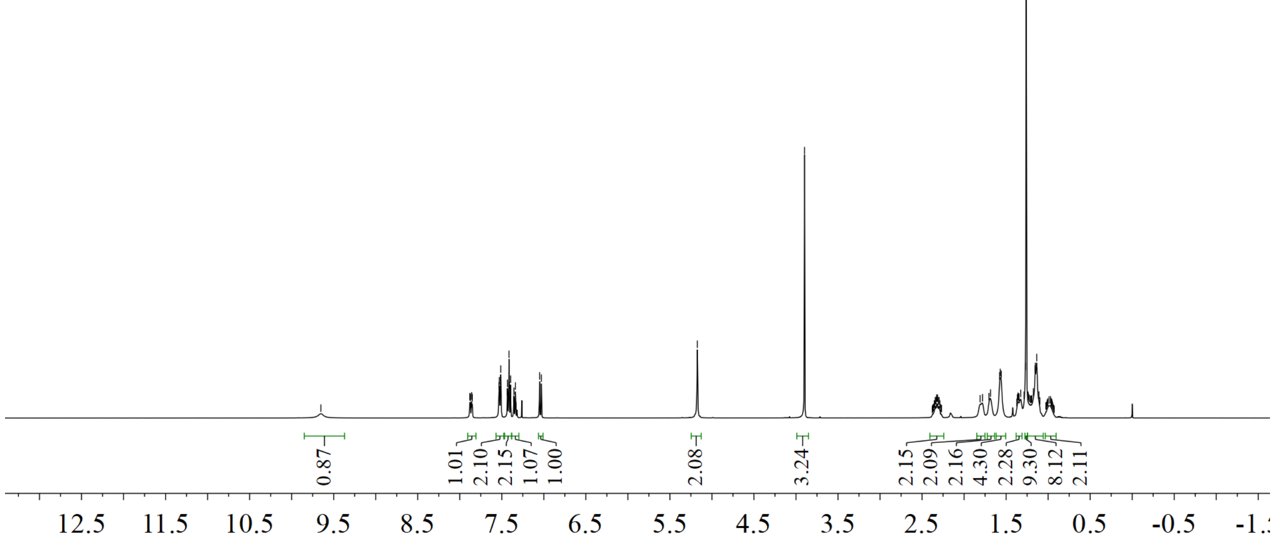
7.880 7.873 7.858 7.851 7.534 7.530 7.512 7.435 7.432 7.414 7.410 7.399 7.395 7.359 7.355 7.337 7.050 7.029 5.173 3.898 2.325 2.316 1.810 1.780 1.704 1.685 1.577 1.570 1.558 1.365 1.359 1.351 1.346 1.327 1.286 1.272 1.260 1.240 1.237 1.226 1.211 1.204 1.194 1.176 1.156 1.152 1.136 1.110 0.989 0.972
0.87 1.01 2.10 2.15 1.07 1.00 2.08 3.24 2.15 2.09 2.16 4.30 2.28 9.30 8.12 2.11
12.5 11.5 10.5 9.5 8.5 7.5 6.5 5.5 4.5 3.5 2.5 1.5 0.5 -0.5

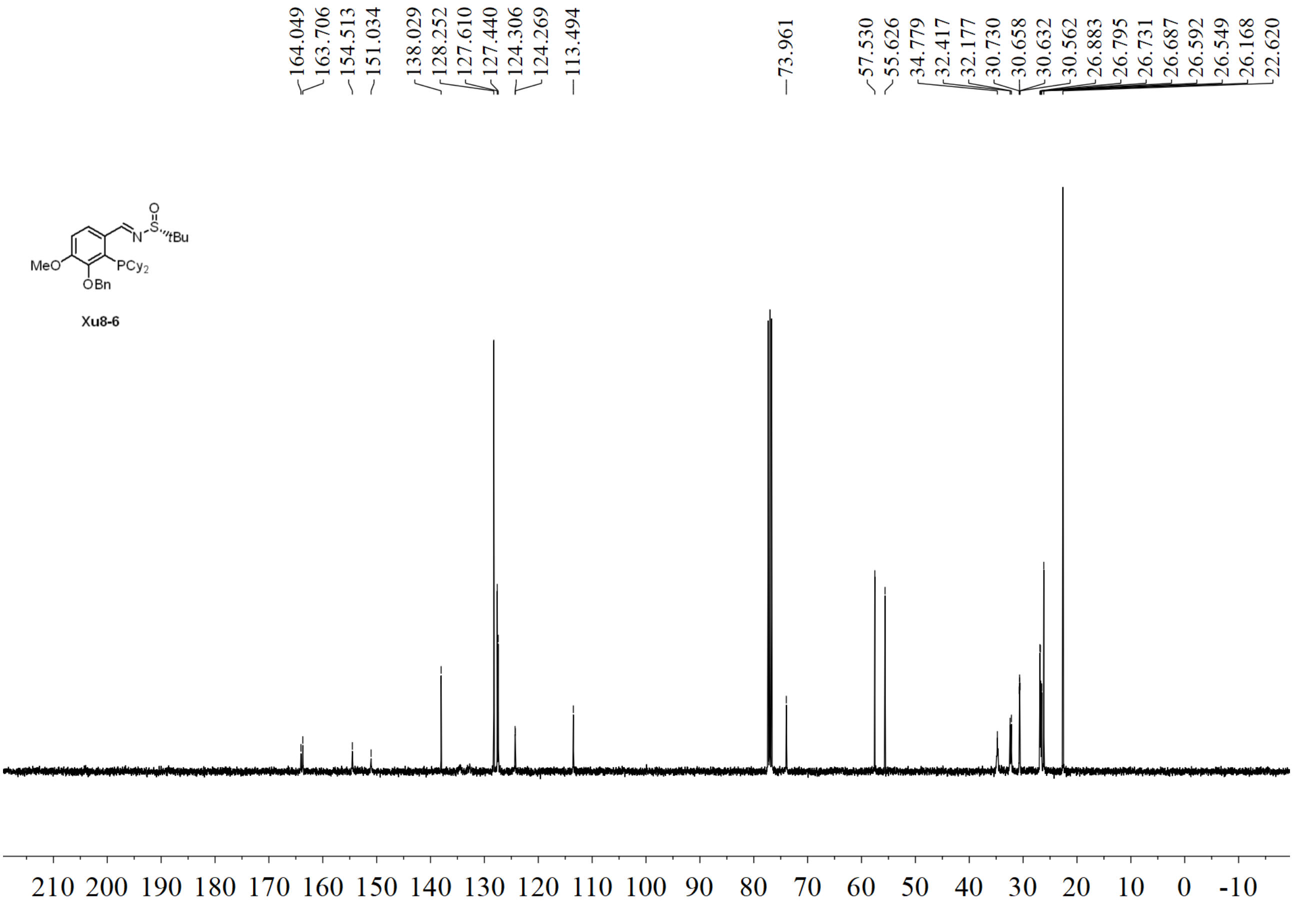

164.049
163.706
154.513
151.034
138.029
128.252
127.610
127.440
124.306
124.269
113.494
73.961
57.530
55.626
34.779
32.417
32.177
30.730
30.658
30.632
30.562
26.883
26.795
26.731
26.687
26.592
26.549
26.168
22.620
MeO
PCy2
OBn
S
O
N
tBu
Xu8-6
210 200 190 180 170 160 150 140 130 120 110 100 90 80 70 60 50 40 30 20 10 0 -10

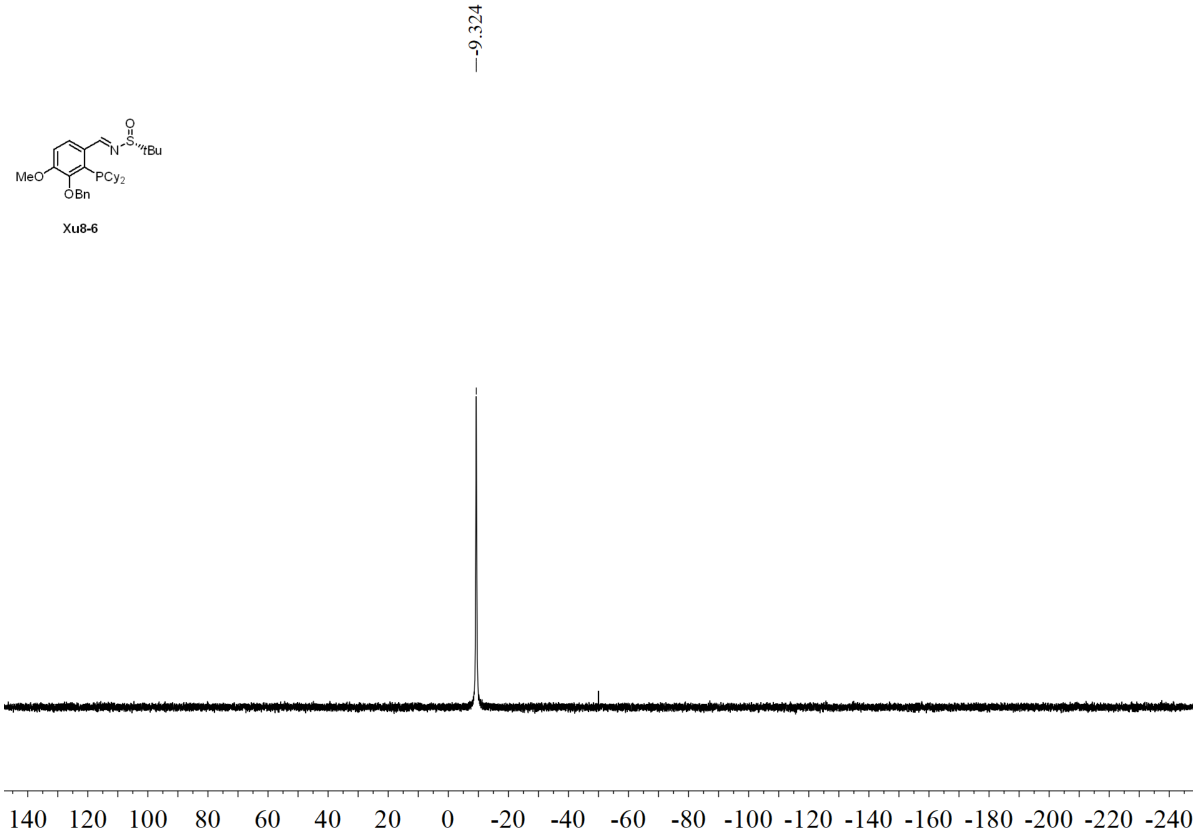

-9.324
MeO
PCy2
OBn
S
O
N
tBu
Xu8-6
140 120 100 80 60 40 20 0 -20 -40 -60 -80 -100 -120 -140 -160 -180 -200 -220 -240

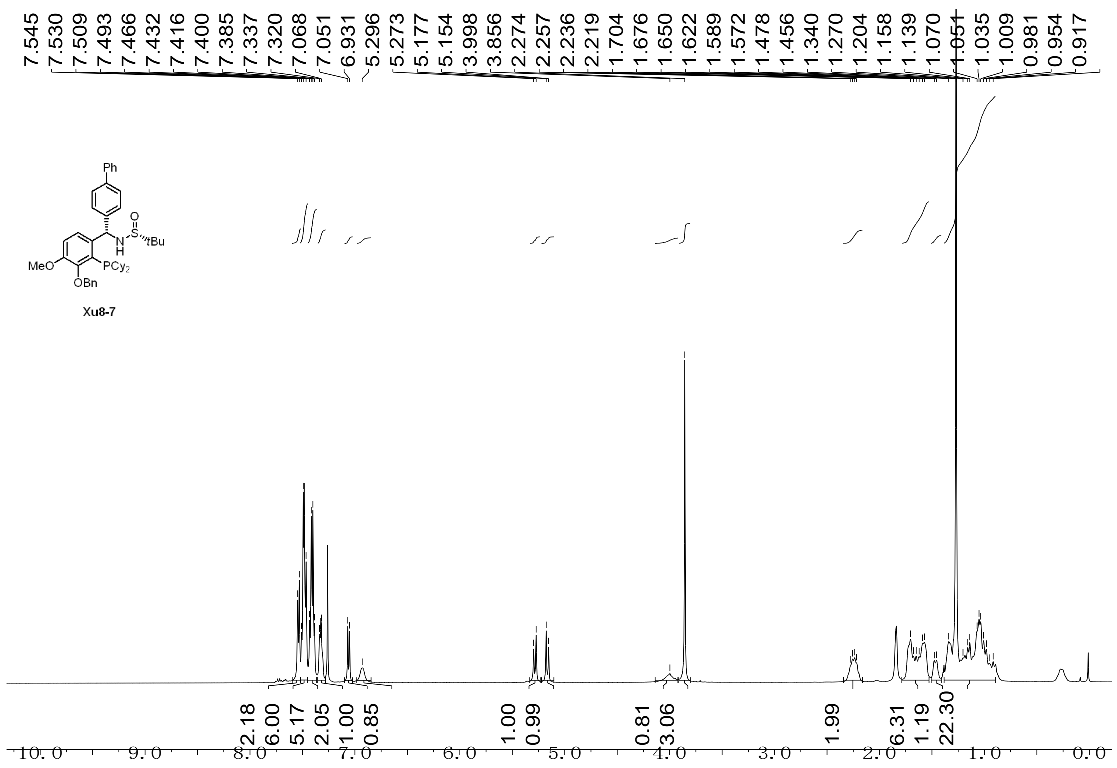
7.545
7.530
7.509
7.493
7.466
7.432
7.416
7.400
7.385
7.337
7.320
7.068
7.051
6.931
5.296
5.273
5.177
5.154
3.998
3.856
2.274
2.257
2.236
2.219
1.704
1.676
1.650
1.622
1.589
1.572
1.478
1.456
1.340
1.270
1.204
1.158
1.139
1.070
1.051
1.035
1.009
0.981
0.954
0.917
Ph
MeO
OBn
PCy2
N
H
S
O
tBu
Xu8-7
2.18
6.00
5.17
2.05
1.00
0.85
1.00
0.99
0.81
3.06
1.99
6.31
1.19
22.30
10.0
9.0
8.0
7.0
6.0
5.0
4.0
3.0
2.0
1.0
0.0

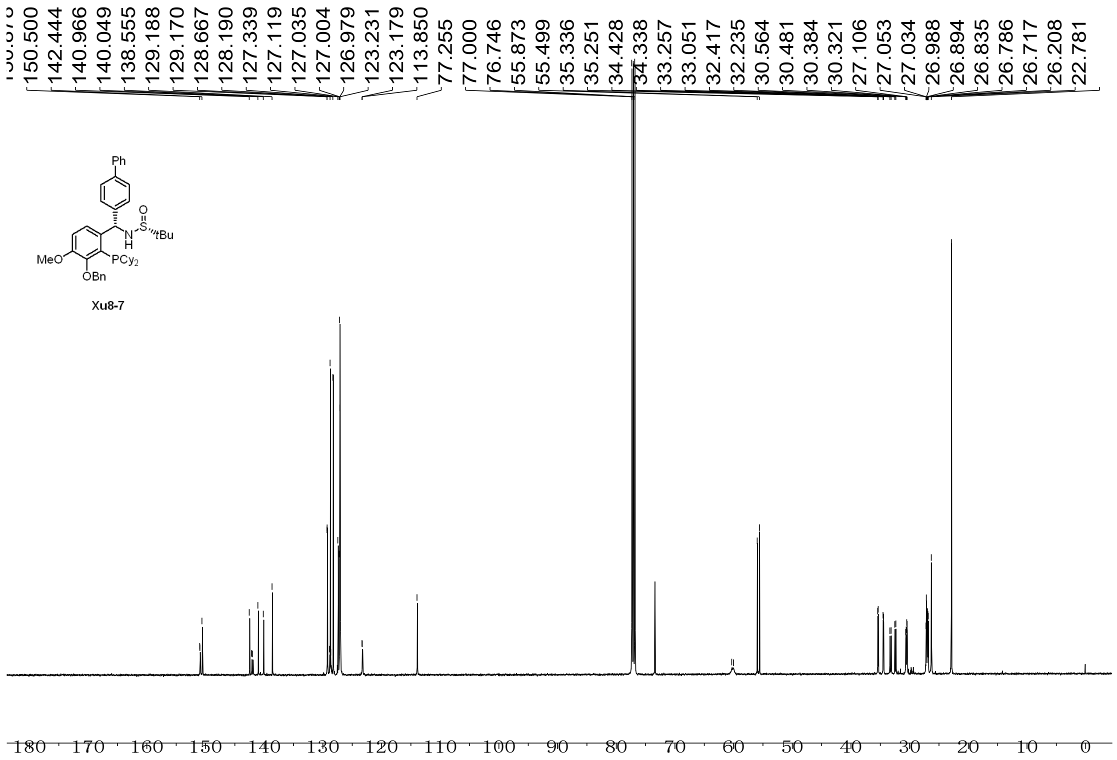
150.500
142.444
140.966
140.049
138.555
129.188
129.170
128.667
128.190
127.339
127.119
127.035
127.004
126.979
123.231
123.179
113.850
77.255
77.000
76.746
55.873
55.499
35.336
35.251
34.428
34.338
33.257
33.051
32.417
32.235
30.564
30.481
30.384
30.321
27.106
27.053
27.034
26.988
26.894
26.835
26.786
26.717
26.208
22.781
Ph
MeO
OBn
PCy2
N
H
S
O
tBu
Xu8-7
180
170
160
150
140
130
120
110
100
90
80
70
60
50
40
30
20
10
0

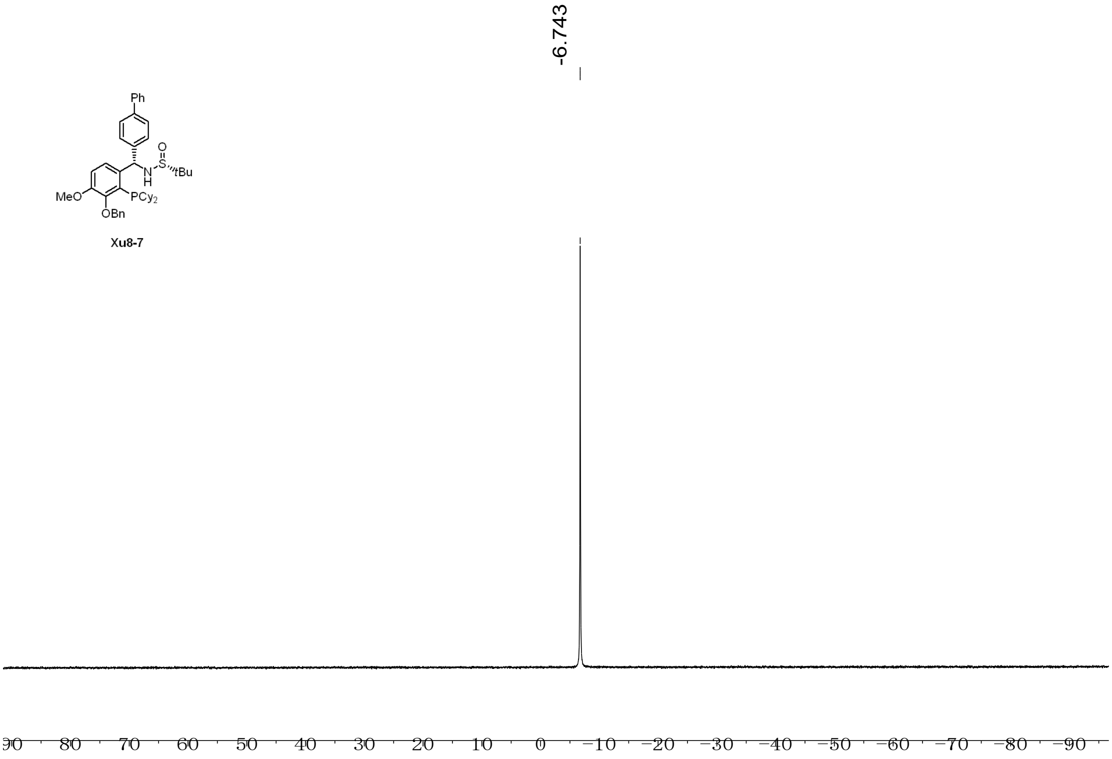
-6.743
Ph
O
S
N
H
tBu
MeO
PCy2
OBn
Xu8-7
90 80 70 60 50 40 30 20 10 0 −10 −20 −30 −40 −50 −60 −70 −80 −90

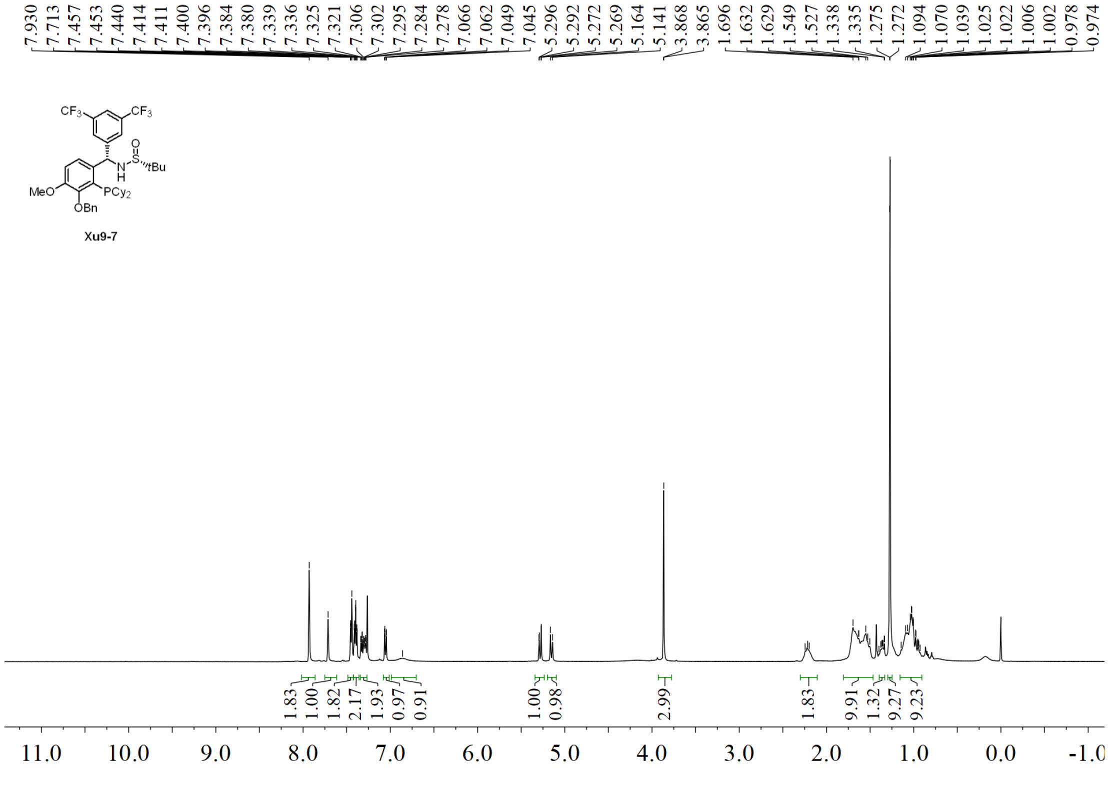
7.930
7.713
7.457
7.453
7.440
7.414
7.411
7.400
7.396
7.384
7.380
7.339
7.336
7.325
7.321
7.306
7.302
7.295
7.284
7.278
7.066
7.062
7.049
7.045
5.296
5.292
5.272
5.269
5.164
5.141
3.868
3.865
1.696
1.632
1.629
1.549
1.527
1.338
1.335
1.275
1.272
1.094
1.070
1.039
1.025
1.022
1.006
1.002
0.978
0.974
CF3
CF3
O
S
N
H
tBu
MeO
PCy2
OBn
Xu9-7
1.83
1.00
1.82
2.17
1.93
0.97
0.91
1.00
0.98
2.99
1.83
9.91
1.32
9.27
9.23
11.0 10.0 9.0 8.0 7.0 6.0 5.0 4.0 3.0 2.0 1.0 0.0 -1.0

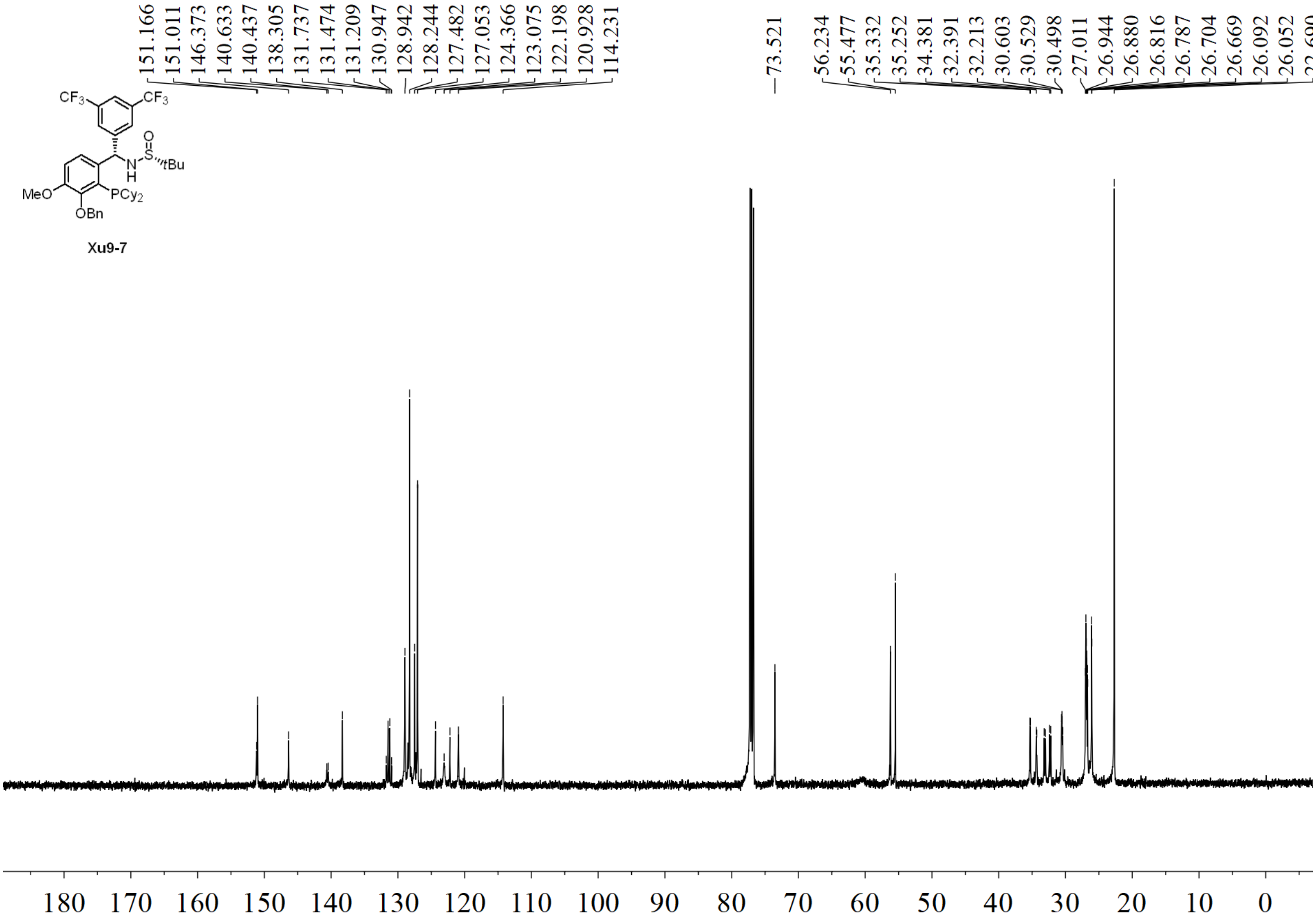
CF3
CF3
O
S
N
H
tBu
MeO
PCy2
OBn

Xu9-7

151.166
151.011
146.373
140.633
140.437
138.305
131.737
131.474
131.209
130.947
128.942
128.244
127.482
127.053
124.366
123.075
122.198
120.928
114.231
73.521
56.234
55.477
35.332
35.252
34.381
32.391
32.213
30.603
30.529
30.498
27.011
26.944
26.880
26.816
26.787
26.704
26.669
26.092
26.052
180 170 160 150 140 130 120 110 100 90 80 70 60 50 40 30 20 10 0

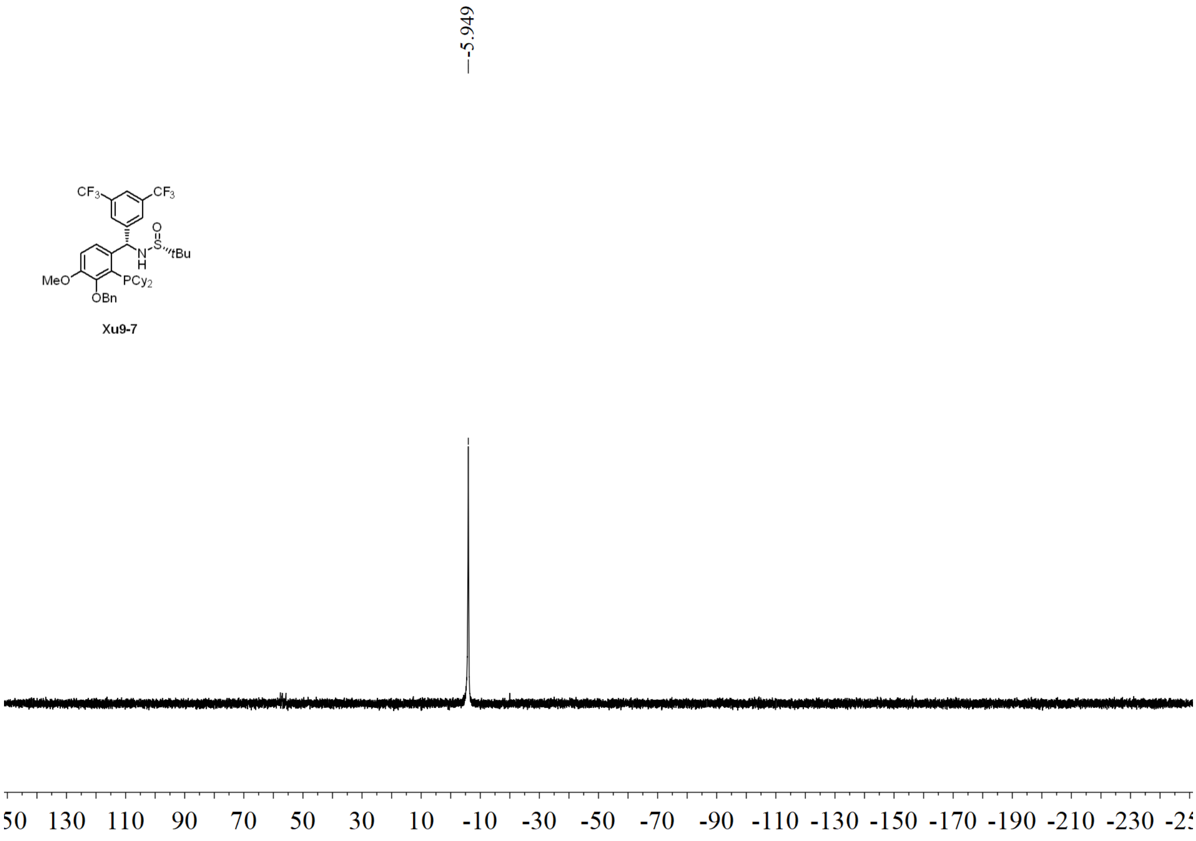
CF3
CF3
O
S
N
H
tBu
MeO
PCy2
OBn

Xu9-7

-5.949
130 110 90 70 50 30 10 -10 -30 -50 -70 -90 -110 -130 -150 -170 -190 -210 -230

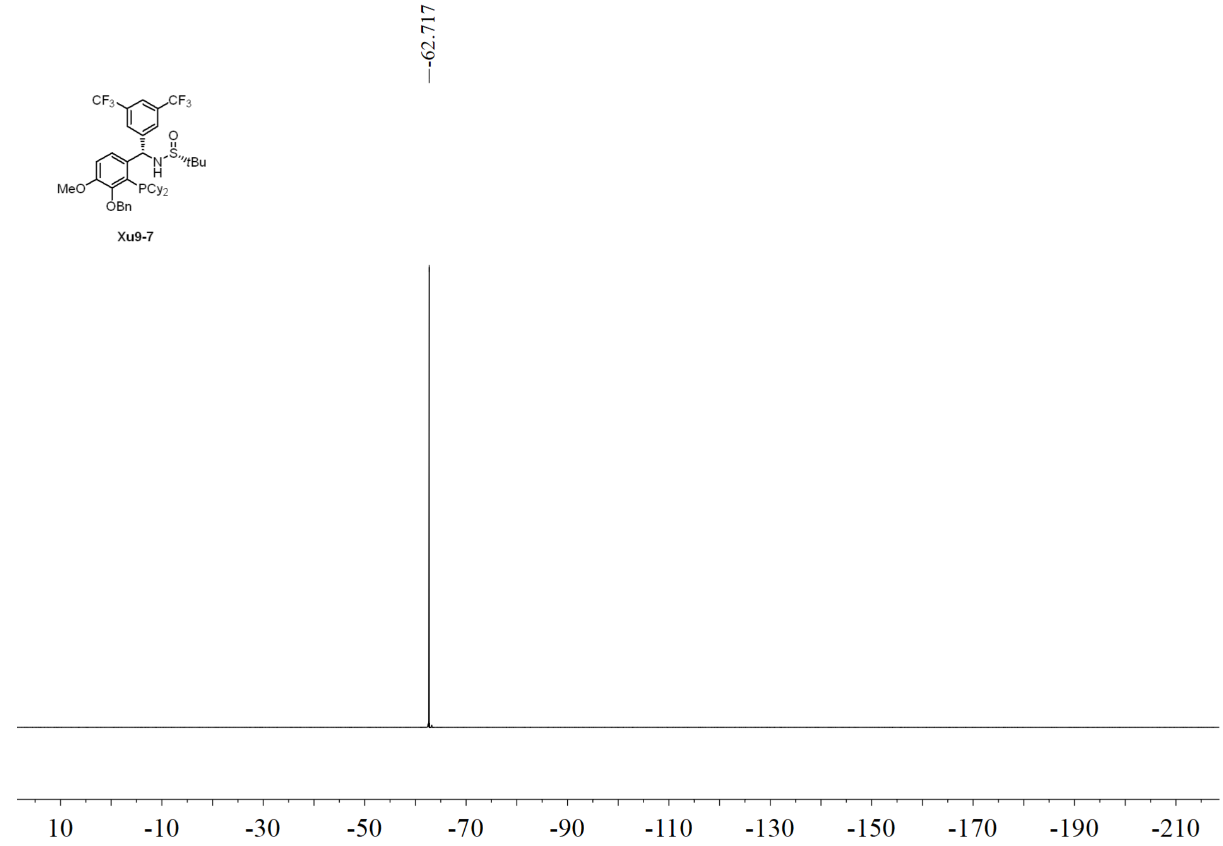
Xu9-7
−62.717

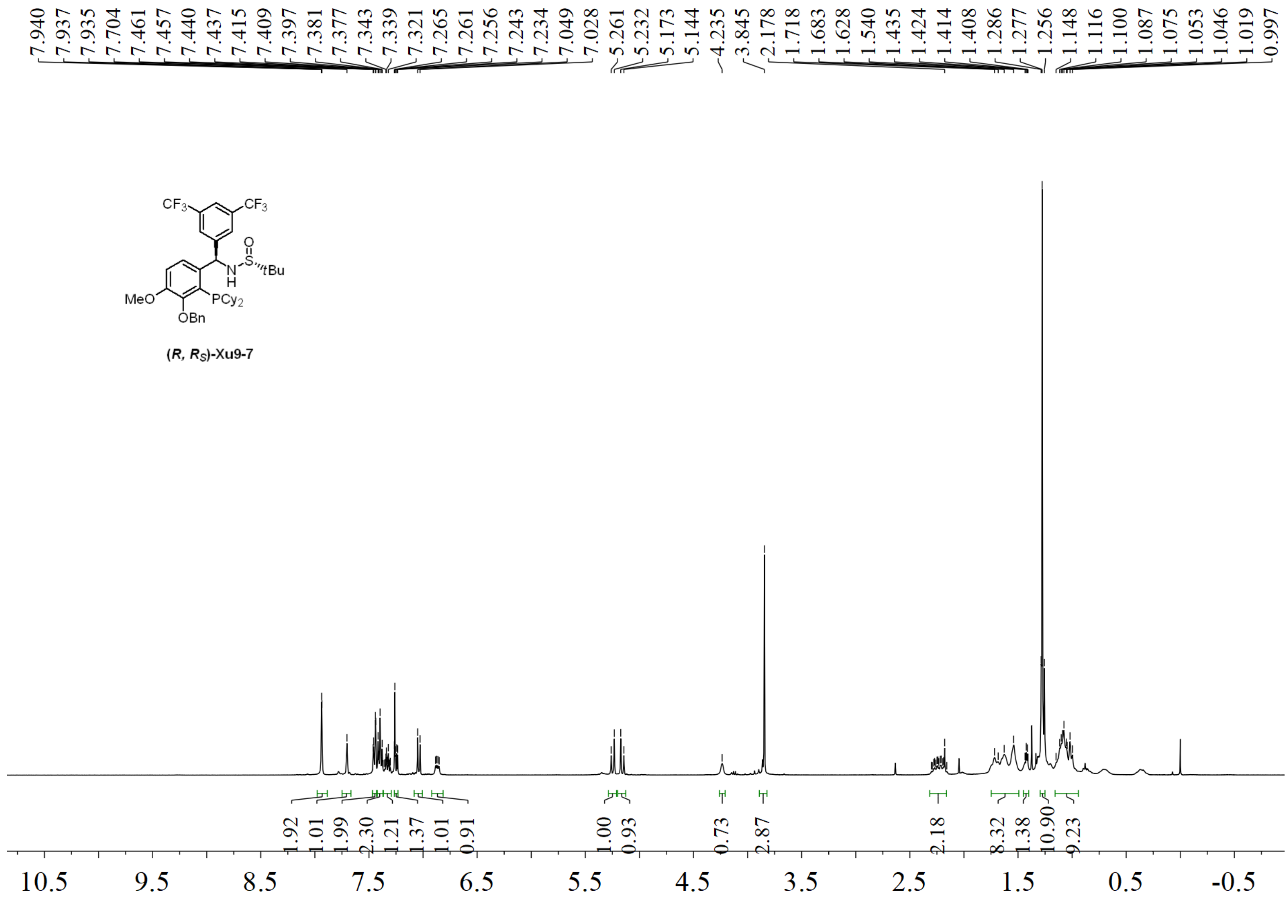
(R, RS)-Xu9-7

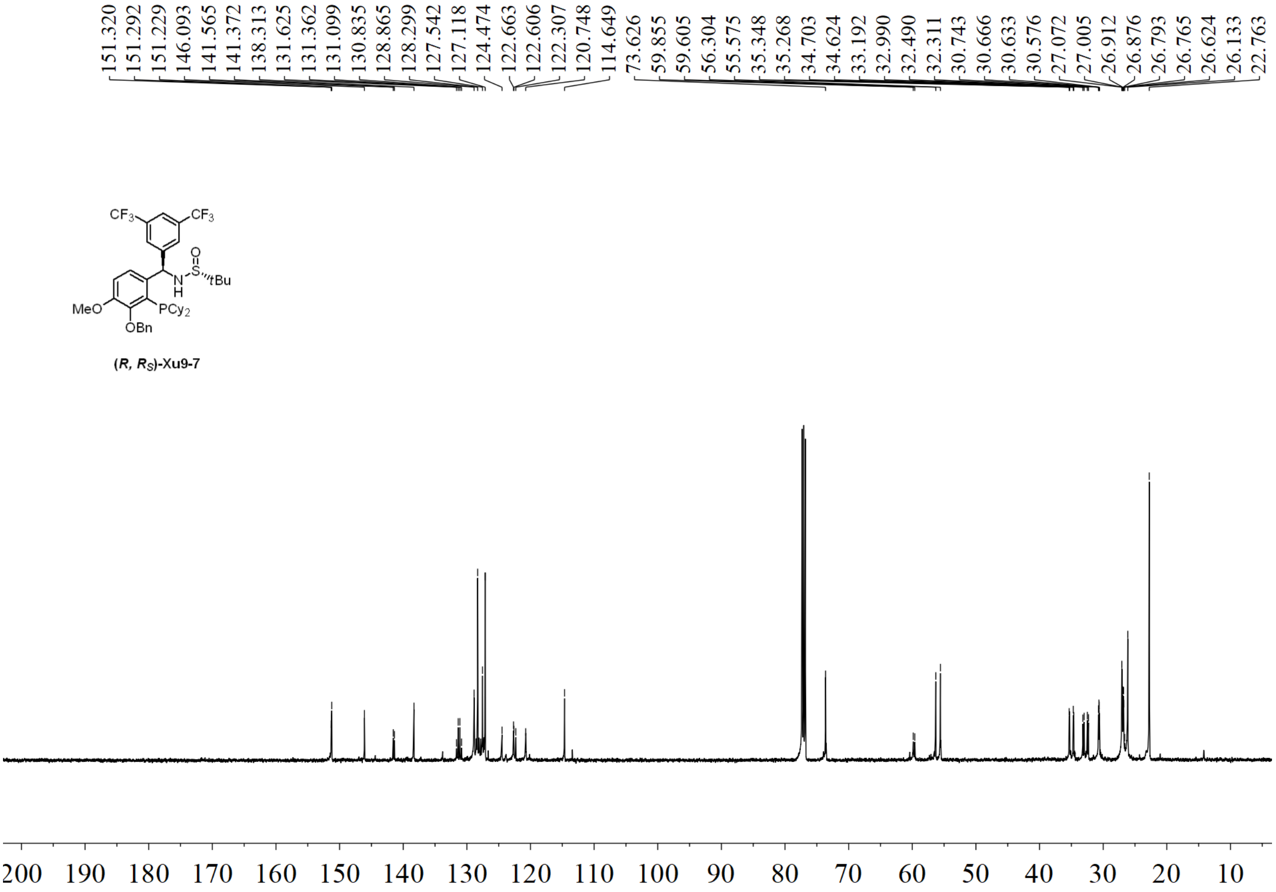
151.320
151.292
151.229
146.093
141.565
141.372
138.313
131.625
131.362
131.099
130.835
128.865
128.299
127.542
127.118
124.474
122.663
122.606
122.307
120.748
114.649
73.626
59.855
59.605
56.304
55.575
35.348
35.268
34.703
34.624
33.192
32.990
32.490
32.311
30.743
30.666
30.633
30.576
27.072
27.005
26.912
26.876
26.793
26.765
26.624
26.133
22.763
CF3
CF3
O
S
tBu
N
H
MeO
PCy2
OBn
(R, RS)-Xu9-7
200 190 180 170 160 150 140 130 120 110 100 90 80 70 60 50 40 30 20 10

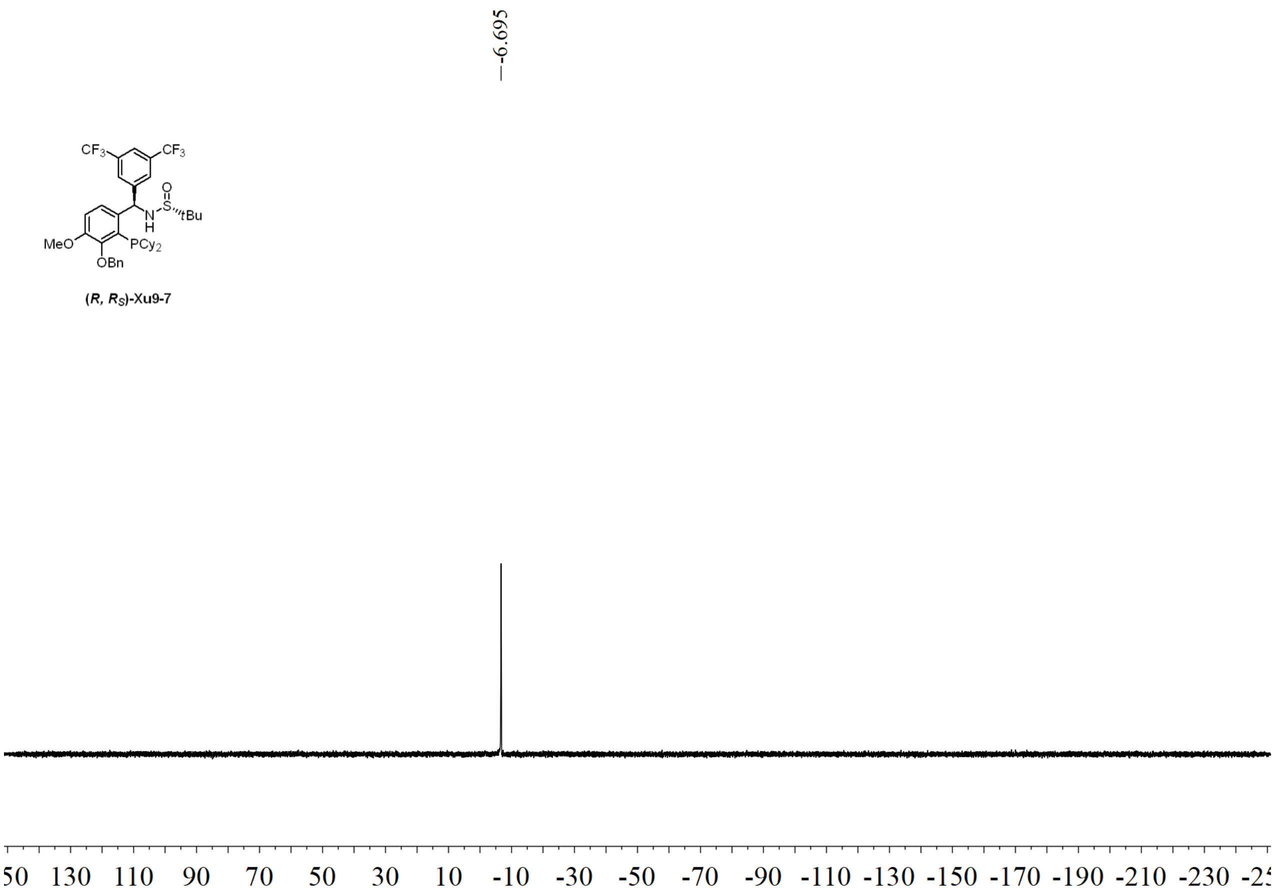
-6.695
CF3
CF3
O
S
tBu
N
H
MeO
PCy2
OBn
(R, RS)-Xu9-7
50 130 110 90 70 50 30 10 -10 -30 -50 -70 -90 -110 -130 -150 -170 -190 -210 -230

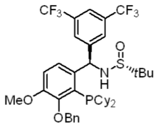

(*R*, *R*$_S$)-Xu9-7

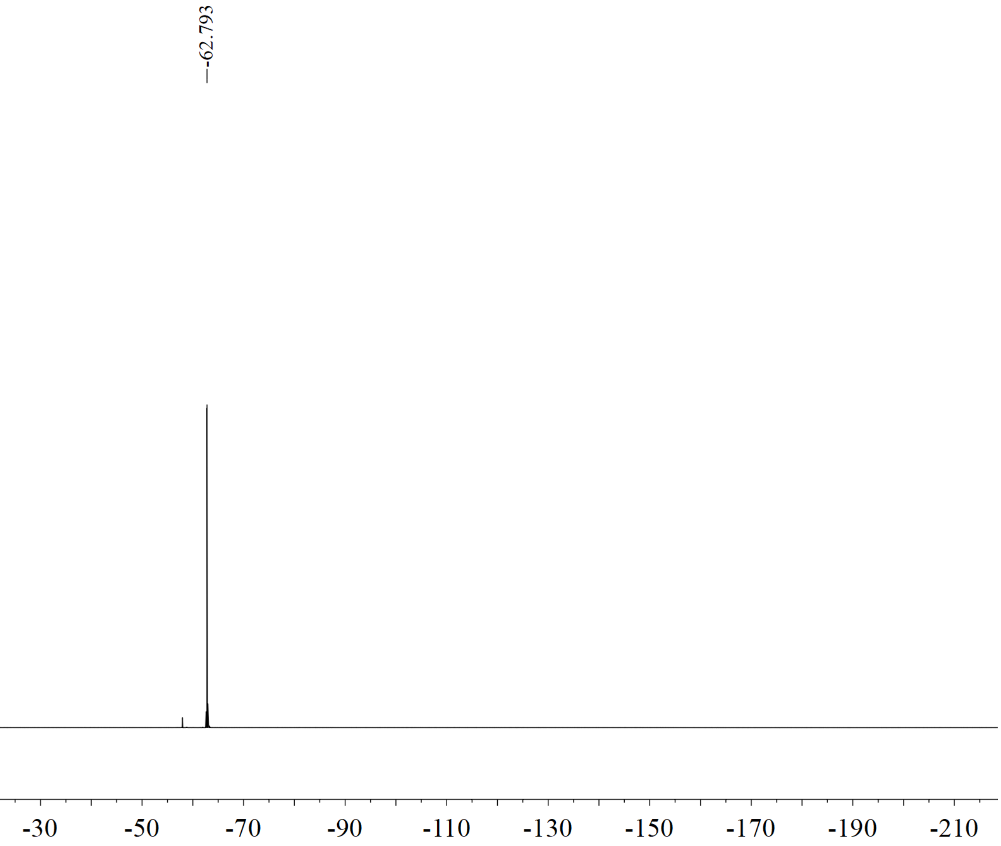

-62.793
10
-10
-30
-50
-70
-90
-110
-130
-150
-170
-190
-210

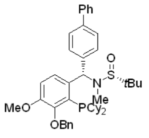

Xu8

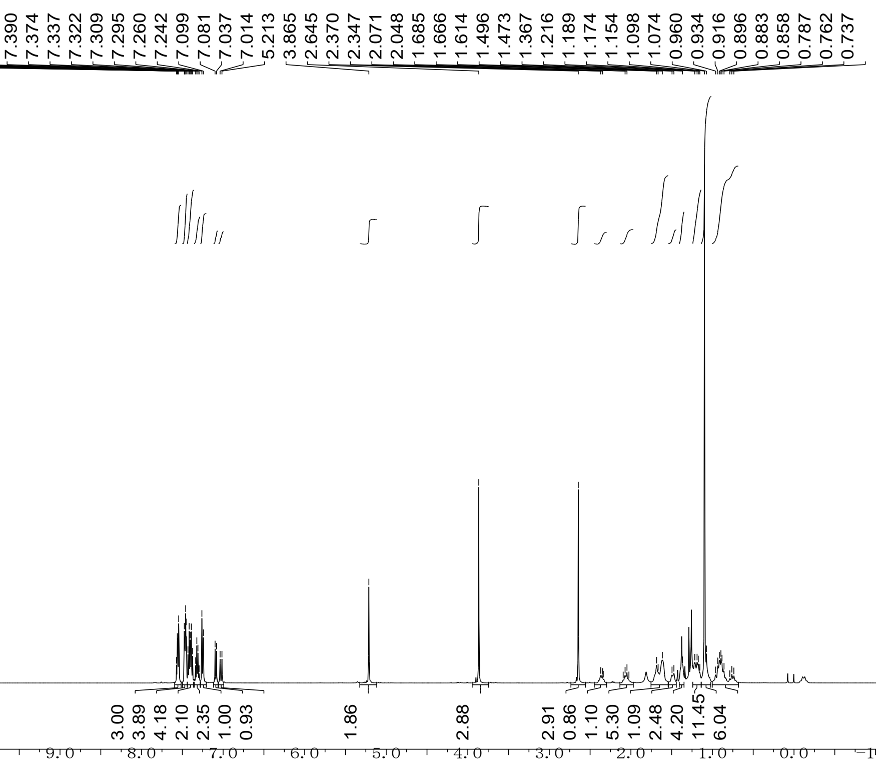

7.561
7.554
7.545
7.476
7.471
7.459
7.453
7.432
7.417
7.404
7.390
7.374
7.337
7.322
7.309
7.295
7.260
7.242
7.099
7.081
7.037
7.014
5.213
3.865
2.645
2.370
2.347
2.071
2.048
1.685
1.666
1.614
1.496
1.473
1.367
1.216
1.189
1.174
1.154
1.098
1.074
0.960
0.934
0.916
0.896
0.883
0.858
0.787
0.762
0.737
3.00
3.89
4.18
2.10
2.35
1.00
0.93
1.86
2.88
2.91
0.86
1.10
5.30
1.09
2.48
4.20
11.45
6.04
12.0
11.0
10.0
9.0
8.0
7.0
6.0
5.0
4.0
3.0
2.0
1.0
0.0

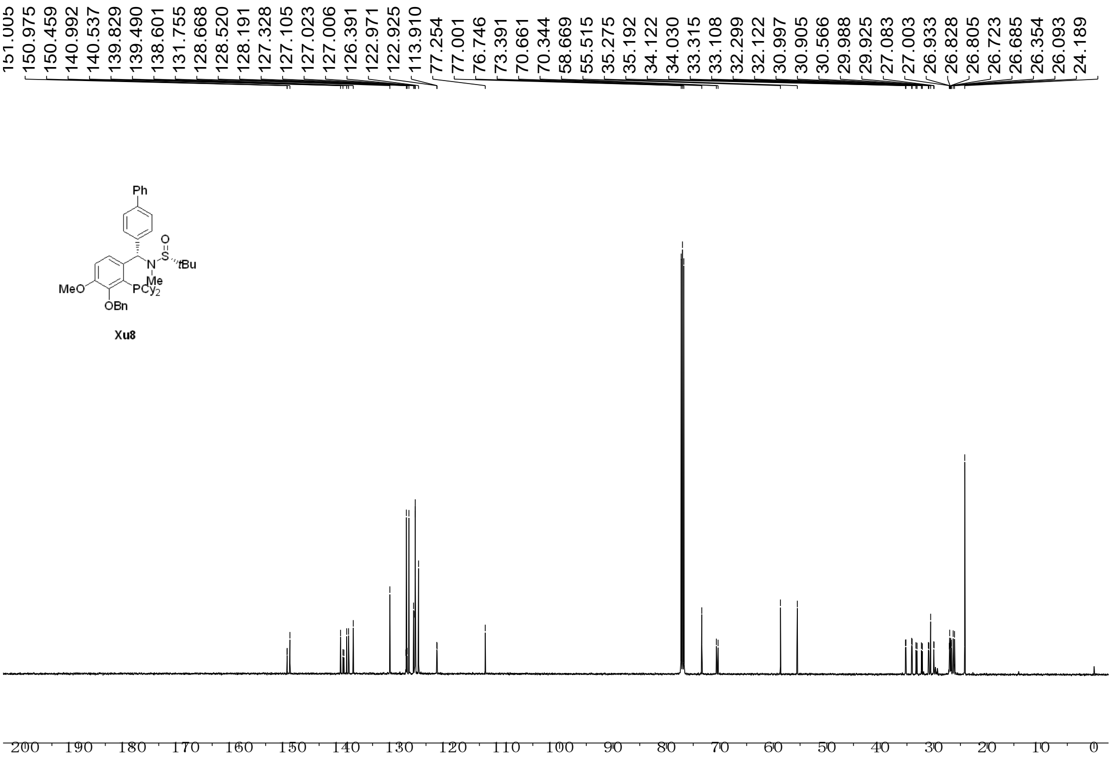
151.005
150.975
150.459
140.992
140.537
139.829
139.490
138.601
131.755
128.668
128.520
128.191
127.328
127.105
127.023
127.006
126.391
122.971
122.925
113.910
77.254
77.001
76.746
73.391
70.661
70.344
58.669
55.515
35.275
35.192
34.122
34.030
33.315
33.108
32.299
32.122
30.997
30.905
30.566
29.988
29.925
27.083
27.003
26.933
26.828
26.805
26.723
26.685
26.354
26.093
24.189
Ph
O
S
N
tBu
Me
MeO
PCy2
OBn
Xu8
200 190 180 170 160 150 140 130 120 110 100 90 80 70 60 50 40 30 20 10 0

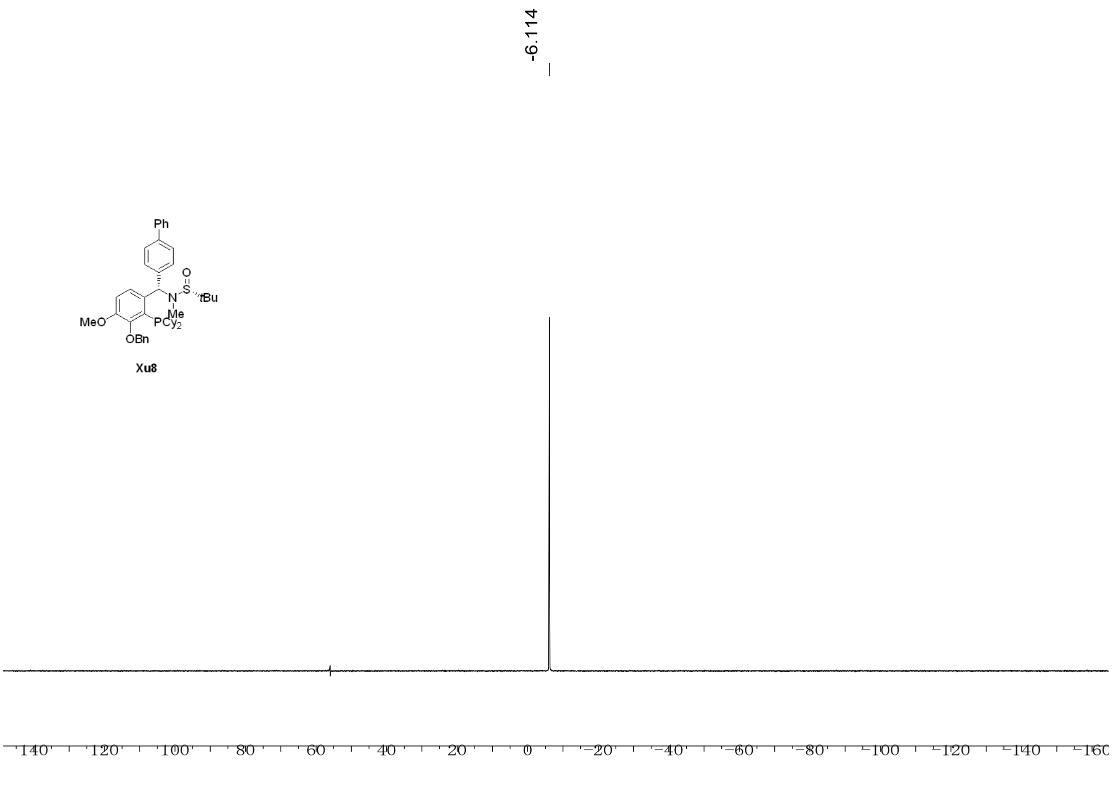
-6.114
Ph
O
S
N
tBu
Me
MeO
PCy2
OBn
Xu8
140 120 100 80 60 40 20 0 -20 -40 -60 -80 -100 -120 -140 -160

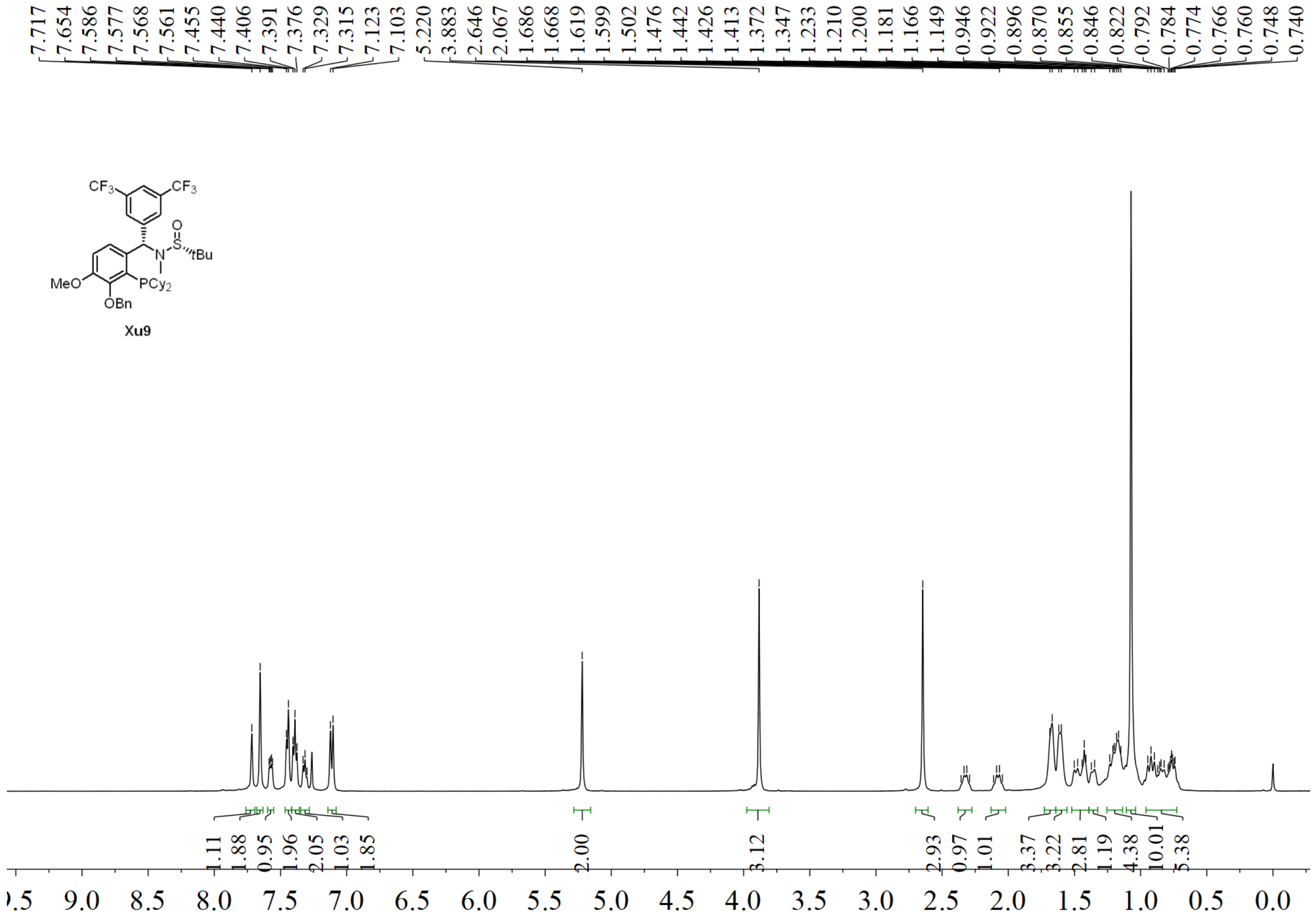
7.717 7.654 7.586 7.577 7.568 7.561 7.455 7.440 7.406 7.391 7.376 7.329 7.315 7.123 7.103 5.220 3.883 2.646 2.067 1.686 1.668 1.619 1.599 1.502 1.476 1.442 1.426 1.413 1.372 1.347 1.233 1.210 1.200 1.181 1.166 1.149 0.946 0.922 0.896 0.870 0.855 0.846 0.822 0.792 0.784 0.774 0.766 0.760 0.748 0.740
CF3 CF3 O S tBu N MeO PCy2 OBn
Xu9
1.11 1.88 0.95 1.96 2.05 1.03 1.85 2.00 3.12 2.93 0.97 1.01 3.37 3.22 2.81 1.19 4.38 10.01 5.38
9.0 8.5 8.0 7.5 7.0 6.5 6.0 5.5 5.0 4.5 4.0 3.5 3.0 2.5 2.0 1.5 1.0 0.5 0.0

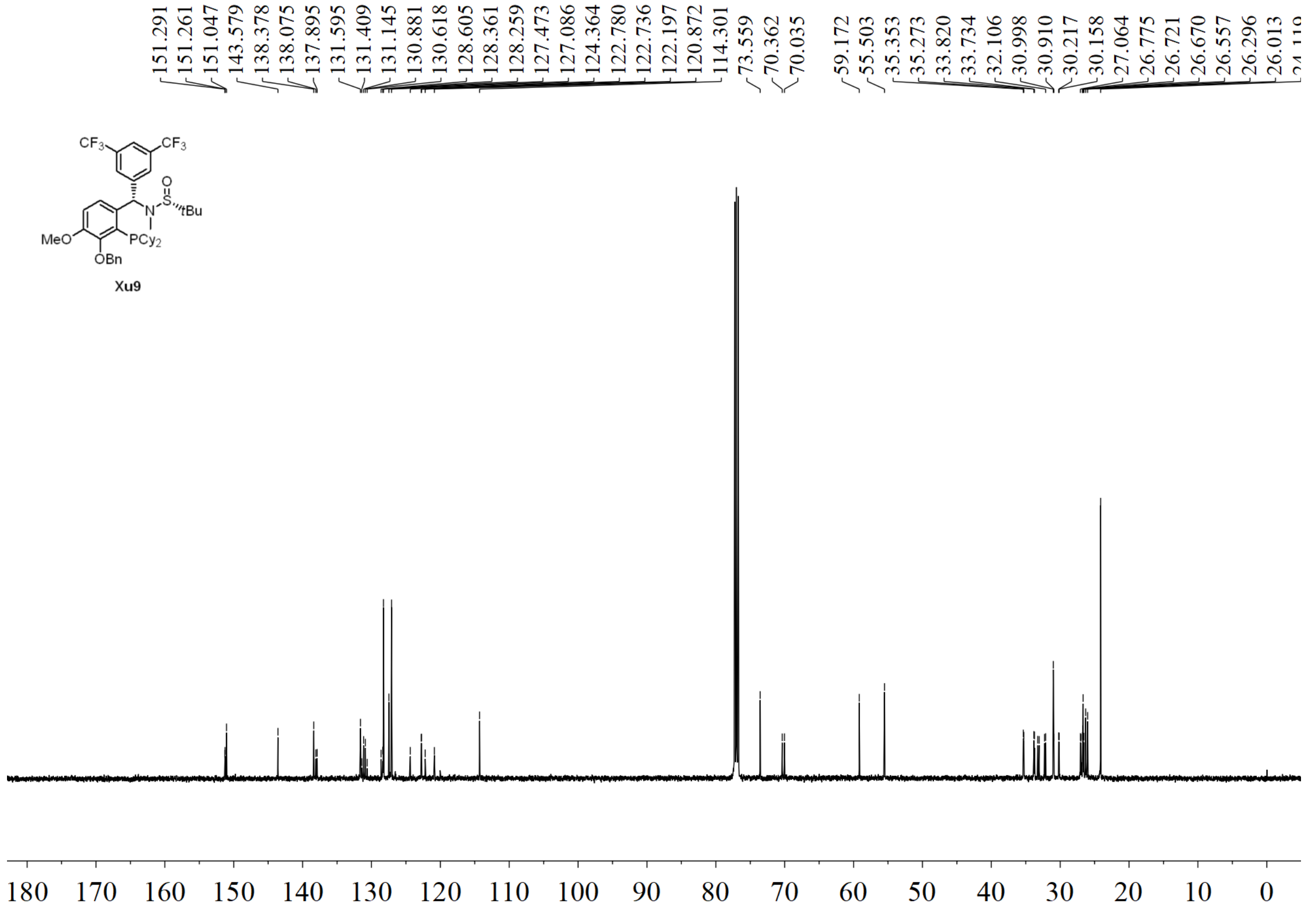
151.291 151.261 151.047 143.579 138.378 138.075 137.895 131.595 131.409 131.145 130.881 130.618 128.605 128.361 128.259 127.473 127.086 124.364 122.780 122.736 122.197 120.872 114.301 73.559 70.362 70.035 59.172 55.503 35.353 35.273 33.820 33.734 32.106 30.998 30.910 30.217 30.158 27.064 26.775 26.721 26.670 26.557 26.296 26.013
CF3 CF3 O S tBu N MeO PCy2 OBn
Xu9
180 170 160 150 140 130 120 110 100 90 80 70 60 50 40 30 20 10 0

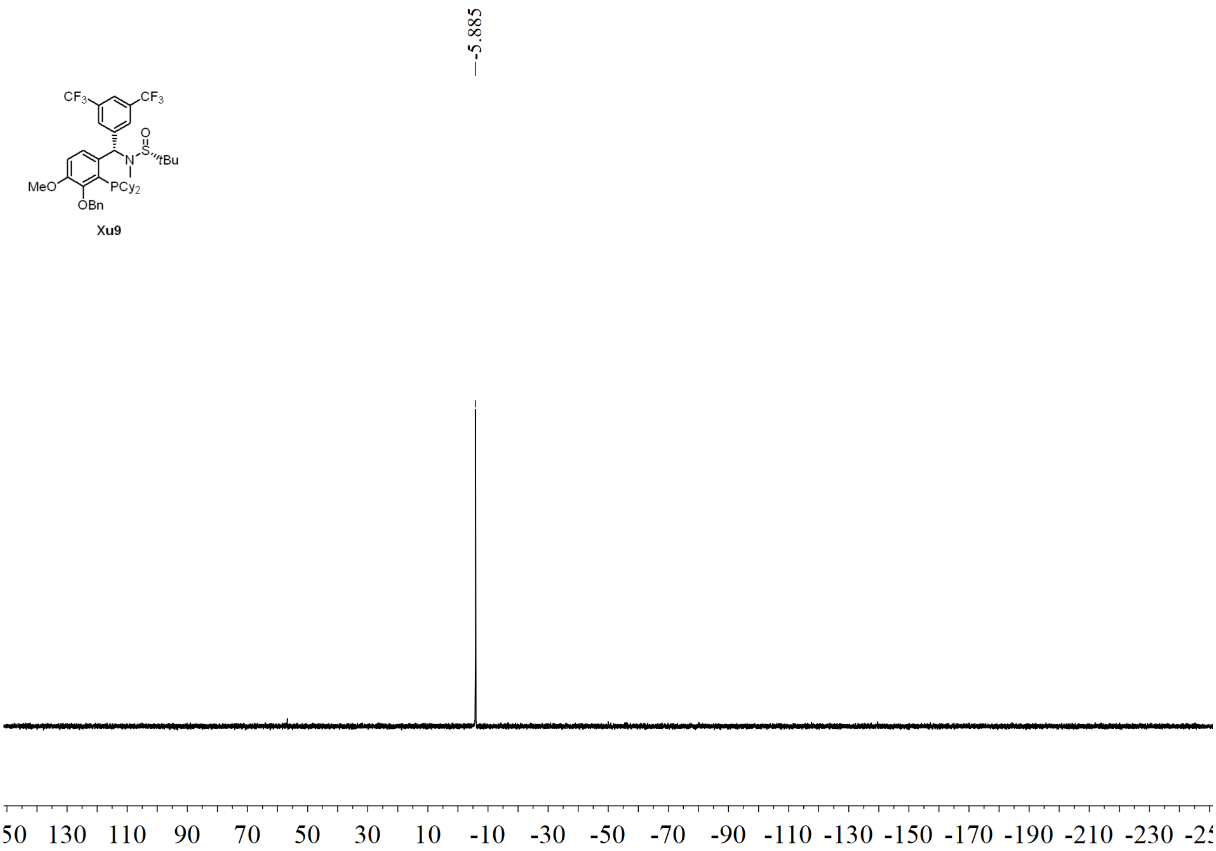

-5.885
CF3
CF3
O
S
N
tBu
MeO
PCy2
OBn
Xu9
50 130 110 90 70 50 30 10 -10 -30 -50 -70 -90 -110 -130 -150 -170 -190 -210 -230

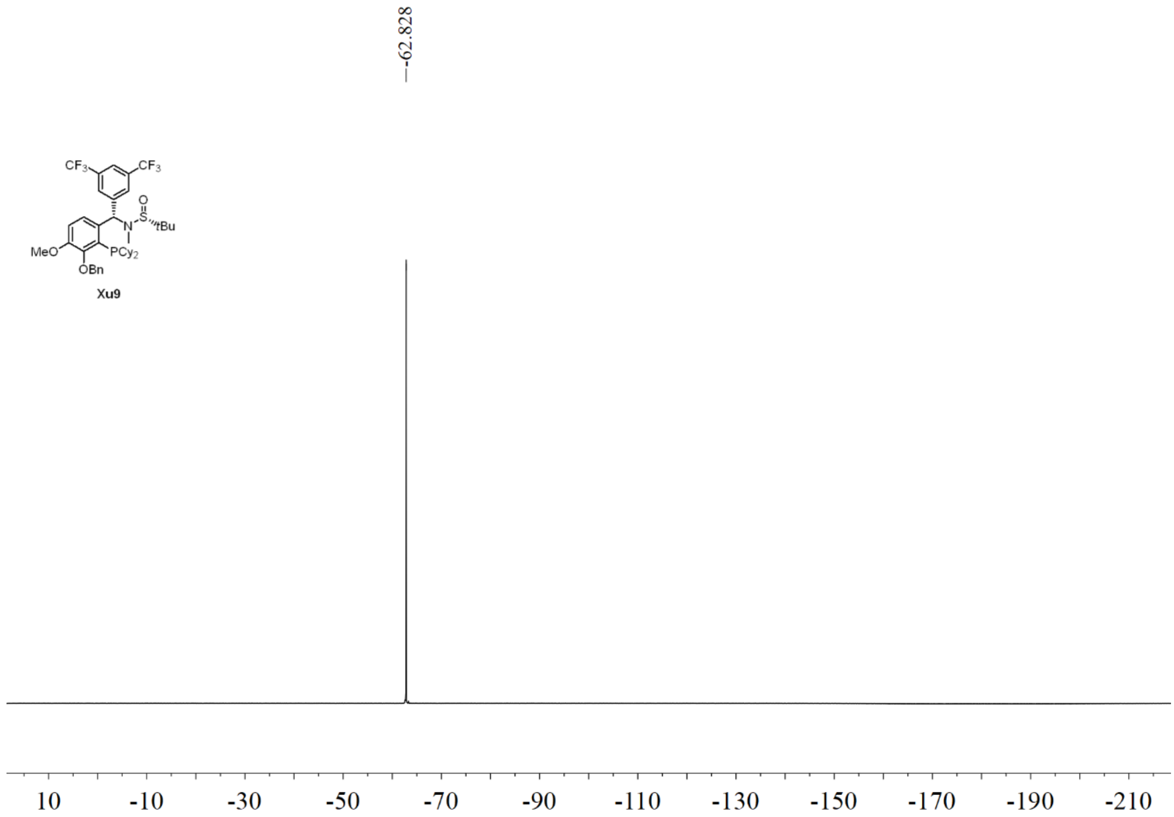

-62.828
CF3
CF3
O
S
N
tBu
MeO
PCy2
OBn
Xu9
10 -10 -30 -50 -70 -90 -110 -130 -150 -170 -190 -210

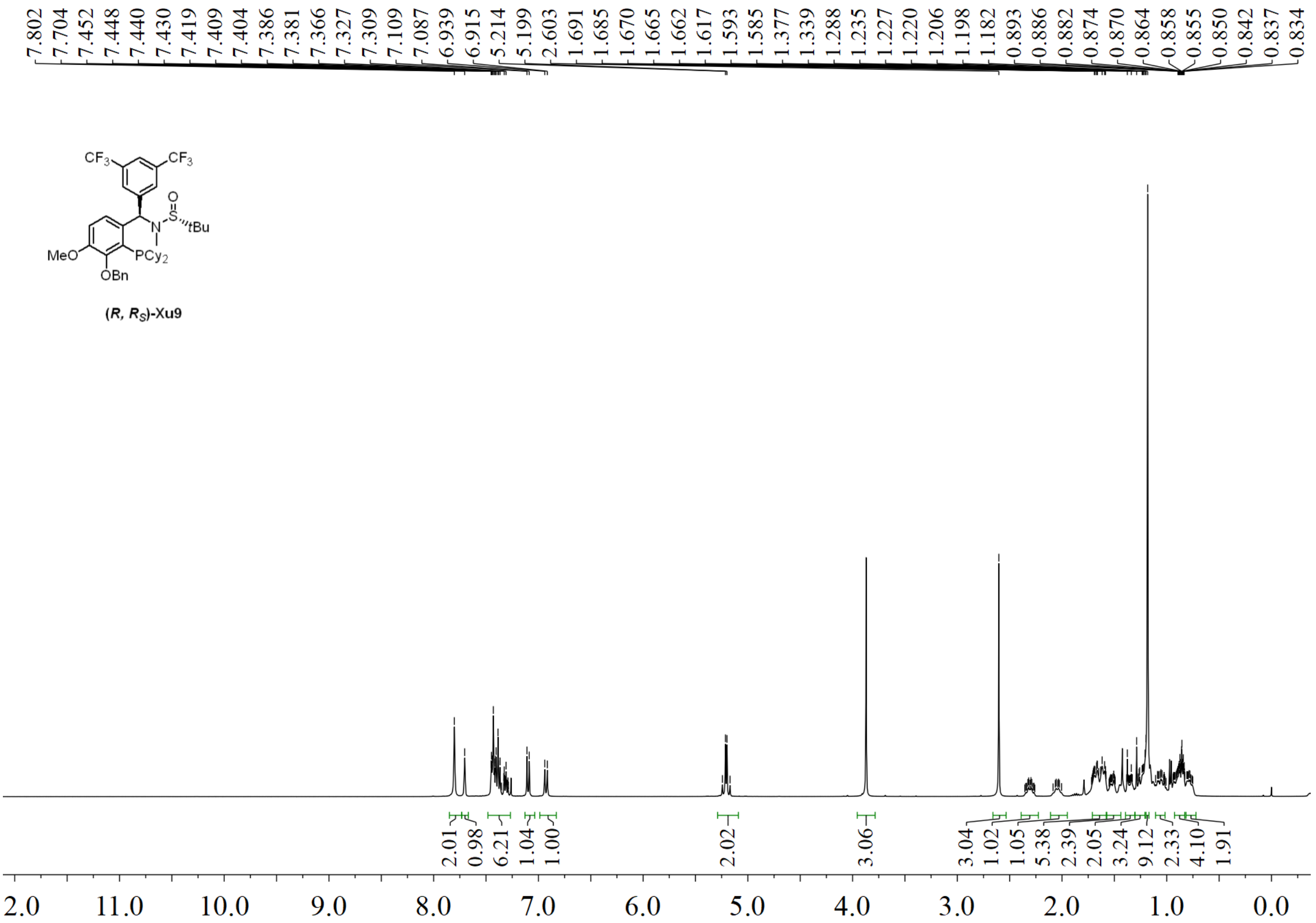
7.802 7.704 7.452 7.448 7.440 7.430 7.419 7.409 7.404 7.386 7.381 7.366 7.327 7.309 7.109 7.087 6.939 6.915 5.214 5.199 2.603 1.691 1.685 1.670 1.665 1.662 1.617 1.593 1.585 1.377 1.339 1.288 1.235 1.227 1.220 1.206 1.198 1.182 0.893 0.886 0.882 0.874 0.870 0.864 0.858 0.855 0.850 0.842 0.837 0.834
CF3
CF3
O
S
tBu
N
MeO
PCy2
OBn
(R, RS)-Xu9
2.01 0.98 6.21 1.04 1.00 2.02 3.06 3.04 1.02 1.05 5.38 2.39 2.05 3.24 9.12 2.33 4.10 1.91
2.0 11.0 10.0 9.0 8.0 7.0 6.0 5.0 4.0 3.0 2.0 1.0 0.0

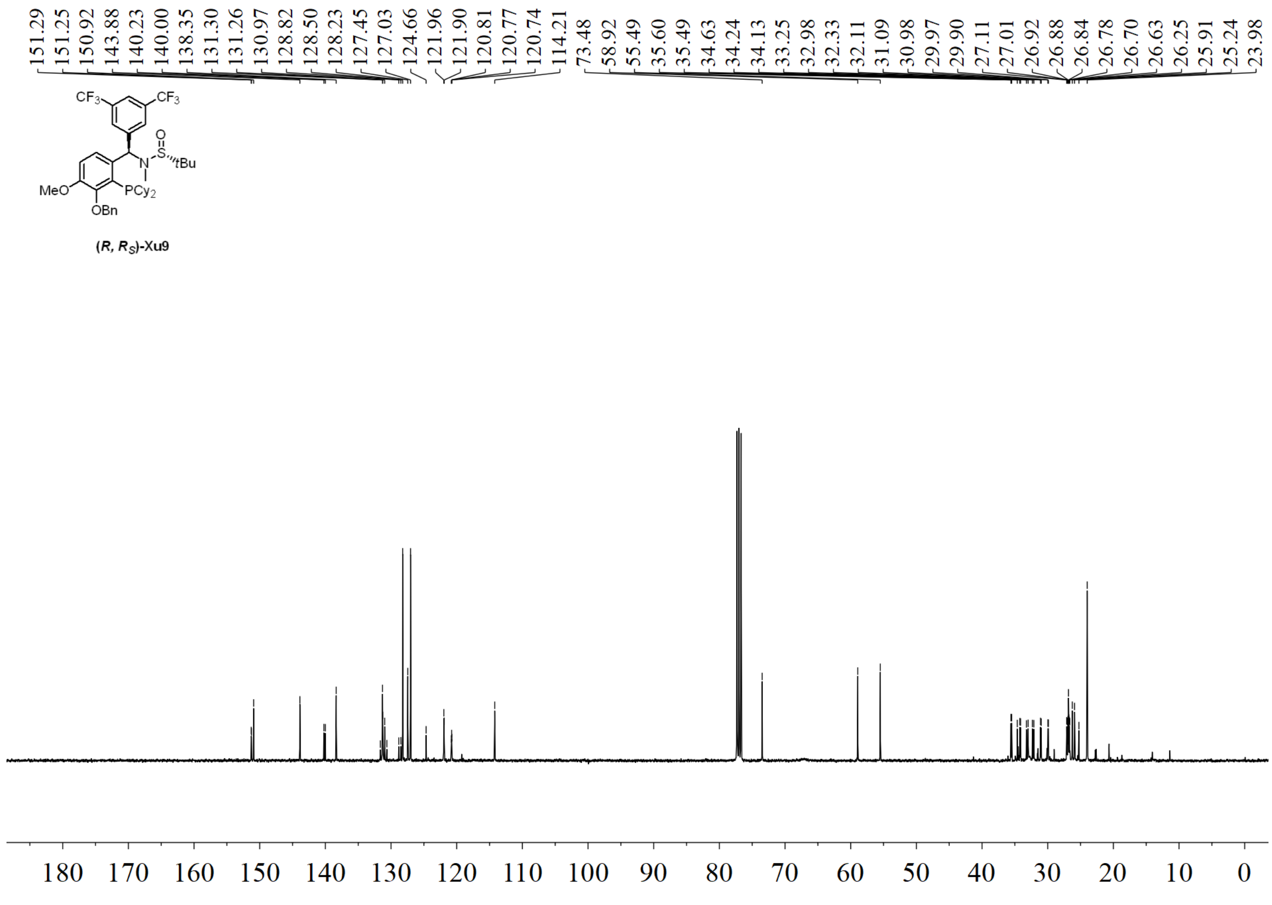
151.29 151.25 150.92 143.88 140.23 140.00 138.35 131.30 131.26 130.97 128.82 128.50 128.23 127.45 127.03 124.66 121.96 121.90 120.81 120.77 120.74 114.21 73.48 58.92 55.49 35.60 35.49 34.63 34.24 34.13 33.25 32.98 32.33 32.11 31.09 30.98 29.97 29.90 27.11 27.01 26.92 26.88 26.84 26.78 26.70 26.63 26.25 25.91 25.24 23.98
CF3
CF3
O
S
tBu
N
MeO
PCy2
OBn
(R, RS)-Xu9
180 170 160 150 140 130 120 110 100 90 80 70 60 50 40 30 20 10 0

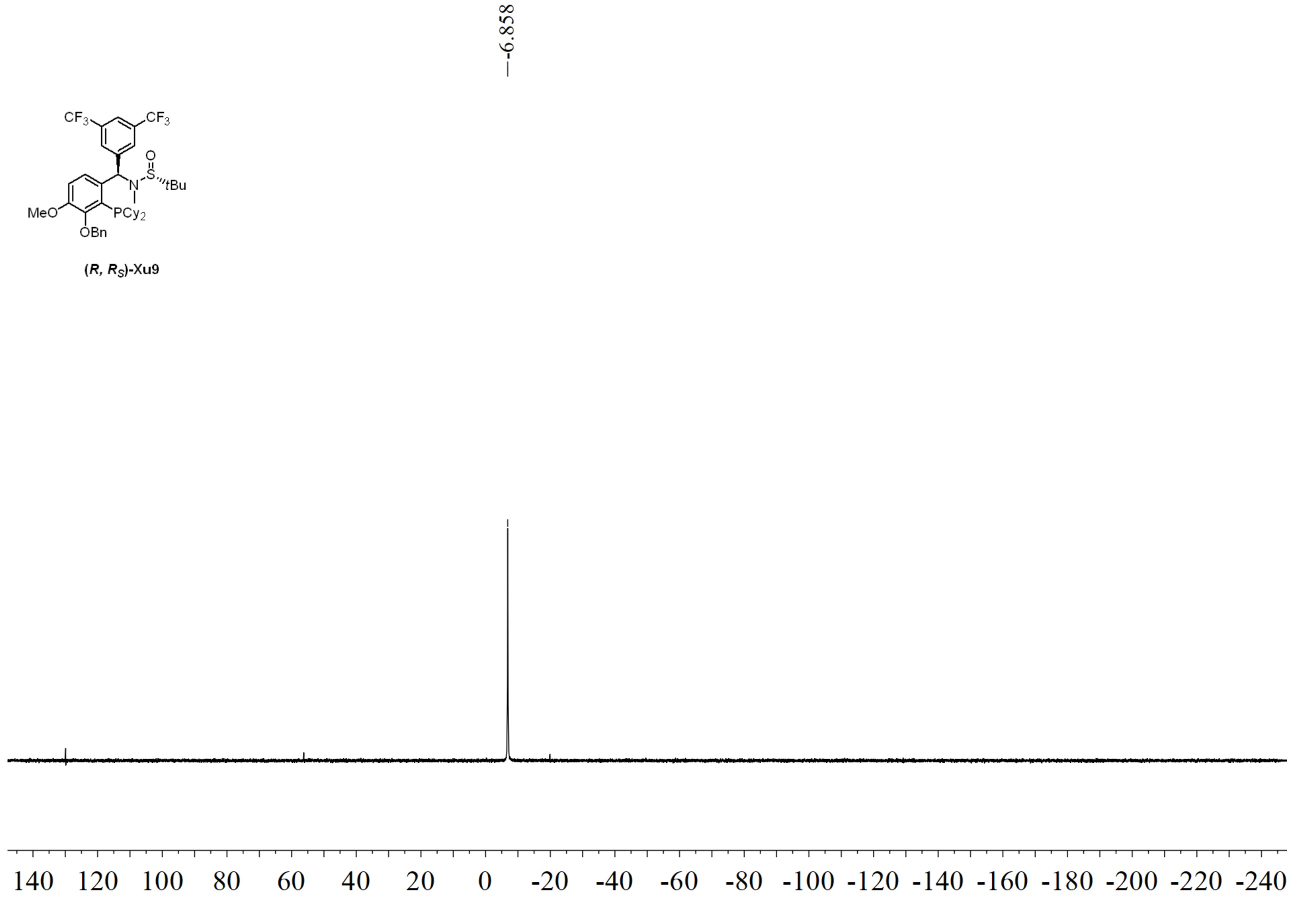

-6.858
CF3
CF3
MeO
OBn
PCy2
N
S
O
tBu
(R, RS)-Xu9
140 120 100 80 60 40 20 0 -20 -40 -60 -80 -100 -120 -140 -160 -180 -200 -220 -240

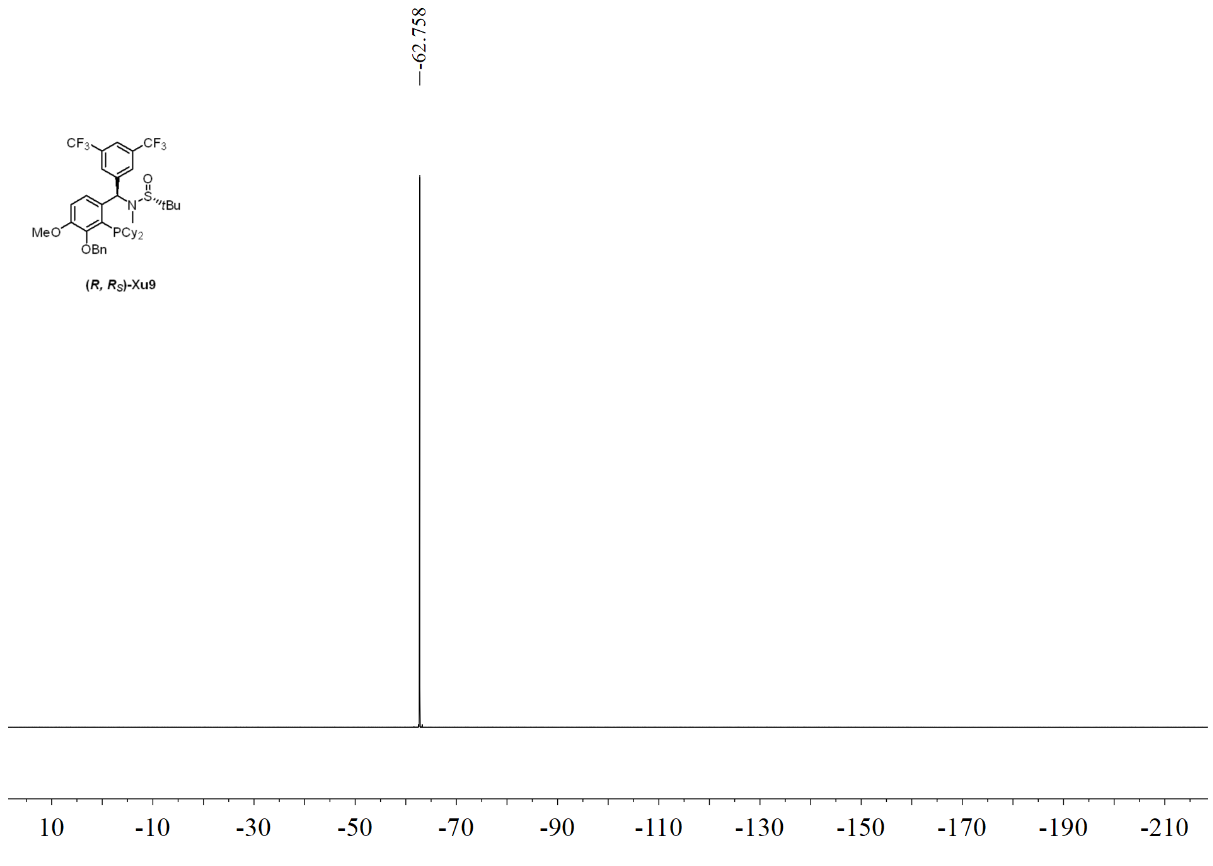

-62.758
CF3
CF3
MeO
OBn
PCy2
N
S
O
tBu
(R, RS)-Xu9
10 -10 -30 -50 -70 -90 -110 -130 -150 -170 -190 -210

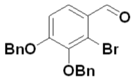

Xu10-2

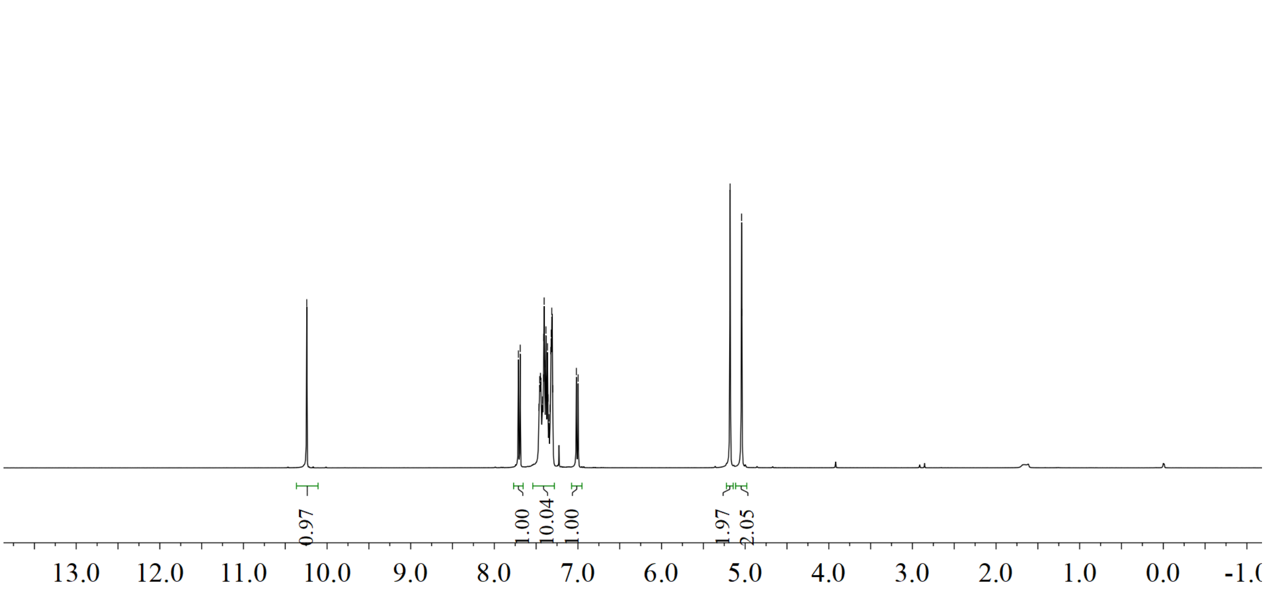

10.243
7.712
7.690
7.466
7.457
7.448
7.442
7.427
7.423
7.413
7.409
7.404
7.398
7.383
7.369
7.364
7.358
7.347
7.331
7.324
7.320
7.313
7.307
7.302
7.019
6.997
5.180
5.043
5.039
0.97
1.00
10.04
1.00
1.97
2.05
13.0 12.0 11.0 10.0 9.0 8.0 7.0 6.0 5.0 4.0 3.0 2.0 1.0 0.0 -1.(

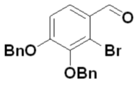

Xu10-2

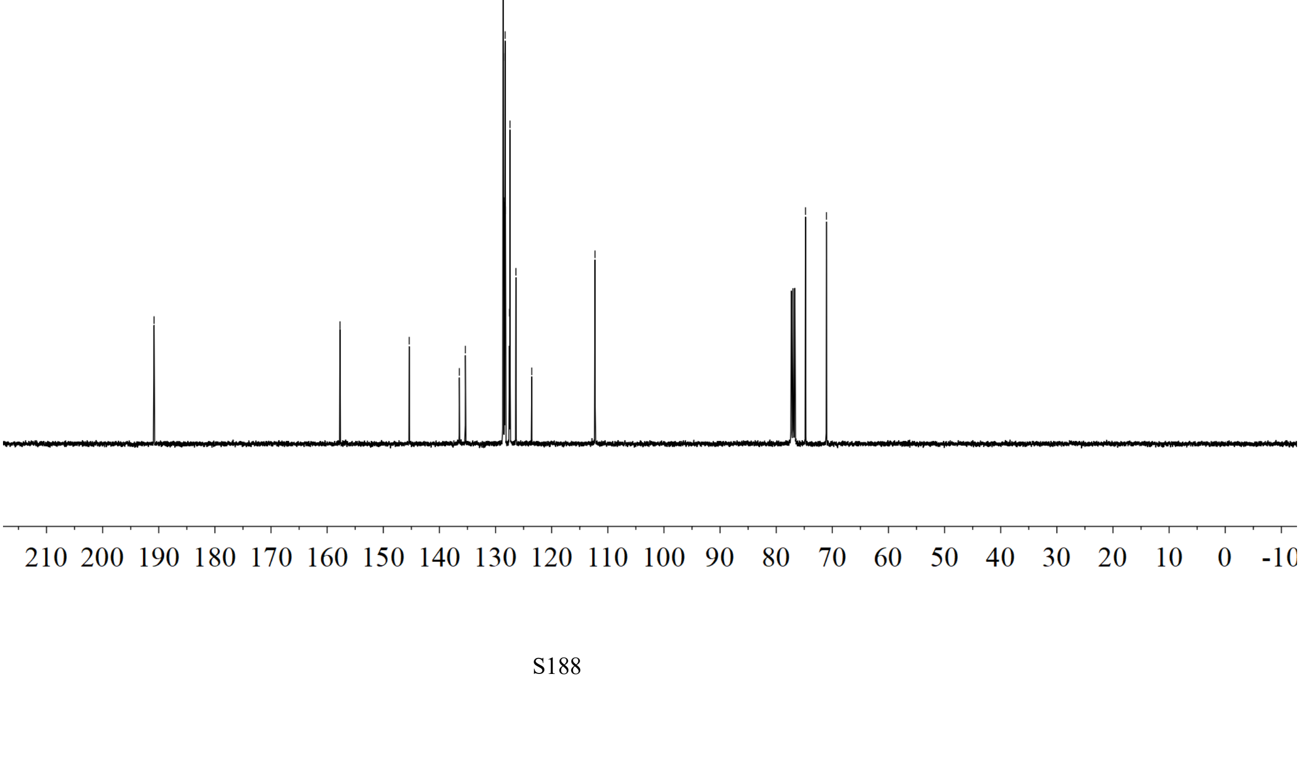

190.838
157.707
145.386
136.452
135.372
128.669
128.581
128.400
128.290
128.227
127.516
127.426
126.372
123.551
112.283
74.781
71.052
210 200 190 180 170 160 150 140 130 120 110 100 90 80 70 60 50 40 30 20 10 0 -10

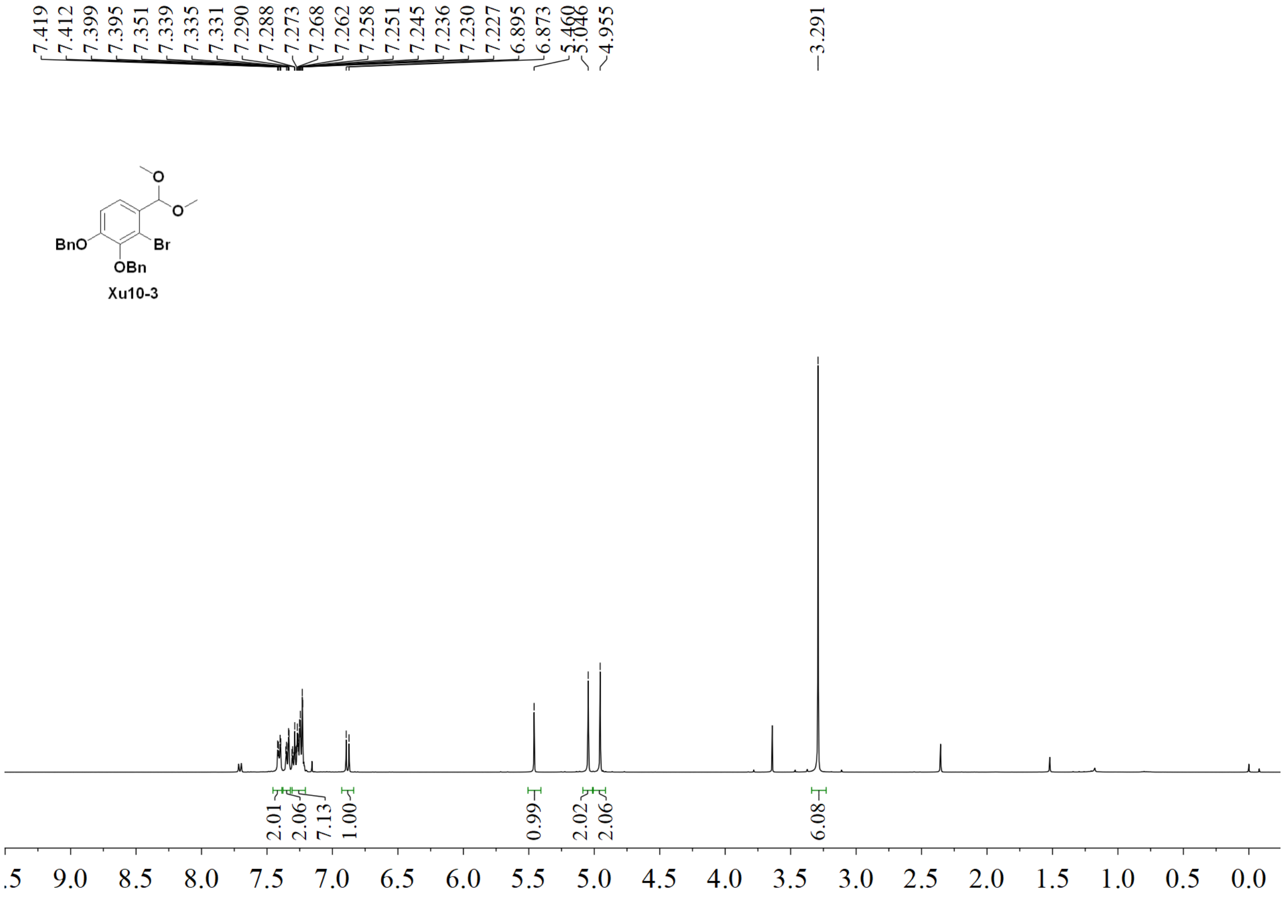
7.419
7.412
7.399
7.395
7.351
7.339
7.335
7.331
7.290
7.288
7.273
7.268
7.262
7.258
7.251
7.245
7.236
7.230
7.227
6.895
6.873
5.460
5.046
4.955
3.291
BnO
Br
OBn
Xu10-3
2.01
2.06
7.13
1.00
0.99
2.02
2.06
6.08
.5 9.0 8.5 8.0 7.5 7.0 6.5 6.0 5.5 5.0 4.5 4.0 3.5 3.0 2.5 2.0 1.5 1.0 0.5 0.0

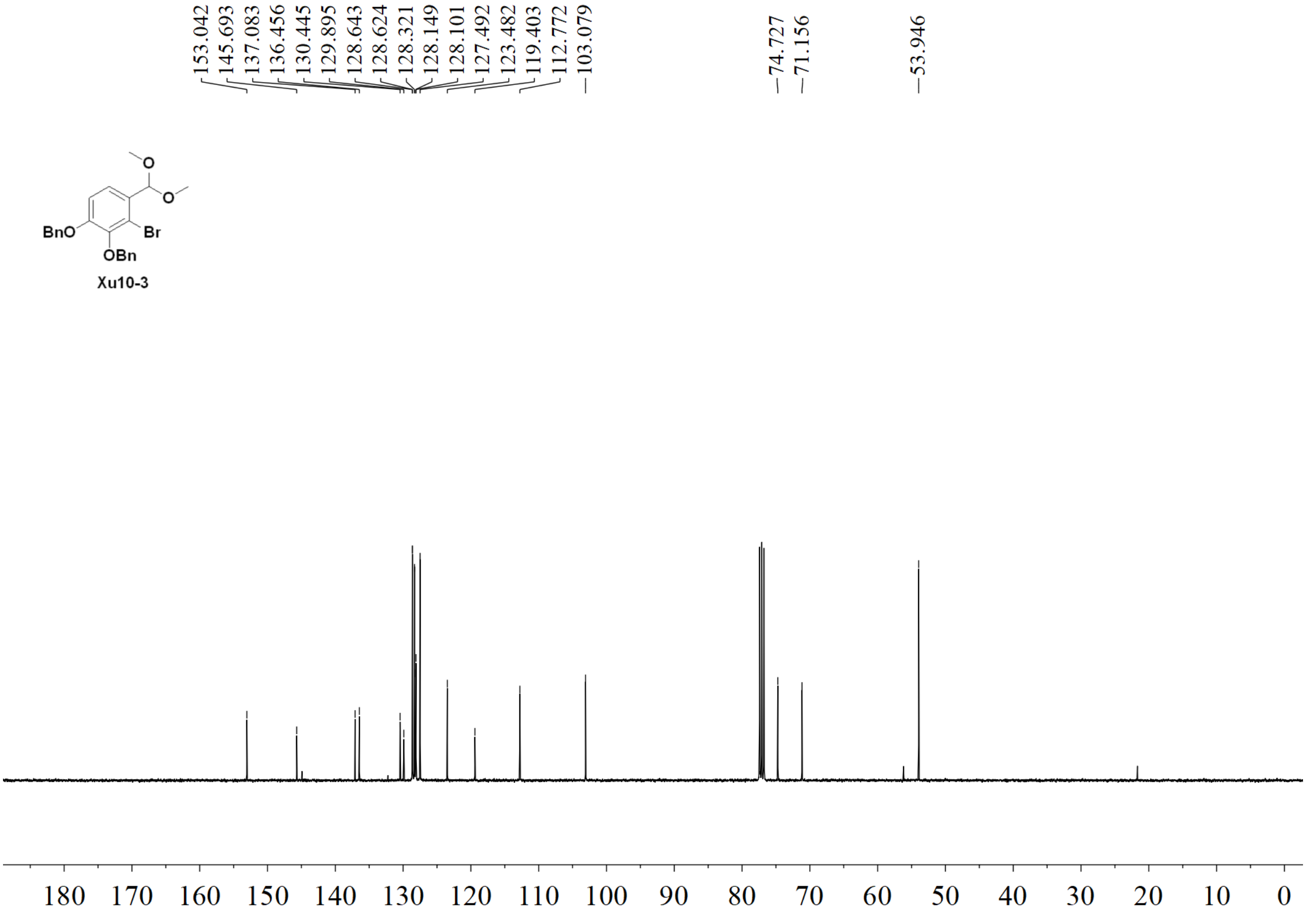
153.042
145.693
137.083
136.456
130.445
129.895
128.643
128.624
128.321
128.149
128.101
127.492
123.482
119.403
112.772
103.079
74.727
71.156
53.946
BnO
Br
OBn
Xu10-3
180 170 160 150 140 130 120 110 100 90 80 70 60 50 40 30 20 10 0

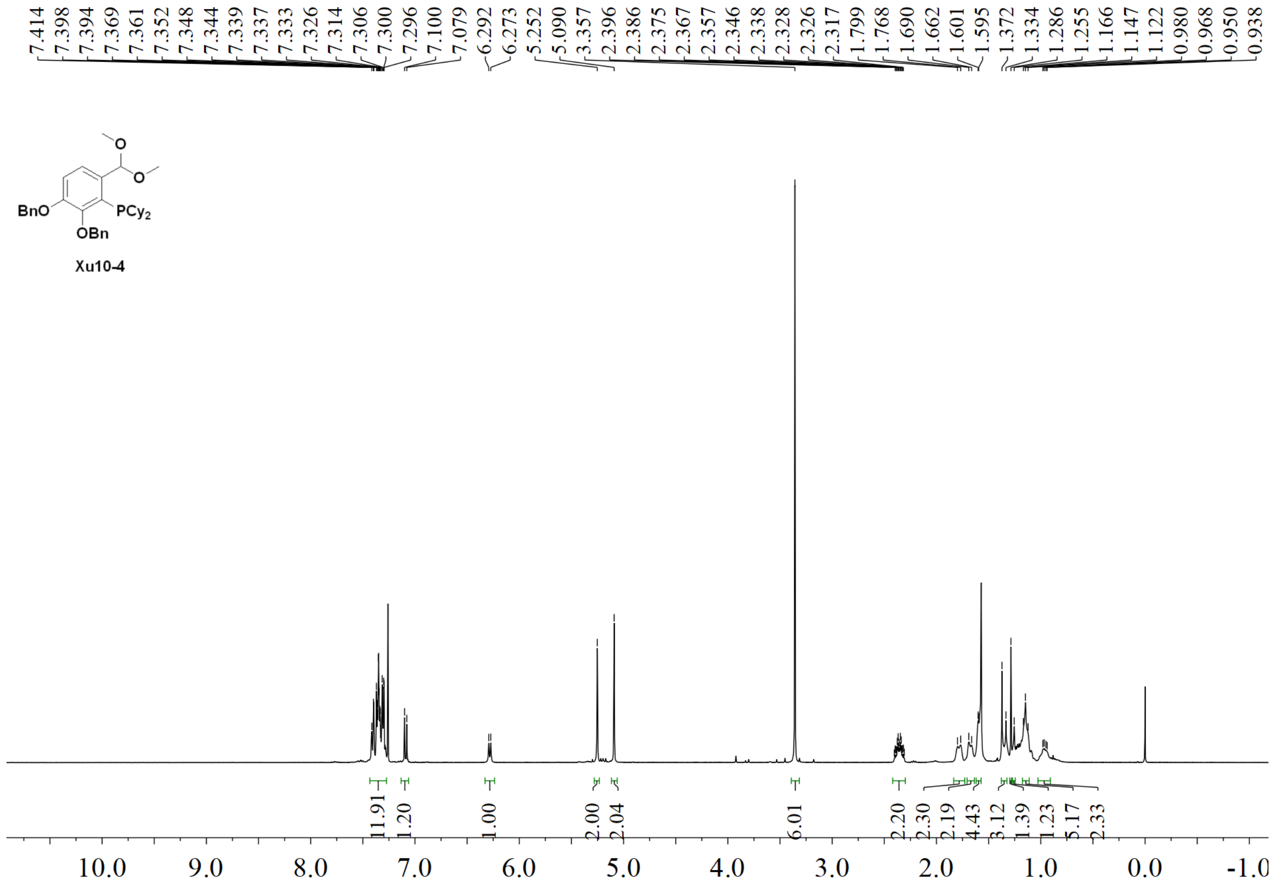
7.414 7.398 7.394 7.369 7.361 7.352 7.348 7.344 7.339 7.337 7.333 7.326 7.314 7.306 7.300 7.296 7.100 7.079 6.292 6.273 5.252 5.090 3.357 2.396 2.386 2.375 2.367 2.357 2.346 2.338 2.328 2.326 2.317 1.799 1.768 1.690 1.662 1.601 1.595 1.372 1.334 1.286 1.255 1.166 1.147 1.122 0.980 0.968 0.950 0.938
BnO
OBn
PCy2
Xu10-4
11.91 1.20 1.00 2.00 2.04 6.01 2.20 2.30 2.19 4.43 3.12 1.39 1.23 5.17 2.33
10.0 9.0 8.0 7.0 6.0 5.0 4.0 3.0 2.0 1.0 0.0 -1.0

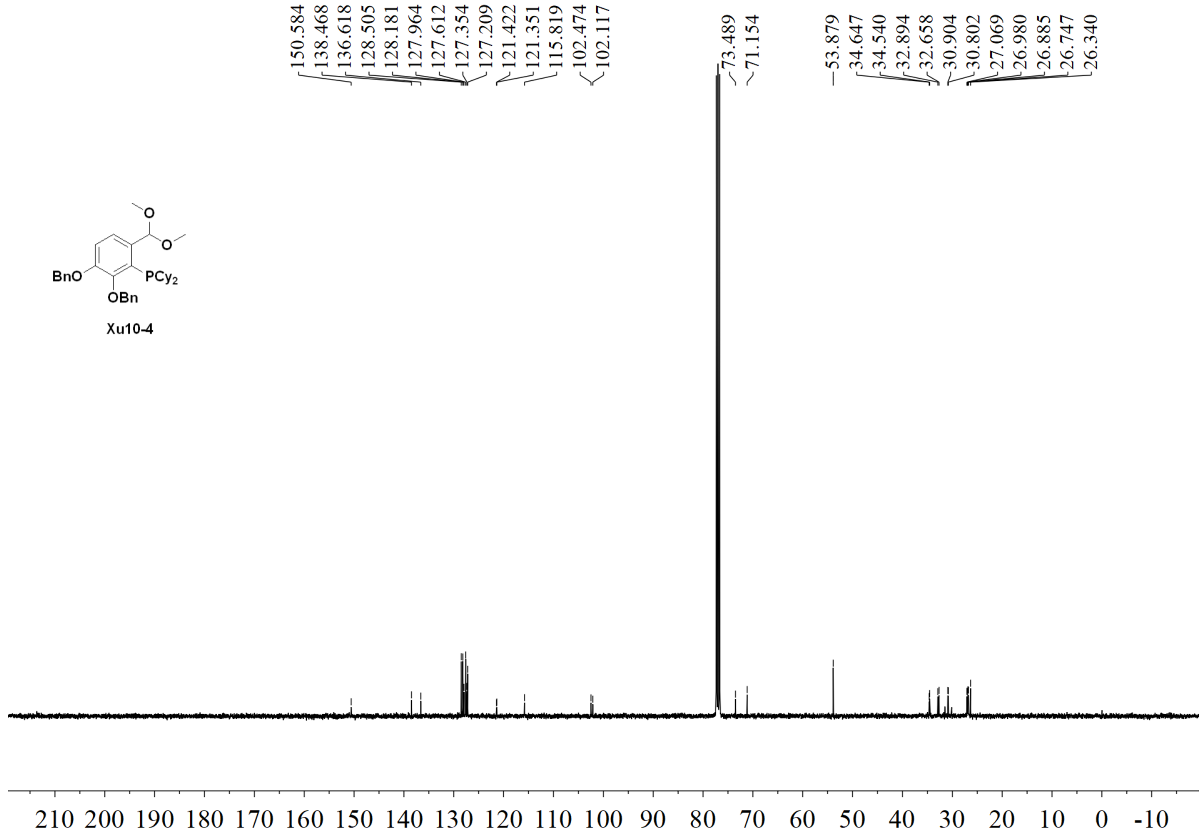
150.584 138.468 136.618 128.505 128.181 127.964 127.612 127.354 127.209 121.422 121.351 115.819 102.474 102.117
73.489 71.154
53.879 34.647 34.540 32.894 32.658 30.904 30.802 27.069 26.980 26.885 26.747 26.340
BnO
OBn
PCy2
Xu10-4
210 200 190 180 170 160 150 140 130 120 110 100 90 80 70 60 50 40 30 20 10 0 -10

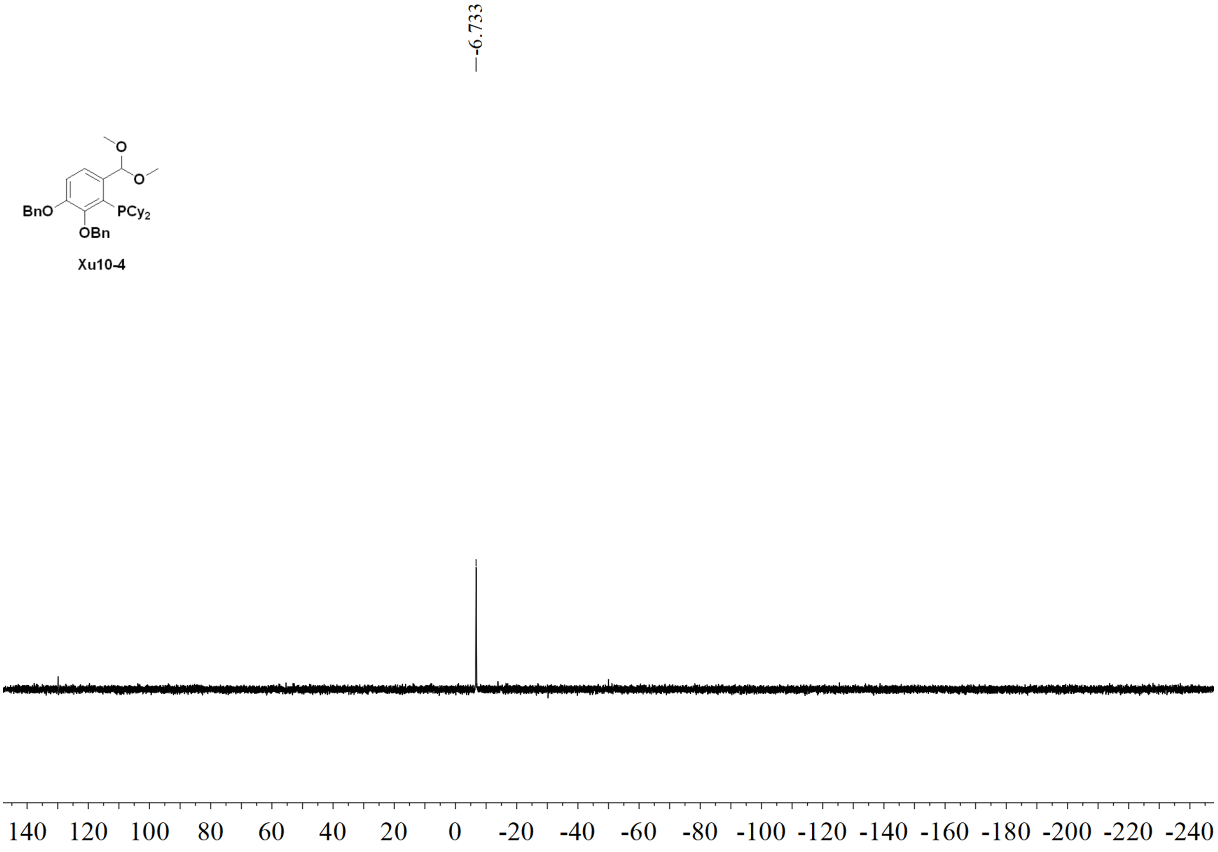

—6.733
BnO
OBn
PCy2
O
O
Xu10-4
140 120 100 80 60 40 20 0 -20 -40 -60 -80 -100 -120 -140 -160 -180 -200 -220 -240

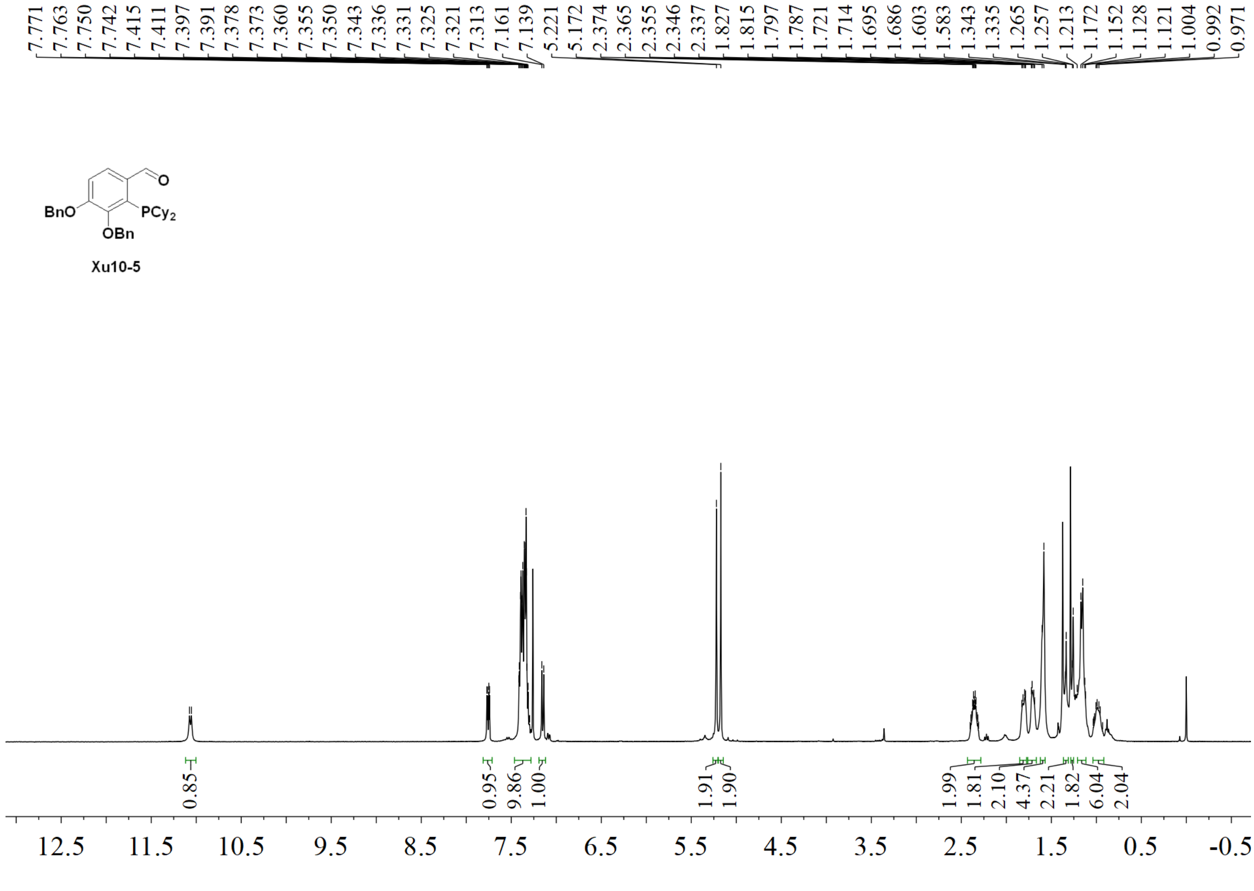

7.771 7.763 7.750 7.742 7.415 7.411 7.397 7.391 7.378 7.373 7.360 7.355 7.350 7.343 7.336 7.331 7.325 7.321 7.313 7.161 7.139 5.221 5.172 2.374 2.365 2.355 2.346 2.337 1.827 1.815 1.797 1.787 1.721 1.714 1.695 1.686 1.603 1.583 1.343 1.335 1.265 1.257 1.213 1.172 1.152 1.128 1.121 1.004 0.992 0.971
BnO
OBn
PCy2
O
Xu10-5
0.85 0.95 9.86 1.00 1.91 1.90 1.99 1.81 2.10 4.37 2.21 1.82 6.04 2.04
12.5 11.5 10.5 9.5 8.5 7.5 6.5 5.5 4.5 3.5 2.5 1.5 0.5 -0.5

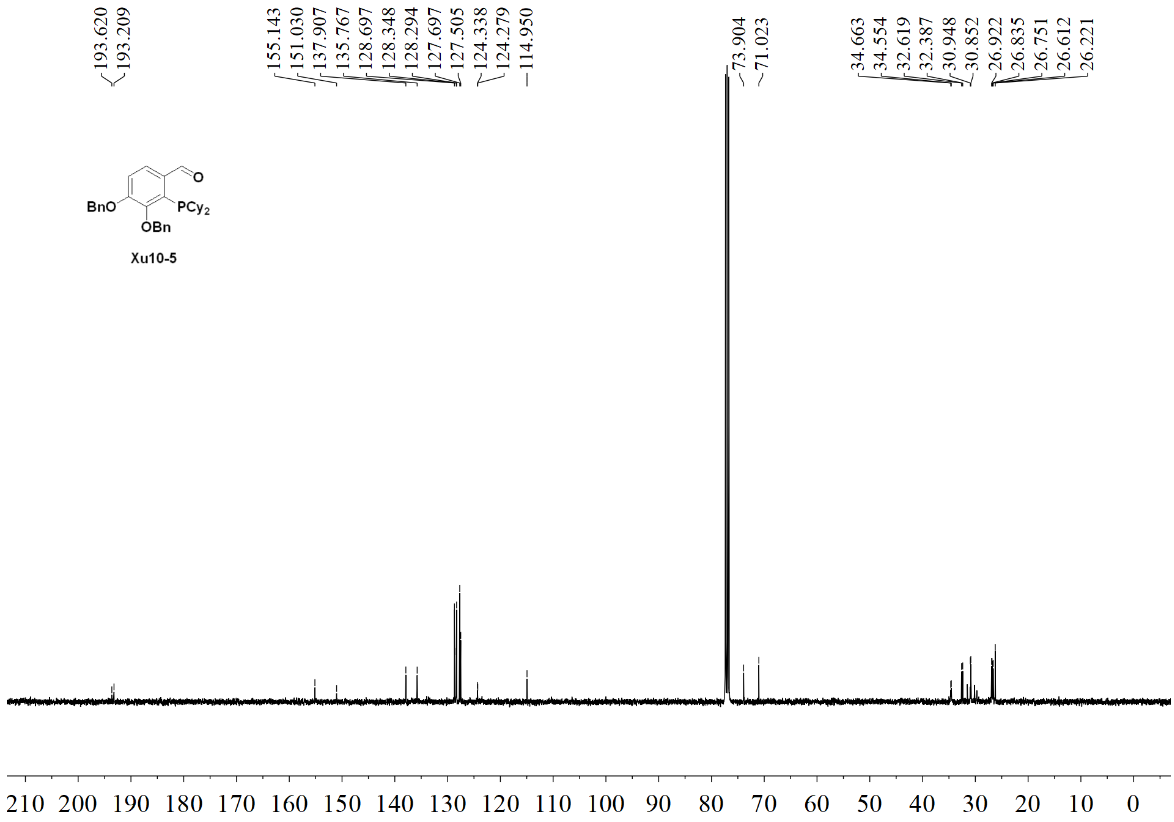
193.620
193.209
155.143
151.030
137.907
135.767
128.697
128.348
128.294
127.697
127.505
124.338
124.279
114.950
73.904
71.023
34.663
34.554
32.619
32.387
30.948
30.852
26.922
26.835
26.751
26.612
26.221
BnO
PCy2
OBn
Xu10-5
210 200 190 180 170 160 150 140 130 120 110 100 90 80 70 60 50 40 30 20 10 0

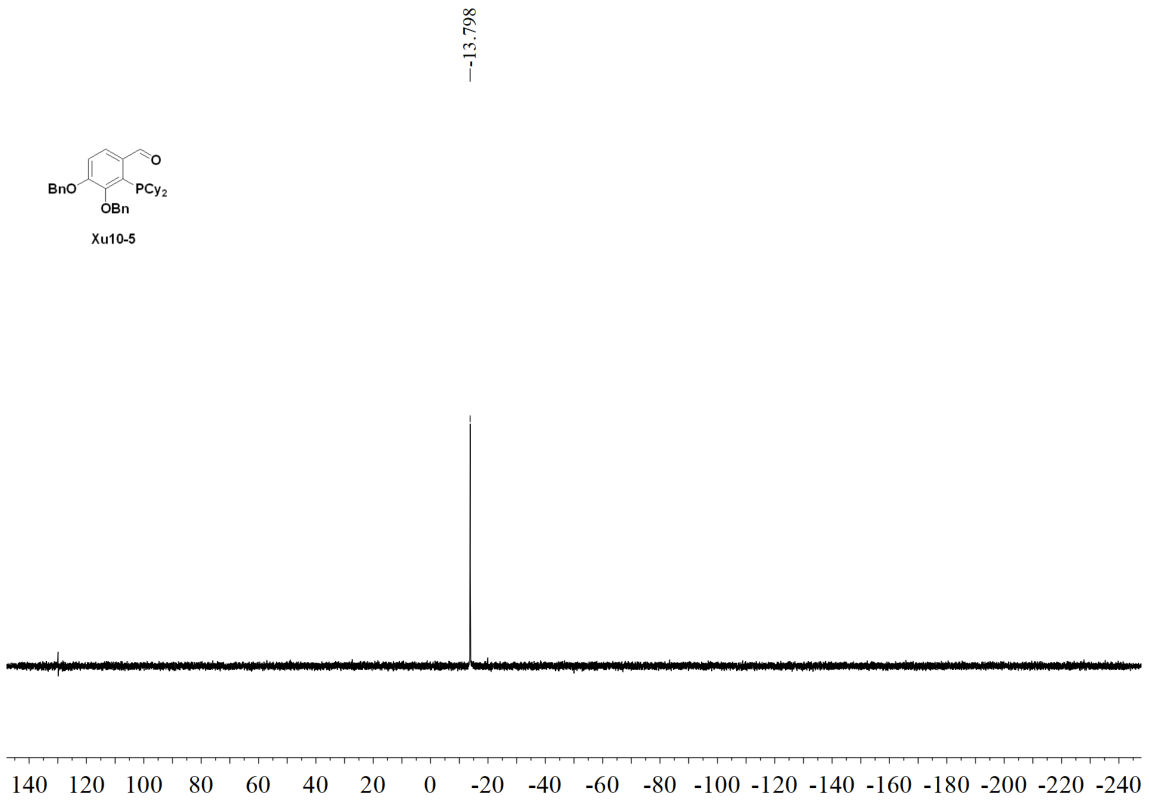
-13.798
BnO
PCy2
OBn
Xu10-5
140 120 100 80 60 40 20 0 -20 -40 -60 -80 -100 -120 -140 -160 -180 -200 -220 -240

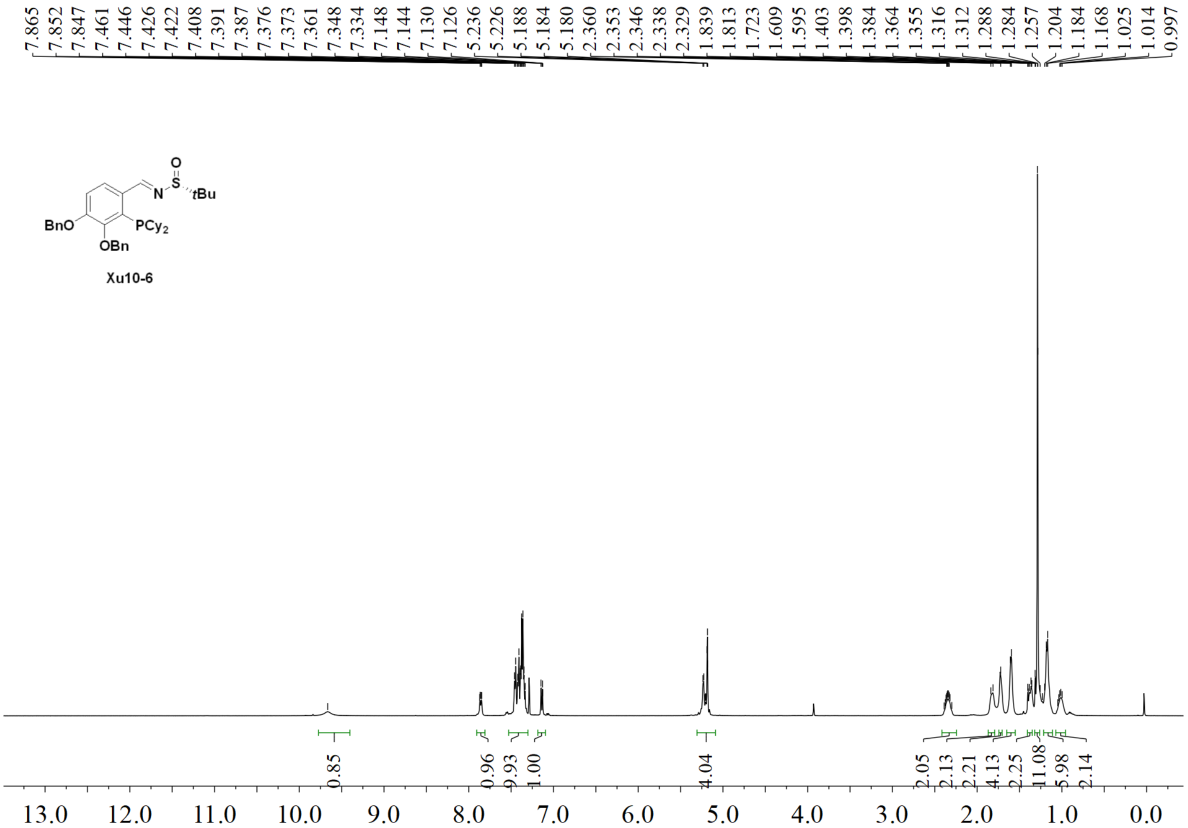
7.865 7.852 7.847 7.461 7.446 7.426 7.422 7.408 7.391 7.387 7.376 7.373 7.361 7.348 7.334 7.148 7.144 7.130 7.126 5.236 5.226 5.188 5.184 5.180 2.360 2.353 2.346 2.338 2.329 1.839 1.813 1.723 1.609 1.595 1.403 1.398 1.384 1.364 1.355 1.316 1.312 1.288 1.284 1.257 1.204 1.184 1.168 1.025 1.014 0.997
O
S
N
tBu
BnO
PCy2
OBn
Xu10-6
0.85
0.96
9.93
1.00
4.04
2.05
2.13
2.21
4.13
2.25
11.08
5.98
2.14
13.0 12.0 11.0 10.0 9.0 8.0 7.0 6.0 5.0 4.0 3.0 2.0 1.0 0.0

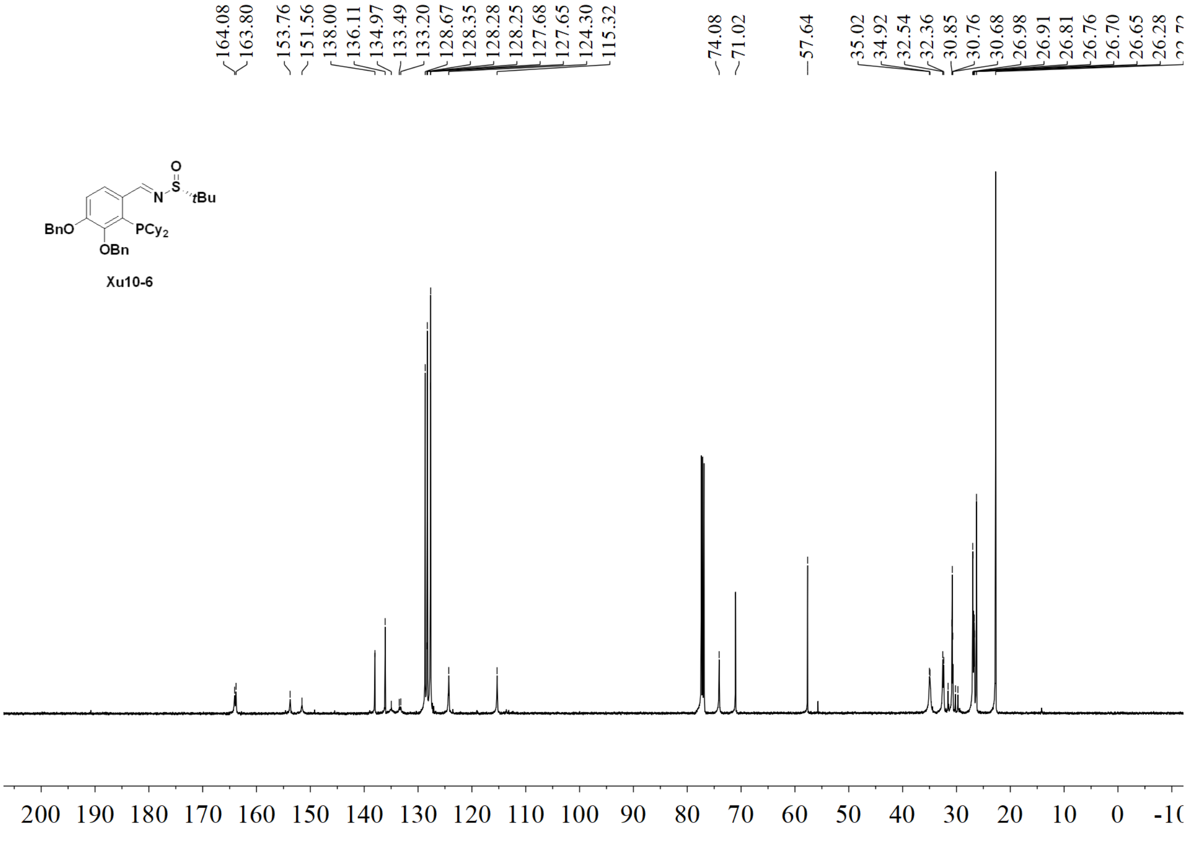
164.08 163.80 153.76 151.56 138.00 136.11 134.97 133.49 133.20 128.67 128.35 128.28 128.25 127.68 127.65 124.30 115.32
74.08 71.02
57.64
35.02 34.92 32.54 32.36 30.85 30.76 30.68 26.98 26.91 26.81 26.76 26.70 26.65 26.28
O
S
N
tBu
BnO
PCy2
OBn
Xu10-6
200 190 180 170 160 150 140 130 120 110 100 90 80 70 60 50 40 30 20 10 0

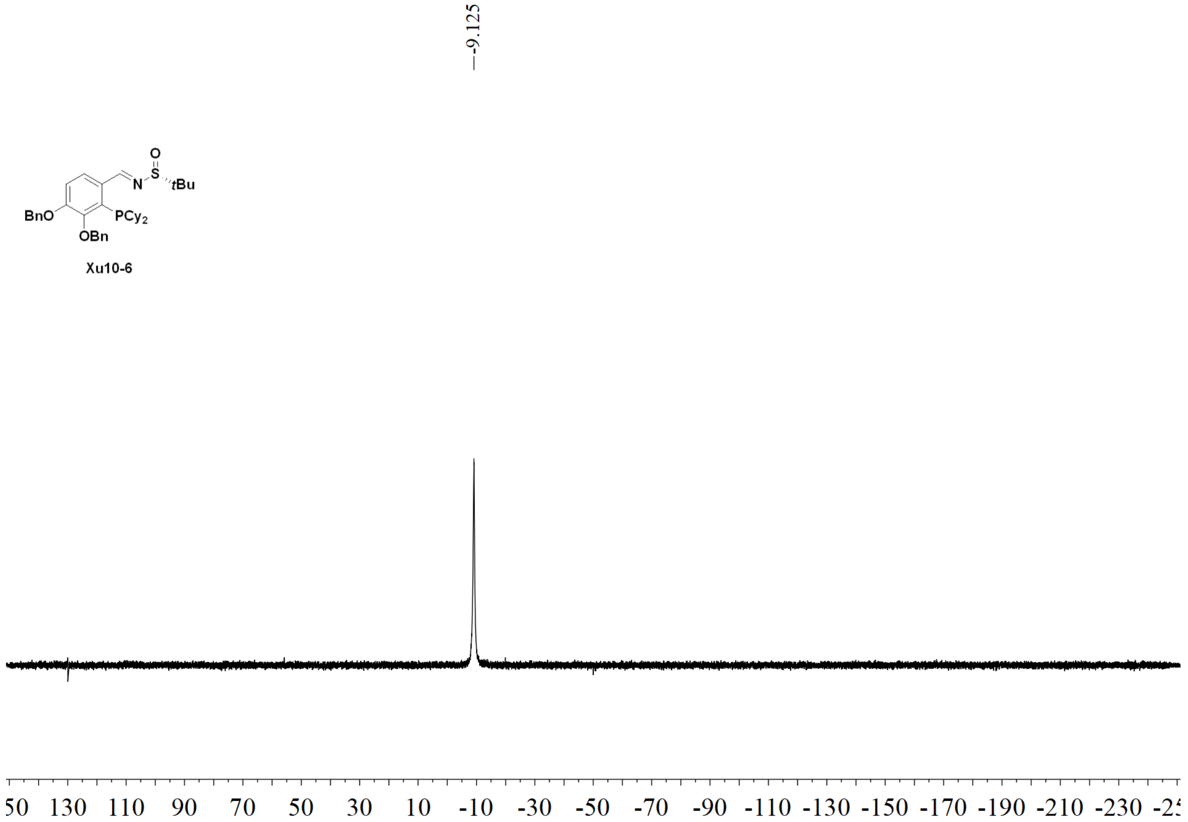

—9.125
BnO
OBn
PCy2
N
S
O
tBu
50 130 110 90 70 50 30 10 -10 -30 -50 -70 -90 -110 -130 -150 -170 -190 -210 -230

Xu10-6

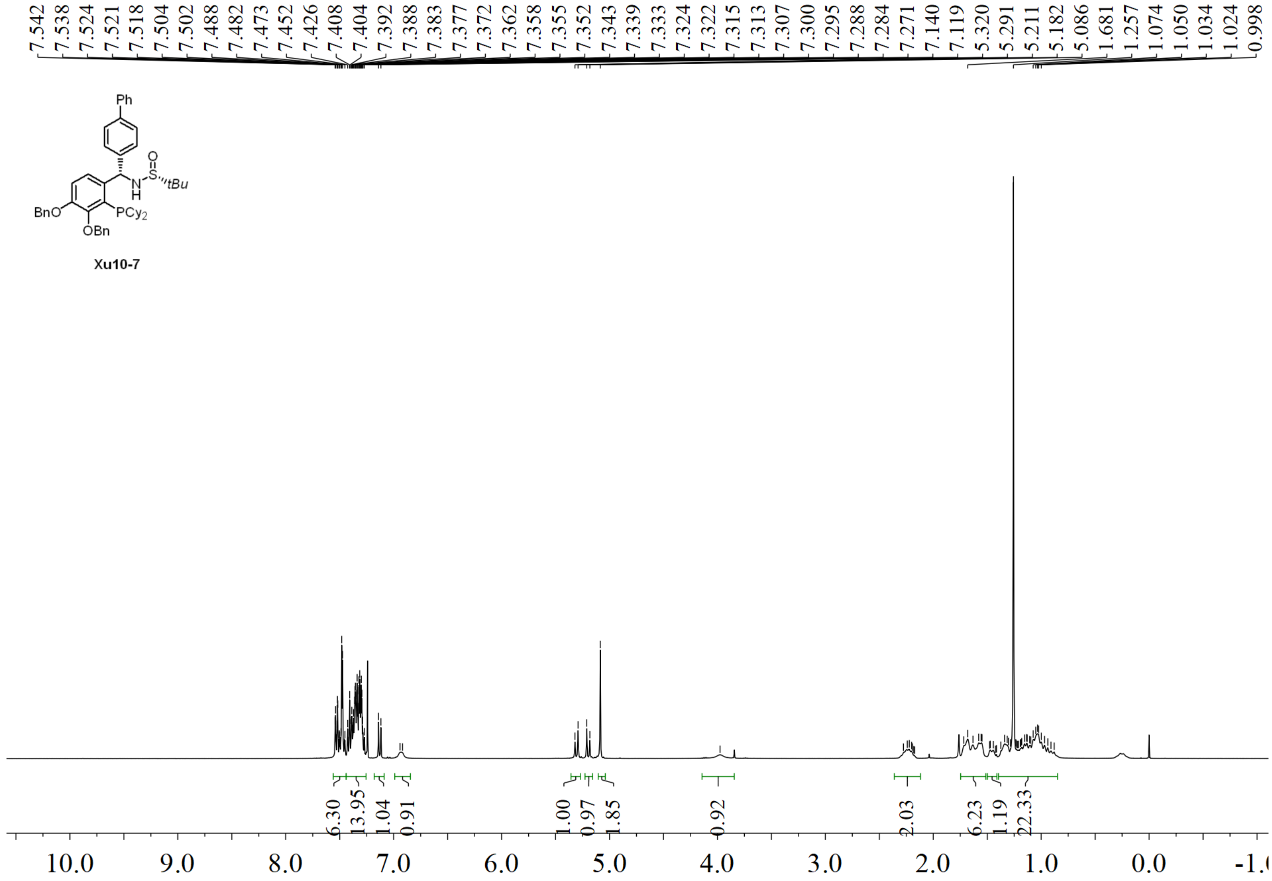

7.542 7.538 7.524 7.521 7.518 7.504 7.502 7.488 7.482 7.473 7.452 7.426 7.408 7.404 7.392 7.388 7.383 7.377 7.372 7.362 7.358 7.355 7.352 7.343 7.339 7.333 7.324 7.322 7.315 7.313 7.307 7.300 7.295 7.288 7.284 7.271 7.140 7.119 5.320 5.291 5.211 5.182 5.086 1.681 1.257 1.074 1.050 1.034 1.024 0.998
Ph
BnO
OBn
PCy2
N
H
S
O
tBu
6.30 13.95 1.04 0.91 1.00 0.97 1.85 0.92 2.03 6.23 1.19 22.33
10.0 9.0 8.0 7.0 6.0 5.0 4.0 3.0 2.0 1.0 0.0

Xu10-7

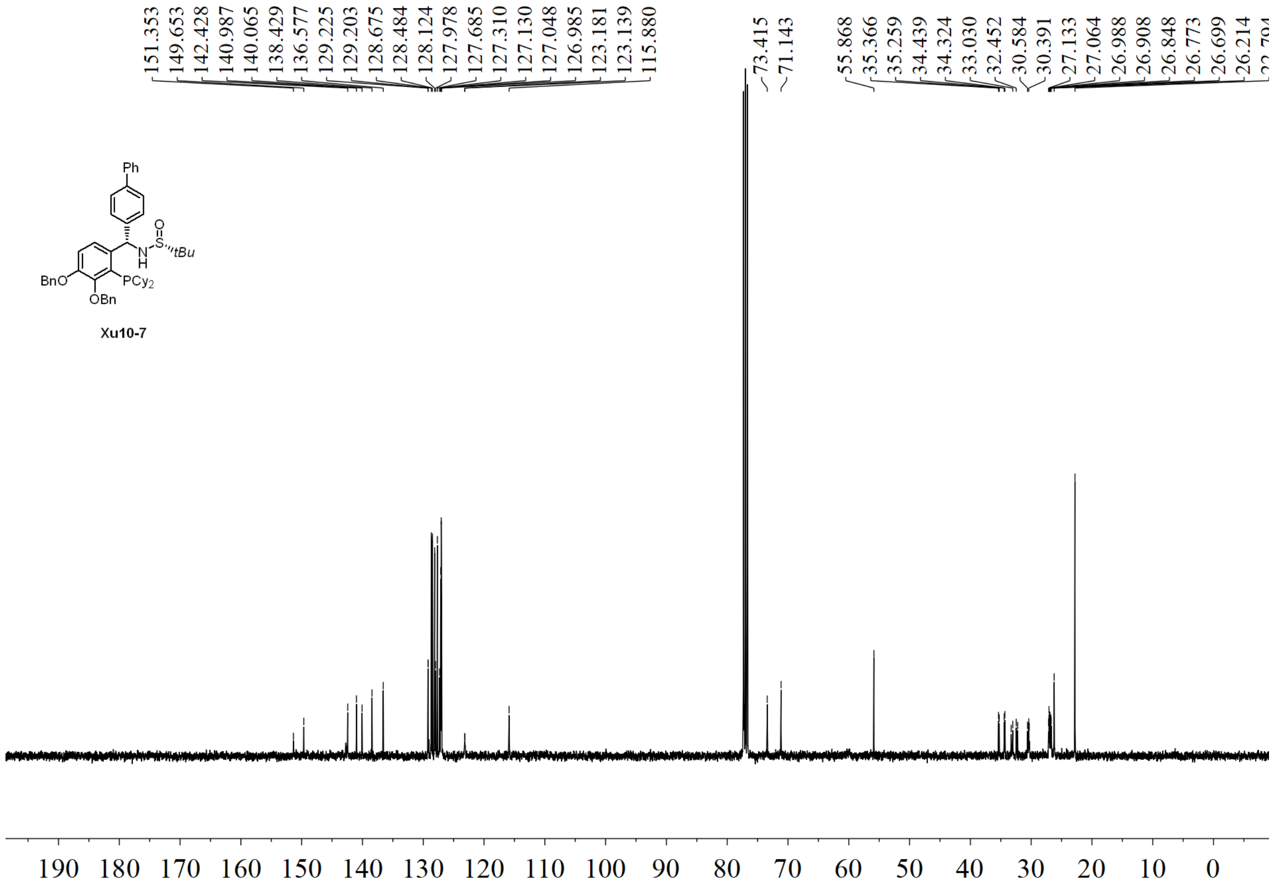
151.353
149.653
142.428
140.987
140.065
138.429
136.577
129.225
129.203
128.675
128.484
128.124
127.978
127.685
127.310
127.130
127.048
126.985
123.181
123.139
115.880
73.415
71.143
55.868
35.366
35.259
34.439
34.324
33.030
32.452
30.584
30.391
27.133
27.064
26.988
26.908
26.848
26.773
26.699
26.214
Ph
O
S
N
H
tBu
BnO
PCy2
OBn
Xu10-7
190 180 170 160 150 140 130 120 110 100 90 80 70 60 50 40 30 20 10 0

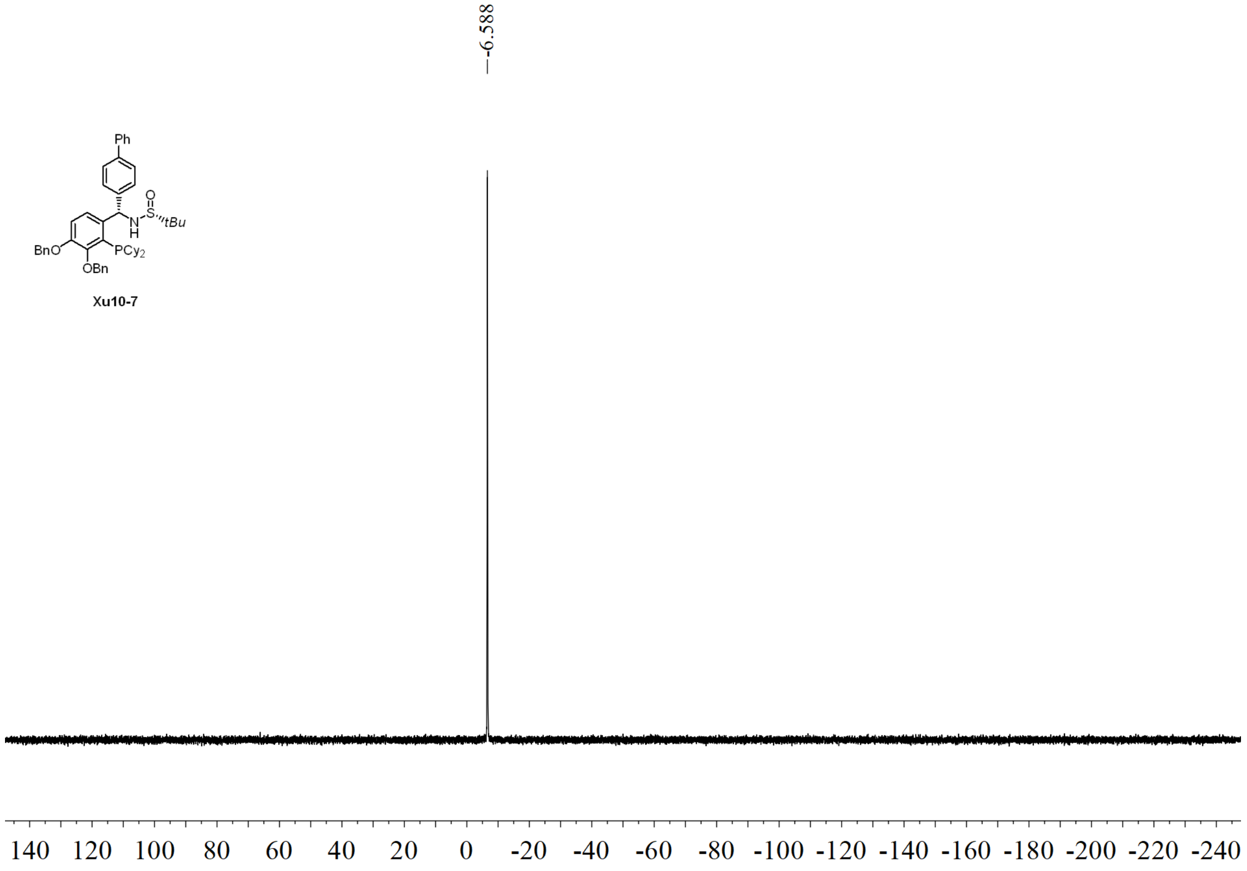
-6.588
Ph
O
S
N
H
tBu
BnO
PCy2
OBn
Xu10-7
140 120 100 80 60 40 20 0 -20 -40 -60 -80 -100 -120 -140 -160 -180 -200 -220 -240

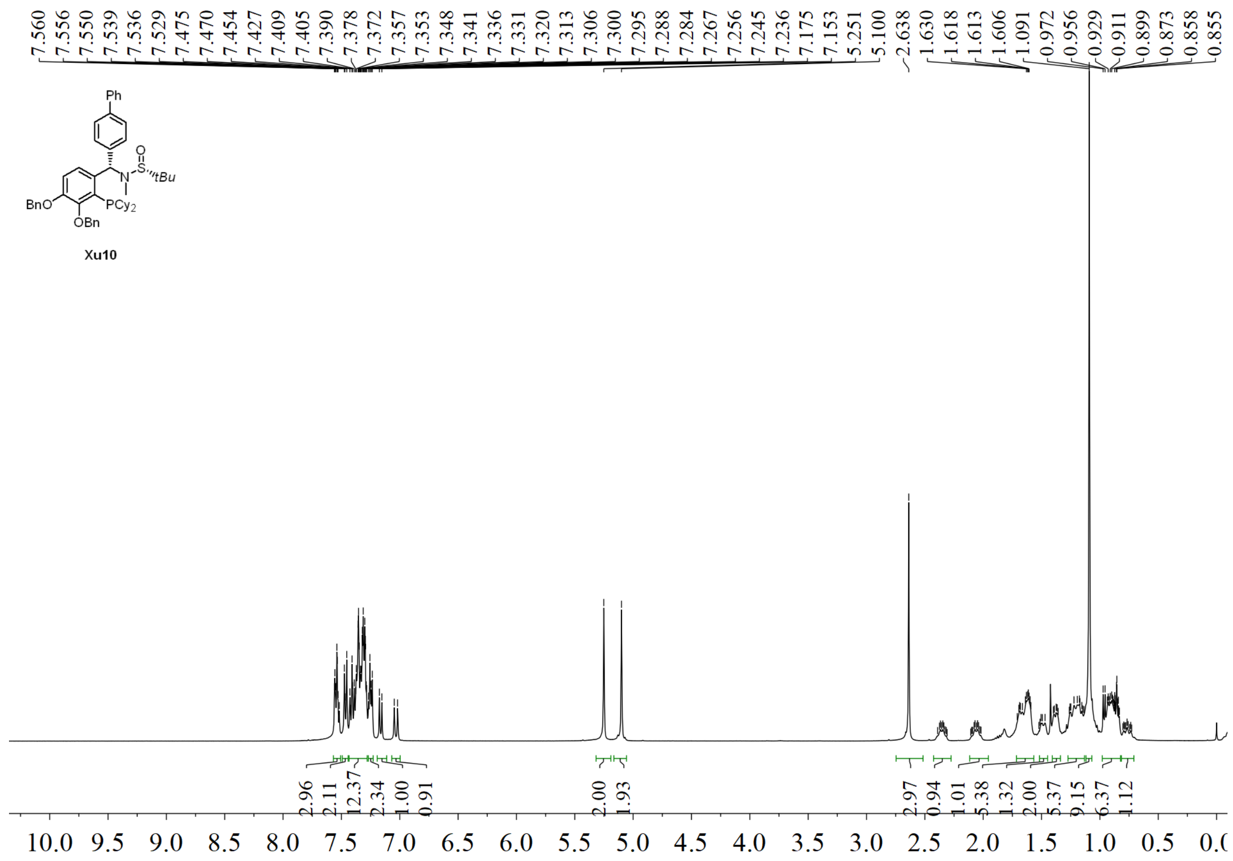
7.560 7.556 7.550 7.539 7.536 7.529 7.475 7.470 7.454 7.427 7.409 7.405 7.390 7.378 7.372 7.357 7.353 7.348 7.341 7.336 7.331 7.320 7.313 7.306 7.300 7.295 7.288 7.284 7.267 7.256 7.245 7.236 7.175 7.153 5.251 5.100 2.638 1.630 1.618 1.613 1.606 1.091 0.972 0.956 0.929 0.911 0.899 0.873 0.858 0.855
Ph
O
S
N
tBu
BnO
OBn
PCy2
Xu10
2.96 2.11 12.37 2.34 1.00 0.91 2.00 1.93 2.97 0.94 1.01 5.38 1.32 2.00 5.37 9.15 6.37 1.12
10.0 9.5 9.0 8.5 8.0 7.5 7.0 6.5 6.0 5.5 5.0 4.5 4.0 3.5 3.0 2.5 2.0 1.5 1.0 0.5 0.(

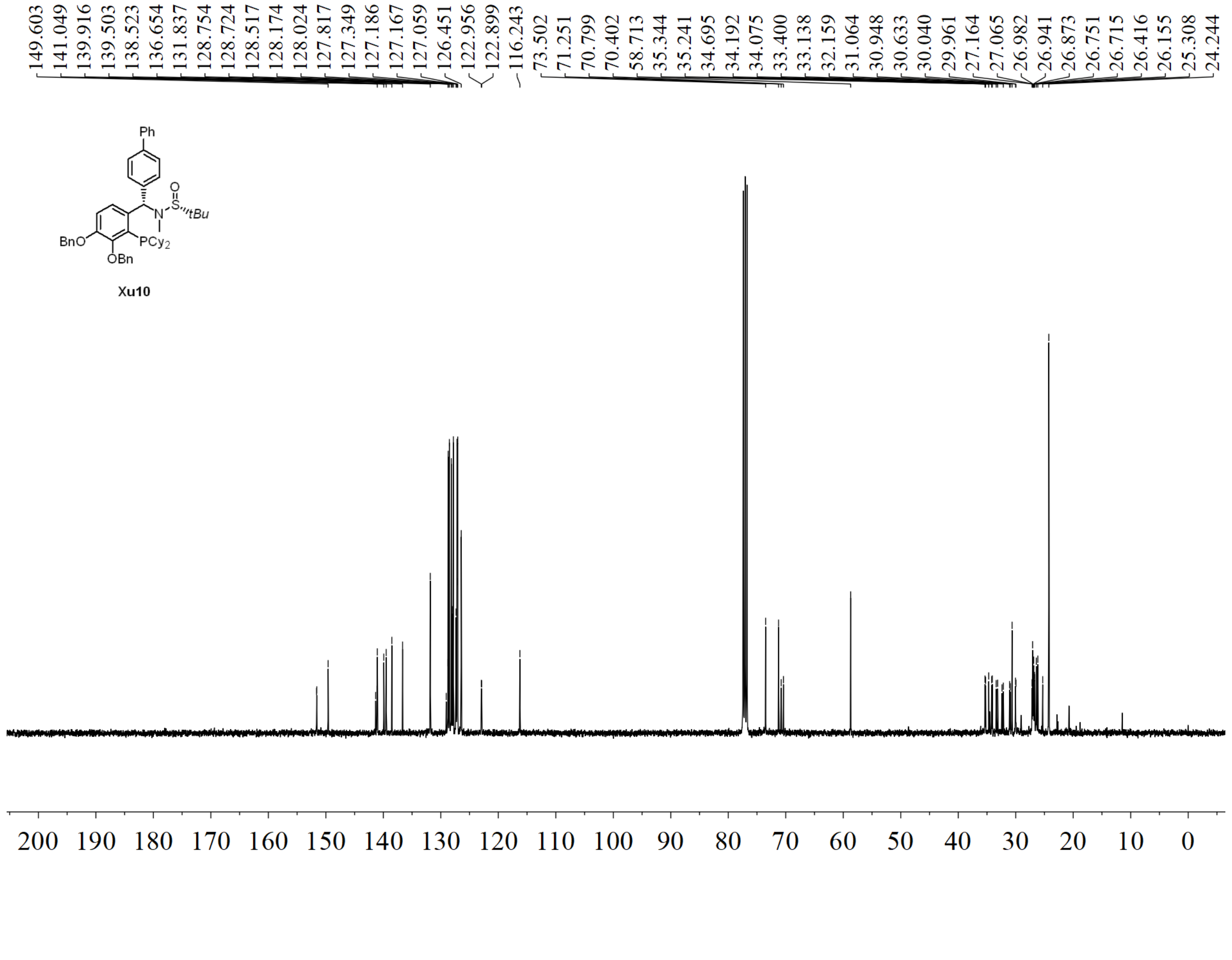
149.603 141.049 139.916 139.503 138.523 136.654 131.837 128.754 128.724 128.517 128.174 128.024 127.817 127.349 127.186 127.167 127.059 126.451 122.956 122.899 116.243 73.502 71.251 70.799 70.402 58.713 35.344 35.241 34.695 34.192 34.075 33.400 33.138 32.159 31.064 30.948 30.633 30.040 29.961 27.164 27.065 26.982 26.941 26.873 26.751 26.715 26.416 26.155 25.308 24.244
Ph
O
S
N
tBu
BnO
OBn
PCy2
Xu10
200 190 180 170 160 150 140 130 120 110 100 90 80 70 60 50 40 30 20 10 0

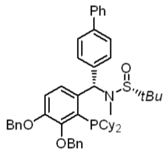
Ph
O
S
N
tBu
BnO
PCy2
OBn

Xu10

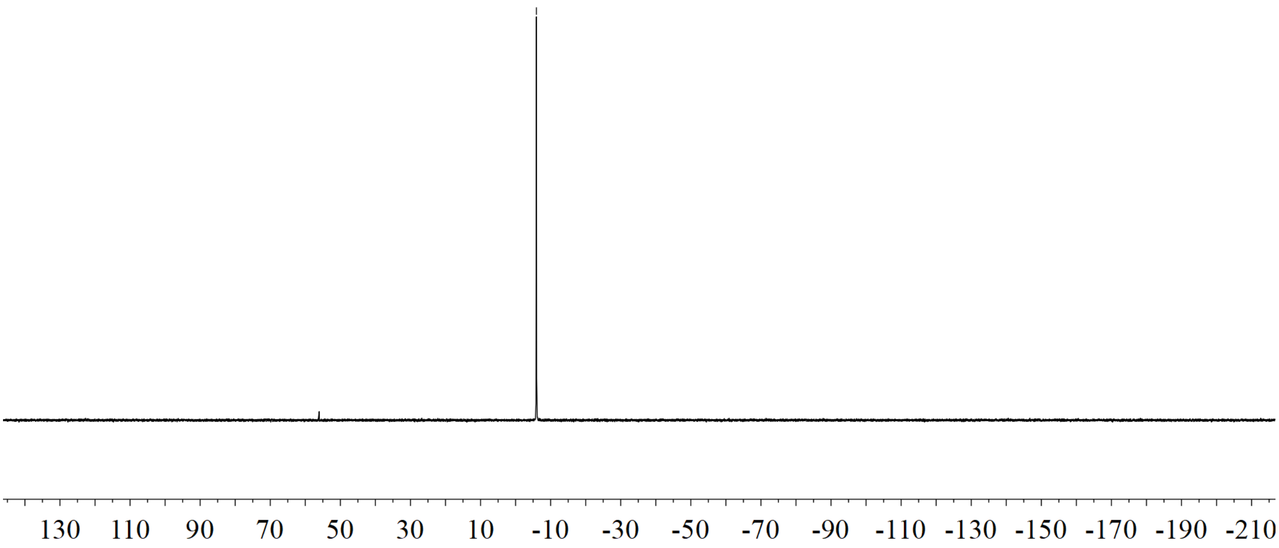
-5.942
130
110
90
70
50
30
10
-10
-30
-50
-70
-90
-110
-130
-150
-170
-190
-210

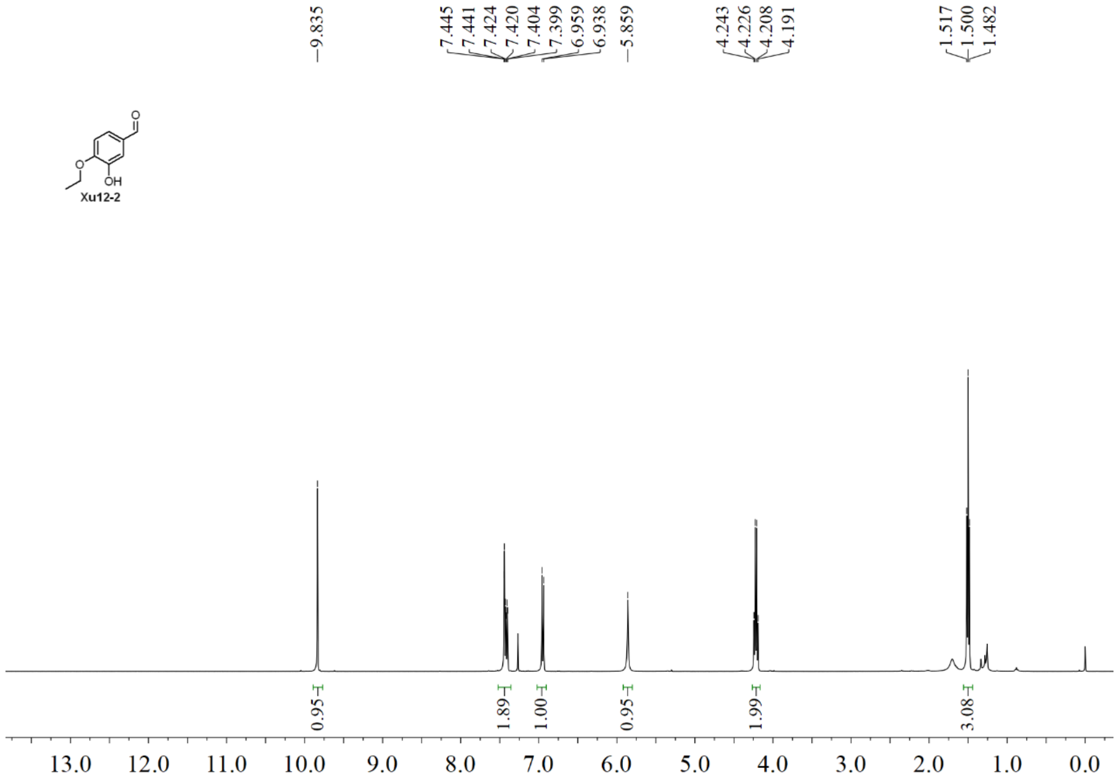
9.835
7.445
7.441
7.424
7.420
7.404
7.399
6.959
6.938
5.859
4.243
4.226
4.208
4.191
1.517
1.500
1.482
OH
Xu12-2
0.95
1.89
1.00
0.95
1.99
3.08
13.0 12.0 11.0 10.0 9.0 8.0 7.0 6.0 5.0 4.0 3.0 2.0 1.0 0.0

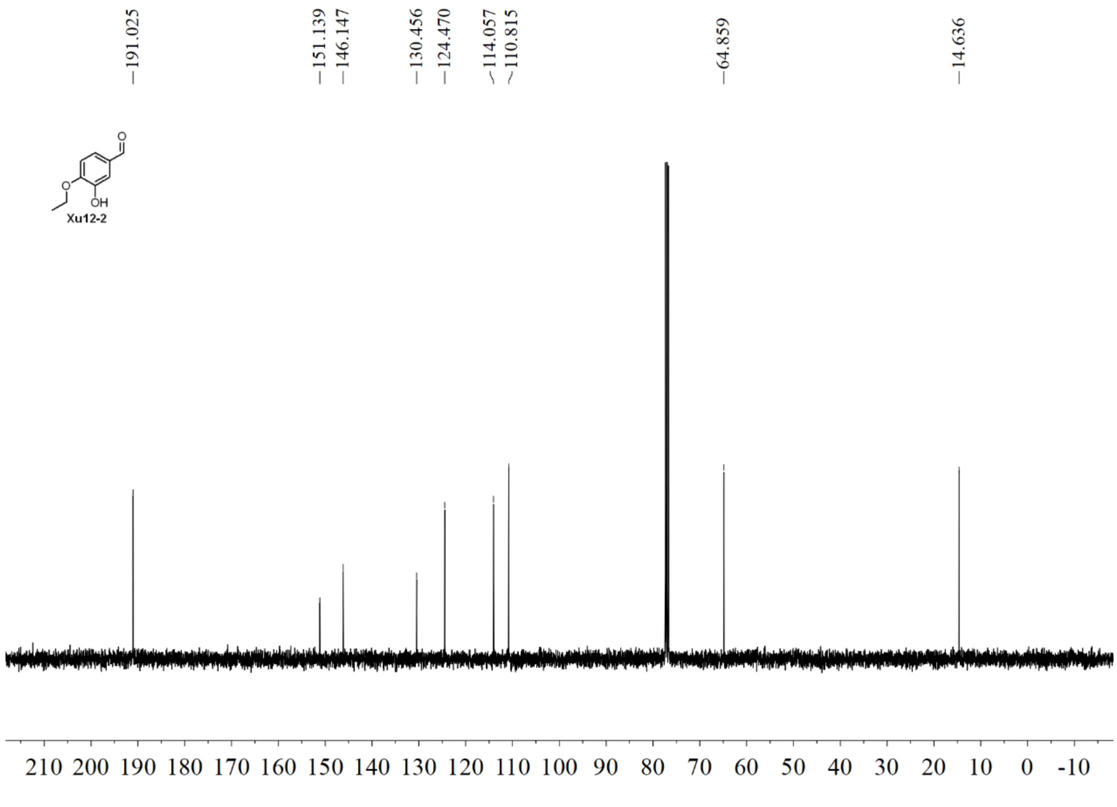
191.025
151.139
146.147
130.456
124.470
114.057
110.815
64.859
14.636
OH
Xu12-2
210 200 190 180 170 160 150 140 130 120 110 100 90 80 70 60 50 40 30 20 10 0 -10

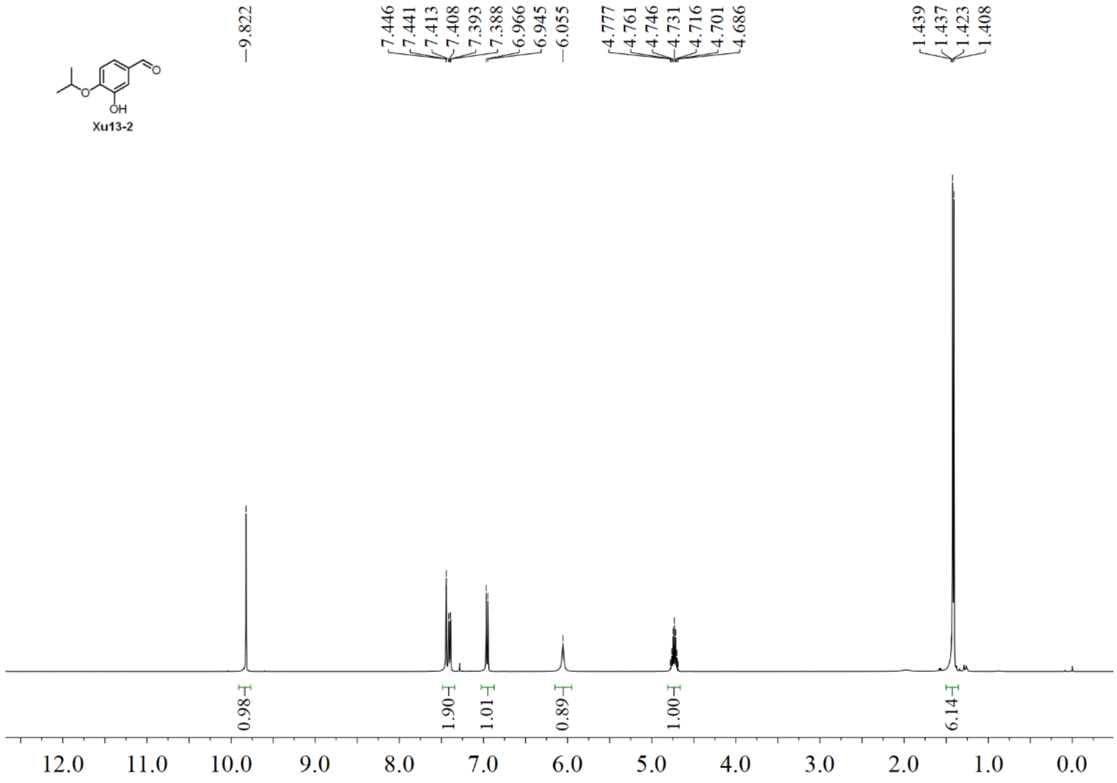
OH
Xu13-2
9.822
7.446
7.441
7.413
7.408
7.393
7.388
6.966
6.945
6.055
4.777
4.761
4.746
4.731
4.716
4.701
4.686
1.439
1.437
1.423
1.408
0.98
1.90
1.01
0.89
1.00
6.14
12.0 11.0 10.0 9.0 8.0 7.0 6.0 5.0 4.0 3.0 2.0 1.0 0.0

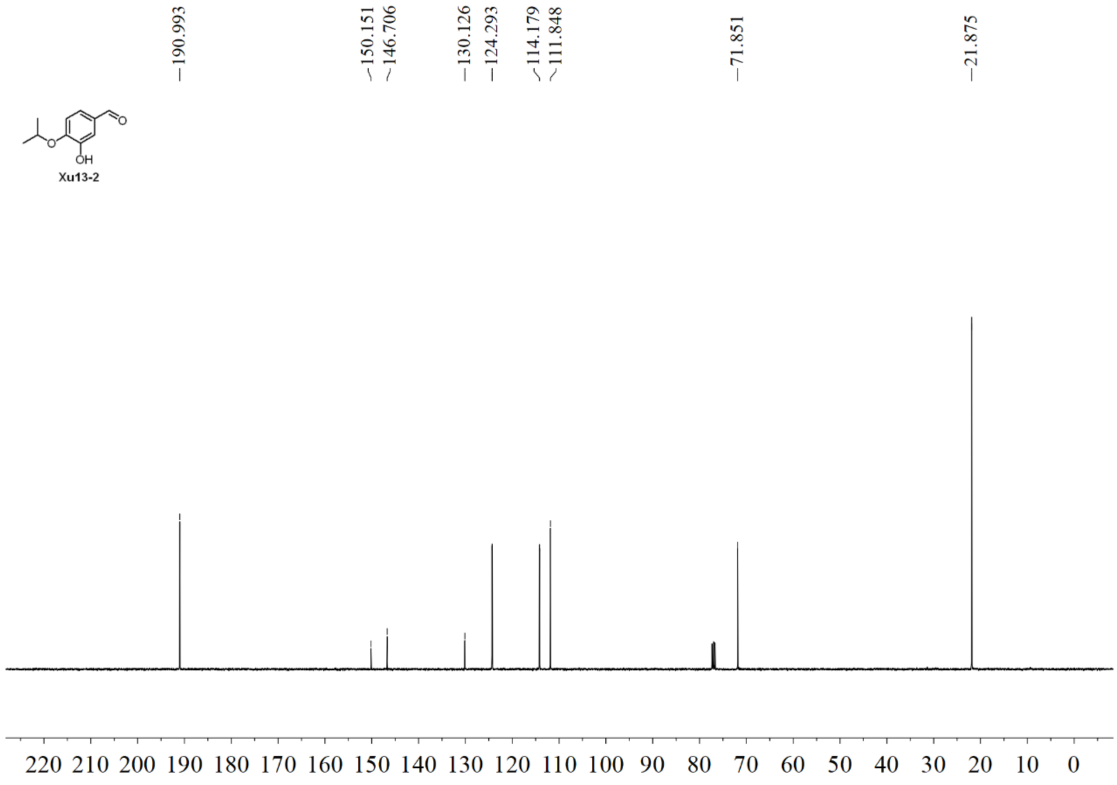
190.993
150.151
146.706
130.126
124.293
114.179
111.848
71.851
21.875
OH
Xu13-2
220 210 200 190 180 170 160 150 140 130 120 110 100 90 80 70 60 50 40 30 20 10 0

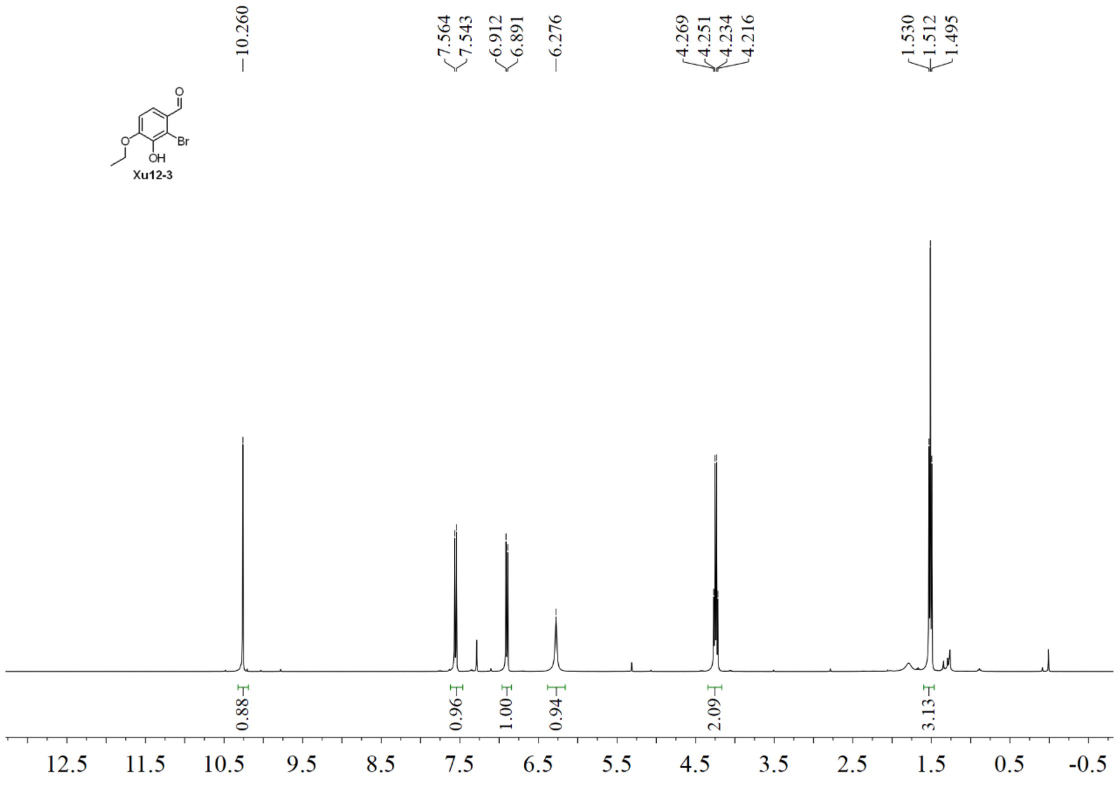

10.260
7.564
7.543
6.912
6.891
6.276
4.269
4.251
4.234
4.216
1.530
1.512
1.495
Br
OH
Xu12-3
0.88
0.96
1.00
0.94
2.09
3.13
12.5 11.5 10.5 9.5 8.5 7.5 6.5 5.5 4.5 3.5 2.5 1.5 0.5 -0.5

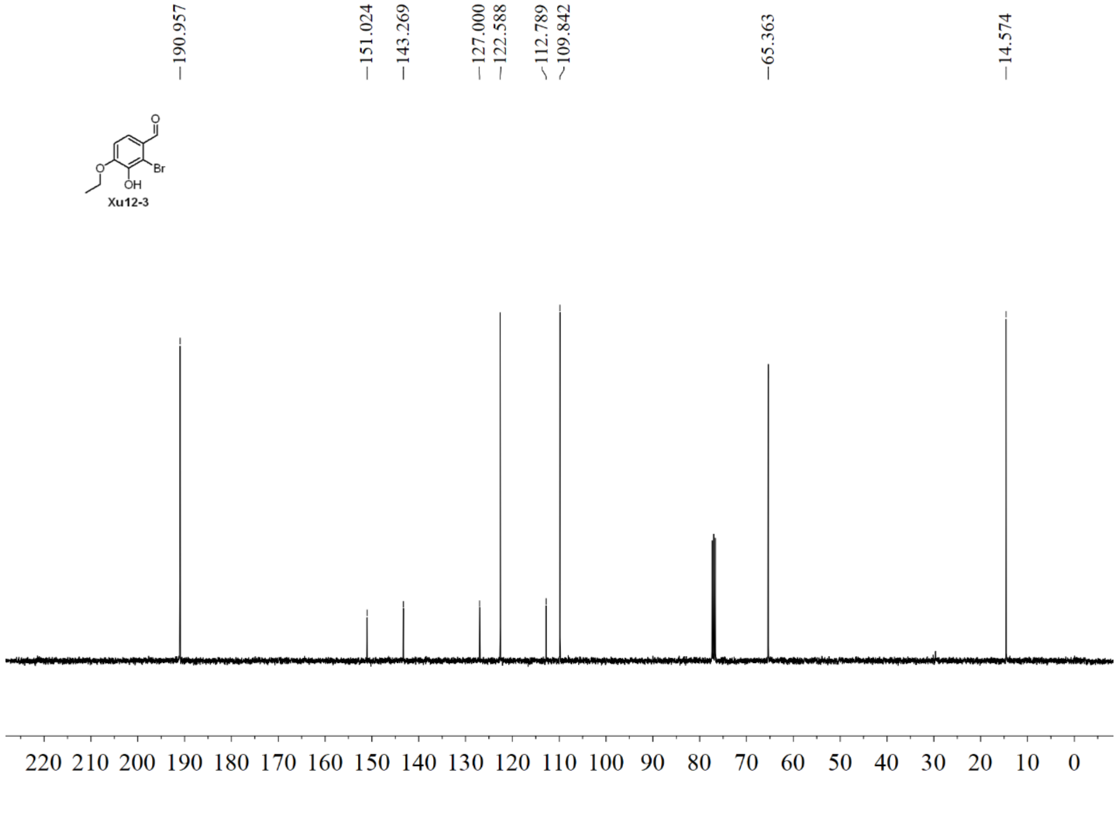

190.957
151.024
143.269
127.000
122.588
112.789
109.842
65.363
14.574
Br
OH
Xu12-3
220 210 200 190 180 170 160 150 140 130 120 110 100 90 80 70 60 50 40 30 20 10 0

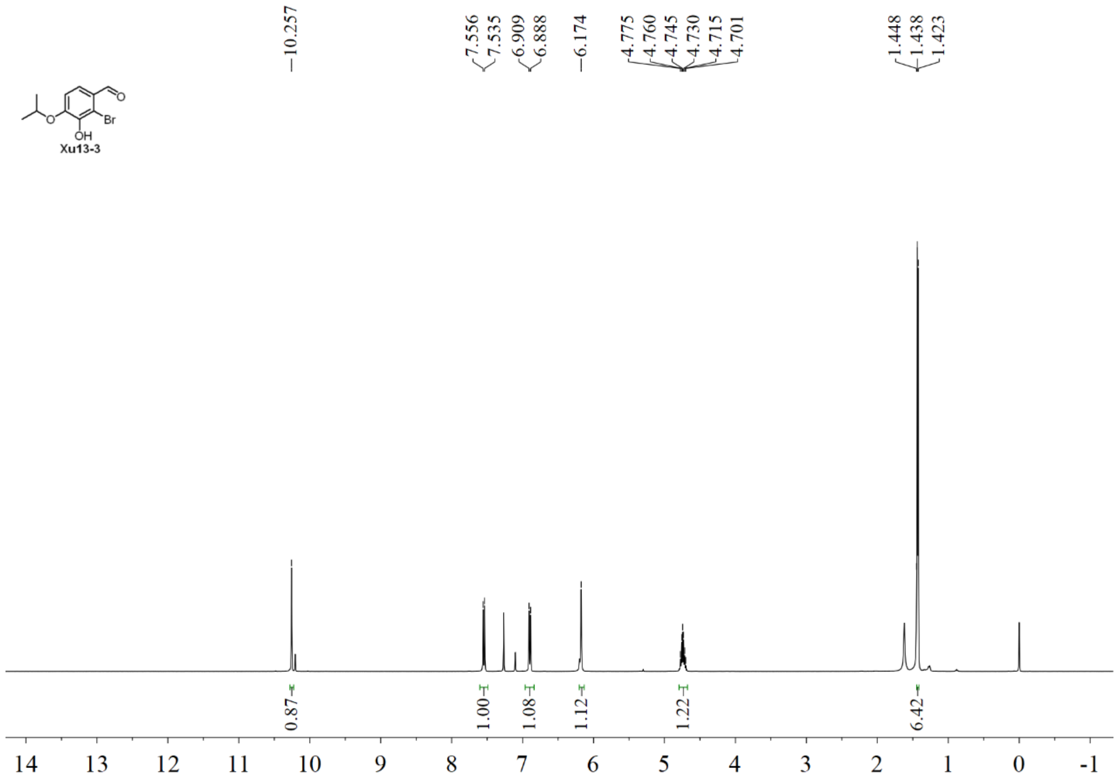

10.257
7.556
7.535
6.909
6.888
6.174
4.775
4.760
4.745
4.730
4.715
4.701
1.448
1.438
1.423
Br
OH
Xu13-3
0.87
1.00
1.08
1.12
1.22
6.42
14 13 12 11 10 9 8 7 6 5 4 3 2 1 0 -1

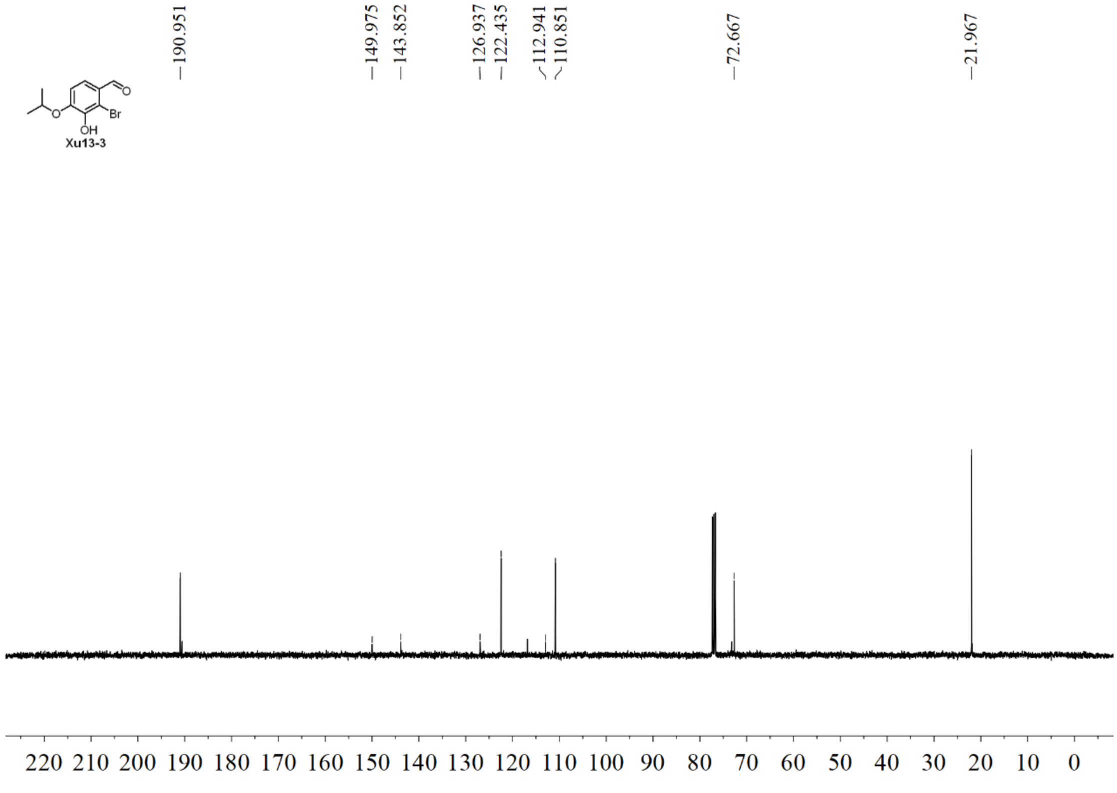

190.951
149.975
143.852
126.937
122.435
112.941
110.851
72.667
21.967
Br
OH
Xu13-3
220 210 200 190 180 170 160 150 140 130 120 110 100 90 80 70 60 50 40 30 20 10 0

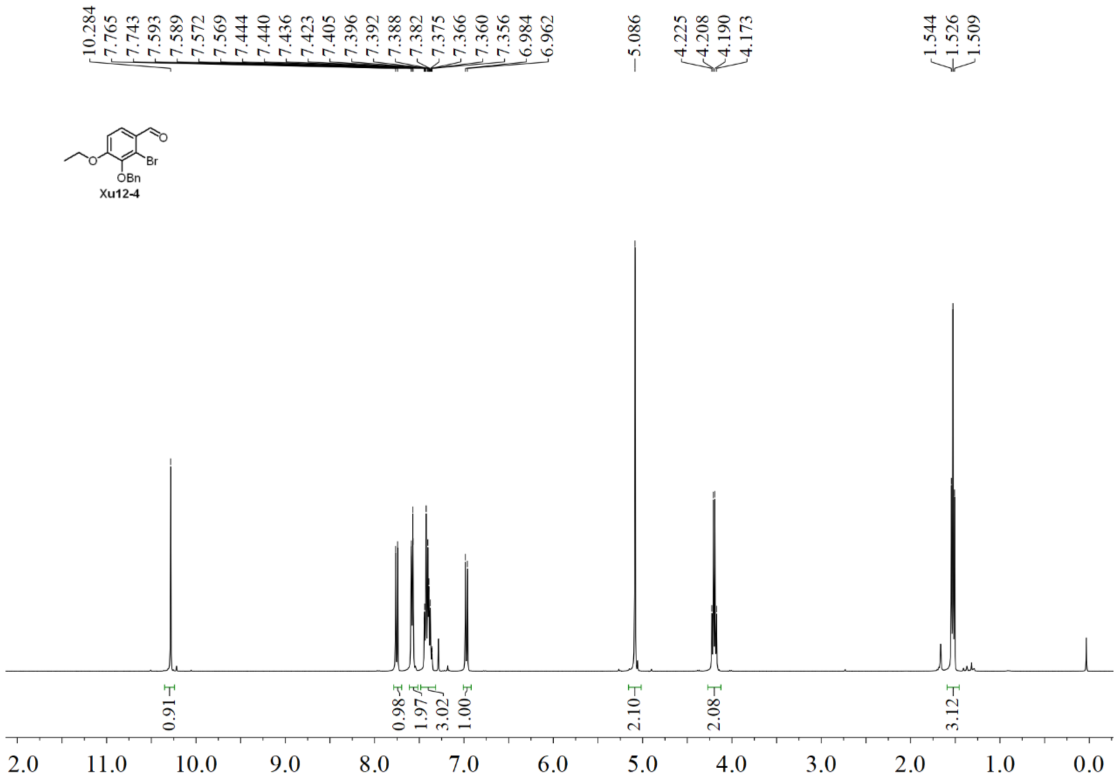
10.284
7.765
7.743
7.593
7.589
7.572
7.569
7.444
7.440
7.436
7.423
7.405
7.396
7.392
7.388
7.382
7.375
7.366
7.360
7.356
6.984
6.962
5.086
4.225
4.208
4.190
4.173
1.544
1.526
1.509
Br
OBn
Xu12-4
0.91
0.98
1.97
3.02
1.00
2.10
2.08
3.12
2.0
11.0
10.0
9.0
8.0
7.0
6.0
5.0
4.0
3.0
2.0
1.0
0.0

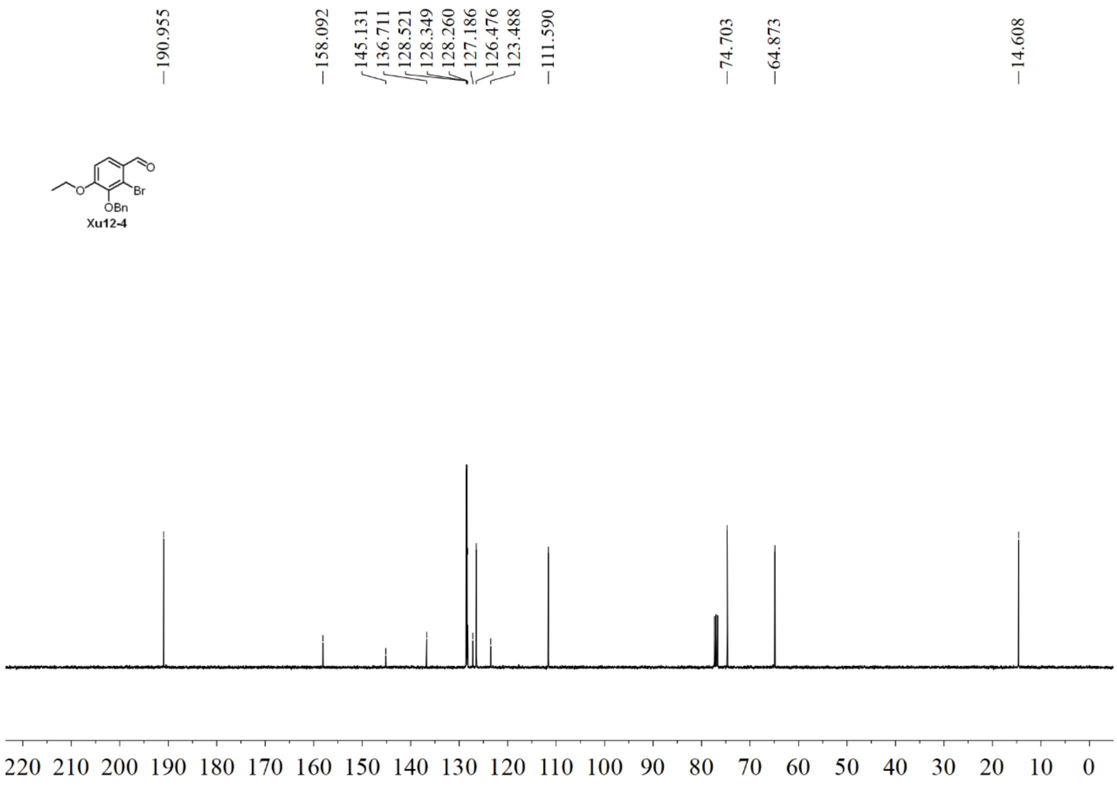
190.955
158.092
145.131
136.711
128.521
128.349
128.260
127.186
126.476
123.488
111.590
74.703
64.873
14.608
Br
OBn
Xu12-4
220 210 200 190 180 170 160 150 140 130 120 110 100 90 80 70 60 50 40 30 20 10 0

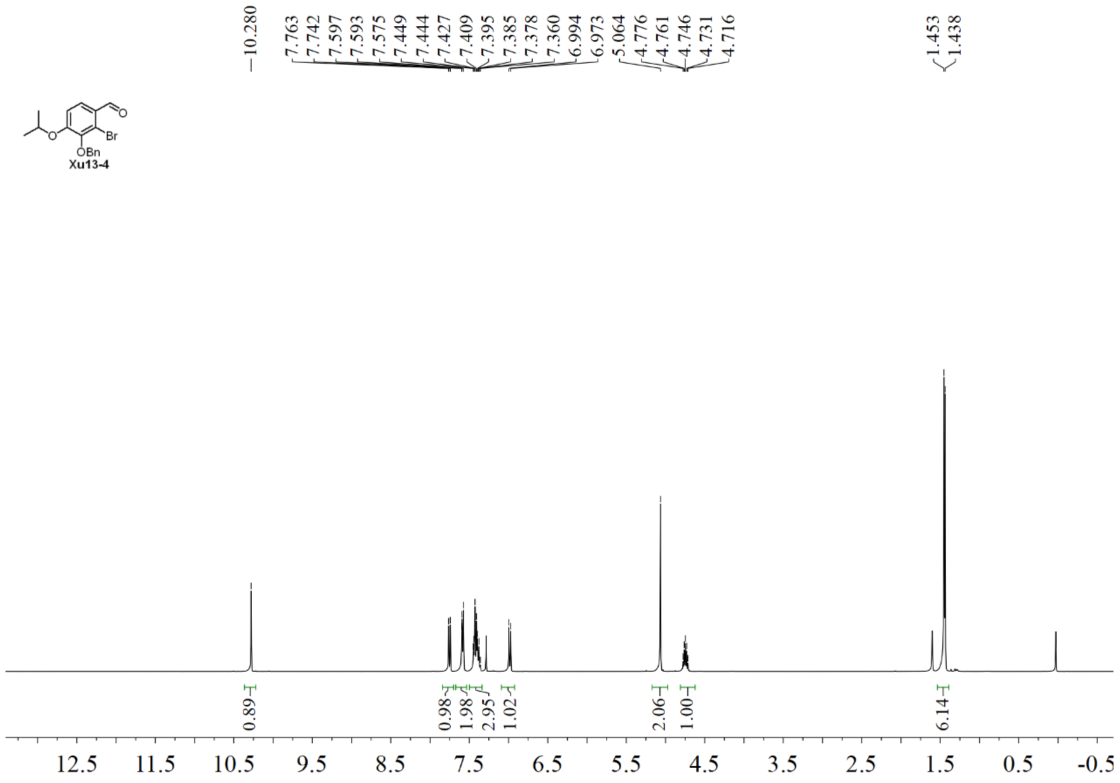
10.280
7.763 7.742 7.597 7.593 7.575 7.449 7.444 7.427 7.409 7.395 7.385 7.378 7.360 6.994 6.973
5.064 4.776 4.761 4.746 4.731 4.716
1.453 1.438
Br
OBn
Xu13-4
0.89 0.98 1.98 2.95 1.02 2.06 1.00 6.14
12.5 11.5 10.5 9.5 8.5 7.5 6.5 5.5 4.5 3.5 2.5 1.5 0.5 -0.5

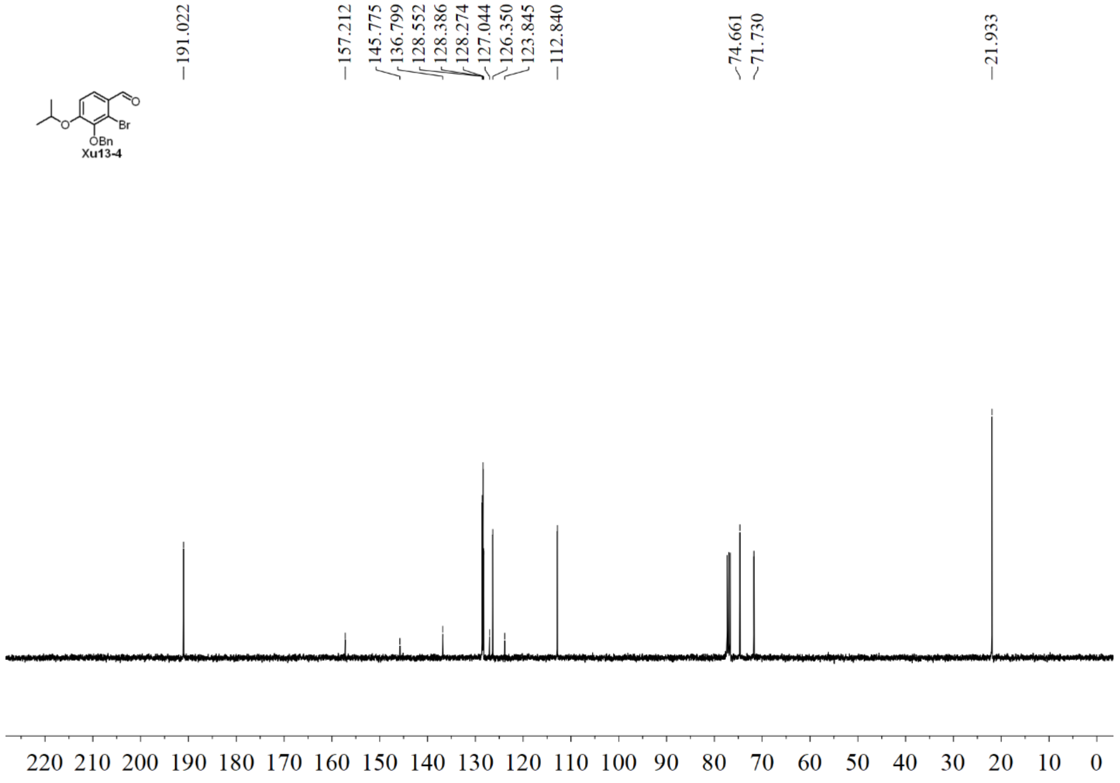
191.022
157.212 145.775 136.799 128.552 128.386 128.274 127.044 126.350 123.845 112.840
74.661 71.730
21.933
Br
OBn
Xu13-4
220 210 200 190 180 170 160 150 140 130 120 110 100 90 80 70 60 50 40 30 20 10 0

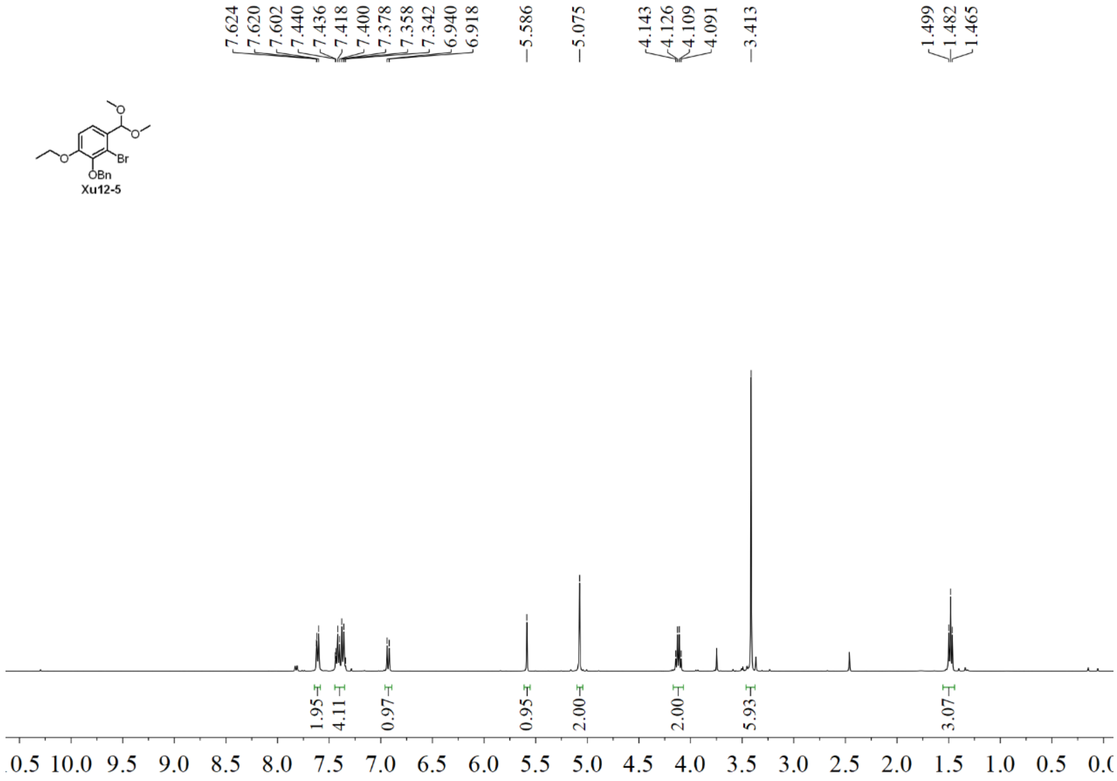
7.624
7.620
7.602
7.440
7.436
7.418
7.400
7.378
7.358
7.342
6.940
6.918
5.586
5.075
4.143
4.126
4.109
4.091
3.413
1.499
1.482
1.465
Br
OBn
Xu12-5
1.95
4.11
0.97
0.95
2.00
2.00
5.93
3.07
.0.5 10.0 9.5 9.0 8.5 8.0 7.5 7.0 6.5 6.0 5.5 5.0 4.5 4.0 3.5 3.0 2.5 2.0 1.5 1.0 0.5 0.0

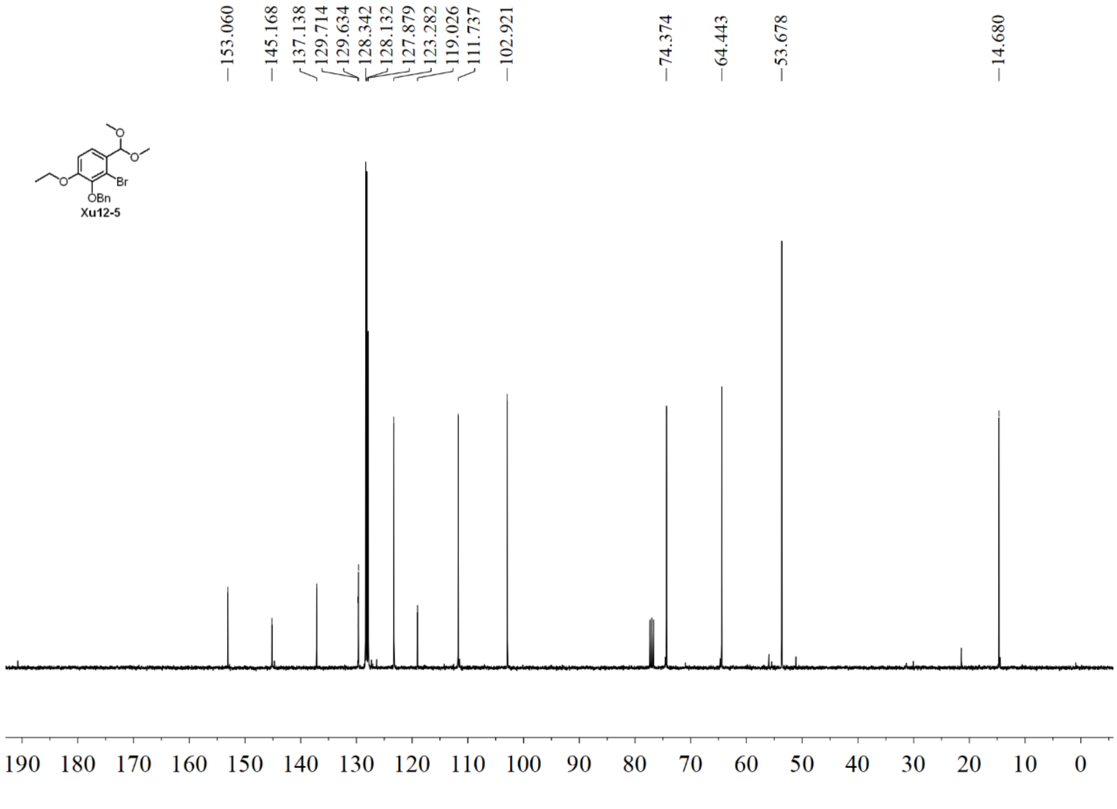
153.060
145.168
137.138
129.714
129.634
128.342
128.132
127.879
123.282
119.026
111.737
102.921
74.374
64.443
53.678
14.680
Br
OBn
Xu12-5
190 180 170 160 150 140 130 120 110 100 90 80 70 60 50 40 30 20 10 0

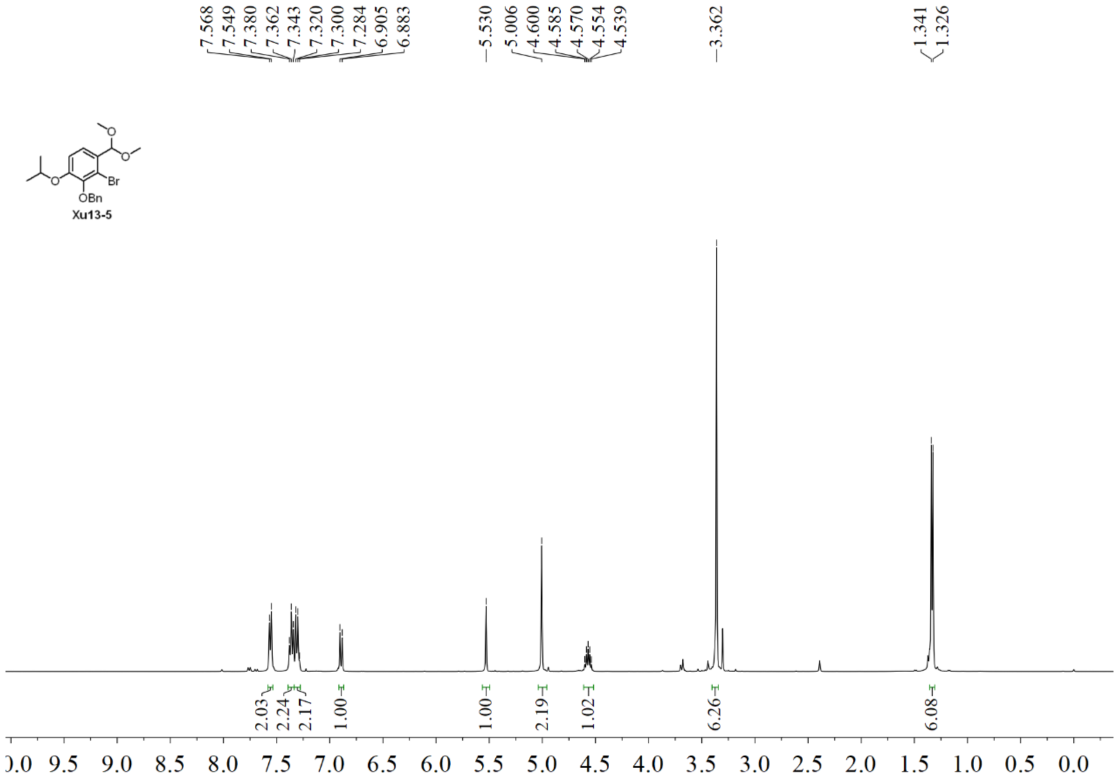
7.568
7.549
7.380
7.362
7.343
7.320
7.300
7.284
6.905
6.883
5.530
5.006
4.600
4.585
4.570
4.554
4.539
3.362
1.341
1.326
Br
OBn
Xu13-5
2.03
2.24
2.17
1.00
1.00
2.19
1.02
6.26
6.08
0.0 9.5 9.0 8.5 8.0 7.5 7.0 6.5 6.0 5.5 5.0 4.5 4.0 3.5 3.0 2.5 2.0 1.5 1.0 0.5 0.0

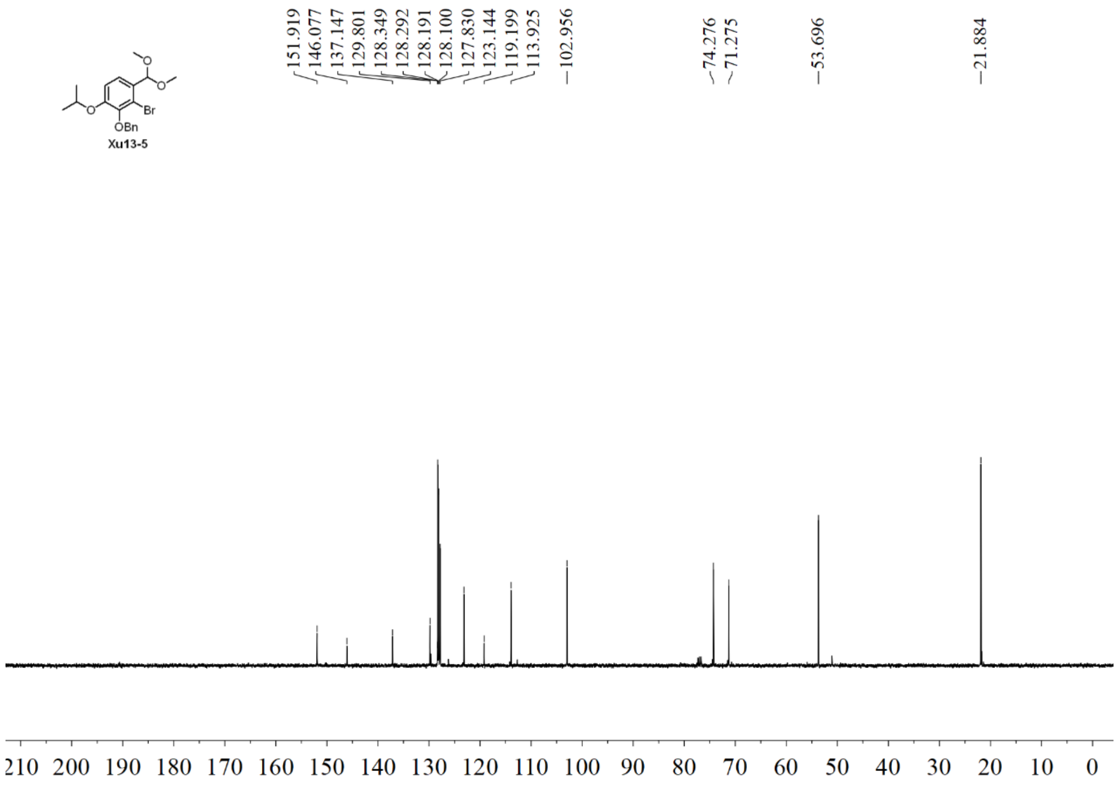
151.919
146.077
137.147
129.801
128.349
128.292
128.191
128.100
127.830
123.144
119.199
113.925
102.956
74.276
71.275
53.696
21.884
Br
OBn
Xu13-5
210 200 190 180 170 160 150 140 130 120 110 100 90 80 70 60 50 40 30 20 10 0

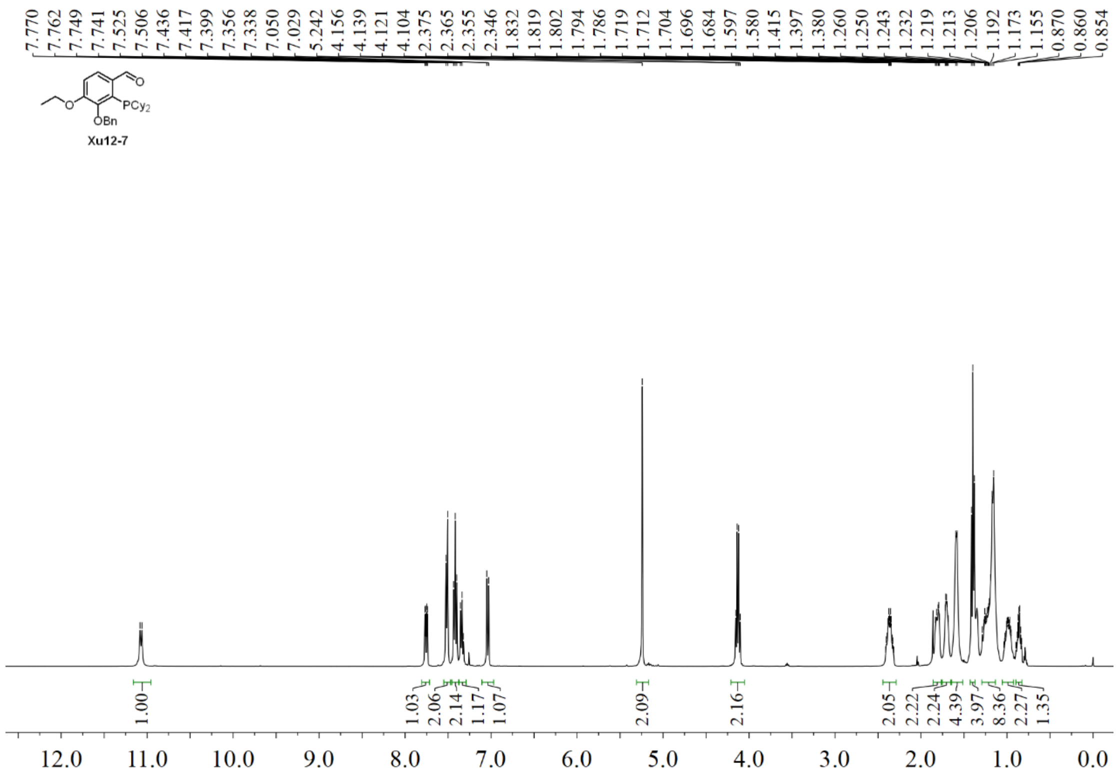
7.770 7.762 7.749 7.741 7.525 7.506 7.436 7.417 7.399 7.356 7.338 7.050 7.029 5.242 4.156 4.139 4.121 4.104 2.375 2.365 2.355 2.346 1.832 1.819 1.802 1.794 1.786 1.719 1.712 1.704 1.696 1.684 1.597 1.580 1.415 1.397 1.380 1.260 1.250 1.243 1.232 1.219 1.213 1.206 1.192 1.173 1.155 0.870 0.860 0.854
PCy2
OBn
Xu12-7
1.00 1.03 2.06 2.14 1.17 1.07 2.09 2.16 2.05 2.22 2.24 4.39 3.97 8.36 2.27 1.35
12.0 11.0 10.0 9.0 8.0 7.0 6.0 5.0 4.0 3.0 2.0 1.0 0.0

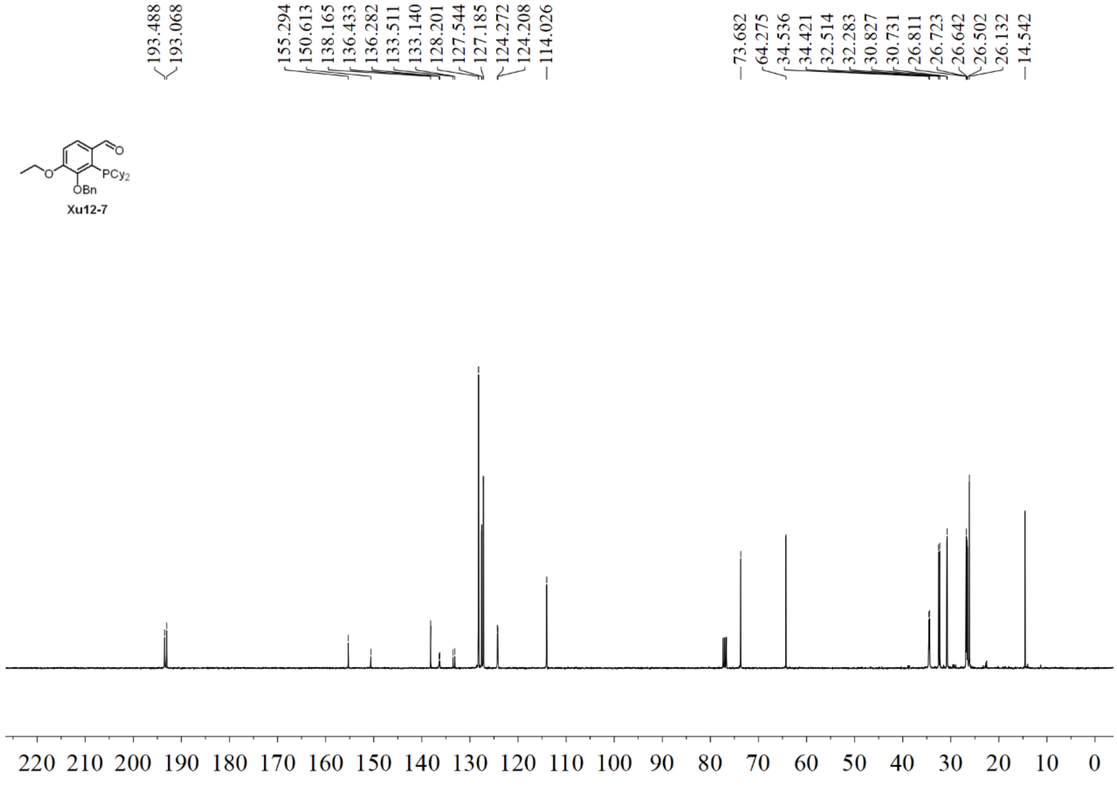
193.488 193.068 155.294 150.613 138.165 136.433 136.282 133.511 133.140 128.201 127.544 127.185 124.272 124.208 114.026 73.682 64.275 34.536 34.421 32.514 32.283 30.827 30.731 26.811 26.723 26.642 26.502 26.132 14.542
PCy2
OBn
Xu12-7
220 210 200 190 180 170 160 150 140 130 120 110 100 90 80 70 60 50 40 30 20 10 0

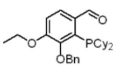

Xu12-7

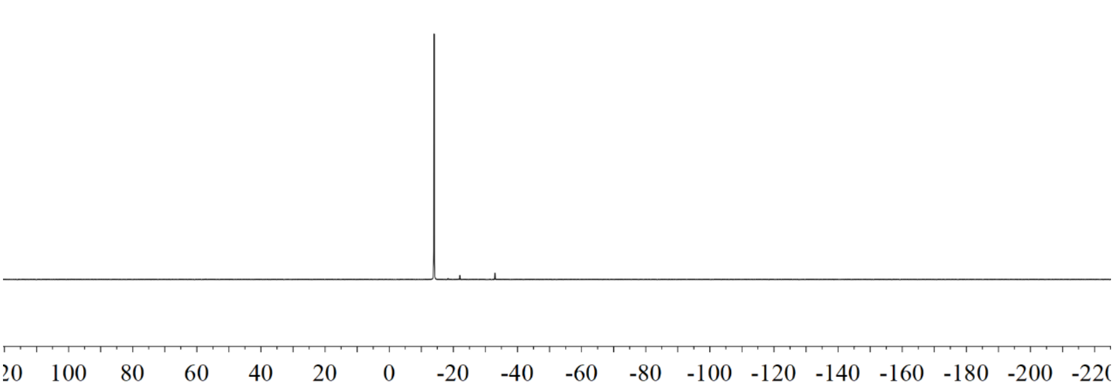

-13.992
20 100 80 60 40 20 0 -20 -40 -60 -80 -100 -120 -140 -160 -180 -200 -220

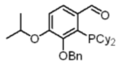

Xu13-7

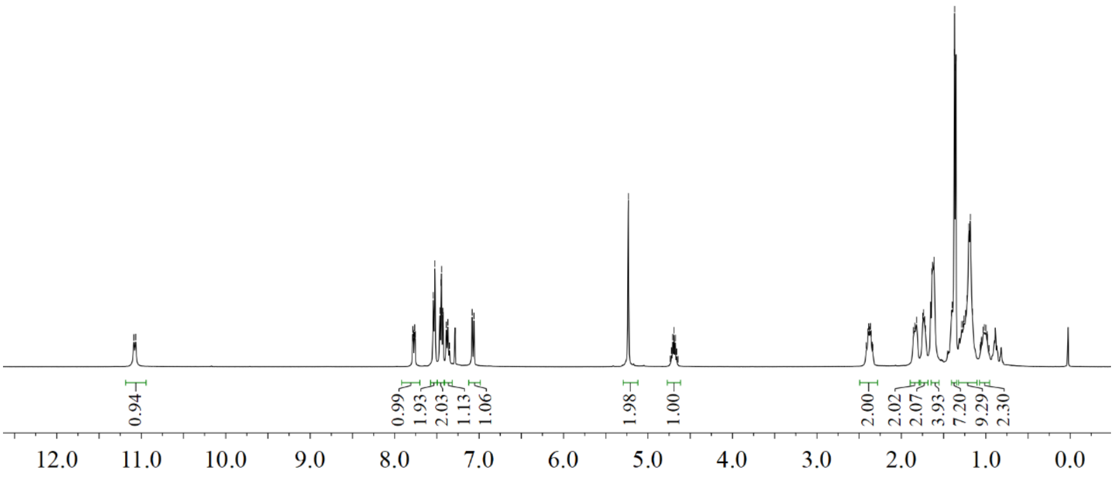

7.784 7.777 7.762 7.755 7.542 7.523 7.465 7.449 7.445 7.429 7.426 7.388 7.369 7.083 7.061 5.233 4.692 2.395 2.387 2.377 2.366 2.358 1.855 1.842 1.819 1.746 1.739 1.724 1.713 1.639 1.629 1.613 1.407 1.397 1.372 1.369 1.357 1.353 1.286 1.269 1.257 1.238 1.238 1.223 1.202 1.182 1.158 1.031 1.017 0.998
0.94 0.99 1.93 2.03 1.13 1.06 1.98 1.00 2.00 2.02 2.07 3.93 7.20 9.29 2.30
12.0 11.0 10.0 9.0 8.0 7.0 6.0 5.0 4.0 3.0 2.0 1.0 0.0

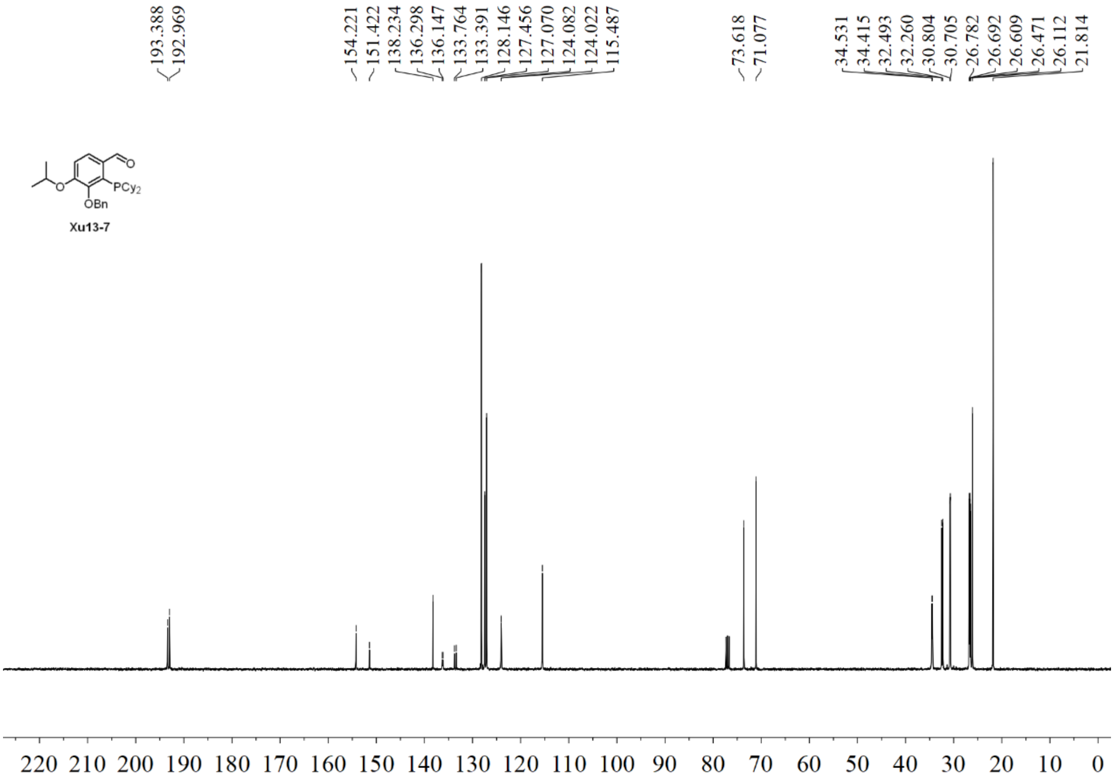
193.388
192.969
154.221
151.422
138.234
136.298
136.147
133.764
133.391
128.146
127.456
127.070
124.082
124.022
115.487
73.618
71.077
34.531
34.415
32.493
32.260
30.804
30.705
26.782
26.692
26.609
26.471
26.112
21.814
PCy2
OBn
Xu13-7
220 210 200 190 180 170 160 150 140 130 120 110 100 90 80 70 60 50 40 30 20 10 0

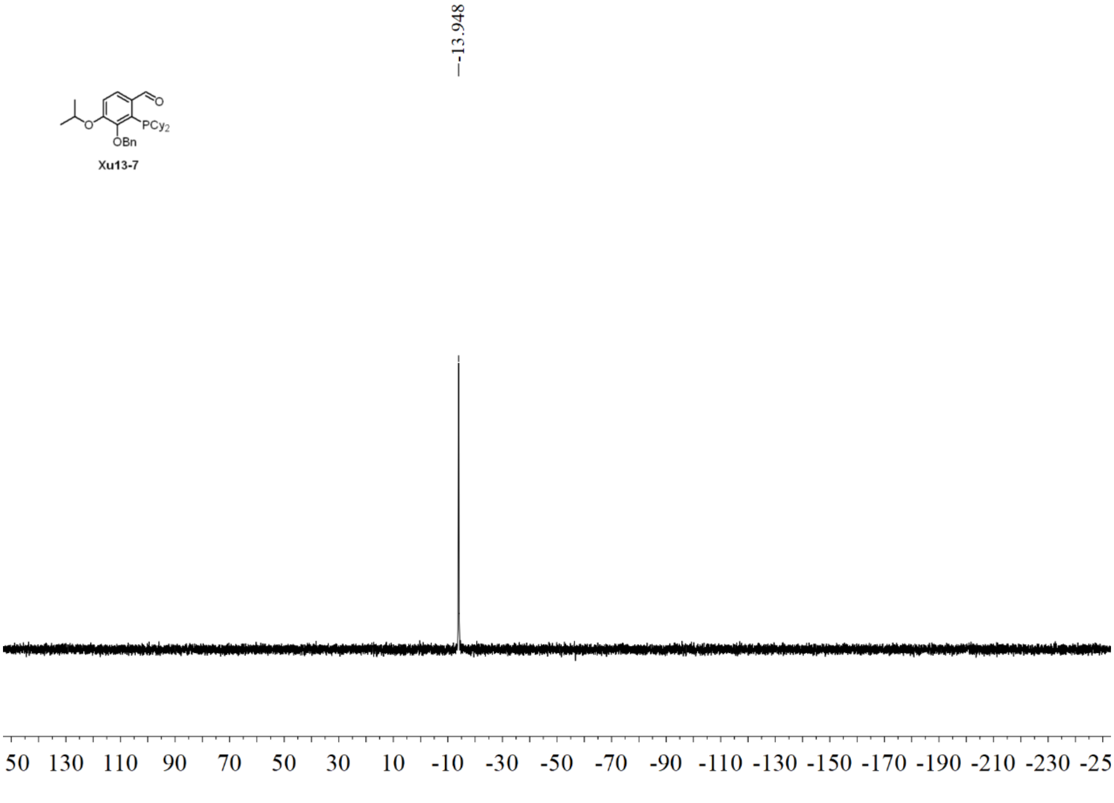
-13.948
PCy2
OBn
Xu13-7
50 130 110 90 70 50 30 10 -10 -30 -50 -70 -90 -110 -130 -150 -170 -190 -210 -230 -25

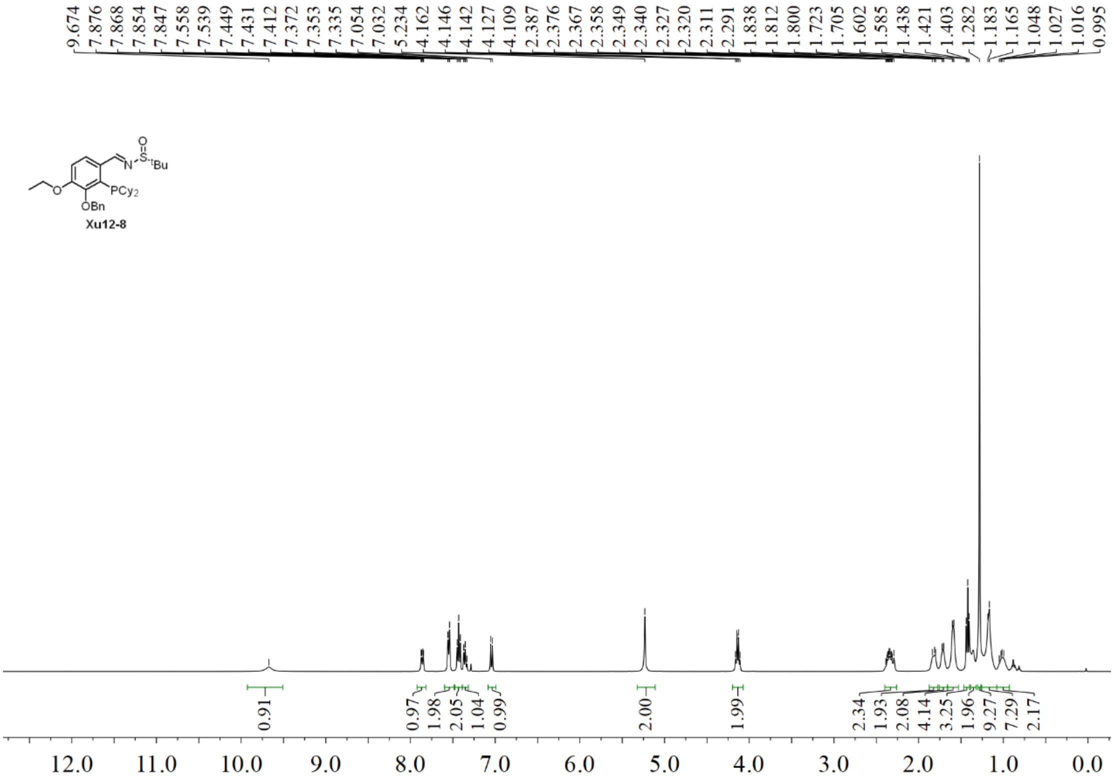
9.674 7.876 7.868 7.854 7.847 7.558 7.539 7.449 7.431 7.412 7.372 7.353 7.335 7.054 7.032 5.234 4.162 4.146 4.142 4.127 4.109 2.387 2.376 2.367 2.358 2.349 2.340 2.327 2.320 2.311 2.291 1.838 1.812 1.800 1.723 1.705 1.602 1.585 1.438 1.421 1.403 1.282 1.183 1.165 1.048 1.027 1.016 0.995
PCy2
OBn
tBu
Xu12-8
0.91
0.97 1.98 2.05 1.04 0.99
2.00
1.99
2.34 1.93 2.08 4.14 3.25 1.96 9.27 7.29 2.17
12.0 11.0 10.0 9.0 8.0 7.0 6.0 5.0 4.0 3.0 2.0 1.0 0.0

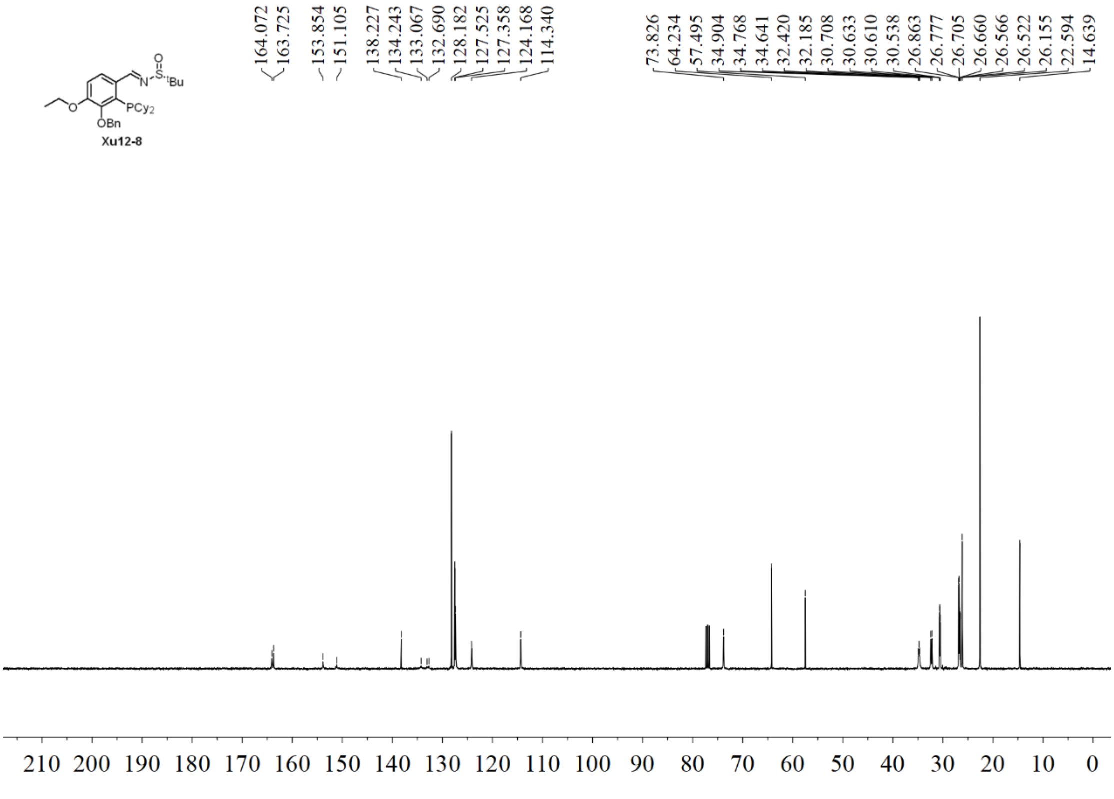
164.072 163.725 153.854 151.105 138.227 134.243 133.067 132.690 128.182 127.525 127.358 124.168 114.340
73.826 64.234 57.495 34.904 34.768 34.641 32.420 32.185 30.708 30.633 30.610 30.538 26.863 26.777 26.705 26.660 26.566 26.522 26.155 22.594 14.639
PCy2
OBn
tBu
Xu12-8
210 200 190 180 170 160 150 140 130 120 110 100 90 80 70 60 50 40 30 20 10 0

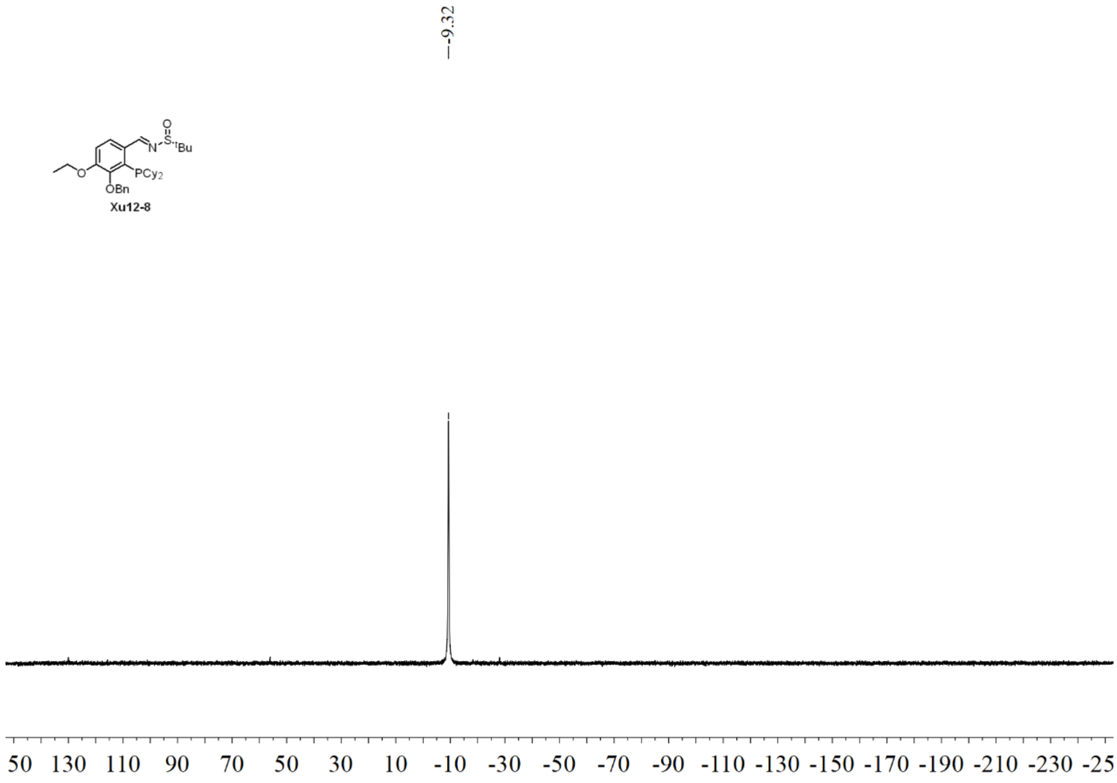
-9.32
Xu12-8
O
N
S
tBu
O
PCy2
OBn
50 130 110 90 70 50 30 10 -10 -30 -50 -70 -90 -110 -130 -150 -170 -190 -210 -230 -25

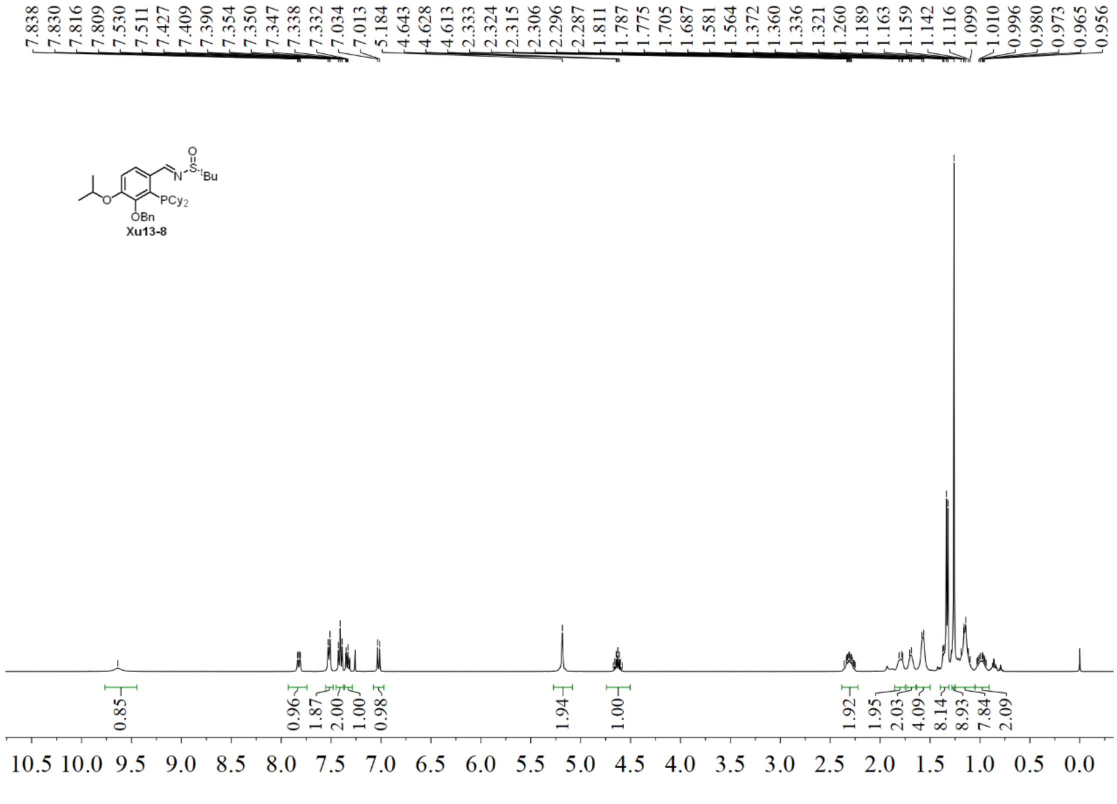
7.838
7.830
7.816
7.809
7.530
7.511
7.427
7.409
7.390
7.354
7.350
7.347
7.338
7.332
7.034
7.013
5.184
4.643
4.628
4.613
2.333
2.324
2.315
2.306
2.296
2.287
1.811
1.787
1.775
1.705
1.687
1.581
1.564
1.372
1.360
1.336
1.321
1.260
1.189
1.163
1.159
1.142
1.116
1.099
1.010
0.996
0.980
0.973
0.965
0.956
Xu13-8
O
N
S
tBu
O
PCy2
OBn
0.85
0.96
1.87
2.00
1.00
0.98
1.94
1.00
1.92
1.95
2.03
4.09
8.14
8.93
7.84
2.09
10.5 10.0 9.5 9.0 8.5 8.0 7.5 7.0 6.5 6.0 5.5 5.0 4.5 4.0 3.5 3.0 2.5 2.0 1.5 1.0 0.5 0.0

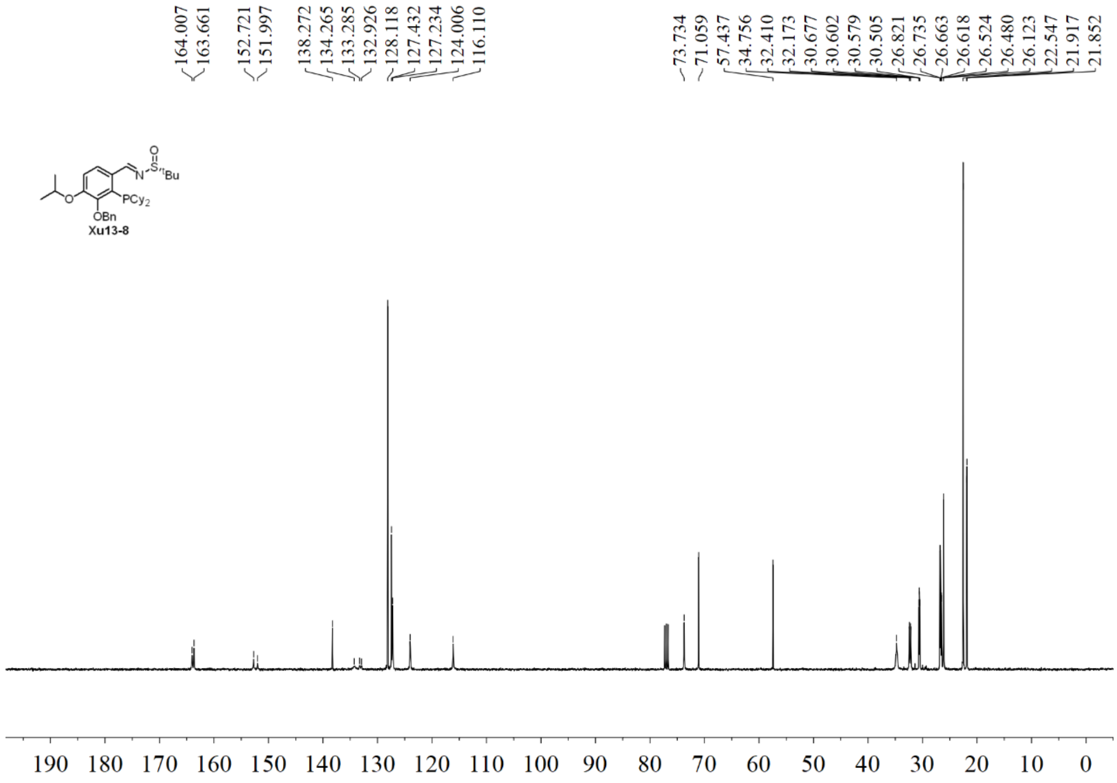
164.007
163.661
152.721
151.997
138.272
134.265
133.285
132.926
128.118
127.432
127.234
124.006
116.110
73.734
71.059
57.437
34.756
32.410
32.173
30.677
30.602
30.579
30.505
26.821
26.735
26.663
26.618
26.524
26.480
26.123
22.547
21.917
21.852
PCy2
OBn
Xu13-8
190 180 170 160 150 140 130 120 110 100 90 80 70 60 50 40 30 20 10 0

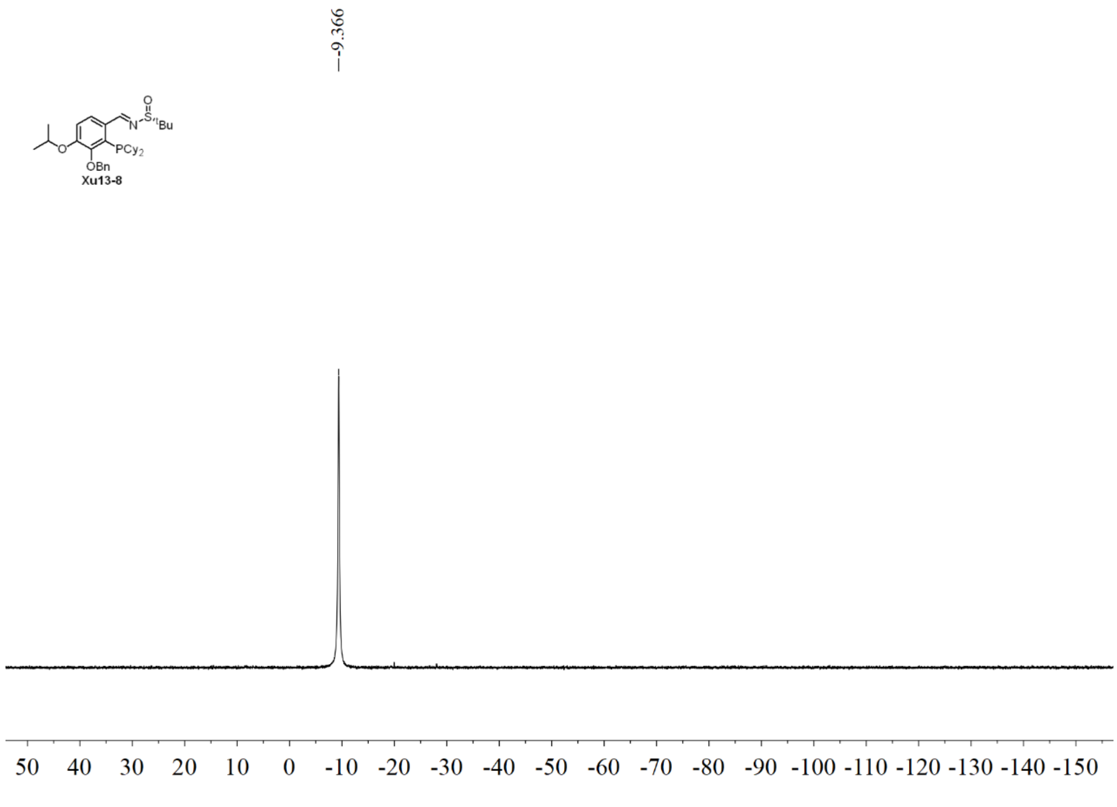
-9.366
PCy2
OBn
Xu13-8
50 40 30 20 10 0 -10 -20 -30 -40 -50 -60 -70 -80 -90 -100 -110 -120 -130 -140 -150

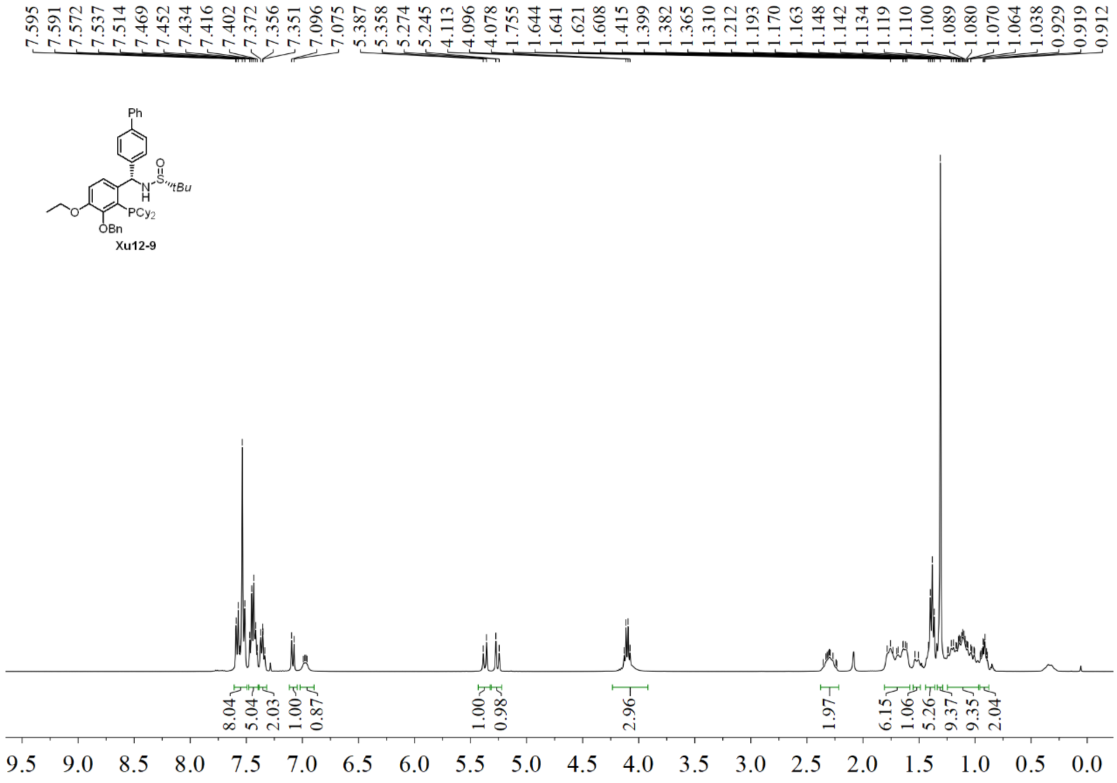
7.595 7.591 7.572 7.537 7.514 7.469 7.452 7.434 7.416 7.402 7.372 7.356 7.351 7.096 7.075 5.387 5.358 5.274 5.245 4.113 4.096 4.078 1.755 1.644 1.641 1.621 1.608 1.415 1.399 1.382 1.365 1.310 1.212 1.193 1.170 1.163 1.148 1.142 1.134 1.119 1.110 1.100 1.089 1.080 1.070 1.064 1.038 0.929 0.919 0.912
Ph
O
N
H
S
tBu
PCy2
OBn
Xu12-9
8.04 5.04 2.03 1.00 0.87 1.00 0.98 2.96 1.97 6.15 1.06 5.26 9.37 9.35 2.04
9.5 9.0 8.5 8.0 7.5 7.0 6.5 6.0 5.5 5.0 4.5 4.0 3.5 3.0 2.5 2.0 1.5 1.0 0.5 0.0

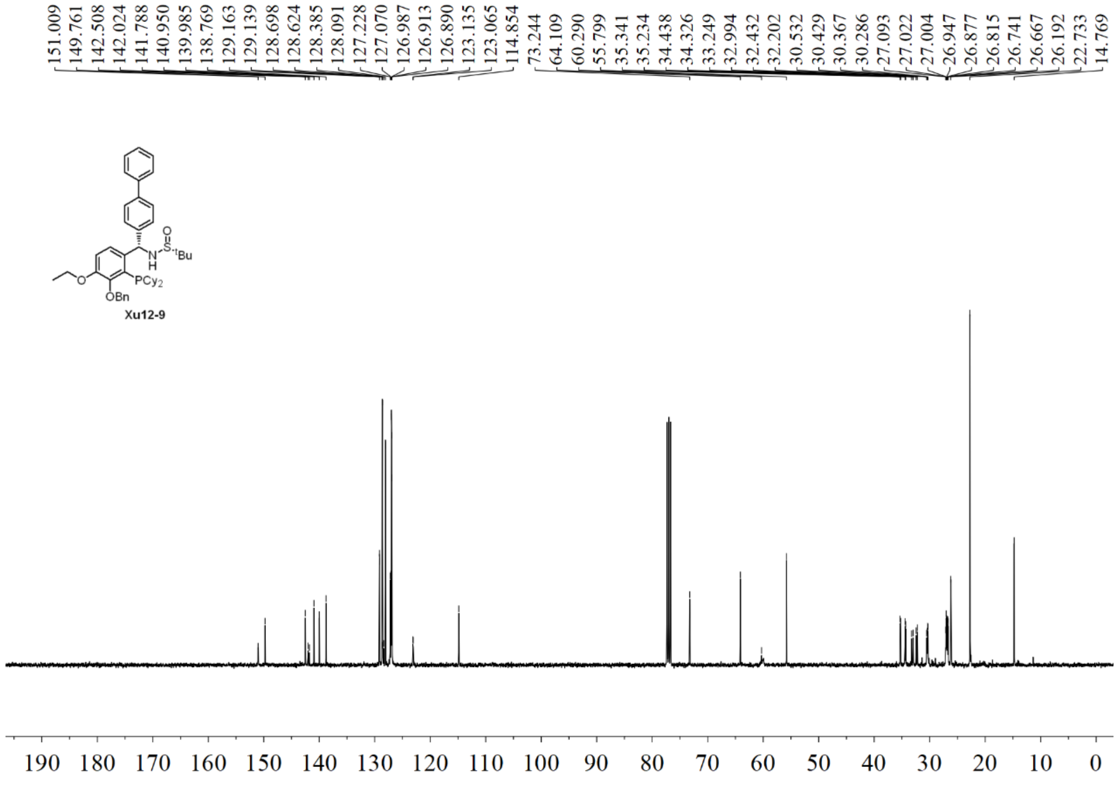
151.009 149.761 142.508 142.024 141.788 140.950 139.985 138.769 129.163 129.139 128.698 128.624 128.385 128.091 127.228 127.070 126.987 126.913 126.890 123.135 123.065 114.854 73.244 64.109 60.290 55.799 35.341 35.234 34.438 34.326 33.249 32.994 32.432 32.202 30.532 30.429 30.367 30.286 27.093 27.022 27.004 26.947 26.877 26.815 26.741 26.667 26.192 22.733 14.769
O
N
H
S
tBu
PCy2
OBn
Xu12-9
190 180 170 160 150 140 130 120 110 100 90 80 70 60 50 40 30 20 10 0

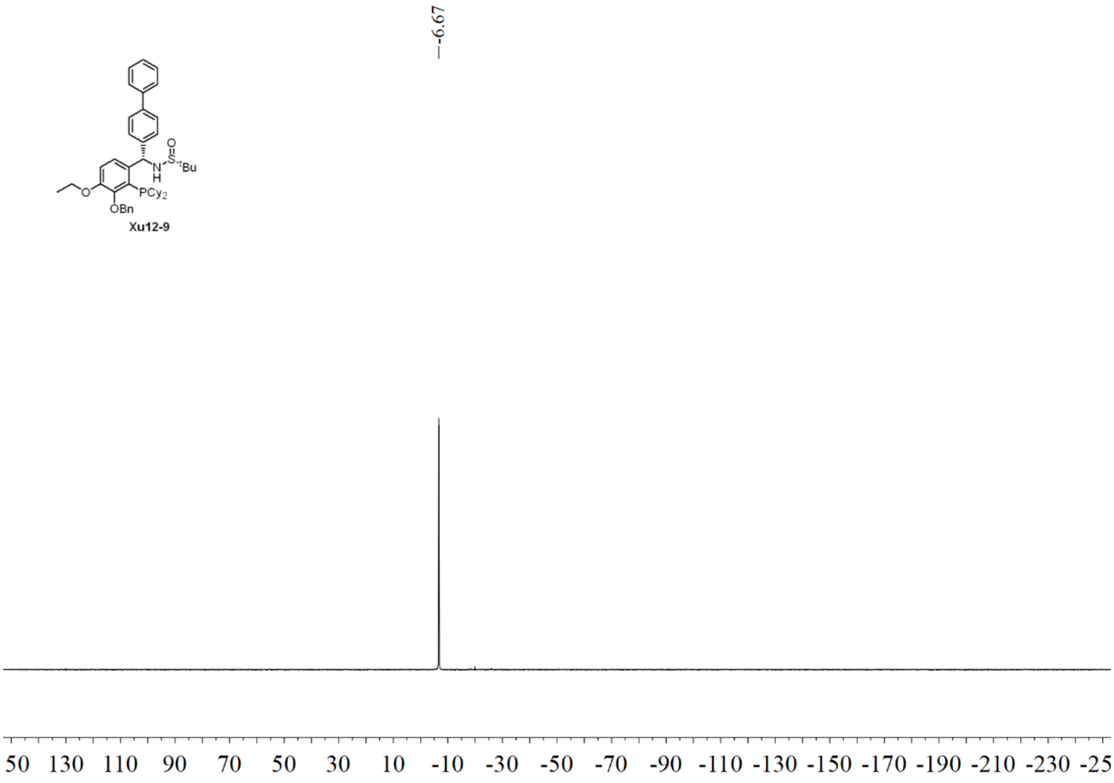

-6.67
PCy2
OBn
S
tBu
Xu12-9

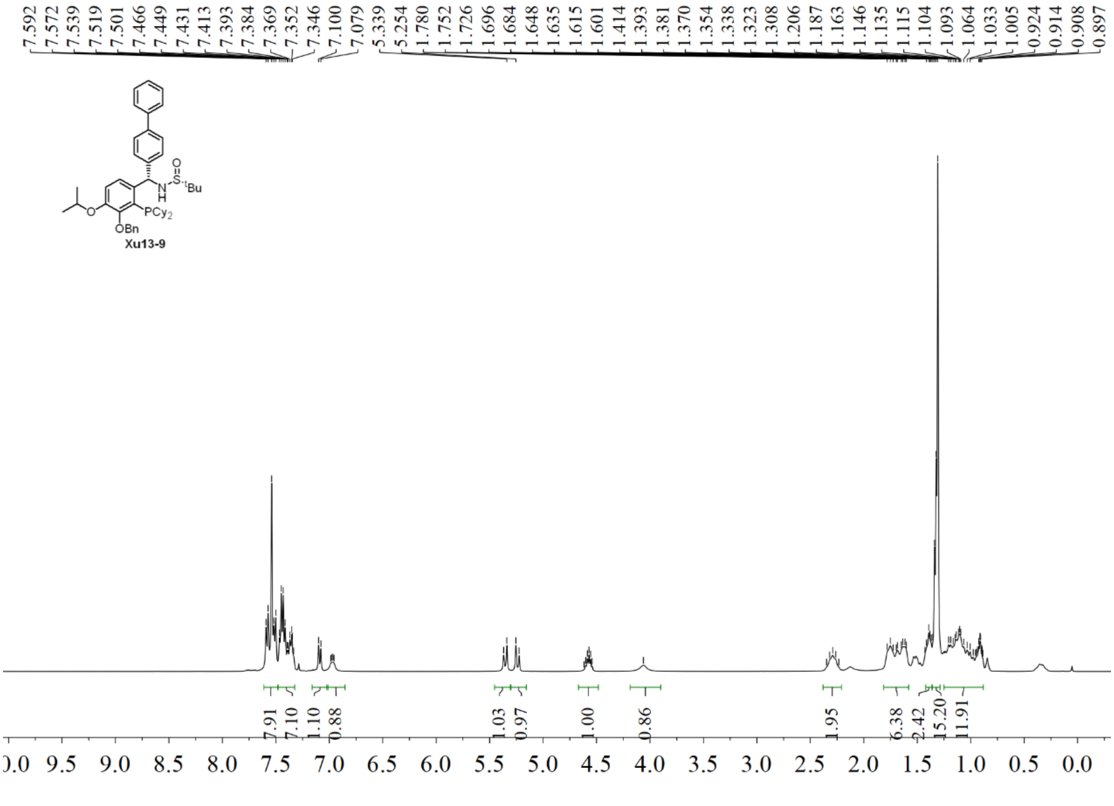

7.592 7.572 7.539 7.519 7.501 7.466 7.449 7.431 7.413 7.393 7.384 7.369 7.352 7.346 7.100 7.079 5.339 5.254 1.780 1.752 1.726 1.696 1.684 1.648 1.635 1.615 1.601 1.414 1.393 1.381 1.370 1.354 1.338 1.323 1.308 1.206 1.187 1.163 1.146 1.135 1.115 1.104 1.093 1.064 1.033 1.005 0.924 0.914 0.908 0.897
PCy2
OBn
S
tBu
Xu13-9
7.91 7.10 1.10 0.88 1.03 0.97 1.00 0.86 1.95 6.38 2.42 15.20 11.91

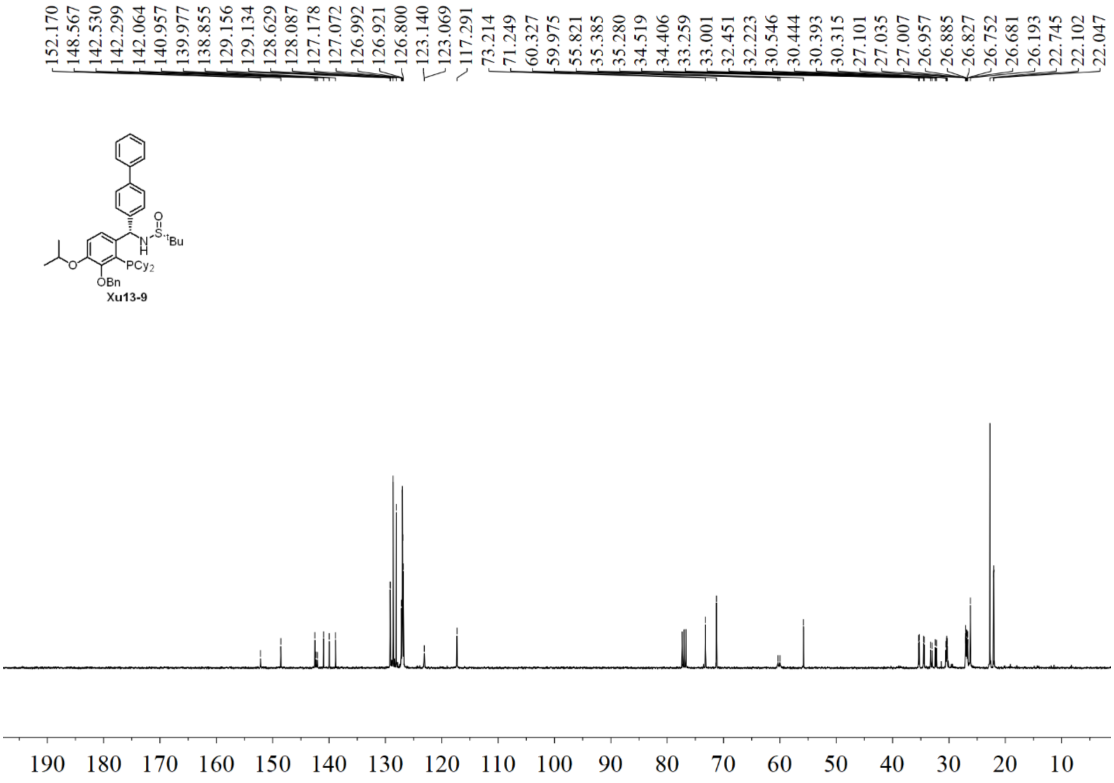
152.170
148.567
142.530
142.299
142.064
140.957
139.977
138.855
129.156
129.134
128.629
128.087
127.178
127.072
126.992
126.921
126.800
123.140
123.069
117.291
73.214
71.249
60.327
59.975
55.821
35.385
35.280
34.519
34.406
33.259
33.001
32.451
32.223
30.546
30.444
30.393
30.315
27.101
27.035
27.007
26.957
26.885
26.827
26.752
26.681
26.193
22.745
22.102
22.047
190 180 170 160 150 140 130 120 110 100 90 80 70 60 50 40 30 20 10

Xu13-9

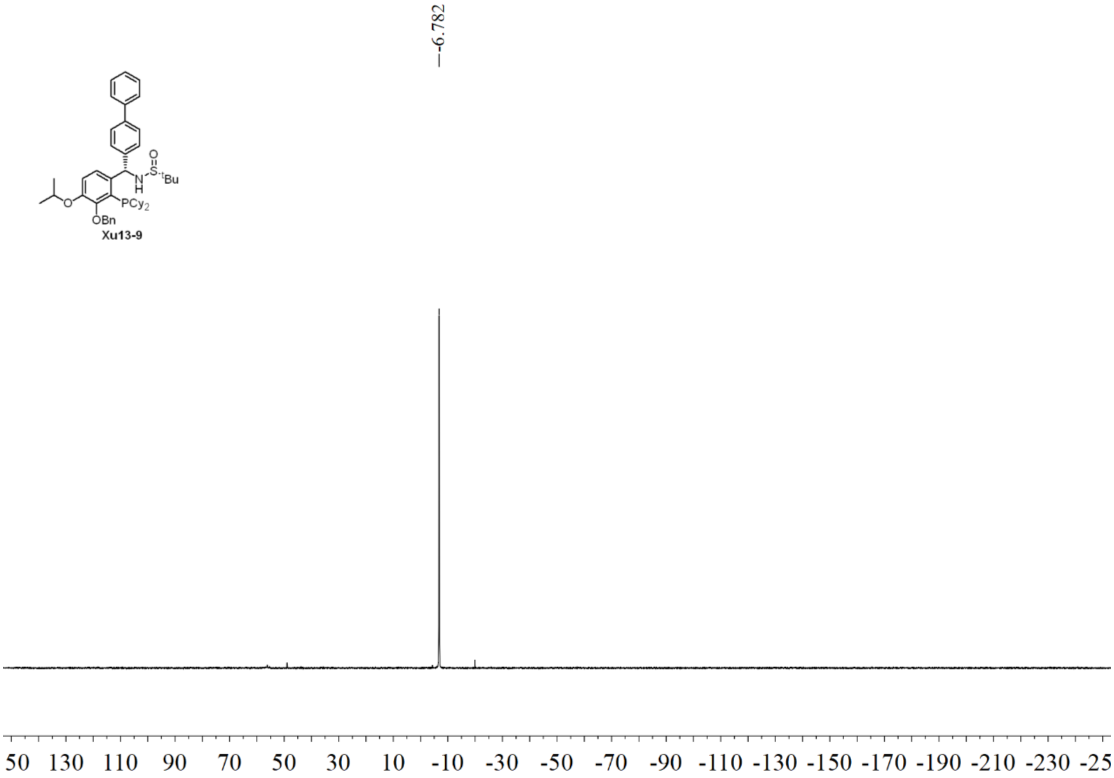
-6.782
50 130 110 90 70 50 30 10 -10 -30 -50 -70 -90 -110 -130 -150 -170 -190 -210 -230 -25

Xu13-9

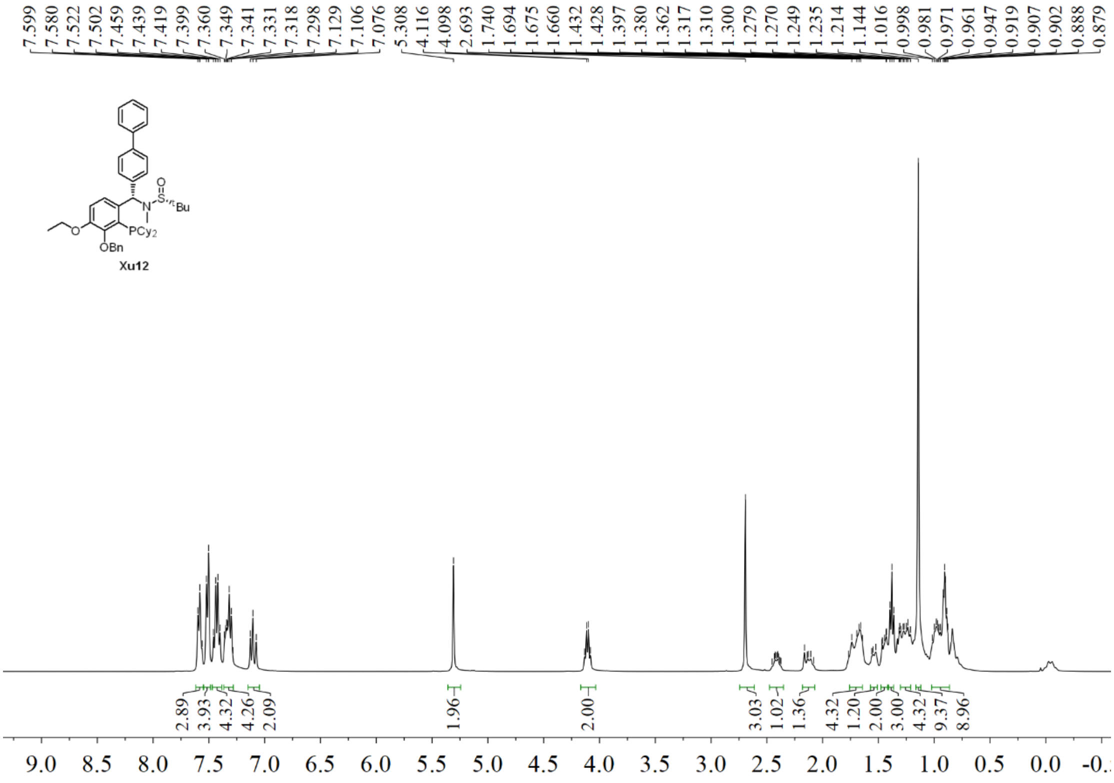
7.599 7.580 7.522 7.502 7.459 7.439 7.419 7.399 7.360 7.349 7.341 7.331 7.318 7.298 7.129 7.106 7.076 5.308 4.116 4.098 2.693 1.740 1.694 1.675 1.660 1.432 1.428 1.397 1.380 1.362 1.317 1.310 1.300 1.279 1.270 1.249 1.235 1.214 1.144 1.016 0.998 0.981 0.971 0.961 0.947 0.919 0.907 0.902 0.888 0.879
O
S
tBu
N
PCy2
O
OBn
Xu12
2.89 3.93 4.32 4.26 2.09 1.96 2.00 3.03 1.02 1.36 4.32 1.20 2.00 3.00 4.32 9.37 8.96
9.0 8.5 8.0 7.5 7.0 6.5 6.0 5.5 5.0 4.5 4.0 3.5 3.0 2.5 2.0 1.5 1.0 0.5 0.0 -0.

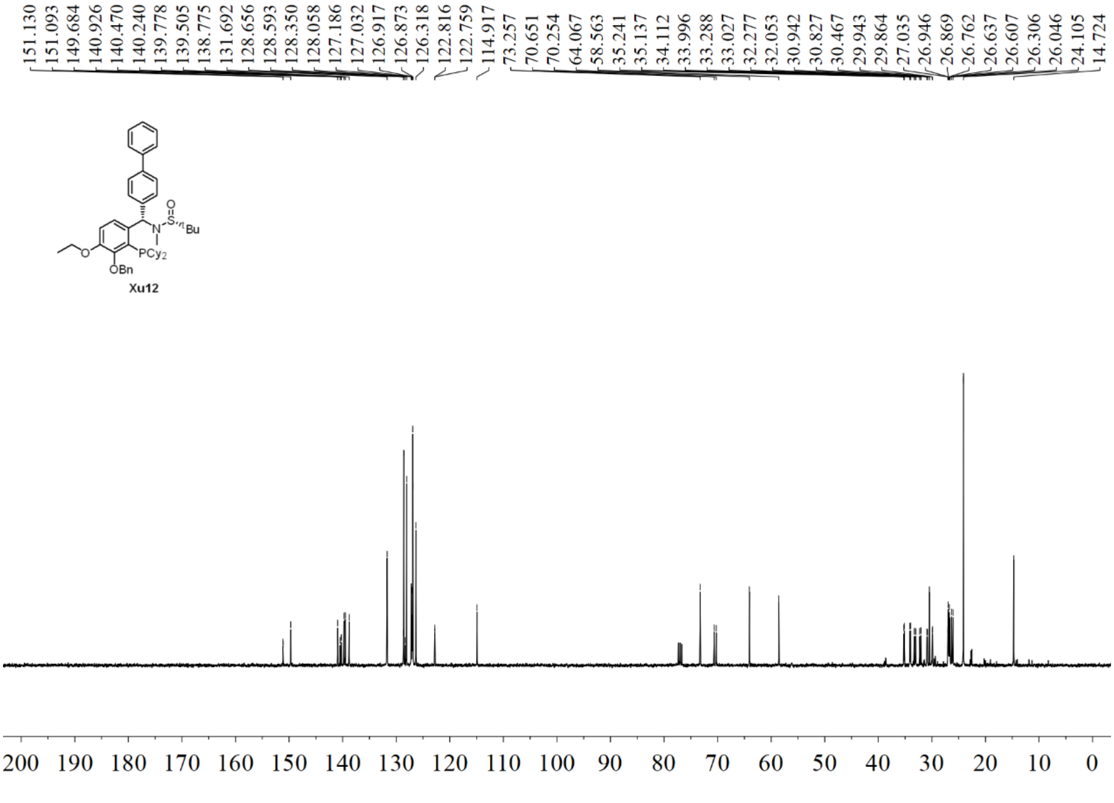
151.130 151.093 149.684 140.926 140.470 140.240 139.778 139.505 138.775 131.692 128.656 128.593 128.350 128.058 127.186 127.032 126.917 126.873 126.318 122.816 122.759 114.917 73.257 70.651 70.254 64.067 58.563 35.241 35.137 34.112 33.996 33.288 33.027 32.277 32.053 30.942 30.827 30.467 29.943 29.864 27.035 26.946 26.869 26.762 26.637 26.607 26.306 26.046 24.105 14.724
O
S
tBu
N
PCy2
O
OBn
Xu12
200 190 180 170 160 150 140 130 120 110 100 90 80 70 60 50 40 30 20 10 0

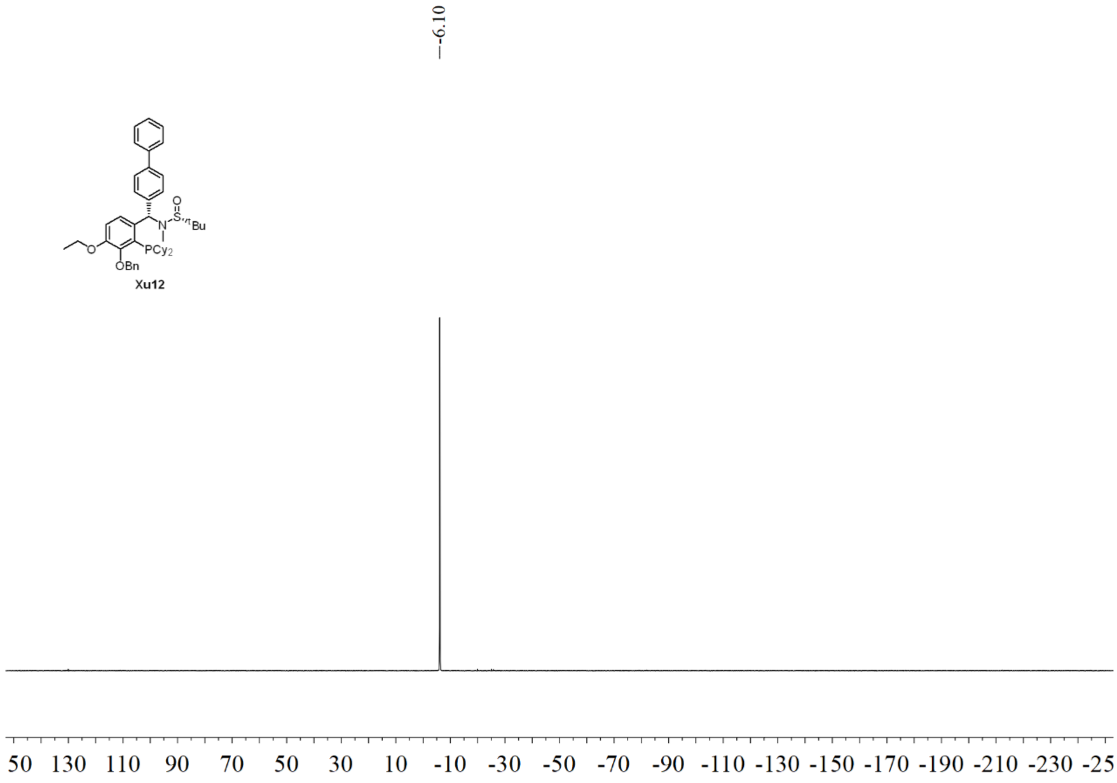
-6.10
Xu12
PCy2
OBn
tBu
50 130 110 90 70 50 30 10 -10 -30 -50 -70 -90 -110 -130 -150 -170 -190 -210 -230 -25

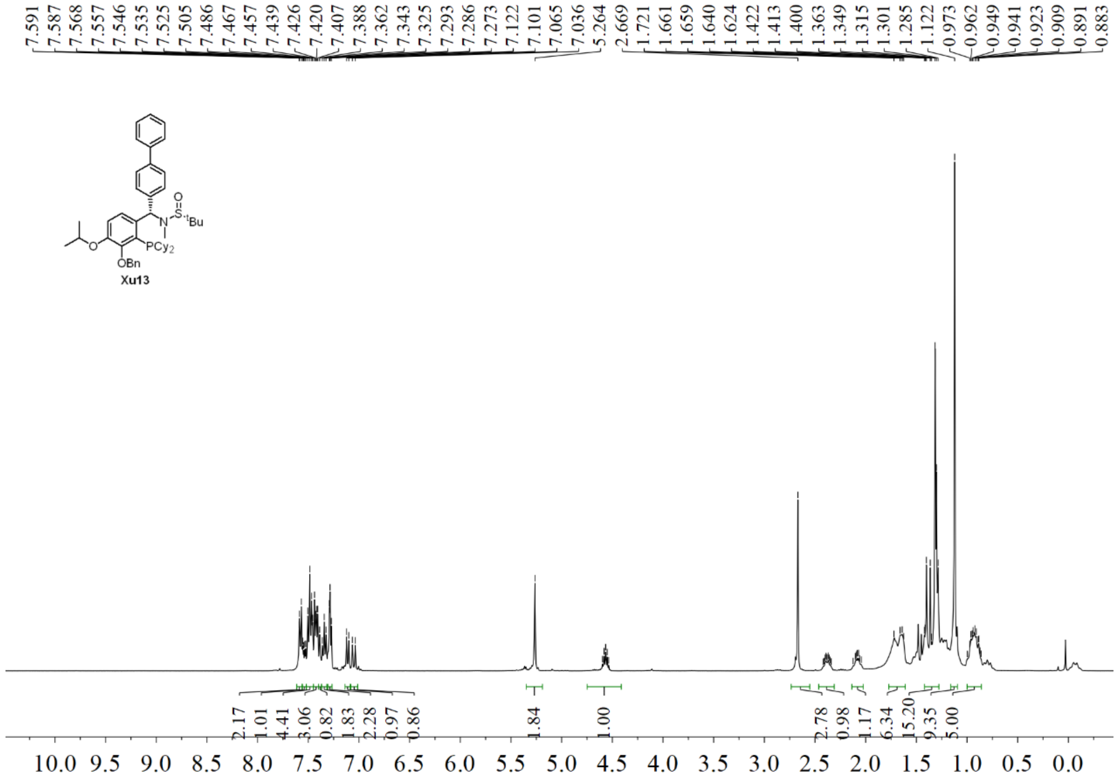
7.591 7.587 7.568 7.557 7.546 7.535 7.525 7.505 7.486 7.467 7.457 7.439 7.426 7.420 7.407 7.388 7.362 7.343 7.325 7.293 7.286 7.273 7.122 7.101 7.065 7.036 5.264 2.669 1.721 1.661 1.659 1.640 1.624 1.422 1.413 1.400 1.363 1.349 1.315 1.301 1.285 1.122 0.973 0.962 0.949 0.941 0.923 0.909 0.891 0.883
Xu13
PCy2
OBn
tBu
2.17 1.01 4.41 3.06 0.82 1.83 2.28 0.97 0.86 1.84 1.00 2.78 0.98 1.17 6.34 15.20 9.35 5.00
10.0 9.5 9.0 8.5 8.0 7.5 7.0 6.5 6.0 5.5 5.0 4.5 4.0 3.5 3.0 2.5 2.0 1.5 1.0 0.5 0.0

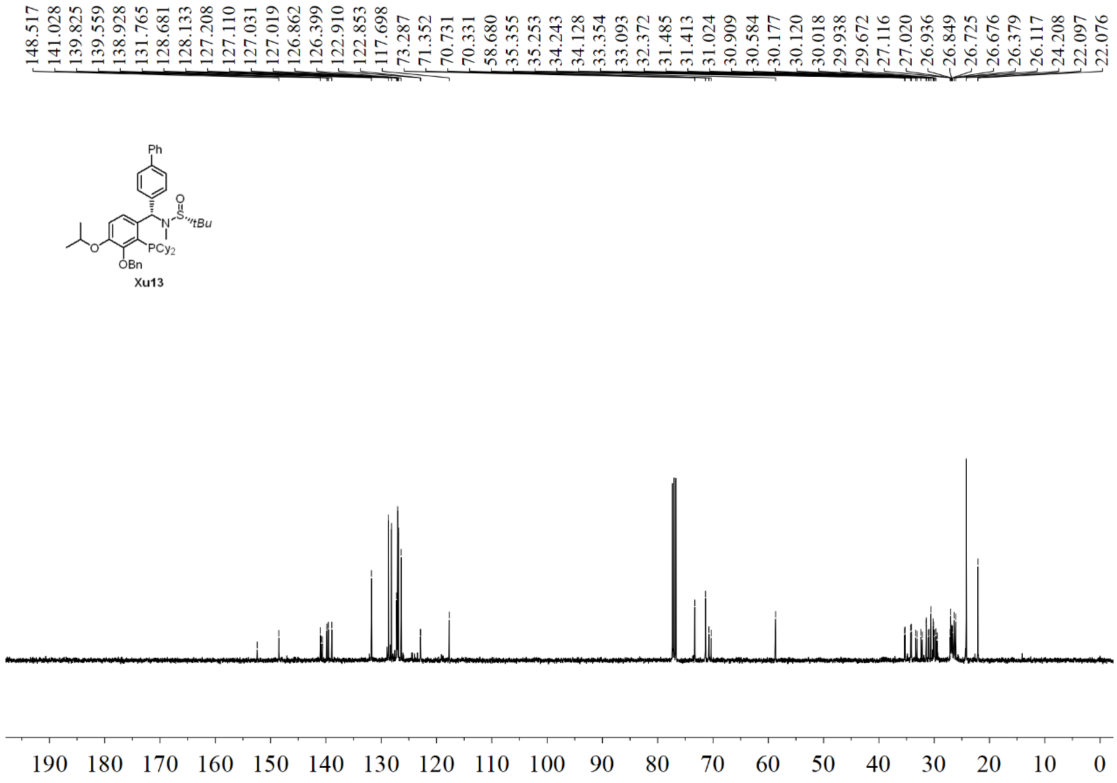
148.517
141.028
139.825
139.559
138.928
131.765
128.681
128.133
127.208
127.110
127.031
127.019
126.862
126.399
122.910
122.853
117.698
73.287
71.352
70.731
70.331
58.680
35.355
35.253
34.243
34.128
33.354
33.093
32.372
31.485
31.413
31.024
30.909
30.584
30.177
30.120
30.018
29.938
29.672
27.116
27.020
26.936
26.849
26.725
26.676
26.379
26.117
24.208
22.097
22.076
Ph
O
S
N
tBu
PCy2
OBn
Xu13
190 180 170 160 150 140 130 120 110 100 90 80 70 60 50 40 30 20 10 0

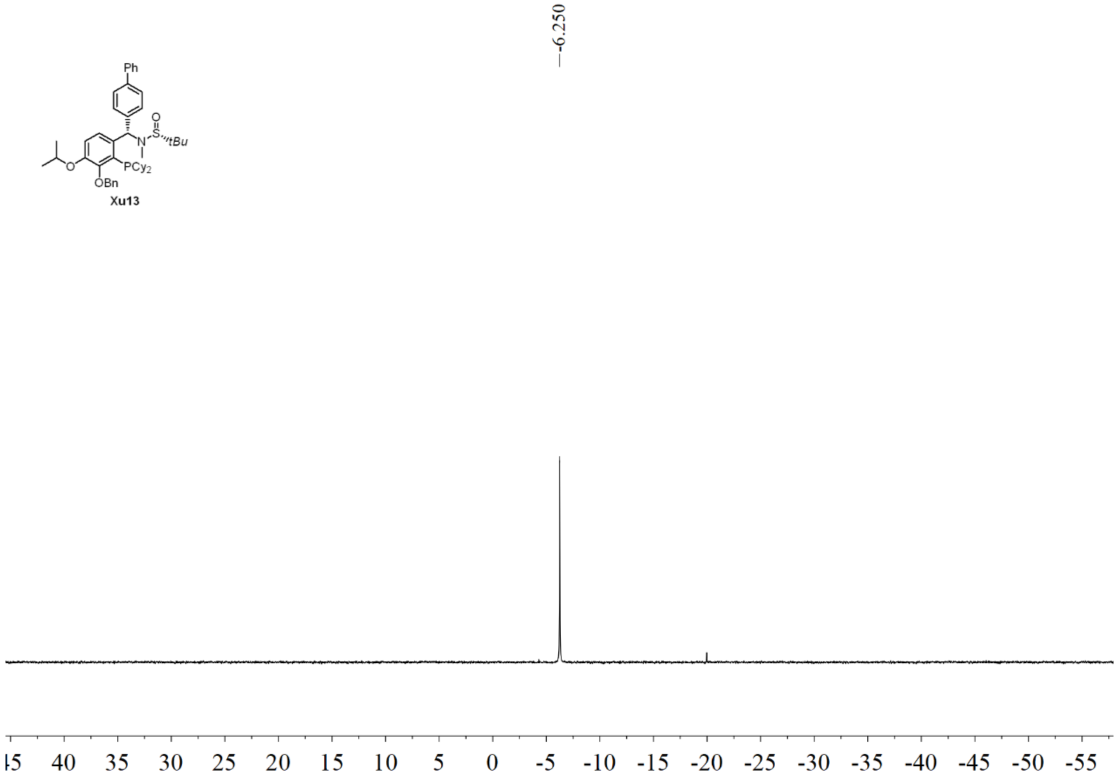
-6.250
Ph
O
S
N
tBu
PCy2
OBn
Xu13
45 40 35 30 25 20 15 10 5 0 -5 -10 -15 -20 -25 -30 -35 -40 -45 -50 -55

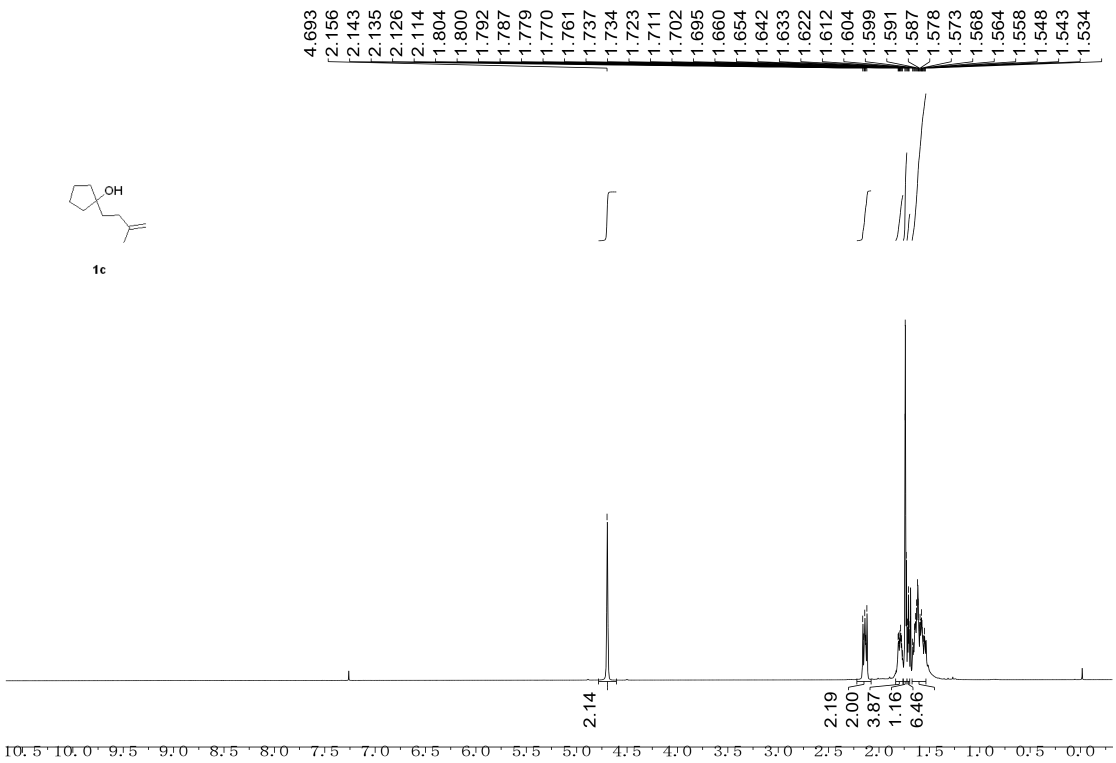
4.693
2.156
2.143
2.135
2.126
2.114
1.804
1.800
1.792
1.787
1.779
1.770
1.761
1.737
1.734
1.723
1.711
1.702
1.695
1.660
1.654
1.642
1.633
1.622
1.612
1.604
1.599
1.591
1.587
1.578
1.573
1.568
1.564
1.558
1.548
1.543
1.534
OH
1c
2.14
2.19
2.00
3.87
1.16
6.46
10.5 10.0 9.5 9.0 8.5 8.0 7.5 7.0 6.5 6.0 5.5 5.0 4.5 4.0 3.5 3.0 2.5 2.0 1.5 1.0 0.5 0.0

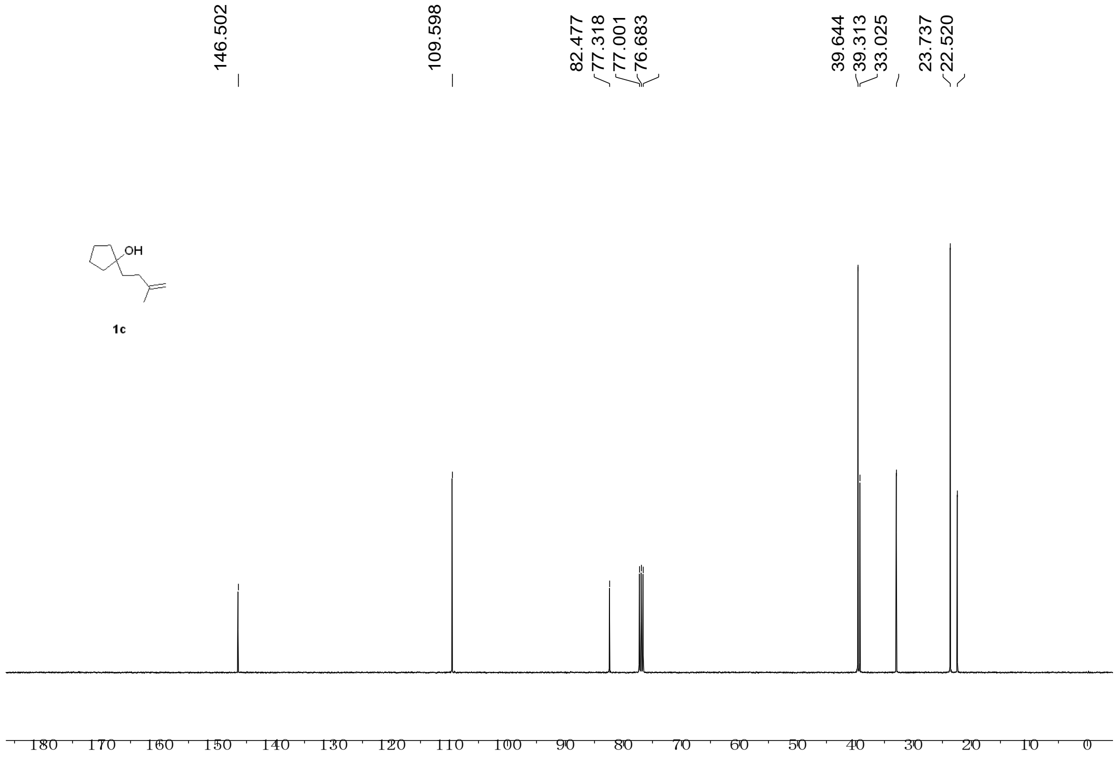
146.502
109.598
82.477
77.318
77.001
76.683
39.644
39.313
33.025
23.737
22.520
OH
1c
180 170 160 150 140 130 120 110 100 90 80 70 60 50 40 30 20 10 0

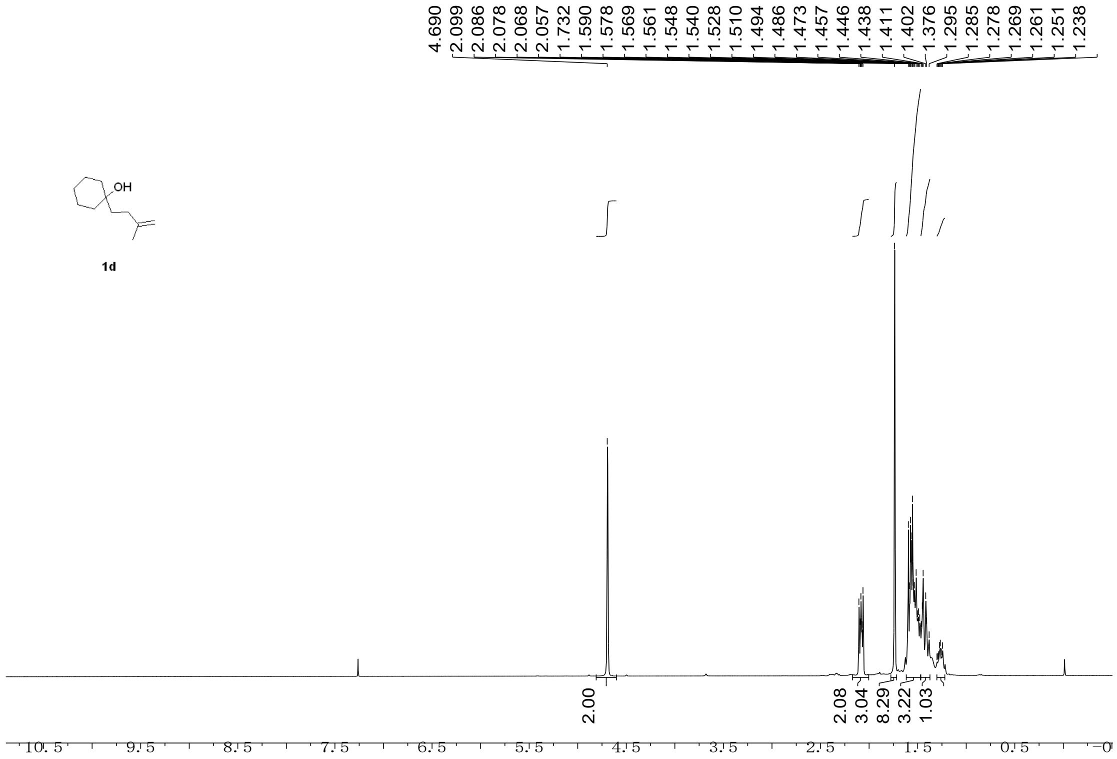
4.690
2.099
2.086
2.078
2.068
2.057
1.732
1.590
1.578
1.569
1.561
1.548
1.540
1.528
1.510
1.494
1.486
1.473
1.457
1.446
1.438
1.411
1.402
1.376
1.295
1.285
1.278
1.269
1.261
1.251
1.238
OH
1d
2.00
2.08
3.04
8.29
3.22
1.03
10.5
9.5
8.5
7.5
6.5
5.5
4.5
3.5
2.5
1.5
0.5
0

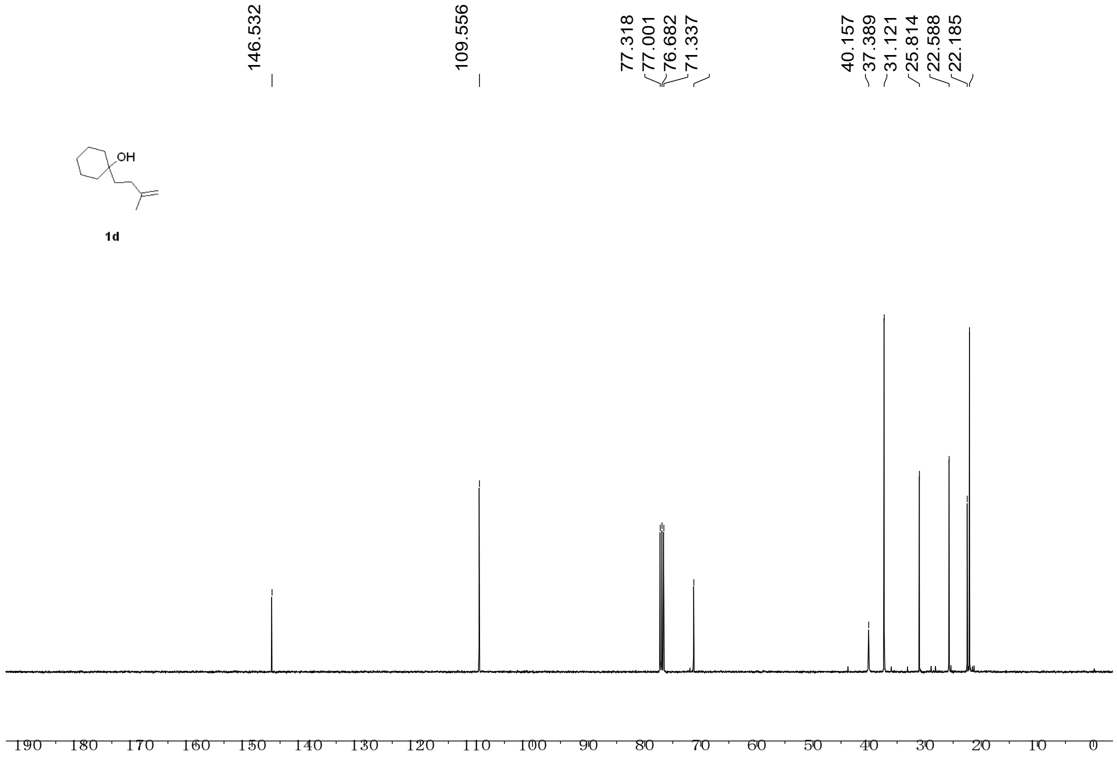
146.532
109.556
77.318
77.001
76.682
71.337
40.157
37.389
31.121
25.814
22.588
22.185
OH
1d
190
180
170
160
150
140
130
120
110
100
90
80
70
60
50
40
30
20
10
0

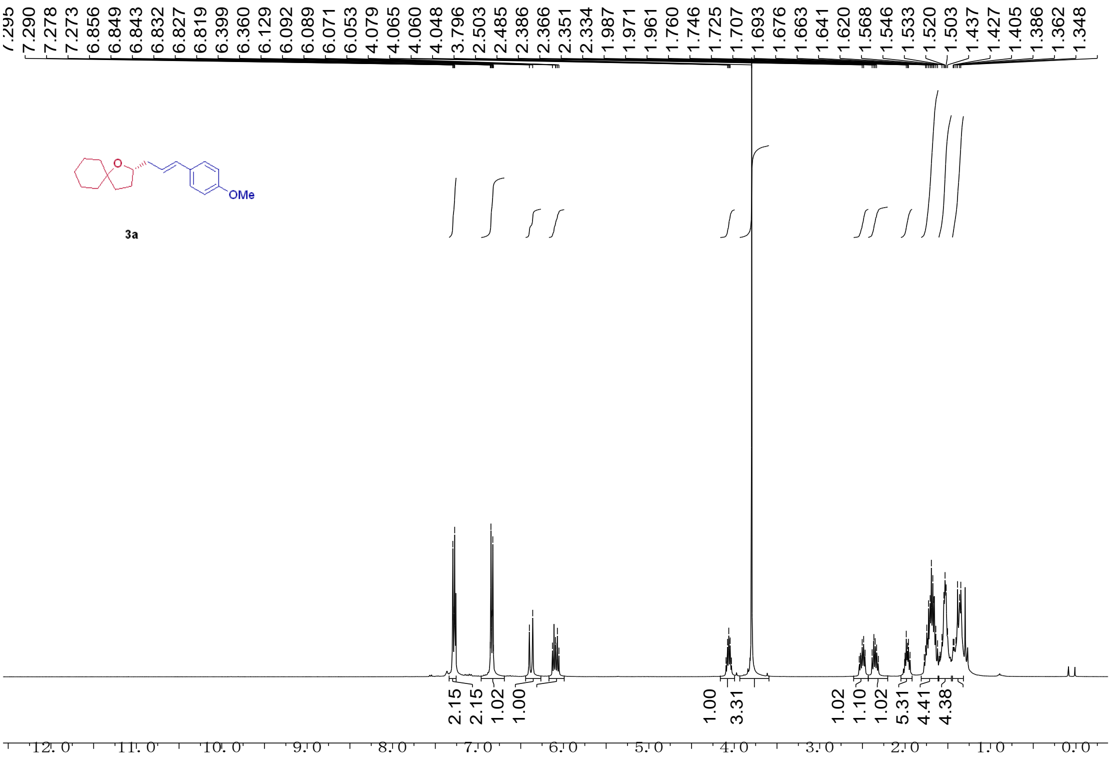
7.295
7.290
7.278
7.273
6.856
6.849
6.843
6.832
6.827
6.819
6.399
6.360
6.129
6.092
6.089
6.071
6.053
4.079
4.065
4.060
4.048
3.796
2.503
2.485
2.386
2.366
2.351
2.334
1.987
1.971
1.961
1.760
1.746
1.725
1.707
1.693
1.676
1.663
1.641
1.620
1.568
1.546
1.533
1.520
1.503
1.437
1.427
1.405
1.386
1.362
1.348
OMe
3a
2.15
2.15
1.02
1.00
1.00
3.31
1.02
1.10
1.02
5.31
4.41
4.38
12.0 11.0 10.0 9.0 8.0 7.0 6.0 5.0 4.0 3.0 2.0 1.0 0.0

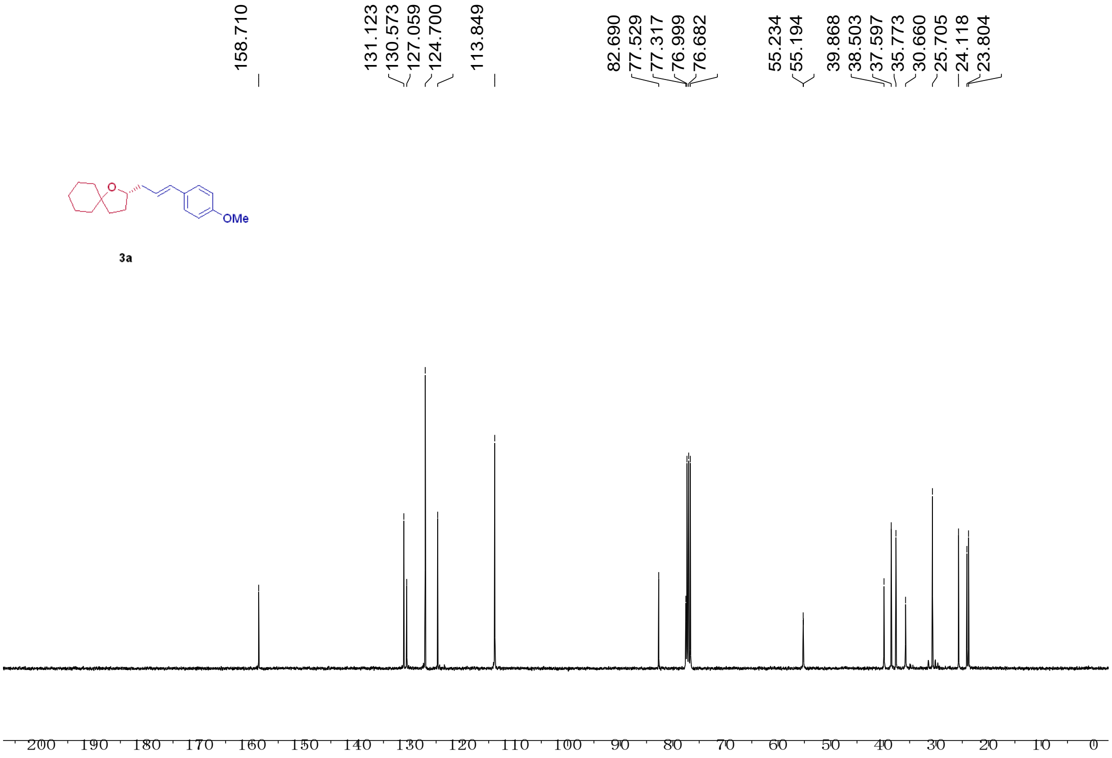
158.710
131.123
130.573
127.059
124.700
113.849
82.690
77.529
77.317
76.999
76.682
55.234
55.194
39.868
38.503
37.597
35.773
30.660
25.705
24.118
23.804
OMe
3a
200 190 180 170 160 150 140 130 120 110 100 90 80 70 60 50 40 30 20 10 0

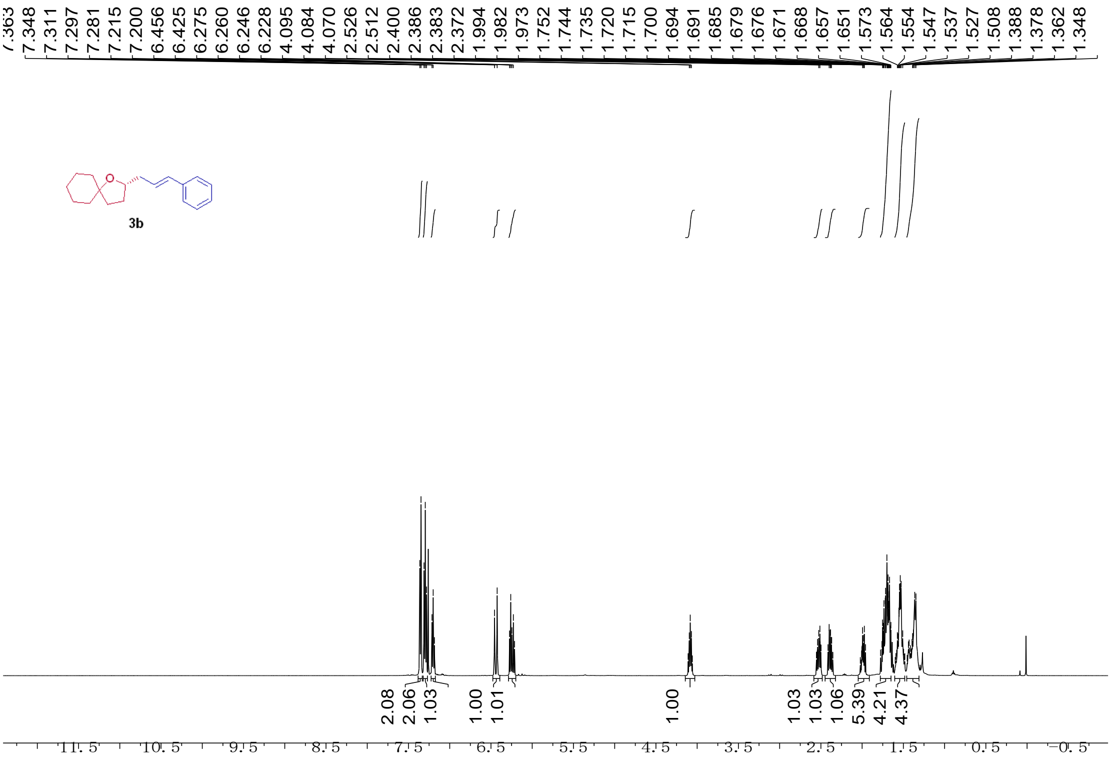

3b
7.363 7.348 7.311 7.297 7.281 7.215 7.200 6.456 6.425 6.275 6.260 6.246 6.228 4.095 4.084 4.070 2.526 2.512 2.400 2.386 2.383 2.372 1.994 1.982 1.973 1.752 1.744 1.735 1.720 1.715 1.700 1.694 1.691 1.685 1.679 1.676 1.671 1.668 1.657 1.651 1.573 1.564 1.554 1.547 1.537 1.527 1.508 1.388 1.378 1.362 1.348
2.08 2.06 1.03 1.00 1.01 1.00 1.03 1.03 1.06 5.39 4.21 4.37
11.5 10.5 9.5 8.5 7.5 6.5 5.5 4.5 3.5 2.5 1.5 0.5 −0.5

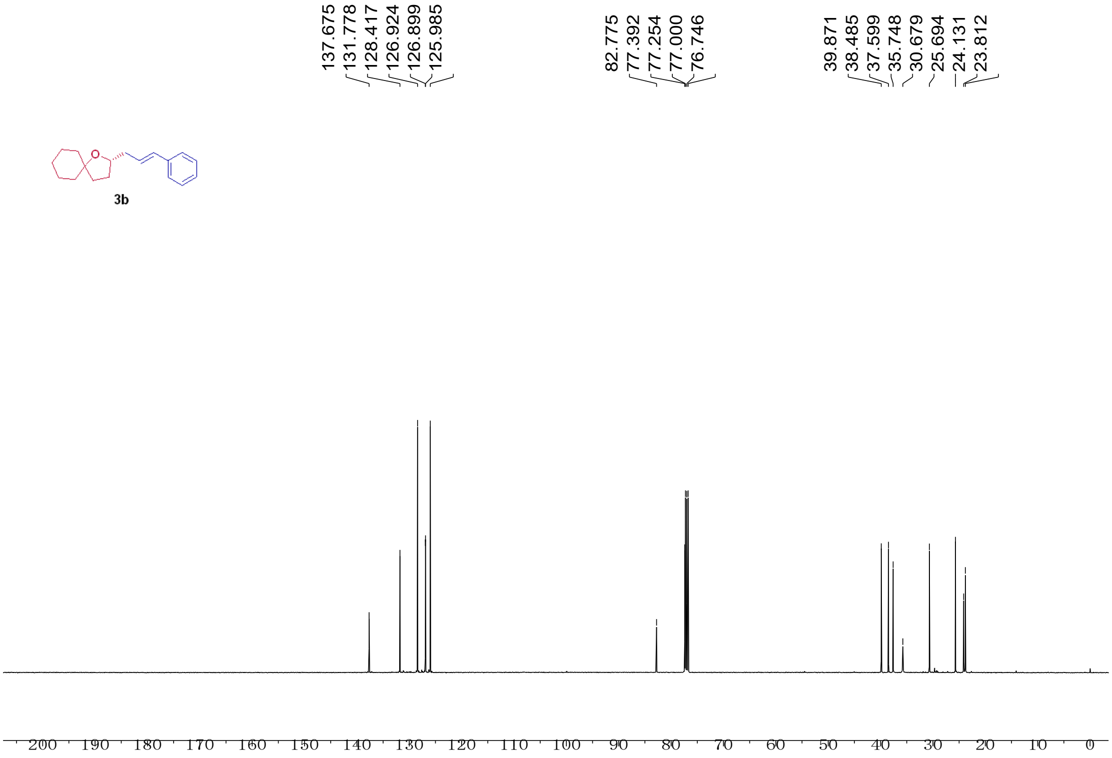

3b
137.675 131.778 128.417 126.924 126.899 125.985
82.775 77.392 77.254 77.000 76.746
39.871 38.485 37.599 35.748 30.679 25.694 24.131 23.812
200 190 180 170 160 150 140 130 120 110 100 90 80 70 60 50 40 30 20 10 0

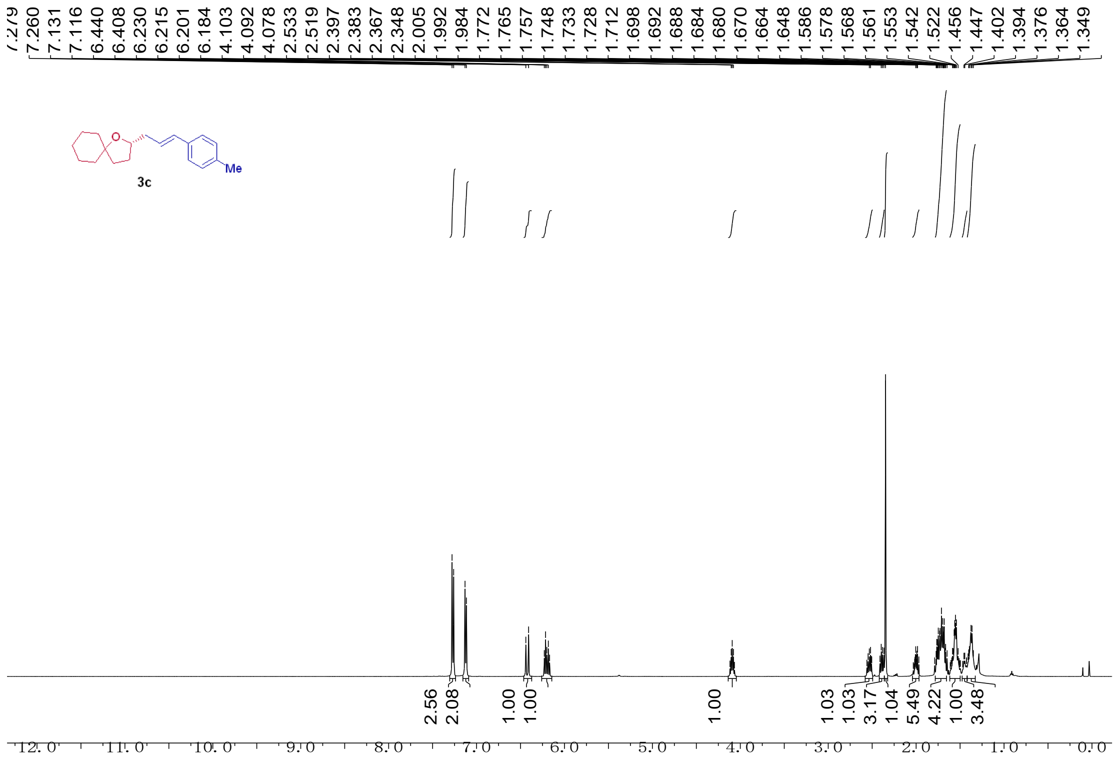
7.279
7.260
7.131
7.116
6.440
6.408
6.230
6.215
6.201
6.184
4.103
4.092
4.078
2.533
2.519
2.397
2.383
2.367
2.348
2.005
1.992
1.984
1.772
1.765
1.757
1.748
1.733
1.728
1.712
1.698
1.692
1.688
1.684
1.680
1.670
1.664
1.648
1.586
1.578
1.568
1.561
1.553
1.542
1.522
1.456
1.447
1.402
1.394
1.376
1.364
1.349
O
Me
3c
2.56
2.08
1.00
1.00
1.00
1.03
1.03
3.17
1.04
5.49
4.22
1.00
3.48
12.0 11.0 10.0 9.0 8.0 7.0 6.0 5.0 4.0 3.0 2.0 1.0 0.0

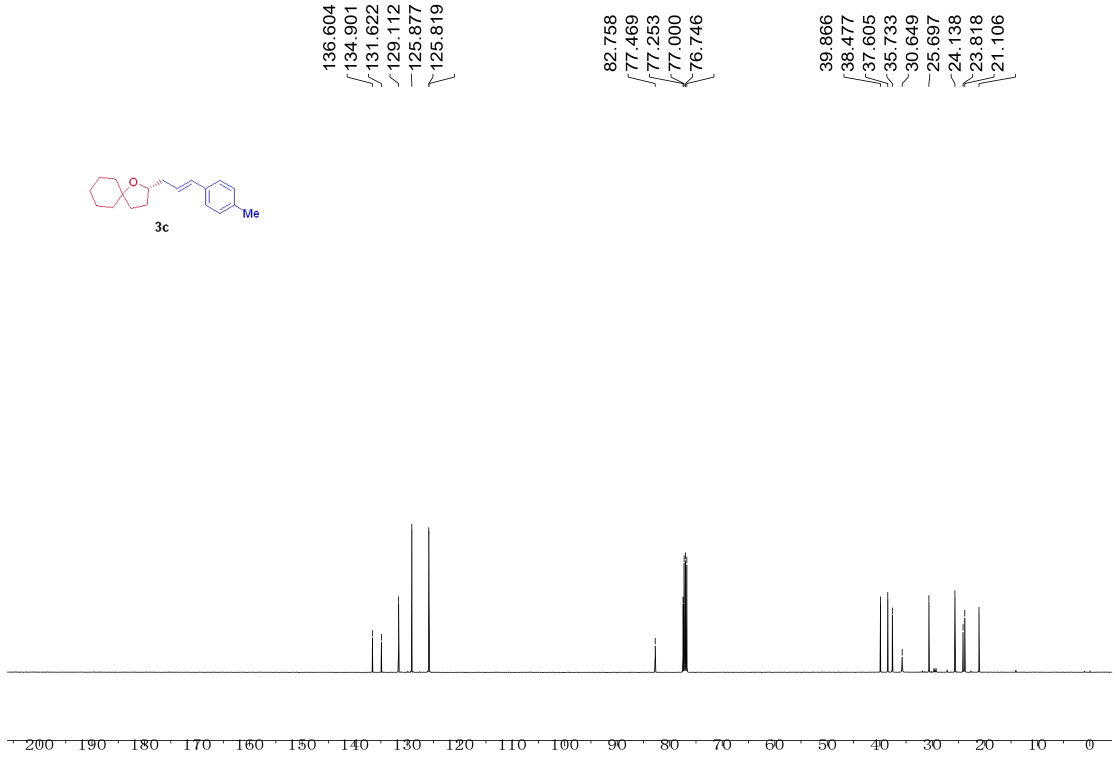
136.604
134.901
131.622
129.112
125.877
125.819
82.758
77.469
77.253
77.000
76.746
39.866
38.477
37.605
35.733
30.649
25.697
24.138
23.818
21.106
O
Me
3c
200 190 180 170 160 150 140 130 120 110 100 90 80 70 60 50 40 30 20 10 0

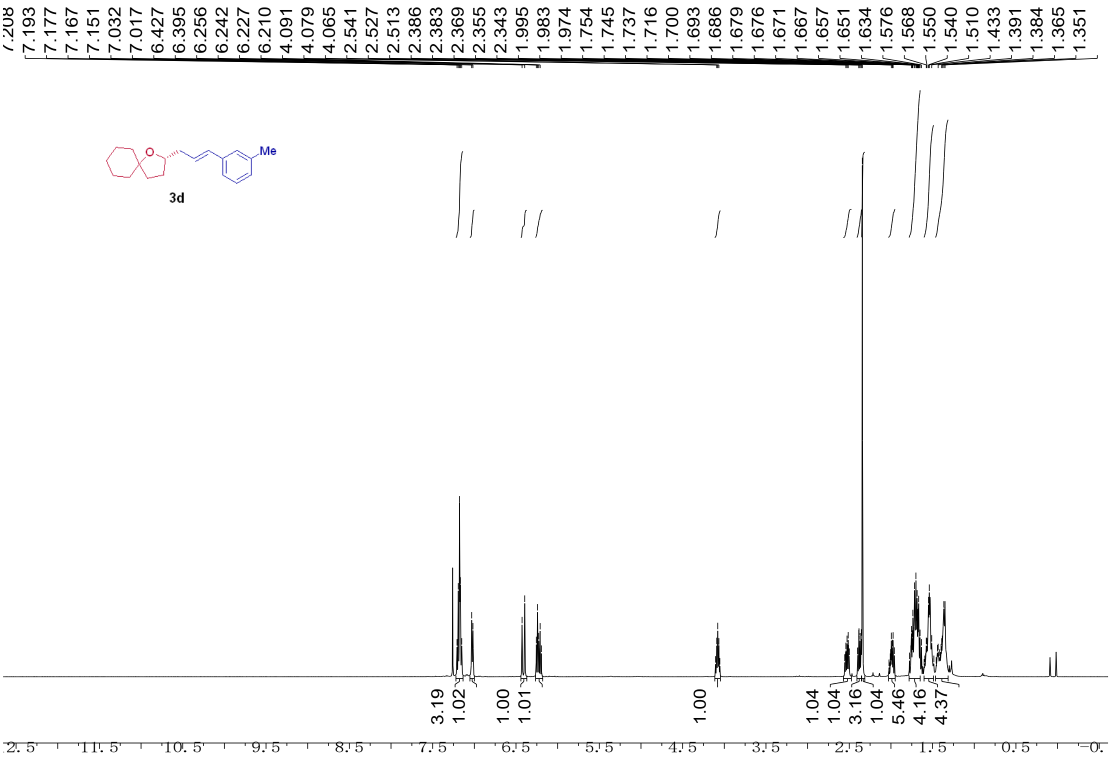
7.193
7.177
7.167
7.151
7.032
7.017
6.427
6.395
6.256
6.242
6.227
6.210
4.091
4.079
4.065
2.541
2.527
2.513
2.386
2.383
2.369
2.355
2.343
1.995
1.983
1.974
1.754
1.745
1.737
1.716
1.700
1.693
1.686
1.679
1.676
1.671
1.667
1.657
1.651
1.634
1.576
1.568
1.550
1.540
1.510
1.433
1.391
1.384
1.365
1.351
Me
O
3d
3.19
1.02
1.00
1.01
1.00
1.04
1.04
3.16
1.04
5.46
4.16
4.37
11.5
10.5
9.5
8.5
7.5
6.5
5.5
4.5
3.5
2.5
1.5
0.5

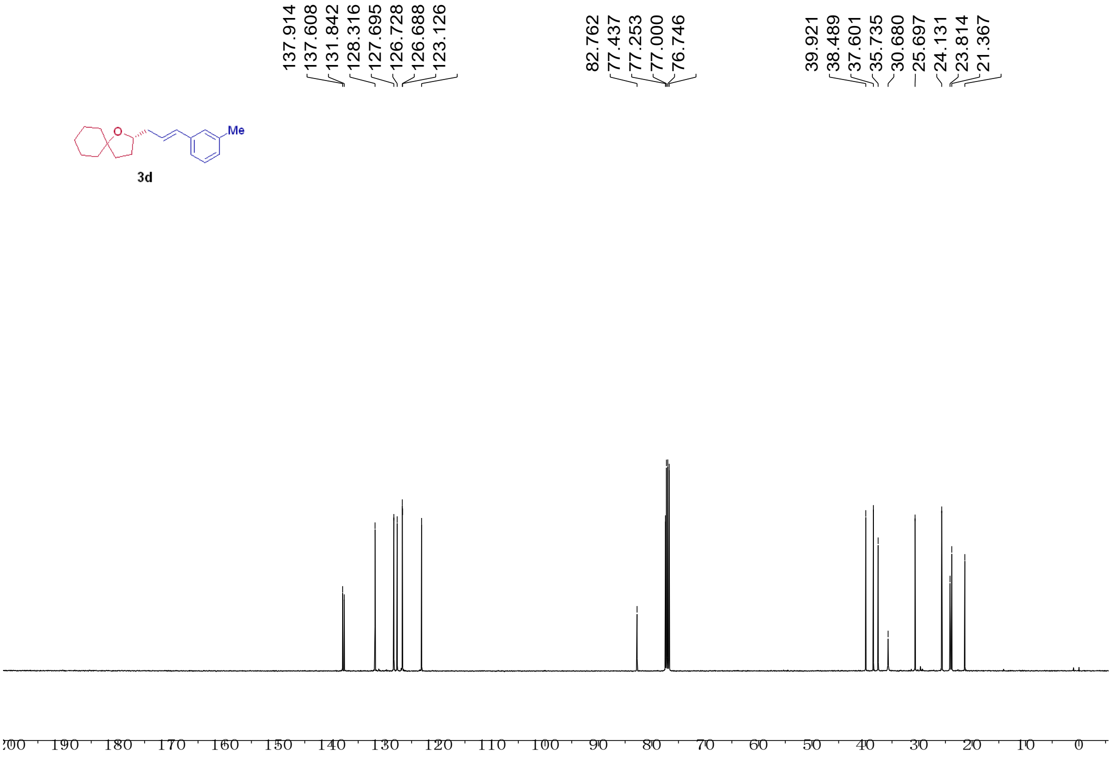
137.914
137.608
131.842
128.316
127.695
126.728
126.688
123.126
82.762
77.437
77.253
77.000
76.746
39.921
38.489
37.601
35.735
30.680
25.697
24.131
23.814
21.367
Me
O
3d
190
180
170
160
150
140
130
120
110
100
90
80
70
60
50
40
30
20
10
0

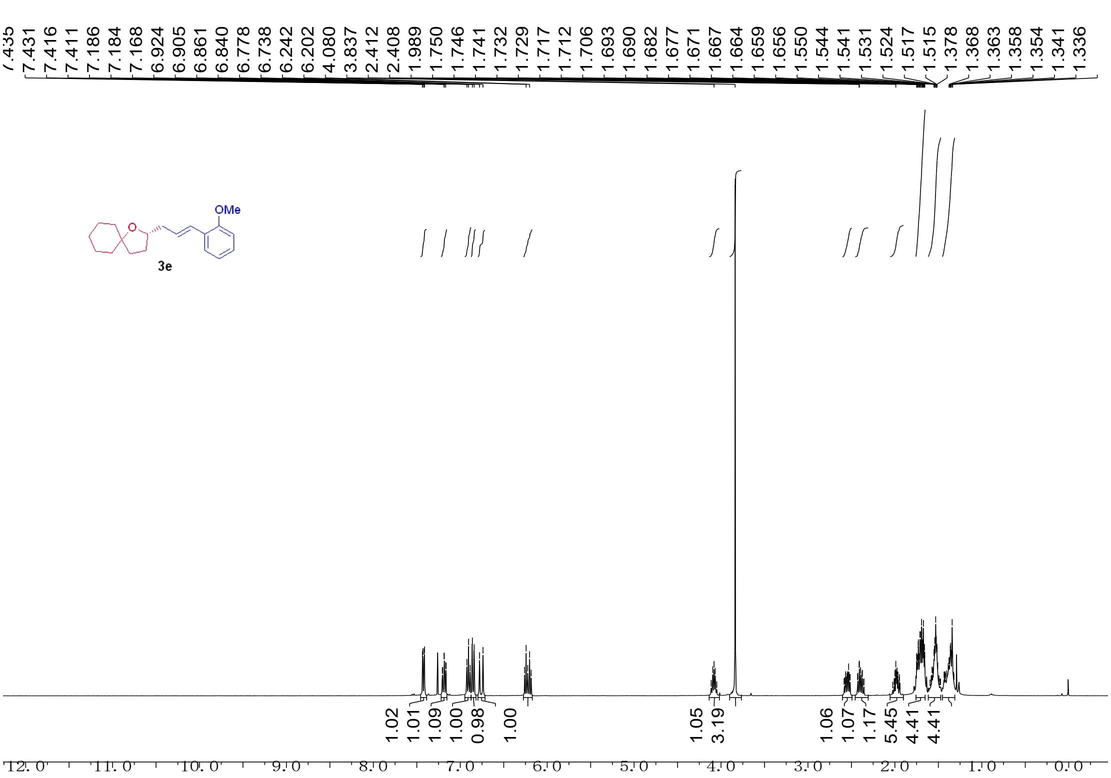
7.435
7.431
7.416
7.411
7.186
7.184
7.168
6.924
6.905
6.861
6.840
6.778
6.738
6.242
6.202
4.080
3.837
2.412
2.408
1.989
1.750
1.746
1.741
1.732
1.729
1.717
1.712
1.706
1.693
1.690
1.682
1.677
1.671
1.667
1.664
1.659
1.656
1.550
1.544
1.541
1.531
1.524
1.517
1.515
1.378
1.368
1.363
1.358
1.354
1.341
1.336
OMe
O
3e
1.02
1.01
1.09
1.00
0.98
1.00
1.05
3.19
1.06
1.07
1.17
5.45
4.41
4.41
12.0
11.0
10.0
9.0
8.0
7.0
6.0
5.0
4.0
3.0
2.0
1.0
0.0

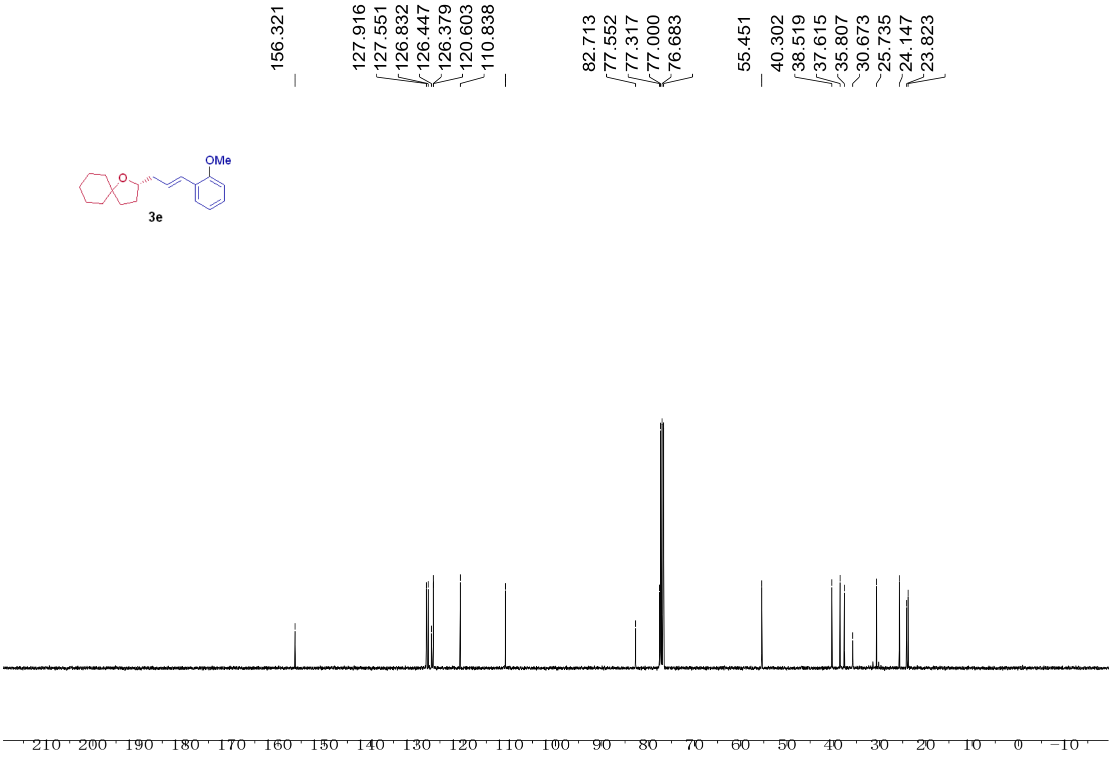
156.321
127.916
127.551
126.832
126.447
126.379
120.603
110.838
82.713
77.552
77.317
77.000
76.683
55.451
40.302
38.519
37.615
35.807
30.673
25.735
24.147
23.823
OMe
O
3e
210
200
190
180
170
160
150
140
130
120
110
100
90
80
70
60
50
40
30
20
10
0
-10

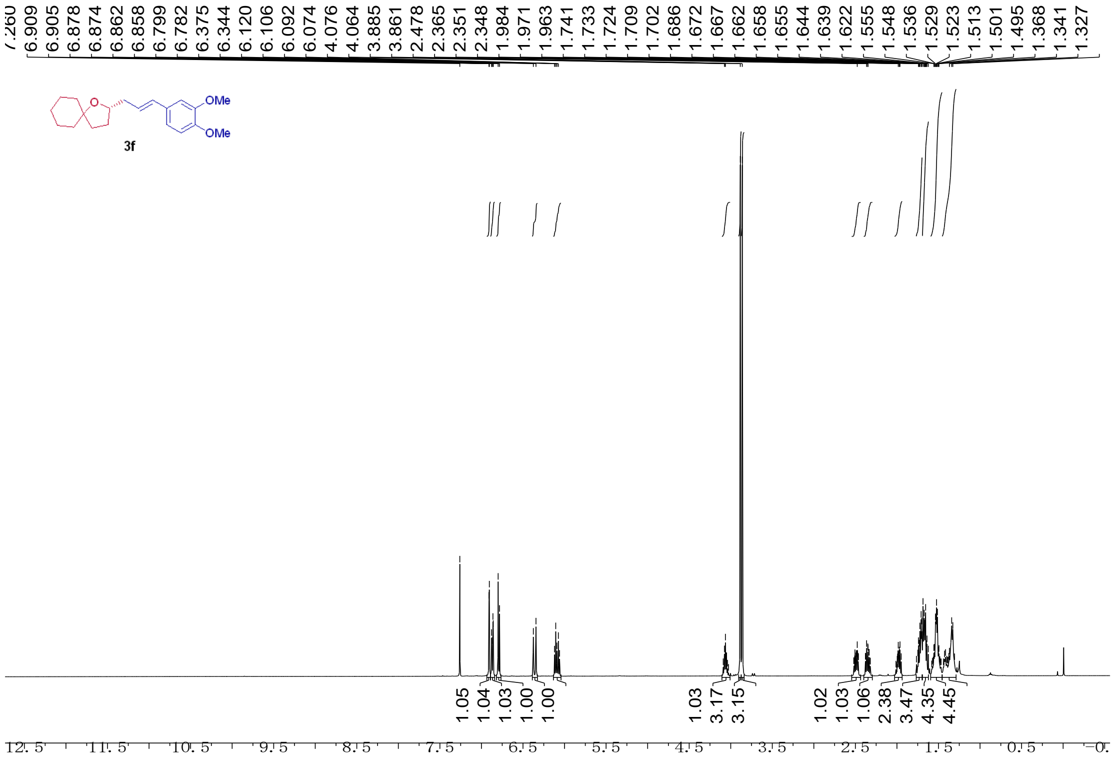

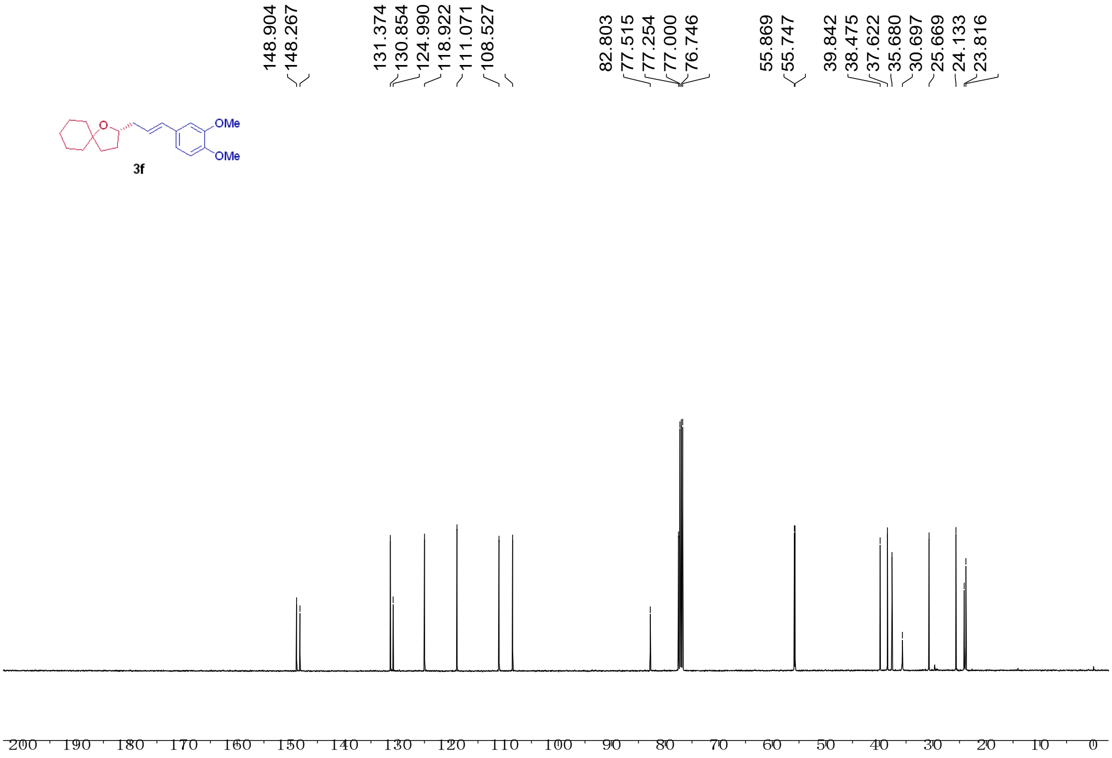

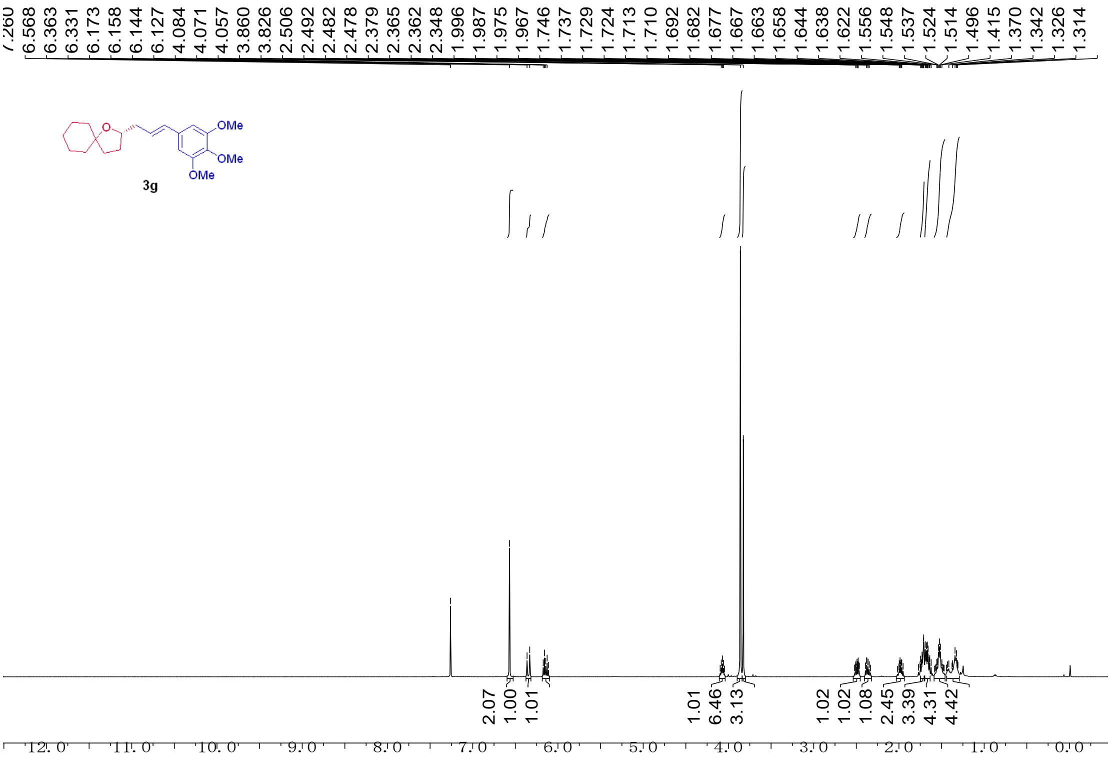
7.260
6.568
6.363
6.331
6.173
6.158
6.144
6.127
4.084
4.071
4.057
3.860
3.826
2.506
2.492
2.482
2.478
2.379
2.365
2.362
2.348
1.996
1.987
1.975
1.967
1.746
1.737
1.729
1.724
1.713
1.710
1.692
1.682
1.677
1.667
1.663
1.658
1.644
1.638
1.622
1.556
1.548
1.537
1.524
1.514
1.496
1.415
1.370
1.342
1.326
1.314
OMe
OMe
OMe
O
3g
2.07
1.00
1.01
1.01
6.46
3.13
1.02
1.02
1.08
2.45
3.39
4.31
4.42
12.0 11.0 10.0 9.0 8.0 7.0 6.0 5.0 4.0 3.0 2.0 1.0 0.0

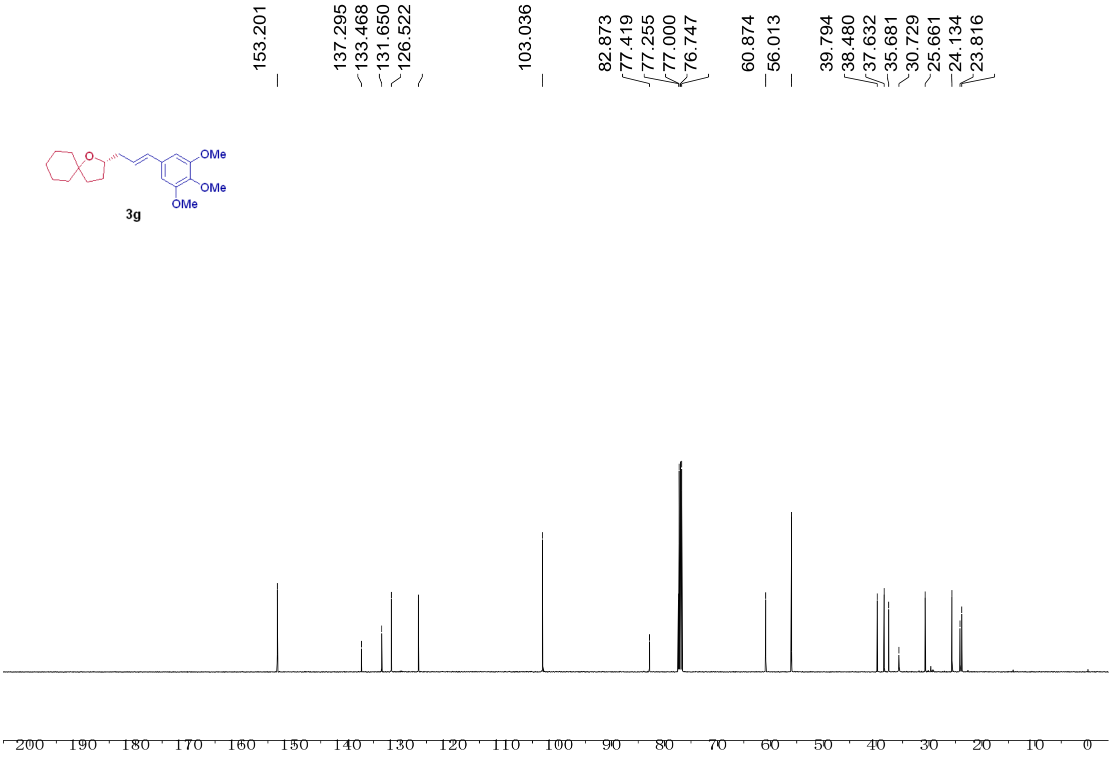
153.201
137.295
133.468
131.650
126.522
103.036
82.873
77.419
77.255
77.000
76.747
60.874
56.013
39.794
38.480
37.632
35.681
30.729
25.661
24.134
23.816
OMe
OMe
OMe
O
3g
200 190 180 170 160 150 140 130 120 110 100 90 80 70 60 50 40 30 20 10 0

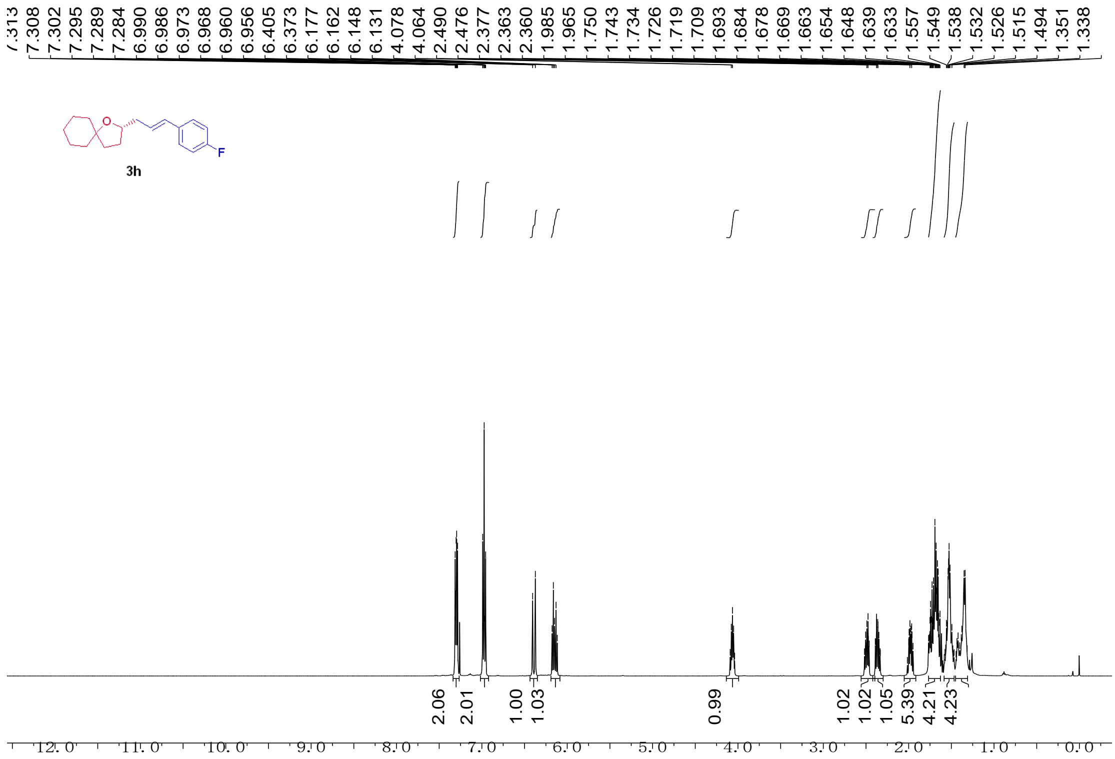
7.313
7.308
7.302
7.295
7.289
7.284
6.990
6.986
6.973
6.968
6.960
6.956
6.405
6.373
6.177
6.162
6.148
6.131
4.078
4.064
2.490
2.476
2.377
2.363
2.360
1.985
1.965
1.750
1.743
1.734
1.726
1.719
1.709
1.693
1.684
1.678
1.669
1.663
1.654
1.648
1.639
1.633
1.557
1.549
1.538
1.532
1.526
1.515
1.494
1.351
1.338
F
3h
2.06
2.01
1.00
1.03
0.99
1.02
1.02
1.05
5.39
4.21
4.23
12.0 11.0 10.0 9.0 8.0 7.0 6.0 5.0 4.0 3.0 2.0 1.0 0.0

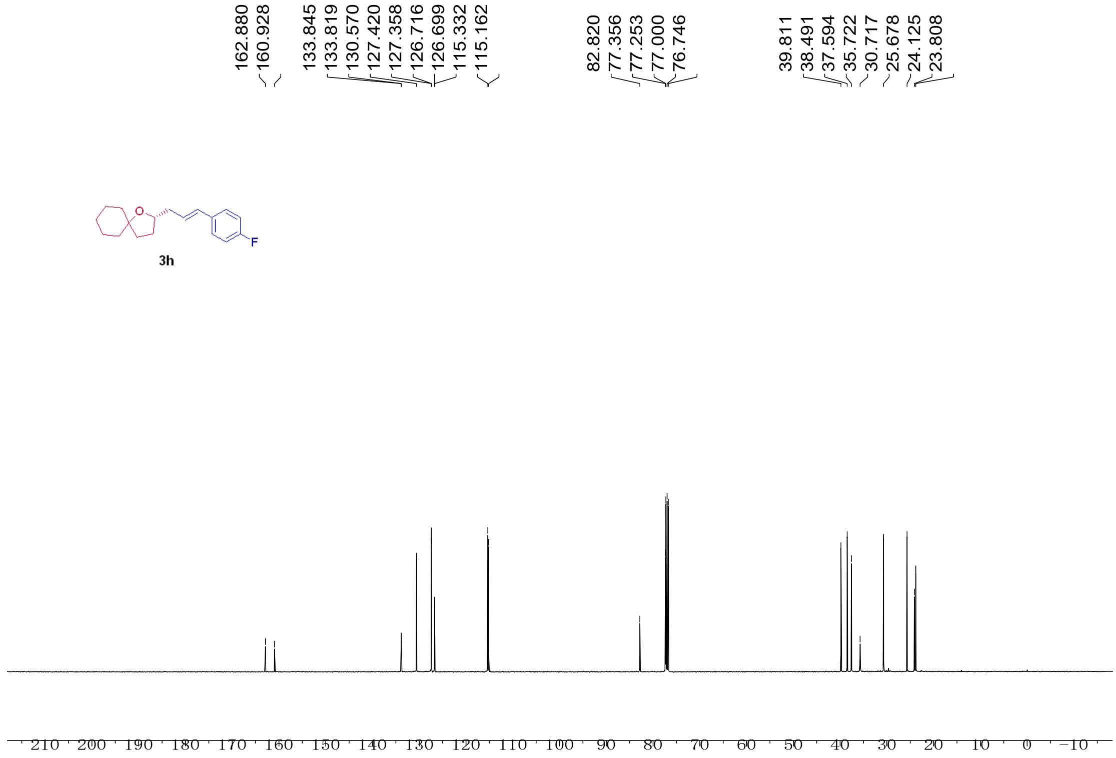
162.880
160.928
133.845
133.819
130.570
127.420
127.358
126.716
126.699
115.332
115.162
82.820
77.356
77.253
77.000
76.746
39.811
38.491
37.594
35.722
30.717
25.678
24.125
23.808
F
3h
210 200 190 180 170 160 150 140 130 120 110 100 90 80 70 60 50 40 30 20 10 0 -10

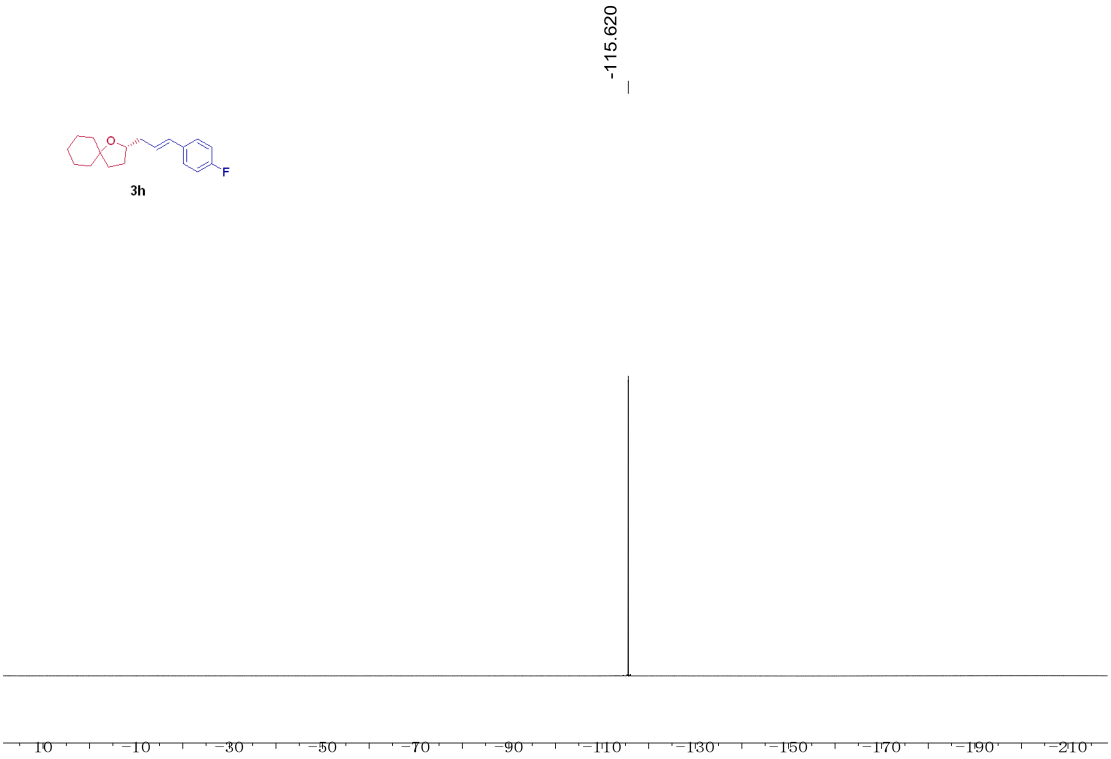
-115.620
3h
10
-10
-30
-50
-70
-90
-110
-130
-150
-170
-190
-210

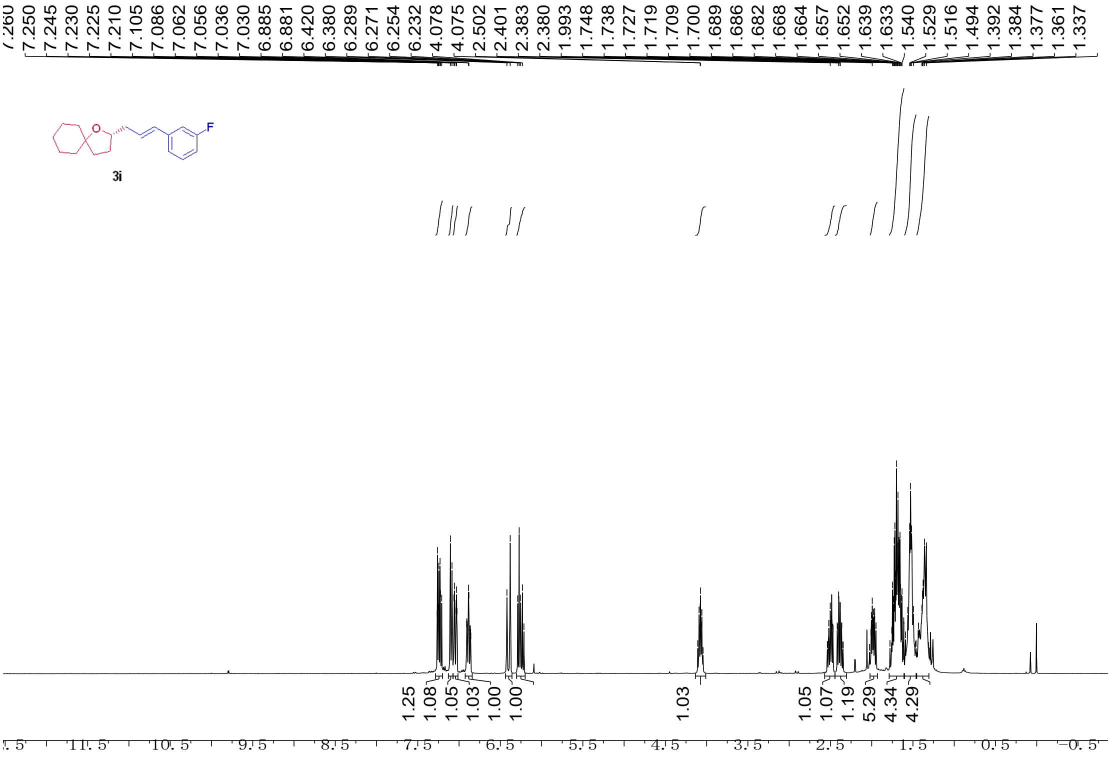
7.250
7.245
7.230
7.225
7.210
7.105
7.086
7.062
7.056
7.036
7.030
6.885
6.881
6.420
6.380
6.289
6.271
6.254
6.232
4.078
4.075
2.502
2.401
2.383
2.380
1.993
1.748
1.738
1.727
1.719
1.709
1.700
1.689
1.686
1.682
1.668
1.664
1.657
1.652
1.639
1.633
1.540
1.529
1.516
1.494
1.392
1.384
1.377
1.361
1.337
3i
1.25
1.08
1.05
1.03
1.00
1.00
1.03
1.05
1.07
1.19
5.29
4.34
4.29
11.5
10.5
9.5
8.5
7.5
6.5
5.5
4.5
3.5
2.5
1.5
0.5

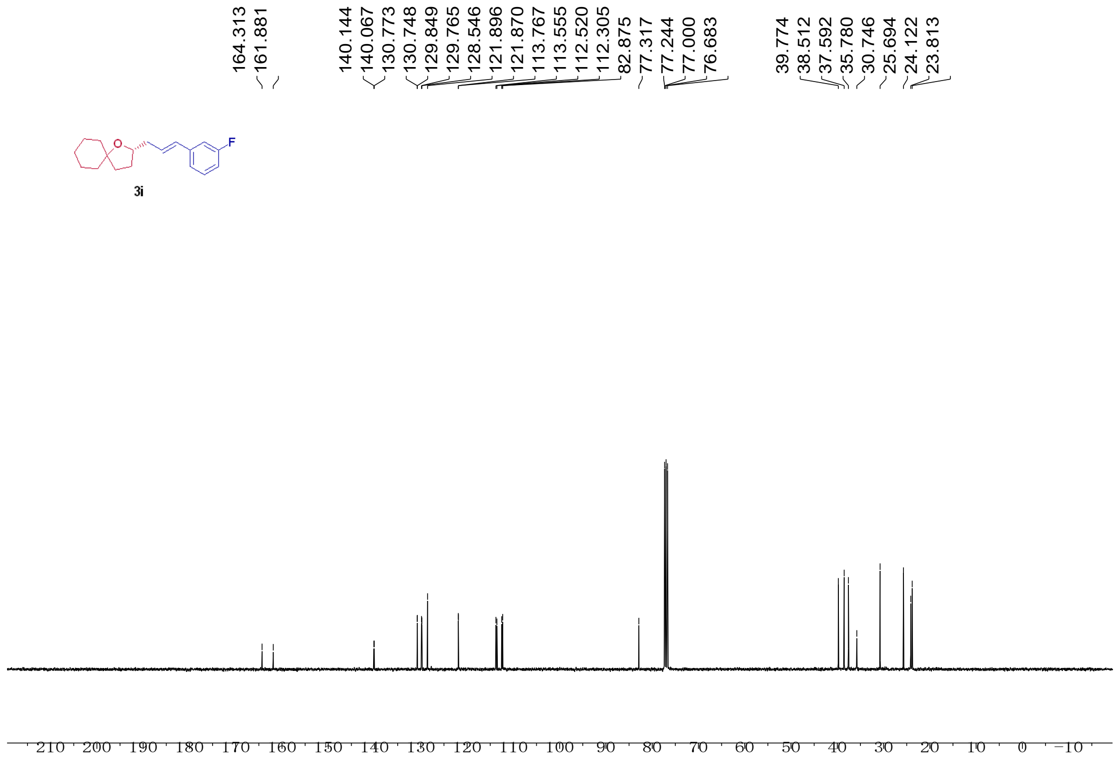
164.313
161.881
140.144
140.067
130.773
130.748
129.849
129.765
128.546
121.896
121.870
113.767
113.555
112.520
112.305
82.875
77.317
77.244
77.000
76.683
39.774
38.512
37.592
35.780
30.746
25.694
24.122
23.813
O
F
3i
210 200 190 180 170 160 150 140 130 120 110 100 90 80 70 60 50 40 30 20 10 0 −10

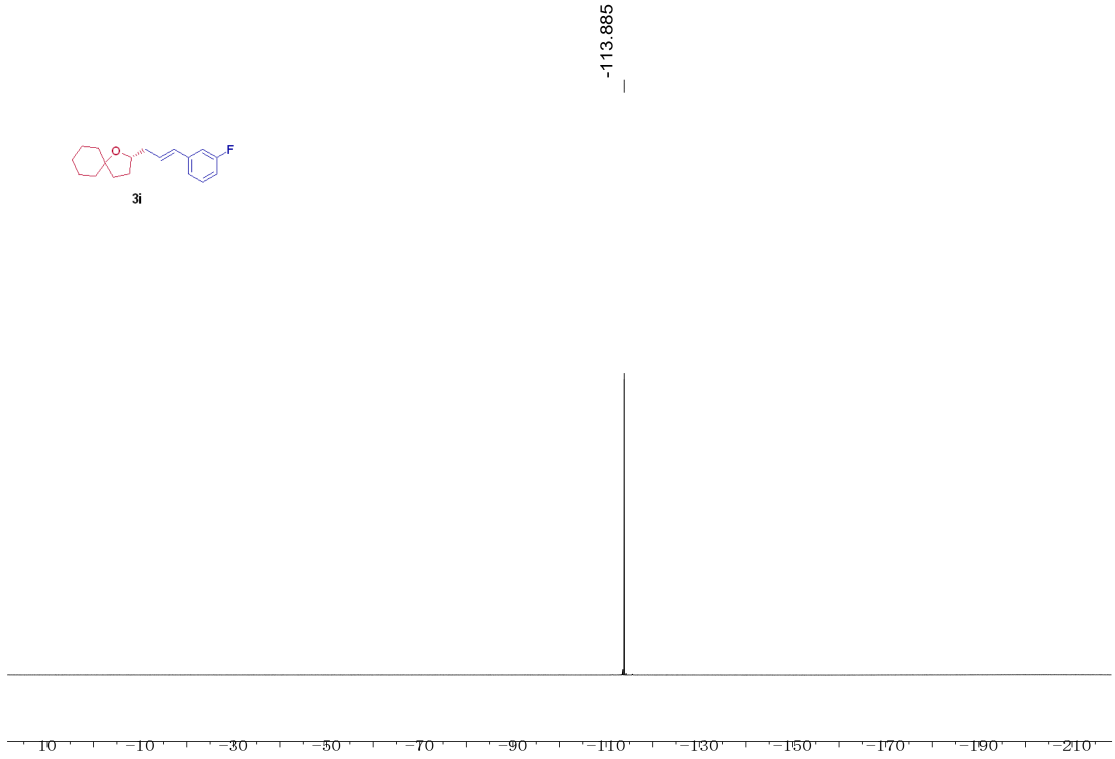
-113.885
O
F
3i
10 −10 −30 −50 −70 −90 −110 −130 −150 −170 −190 −210

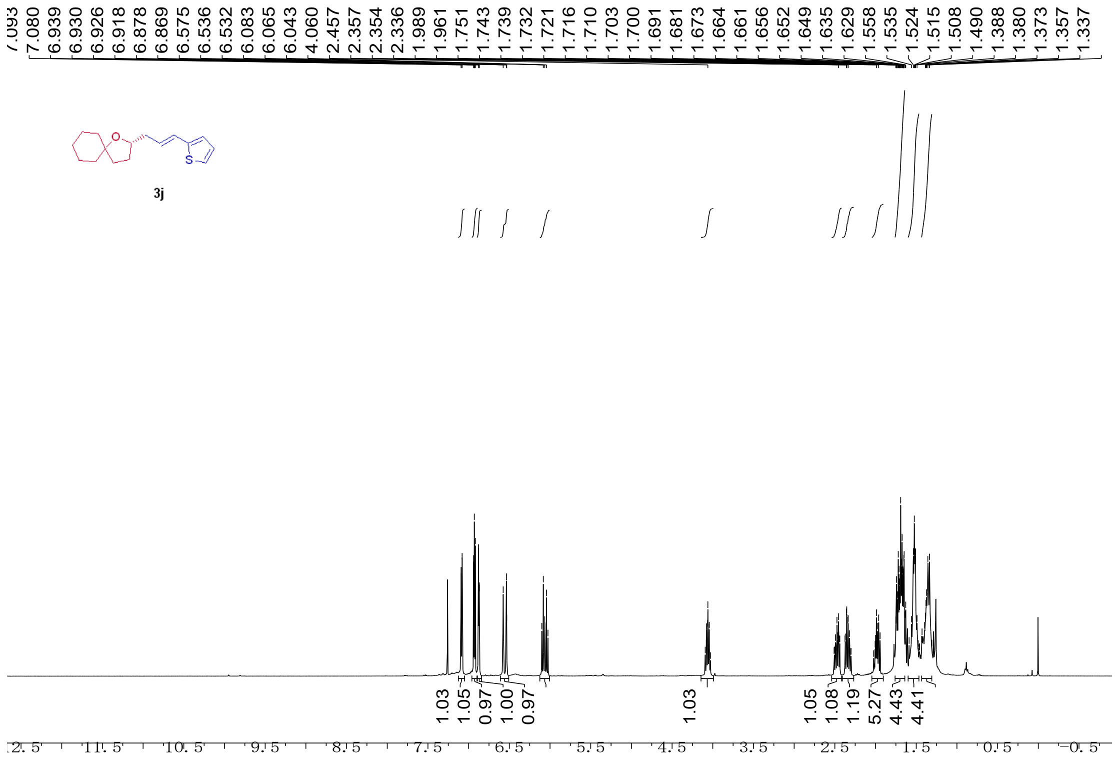
7.093
7.080
6.939
6.930
6.926
6.918
6.878
6.869
6.575
6.536
6.532
6.083
6.065
6.043
4.060
2.457
2.357
2.354
2.336
1.989
1.961
1.751
1.743
1.739
1.732
1.721
1.716
1.710
1.703
1.700
1.691
1.681
1.673
1.664
1.661
1.656
1.652
1.649
1.635
1.629
1.558
1.535
1.524
1.515
1.508
1.490
1.388
1.380
1.373
1.357
1.337
3j
1.03
1.05
0.97
1.00
0.97
1.03
1.05
1.08
1.19
5.27
4.43
4.41

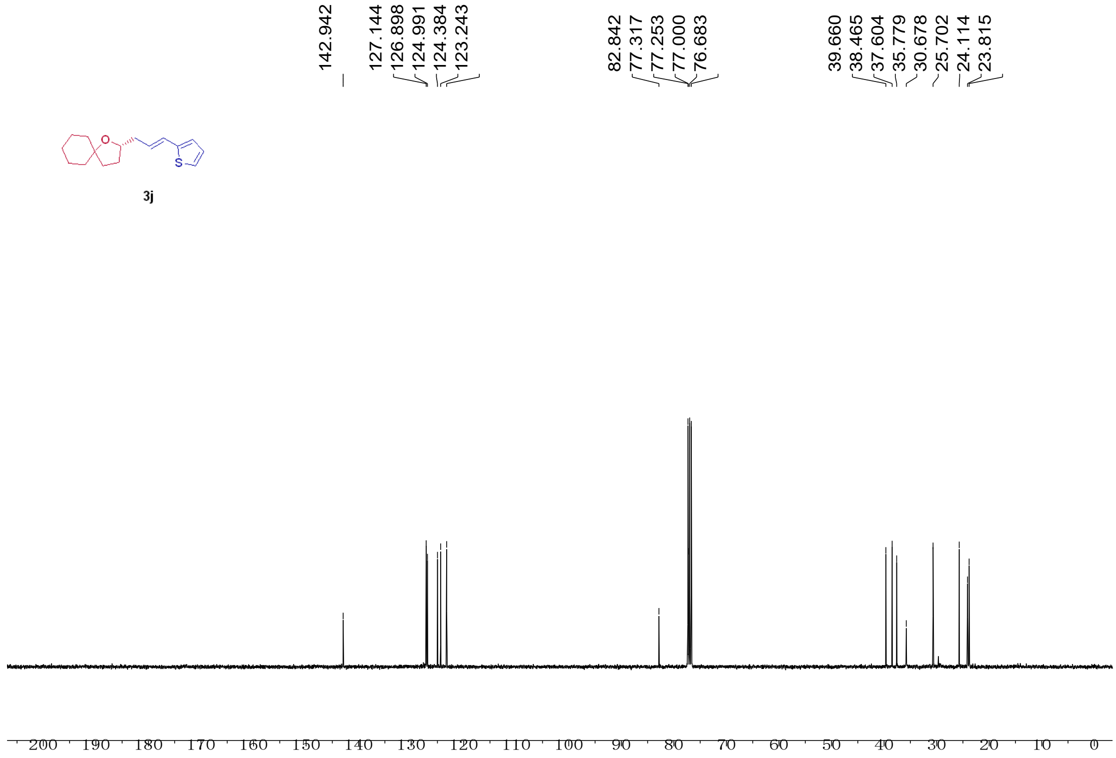
142.942
127.144
126.898
124.991
124.384
123.243
82.842
77.317
77.253
77.000
76.683
39.660
38.465
37.604
35.779
30.678
25.702
24.114
23.815
3j
200
190
180
170
160
150
140
130
120
110
100
90
80
70
60
50
40
30
20
10
0

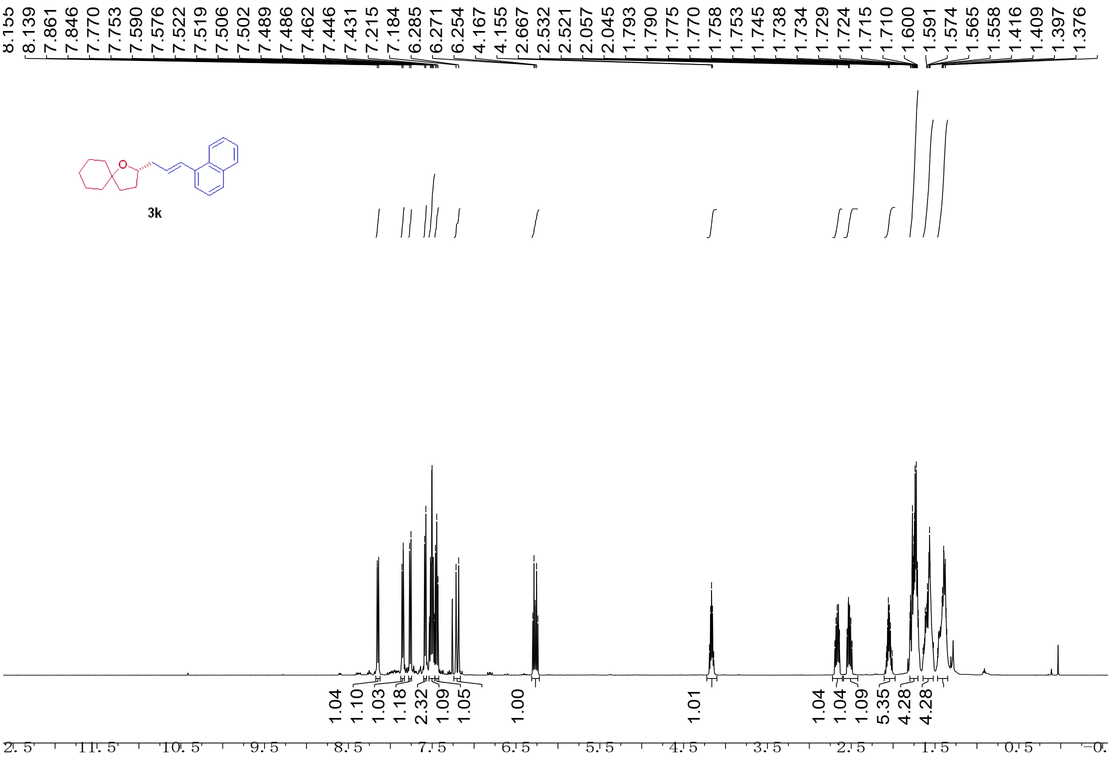
8.155
8.139
7.861
7.846
7.770
7.753
7.590
7.576
7.522
7.519
7.506
7.502
7.489
7.486
7.462
7.446
7.431
7.215
7.184
6.285
6.271
6.254
4.167
4.155
2.667
2.532
2.521
2.057
2.045
1.793
1.790
1.775
1.770
1.758
1.753
1.745
1.738
1.734
1.729
1.724
1.715
1.710
1.600
1.591
1.574
1.565
1.558
1.416
1.409
1.397
1.376
3k
1.04
1.10
1.03
1.18
2.32
1.09
1.05
1.00
1.01
1.04
1.04
1.09
5.35
4.28
4.28
12.5 11.5 10.5 9.5 8.5 7.5 6.5 5.5 4.5 3.5 2.5 1.5 0.5

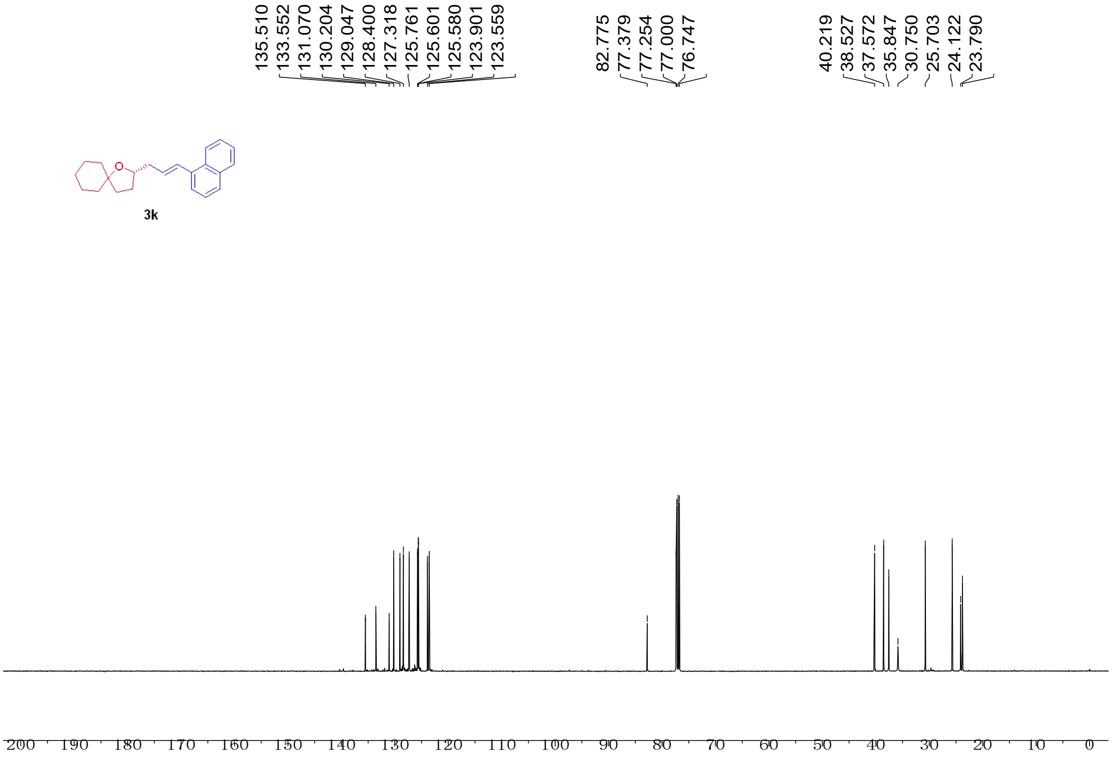
135.510
133.552
131.070
130.204
129.047
128.400
127.318
125.761
125.601
125.580
123.901
123.559
82.775
77.379
77.254
77.000
76.747
40.219
38.527
37.572
35.847
30.750
25.703
24.122
23.790
3k
200 190 180 170 160 150 140 130 120 110 100 90 80 70 60 50 40 30 20 10 0

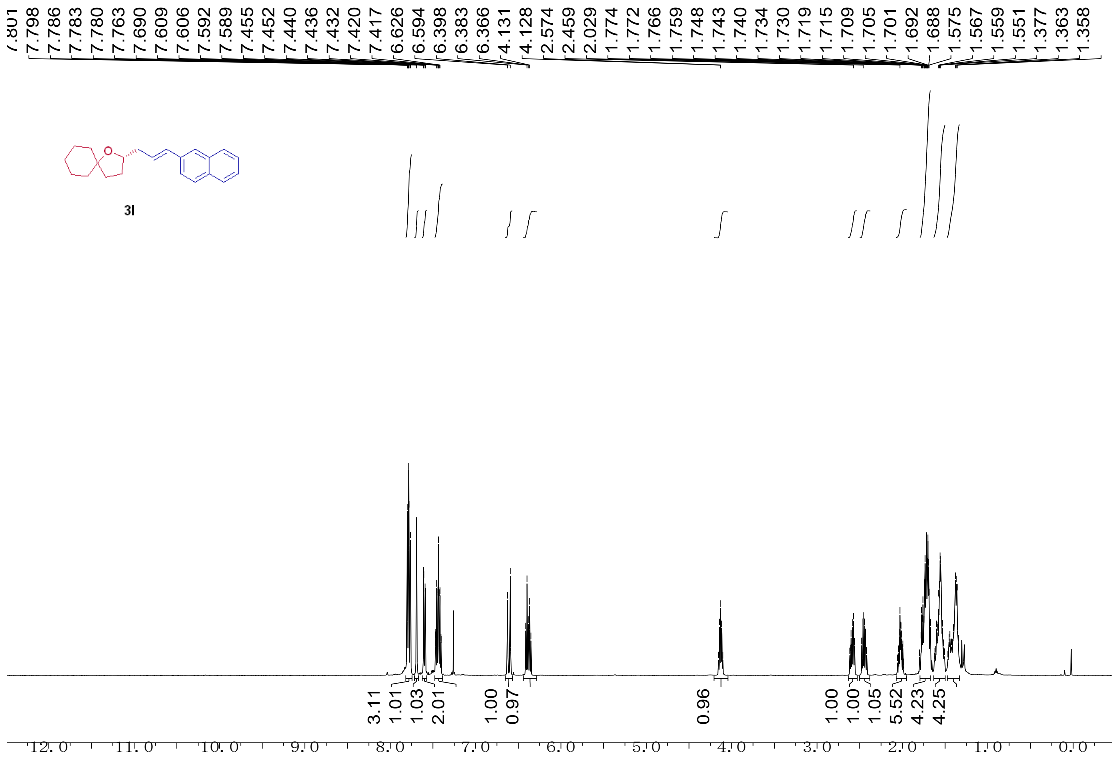

7.801
7.798
7.786
7.783
7.780
7.763
7.690
7.609
7.606
7.592
7.589
7.455
7.452
7.440
7.436
7.432
7.420
7.417
6.626
6.594
6.398
6.383
6.366
4.131
4.128
2.574
2.459
2.029
1.774
1.772
1.766
1.759
1.748
1.743
1.740
1.734
1.730
1.719
1.715
1.709
1.705
1.701
1.692
1.688
1.575
1.567
1.559
1.551
1.377
1.363
1.358
3l
3.11
1.01
1.03
2.01
1.00
0.97
0.96
1.00
1.00
1.05
5.52
4.23
4.25
12.0 11.0 10.0 9.0 8.0 7.0 6.0 5.0 4.0 3.0 2.0 1.0 0.0

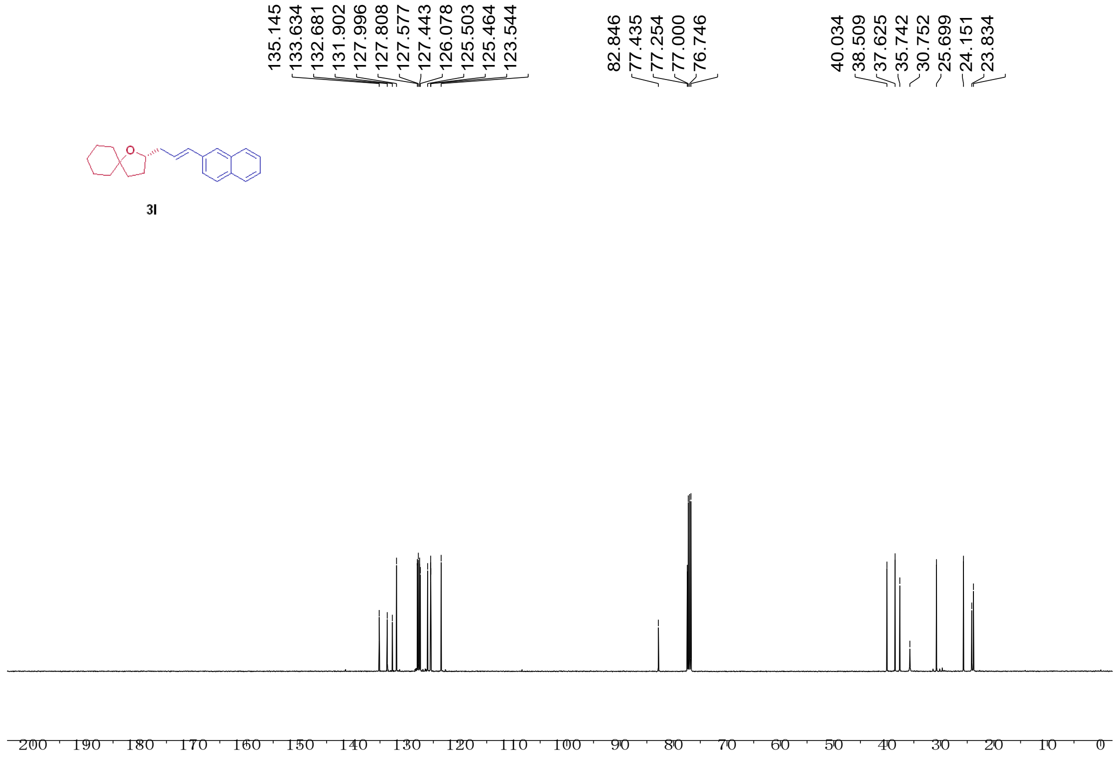

135.145
133.634
132.681
131.902
127.996
127.808
127.577
127.443
126.078
125.503
125.464
123.544
82.846
77.435
77.254
77.000
76.746
40.034
38.509
37.625
35.742
30.752
25.699
24.151
23.834
3l
200 190 180 170 160 150 140 130 120 110 100 90 80 70 60 50 40 30 20 10 0

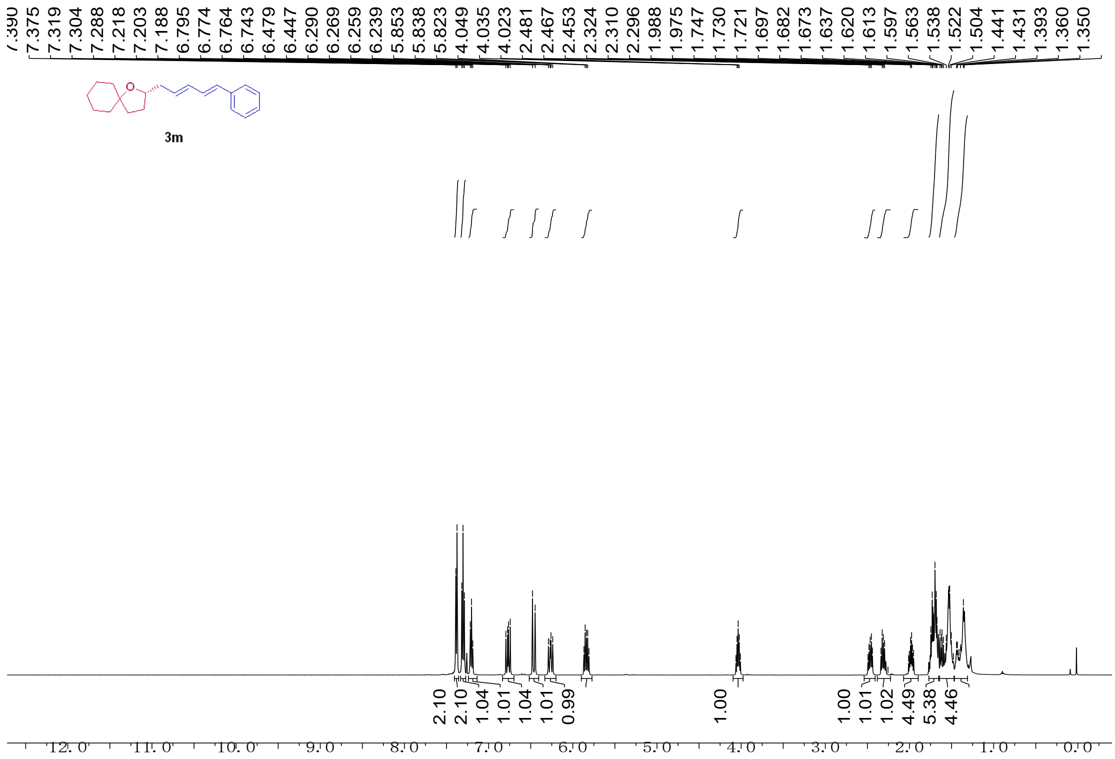
7.390
7.375
7.319
7.304
7.288
7.218
7.203
7.188
6.795
6.774
6.764
6.743
6.479
6.447
6.290
6.269
6.259
6.239
5.853
5.838
5.823
4.049
4.035
4.023
2.481
2.467
2.453
2.324
2.310
2.296
1.988
1.975
1.747
1.730
1.721
1.697
1.682
1.673
1.637
1.620
1.613
1.597
1.563
1.538
1.522
1.504
1.441
1.431
1.393
1.360
1.350
3m
2.10
2.10
1.04
1.01
1.04
1.01
0.99
1.00
1.00
1.01
1.02
4.49
5.38
4.46
12.0
11.0
10.0
9.0
8.0
7.0
6.0
5.0
4.0
3.0
2.0
1.0
0.0

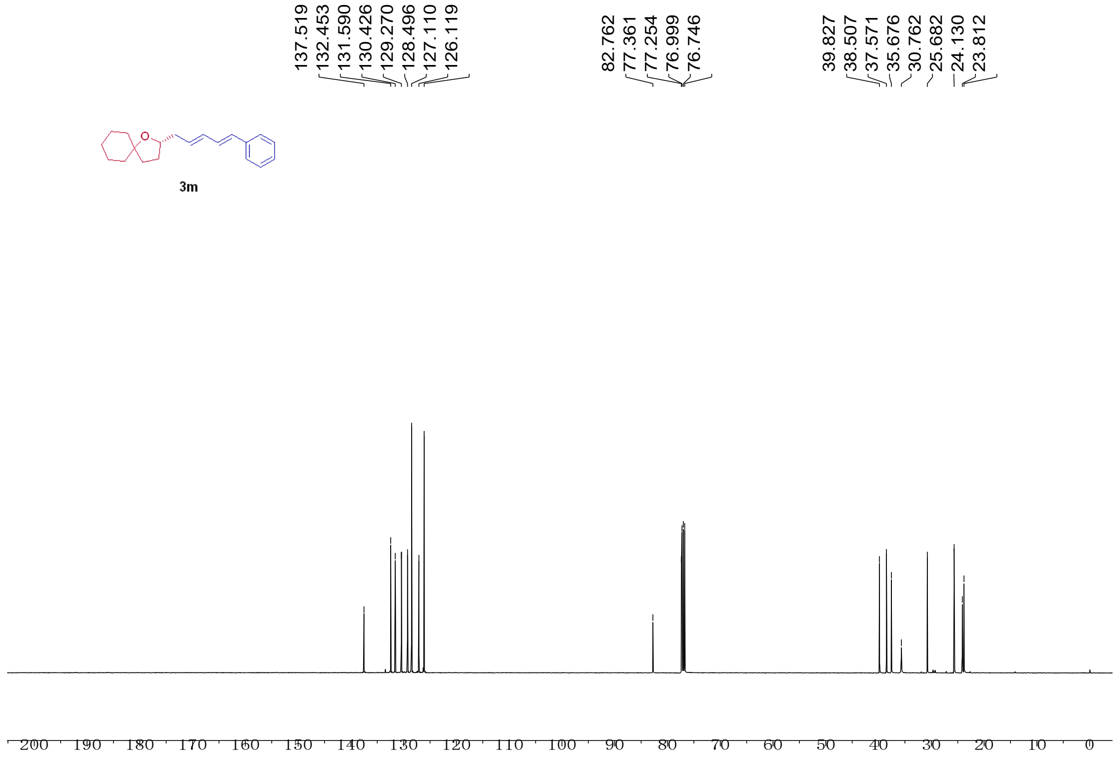
137.519
132.453
131.590
130.426
129.270
128.496
127.110
126.119
82.762
77.361
77.254
76.999
76.746
39.827
38.507
37.571
35.676
30.762
25.682
24.130
23.812
3m
200
190
180
170
160
150
140
130
120
110
100
90
80
70
60
50
40
30
20
10
0

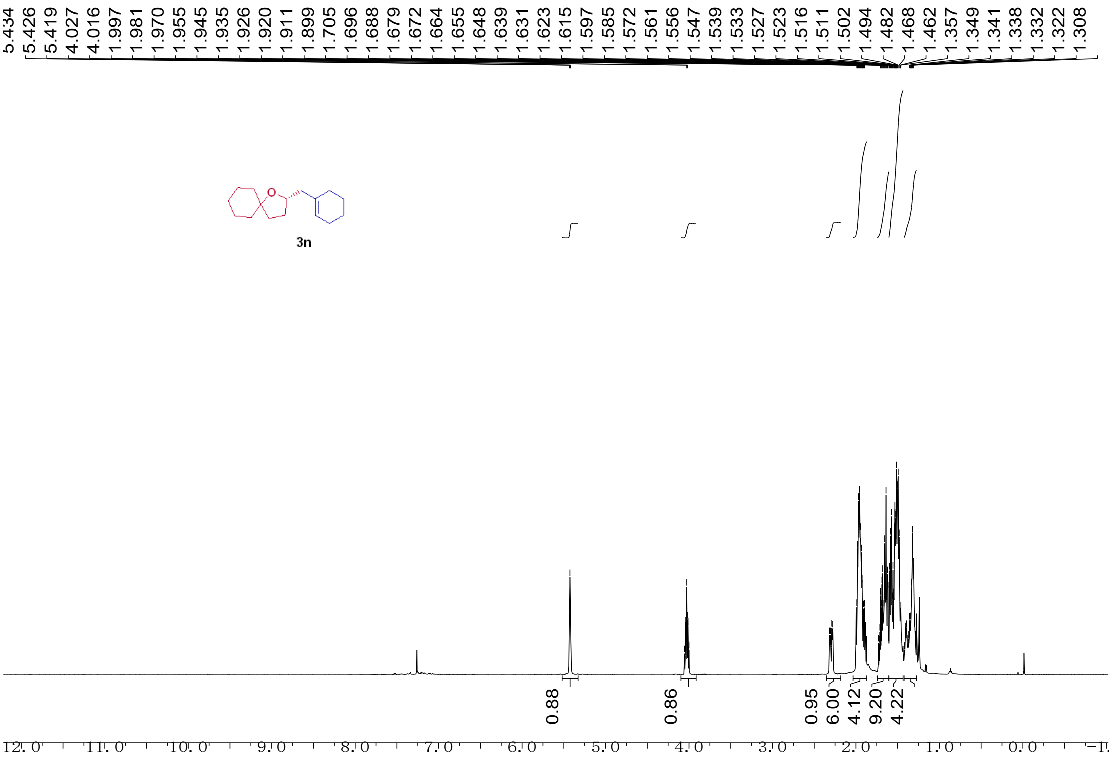
5.434
5.426
5.419
4.027
4.016
1.997
1.981
1.970
1.955
1.945
1.935
1.926
1.920
1.911
1.899
1.705
1.696
1.688
1.679
1.672
1.664
1.655
1.648
1.639
1.631
1.623
1.615
1.597
1.585
1.572
1.561
1.556
1.547
1.539
1.533
1.527
1.523
1.516
1.511
1.502
1.494
1.482
1.468
1.462
1.357
1.349
1.341
1.338
1.332
1.322
1.308
3n
0.88
0.86
0.95
6.00
4.12
9.20
4.22
12.0 11.0 10.0 9.0 8.0 7.0 6.0 5.0 4.0 3.0 2.0 1.0 0.0

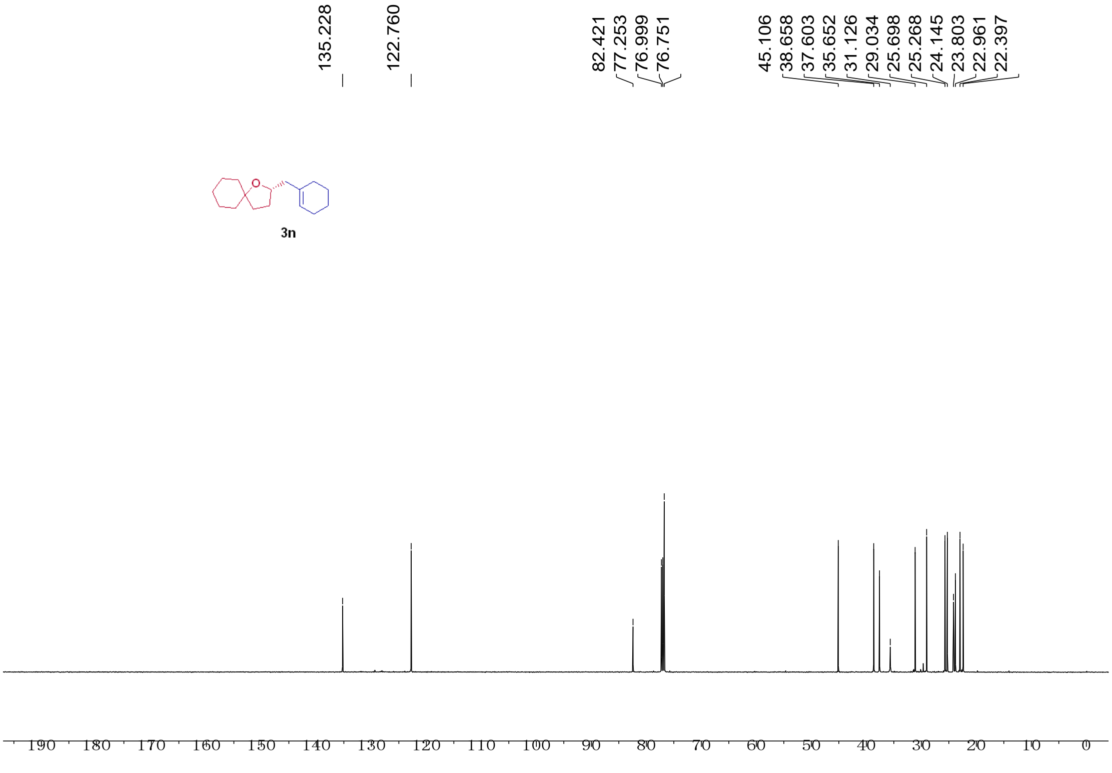
135.228
122.760
82.421
77.253
76.999
76.751
45.106
38.658
37.603
35.652
31.126
29.034
25.698
25.268
24.145
23.803
22.961
22.397
3n
190 180 170 160 150 140 130 120 110 100 90 80 70 60 50 40 30 20 10 0

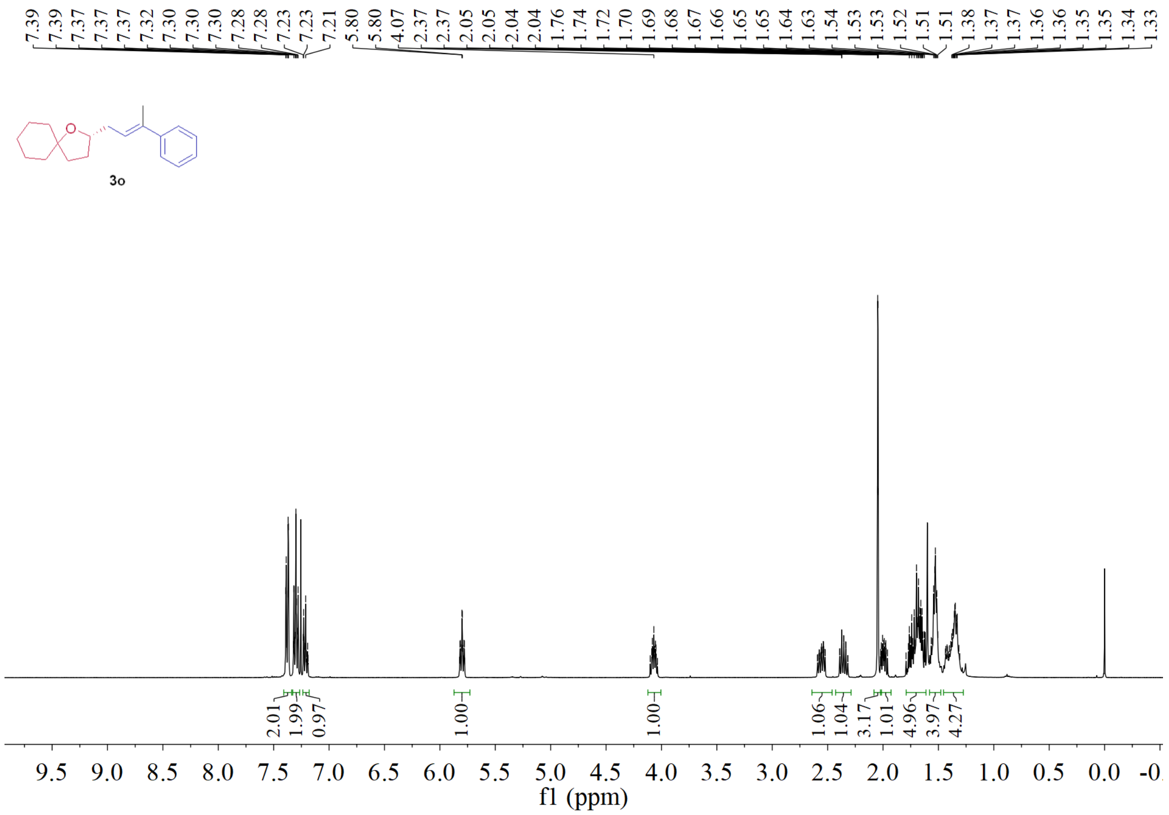
7.39 7.39 7.37 7.37 7.37 7.32 7.30 7.30 7.30 7.28 7.28 7.23 7.23 7.21 5.80 5.80 4.07 2.37 2.37 2.05 2.05 2.04 2.04 1.76 1.74 1.72 1.70 1.69 1.68 1.67 1.66 1.65 1.65 1.64 1.63 1.54 1.53 1.53 1.52 1.51 1.51 1.38 1.37 1.37 1.36 1.36 1.35 1.35 1.34 1.33
3o
2.01 1.99 0.97 1.00 1.00 1.06 1.04 3.17 1.01 4.96 3.97 4.27
f1 (ppm)

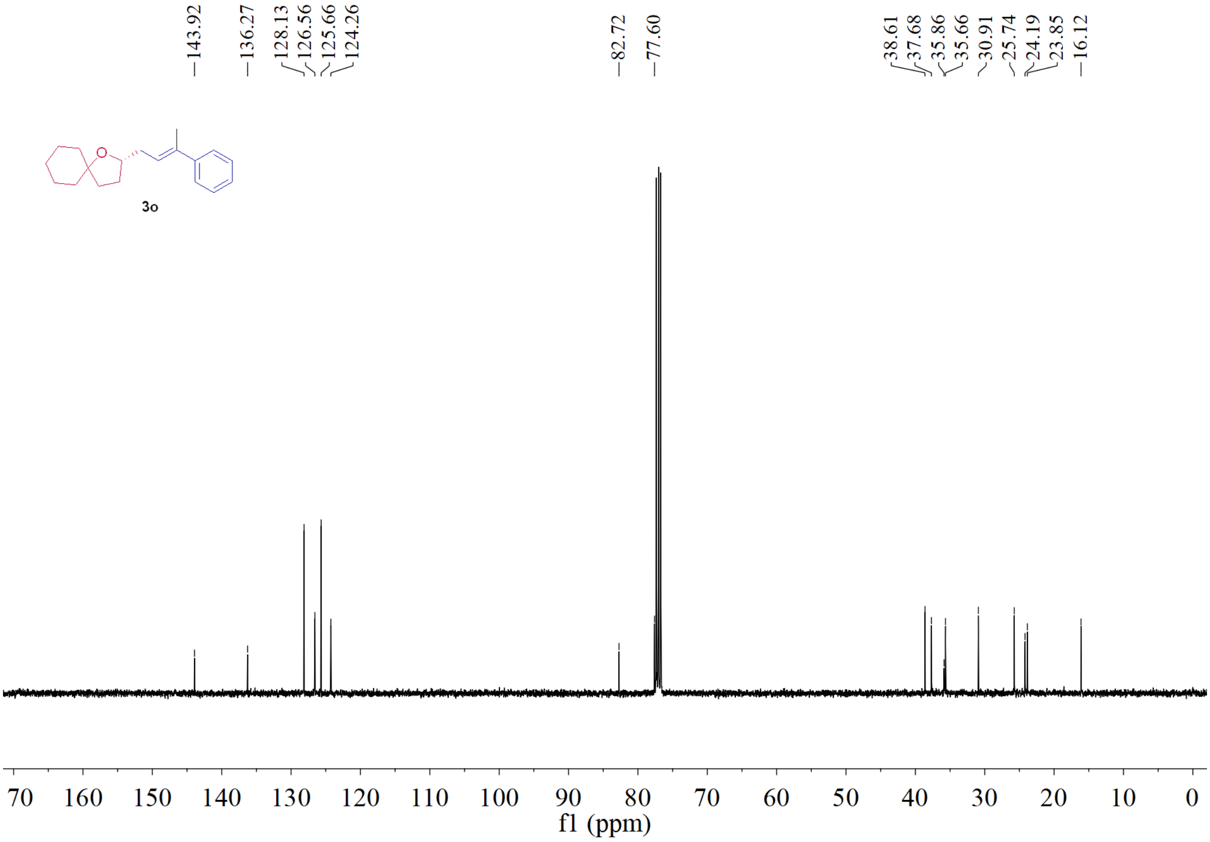
143.92 136.27 128.13 126.56 125.66 124.26 82.72 77.60 38.61 37.68 35.86 35.66 30.91 25.74 24.19 23.85 16.12
3o
f1 (ppm)

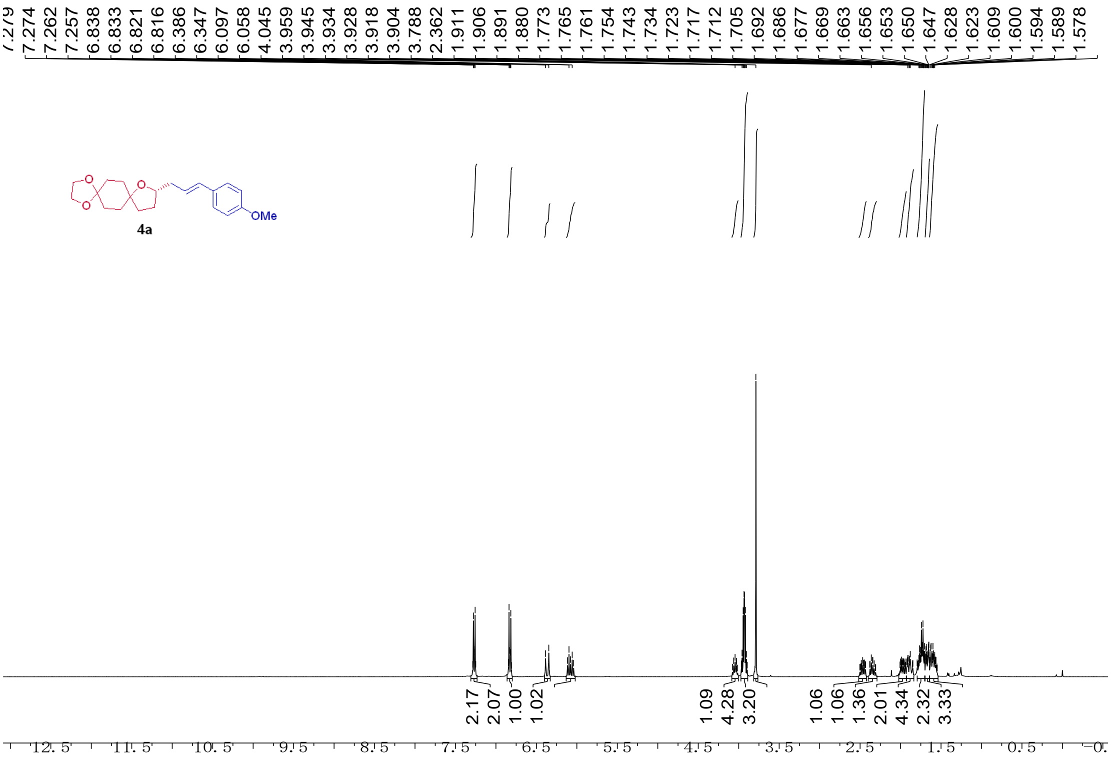
7.274
7.262
7.257
6.838
6.833
6.821
6.816
6.386
6.347
6.097
6.058
4.045
3.959
3.945
3.934
3.928
3.918
3.904
3.788
2.362
1.911
1.906
1.891
1.880
1.773
1.765
1.761
1.754
1.743
1.734
1.723
1.717
1.712
1.705
1.692
1.686
1.677
1.669
1.663
1.656
1.653
1.650
1.647
1.628
1.623
1.609
1.600
1.594
1.589
1.578
OMe
4a
2.17
2.07
1.00
1.02
1.09
4.28
3.20
1.06
1.06
1.36
2.01
4.34
2.32
3.33

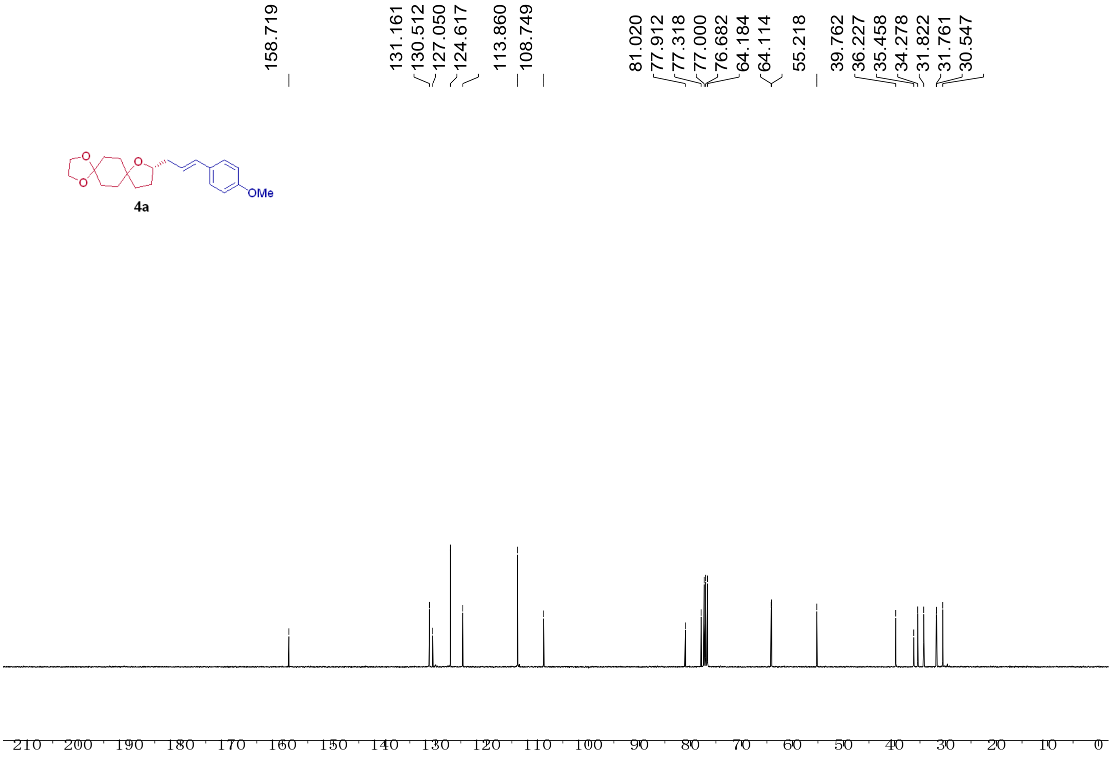
158.719
131.161
130.512
127.050
124.617
113.860
108.749
81.020
77.912
77.318
77.000
76.682
64.184
64.114
55.218
39.762
36.227
35.458
34.278
31.822
31.761
30.547
OMe
4a
210
200
190
180
170
160
150
140
130
120
110
100
90
80
70
60
50
40
30
20
10
0

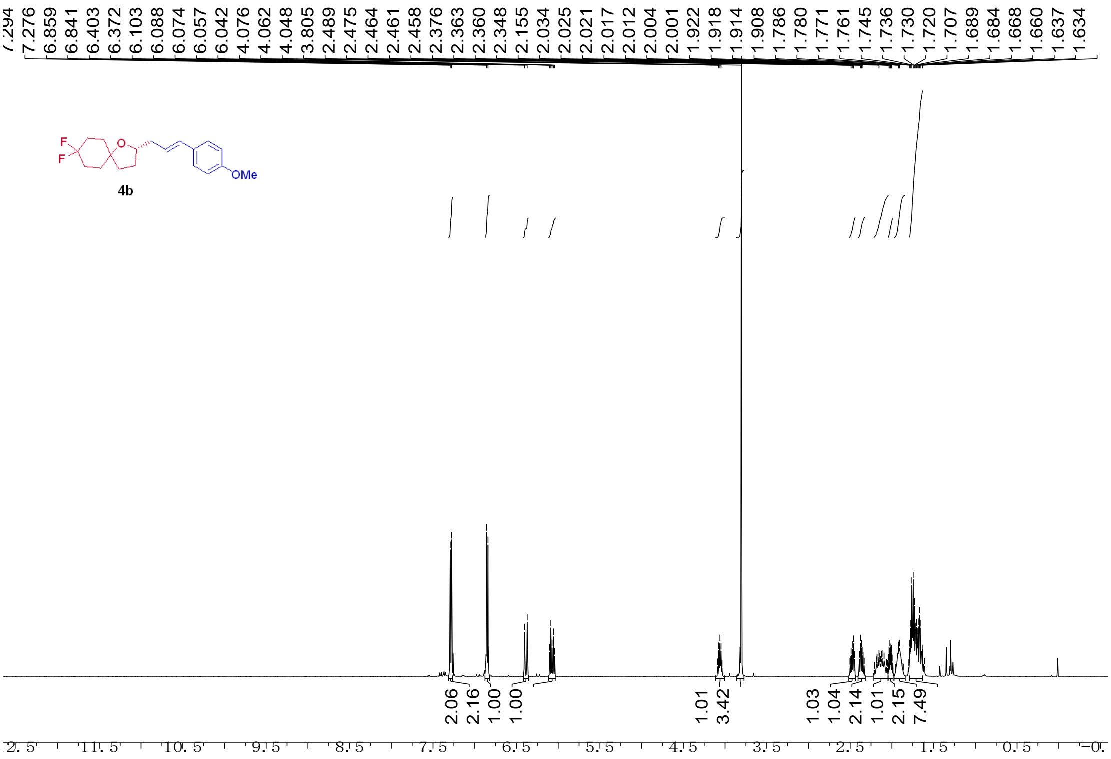
4b
OMe
F
F
O

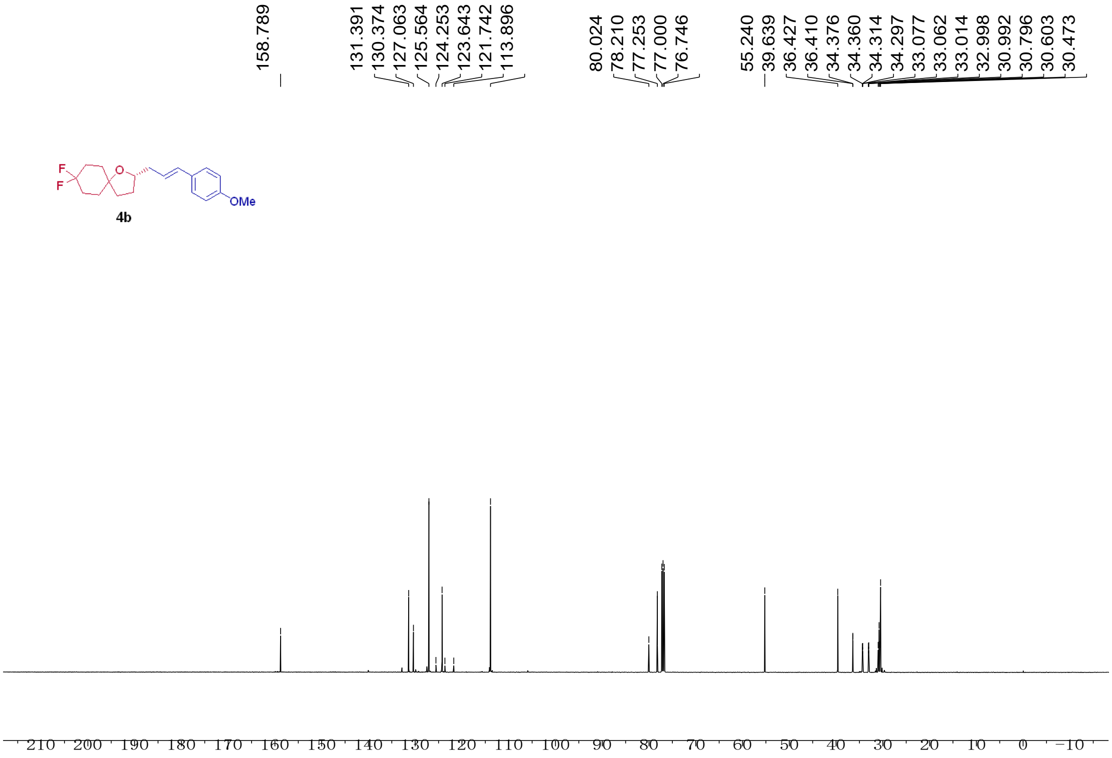
4b
OMe
F
F
O
158.789
131.391
130.374
127.063
125.564
124.253
123.643
121.742
113.896
80.024
78.210
77.253
77.000
76.746
55.240
39.639
36.427
36.410
34.376
34.360
34.314
34.297
33.077
33.062
33.014
32.998
30.992
30.796
30.603
30.473

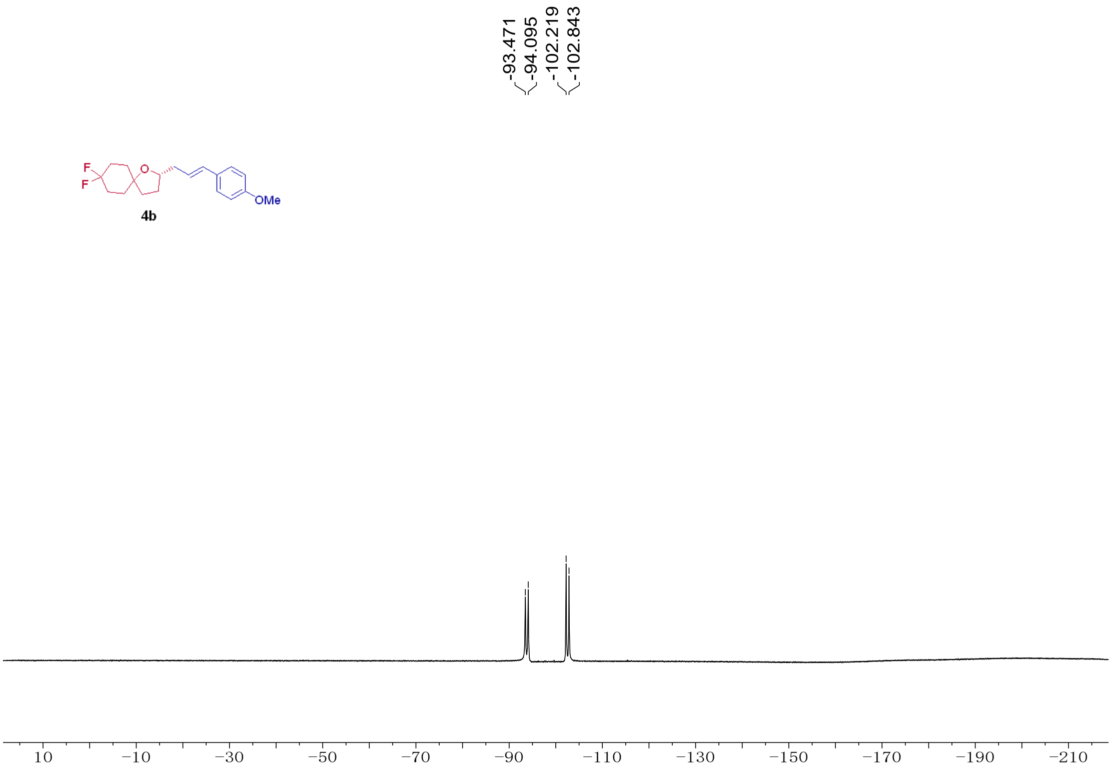
-93.471
-94.095
-102.219
-102.843
F
F
O
OMe
10 −10 −30 −50 −70 −90 −110 −130 −150 −170 −190 −210

4b

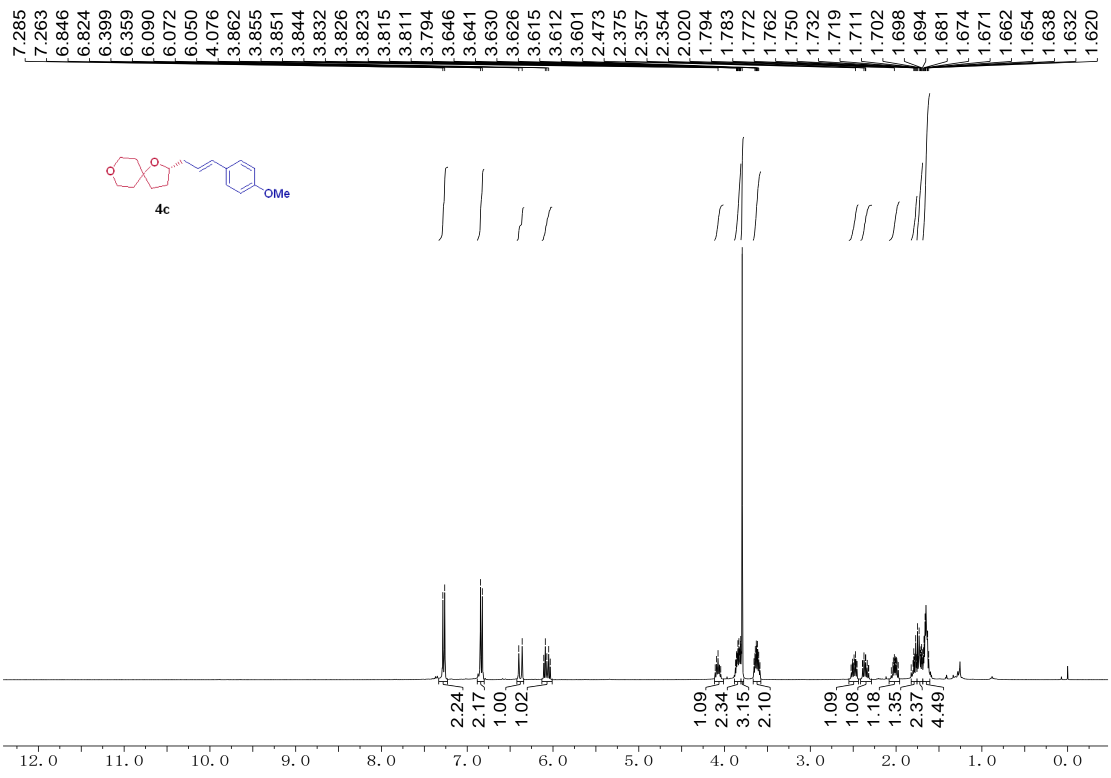
7.285
7.263
6.846
6.824
6.399
6.359
6.090
6.072
6.050
4.076
3.862
3.855
3.851
3.844
3.832
3.826
3.823
3.815
3.811
3.794
3.646
3.641
3.630
3.626
3.615
3.612
3.601
2.473
2.375
2.357
2.354
2.020
1.794
1.783
1.772
1.762
1.750
1.732
1.719
1.711
1.702
1.698
1.694
1.681
1.674
1.671
1.662
1.654
1.638
1.632
1.620
O
O
OMe
2.24
2.17
1.00
1.02
1.09
2.34
3.15
2.10
1.09
1.08
1.18
1.35
2.37
4.49
12.0 11.0 10.0 9.0 8.0 7.0 6.0 5.0 4.0 3.0 2.0 1.0 0.0

4c

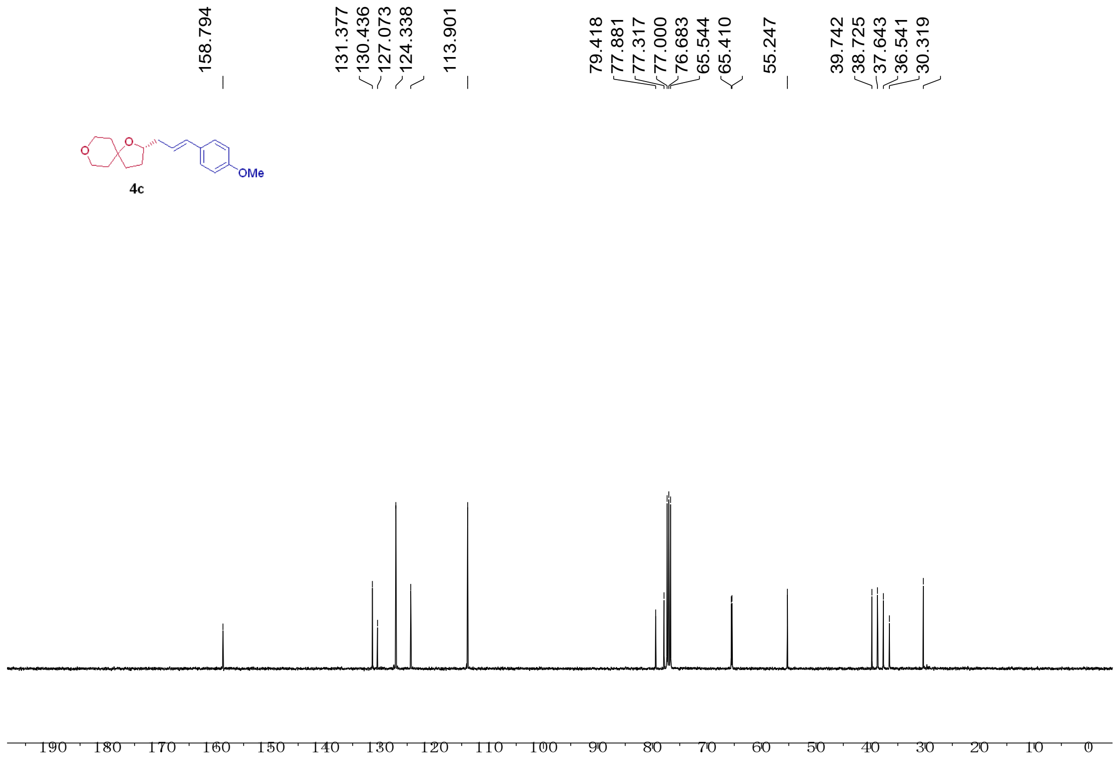
158.794
131.377
130.436
127.073
124.338
113.901
79.418
77.881
77.317
77.000
76.683
65.544
65.410
55.247
39.742
38.725
37.643
36.541
30.319
OMe
4c
190 180 170 160 150 140 130 120 110 100 90 80 70 60 50 40 30 20 10 0

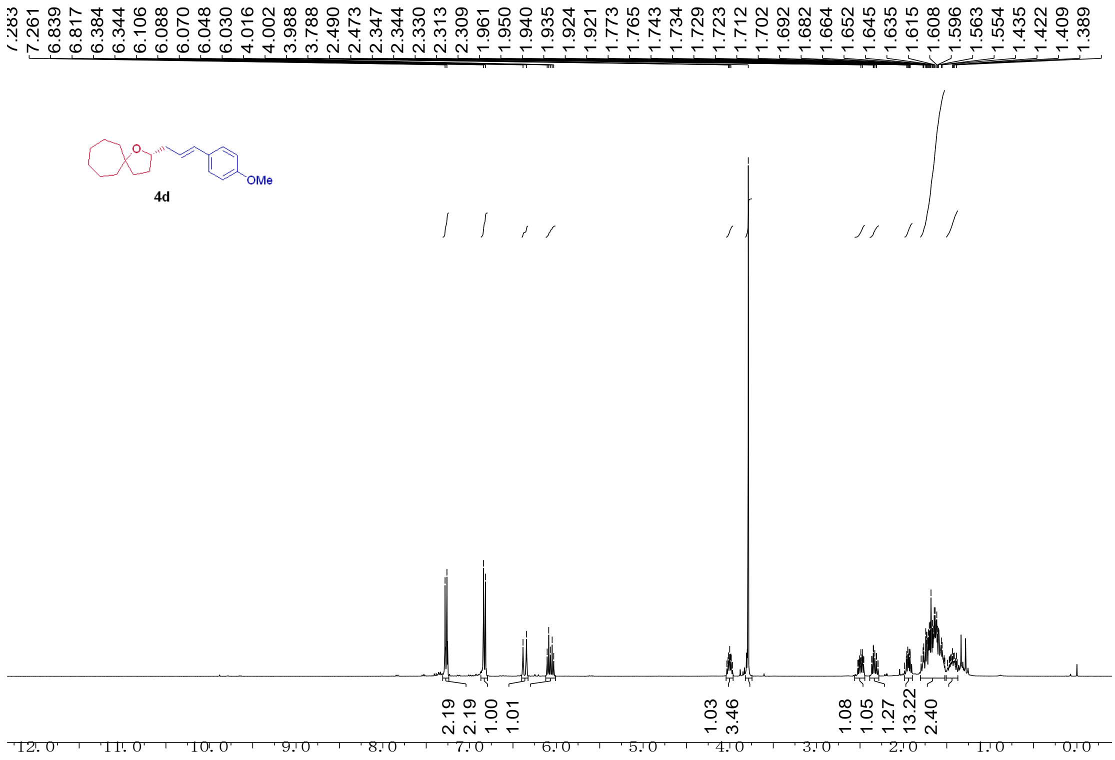
7.261
6.839
6.817
6.384
6.344
6.106
6.088
6.070
6.048
6.030
4.016
4.002
3.988
3.788
2.490
2.473
2.347
2.344
2.330
2.313
2.309
1.961
1.950
1.940
1.935
1.924
1.921
1.773
1.765
1.743
1.734
1.729
1.723
1.712
1.702
1.692
1.682
1.664
1.652
1.645
1.635
1.615
1.608
1.596
1.563
1.554
1.435
1.422
1.409
1.389
OMe
4d
2.19
2.19
1.00
1.01
1.03
3.46
1.08
1.05
1.27
13.22
2.40
12.0 11.0 10.0 9.0 8.0 7.0 6.0 5.0 4.0 3.0 2.0 1.0 0.0

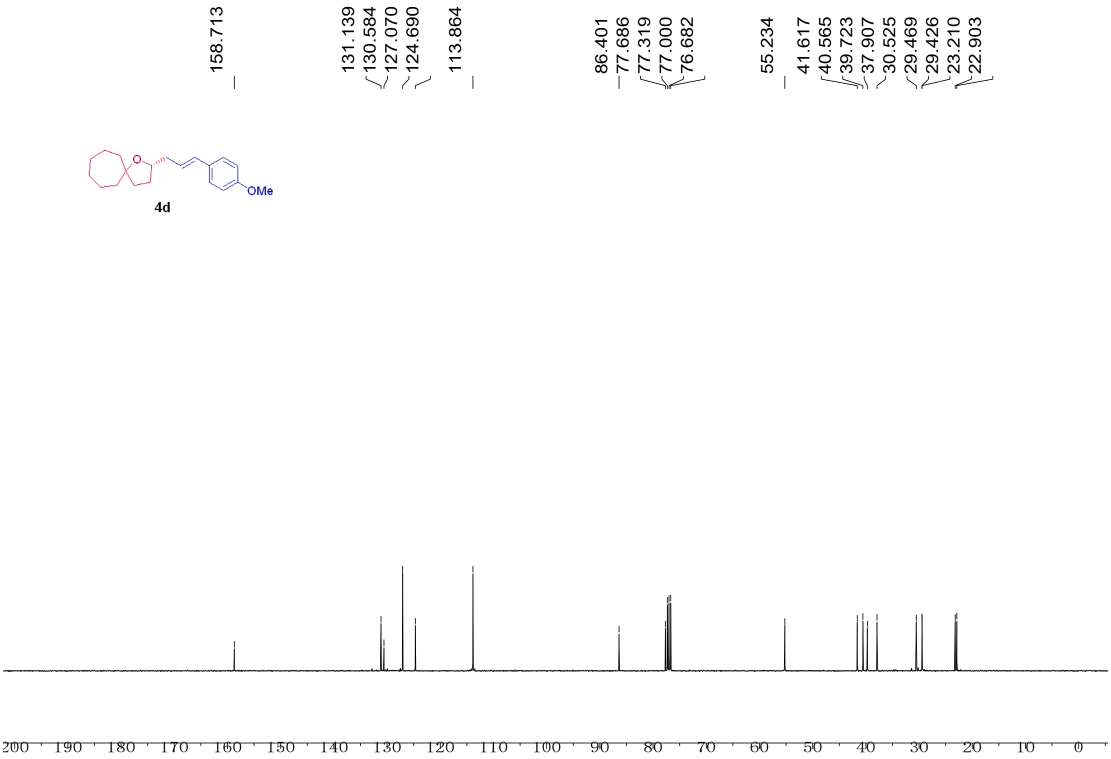
158.713
131.139
130.584
127.070
124.690
113.864
86.401
77.686
77.319
77.000
76.682
55.234
41.617
40.565
39.723
37.907
30.525
29.469
29.426
23.210
22.903
OMe
4d
200
190
180
170
160
150
140
130
120
110
100
90
80
70
60
50
40
30
20
10
0

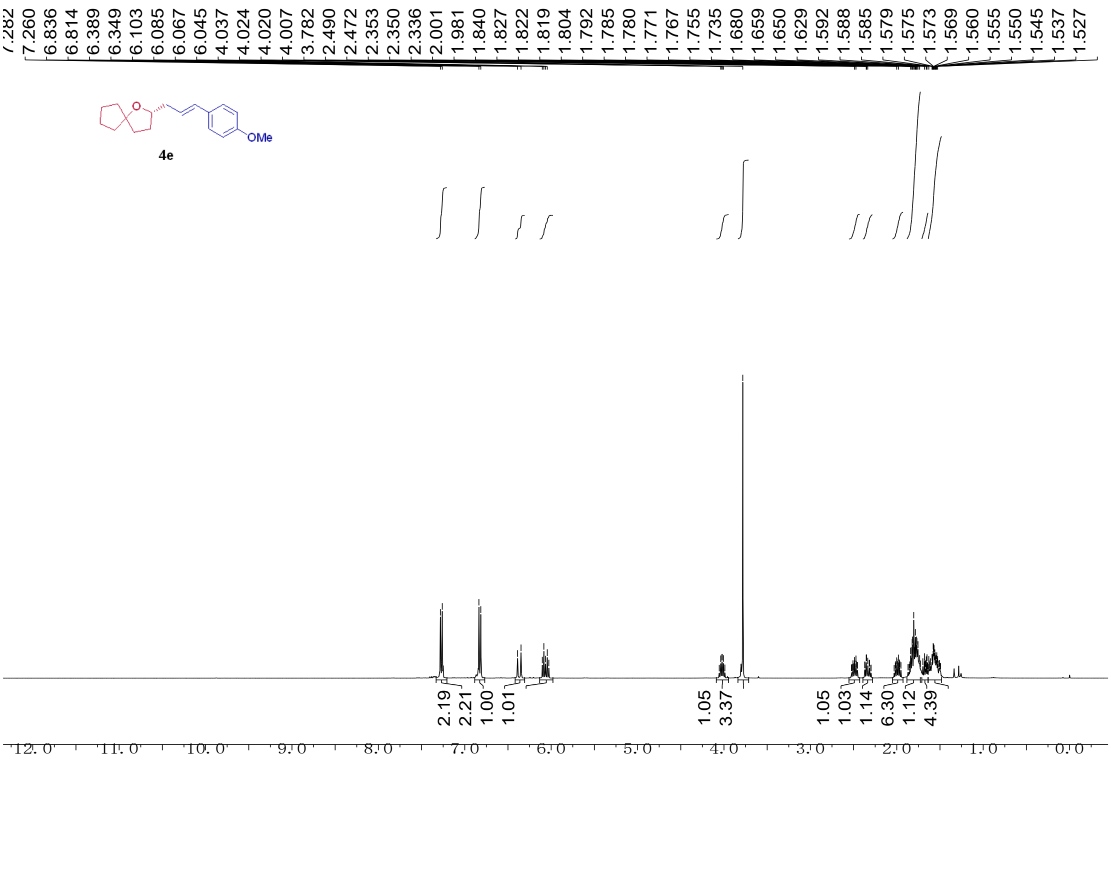
7.282
7.260
6.836
6.814
6.389
6.349
6.103
6.085
6.067
6.045
4.037
4.024
4.020
4.007
3.782
2.490
2.472
2.353
2.350
2.336
2.001
1.981
1.840
1.827
1.822
1.819
1.804
1.792
1.785
1.780
1.771
1.767
1.755
1.735
1.680
1.659
1.650
1.629
1.592
1.588
1.585
1.579
1.575
1.573
1.569
1.560
1.555
1.550
1.545
1.537
1.527
OMe
4e
2.19
2.21
1.00
1.01
1.05
3.37
1.05
1.03
1.14
6.30
1.12
4.39
12.0
11.0
10.0
9.0
8.0
7.0
6.0
5.0
4.0
3.0
2.0
1.0
0.0

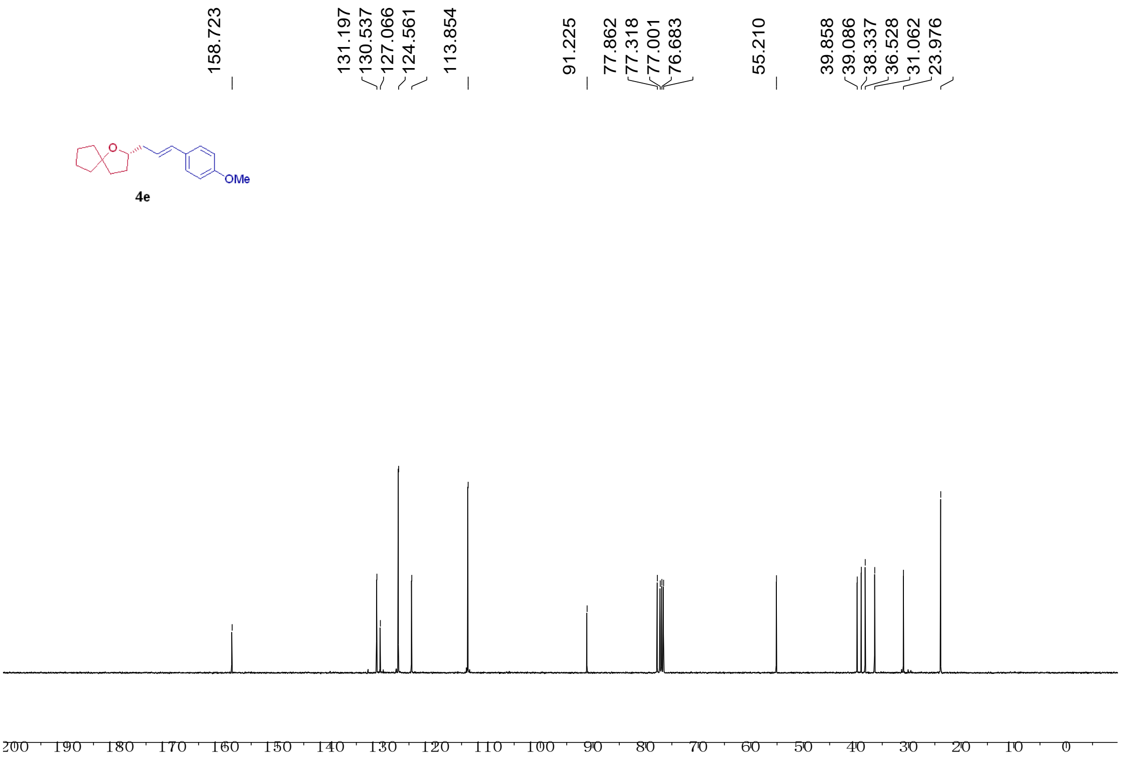
158.723
131.197
130.537
127.066
124.561
113.854
91.225
77.862
77.318
77.001
76.683
55.210
39.858
39.086
38.337
36.528
31.062
23.976
OMe
4e
200 190 180 170 160 150 140 130 120 110 100 90 80 70 60 50 40 30 20 10 0

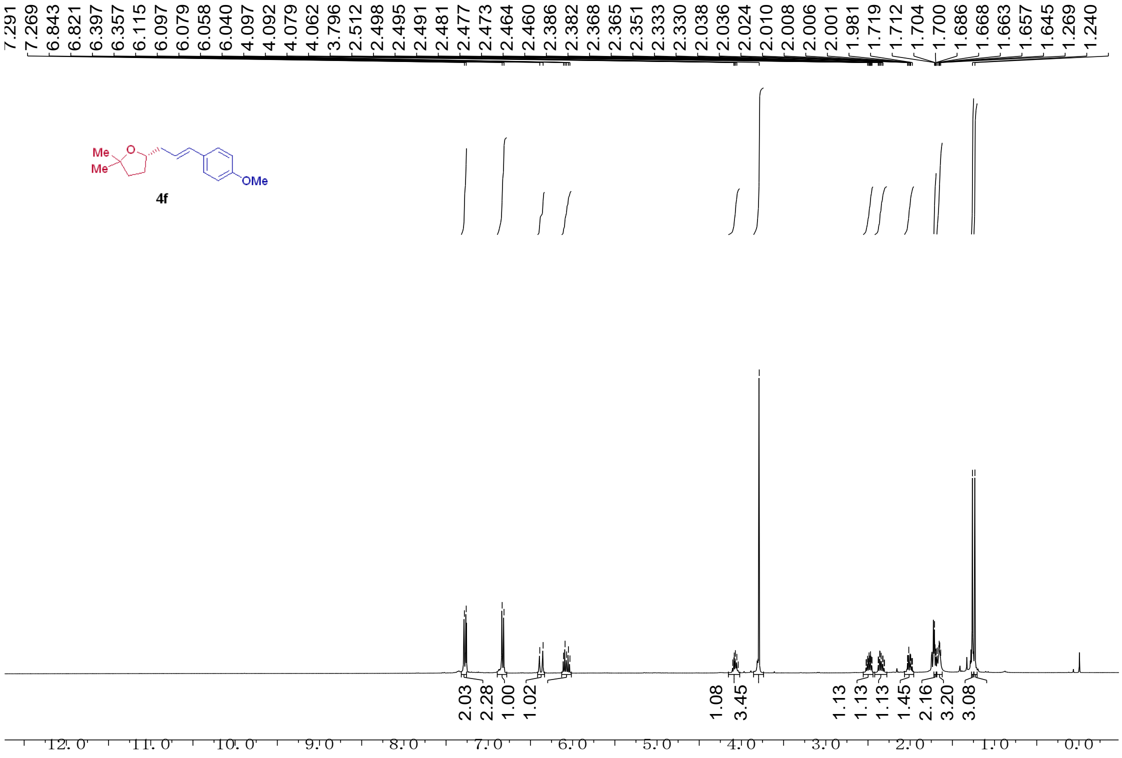
7.291
7.269
6.843
6.821
6.397
6.357
6.115
6.097
6.079
6.058
6.040
4.097
4.092
4.079
4.062
3.796
2.512
2.498
2.495
2.491
2.481
2.477
2.473
2.464
2.460
2.386
2.382
2.368
2.365
2.351
2.333
2.330
2.038
2.036
2.024
2.010
2.008
2.006
2.001
1.981
1.719
1.712
1.704
1.700
1.686
1.668
1.663
1.657
1.645
1.269
1.240
Me
Me
OMe
4f
2.03
2.28
1.00
1.02
1.08
3.45
1.13
1.13
1.13
1.45
2.16
3.20
3.08
12.0 11.0 10.0 9.0 8.0 7.0 6.0 5.0 4.0 3.0 2.0 1.0 0.0

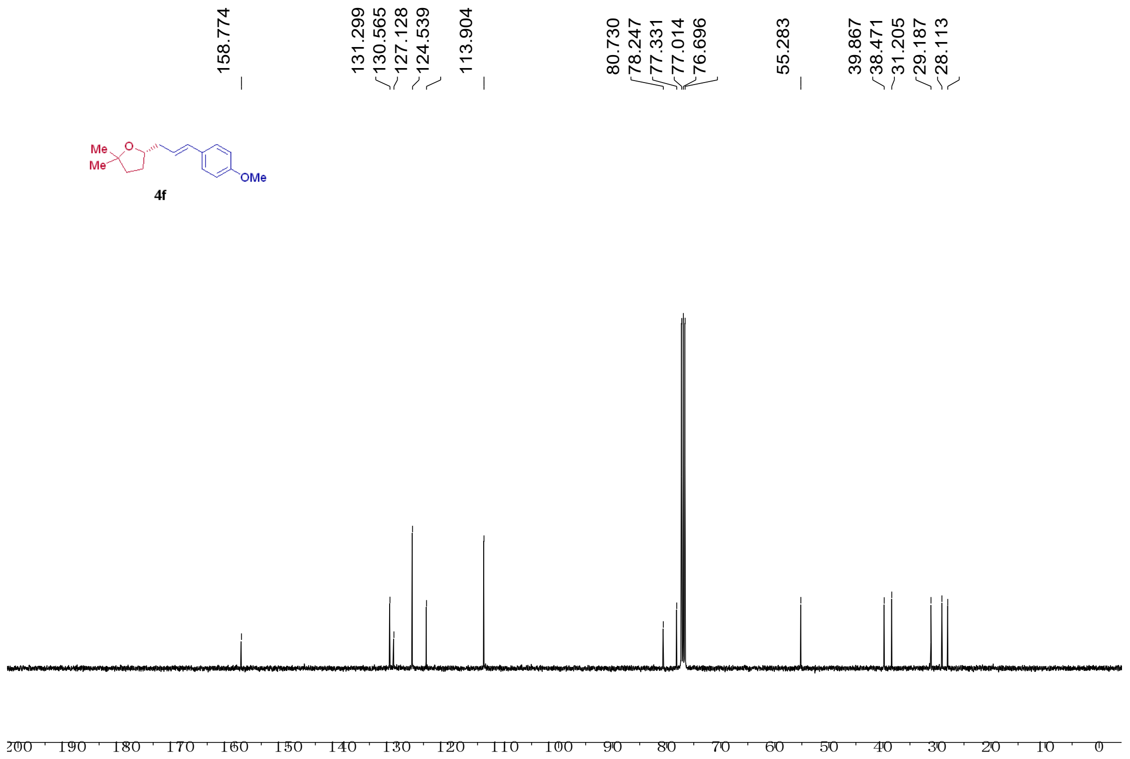
158.774
131.299
130.565
127.128
124.539
113.904
80.730
78.247
77.331
77.014
76.696
55.283
39.867
38.471
31.205
29.187
28.113
Me
Me
O
OMe
4f
200 190 180 170 160 150 140 130 120 110 100 90 80 70 60 50 40 30 20 10 0

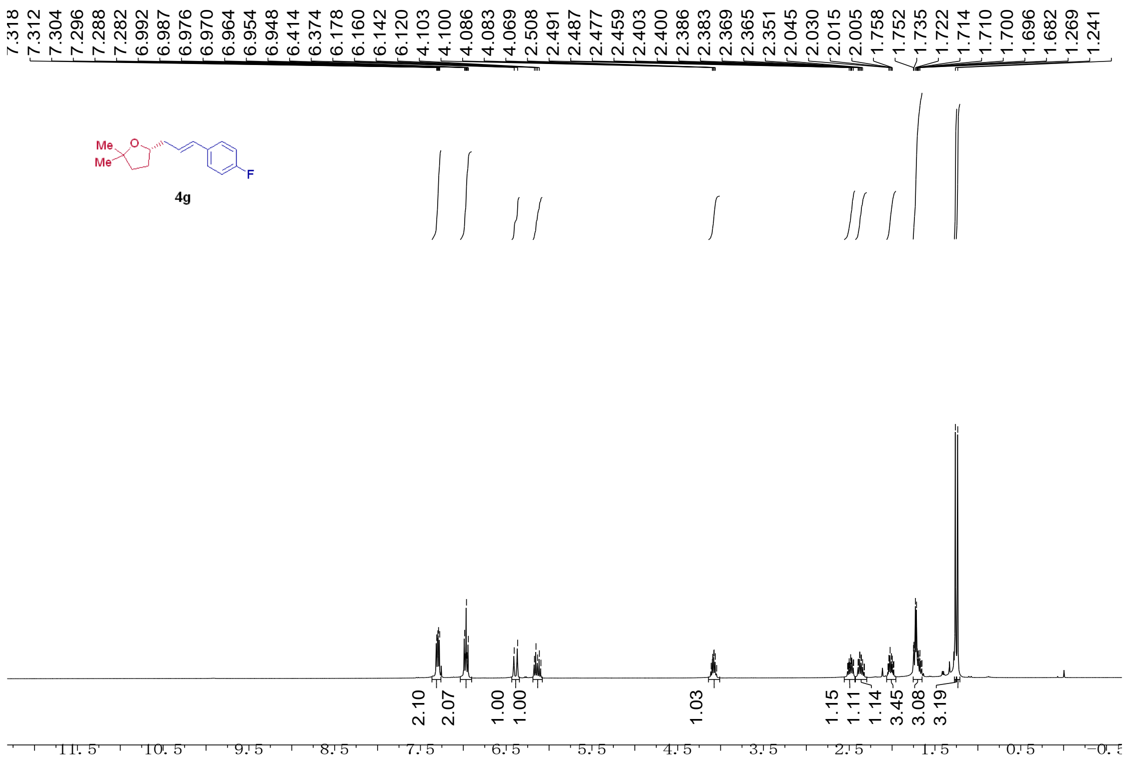
7.318
7.312
7.304
7.296
7.288
7.282
6.992
6.987
6.976
6.970
6.964
6.954
6.948
6.414
6.374
6.178
6.160
6.142
6.120
4.103
4.100
4.086
4.083
4.069
2.508
2.491
2.487
2.477
2.459
2.403
2.400
2.386
2.383
2.369
2.365
2.351
2.045
2.030
2.015
2.005
1.758
1.752
1.735
1.722
1.714
1.710
1.700
1.696
1.682
1.269
1.241
Me
Me
O
F
4g
2.10
2.07
1.00
1.00
1.03
1.15
1.11
1.14
3.45
3.08
3.19

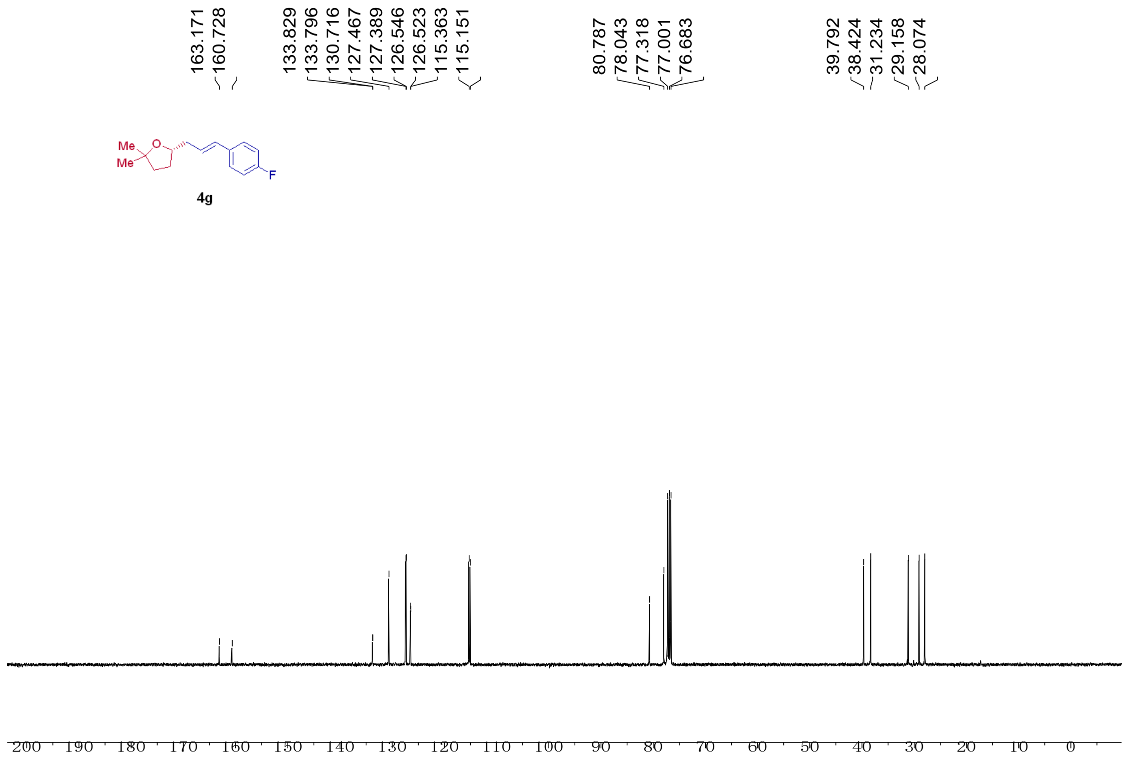
163.171
160.728
133.829
133.796
130.716
127.467
127.389
126.546
126.523
115.363
115.151
80.787
78.043
77.318
77.001
76.683
39.792
38.424
31.234
29.158
28.074
Me
Me
O
F
4g
200 190 180 170 160 150 140 130 120 110 100 90 80 70 60 50 40 30 20 10 0

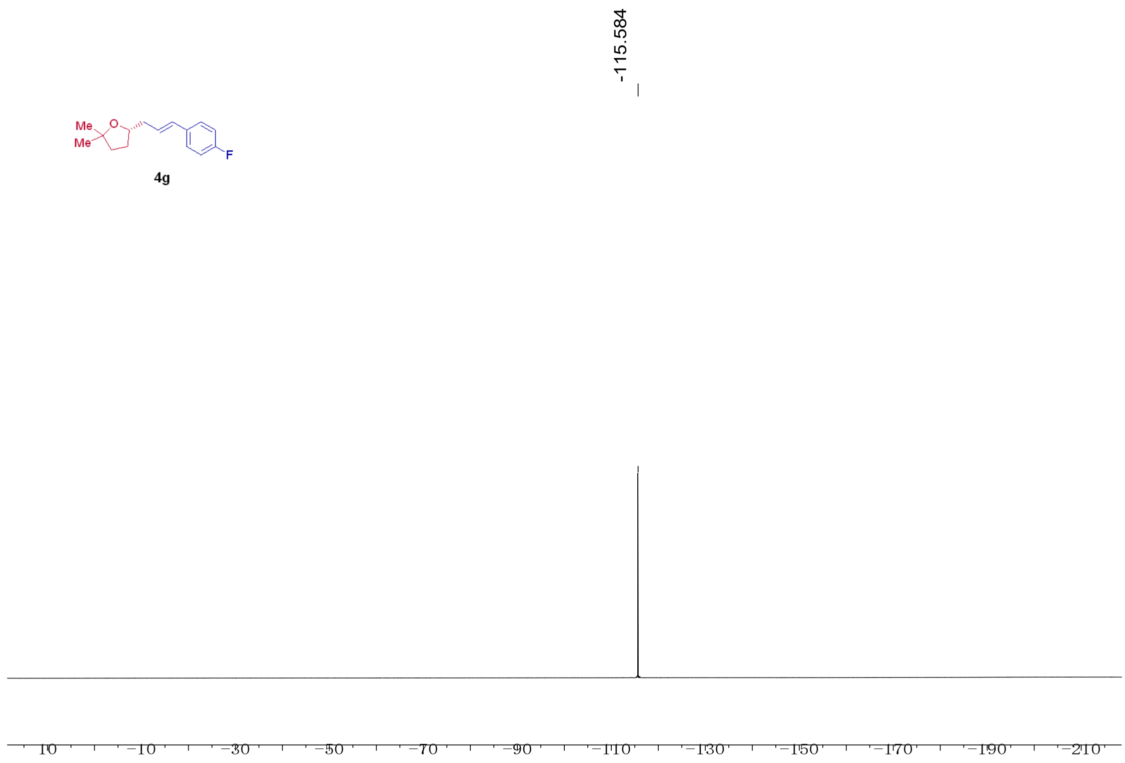
-115.584
Me
Me
O
F
4g
10 −10 −30 −50 −70 −90 −110 −130 −150 −170 −190 −210

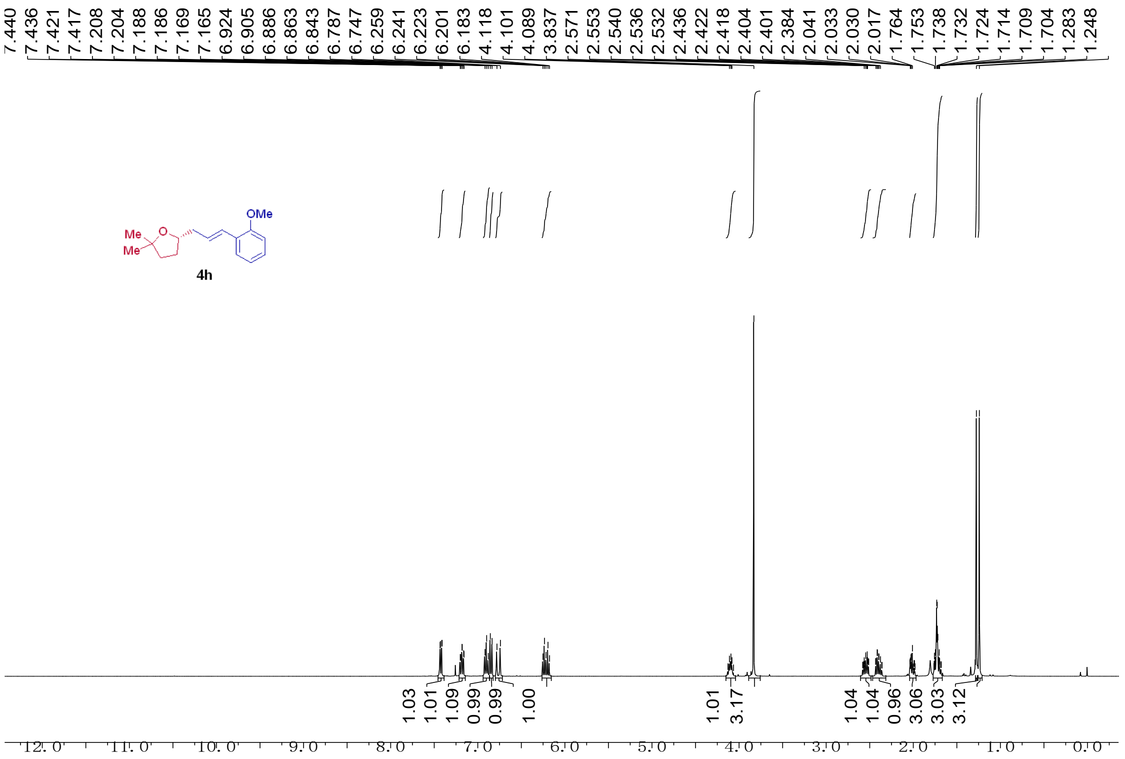
7.440
7.436
7.421
7.417
7.208
7.204
7.188
7.186
7.169
7.165
6.924
6.905
6.886
6.863
6.843
6.787
6.747
6.259
6.241
6.223
6.201
6.183
4.118
4.101
4.089
3.837
2.571
2.553
2.540
2.536
2.532
2.436
2.422
2.418
2.404
2.401
2.384
2.041
2.033
2.030
2.017
1.764
1.753
1.738
1.732
1.724
1.714
1.709
1.704
1.283
1.248
OMe
Me
O
Me
4h
1.03
1.01
1.09
0.99
0.99
1.00
1.01
3.17
1.04
1.04
0.96
3.06
3.03
3.12
12.0 11.0 10.0 9.0 8.0 7.0 6.0 5.0 4.0 3.0 2.0 1.0 0.0

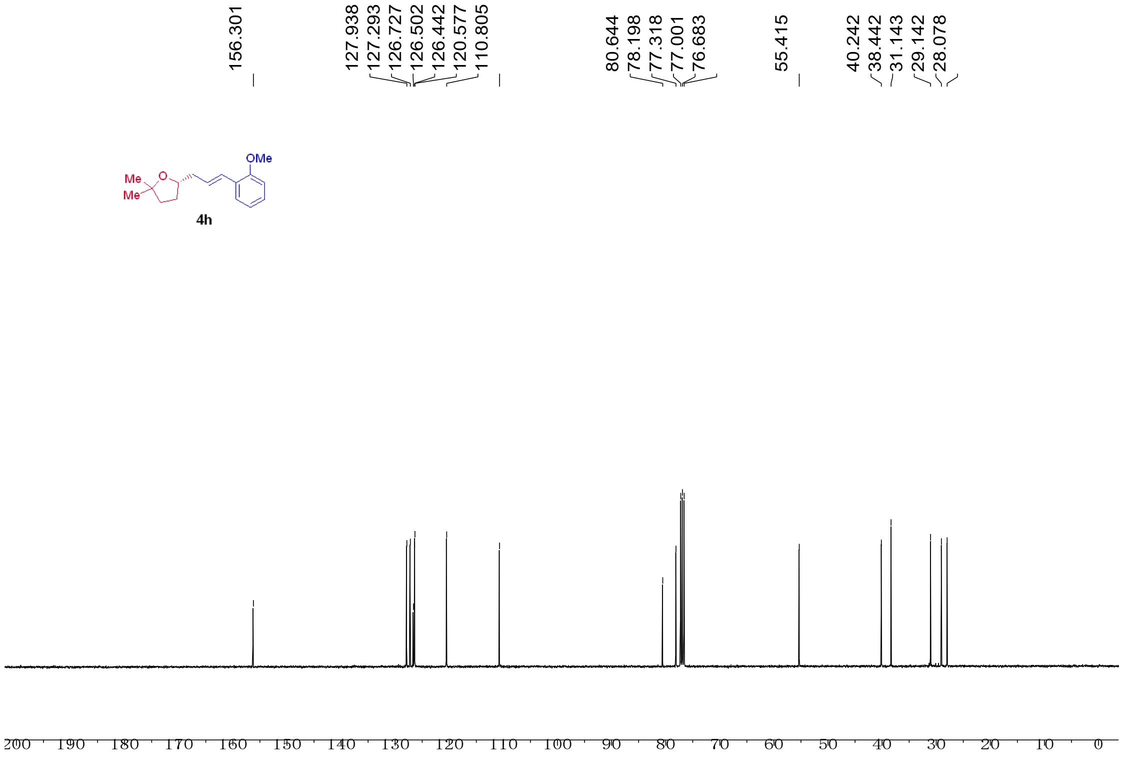
156.301
127.938
127.293
126.727
126.502
126.442
120.577
110.805
80.644
78.198
77.318
77.001
76.683
55.415
40.242
38.442
31.143
29.142
28.078
OMe
Me
O
Me
4h
200 190 180 170 160 150 140 130 120 110 100 90 80 70 60 50 40 30 20 10 0

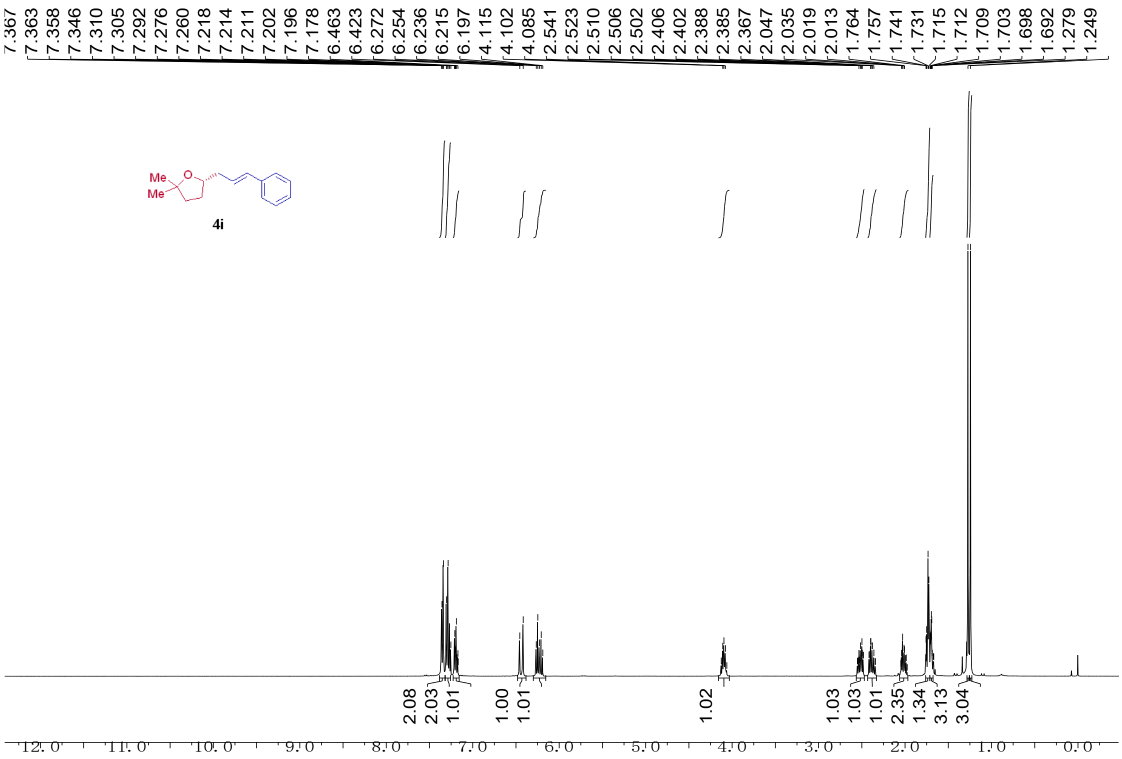
7.367
7.363
7.358
7.346
7.310
7.305
7.292
7.276
7.260
7.218
7.214
7.211
7.202
7.196
7.178
6.463
6.423
6.272
6.254
6.236
6.215
6.197
4.115
4.102
4.085
2.541
2.523
2.510
2.506
2.502
2.406
2.402
2.388
2.385
2.367
2.047
2.035
2.019
2.013
1.764
1.757
1.741
1.731
1.715
1.712
1.709
1.703
1.698
1.692
1.279
1.249
Me
Me
O
4i
2.08
2.03
1.01
1.00
1.01
1.02
1.03
1.03
1.01
2.35
1.34
3.13
3.04
12.0 11.0 10.0 9.0 8.0 7.0 6.0 5.0 4.0 3.0 2.0 1.0 0.0

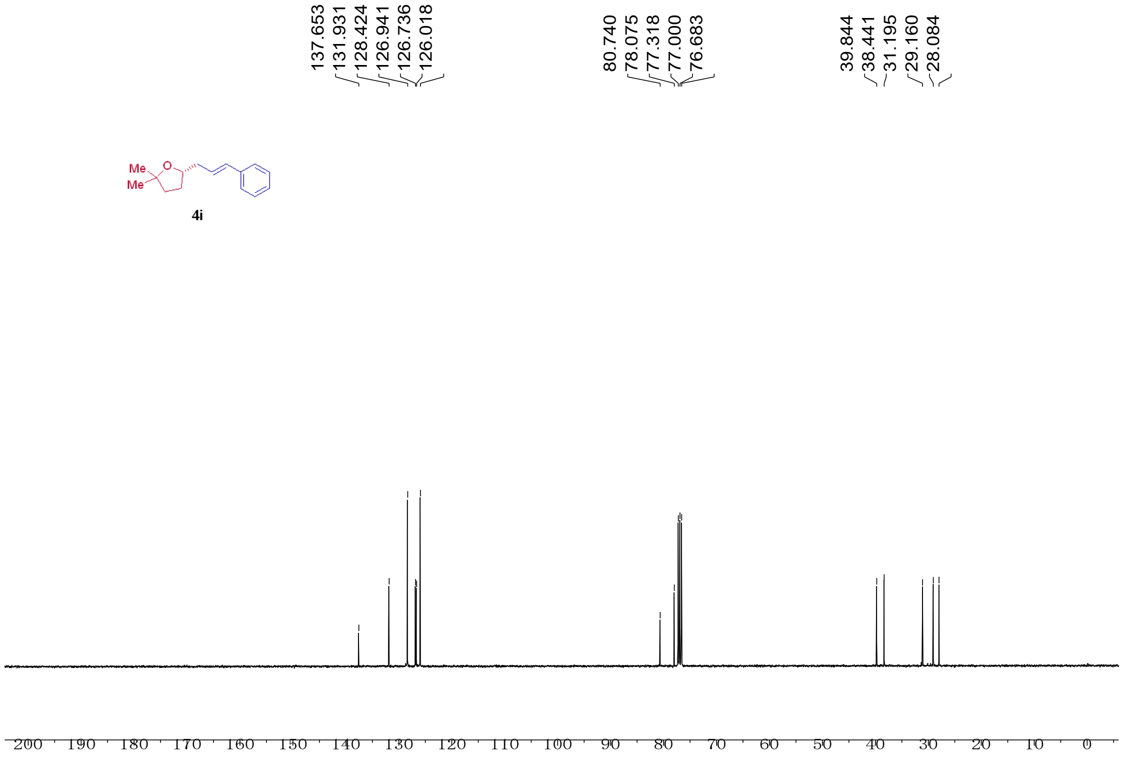
137.653
131.931
128.424
126.941
126.736
126.018
80.740
78.075
77.318
77.000
76.683
39.844
38.441
31.195
29.160
28.084
Me
Me
O
4i
200 190 180 170 160 150 140 130 120 110 100 90 80 70 60 50 40 30 20 10 0

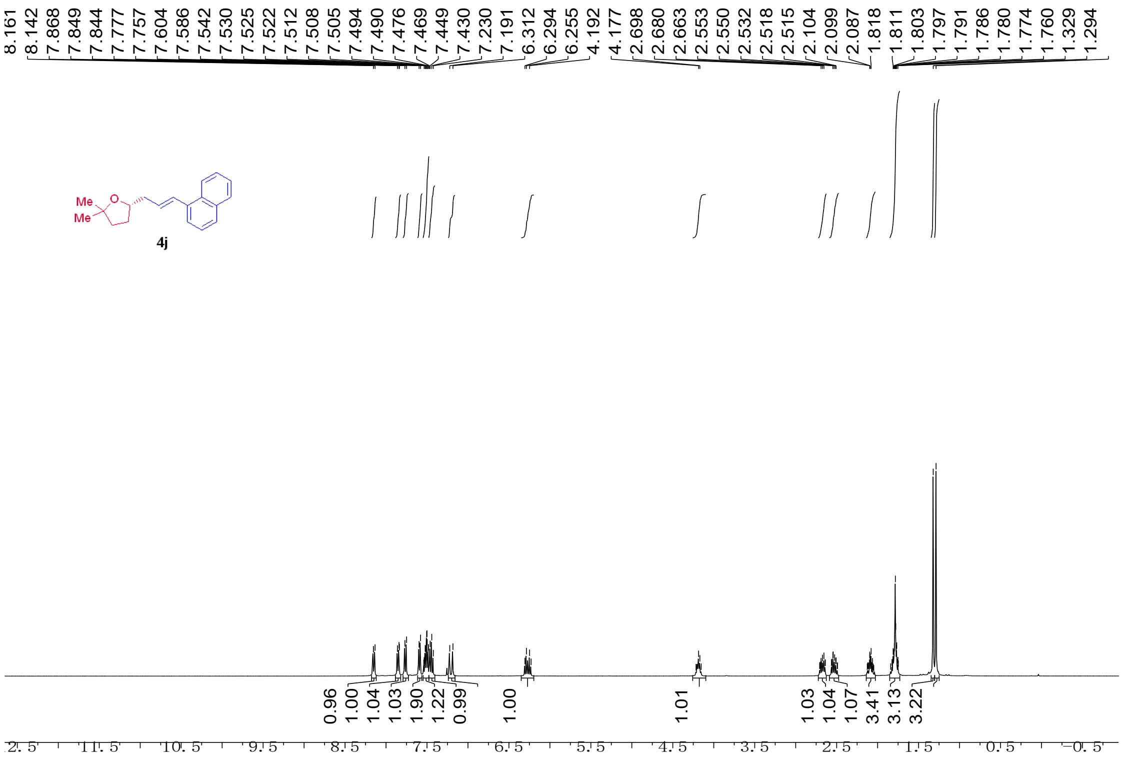
8.161
8.142
7.868
7.849
7.844
7.777
7.757
7.604
7.586
7.542
7.530
7.525
7.522
7.512
7.508
7.505
7.494
7.490
7.476
7.469
7.449
7.430
7.230
7.191
6.312
6.294
6.255
4.192
4.177
2.698
2.680
2.663
2.553
2.550
2.532
2.518
2.515
2.104
2.099
2.087
1.818
1.811
1.803
1.797
1.791
1.786
1.780
1.774
1.760
1.329
1.294
Me
Me
O
4j
0.96
1.00
1.04
1.03
1.90
1.22
0.99
1.00
1.01
1.03
1.04
1.07
3.41
3.13
3.22
12.5 11.5 10.5 9.5 8.5 7.5 6.5 5.5 4.5 3.5 2.5 1.5 0.5 -0.5

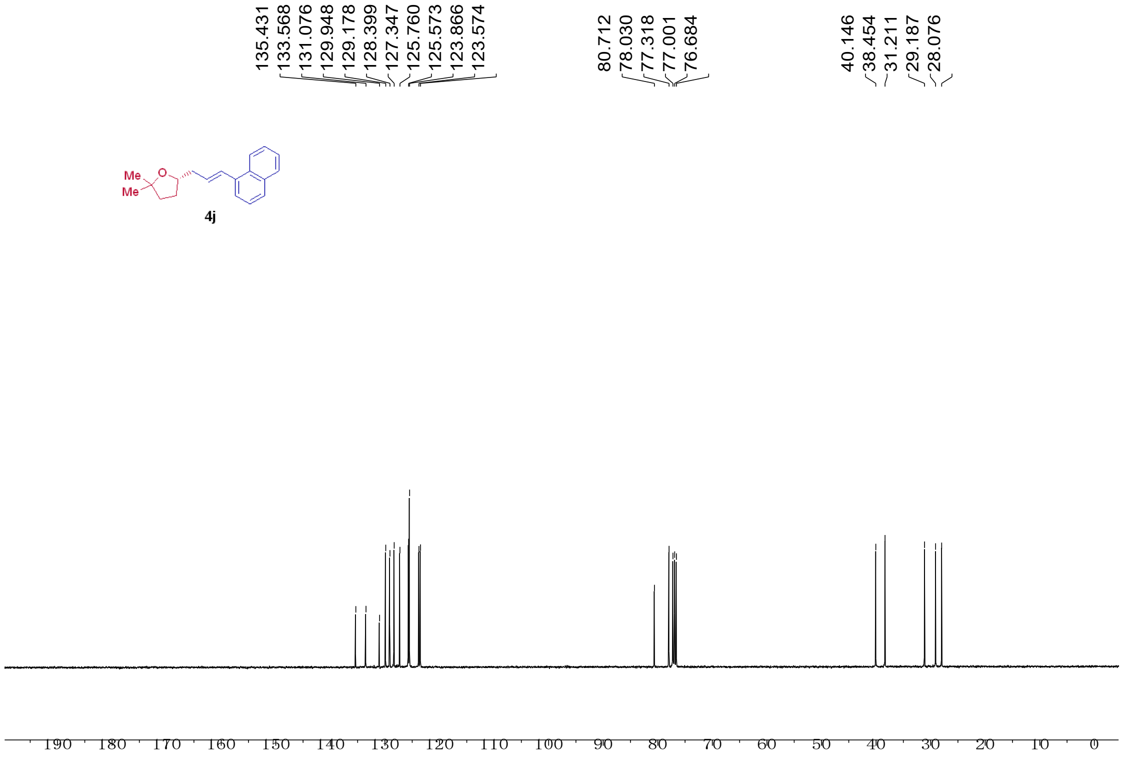
135.431
133.568
131.076
129.948
129.178
128.399
127.347
125.760
125.573
123.866
123.574
80.712
78.030
77.318
77.001
76.684
40.146
38.454
31.211
29.187
28.076
Me
Me
O
4j
190 180 170 160 150 140 130 120 110 100 90 80 70 60 50 40 30 20 10 0

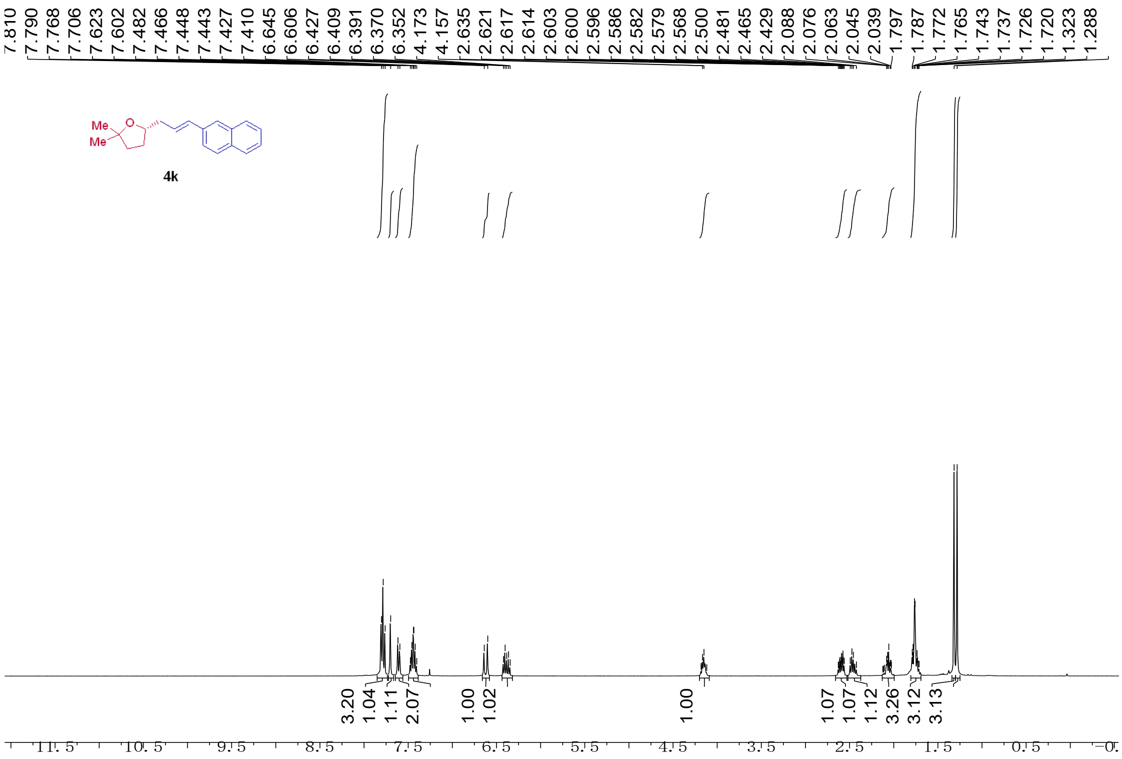
7.810 7.790 7.768 7.706 7.623 7.602 7.482 7.466 7.448 7.443 7.427 7.410 6.645 6.606 6.427 6.409 6.391 6.370 6.352 4.173 4.157 2.635 2.621 2.617 2.614 2.603 2.600 2.596 2.586 2.582 2.579 2.568 2.500 2.481 2.465 2.429 2.088 2.076 2.063 2.045 2.039 1.797 1.787 1.772 1.765 1.743 1.737 1.726 1.720 1.323 1.288
Me
Me
O
4k
3.20 1.04 1.11 2.07 1.00 1.02 1.00 1.07 1.07 1.12 3.26 3.12 3.13
11.5 10.5 9.5 8.5 7.5 6.5 5.5 4.5 3.5 2.5 1.5 0.5

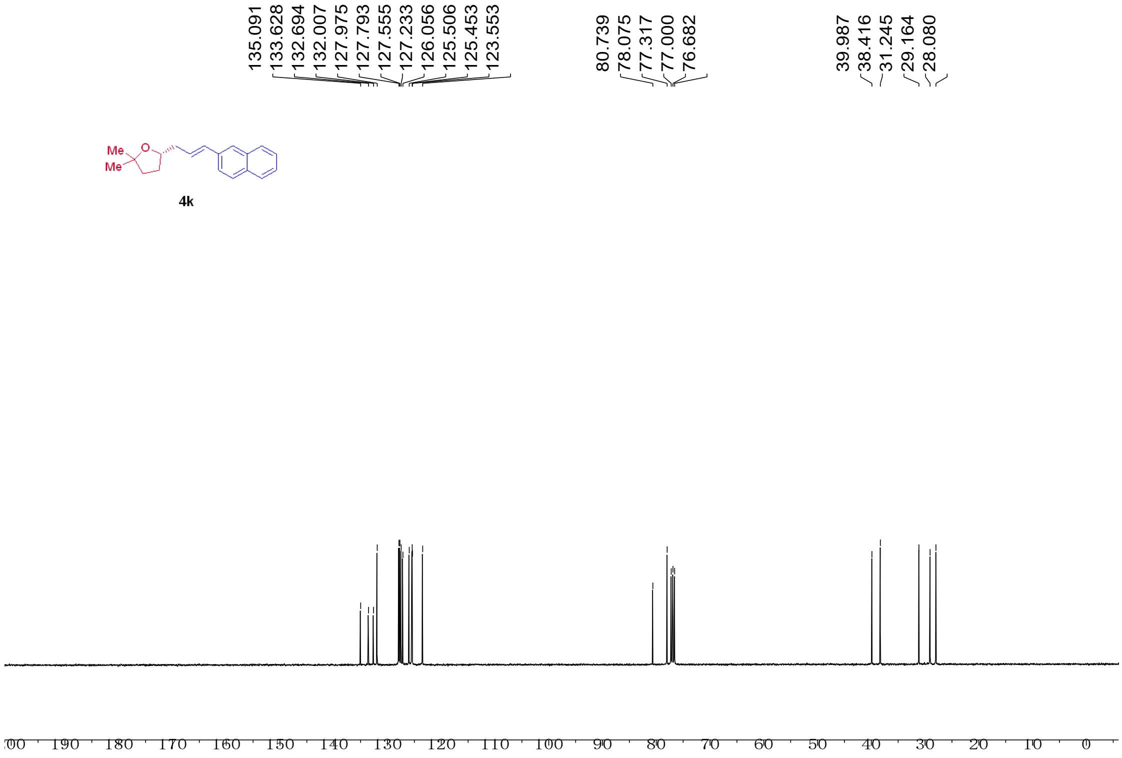
135.091 133.628 132.694 132.007 127.975 127.793 127.555 127.233 126.056 125.506 125.453 123.553
80.739 78.075 77.317 77.000 76.682
39.987 38.416 31.245 29.164 28.080
Me
Me
O
4k
190 180 170 160 150 140 130 120 110 100 90 80 70 60 50 40 30 20 10 0

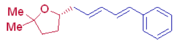
Me
Me
O
4l

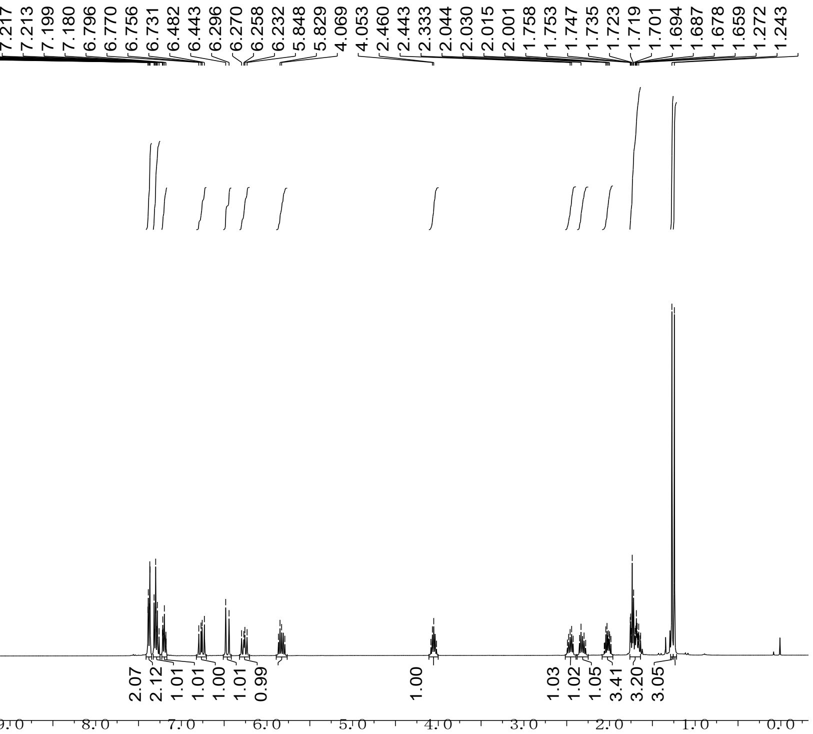
7.390 7.386 7.372 7.368 7.366 7.318 7.313 7.300 7.296 7.284 7.280 7.260 7.220 7.217 7.213 7.199 7.180 6.796 6.770 6.756 6.731 6.482 6.443 6.296 6.270 6.258 6.232 5.848 5.829 4.069 4.053 2.460 2.443 2.333 2.044 2.030 2.015 2.001 1.758 1.753 1.747 1.735 1.723 1.719 1.701 1.694 1.687 1.678 1.659 1.272 1.243
2.07 2.12 1.01 1.01 1.00 1.01 0.99 1.00 1.03 1.02 1.05 3.41 3.20 3.05
12.0 11.0 10.0 9.0 8.0 7.0 6.0 5.0 4.0 3.0 2.0 1.0 0.0

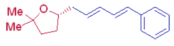
Me
Me
O
4l

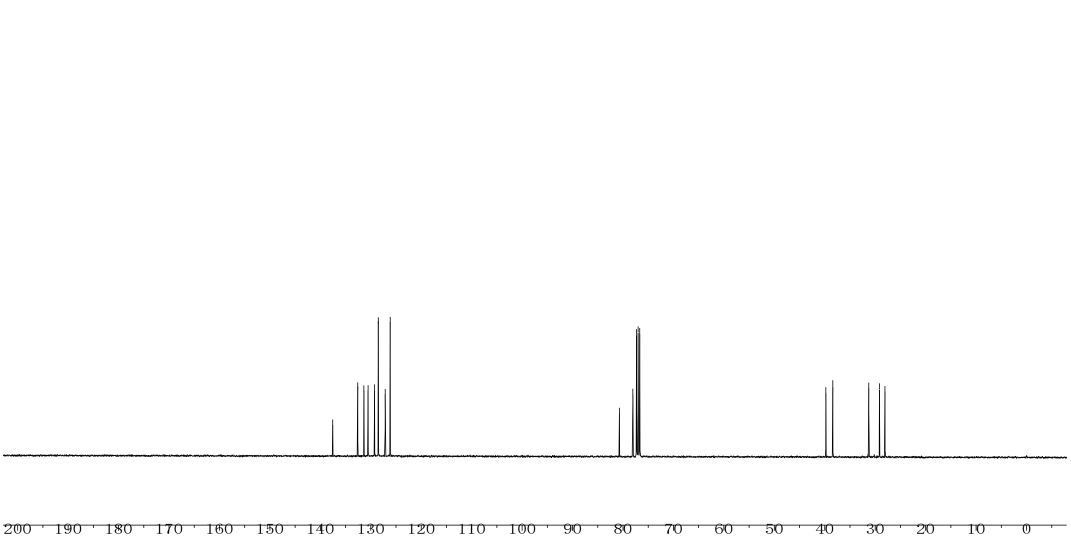
137.543 132.583 131.371 130.534 129.251 128.512 127.142 126.146
80.715 78.045 77.318 77.000 76.682
39.787 38.404 31.276 29.161 28.066
200 190 180 170 160 150 140 130 120 110 100 90 80 70 60 50 40 30 20 10 0

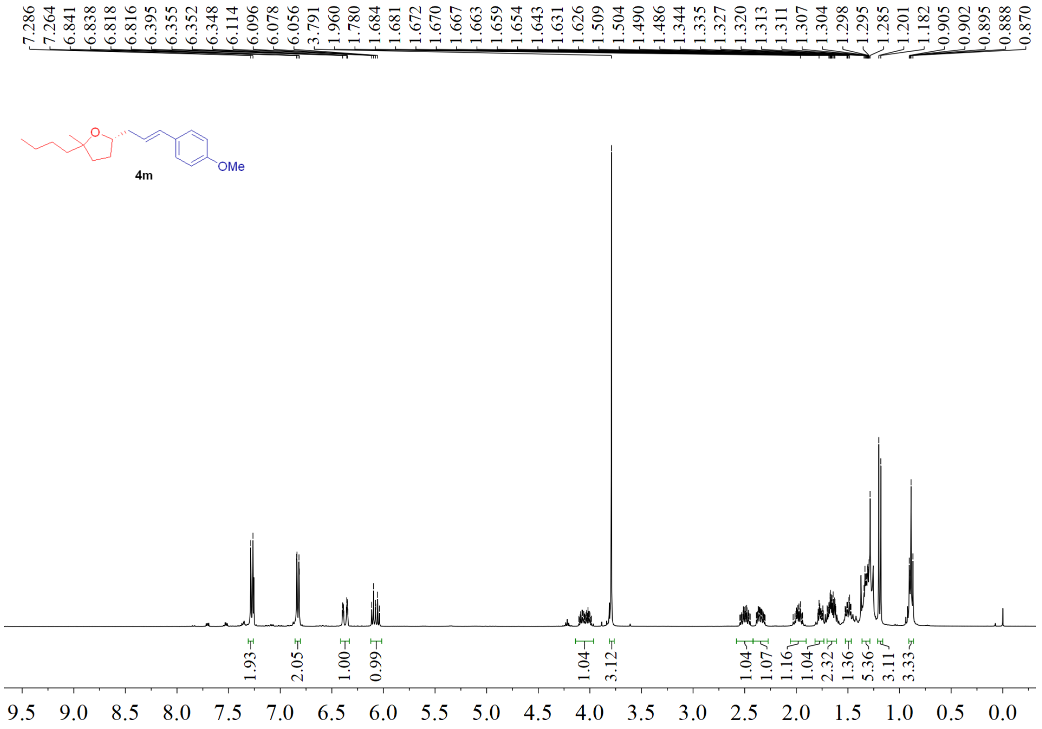
7.286 7.264 6.841 6.838 6.818 6.816 6.395 6.355 6.352 6.348 6.114 6.096 6.078 6.056 3.791 1.960 1.780 1.684 1.681 1.672 1.670 1.667 1.663 1.659 1.654 1.643 1.631 1.626 1.509 1.504 1.490 1.486 1.344 1.335 1.327 1.320 1.313 1.311 1.307 1.304 1.298 1.295 1.285 1.201 1.182 0.905 0.902 0.895 0.888 0.870
OMe
4m
1.93 2.05 1.00 0.99 1.04 3.12 1.04 1.07 1.16 1.04 2.32 1.36 5.36 3.11 3.33
9.5 9.0 8.5 8.0 7.5 7.0 6.5 6.0 5.5 5.0 4.5 4.0 3.5 3.0 2.5 2.0 1.5 1.0 0.5 0.0

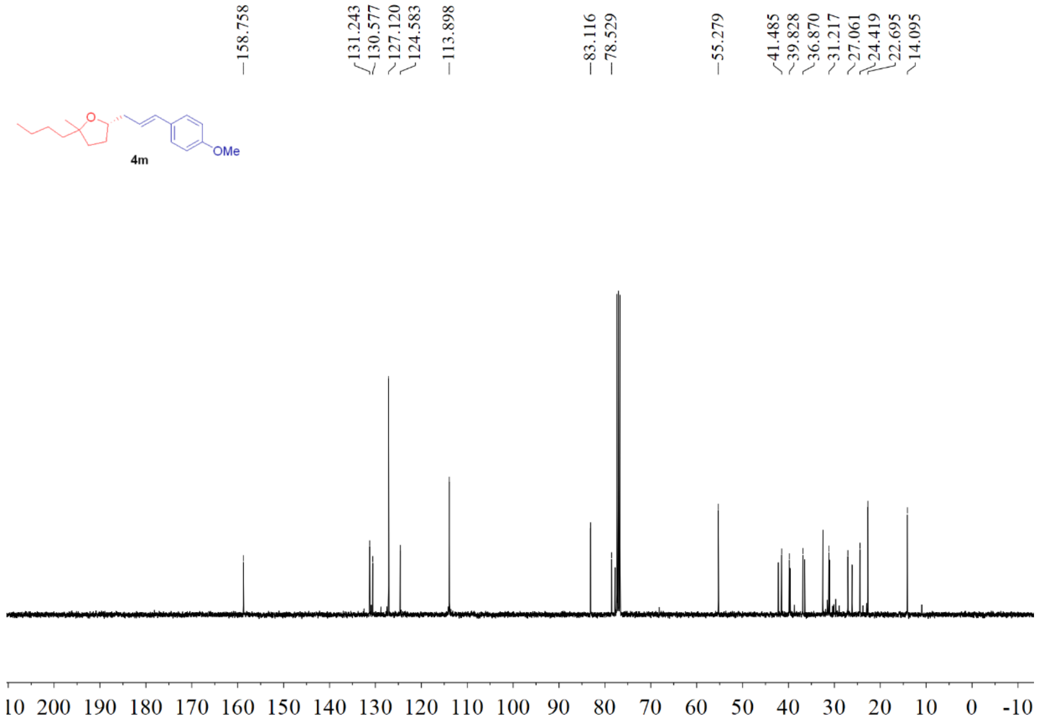
158.758
131.243 130.577 127.120 124.583
113.898
83.116 78.529
55.279
41.485 39.828 36.870 31.217 27.061 24.419 22.695 14.095
OMe
4m
10 200 190 180 170 160 150 140 130 120 110 100 90 80 70 60 50 40 30 20 10 0 -10

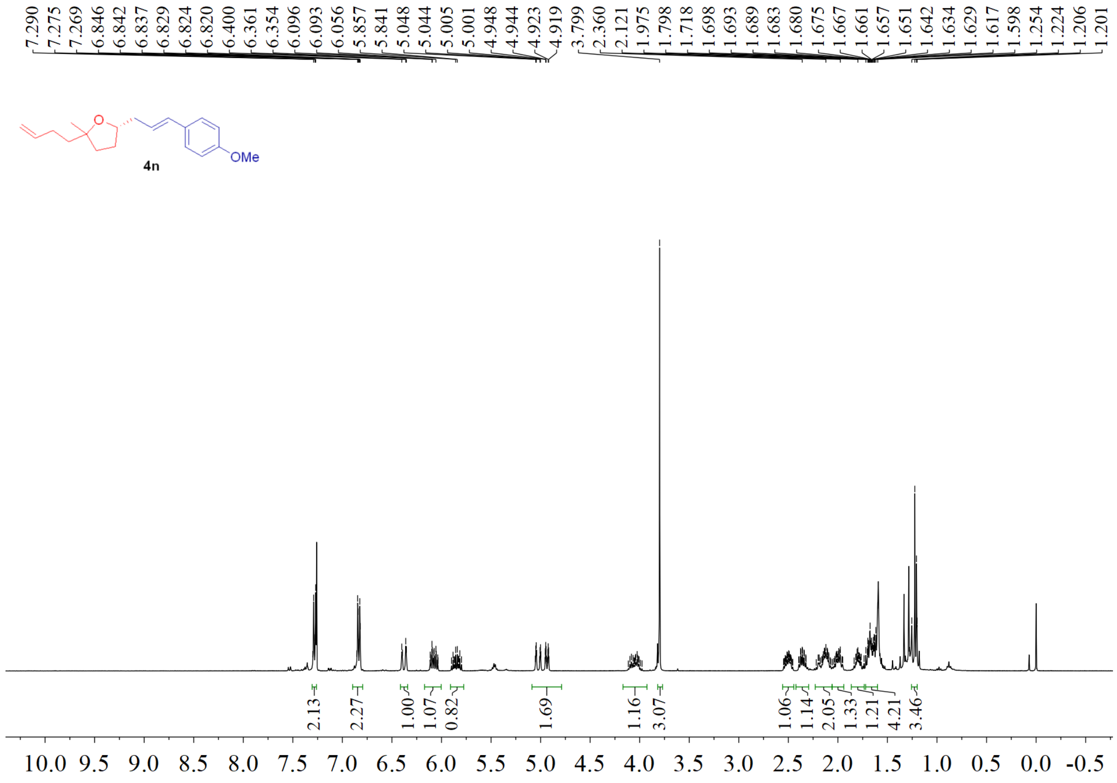
7.290
7.275
7.269
6.846
6.842
6.837
6.829
6.824
6.820
6.400
6.361
6.354
6.096
6.093
6.056
5.857
5.841
5.048
5.044
5.005
5.001
4.948
4.944
4.923
4.919
3.799
2.360
2.121
1.975
1.798
1.718
1.698
1.693
1.689
1.683
1.680
1.675
1.667
1.661
1.657
1.651
1.642
1.634
1.629
1.617
1.598
1.254
1.224
1.206
1.201
OMe
4n
2.13
2.27
1.00
1.07
0.82
1.69
1.16
3.07
1.06
1.14
2.05
1.33
1.21
4.21
3.46
10.0 9.5 9.0 8.5 8.0 7.5 7.0 6.5 6.0 5.5 5.0 4.5 4.0 3.5 3.0 2.5 2.0 1.5 1.0 0.5 0.0 -0.5

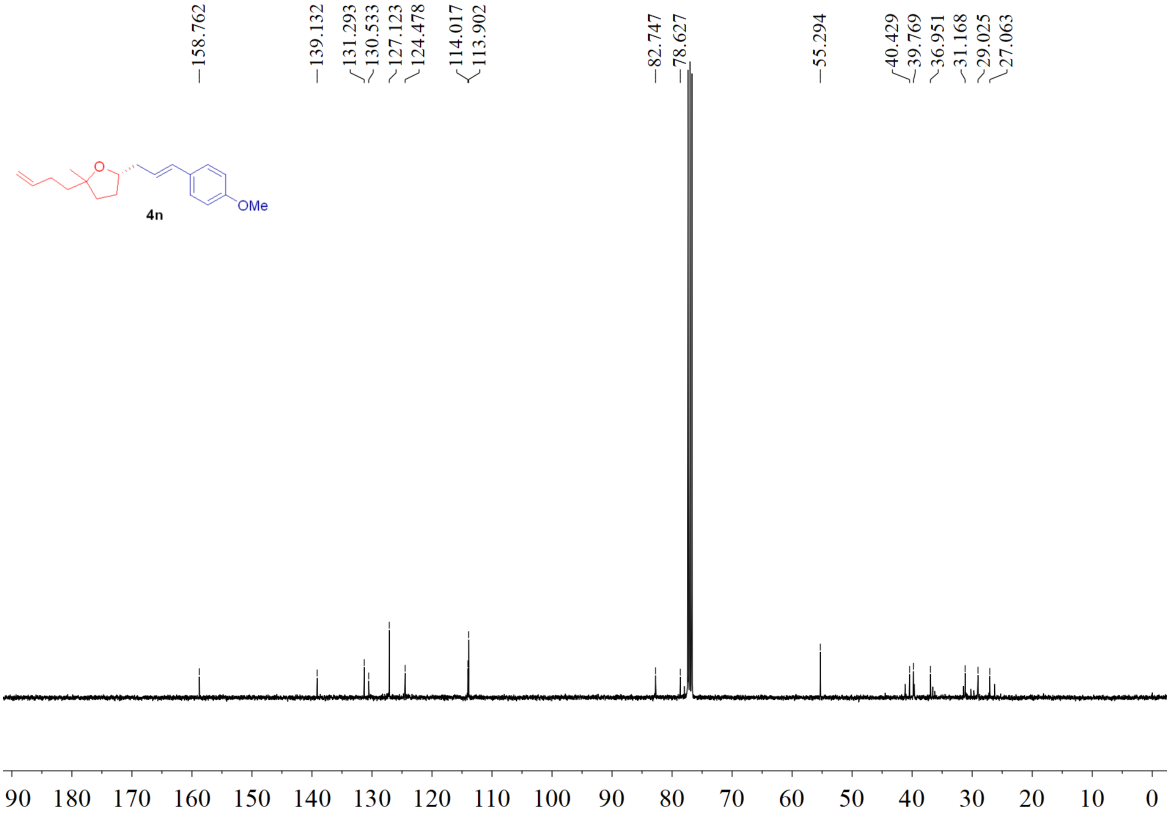
158.762
139.132
131.293
130.533
127.123
124.478
114.017
113.902
82.747
78.627
55.294
40.429
39.769
36.951
31.168
29.025
27.063
OMe
4n
90 180 170 160 150 140 130 120 110 100 90 80 70 60 50 40 30 20 10 0

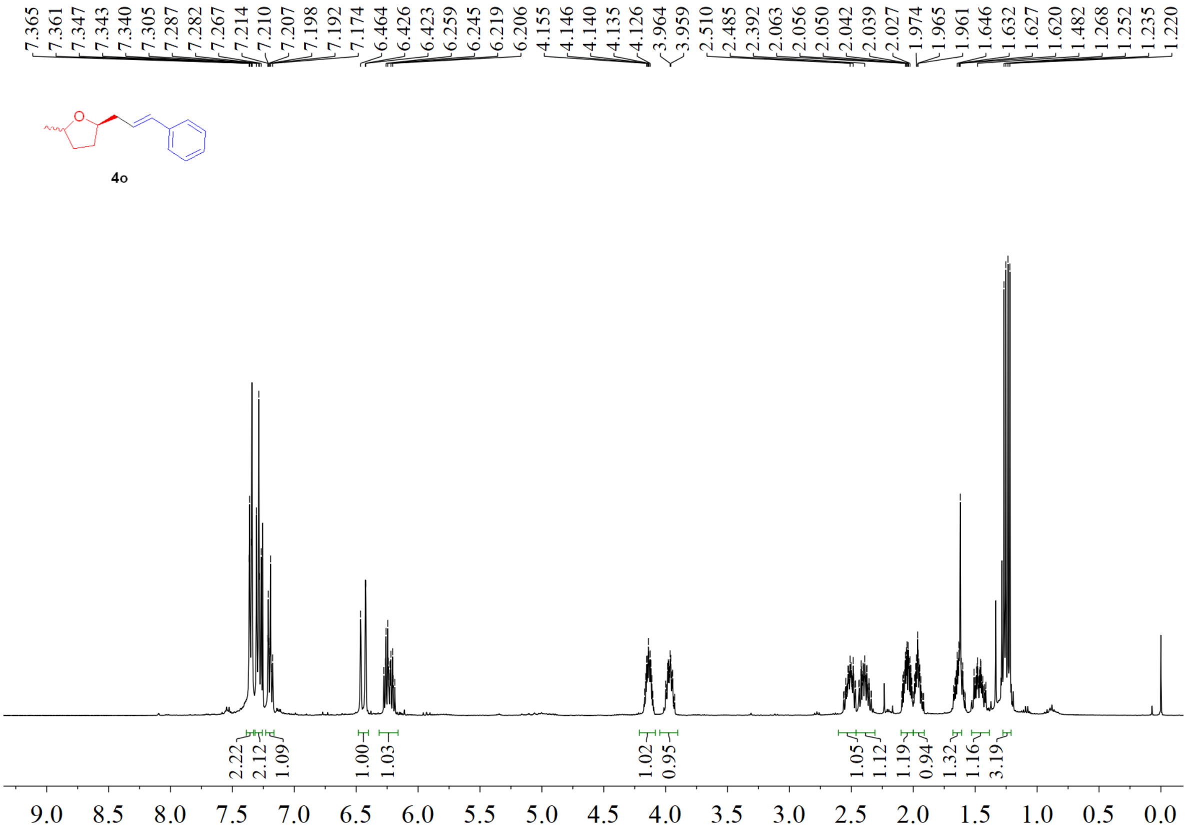
7.365
7.361
7.347
7.343
7.340
7.305
7.287
7.282
7.267
7.214
7.210
7.207
7.198
7.192
7.174
6.464
6.426
6.423
6.259
6.245
6.219
6.206
4.155
4.146
4.140
4.135
4.126
3.964
3.959
2.510
2.485
2.392
2.063
2.056
2.050
2.042
2.039
2.027
1.974
1.965
1.961
1.646
1.632
1.627
1.620
1.482
1.268
1.252
1.235
1.220
4o
2.22
2.12
1.09
1.00
1.03
1.02
0.95
1.05
1.12
1.19
0.94
1.32
1.16
3.19
9.0 8.5 8.0 7.5 7.0 6.5 6.0 5.5 5.0 4.5 4.0 3.5 3.0 2.5 2.0 1.5 1.0 0.5 0.0

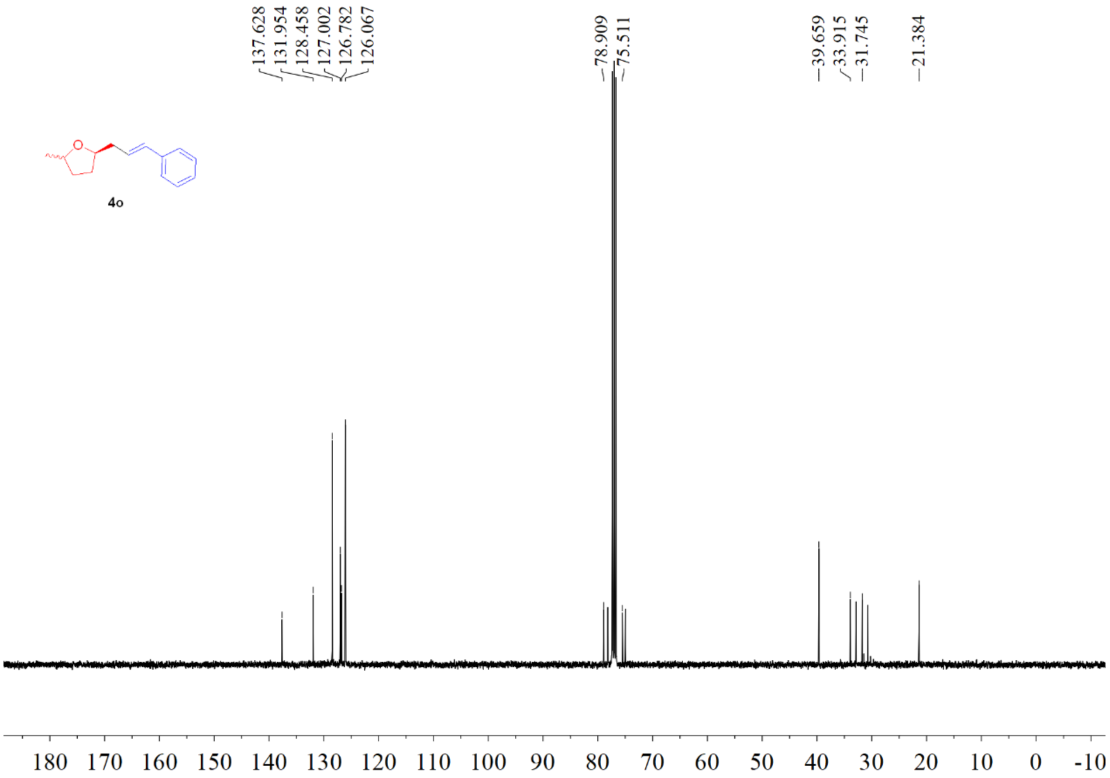
137.628
131.954
128.458
127.002
126.782
126.067
78.909
75.511
39.659
33.915
31.745
21.384
4o
180 170 160 150 140 130 120 110 100 90 80 70 60 50 40 30 20 10 0 -10

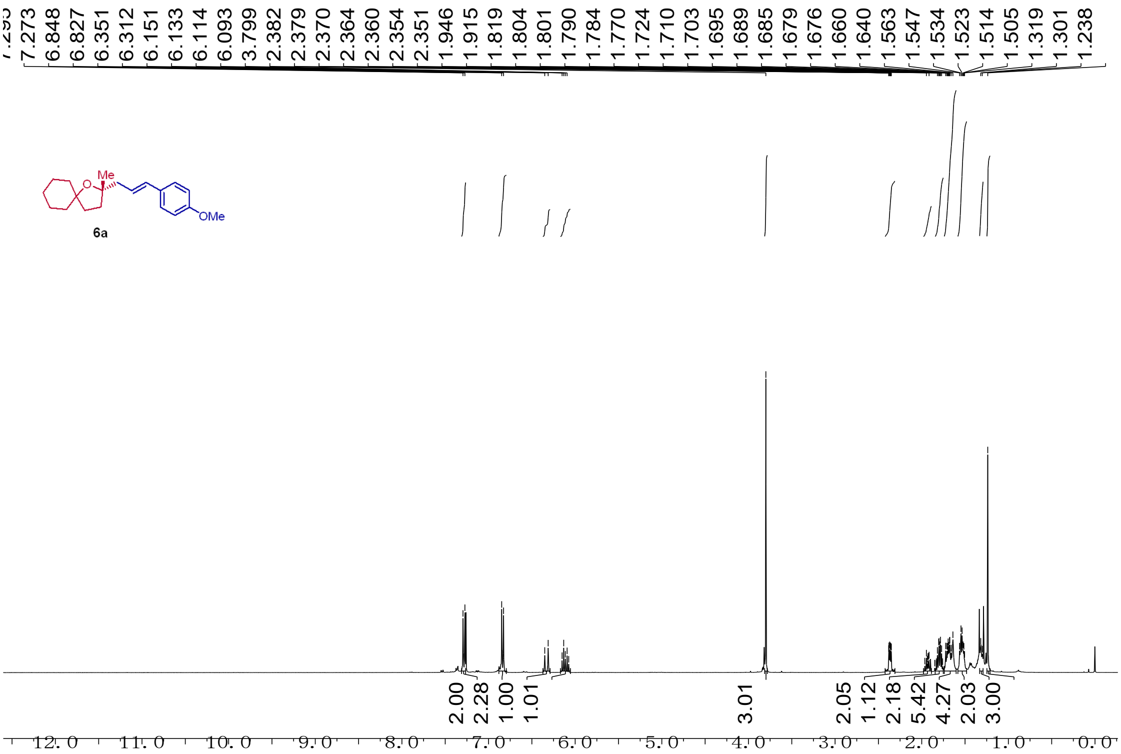
7.273
6.848
6.827
6.351
6.312
6.151
6.133
6.114
6.093
3.799
2.382
2.379
2.370
2.364
2.360
2.354
2.351
1.946
1.915
1.819
1.804
1.801
1.790
1.784
1.770
1.724
1.710
1.703
1.695
1.689
1.685
1.679
1.676
1.660
1.640
1.563
1.547
1.534
1.523
1.514
1.505
1.319
1.301
1.238
Me
O
OMe
6a
2.00
2.28
1.00
1.01
3.01
2.05
1.12
2.18
5.42
4.27
2.03
3.00
12.0
11.0
10.0
9.0
8.0
7.0
6.0
5.0
4.0
3.0
2.0
1.0
0.0

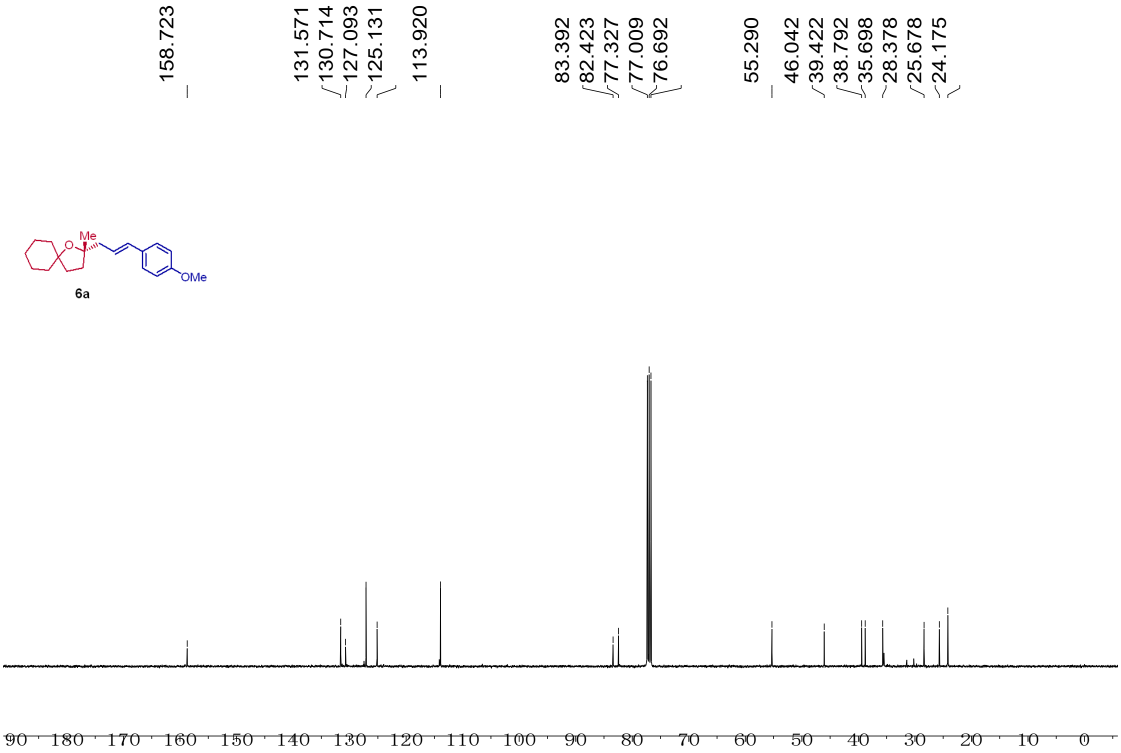
158.723
131.571
130.714
127.093
125.131
113.920
83.392
82.423
77.327
77.009
76.692
55.290
46.042
39.422
38.792
35.698
28.378
25.678
24.175
Me
O
OMe
6a
180
170
160
150
140
130
120
110
100
90
80
70
60
50
40
30
20
10
0

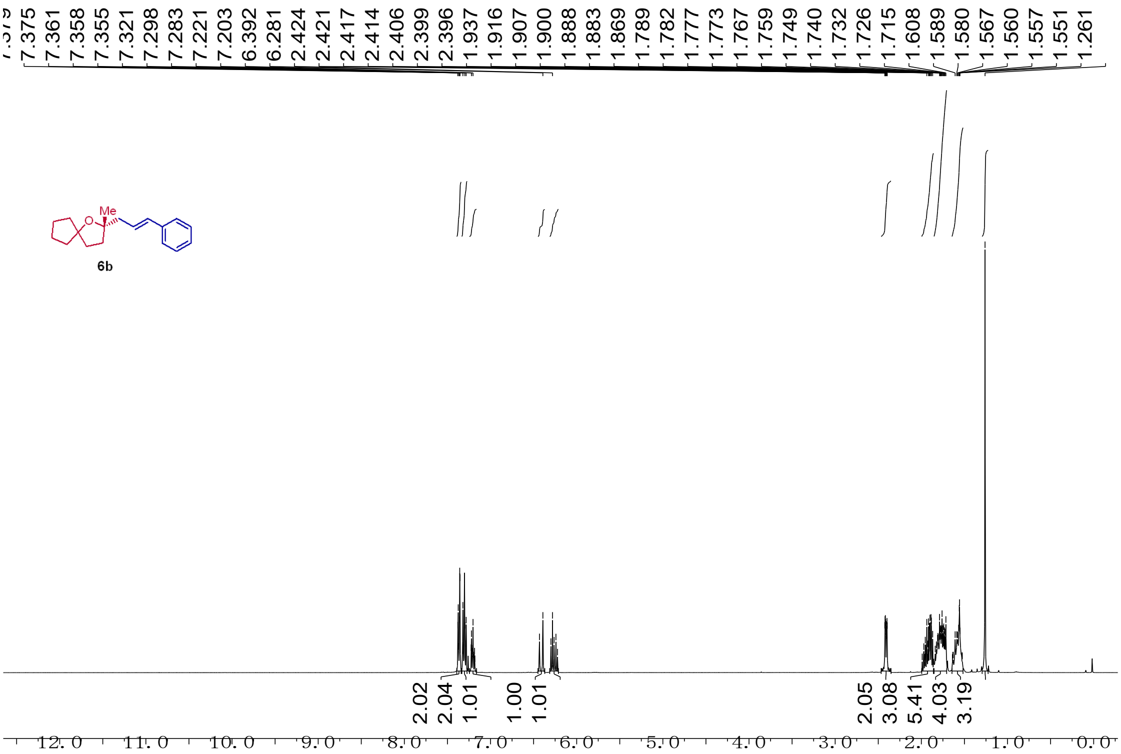

7.375
7.361
7.358
7.355
7.321
7.298
7.283
7.221
7.203
6.392
6.281
2.424
2.421
2.417
2.414
2.406
2.399
2.396
1.937
1.916
1.907
1.900
1.888
1.883
1.869
1.789
1.782
1.777
1.773
1.767
1.759
1.749
1.740
1.732
1.726
1.715
1.608
1.589
1.580
1.567
1.560
1.557
1.551
1.261
Me
O
6b
2.02
2.04
1.01
1.00
1.01
2.05
3.08
5.41
4.03
3.19
12.0 11.0 10.0 9.0 8.0 7.0 6.0 5.0 4.0 3.0 2.0 1.0 0.0

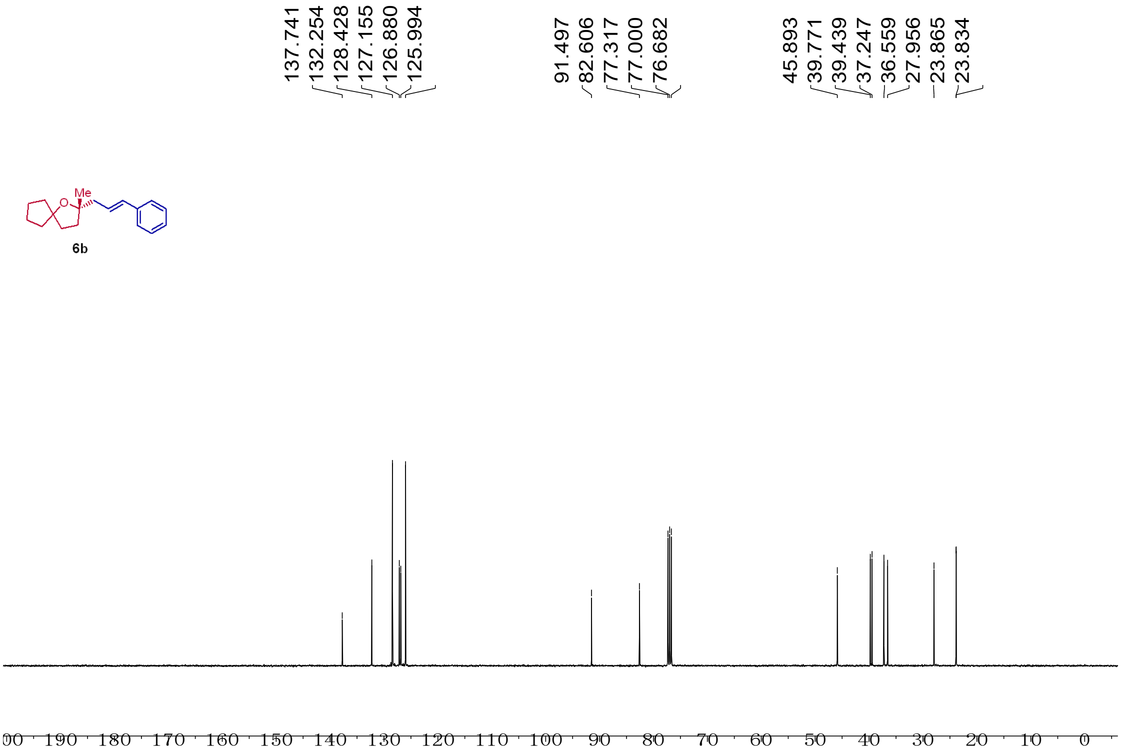

137.741
132.254
128.428
127.155
126.880
125.994
91.497
82.606
77.317
77.000
76.682
45.893
39.771
39.439
37.247
36.559
27.956
23.865
23.834
Me
O
6b
190 180 170 160 150 140 130 120 110 100 90 80 70 60 50 40 30 20 10 0

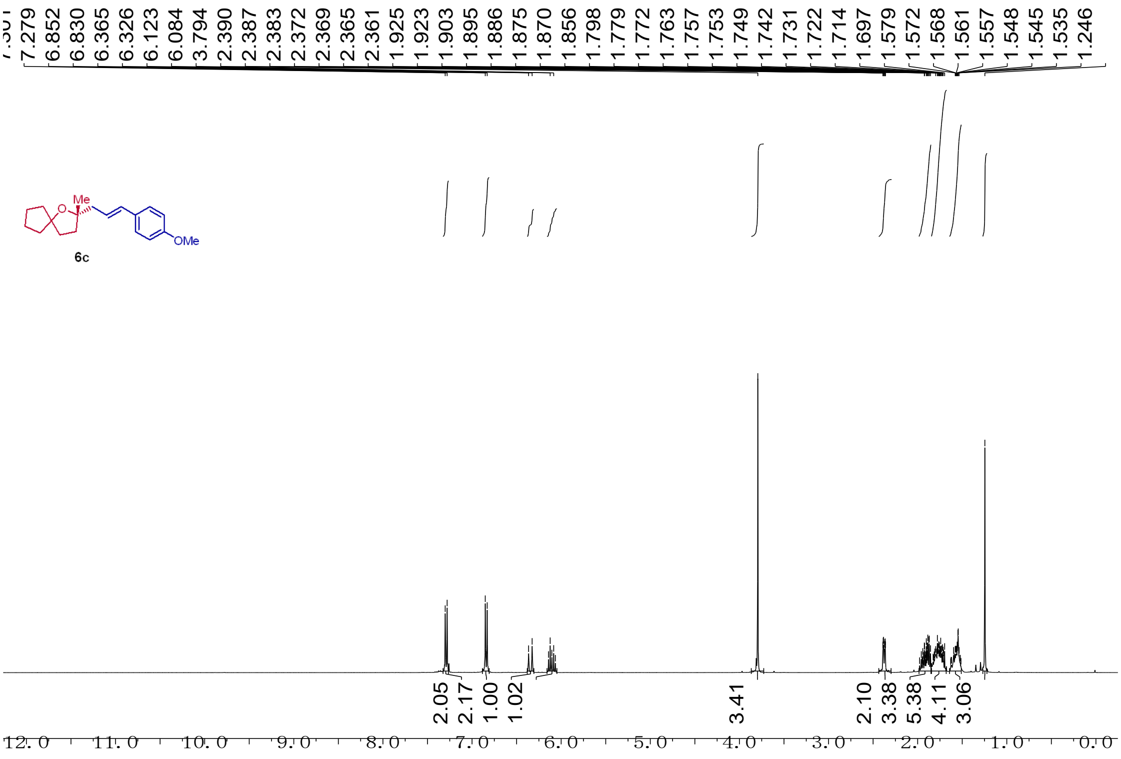
7.279
6.852
6.830
6.365
6.326
6.123
6.084
3.794
2.390
2.387
2.383
2.372
2.369
2.365
2.361
1.925
1.923
1.903
1.895
1.886
1.875
1.870
1.856
1.798
1.779
1.772
1.763
1.757
1.753
1.749
1.742
1.731
1.722
1.714
1.697
1.579
1.572
1.568
1.561
1.557
1.548
1.545
1.535
1.246
Me
O
OMe
6c
2.05
2.17
1.00
1.02
3.41
2.10
3.38
5.38
4.11
3.06
12.0 11.0 10.0 9.0 8.0 7.0 6.0 5.0 4.0 3.0 2.0 1.0 0.0

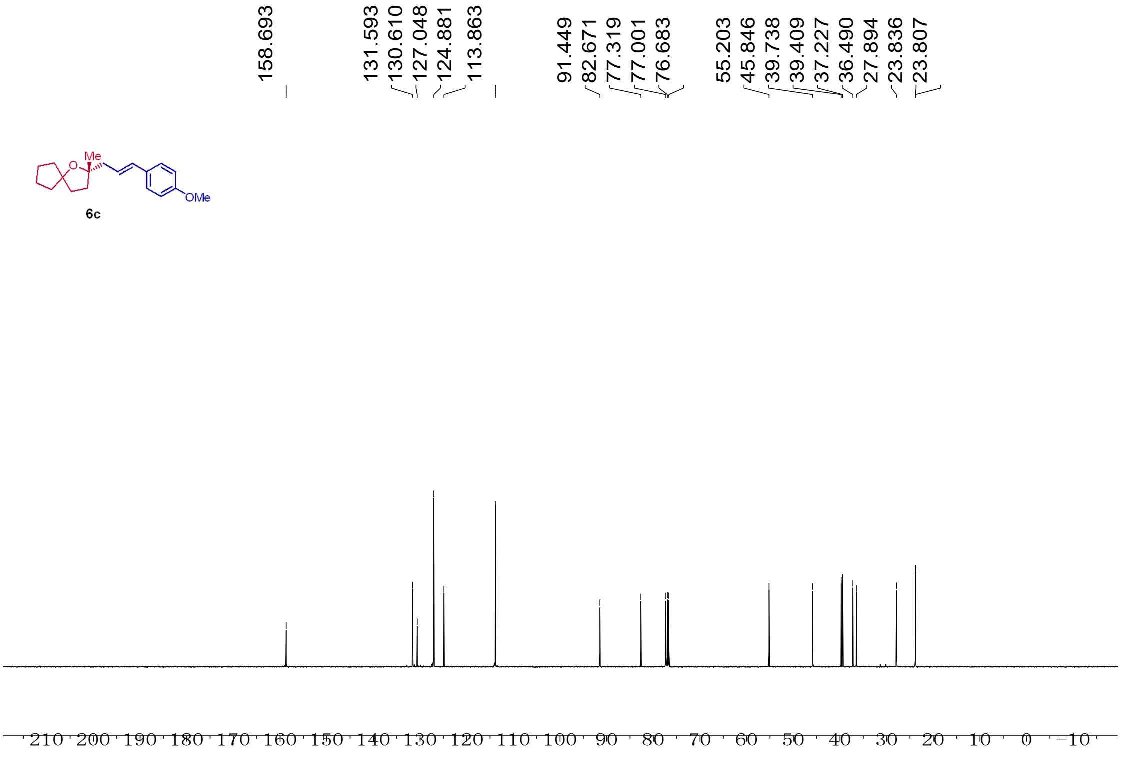
158.693
131.593
130.610
127.048
124.881
113.863
91.449
82.671
77.319
77.001
76.683
55.203
45.846
39.738
39.409
37.227
36.490
27.894
23.836
23.807
Me
O
OMe
6c
210 200 190 180 170 160 150 140 130 120 110 100 90 80 70 60 50 40 30 20 10 0 -10

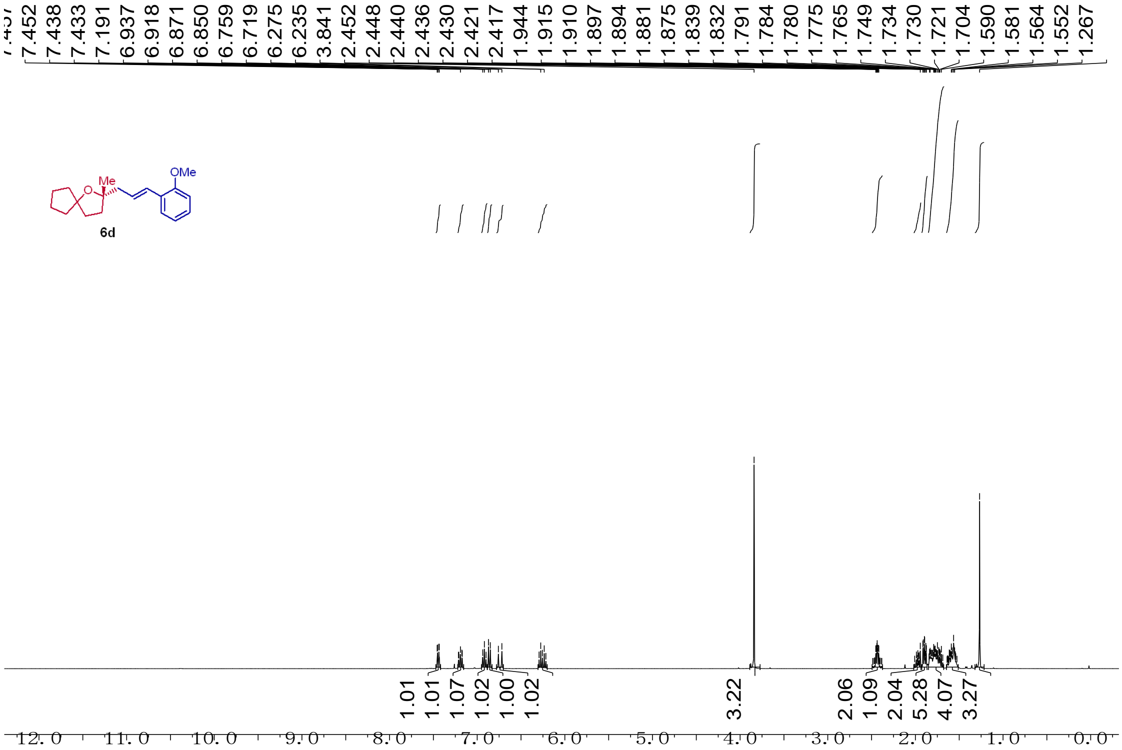
7.452
7.438
7.433
7.191
6.937
6.918
6.871
6.850
6.759
6.719
6.275
6.235
3.841
2.452
2.448
2.440
2.436
2.430
2.421
2.417
1.944
1.915
1.910
1.897
1.894
1.881
1.875
1.839
1.832
1.791
1.784
1.780
1.775
1.765
1.749
1.734
1.730
1.721
1.704
1.590
1.581
1.564
1.552
1.267
1.01
1.01
1.07
1.02
1.00
1.02
3.22
2.06
1.09
2.04
5.28
4.07
3.27
12.0 11.0 10.0 9.0 8.0 7.0 6.0 5.0 4.0 3.0 2.0 1.0 0.0

OMe
O
Me

6d

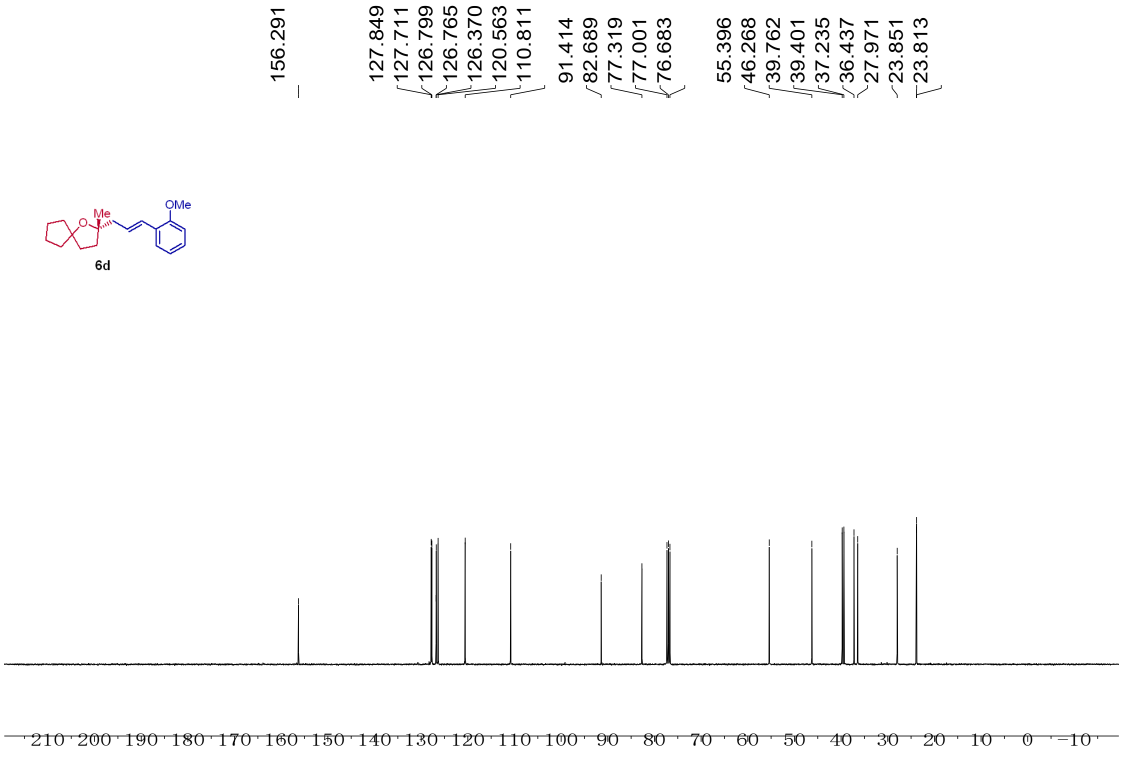
156.291
127.849
127.711
126.799
126.765
126.370
120.563
110.811
91.414
82.689
77.319
77.001
76.683
55.396
46.268
39.762
39.401
37.235
36.437
27.971
23.851
23.813
210 200 190 180 170 160 150 140 130 120 110 100 90 80 70 60 50 40 30 20 10 0 −10

OMe
O
Me

6d

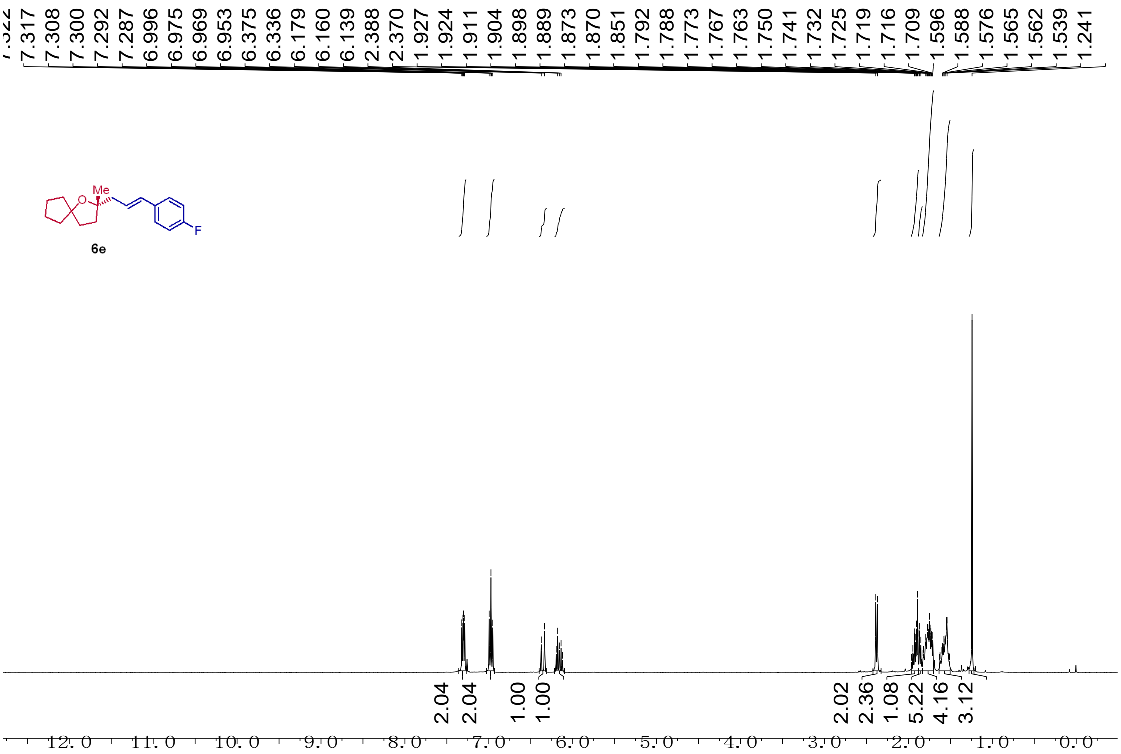
7.317
7.308
7.300
7.292
7.287
6.996
6.975
6.969
6.953
6.375
6.336
6.179
6.160
6.139
2.388
2.370
1.927
1.924
1.911
1.904
1.898
1.889
1.873
1.870
1.851
1.792
1.788
1.773
1.767
1.763
1.750
1.741
1.732
1.725
1.719
1.716
1.709
1.596
1.588
1.576
1.565
1.562
1.539
1.241
Me
O
F
6e
2.04
2.04
1.00
1.00
2.02
2.36
1.08
5.22
4.16
3.12
12.0
11.0
10.0
9.0
8.0
7.0
6.0
5.0
4.0
3.0
2.0
1.0
0.0

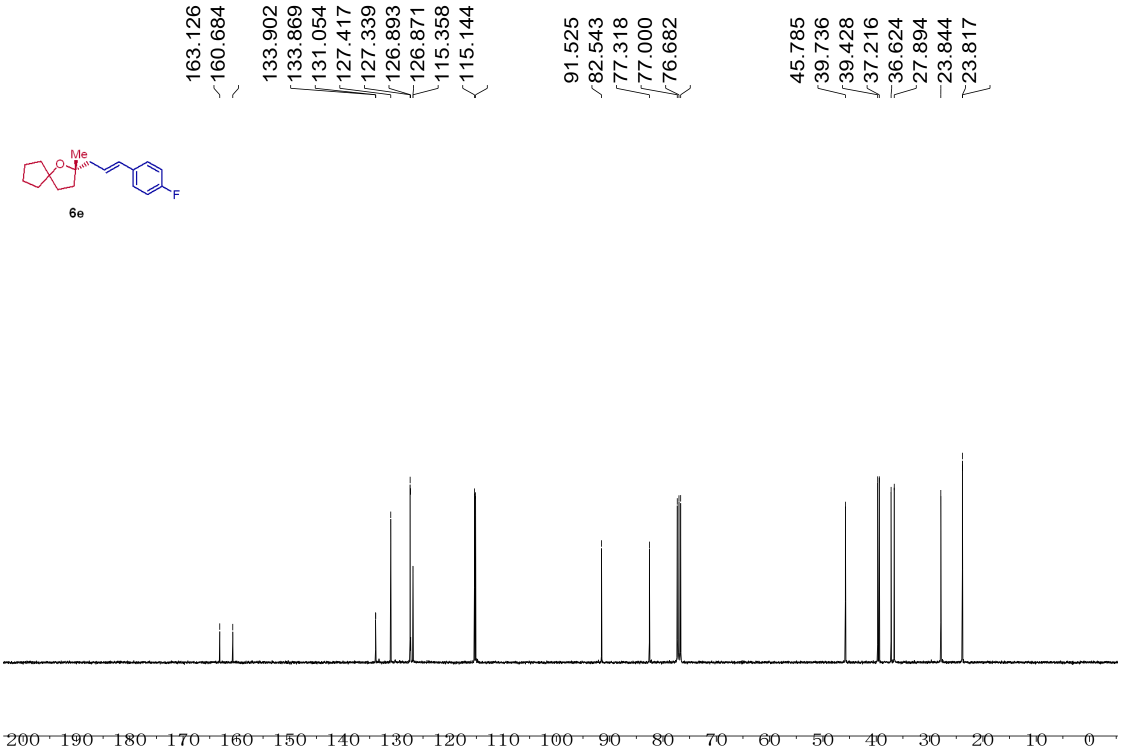
163.126
160.684
133.902
133.869
131.054
127.417
127.339
126.893
126.871
115.358
115.144
91.525
82.543
77.318
77.000
76.682
45.785
39.736
39.428
37.216
36.624
27.894
23.844
23.817
Me
O
F
6e
200
190
180
170
160
150
140
130
120
110
100
90
80
70
60
50
40
30
20
10
0

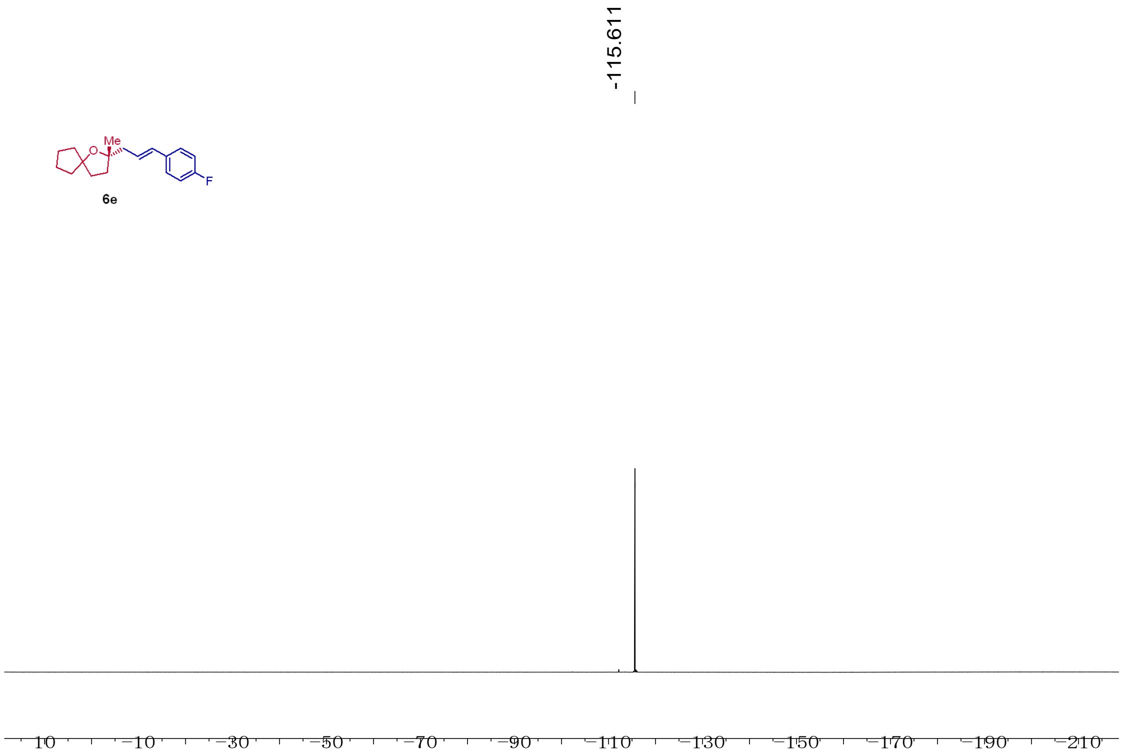
-115.611
Me
O
F
6e
10 −10 −30 −50 −70 −90 −110 −130 −150 −170 −190 −210

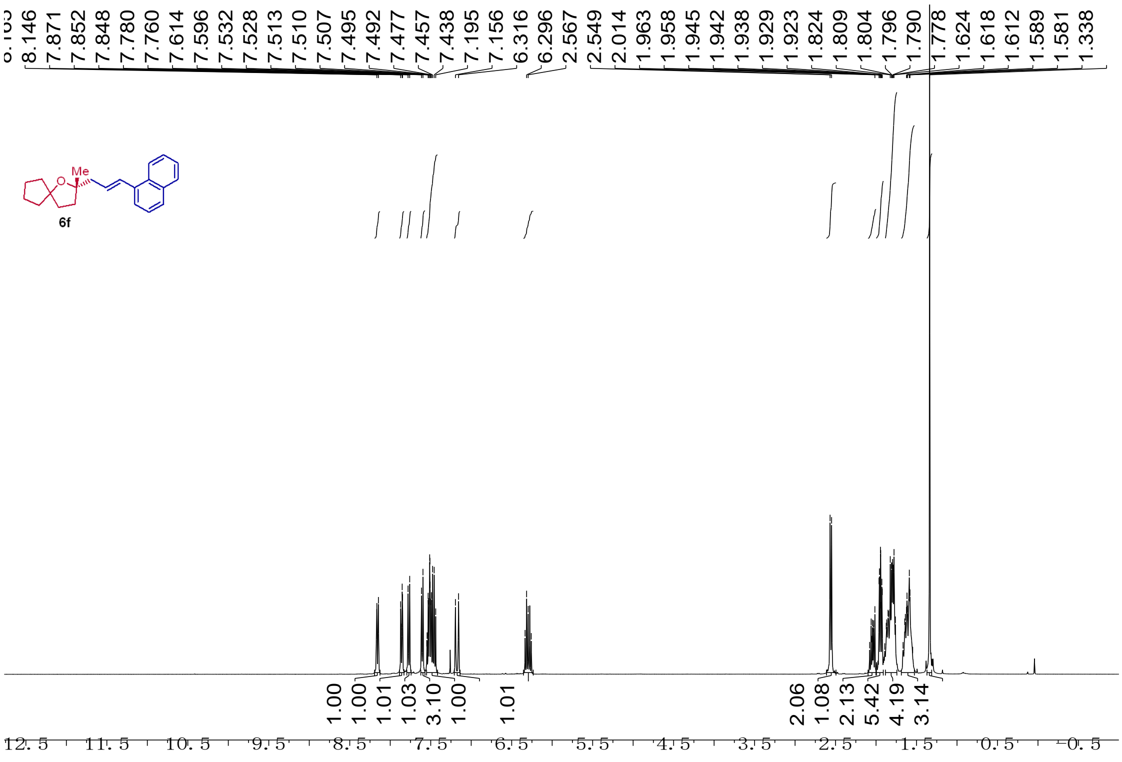
8.146 7.871 7.852 7.848 7.780 7.760 7.614 7.596 7.532 7.528 7.513 7.510 7.507 7.495 7.492 7.477 7.457 7.438 7.195 7.156 6.316 6.296 2.567 2.549 2.014 1.963 1.958 1.945 1.942 1.938 1.929 1.923 1.824 1.809 1.804 1.796 1.790 1.778 1.624 1.618 1.612 1.589 1.581 1.338
Me
O
6f
1.00 1.00 1.01 1.03 3.10 1.00 1.01 2.06 1.08 2.13 5.42 4.19 3.14
12.5 11.5 10.5 9.5 8.5 7.5 6.5 5.5 4.5 3.5 2.5 1.5 0.5 −0.5

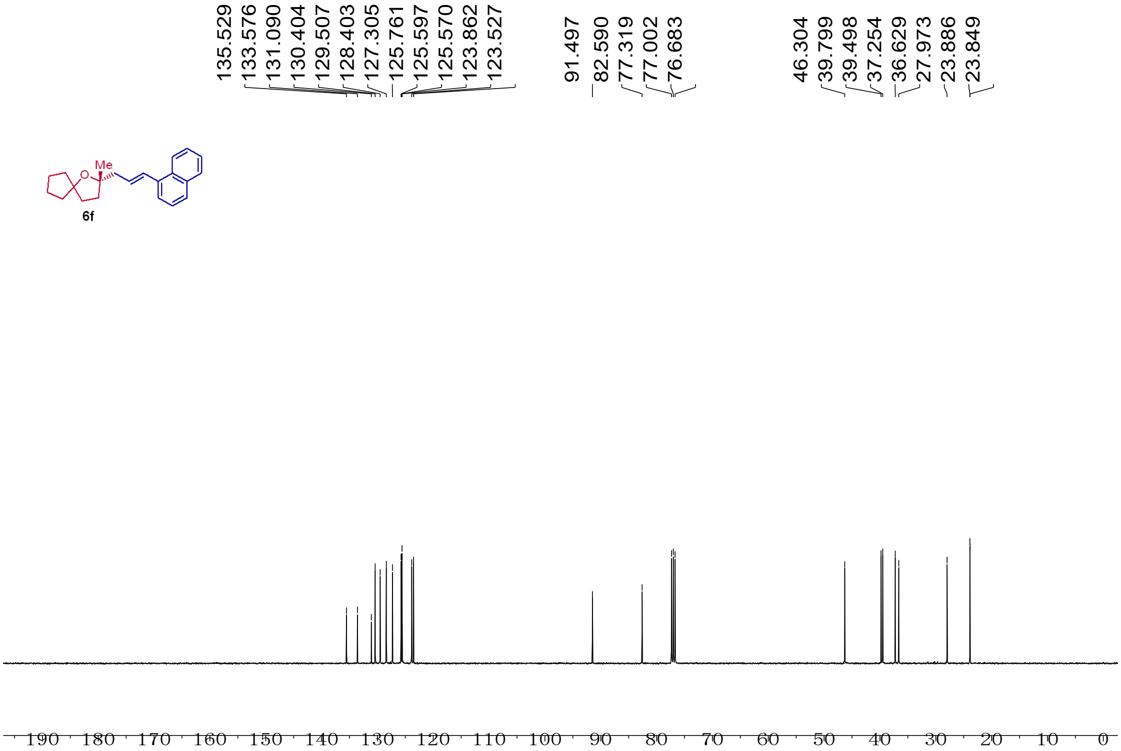
135.529
133.576
131.090
130.404
129.507
128.403
127.305
125.761
125.597
125.570
123.862
123.527
91.497
82.590
77.319
77.002
76.683
46.304
39.799
39.498
37.254
36.629
27.973
23.886
23.849
Me
O
6f
190 180 170 160 150 140 130 120 110 100 90 80 70 60 50 40 30 20 10 0

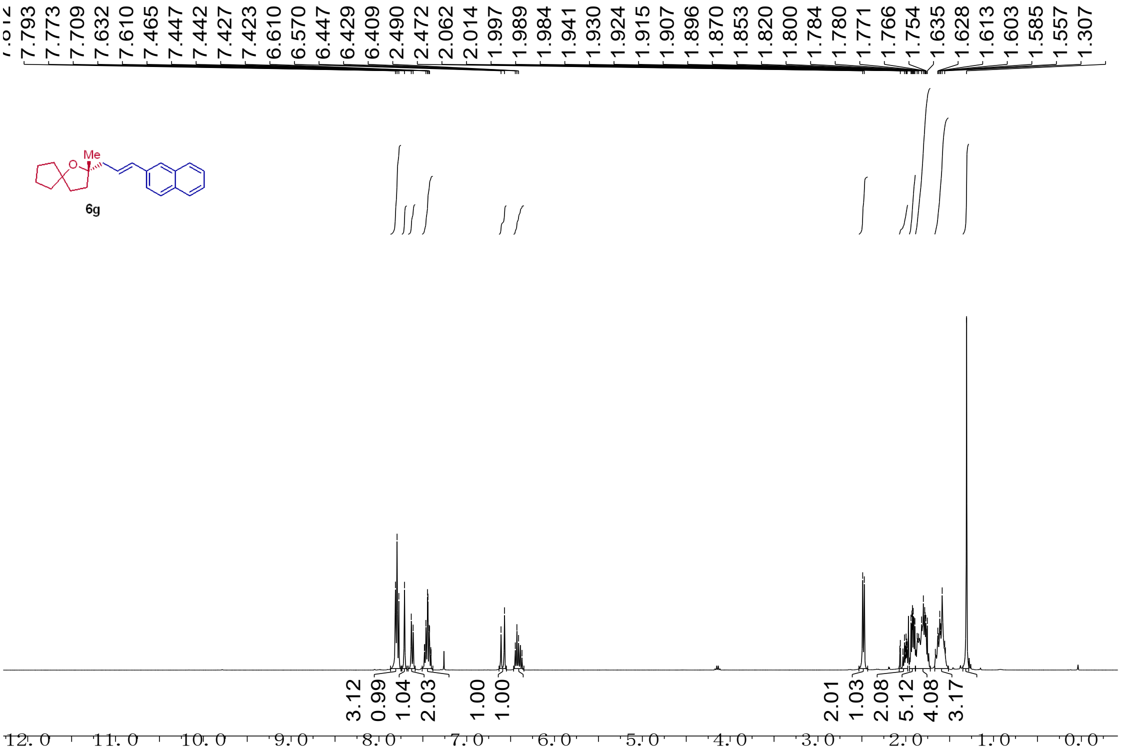
7.793
7.773
7.709
7.632
7.610
7.465
7.447
7.442
7.427
7.423
6.610
6.570
6.447
6.429
6.409
2.490
2.472
2.062
2.014
1.997
1.989
1.984
1.941
1.930
1.924
1.915
1.907
1.896
1.870
1.853
1.820
1.800
1.784
1.780
1.771
1.766
1.754
1.635
1.628
1.613
1.603
1.585
1.557
1.307
Me
O
6g
3.12
0.99
1.04
2.03
1.00
1.00
2.01
1.03
2.08
5.12
4.08
3.17
12.0 11.0 10.0 9.0 8.0 7.0 6.0 5.0 4.0 3.0 2.0 1.0 0.0

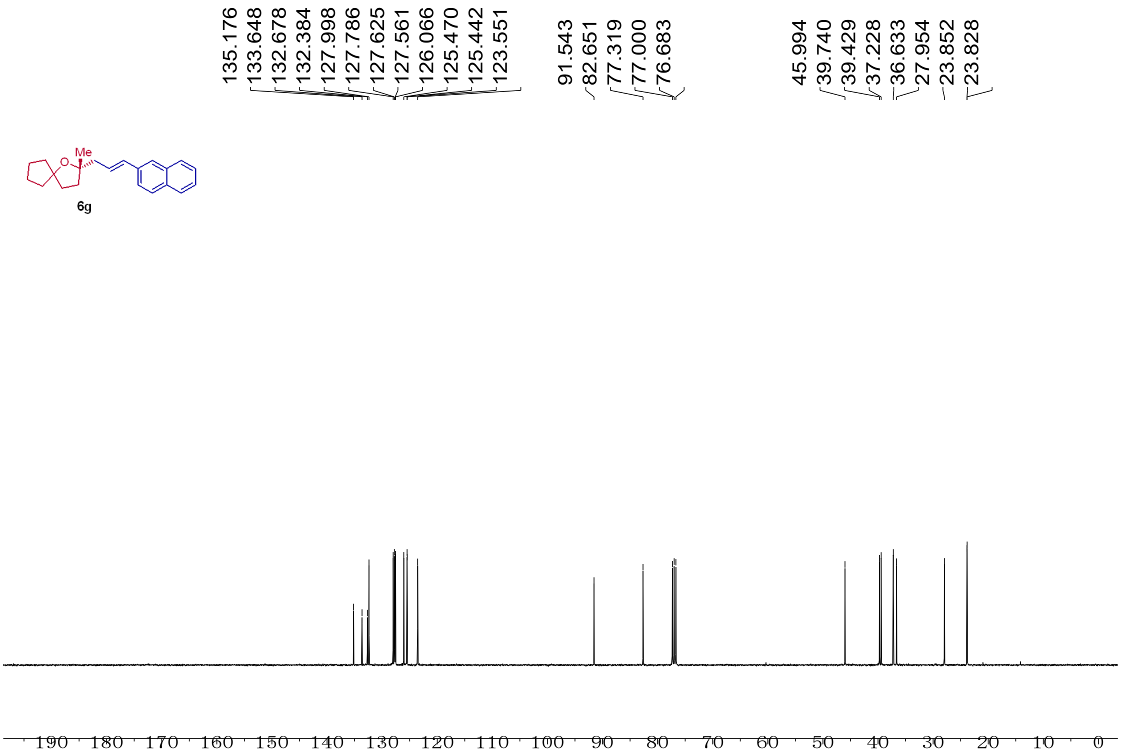
135.176
133.648
132.678
132.384
127.998
127.786
127.625
127.561
126.066
125.470
125.442
123.551
91.543
82.651
77.319
77.000
76.683
45.994
39.740
39.429
37.228
36.633
27.954
23.852
23.828
Me
O
6g
190 180 170 160 150 140 130 120 110 100 90 80 70 60 50 40 30 20 10 0

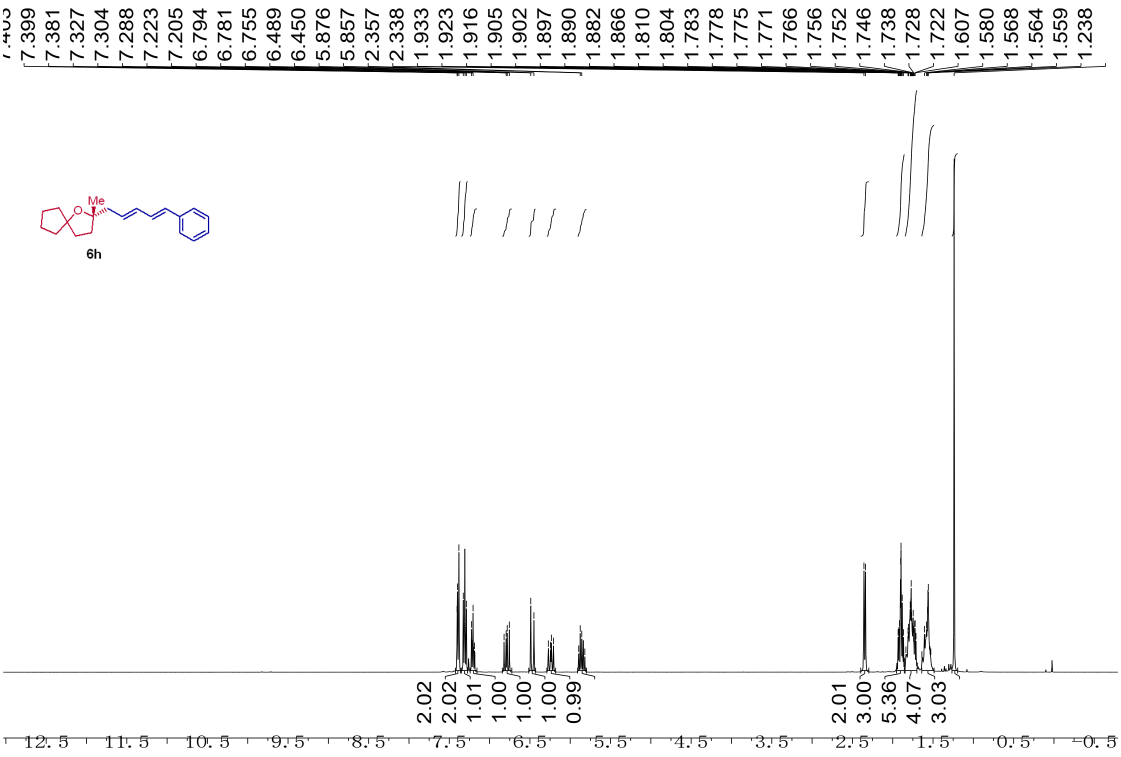
7.399
7.381
7.327
7.304
7.288
7.223
7.205
6.794
6.781
6.755
6.489
6.450
5.876
5.857
2.357
2.338
1.933
1.923
1.916
1.905
1.902
1.897
1.890
1.882
1.866
1.810
1.804
1.783
1.778
1.775
1.771
1.766
1.756
1.752
1.746
1.738
1.728
1.722
1.607
1.580
1.568
1.564
1.559
1.238
Me
O
6h
2.02
2.02
1.01
1.00
1.00
1.00
0.99
2.01
3.00
5.36
4.07
3.03
12.5 11.5 10.5 9.5 8.5 7.5 6.5 5.5 4.5 3.5 2.5 1.5 0.5 -0.5

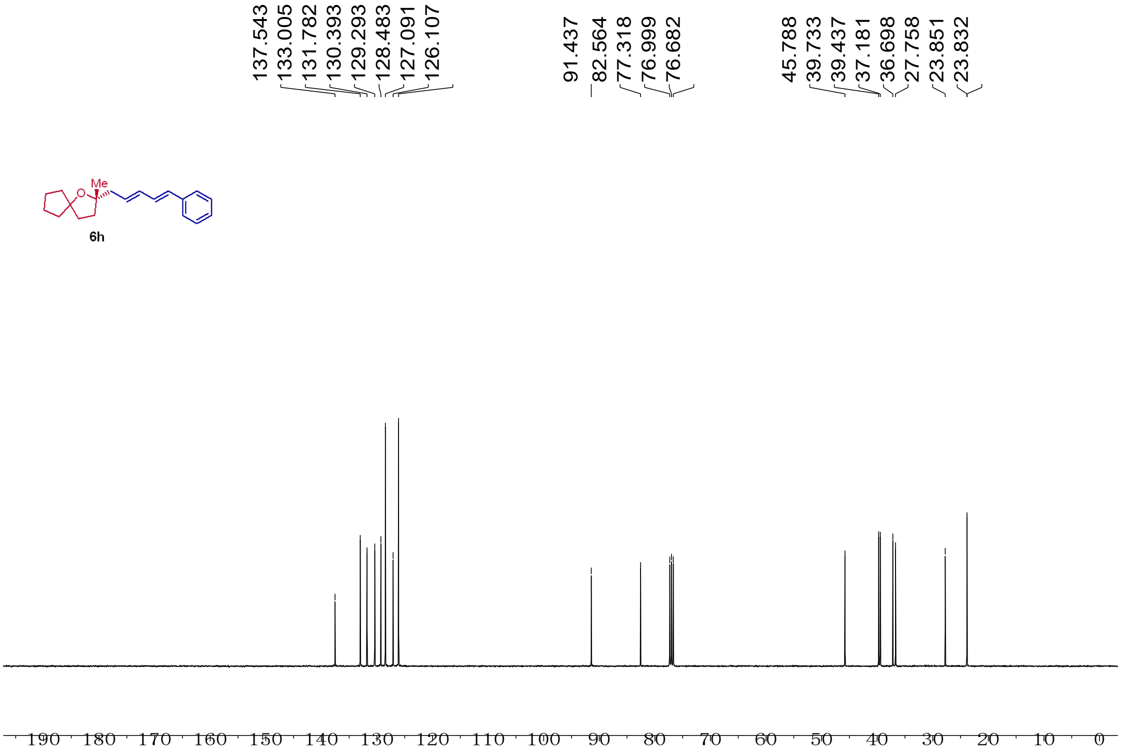
137.543
133.005
131.782
130.393
129.293
128.483
127.091
126.107
91.437
82.564
77.318
76.999
76.682
45.788
39.733
39.437
37.181
36.698
27.758
23.851
23.832
Me
O
6h
190 180 170 160 150 140 130 120 110 100 90 80 70 60 50 40 30 20 10 0

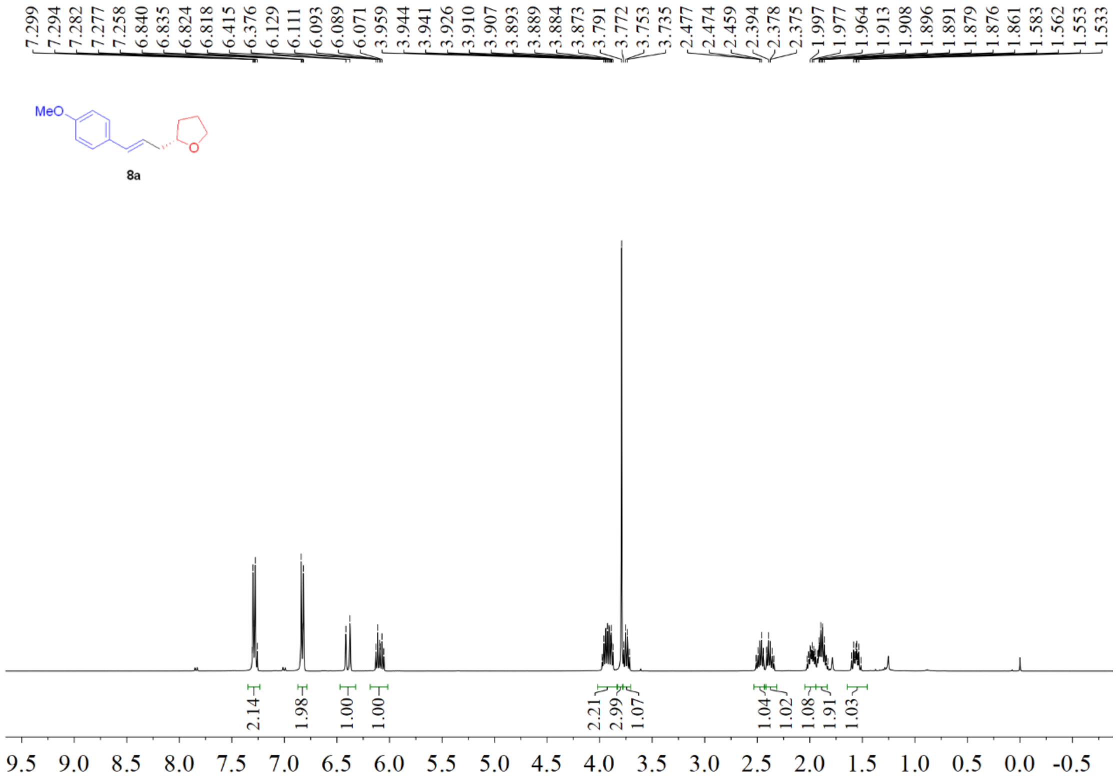
7.299 7.294 7.282 7.277 7.258 6.840 6.835 6.824 6.818 6.415 6.376 6.129 6.111 6.093 6.089 6.071 3.959 3.944 3.941 3.926 3.910 3.907 3.893 3.889 3.884 3.873 3.791 3.772 3.753 3.735 2.477 2.474 2.459 2.394 2.378 2.375 1.997 1.977 1.964 1.913 1.908 1.896 1.891 1.879 1.876 1.861 1.583 1.562 1.553 1.533
MeO
8a
2.14 1.98 1.00 1.00 2.21 2.99 1.07 1.04 1.02 1.08 1.91 1.03
9.5 9.0 8.5 8.0 7.5 7.0 6.5 6.0 5.5 5.0 4.5 4.0 3.5 3.0 2.5 2.0 1.5 1.0 0.5 0.0 -0.5

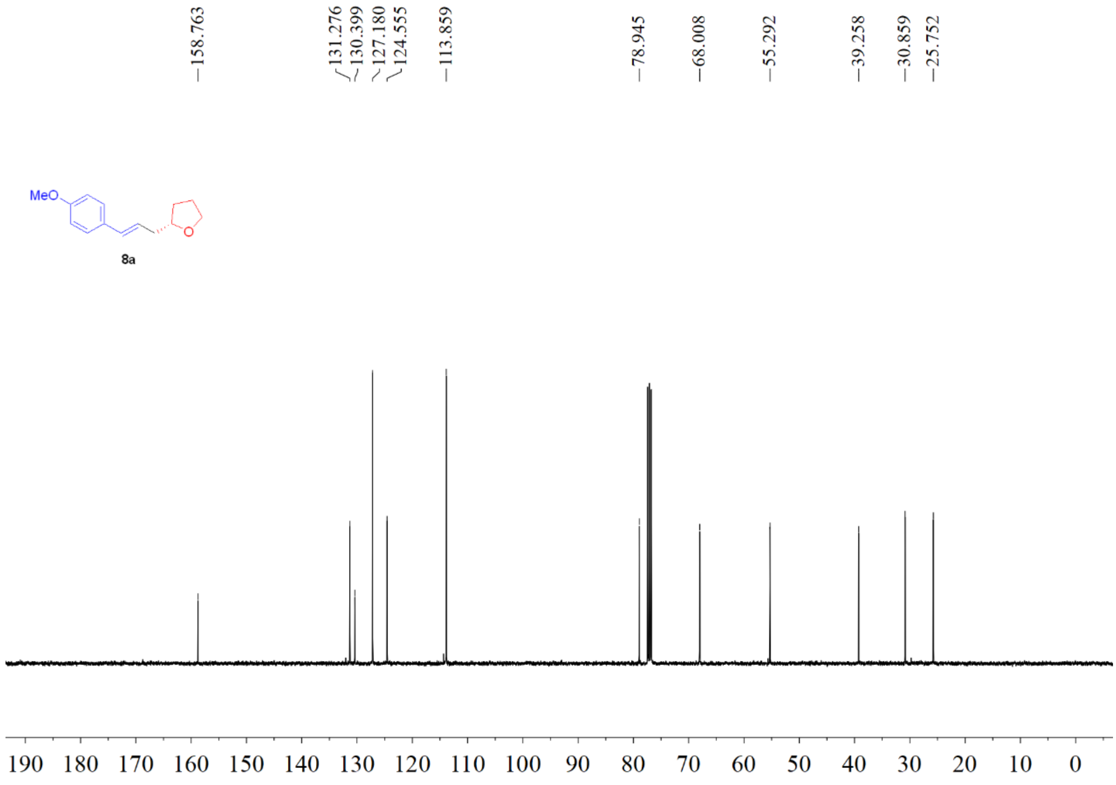
158.763
131.276 130.399 127.180 124.555
113.859
78.945
68.008
55.292
39.258
30.859
25.752
MeO
8a
190 180 170 160 150 140 130 120 110 100 90 80 70 60 50 40 30 20 10 0

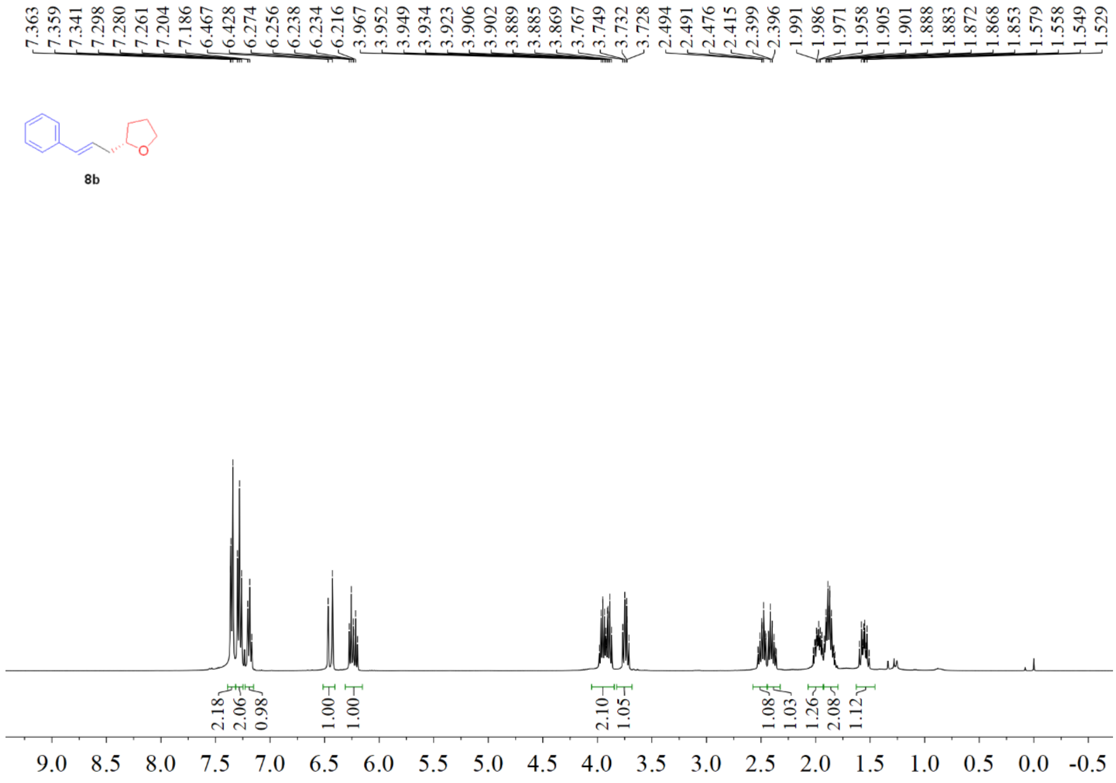
7.363
7.359
7.341
7.298
7.280
7.261
7.204
7.186
6.467
6.428
6.274
6.256
6.238
6.234
6.216
3.967
3.952
3.949
3.934
3.923
3.906
3.902
3.889
3.885
3.869
3.767
3.749
3.732
3.728
2.494
2.491
2.476
2.415
2.399
2.396
1.991
1.986
1.971
1.958
1.905
1.901
1.888
1.883
1.872
1.868
1.853
1.579
1.558
1.549
1.529
8b
2.18
2.06
0.98
1.00
1.00
2.10
1.05
1.08
1.03
1.26
2.08
1.12
9.0 8.5 8.0 7.5 7.0 6.5 6.0 5.5 5.0 4.5 4.0 3.5 3.0 2.5 2.0 1.5 1.0 0.5 0.0 -0.5

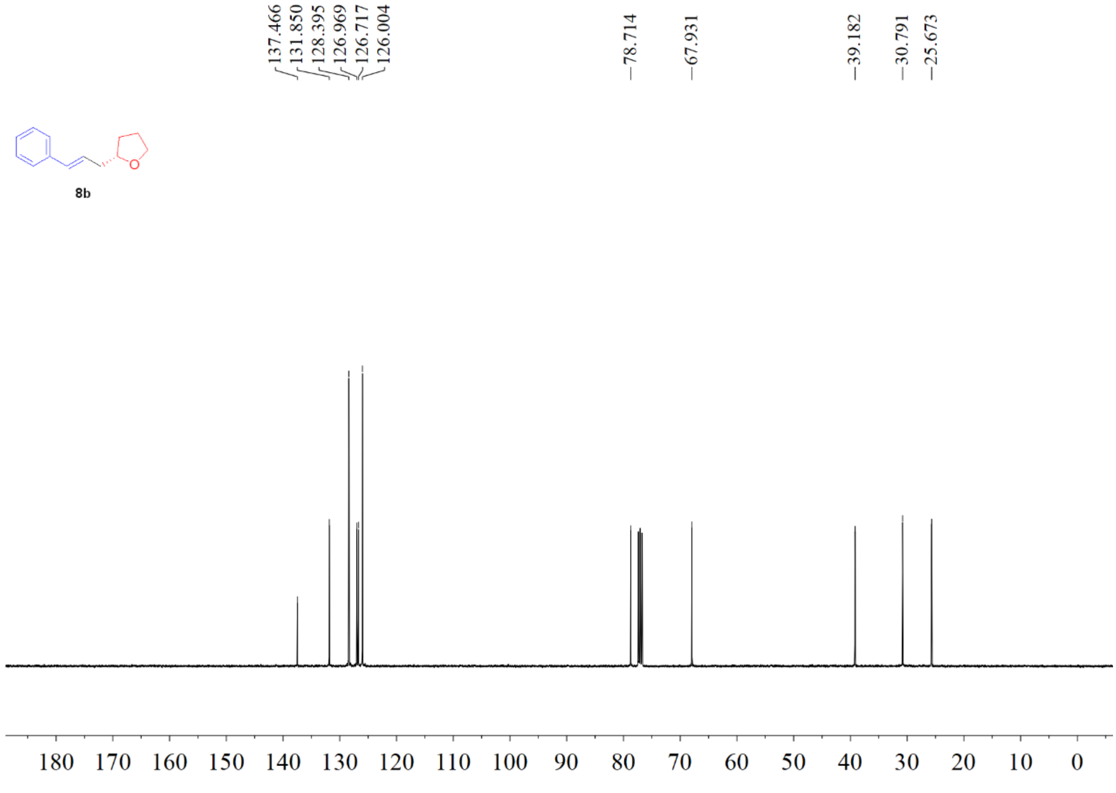
137.466
131.850
128.395
126.969
126.717
126.004
78.714
67.931
39.182
30.791
25.673
8b
180 170 160 150 140 130 120 110 100 90 80 70 60 50 40 30 20 10 0

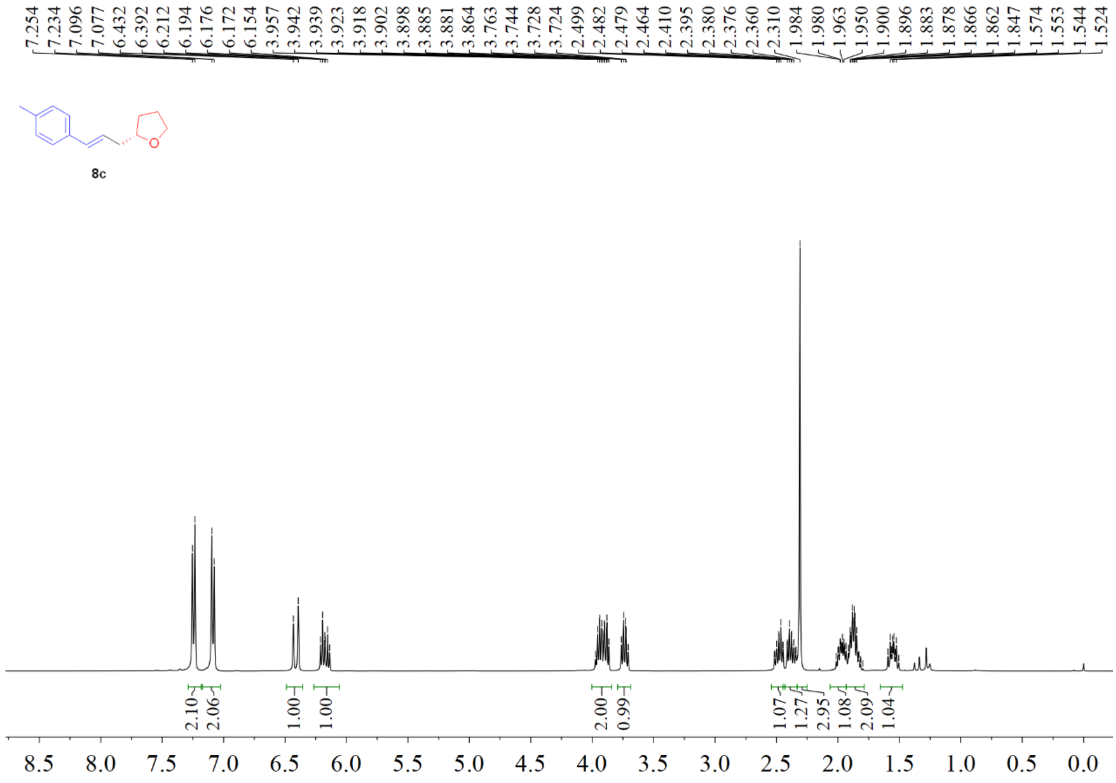
7.254
7.234
7.096
7.077
6.432
6.392
6.212
6.194
6.176
6.172
6.154
3.957
3.942
3.939
3.923
3.918
3.902
3.898
3.885
3.881
3.864
3.763
3.744
3.728
3.724
2.499
2.482
2.479
2.464
2.410
2.395
2.380
2.376
2.360
2.310
1.984
1.980
1.963
1.950
1.900
1.896
1.883
1.878
1.866
1.862
1.847
1.574
1.553
1.544
1.524
8c
2.10
2.06
1.00
1.00
2.00
0.99
1.07
1.27
2.95
1.08
2.09
1.04
8.5 8.0 7.5 7.0 6.5 6.0 5.5 5.0 4.5 4.0 3.5 3.0 2.5 2.0 1.5 1.0 0.5 0.0
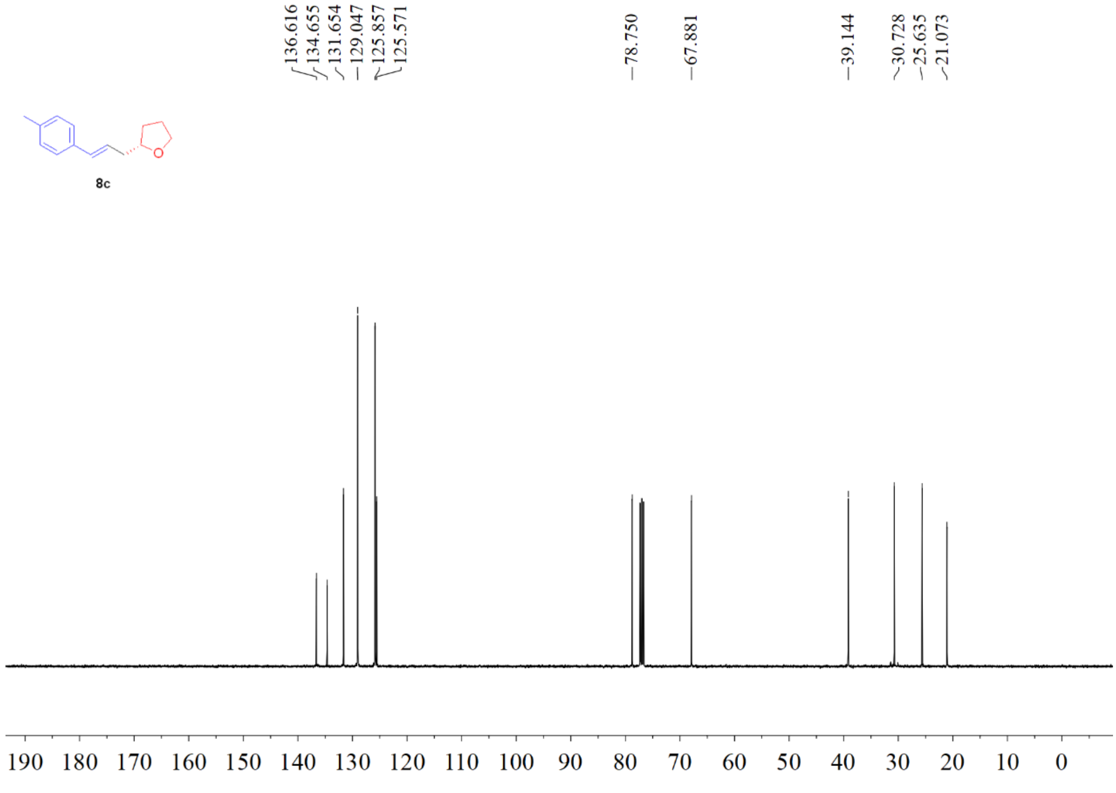
136.616
134.655
131.654
129.047
125.857
125.571
78.750
67.881
39.144
30.728
25.635
21.073
8c
190 180 170 160 150 140 130 120 110 100 90 80 70 60 50 40 30 20 10 0

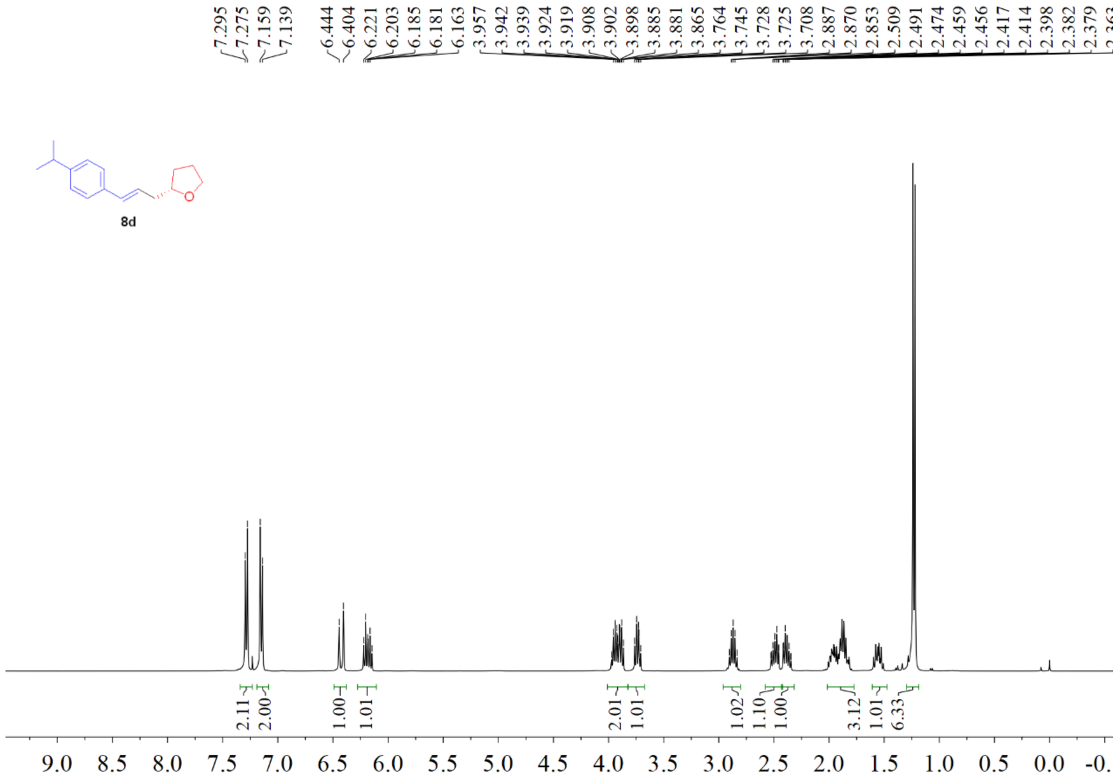

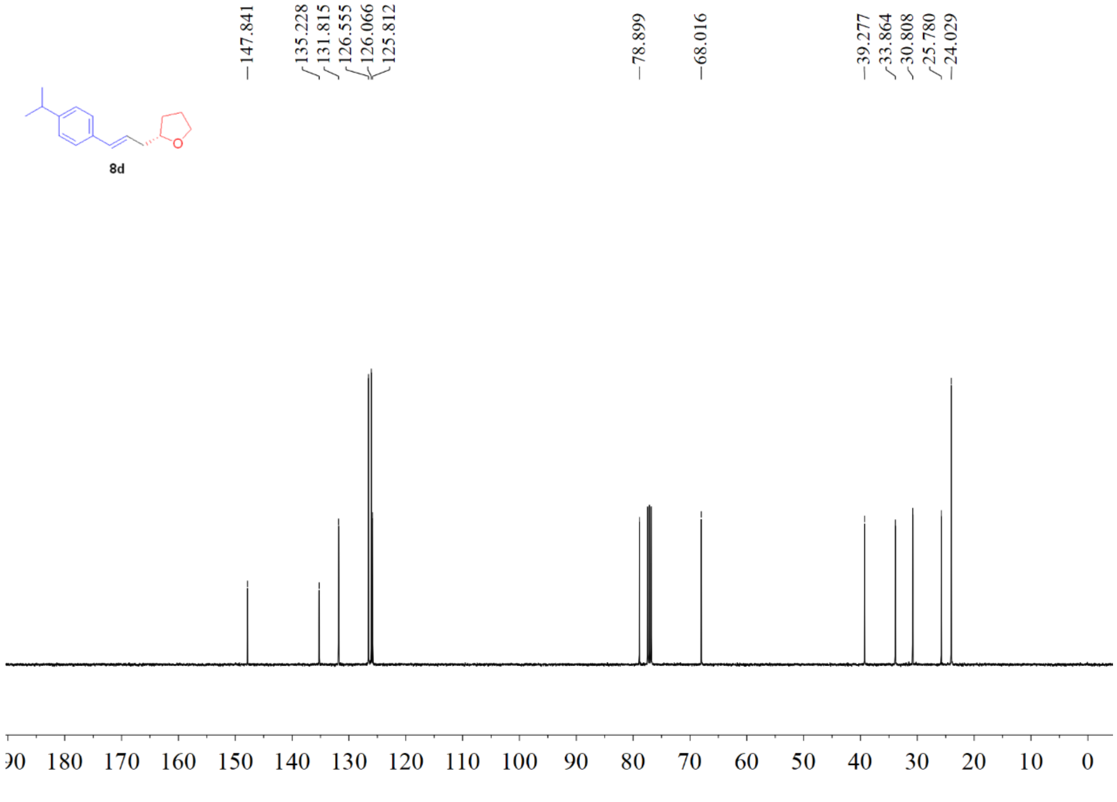

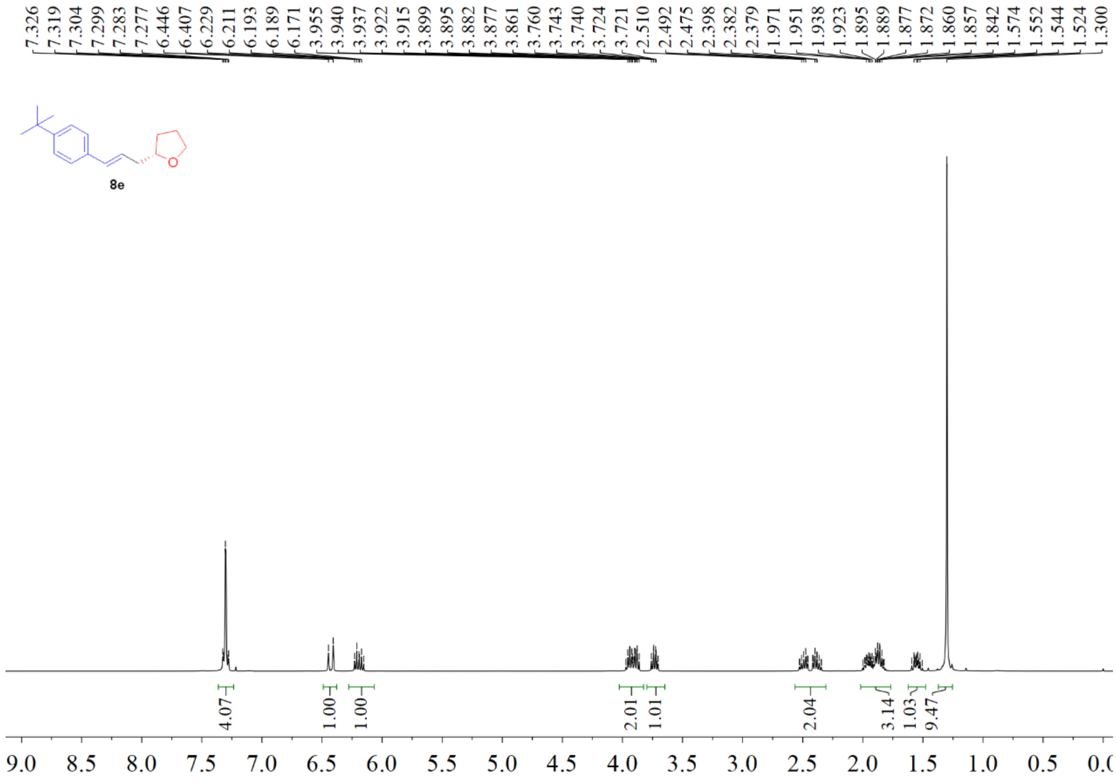

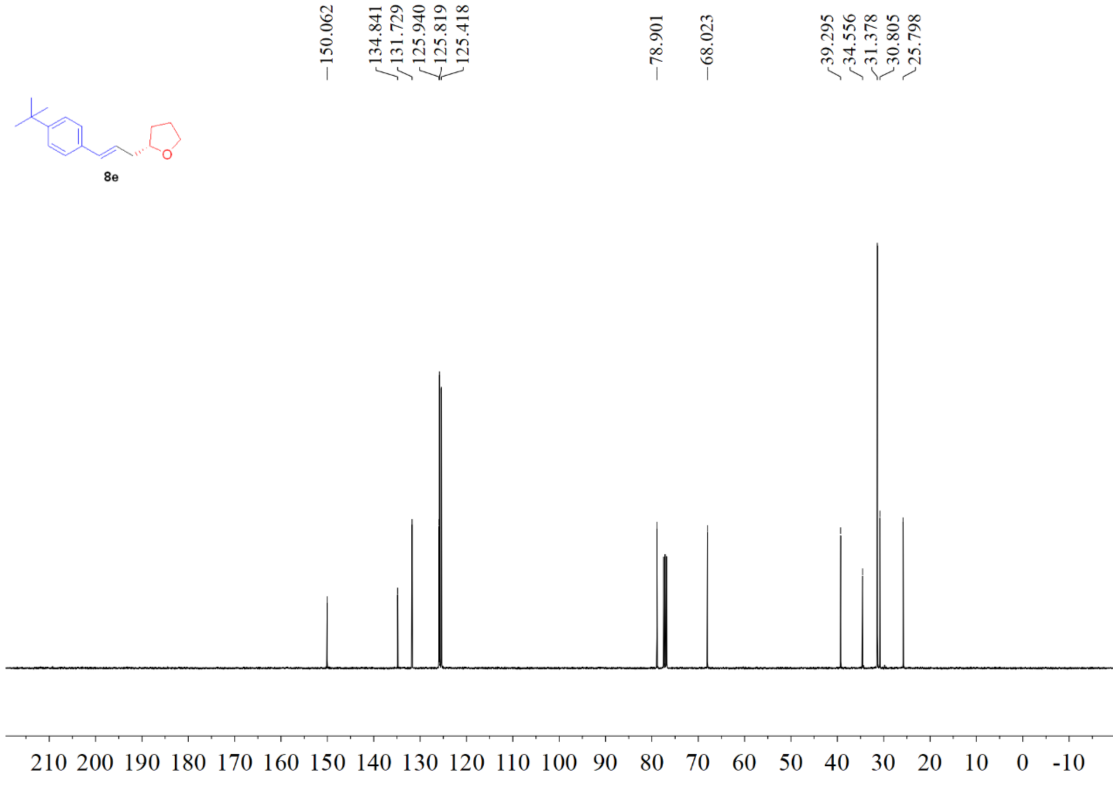

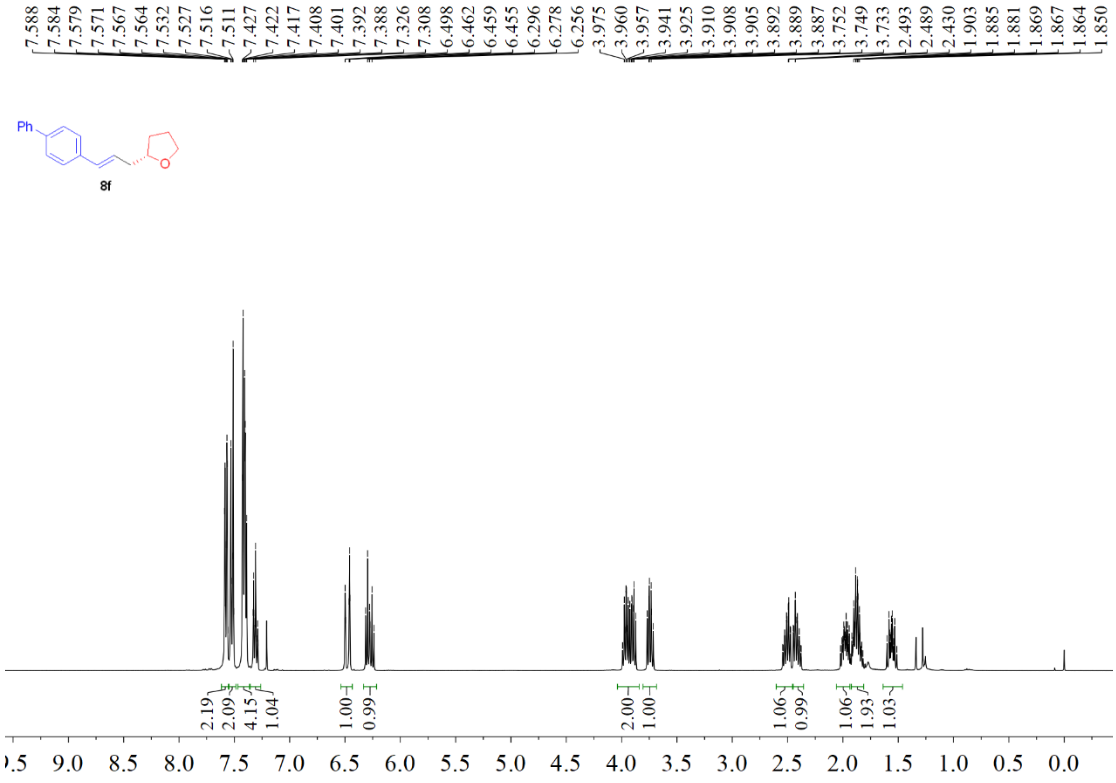
7.588
7.584
7.579
7.571
7.567
7.564
7.532
7.527
7.516
7.511
7.427
7.422
7.417
7.408
7.401
7.392
7.388
7.326
7.308
6.498
6.462
6.459
6.455
6.296
6.278
6.256
3.975
3.960
3.957
3.941
3.925
3.910
3.908
3.905
3.892
3.889
3.887
3.752
3.749
3.733
2.493
2.489
2.430
1.903
1.885
1.881
1.869
1.867
1.864
1.850
Ph
O
8f
2.19
2.09
4.15
1.04
1.00
0.99
2.00
1.00
1.06
0.99
1.06
1.93
1.03
.5 9.0 8.5 8.0 7.5 7.0 6.5 6.0 5.5 5.0 4.5 4.0 3.5 3.0 2.5 2.0 1.5 1.0 0.5 0.0

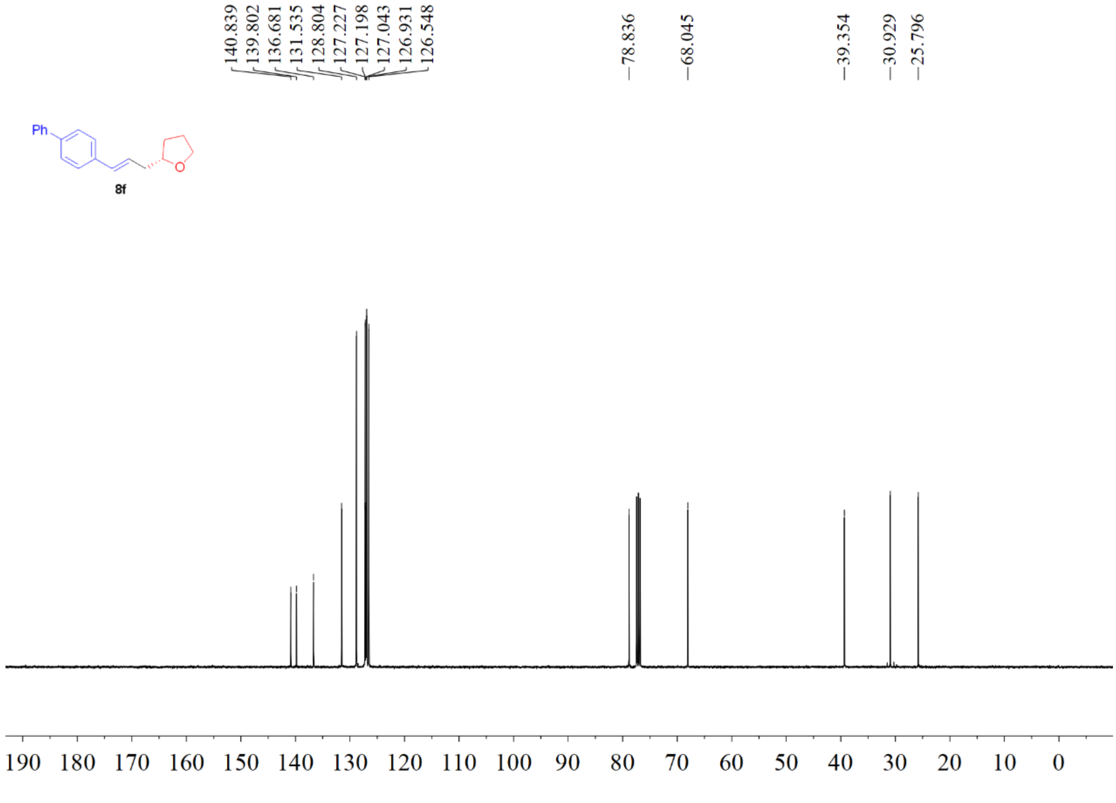
140.839
139.802
136.681
131.535
128.804
127.227
127.198
127.043
126.931
126.548
78.836
68.045
39.354
30.929
25.796
Ph
O
8f
190 180 170 160 150 140 130 120 110 100 90 80 70 60 50 40 30 20 10 0

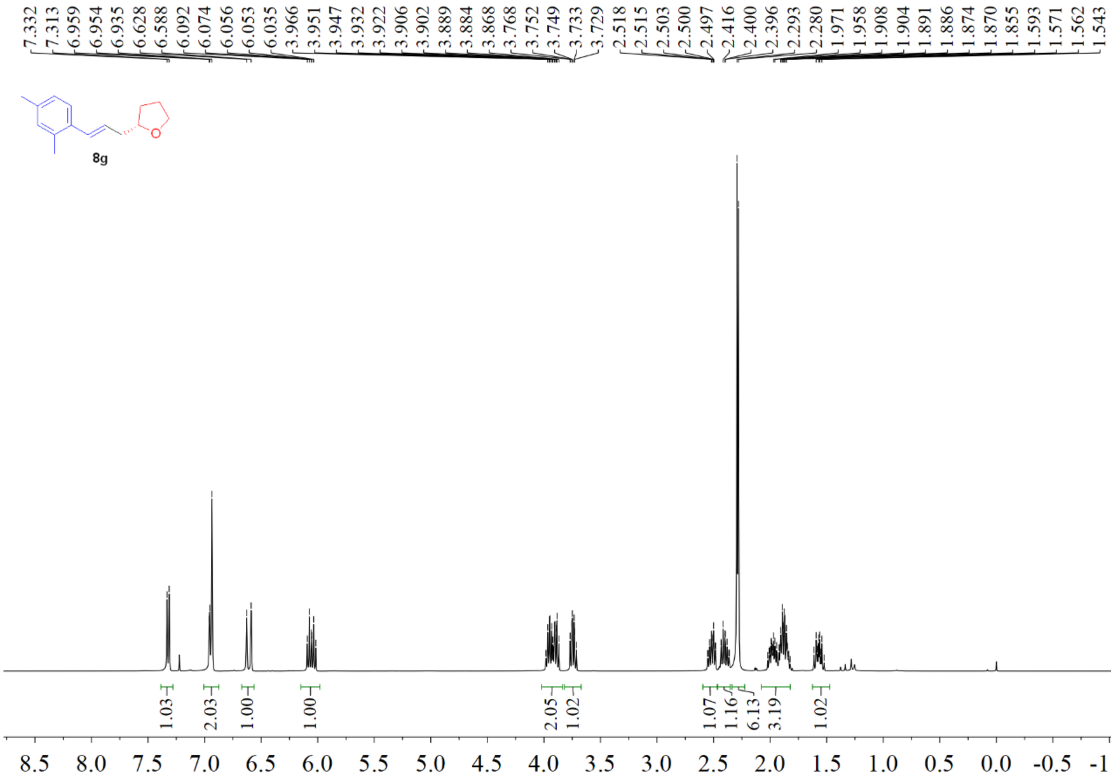
7.332
7.313
6.959
6.954
6.935
6.628
6.588
6.092
6.074
6.056
6.053
6.035
3.966
3.951
3.947
3.932
3.922
3.906
3.902
3.889
3.884
3.868
3.768
3.752
3.749
3.733
3.729
2.518
2.515
2.503
2.500
2.497
2.416
2.400
2.396
2.293
2.280
1.971
1.958
1.908
1.904
1.891
1.886
1.874
1.870
1.855
1.593
1.571
1.562
1.543
8g
1.03
2.03
1.00
1.00
2.05
1.02
1.07
1.16
6.13
3.19
1.02
8.5 8.0 7.5 7.0 6.5 6.0 5.5 5.0 4.5 4.0 3.5 3.0 2.5 2.0 1.5 1.0 0.5 0.0 -0.5 -1

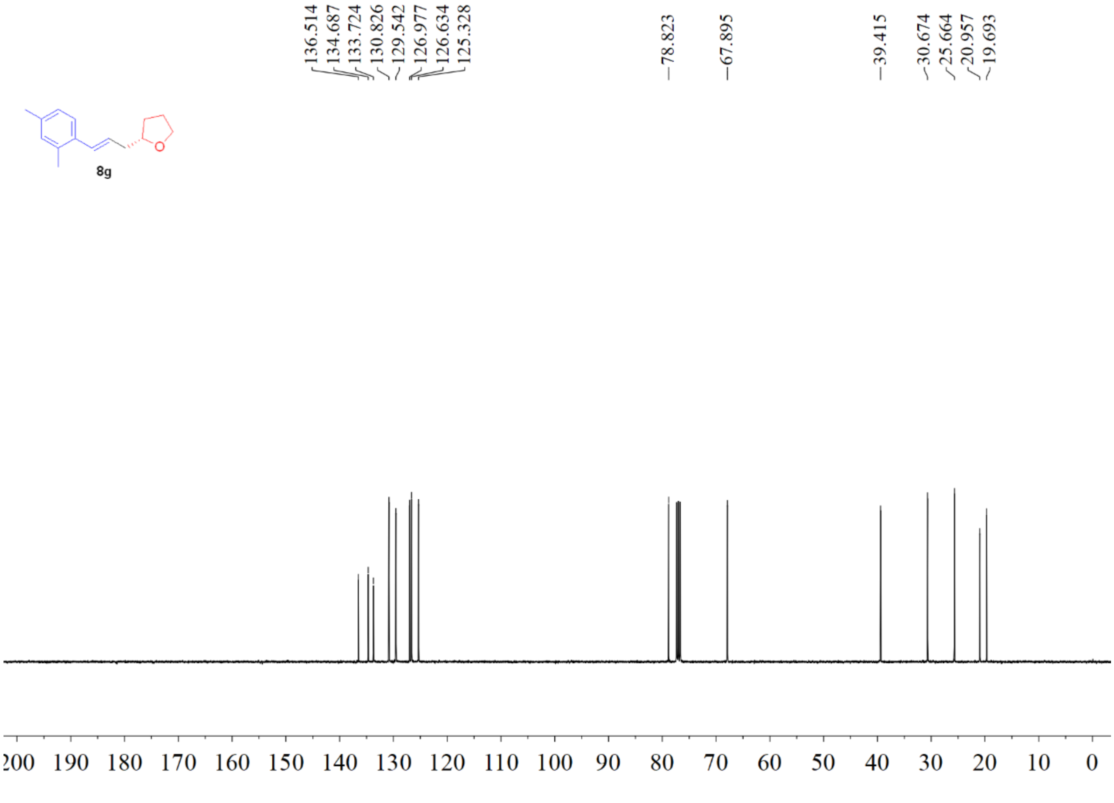
136.514
134.687
133.724
130.826
129.542
126.977
126.634
125.328
78.823
67.895
39.415
30.674
25.664
20.957
19.693
8g
200 190 180 170 160 150 140 130 120 110 100 90 80 70 60 50 40 30 20 10 0

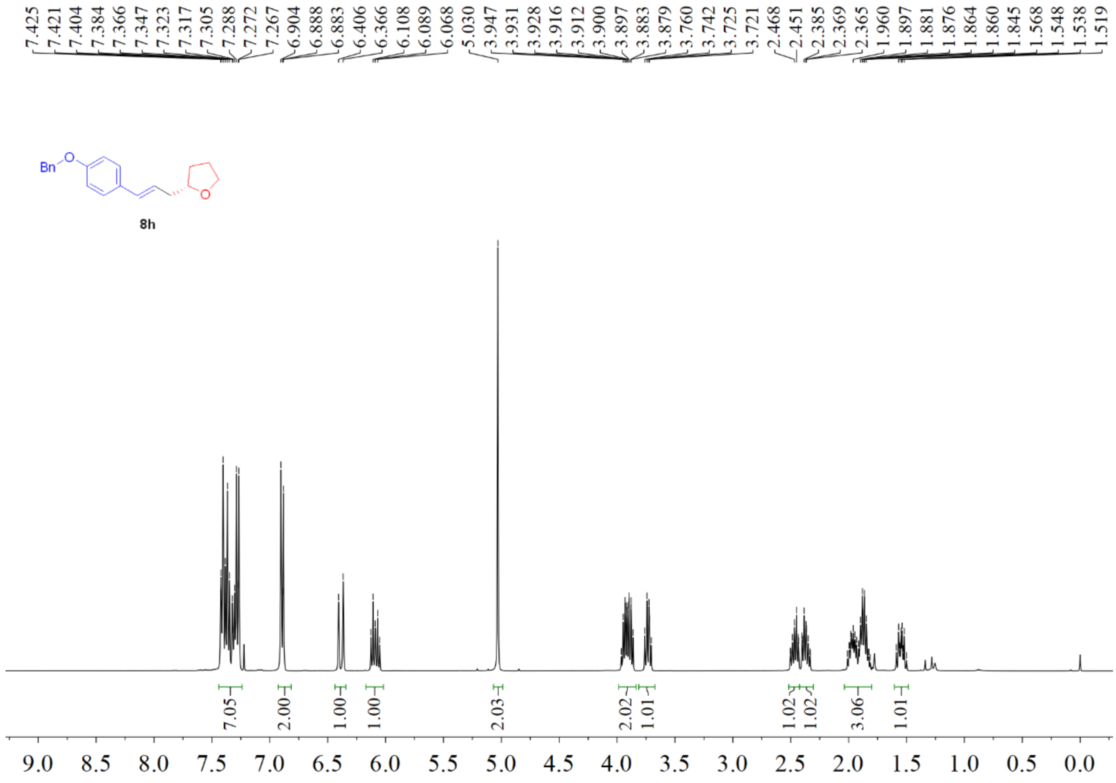
7.425
7.421
7.404
7.384
7.366
7.347
7.323
7.317
7.305
7.288
7.272
7.267
6.904
6.888
6.883
6.406
6.366
6.108
6.089
6.068
5.030
3.947
3.931
3.928
3.916
3.912
3.900
3.897
3.883
3.879
3.760
3.742
3.725
3.721
2.468
2.451
2.385
2.369
2.365
1.960
1.897
1.881
1.876
1.864
1.860
1.845
1.568
1.548
1.538
1.519
Bn
O
O
8h
7.05
2.00
1.00
1.00
2.03
2.02
1.01
1.02
1.02
3.06
1.01
9.0
8.5
8.0
7.5
7.0
6.5
6.0
5.5
5.0
4.5
4.0
3.5
3.0
2.5
2.0
1.5
1.0
0.5
0.0

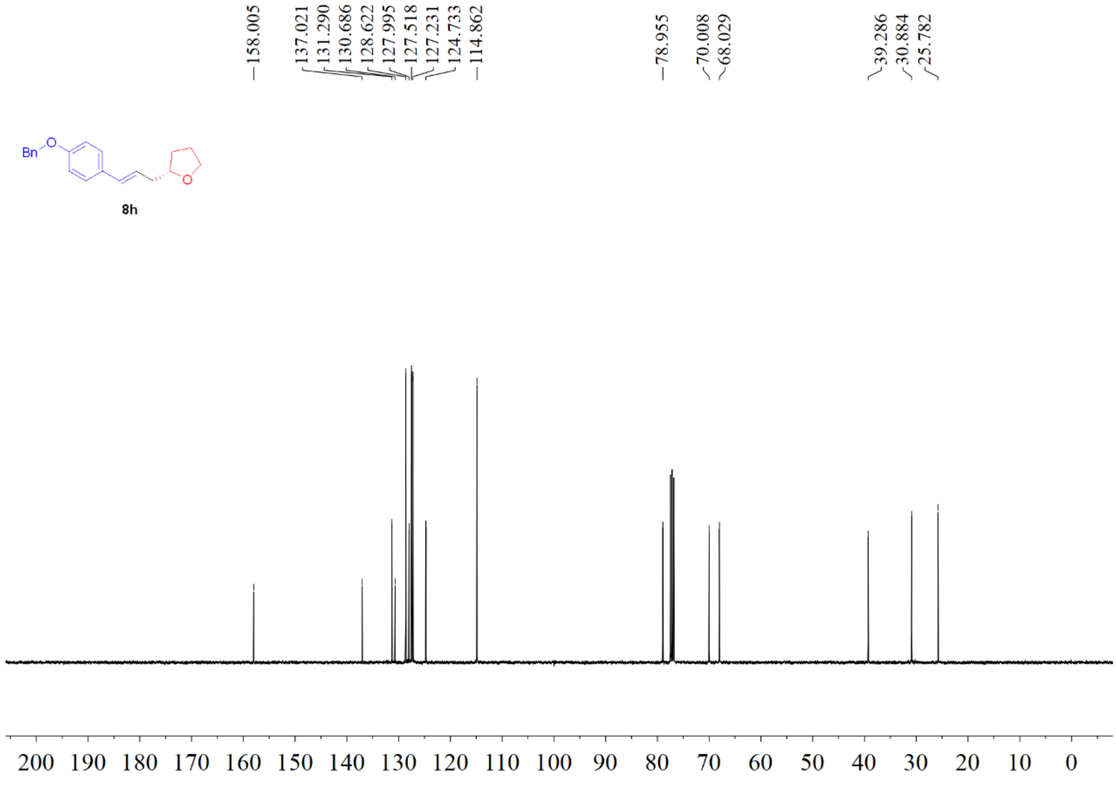
158.005
137.021
131.290
130.686
128.622
127.995
127.518
127.231
124.733
114.862
78.955
70.008
68.029
39.286
30.884
25.782
Bn
O
O
8h
200
190
180
170
160
150
140
130
120
110
100
90
80
70
60
50
40
30
20
10
0

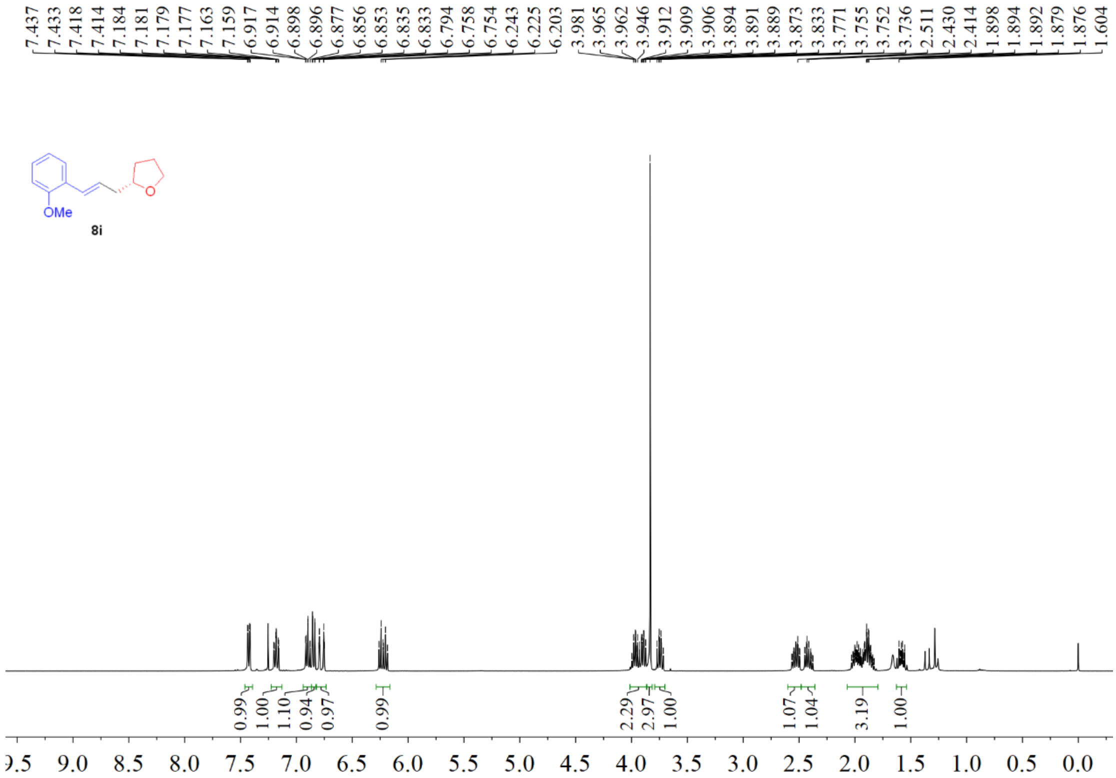
7.437
7.433
7.418
7.414
7.184
7.181
7.179
7.177
7.163
7.159
6.917
6.914
6.898
6.896
6.877
6.856
6.853
6.835
6.833
6.794
6.758
6.754
6.243
6.225
6.203
3.981
3.965
3.962
3.946
3.912
3.909
3.906
3.894
3.891
3.889
3.873
3.833
3.771
3.755
3.752
3.736
2.511
2.430
2.414
1.898
1.894
1.892
1.879
1.876
1.604
OMe
8i
0.99
1.00
1.10
0.94
0.97
0.99
2.29
2.97
1.00
1.07
1.04
3.19
1.00
9.5 9.0 8.5 8.0 7.5 7.0 6.5 6.0 5.5 5.0 4.5 4.0 3.5 3.0 2.5 2.0 1.5 1.0 0.5 0.0

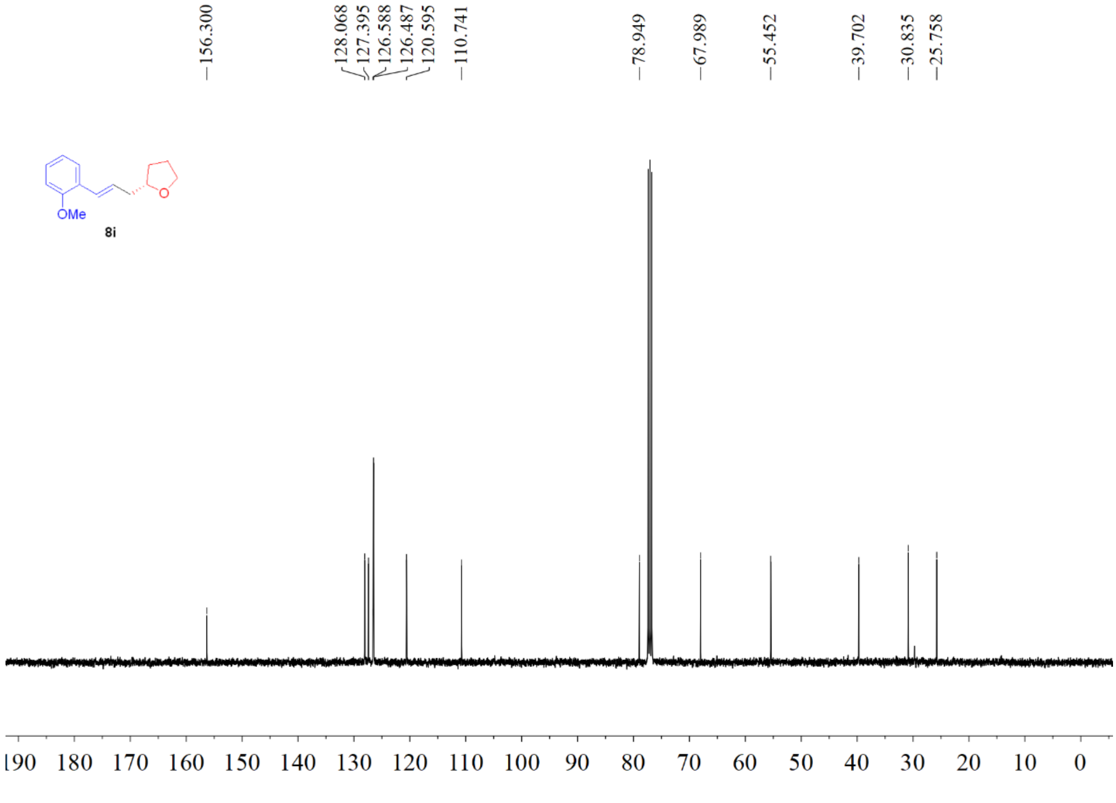
156.300
128.068
127.395
126.588
126.487
120.595
110.741
78.949
67.989
55.452
39.702
30.835
25.758
OMe
8i
190 180 170 160 150 140 130 120 110 100 90 80 70 60 50 40 30 20 10 0

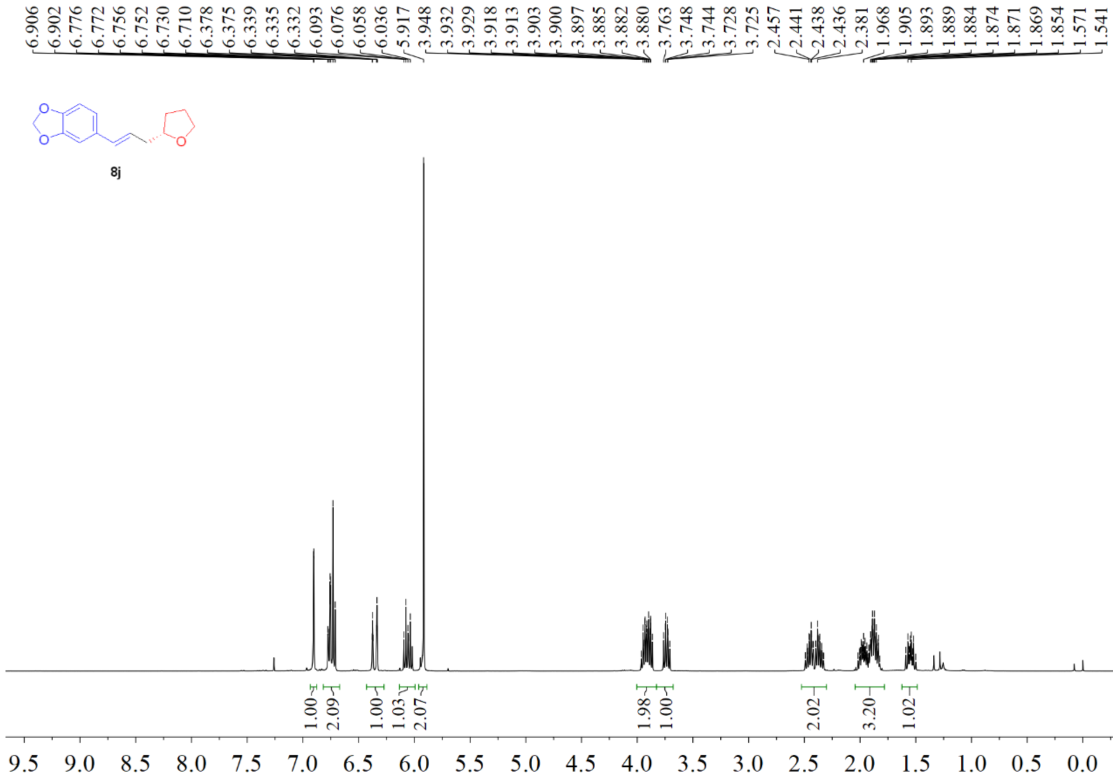
6.906
6.902
6.776
6.772
6.756
6.752
6.730
6.710
6.378
6.375
6.339
6.335
6.332
6.093
6.076
6.058
6.036
5.917
3.948
3.932
3.929
3.918
3.913
3.903
3.900
3.897
3.885
3.882
3.880
3.763
3.748
3.744
3.728
3.725
2.457
2.441
2.438
2.436
2.381
1.968
1.905
1.893
1.889
1.884
1.874
1.871
1.869
1.854
1.571
1.541
8j
1.00
2.09
1.00
1.03
2.07
1.98
1.00
2.02
3.20
1.02
9.5 9.0 8.5 8.0 7.5 7.0 6.5 6.0 5.5 5.0 4.5 4.0 3.5 3.0 2.5 2.0 1.5 1.0 0.5 0.0

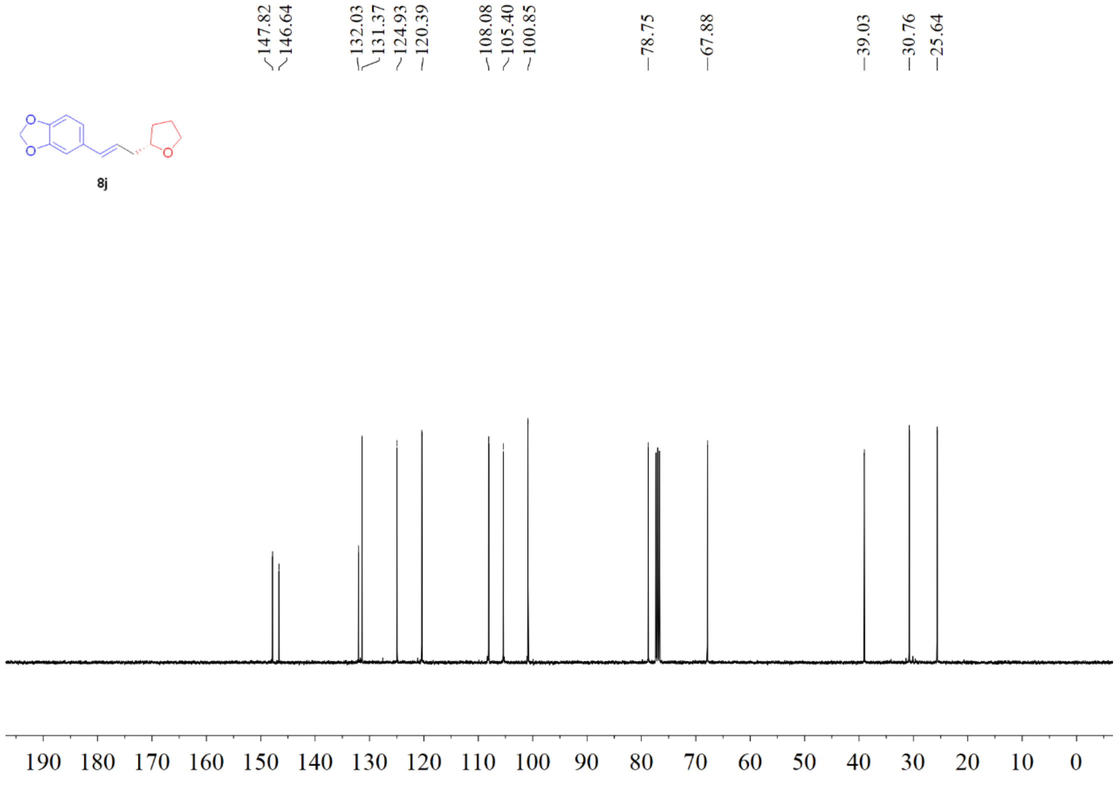
147.82
146.64
132.03
131.37
124.93
120.39
108.08
105.40
100.85
78.75
67.88
39.03
30.76
25.64
8j
190 180 170 160 150 140 130 120 110 100 90 80 70 60 50 40 30 20 10 0

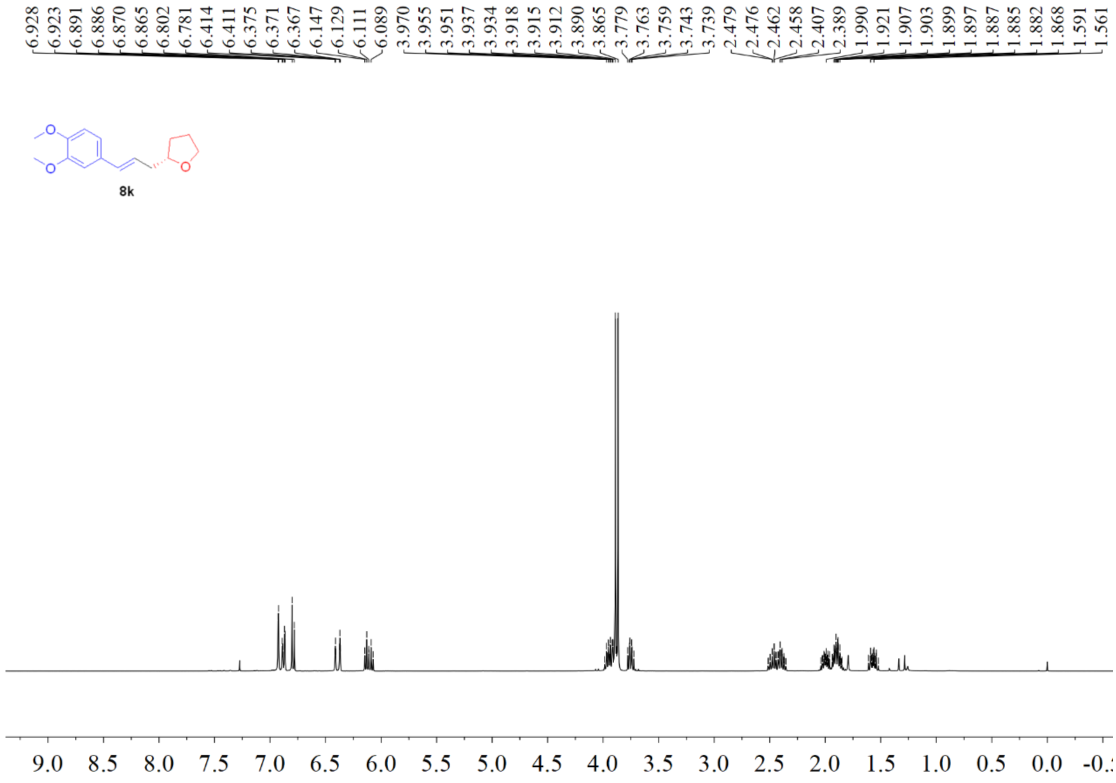
6.928
6.923
6.891
6.886
6.870
6.865
6.802
6.781
6.414
6.411
6.375
6.371
6.367
6.147
6.129
6.111
6.089
3.970
3.955
3.951
3.937
3.934
3.918
3.915
3.912
3.890
3.865
3.779
3.763
3.759
3.743
3.739
2.479
2.476
2.462
2.458
2.407
2.389
1.990
1.921
1.907
1.903
1.899
1.897
1.887
1.885
1.882
1.868
1.591
1.561
8k
9.0 8.5 8.0 7.5 7.0 6.5 6.0 5.5 5.0 4.5 4.0 3.5 3.0 2.5 2.0 1.5 1.0 0.5 0.0

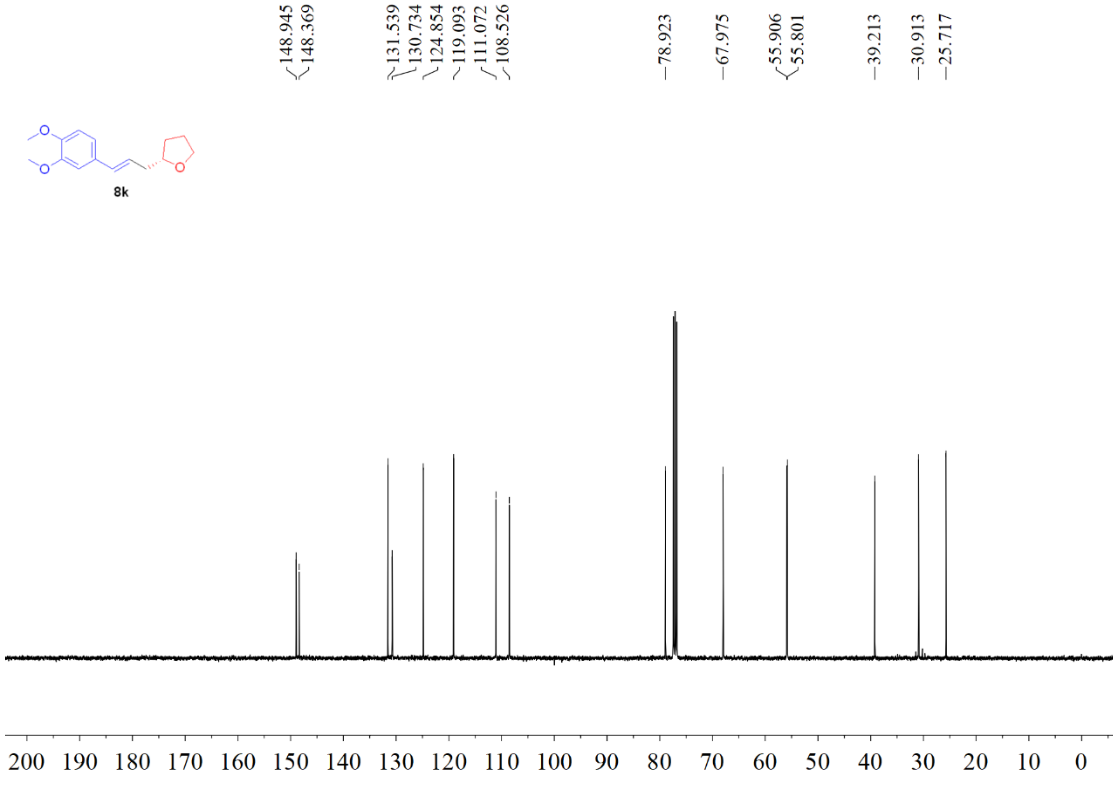
148.945
148.369
131.539
130.734
124.854
119.093
111.072
108.526
78.923
67.975
55.906
55.801
39.213
30.913
25.717
8k
200 190 180 170 160 150 140 130 120 110 100 90 80 70 60 50 40 30 20 10 0

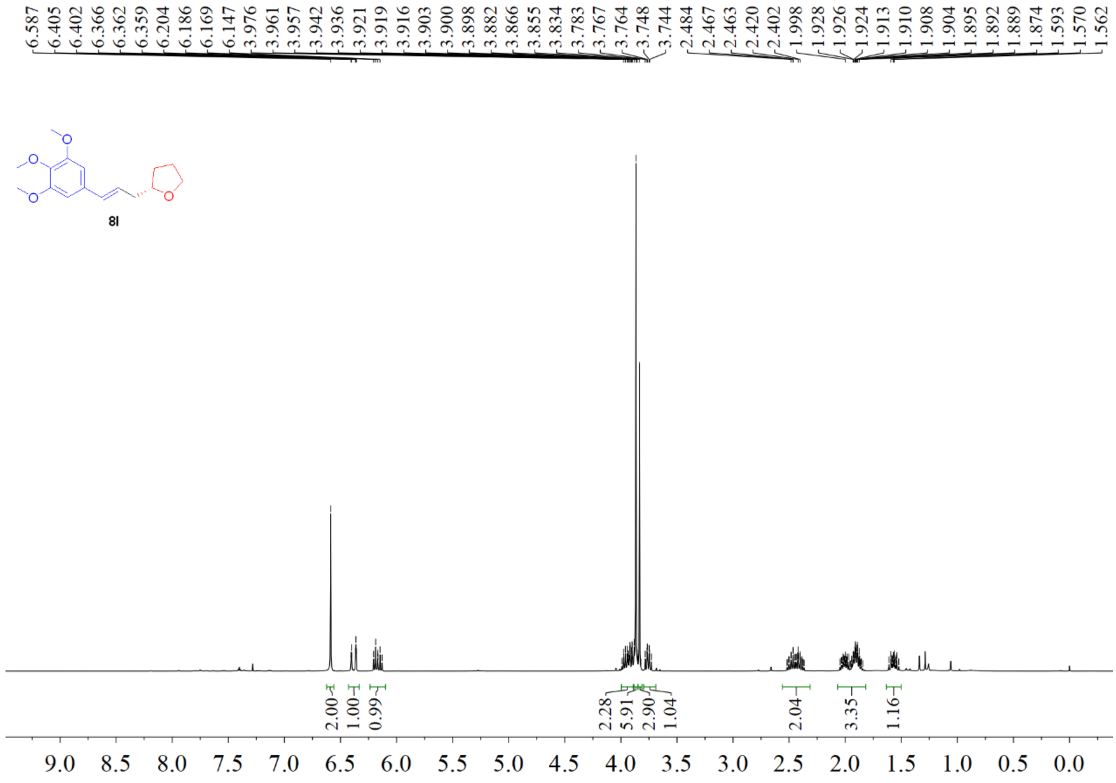
6.587
6.405
6.402
6.366
6.362
6.359
6.204
6.186
6.169
6.147
3.976
3.961
3.957
3.942
3.936
3.921
3.919
3.916
3.903
3.900
3.898
3.882
3.866
3.855
3.834
3.783
3.767
3.764
3.748
3.744
2.484
2.467
2.463
2.420
2.402
1.998
1.928
1.926
1.924
1.913
1.910
1.908
1.904
1.895
1.892
1.889
1.874
1.593
1.570
1.562
8l
2.00
1.00
0.99
2.28
5.91
2.90
1.04
2.04
3.35
1.16
9.0
8.5
8.0
7.5
7.0
6.5
6.0
5.5
5.0
4.5
4.0
3.5
3.0
2.5
2.0
1.5
1.0
0.5
0.0

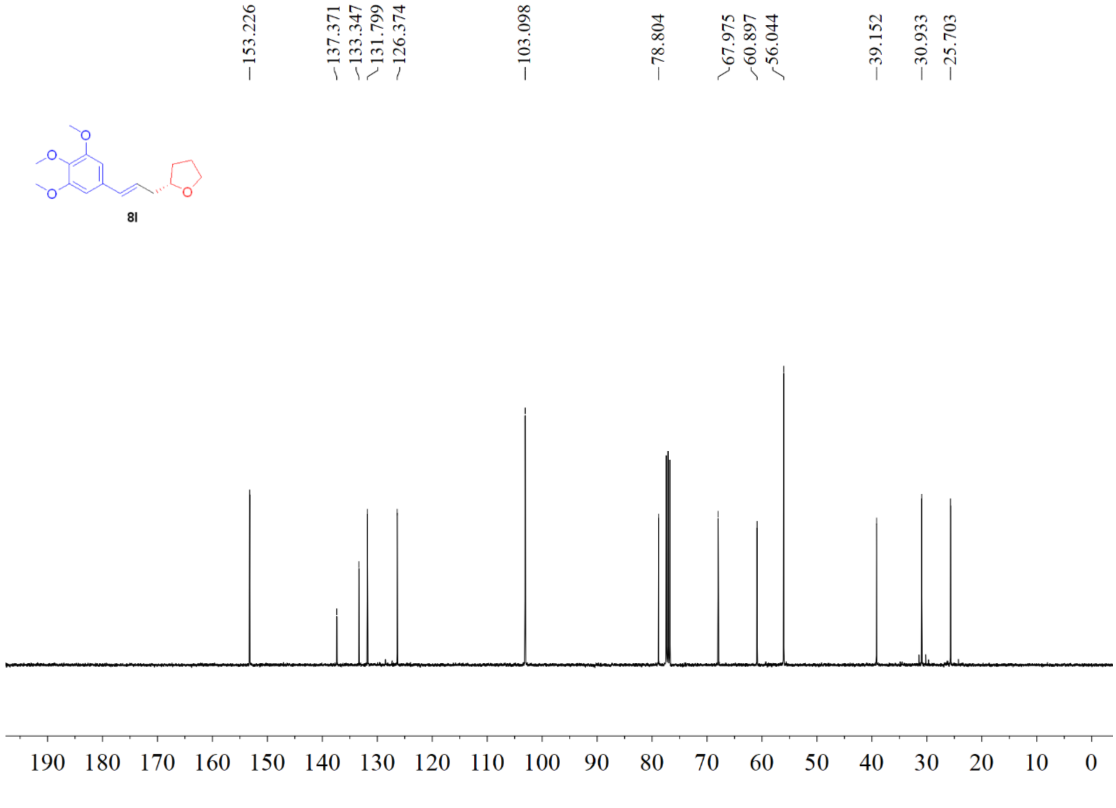
153.226
137.371
133.347
131.799
126.374
103.098
78.804
67.975
60.897
56.044
39.152
30.933
25.703
8l
190
180
170
160
150
140
130
120
110
100
90
80
70
60
50
40
30
20
10
0

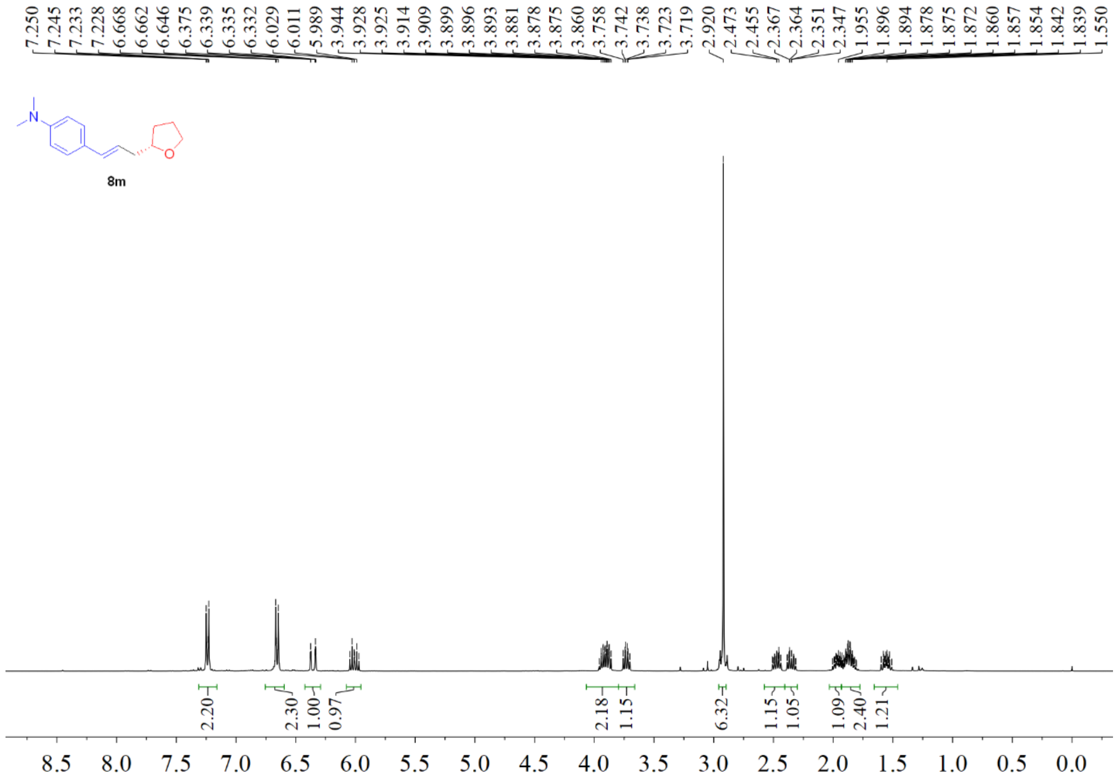

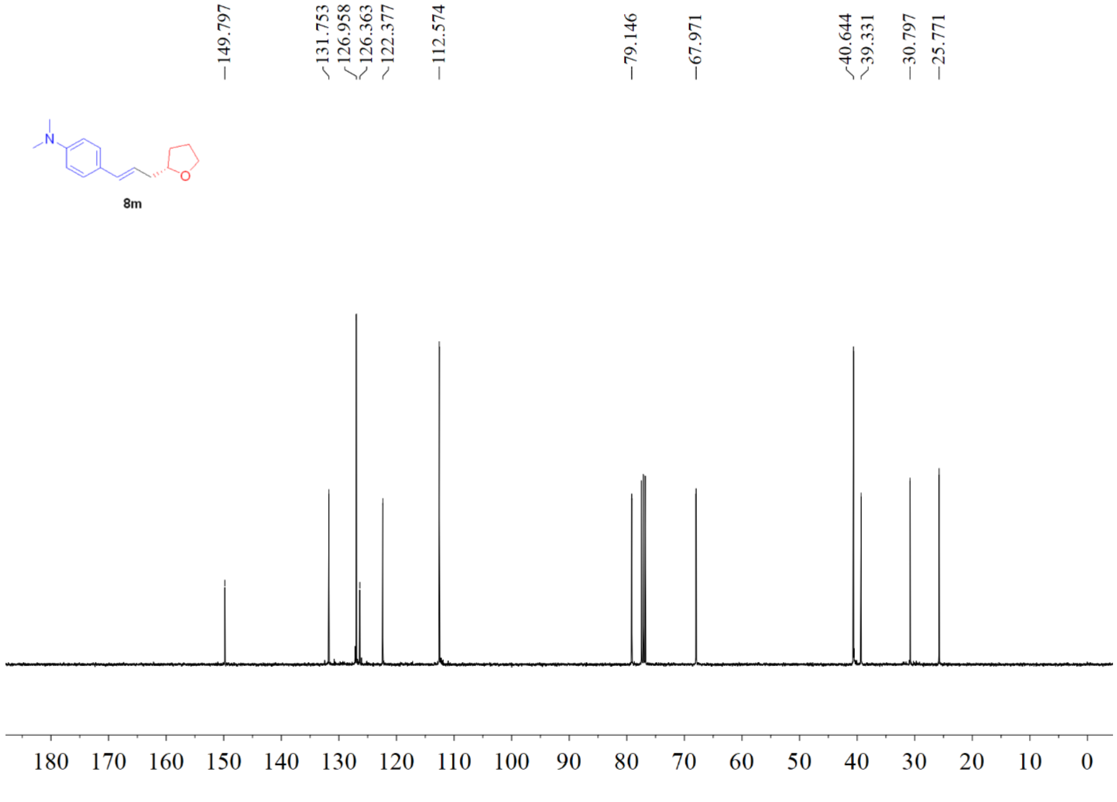

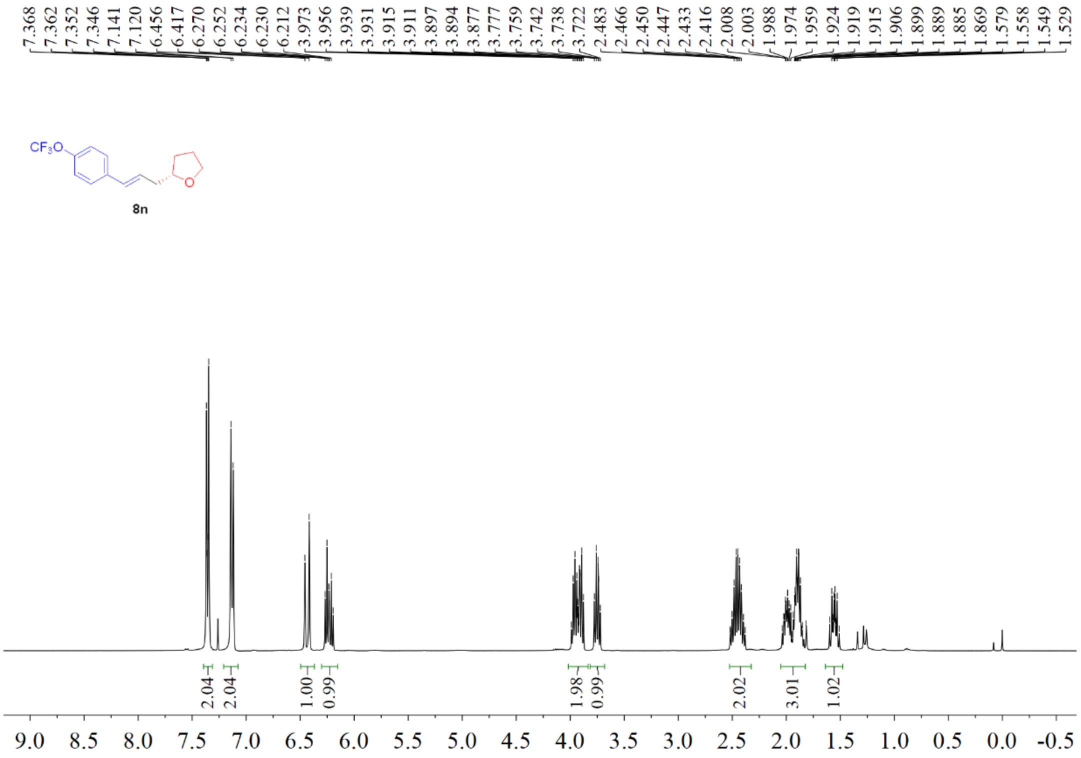
7.368 7.362 7.352 7.346 7.141 7.120 6.456 6.417 6.270 6.252 6.234 6.230 6.212 3.973 3.956 3.939 3.931 3.915 3.911 3.897 3.894 3.877 3.777 3.759 3.742 3.738 3.722 2.483 2.466 2.450 2.447 2.433 2.416 2.008 2.003 1.988 1.974 1.959 1.924 1.919 1.915 1.906 1.899 1.889 1.885 1.869 1.579 1.558 1.549 1.529
8n
2.04 2.04 1.00 0.99 1.98 0.99 2.02 3.01 1.02
9.0 8.5 8.0 7.5 7.0 6.5 6.0 5.5 5.0 4.5 4.0 3.5 3.0 2.5 2.0 1.5 1.0 0.5 0.0 -0.5

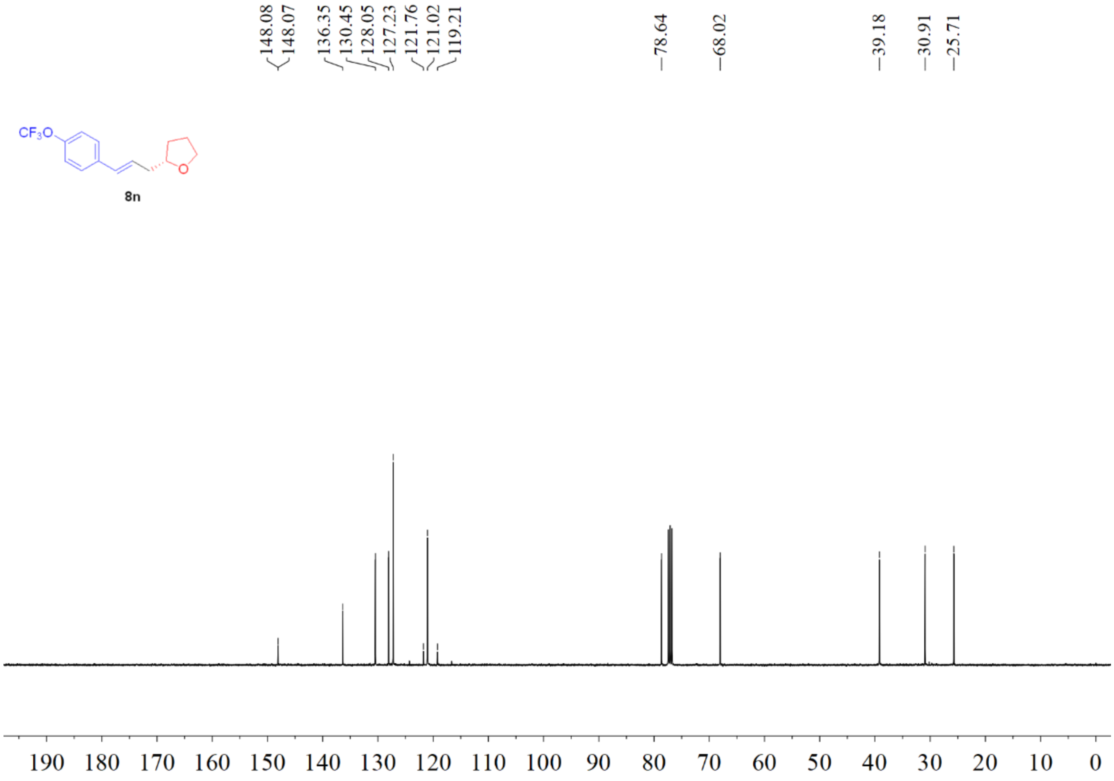
148.08 148.07 136.35 130.45 128.05 127.23 121.76 121.02 119.21 78.64 68.02 39.18 30.91 25.71
8n
190 180 170 160 150 140 130 120 110 100 90 80 70 60 50 40 30 20 10 0

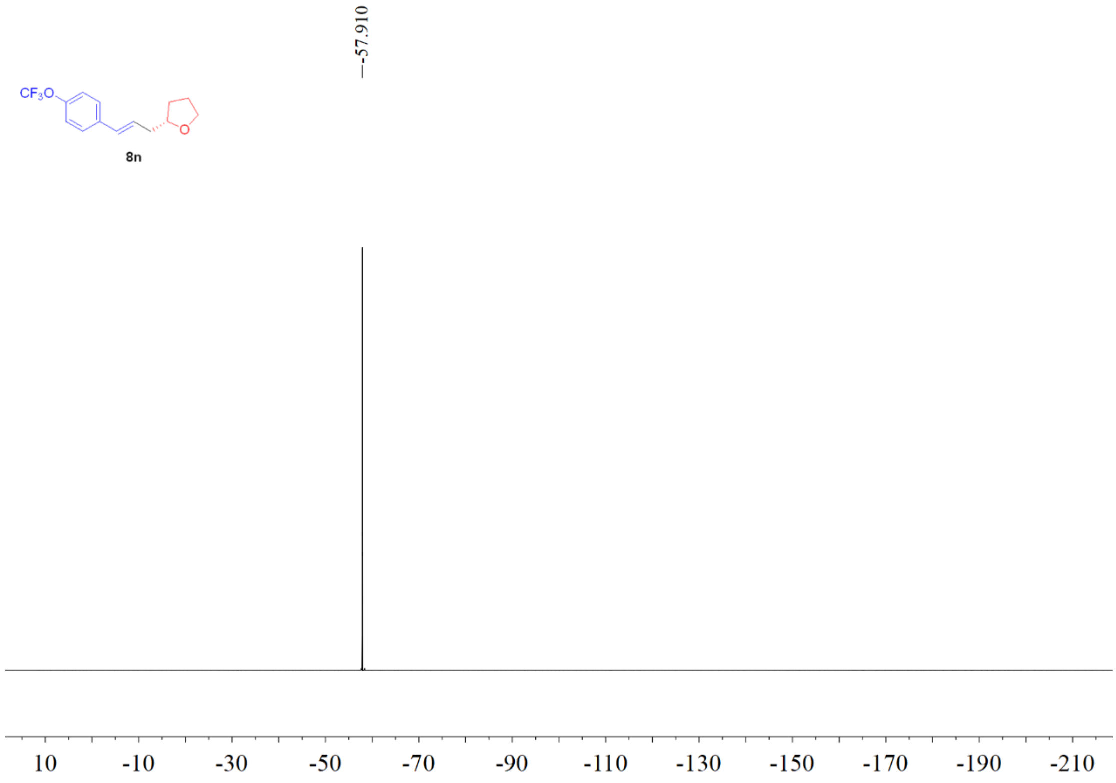

-57.910
CF3O
8n
10 -10 -30 -50 -70 -90 -110 -130 -150 -170 -190 -210

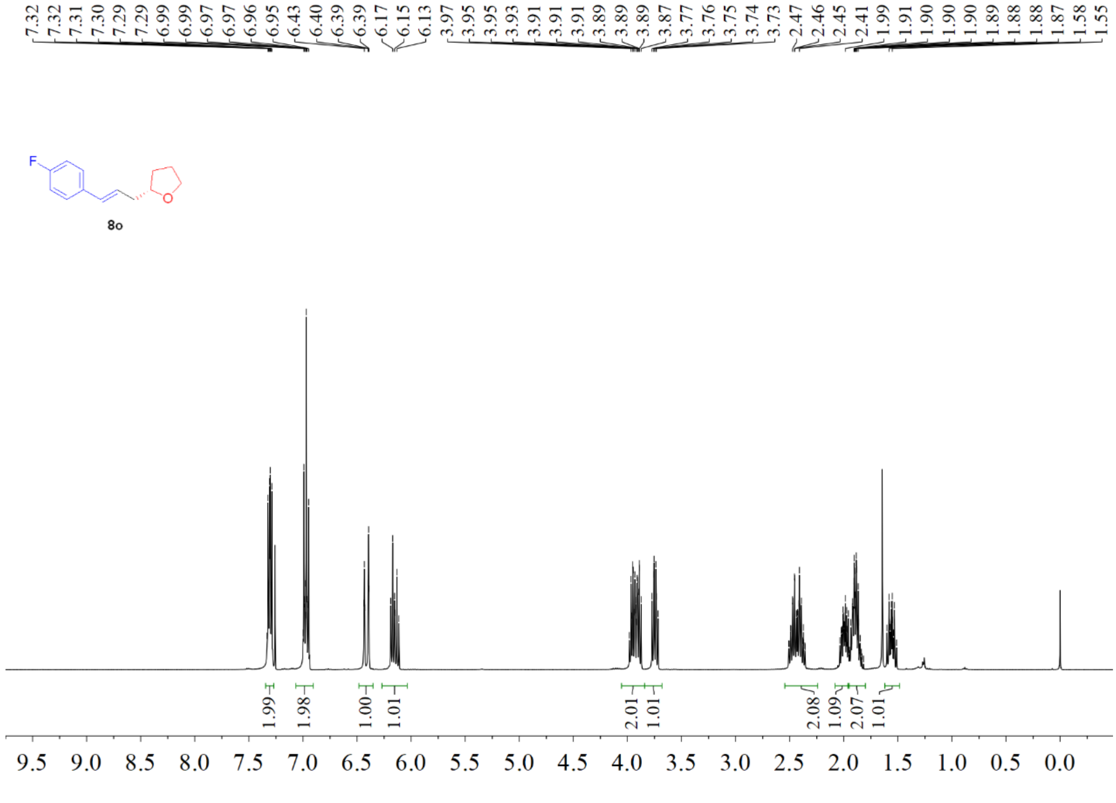

7.32 7.32 7.31 7.30 7.29 7.29 6.99 6.99 6.97 6.97 6.96 6.95 6.43 6.40 6.39 6.39 6.17 6.15 6.13 3.97 3.95 3.95 3.93 3.91 3.91 3.91 3.89 3.89 3.89 3.87 3.77 3.76 3.75 3.74 3.73 2.47 2.46 2.45 2.41 1.99 1.91 1.90 1.90 1.90 1.89 1.88 1.88 1.87 1.58 1.55
F
8o
1.99 1.98 1.00 1.01 2.01 1.01 2.08 1.09 2.07 1.01
9.5 9.0 8.5 8.0 7.5 7.0 6.5 6.0 5.5 5.0 4.5 4.0 3.5 3.0 2.5 2.0 1.5 1.0 0.5 0.0

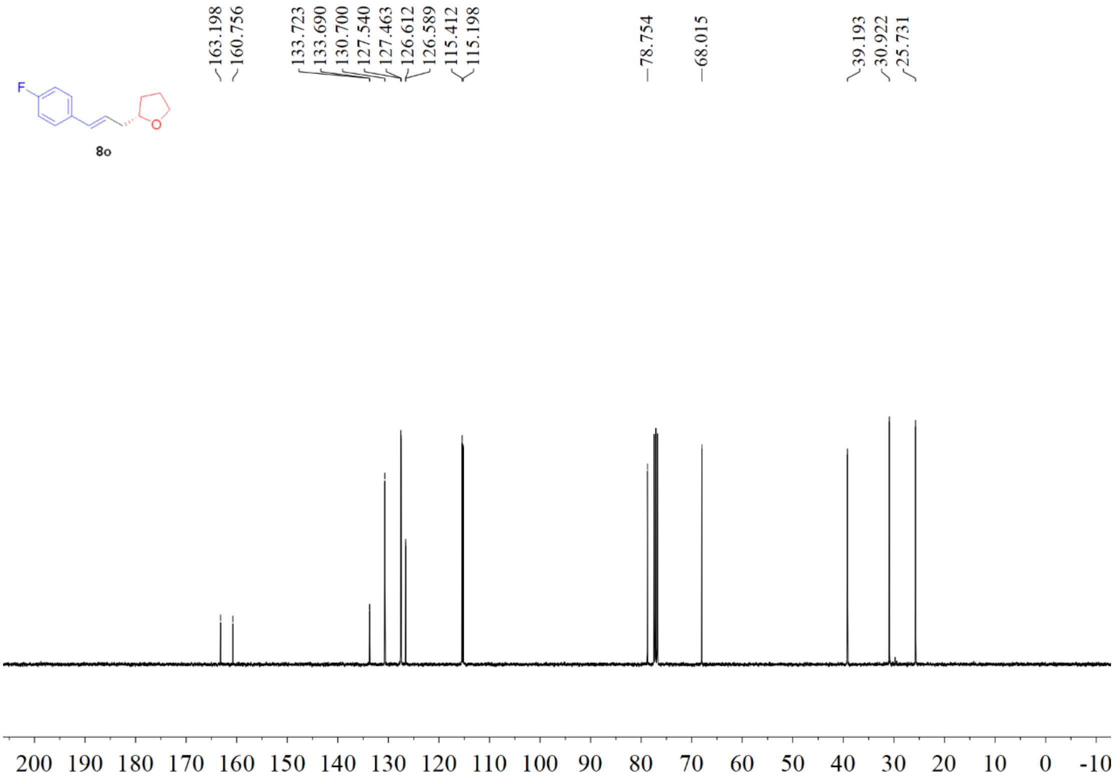
163.198
160.756
133.723
133.690
130.700
127.540
127.463
126.612
126.589
115.412
115.198
78.754
68.015
39.193
30.922
25.731
F
O
8o
200 190 180 170 160 150 140 130 120 110 100 90 80 70 60 50 40 30 20 10 0 -10

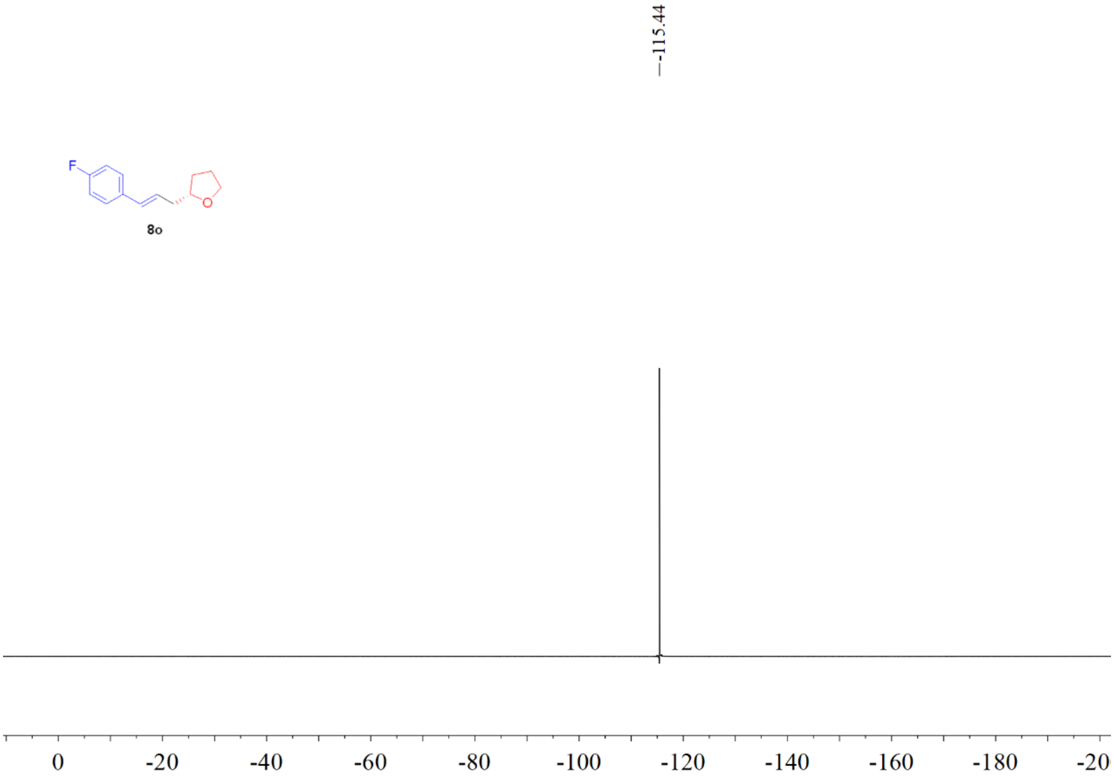
-115.44
F
O
8o
0 -20 -40 -60 -80 -100 -120 -140 -160 -180 -20

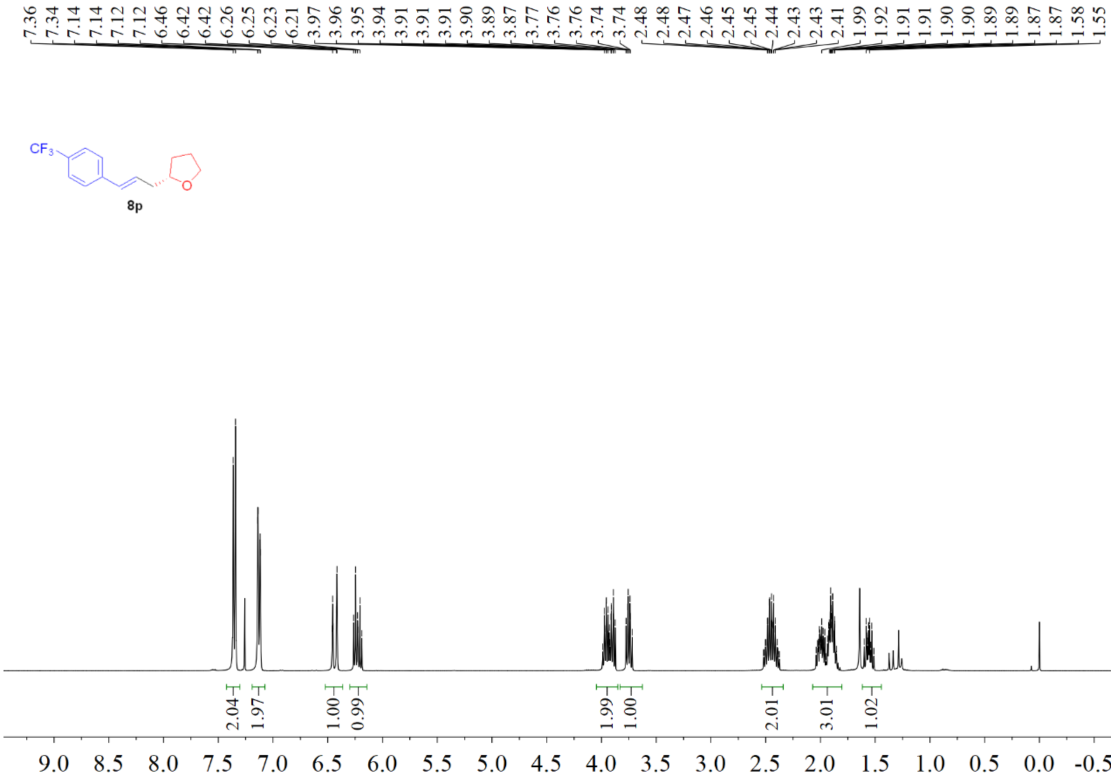

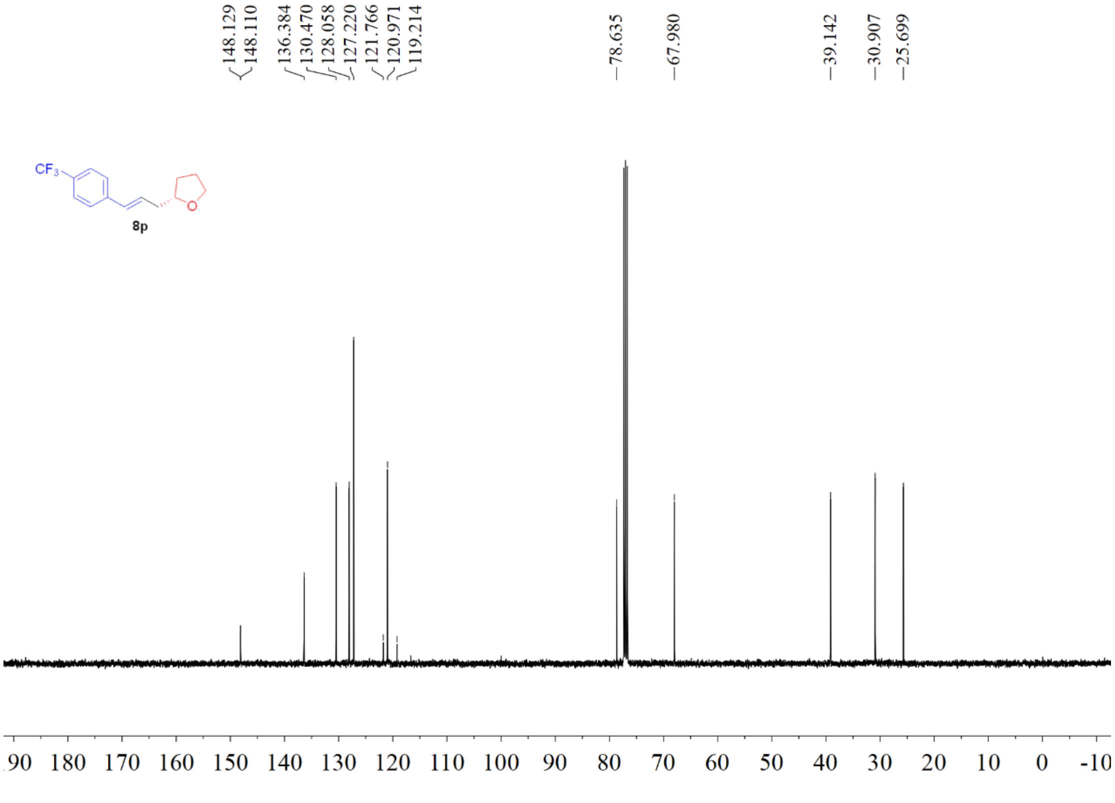

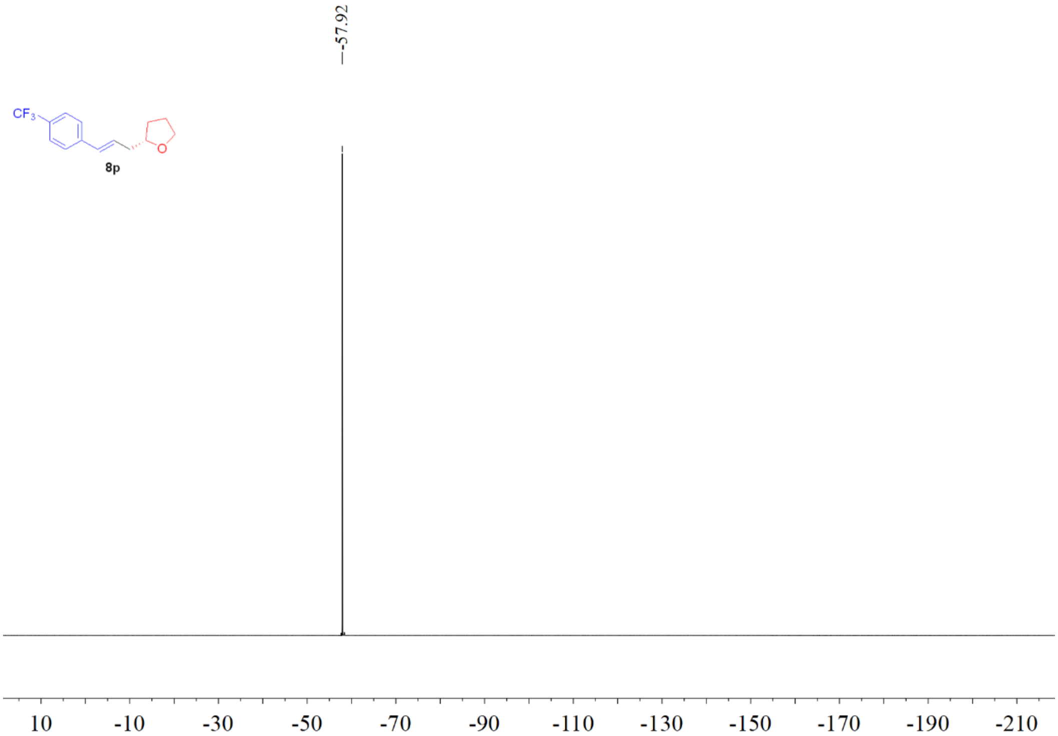
-57.92
CF3
O
8p
10 -10 -30 -50 -70 -90 -110 -130 -150 -170 -190 -210

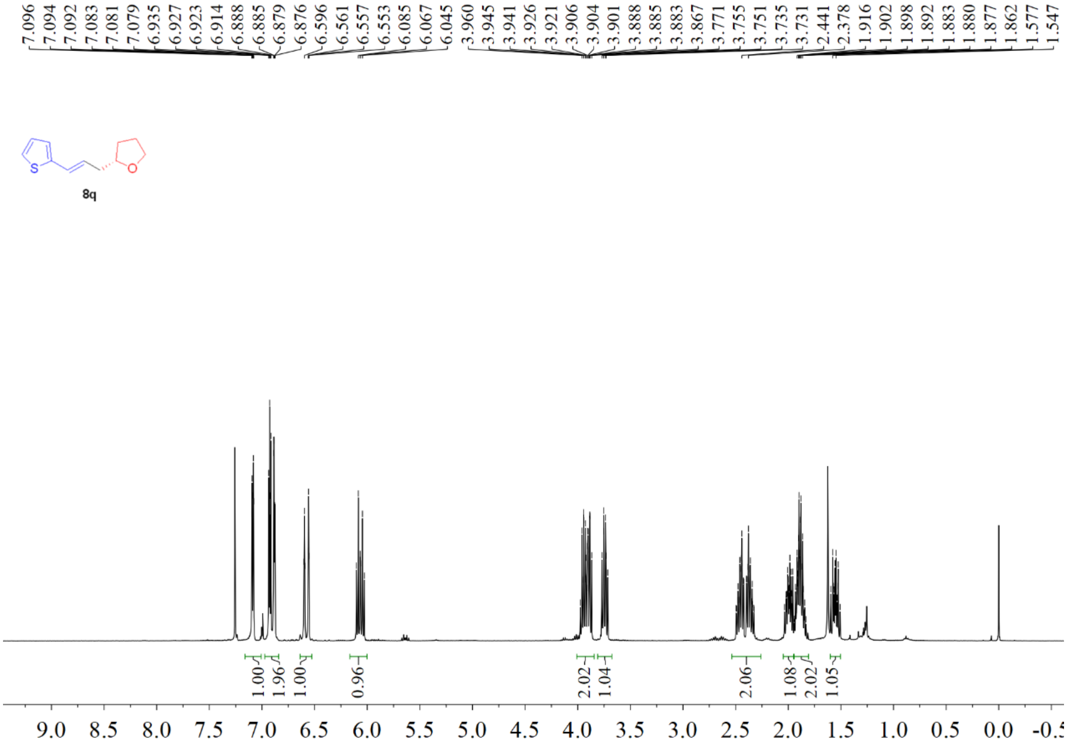
7.096 7.094 7.092 7.083 7.081 7.079 6.935 6.927 6.923 6.914 6.888 6.885 6.879 6.876 6.596 6.561 6.557 6.553 6.085 6.067 6.045 3.960 3.945 3.941 3.926 3.921 3.906 3.904 3.901 3.888 3.885 3.883 3.867 3.771 3.755 3.751 3.735 3.731 2.441 2.378 1.916 1.902 1.898 1.892 1.883 1.880 1.877 1.862 1.577 1.547
S
O
8q
1.00 1.96 1.00 0.96 2.02 1.04 2.06 1.08 2.02 1.05
9.0 8.5 8.0 7.5 7.0 6.5 6.0 5.5 5.0 4.5 4.0 3.5 3.0 2.5 2.0 1.5 1.0 0.5 0.0

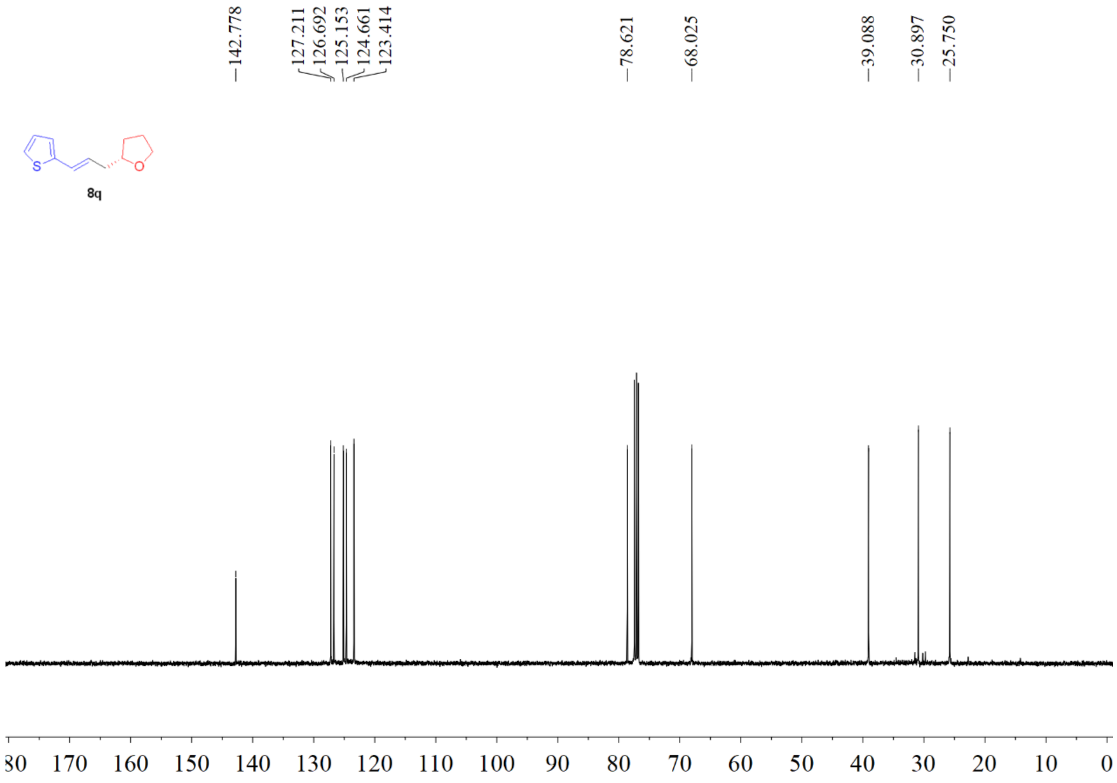
142.778
127.211
126.692
125.153
124.661
123.414
78.621
68.025
39.088
30.897
25.750
8q
80 170 160 150 140 130 120 110 100 90 80 70 60 50 40 30 20 10 0

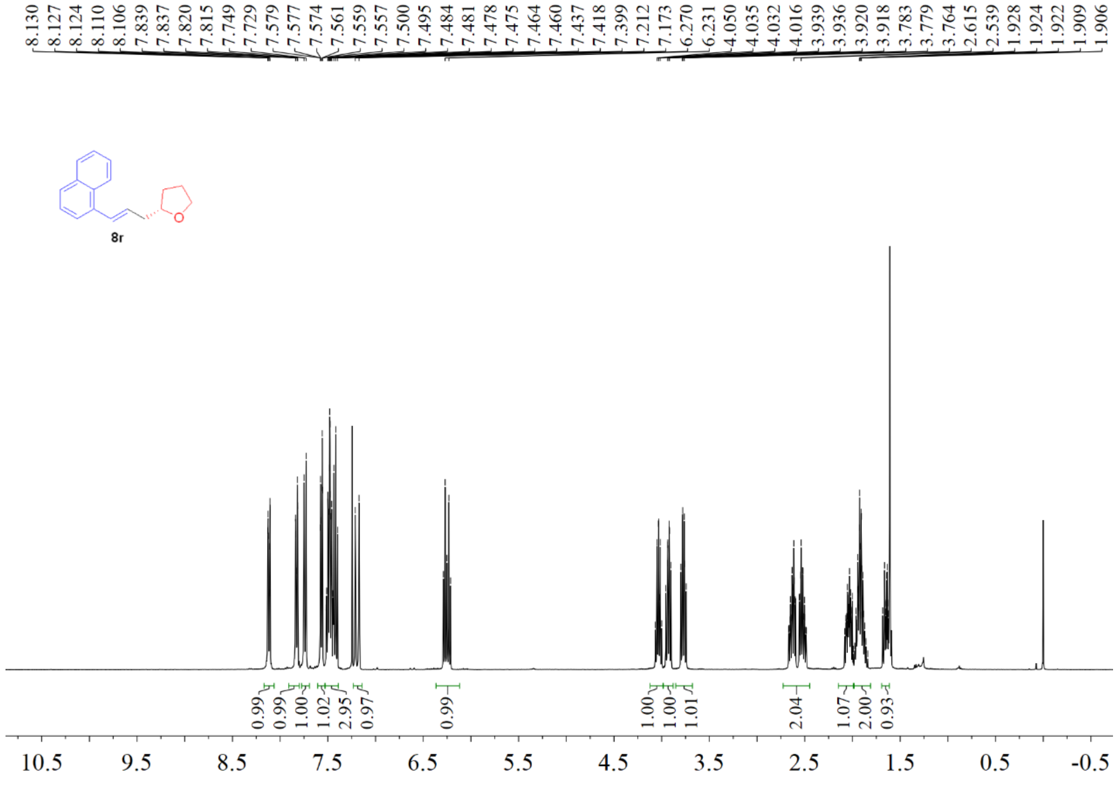
8.130
8.127
8.124
8.110
8.106
7.839
7.837
7.820
7.815
7.749
7.729
7.579
7.577
7.574
7.561
7.559
7.557
7.500
7.495
7.484
7.481
7.478
7.475
7.464
7.460
7.437
7.418
7.399
7.212
7.173
6.270
6.231
4.050
4.035
4.032
4.016
3.939
3.936
3.920
3.918
3.783
3.779
3.764
2.615
2.539
1.928
1.924
1.922
1.909
1.906
8r
0.99
0.99
1.00
1.02
2.95
0.97
0.99
1.00
1.00
1.01
2.04
1.07
2.00
0.93
10.5 9.5 8.5 7.5 6.5 5.5 4.5 3.5 2.5 1.5 0.5 -0.5

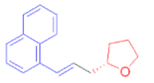

8r

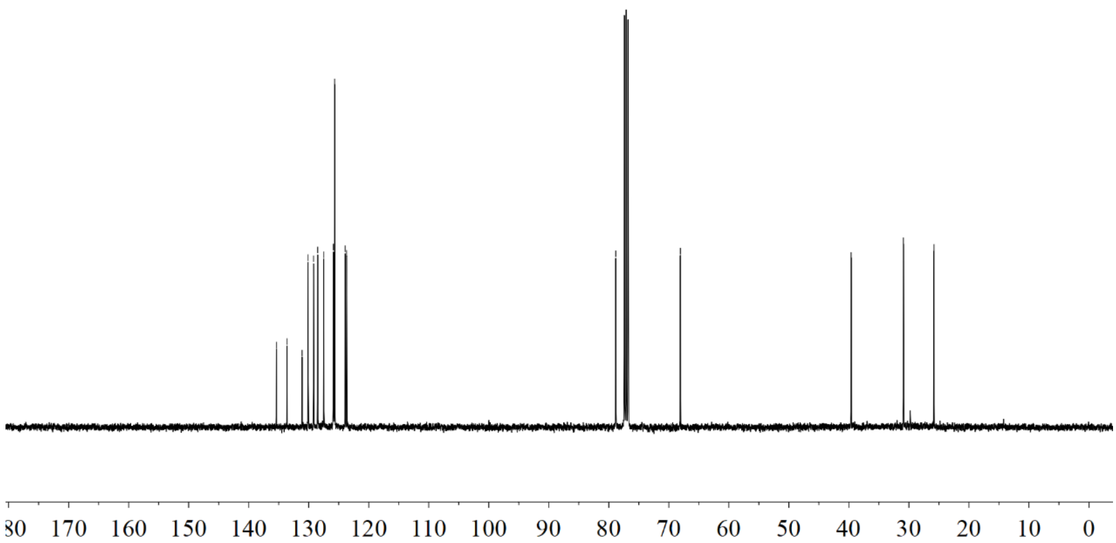

135.386
133.606
131.100
130.094
129.191
128.502
127.492
125.881
125.680
123.924
123.680
78.842
68.074
39.628
30.915
25.820
80 170 160 150 140 130 120 110 100 90 80 70 60 50 40 30 20 10 0

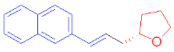

8s

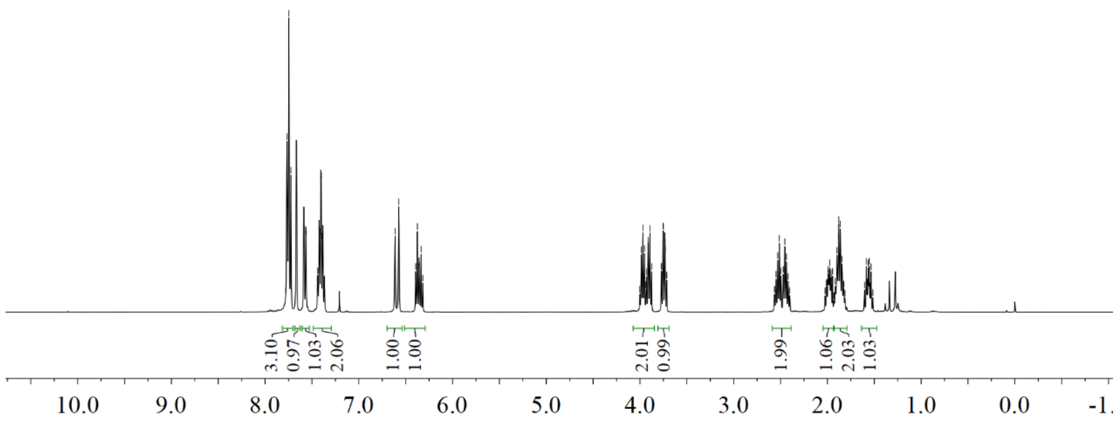

7.767
7.746
7.725
7.668
7.664
7.588
7.584
7.567
7.562
7.423
7.419
7.406
7.401
7.388
7.383
6.613
6.573
6.392
6.375
6.357
6.353
6.335
3.986
3.969
3.952
3.914
3.911
3.894
3.877
3.770
3.752
3.735
3.731
2.530
2.512
2.471
2.454
2.437
1.994
1.974
1.898
1.882
1.879
1.862
1.849
1.844
1.584
1.564
1.555
1.535
3.10
0.97
1.03
2.06
1.00
1.00
2.01
0.99
1.99
1.06
2.03
1.03
10.0 9.0 8.0 7.0 6.0 5.0 4.0 3.0 2.0 1.0 0.0 -1.

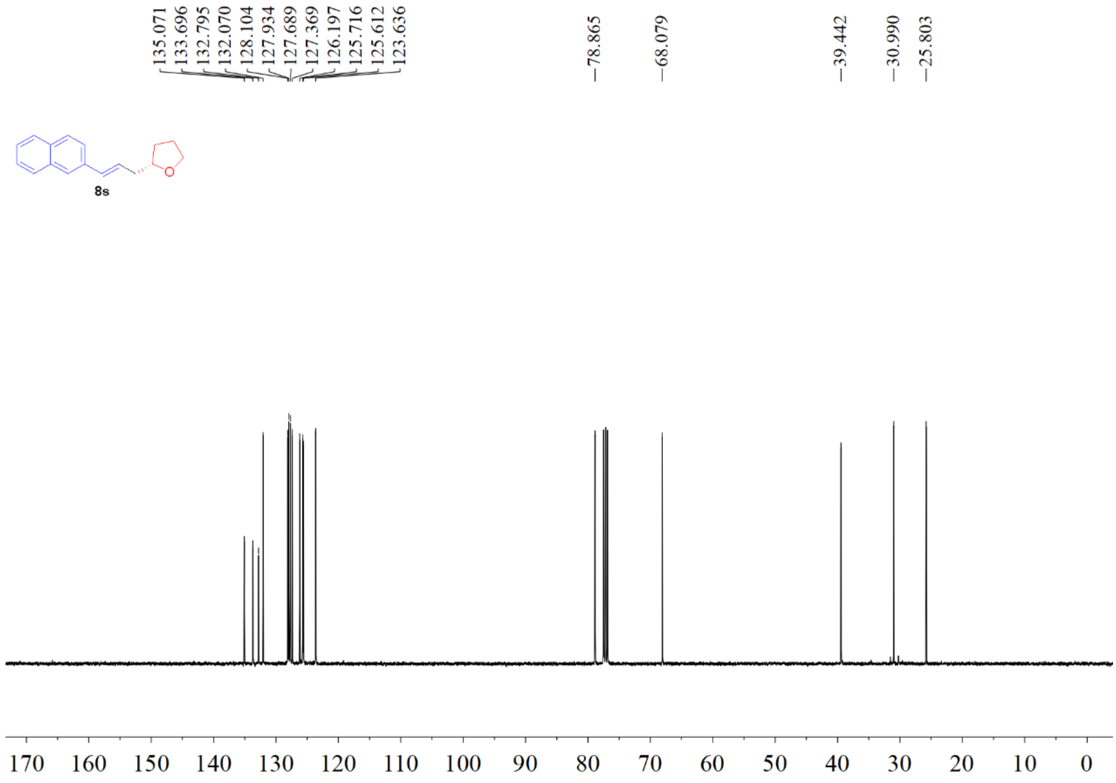
135.071
133.696
132.795
132.070
128.104
127.934
127.689
127.369
126.197
125.716
125.612
123.636
78.865
68.079
39.442
30.990
25.803
8s
170 160 150 140 130 120 110 100 90 80 70 60 50 40 30 20 10 0

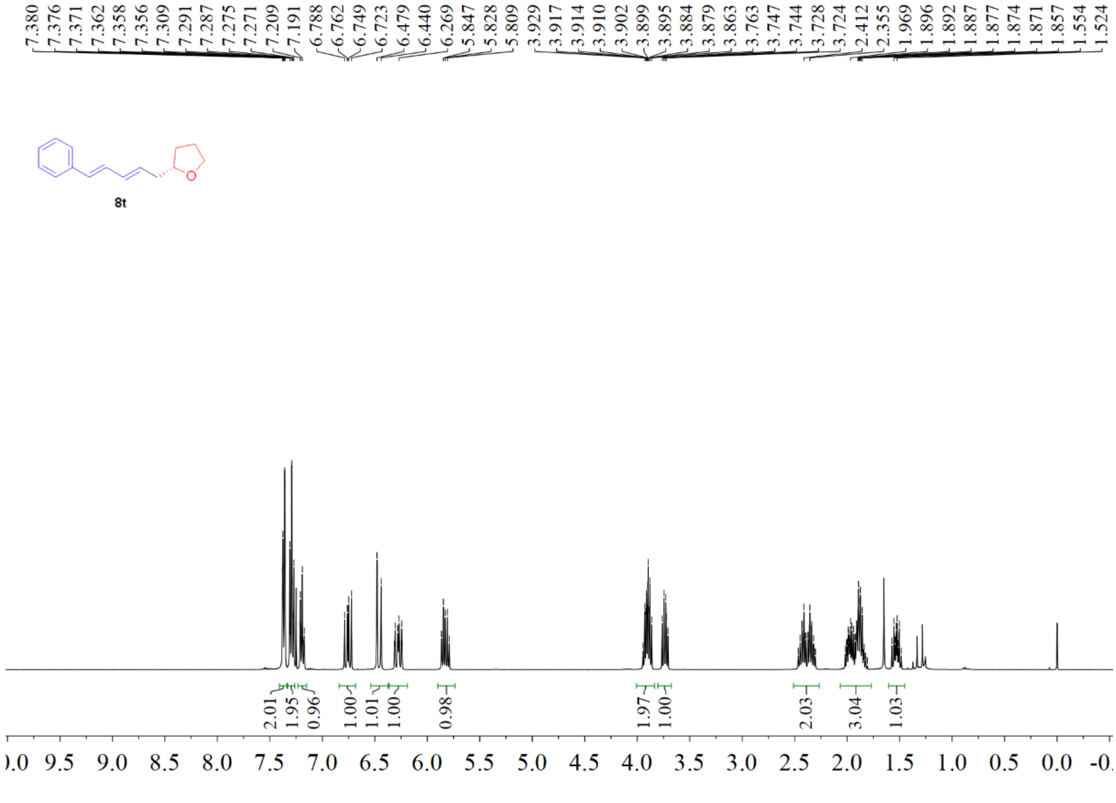
7.380
7.376
7.371
7.362
7.358
7.356
7.309
7.291
7.287
7.275
7.271
7.209
7.191
6.788
6.762
6.749
6.723
6.479
6.440
6.269
5.847
5.828
5.809
3.929
3.917
3.914
3.910
3.902
3.899
3.895
3.884
3.879
3.863
3.763
3.747
3.744
3.728
3.724
2.412
2.355
1.969
1.896
1.892
1.887
1.877
1.874
1.871
1.857
1.554
1.524
8t
2.01
1.95
0.96
1.00
1.01
1.00
0.98
1.97
1.00
2.03
3.04
1.03
9.5 9.0 8.5 8.0 7.5 7.0 6.5 6.0 5.5 5.0 4.5 4.0 3.5 3.0 2.5 2.0 1.5 1.0 0.5 0.0

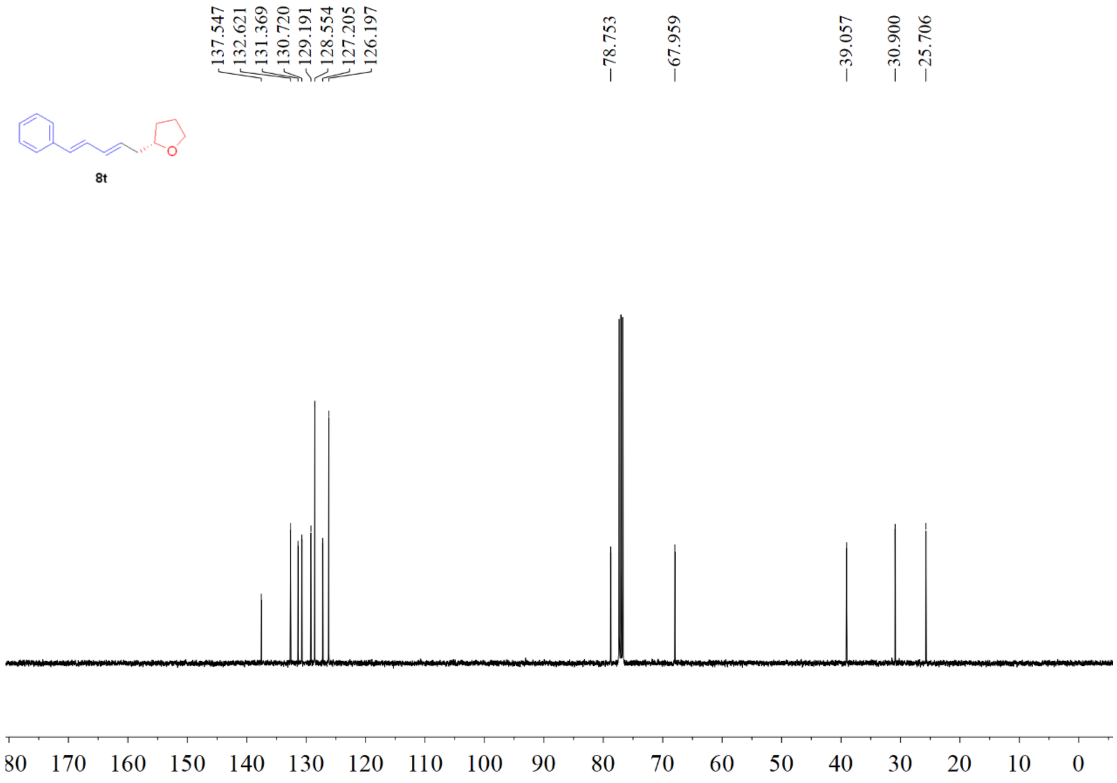

137.547
132.621
131.369
130.720
129.191
128.554
127.205
126.197
78.753
67.959
39.057
30.900
25.706
8t
80 170 160 150 140 130 120 110 100 90 80 70 60 50 40 30 20 10 0

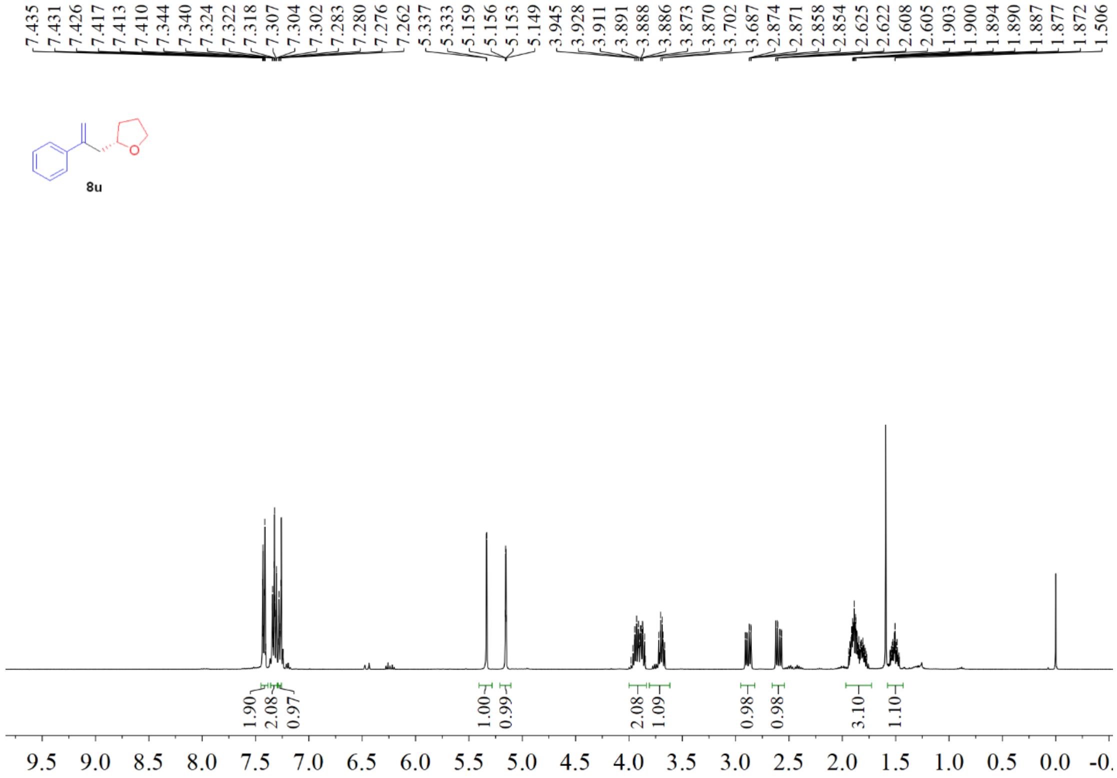

7.435
7.431
7.426
7.417
7.413
7.410
7.344
7.340
7.324
7.322
7.318
7.307
7.304
7.302
7.283
7.280
7.276
7.262
5.337
5.333
5.159
5.156
5.153
5.149
3.945
3.928
3.911
3.891
3.888
3.886
3.873
3.870
3.702
3.687
2.874
2.871
2.858
2.854
2.625
2.622
2.608
2.605
1.903
1.900
1.894
1.890
1.887
1.877
1.872
1.506
8u
1.90
2.08
0.97
1.00
0.99
2.08
1.09
0.98
0.98
3.10
1.10
9.5 9.0 8.5 8.0 7.5 7.0 6.5 6.0 5.5 5.0 4.5 4.0 3.5 3.0 2.5 2.0 1.5 1.0 0.5 0.0 -0.

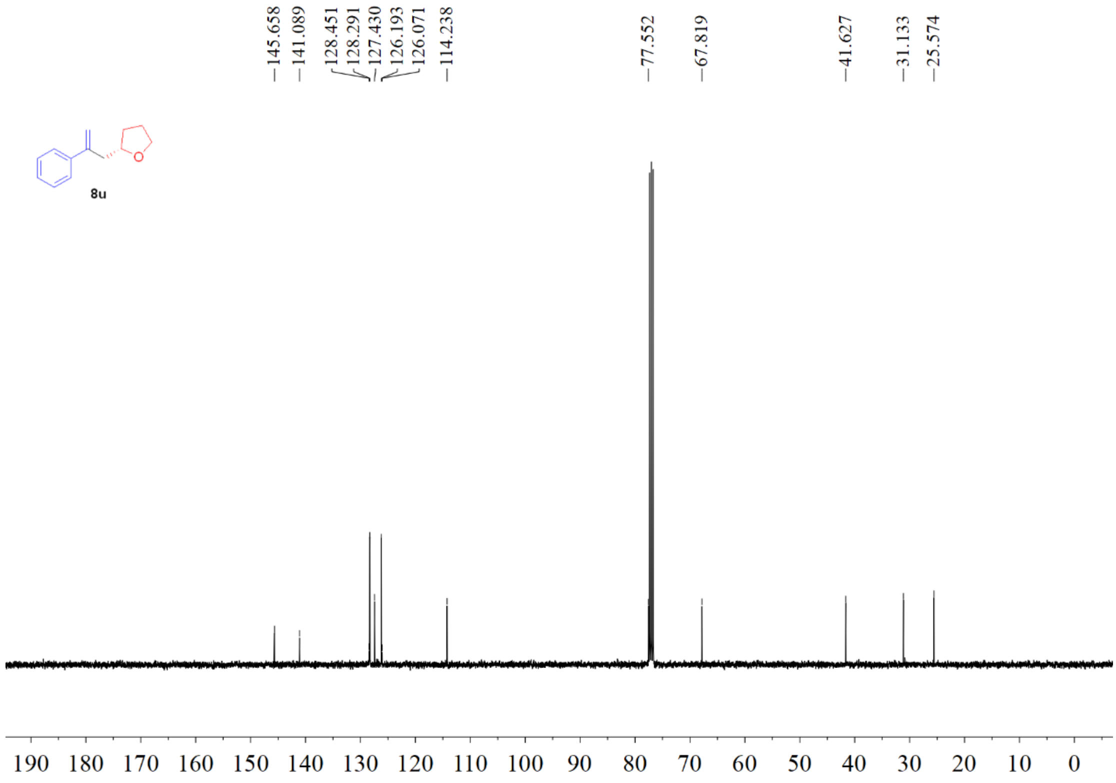
145.658
141.089
128.451
128.291
127.430
126.193
126.071
114.238
77.552
67.819
41.627
31.133
25.574
8u
190 180 170 160 150 140 130 120 110 100 90 80 70 60 50 40 30 20 10 0

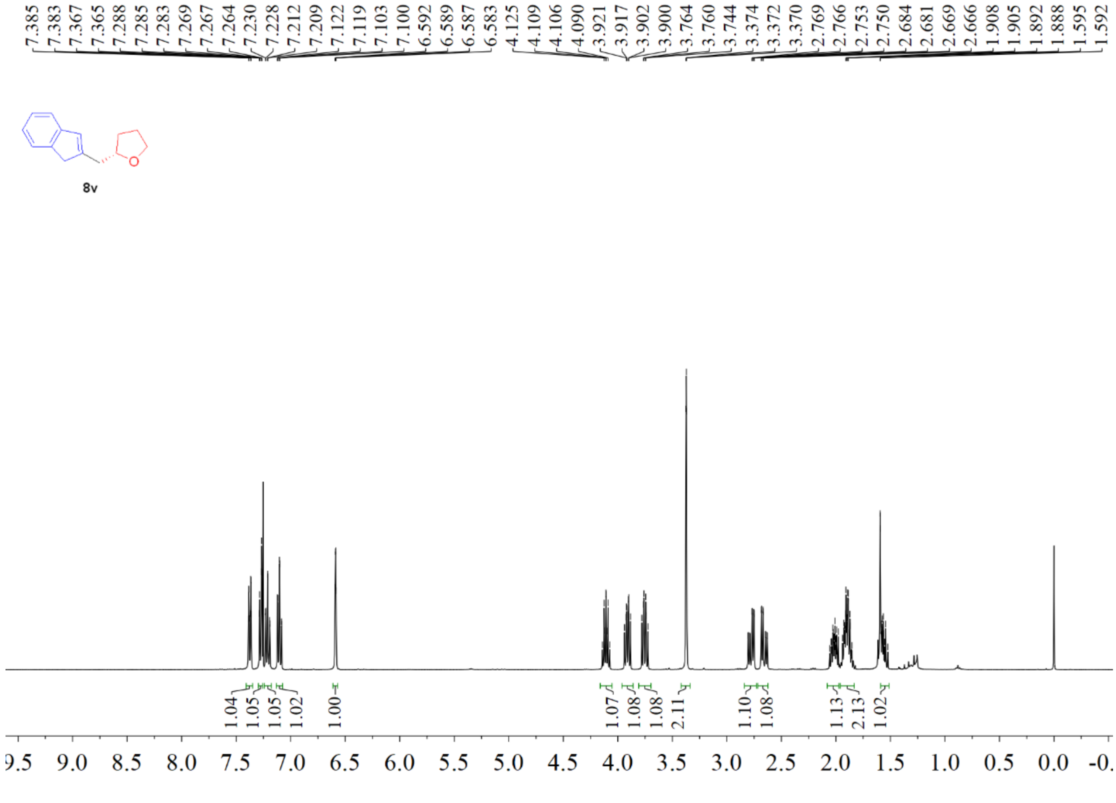
7.385
7.383
7.367
7.365
7.288
7.285
7.283
7.269
7.267
7.264
7.230
7.228
7.212
7.209
7.122
7.119
7.103
7.100
6.592
6.589
6.587
6.583
4.125
4.109
4.106
4.090
3.921
3.917
3.902
3.900
3.764
3.760
3.744
3.374
3.372
3.370
2.769
2.766
2.753
2.750
2.684
2.681
2.669
2.666
1.908
1.905
1.892
1.888
1.595
1.592
8v
1.04
1.05
1.05
1.02
1.00
1.07
1.08
1.08
2.11
1.10
1.08
1.13
2.13
1.02
9.5 9.0 8.5 8.0 7.5 7.0 6.5 6.0 5.5 5.0 4.5 4.0 3.5 3.0 2.5 2.0 1.5 1.0 0.5 0.0 -0.

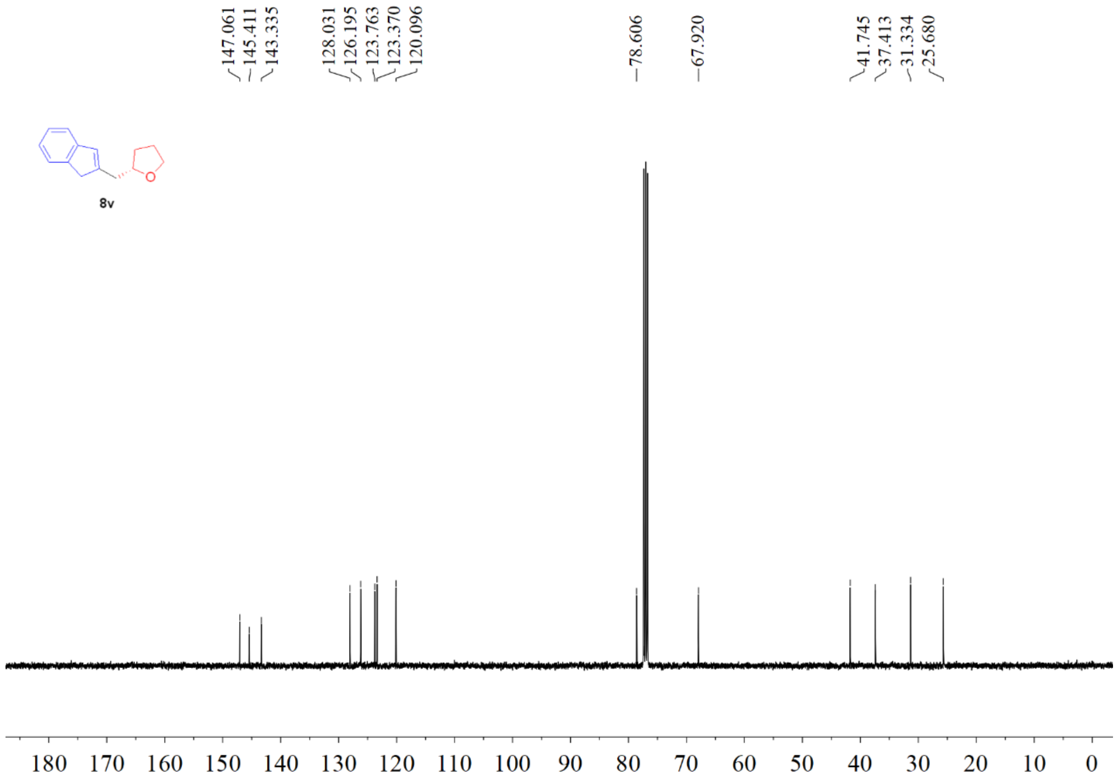
147.061
145.411
143.335
128.031
126.195
123.763
123.370
120.096
78.606
67.920
41.745
37.413
31.334
25.680
8v
180 170 160 150 140 130 120 110 100 90 80 70 60 50 40 30 20 10 0

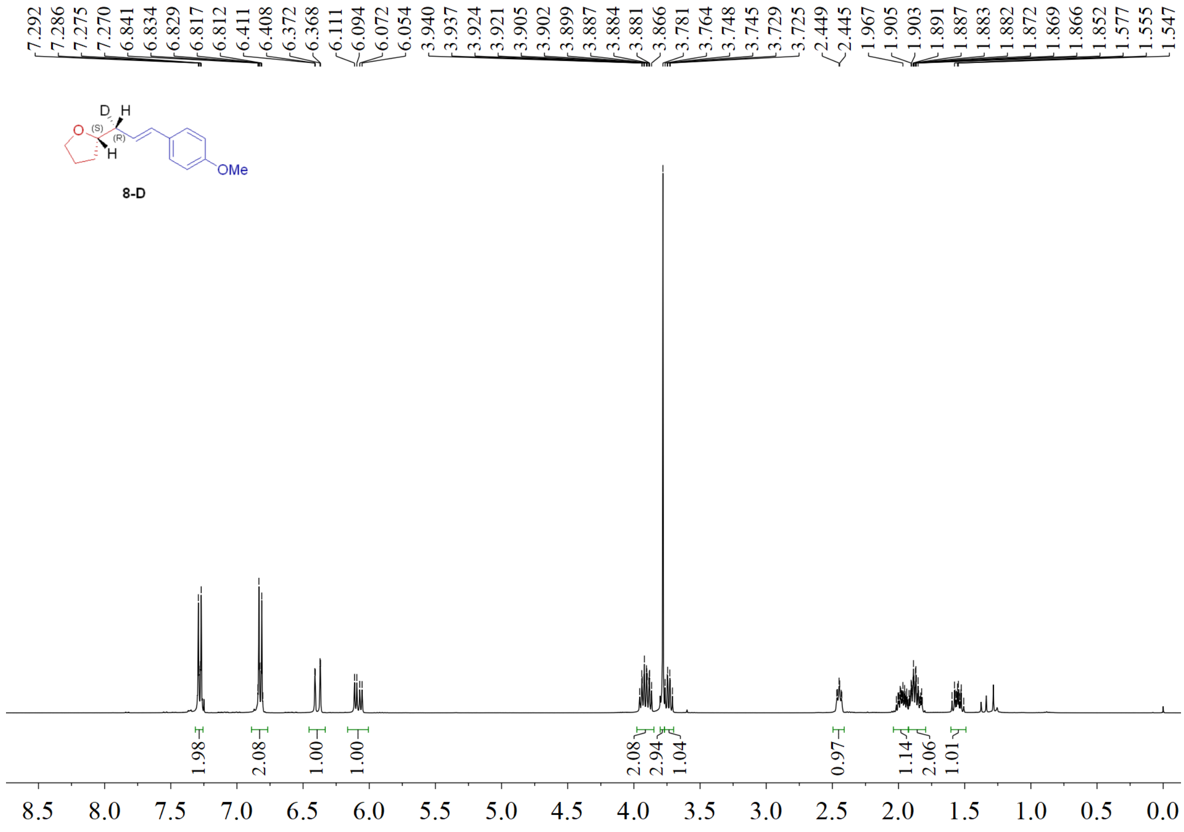
7.292
7.286
7.275
7.270
6.841
6.834
6.829
6.817
6.812
6.411
6.408
6.372
6.368
6.111
6.094
6.072
6.054
3.940
3.937
3.924
3.921
3.905
3.902
3.899
3.887
3.884
3.881
3.866
3.781
3.764
3.748
3.745
3.729
3.725
2.449
2.445
1.967
1.905
1.903
1.891
1.887
1.883
1.882
1.872
1.869
1.866
1.852
1.577
1.555
1.547
OMe
8-D
1.98
2.08
1.00
1.00
2.08
2.94
1.04
0.97
1.14
2.06
1.01
8.5 8.0 7.5 7.0 6.5 6.0 5.5 5.0 4.5 4.0 3.5 3.0 2.5 2.0 1.5 1.0 0.5 0.0

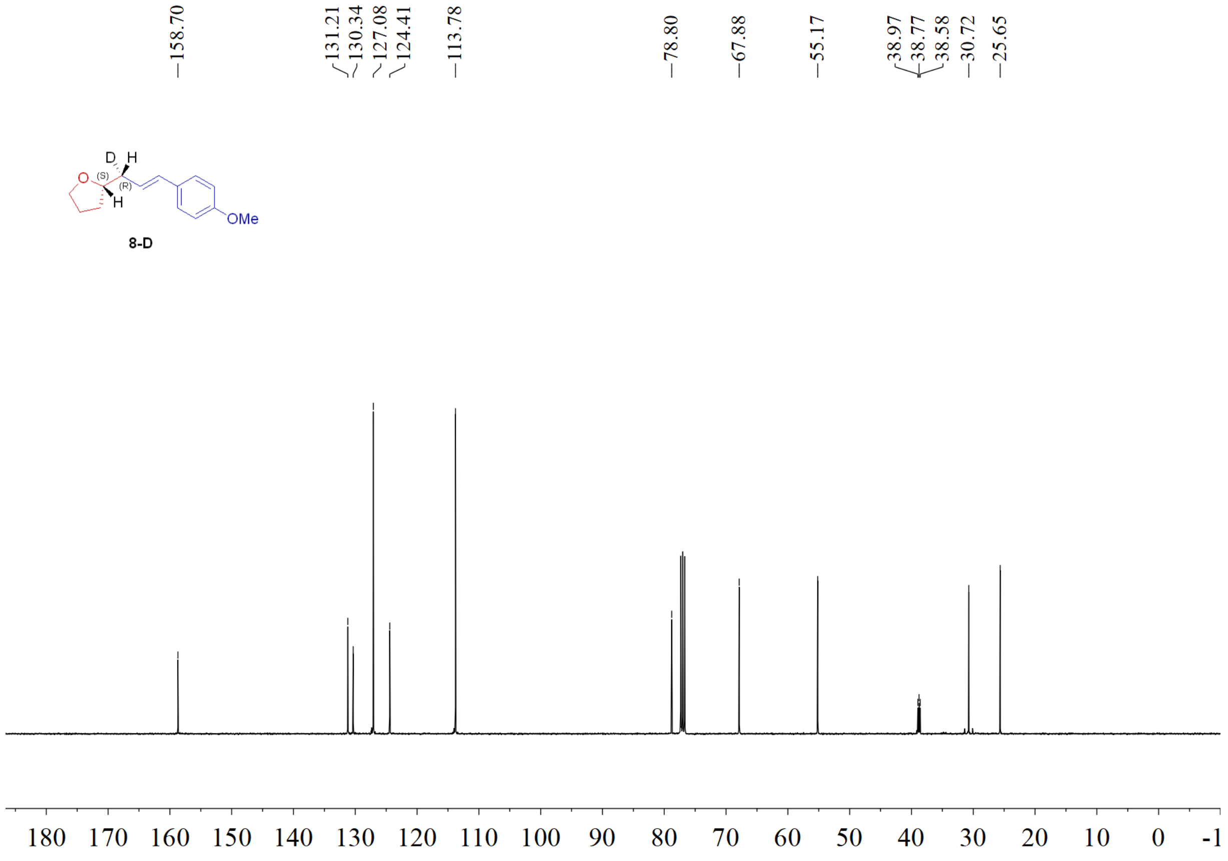
158.70
131.21
130.34
127.08
124.41
113.78
78.80
67.88
55.17
38.97
38.77
38.58
30.72
25.65
D
H
O
(S)
(R)
H
OMe
8-D
180 170 160 150 140 130 120 110 100 90 80 70 60 50 40 30 20 10 0 -1

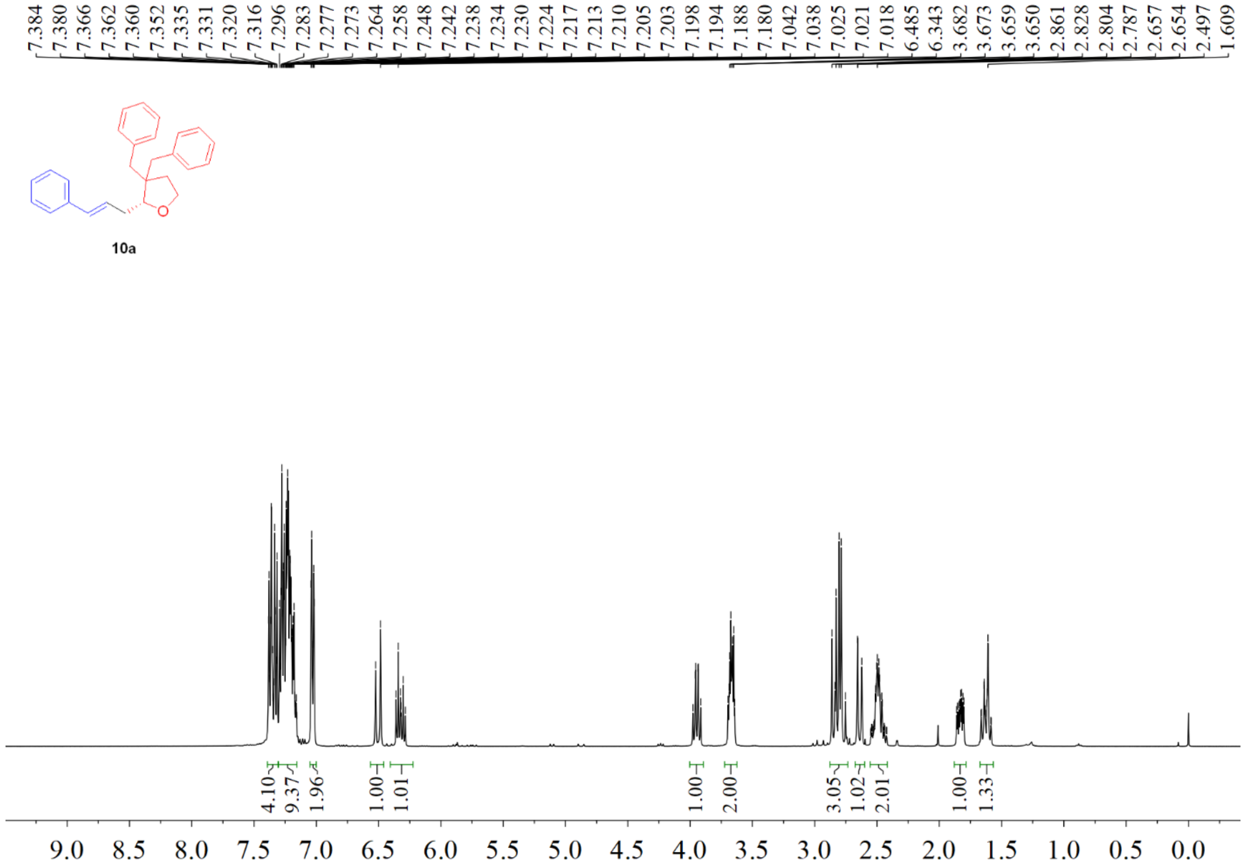
7.384
7.380
7.366
7.362
7.360
7.352
7.335
7.331
7.320
7.316
7.296
7.283
7.277
7.273
7.264
7.258
7.248
7.242
7.238
7.234
7.230
7.224
7.217
7.213
7.210
7.205
7.203
7.198
7.194
7.188
7.180
7.042
7.038
7.025
7.021
7.018
6.485
6.343
3.682
3.673
3.659
3.650
2.861
2.828
2.804
2.787
2.657
2.654
2.497
1.609
10a
4.10
9.37
1.96
1.00
1.01
1.00
2.00
3.05
1.02
2.01
1.00
1.33
9.0 8.5 8.0 7.5 7.0 6.5 6.0 5.5 5.0 4.5 4.0 3.5 3.0 2.5 2.0 1.5 1.0 0.5 0.0

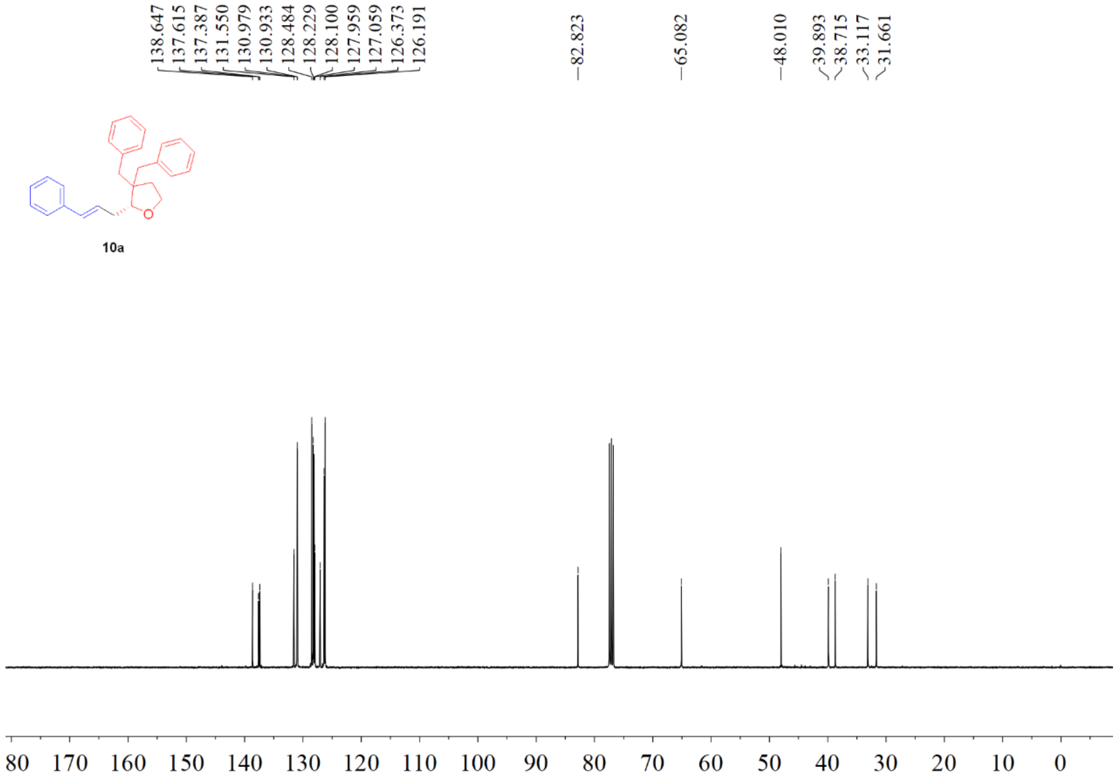
138.647
137.615
137.387
131.550
130.979
130.933
128.484
128.229
128.100
127.959
127.059
126.373
126.191
82.823
65.082
48.010
39.893
38.715
33.117
31.661
10a
80 170 160 150 140 130 120 110 100 90 80 70 60 50 40 30 20 10 0

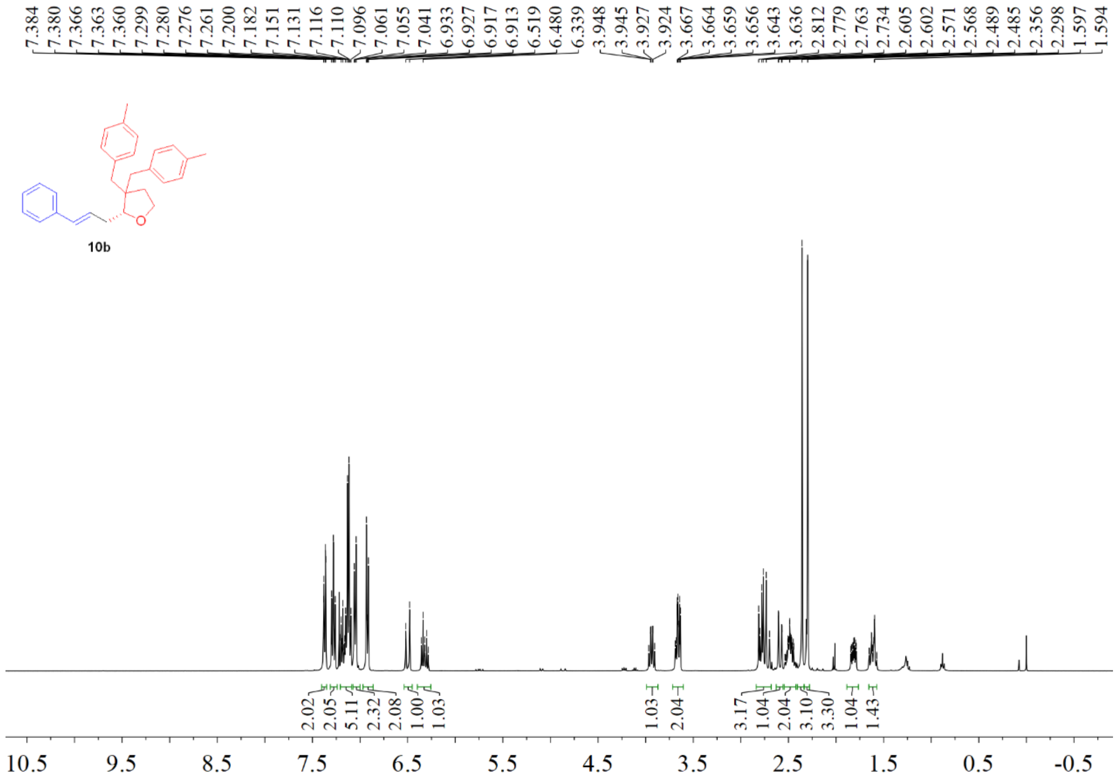
7.384
7.380
7.366
7.363
7.360
7.299
7.280
7.276
7.261
7.200
7.182
7.151
7.131
7.116
7.110
7.096
7.061
7.055
7.041
6.933
6.927
6.917
6.913
6.519
6.480
6.339
3.948
3.945
3.927
3.924
3.667
3.664
3.659
3.656
3.643
3.636
2.812
2.779
2.763
2.734
2.605
2.602
2.571
2.568
2.489
2.485
2.356
2.298
1.597
1.594
10b
2.02
2.05
5.11
2.32
2.08
1.00
1.03
1.03
2.04
3.17
1.04
2.04
3.10
3.30
1.04
1.43
10.5
9.5
8.5
7.5
6.5
5.5
4.5
3.5
2.5
1.5
0.5
-0.5

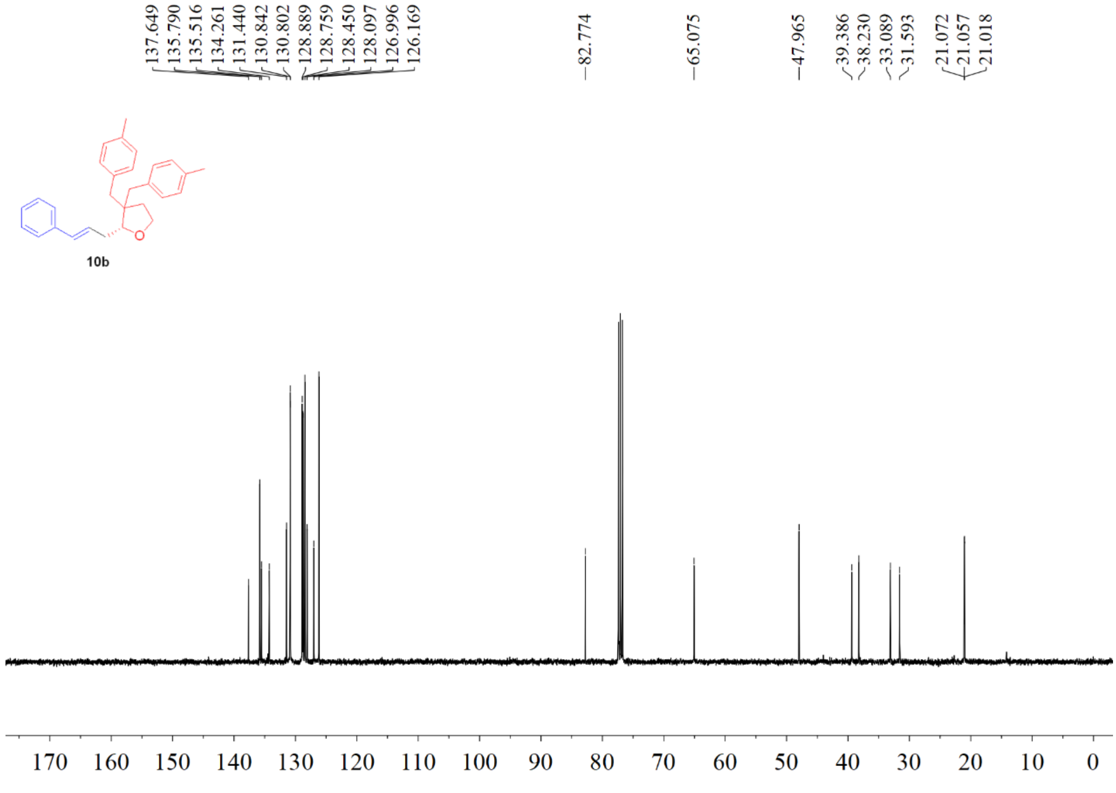
137.649
135.790
135.516
134.261
131.440
130.842
130.802
128.889
128.759
128.450
128.097
126.996
126.169
82.774
65.075
47.965
39.386
38.230
33.089
31.593
21.072
21.057
21.018
10b
170
160
150
140
130
120
110
100
90
80
70
60
50
40
30
20
10
0

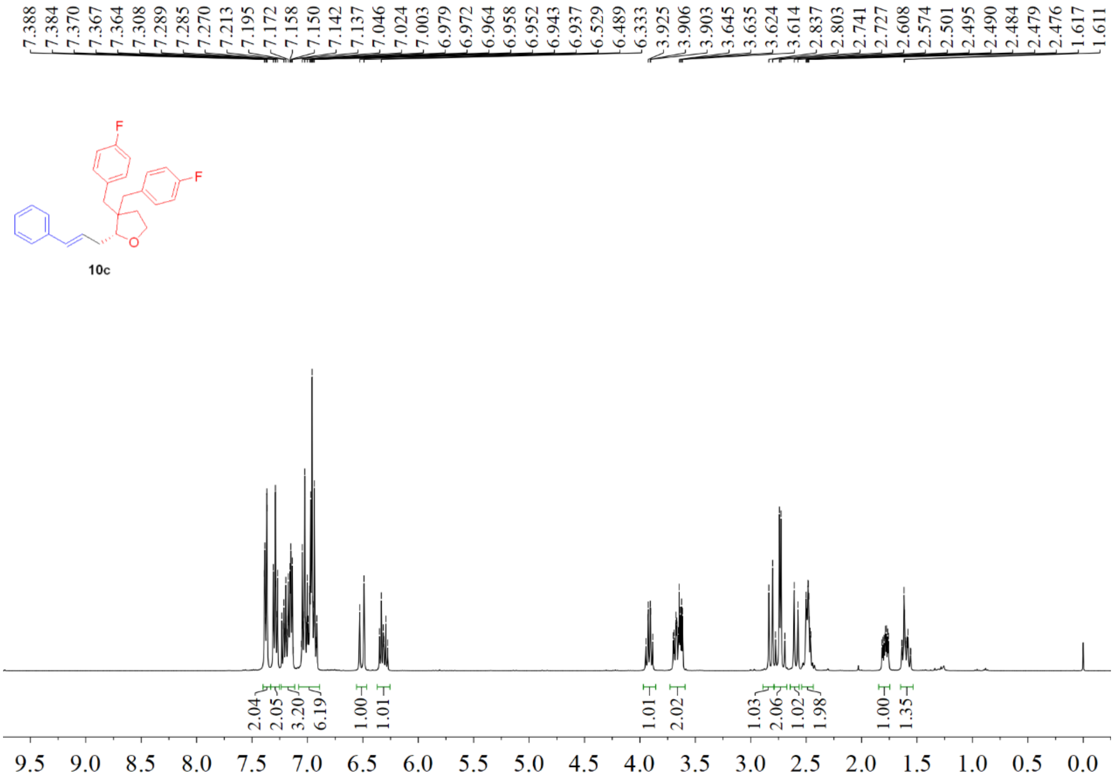
7.388
7.384
7.370
7.367
7.364
7.308
7.289
7.285
7.270
7.213
7.195
7.172
7.158
7.150
7.142
7.137
7.046
7.024
7.003
6.979
6.972
6.964
6.958
6.952
6.943
6.937
6.529
6.489
6.333
3.925
3.906
3.903
3.645
3.635
3.624
3.614
2.837
2.803
2.741
2.727
2.608
2.574
2.501
2.495
2.490
2.484
2.479
2.476
1.617
1.611
F
F
O
10c
2.04
2.05
3.20
6.19
1.00
1.01
1.01
2.02
1.03
2.06
1.02
1.98
1.00
1.35
9.5 9.0 8.5 8.0 7.5 7.0 6.5 6.0 5.5 5.0 4.5 4.0 3.5 3.0 2.5 2.0 1.5 1.0 0.5 0.0

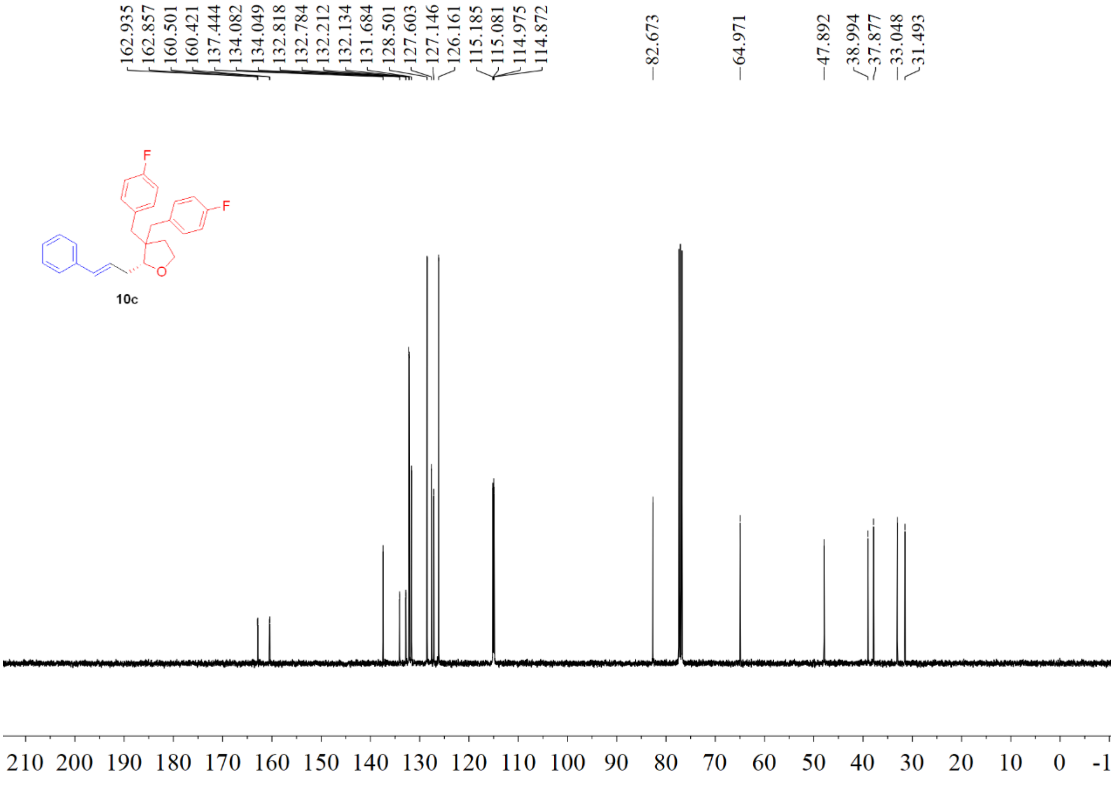
162.935
162.857
160.501
160.421
137.444
134.082
134.049
132.818
132.784
132.212
132.134
131.684
128.501
127.603
127.146
126.161
115.185
115.081
114.975
114.872
82.673
64.971
47.892
38.994
37.877
33.048
31.493
F
F
O
10c
210 200 190 180 170 160 150 140 130 120 110 100 90 80 70 60 50 40 30 20 10 0 -1

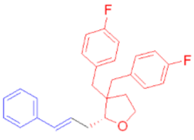

10c

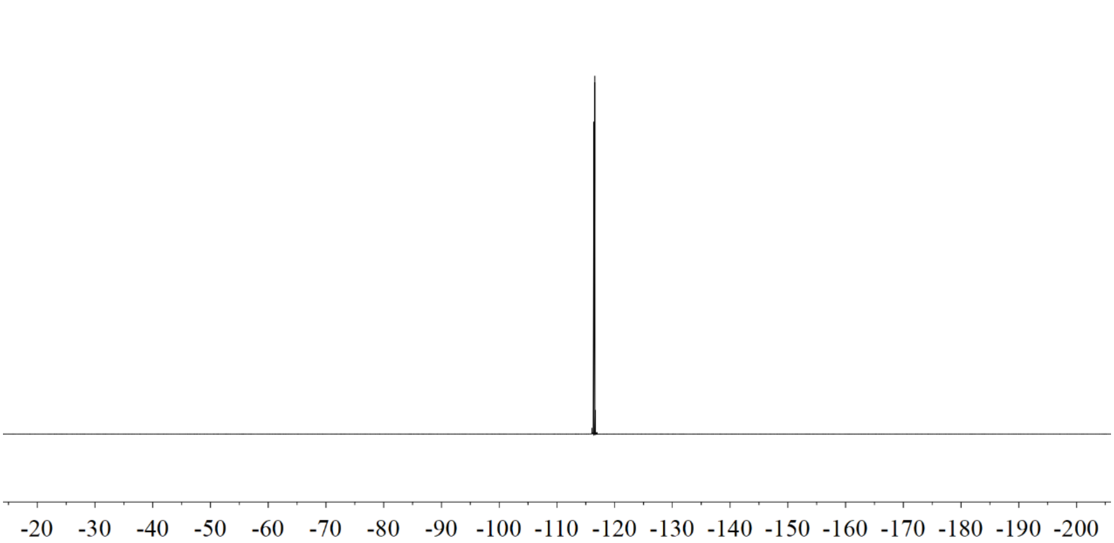
-116.369
-116.567
-20 -30 -40 -50 -60 -70 -80 -90 -100 -110 -120 -130 -140 -150 -160 -170 -180 -190 -200

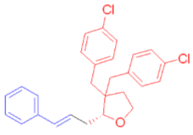

10d

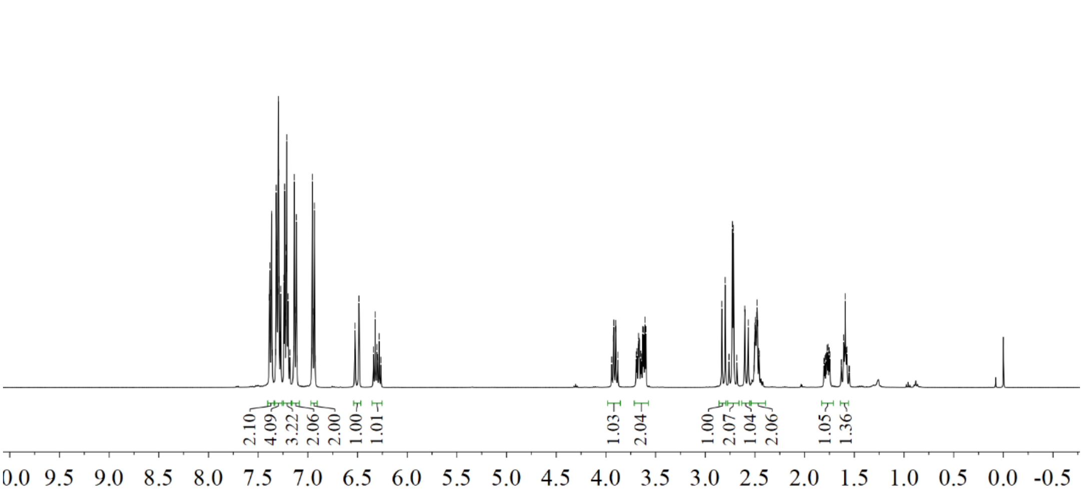
7.387 7.383 7.369 7.366 7.363 7.318 7.313 7.311 7.307 7.302 7.297 7.293 7.289 7.274 7.241 7.234 7.229 7.221 7.218 7.213 7.200 7.137 7.132 7.121 7.116 6.955 6.950 6.939 6.934 6.525 6.485 6.322 3.920 3.902 3.899 3.608 3.598 2.834 2.800 2.727 2.716 2.602 2.600 2.567 2.500 2.495 2.483 2.477 2.473 1.591
2.10 4.09 3.22 2.06 2.00 1.00 1.01 1.03 2.04 1.00 2.07 1.04 2.06 1.05 1.36
0.0 9.5 9.0 8.5 8.0 7.5 7.0 6.5 6.0 5.5 5.0 4.5 4.0 3.5 3.0 2.5 2.0 1.5 1.0 0.5 0.0 -0.5

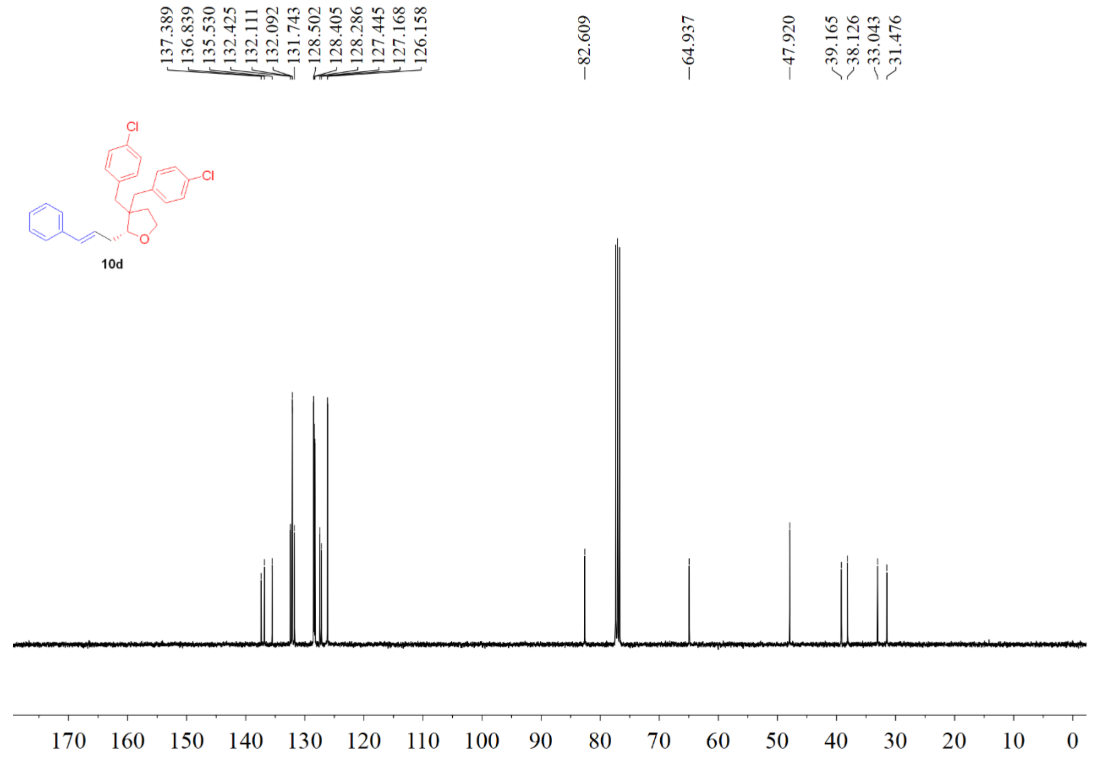
137.389
136.839
135.530
132.425
132.111
132.092
131.743
128.502
128.405
128.286
127.445
127.168
126.158
82.609
64.937
47.920
39.165
38.126
33.043
31.476
Cl
Cl
O
10d
170 160 150 140 130 120 110 100 90 80 70 60 50 40 30 20 10 0

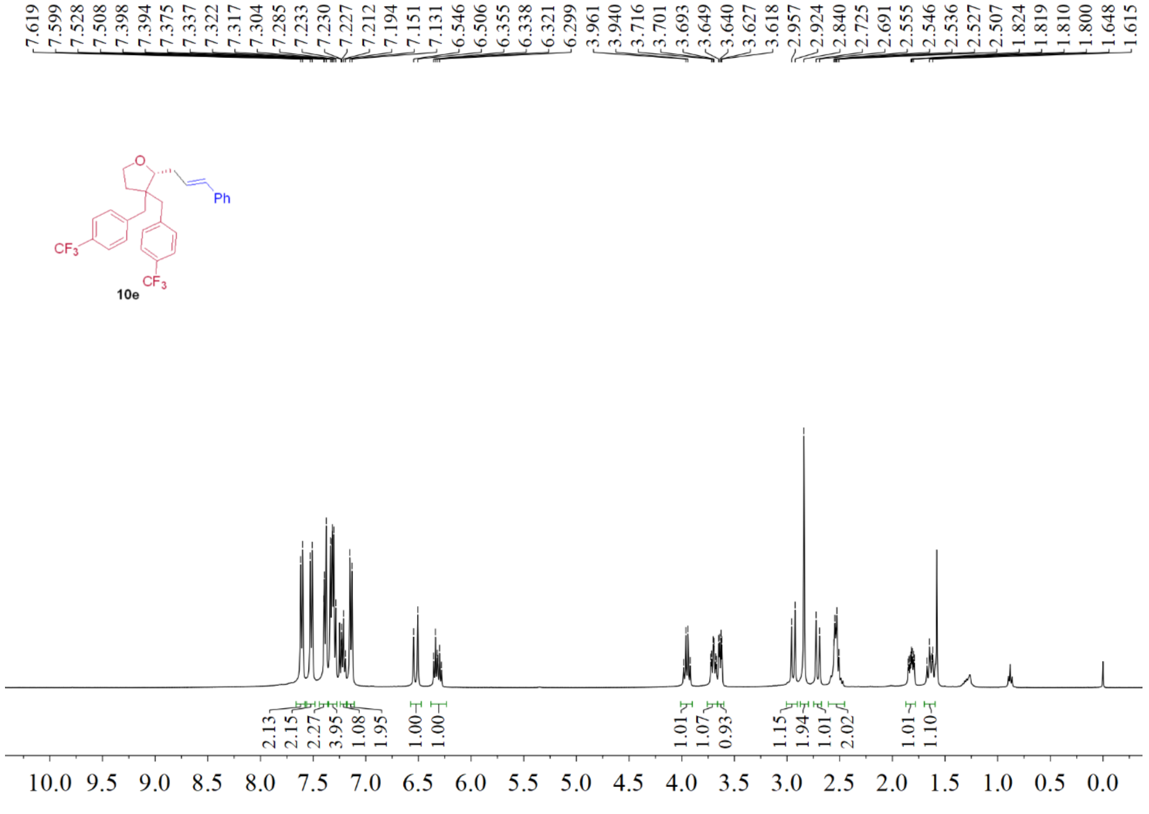
7.619
7.599
7.528
7.508
7.398
7.394
7.375
7.337
7.322
7.317
7.304
7.285
7.233
7.230
7.227
7.212
7.194
7.151
7.131
6.546
6.506
6.355
6.338
6.321
6.299
3.961
3.940
3.716
3.701
3.693
3.649
3.640
3.627
3.618
2.957
2.924
2.840
2.725
2.691
2.555
2.546
2.536
2.527
2.507
1.824
1.819
1.810
1.800
1.648
1.615
O
Ph
CF3
CF3
10e
2.13
2.15
2.27
3.95
1.08
1.95
1.00
1.00
1.01
1.07
0.93
1.15
1.94
1.01
2.02
1.01
1.10
10.0 9.5 9.0 8.5 8.0 7.5 7.0 6.5 6.0 5.5 5.0 4.5 4.0 3.5 3.0 2.5 2.0 1.5 1.0 0.5 0.0

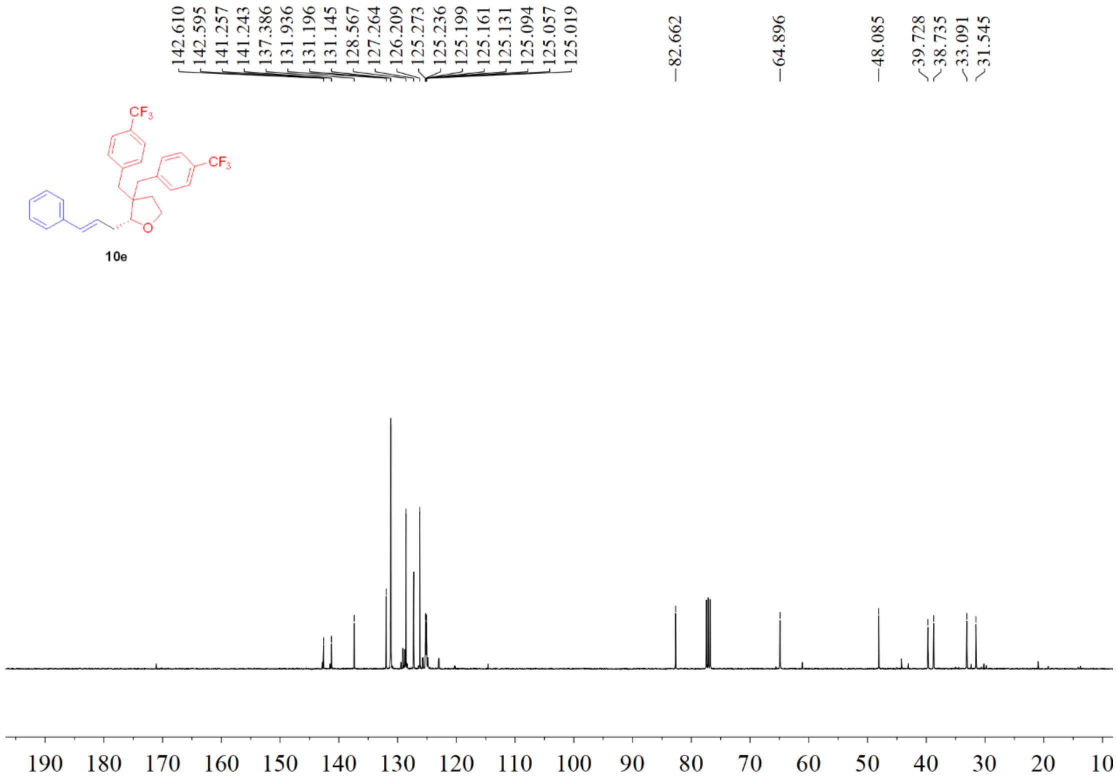
142.610
142.595
141.257
141.243
137.386
131.936
131.196
131.145
128.567
127.264
126.209
125.273
125.236
125.199
125.161
125.131
125.094
125.057
125.019
82.662
64.896
48.085
39.728
38.735
33.091
31.545
CF3
CF3
O
10e
190 180 170 160 150 140 130 120 110 100 90 80 70 60 50 40 30 20 10

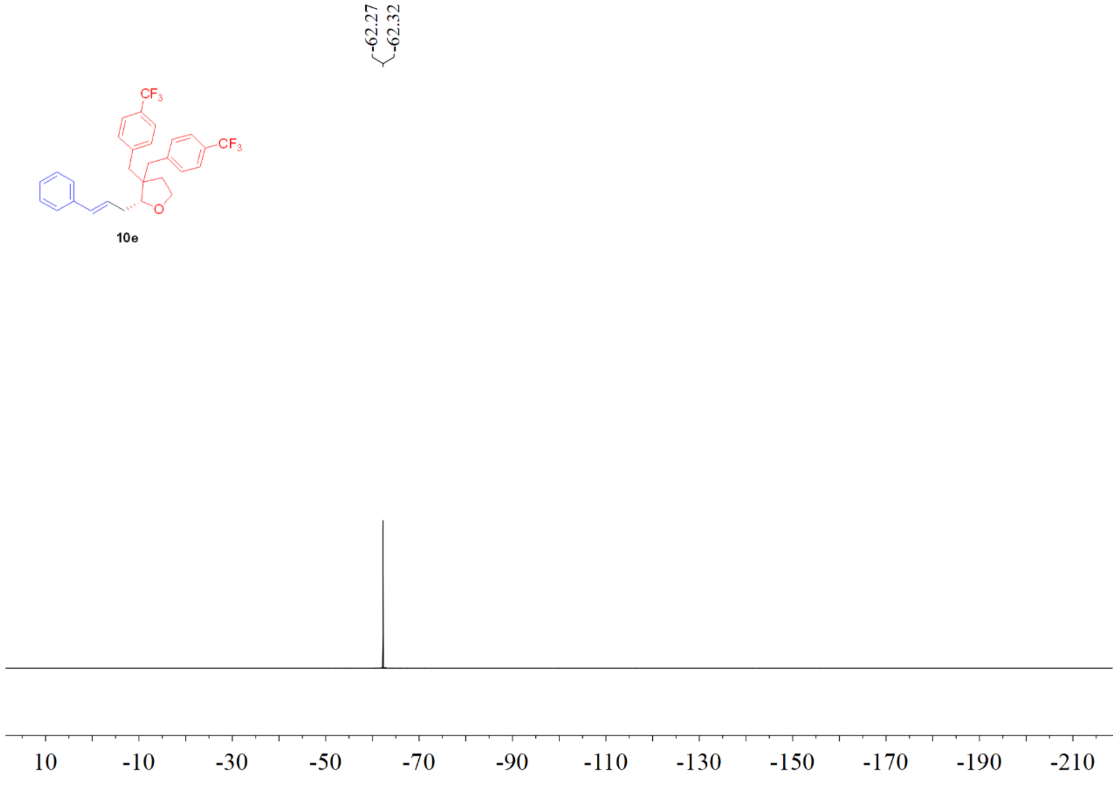
-62.27
-62.32
CF3
CF3
O
10e
10 -10 -30 -50 -70 -90 -110 -130 -150 -170 -190 -210

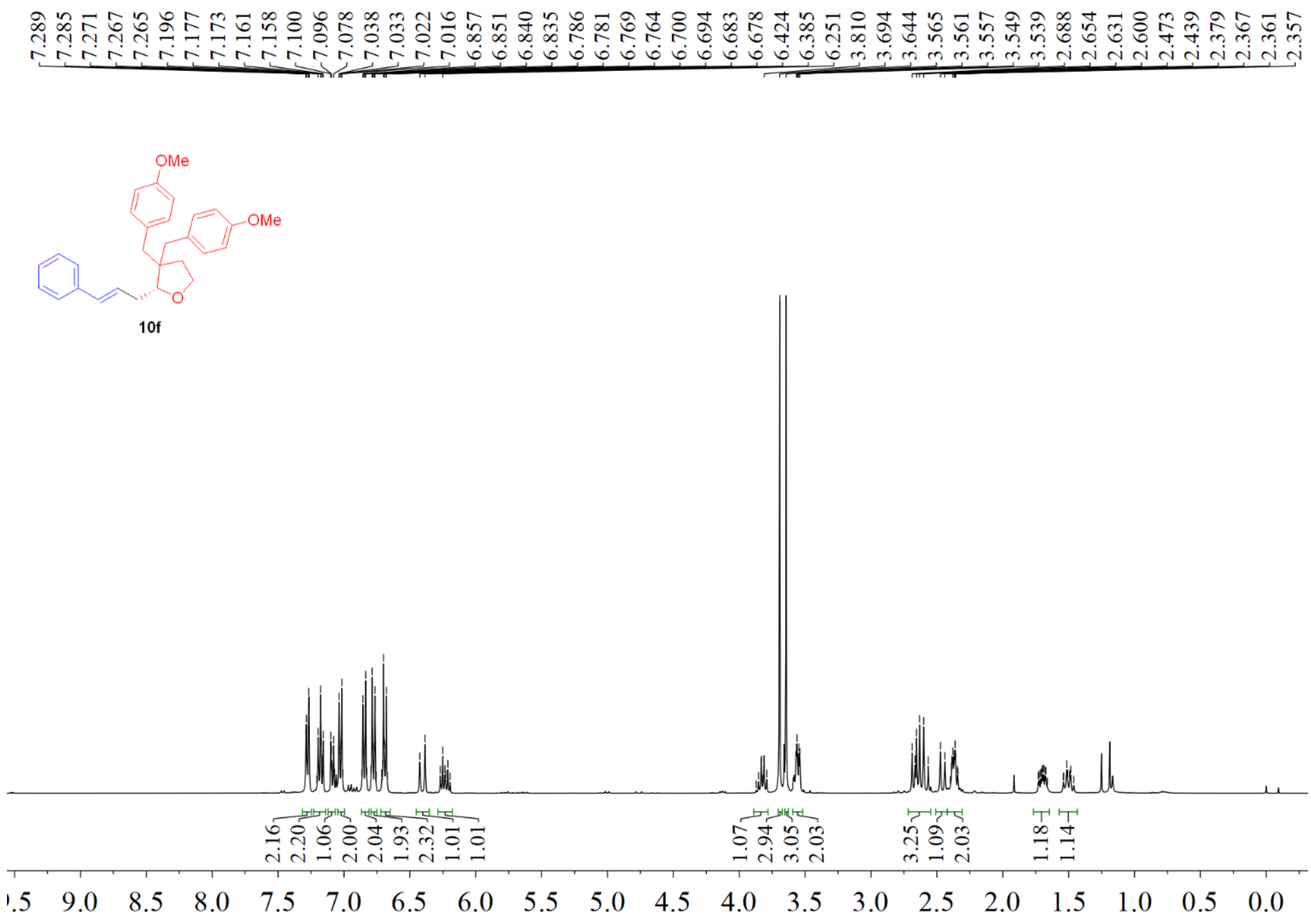
7.289
7.285
7.271
7.267
7.265
7.196
7.177
7.173
7.161
7.158
7.100
7.096
7.078
7.038
7.033
7.022
7.016
6.857
6.851
6.840
6.835
6.786
6.781
6.769
6.764
6.700
6.694
6.683
6.678
6.424
6.385
6.251
3.810
3.694
3.644
3.565
3.561
3.557
3.549
3.539
2.688
2.654
2.631
2.600
2.473
2.439
2.379
2.367
2.361
2.357
OMe
OMe
10f
2.16
2.20
1.06
2.00
2.04
1.93
2.32
1.01
1.01
1.07
2.94
3.05
2.03
3.25
1.09
2.03
1.18
1.14
9.0 8.5 8.0 7.5 7.0 6.5 6.0 5.5 5.0 4.5 4.0 3.5 3.0 2.5 2.0 1.5 1.0 0.5 0.0

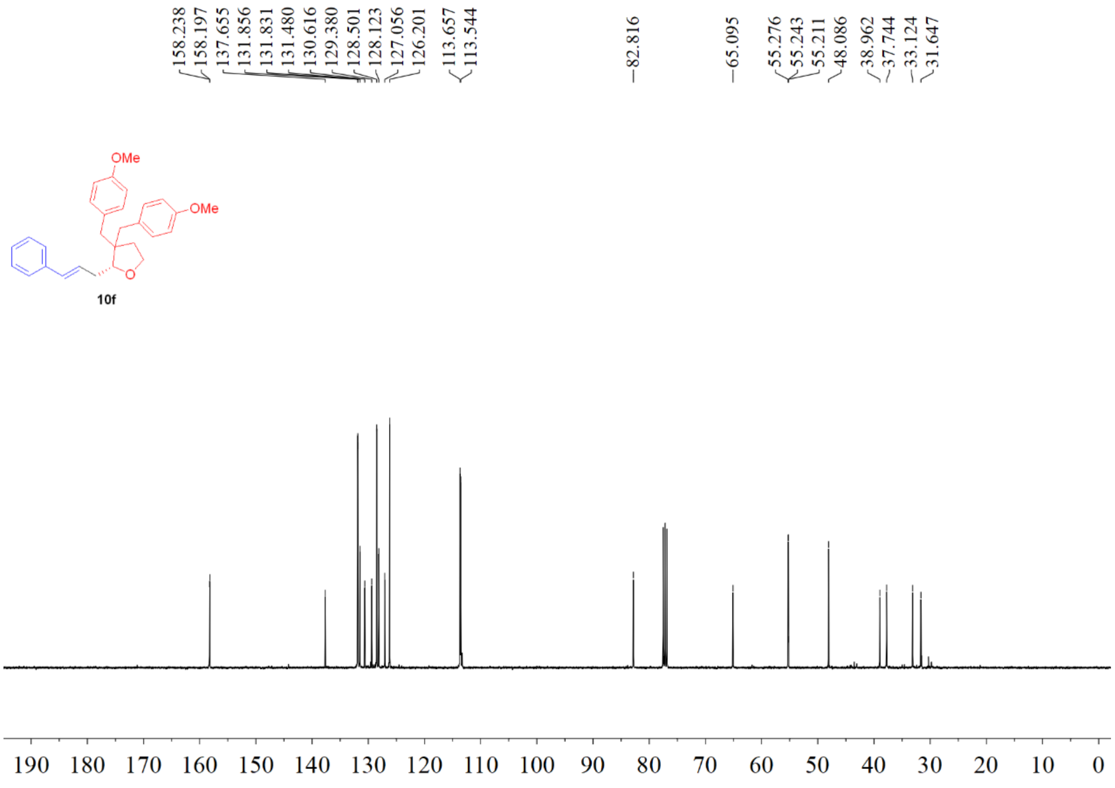
158.238
158.197
137.655
131.856
131.831
131.480
130.616
129.380
128.501
128.123
127.056
126.201
113.657
113.544
82.816
65.095
55.276
55.243
55.211
48.086
38.962
37.744
33.124
31.647
OMe
OMe
10f
190 180 170 160 150 140 130 120 110 100 90 80 70 60 50 40 30 20 10 0

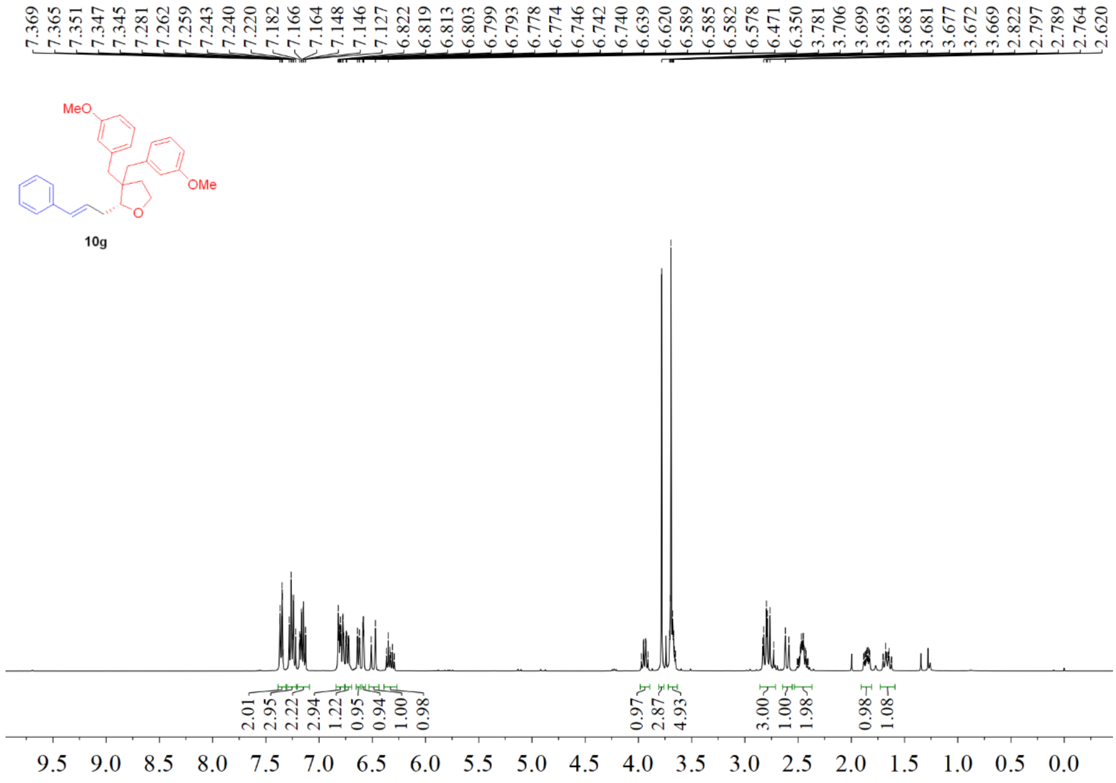
7.369
7.365
7.351
7.347
7.345
7.281
7.262
7.259
7.243
7.240
7.220
7.182
7.166
7.164
7.148
7.146
7.127
6.822
6.819
6.813
6.803
6.799
6.793
6.778
6.774
6.746
6.742
6.740
6.639
6.620
6.589
6.585
6.582
6.578
6.471
6.350
3.781
3.706
3.699
3.693
3.683
3.681
3.677
3.672
3.669
2.822
2.797
2.789
2.764
2.620
MeO
OMe
10g
2.01
2.95
2.22
2.94
1.22
0.95
0.94
1.00
0.98
0.97
2.87
4.93
3.00
1.00
1.98
0.98
1.08
9.5 9.0 8.5 8.0 7.5 7.0 6.5 6.0 5.5 5.0 4.5 4.0 3.5 3.0 2.5 2.0 1.5 1.0 0.5 0.0

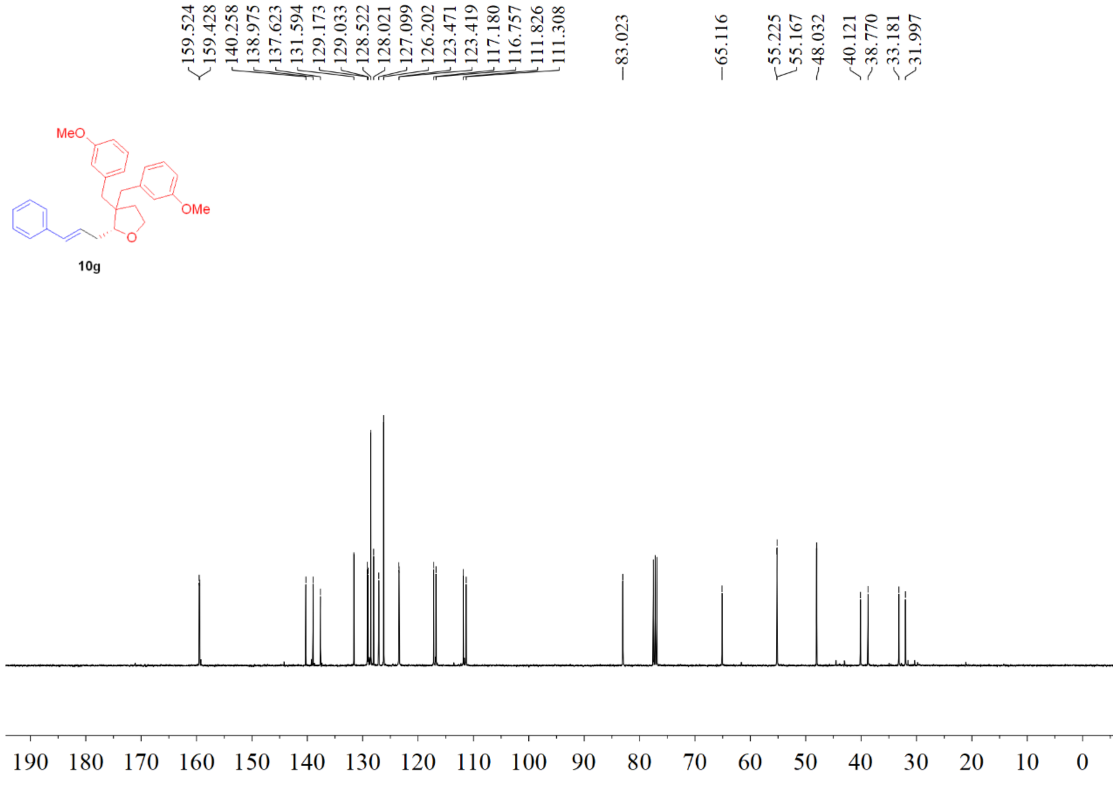
159.524
159.428
140.258
138.975
137.623
131.594
129.173
129.033
128.522
128.021
127.099
126.202
123.471
123.419
117.180
116.757
111.826
111.308
83.023
65.116
55.225
55.167
48.032
40.121
38.770
33.181
31.997
MeO
OMe
10g
190 180 170 160 150 140 130 120 110 100 90 80 70 60 50 40 30 20 10 0

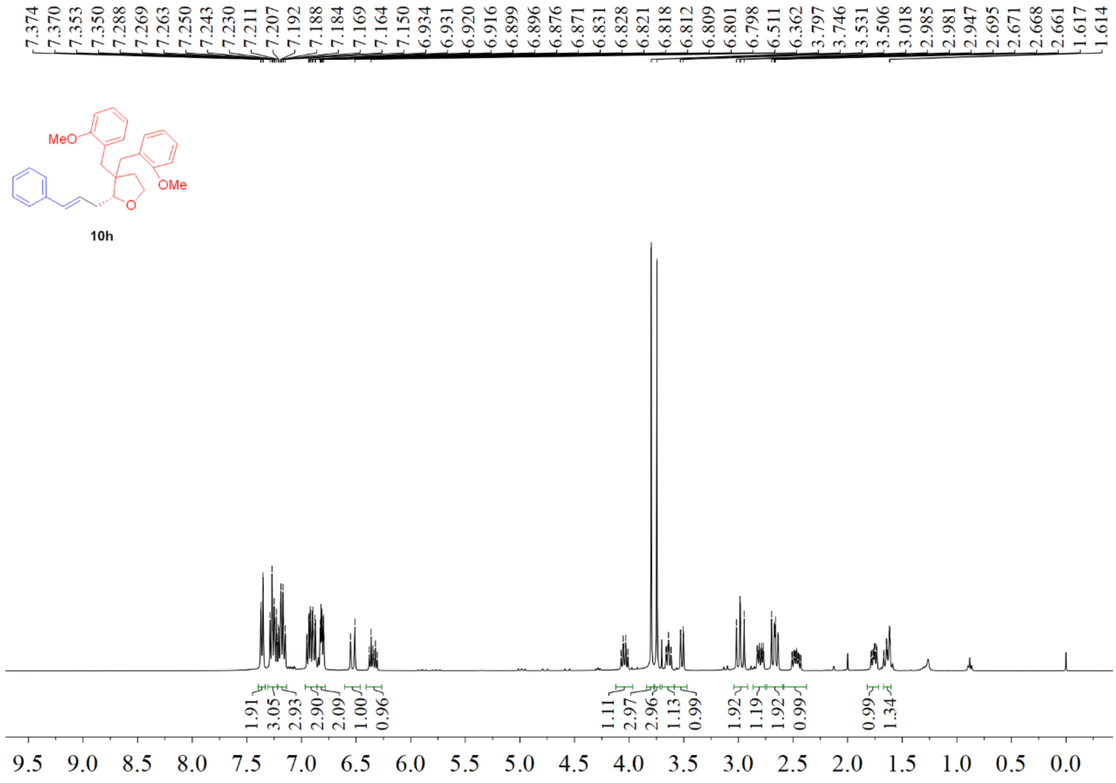
7.374 7.370 7.353 7.350 7.288 7.269 7.263 7.250 7.243 7.230 7.211 7.207 7.192 7.188 7.184 7.169 7.164 7.150 6.934 6.931 6.920 6.916 6.899 6.896 6.876 6.871 6.831 6.828 6.821 6.818 6.812 6.809 6.801 6.798 6.511 6.362 3.797 3.746 3.531 3.506 3.018 2.985 2.981 2.947 2.695 2.671 2.668 2.661 1.617 1.614
MeO
OMe
O
10h
1.91 3.05 2.93 2.90 2.09 1.00 0.96 1.11 2.97 2.96 1.13 0.99 1.92 1.19 1.92 0.99 0.99 1.34
9.5 9.0 8.5 8.0 7.5 7.0 6.5 6.0 5.5 5.0 4.5 4.0 3.5 3.0 2.5 2.0 1.5 1.0 0.5 0.0

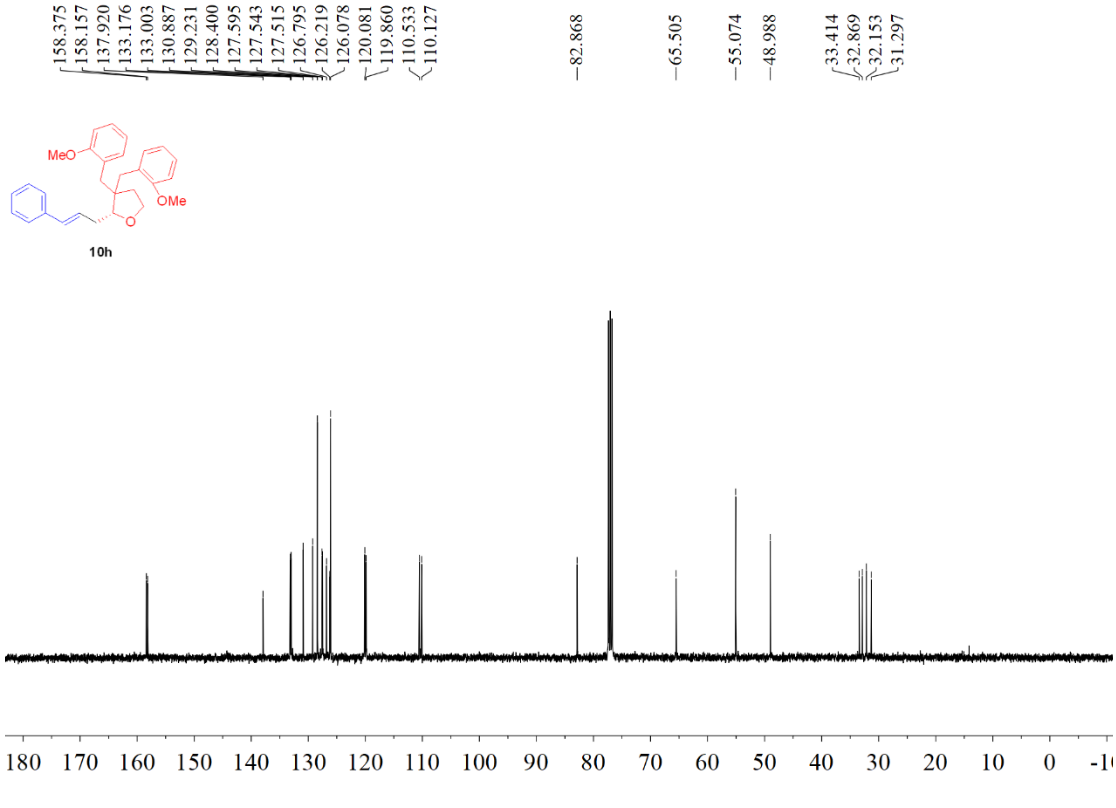
158.375 158.157 137.920 133.176 133.003 130.887 129.231 128.400 127.595 127.543 127.515 126.795 126.219 126.078 120.081 119.860 110.533 110.127
82.868
65.505
55.074
48.988
33.414 32.869 32.153 31.297
MeO
OMe
O
10h
180 170 160 150 140 130 120 110 100 90 80 70 60 50 40 30 20 10 0

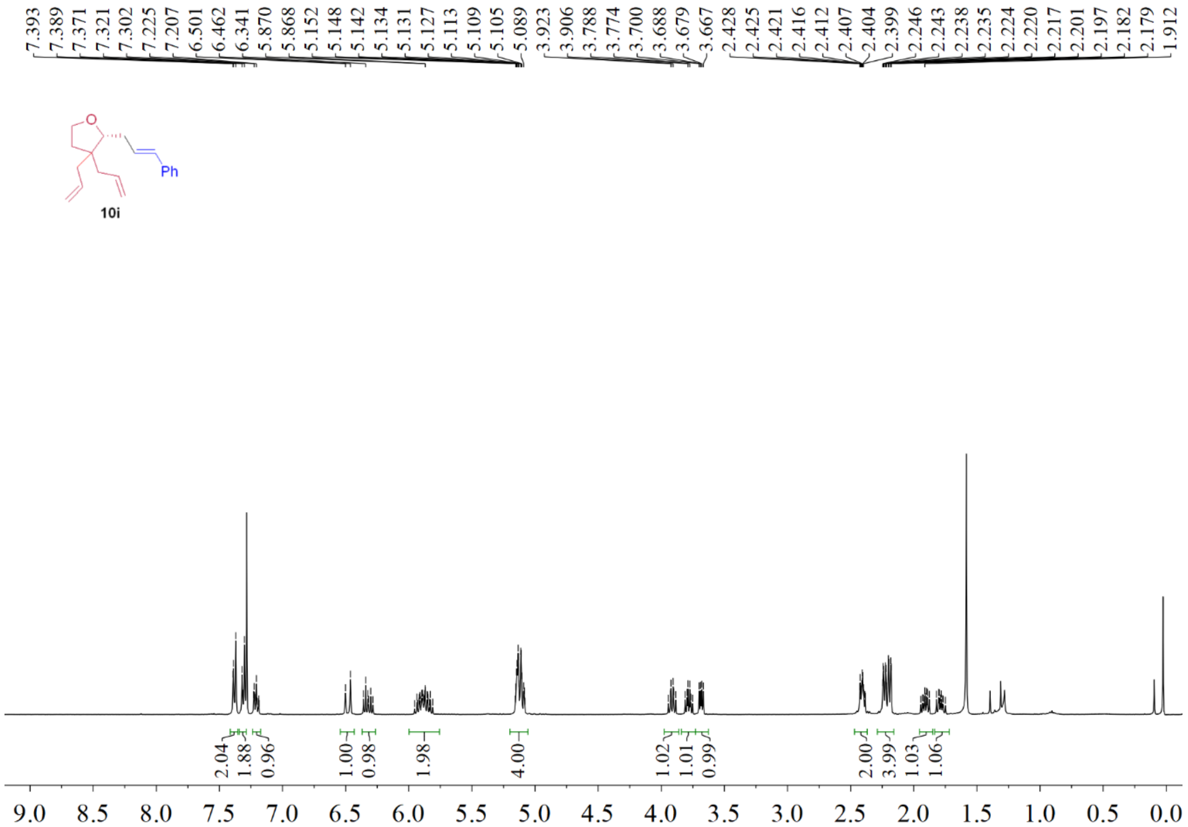
7.393 7.389 7.371 7.321 7.302 7.225 7.207 6.501 6.462 6.341 5.870 5.868 5.152 5.148 5.142 5.134 5.131 5.127 5.113 5.109 5.105 5.089 3.923 3.906 3.788 3.774 3.700 3.688 3.679 3.667 2.428 2.425 2.421 2.416 2.412 2.407 2.404 2.399 2.246 2.243 2.238 2.235 2.224 2.220 2.217 2.201 2.197 2.182 2.179 1.912
Ph
10i
2.04 1.88 0.96 1.00 0.98 1.98 4.00 1.02 1.01 0.99 2.00 3.99 1.03 1.06
9.0 8.5 8.0 7.5 7.0 6.5 6.0 5.5 5.0 4.5 4.0 3.5 3.0 2.5 2.0 1.5 1.0 0.5 0.0

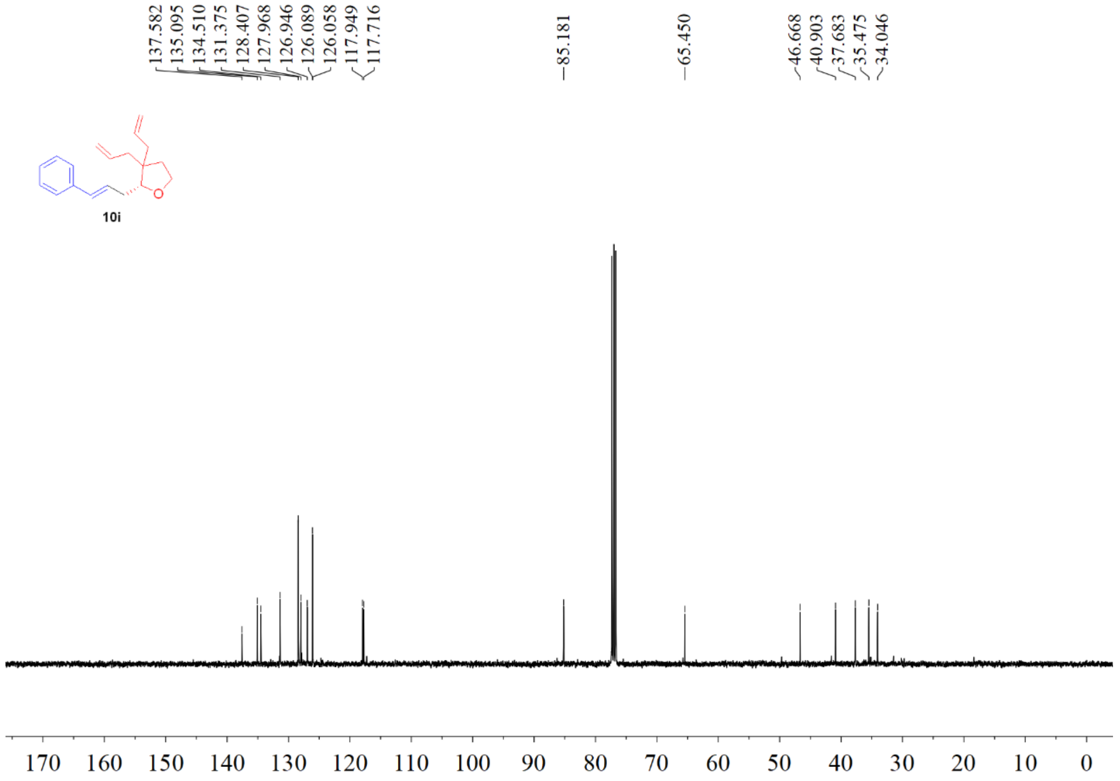
137.582 135.095 134.510 131.375 128.407 127.968 126.946 126.089 126.058 117.949 117.716
85.181
65.450
46.668 40.903 37.683 35.475 34.046
10i
170 160 150 140 130 120 110 100 90 80 70 60 50 40 30 20 10 0

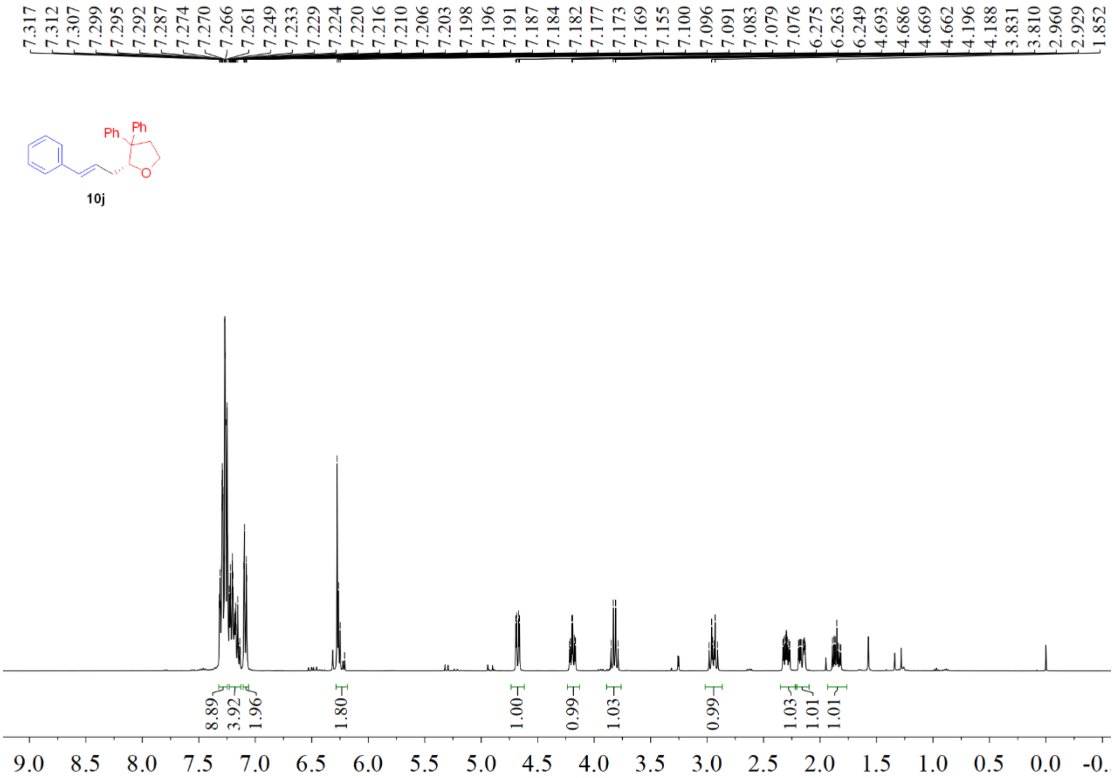
7.317
7.312
7.307
7.299
7.295
7.292
7.287
7.274
7.270
7.266
7.261
7.249
7.233
7.229
7.224
7.220
7.216
7.210
7.206
7.203
7.198
7.196
7.191
7.187
7.184
7.182
7.177
7.173
7.169
7.155
7.100
7.096
7.091
7.083
7.079
7.076
6.275
6.263
6.249
4.693
4.686
4.669
4.662
4.196
4.188
3.831
3.810
2.960
2.929
1.852
Ph
Ph
O
10j
8.89
3.92
1.96
1.80
1.00
0.99
1.03
0.99
1.03
1.01
1.01
9.0 8.5 8.0 7.5 7.0 6.5 6.0 5.5 5.0 4.5 4.0 3.5 3.0 2.5 2.0 1.5 1.0 0.5 0.0 -0.

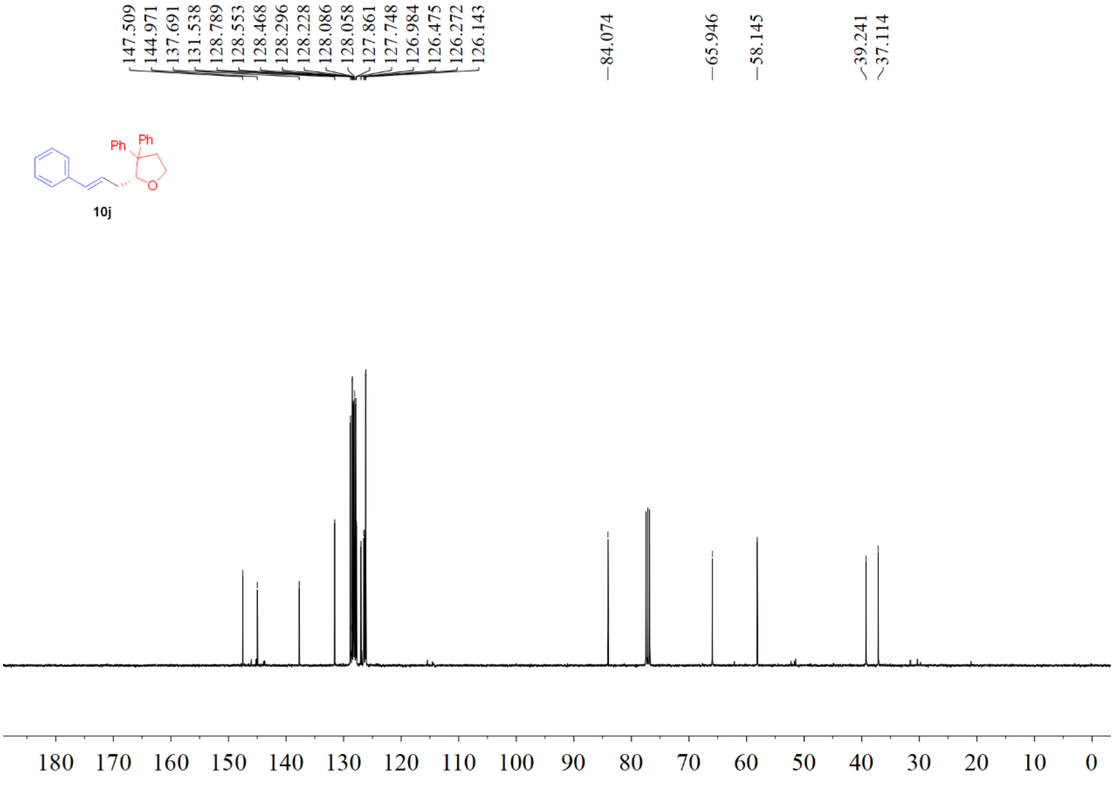
147.509
144.971
137.691
131.538
128.789
128.553
128.468
128.296
128.228
128.086
128.058
127.861
127.748
126.984
126.475
126.272
126.143
84.074
65.946
58.145
39.241
37.114
Ph
Ph
O
10j
180 170 160 150 140 130 120 110 100 90 80 70 60 50 40 30 20 10 0

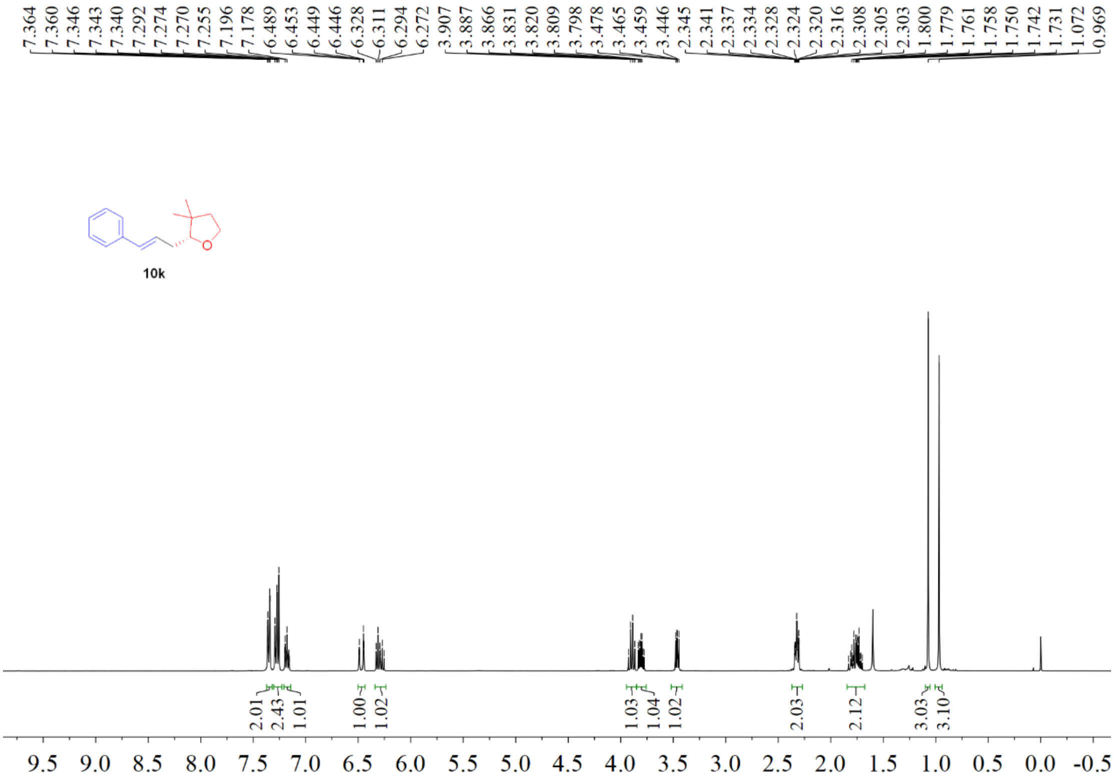
7.364
7.360
7.346
7.343
7.340
7.292
7.274
7.270
7.255
7.196
7.178
6.489
6.453
6.449
6.446
6.328
6.311
6.294
6.272
3.907
3.887
3.866
3.831
3.820
3.809
3.798
3.478
3.465
3.459
3.446
2.345
2.341
2.337
2.334
2.328
2.324
2.320
2.316
2.308
2.305
2.303
1.800
1.779
1.761
1.758
1.750
1.742
1.731
1.072
0.969
10k
2.01
2.43
1.01
1.00
1.02
1.03
1.04
1.02
2.03
2.12
3.03
3.10
9.5 9.0 8.5 8.0 7.5 7.0 6.5 6.0 5.5 5.0 4.5 4.0 3.5 3.0 2.5 2.0 1.5 1.0 0.5 0.0 -0.5

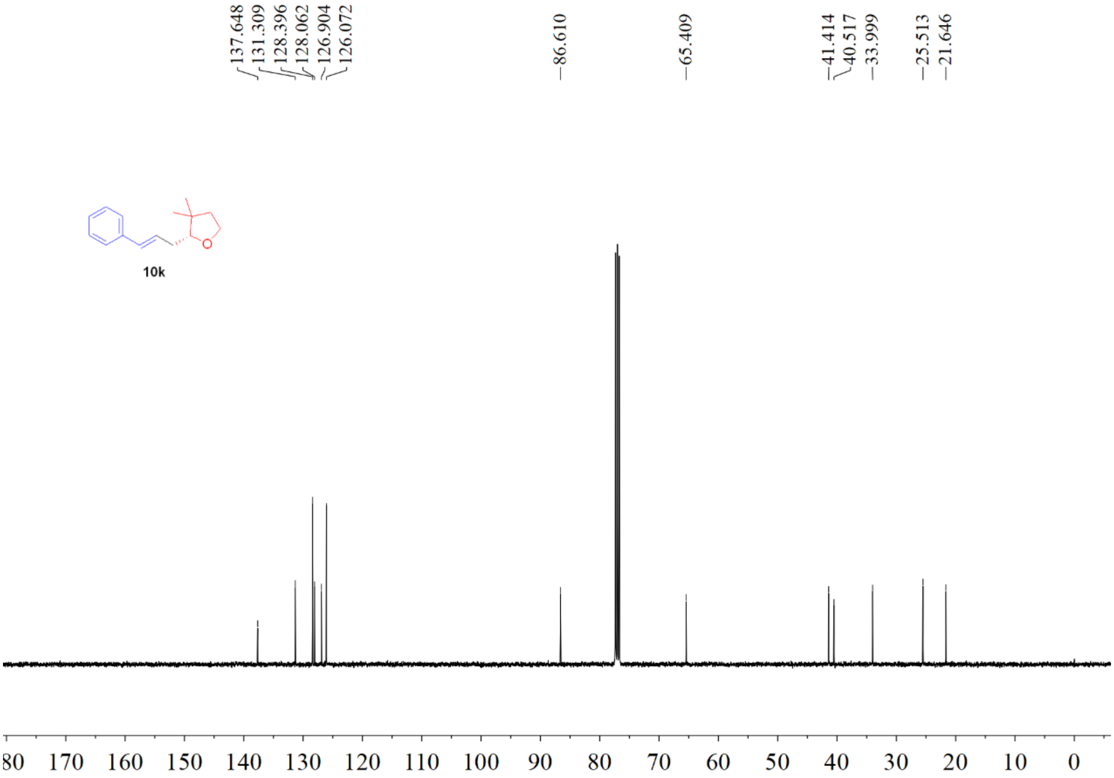
137.648
131.309
128.396
128.062
126.904
126.072
86.610
65.409
41.414
40.517
33.999
25.513
21.646
10k
80 170 160 150 140 130 120 110 100 90 80 70 60 50 40 30 20 10 0

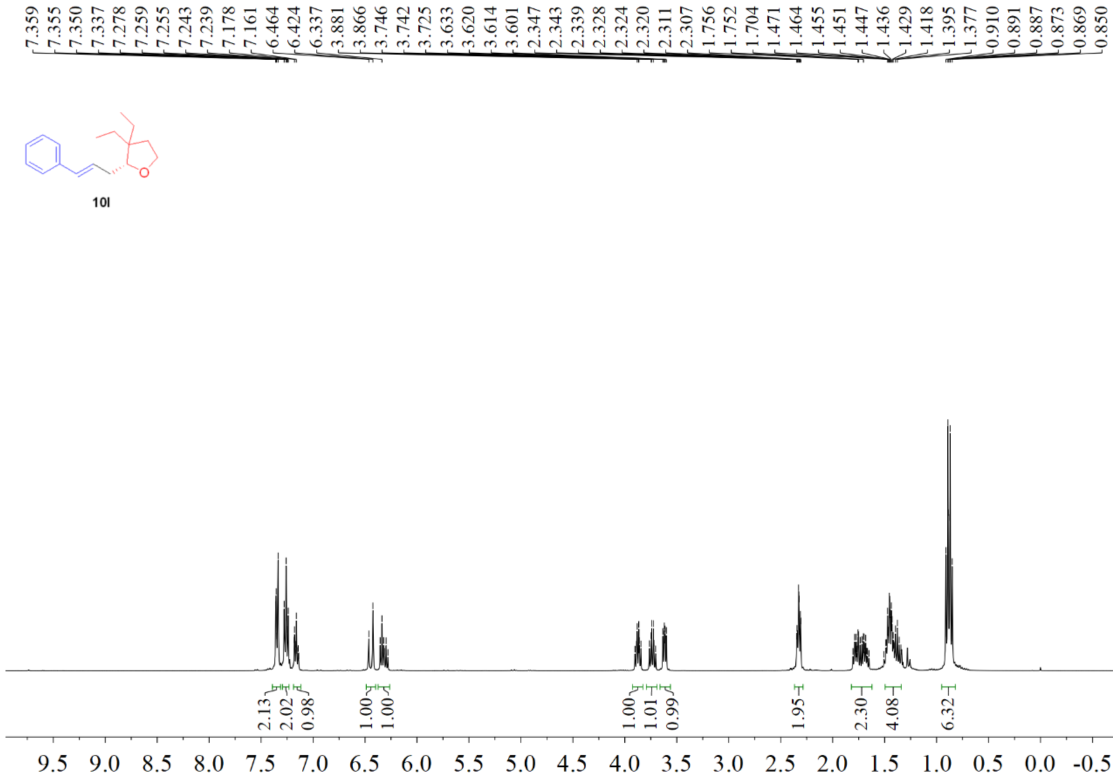
7.359
7.355
7.350
7.337
7.278
7.259
7.255
7.243
7.239
7.178
7.161
6.464
6.424
6.337
3.881
3.866
3.746
3.742
3.725
3.633
3.620
3.614
3.601
2.347
2.343
2.339
2.328
2.324
2.320
2.311
2.307
1.756
1.752
1.704
1.471
1.464
1.455
1.451
1.447
1.436
1.429
1.418
1.395
1.377
0.910
0.891
0.887
0.873
0.869
0.850
10l
2.13
2.02
0.98
1.00
1.00
1.00
1.01
0.99
1.95
2.30
4.08
6.32
9.5 9.0 8.5 8.0 7.5 7.0 6.5 6.0 5.5 5.0 4.5 4.0 3.5 3.0 2.5 2.0 1.5 1.0 0.5 0.0 -0.5

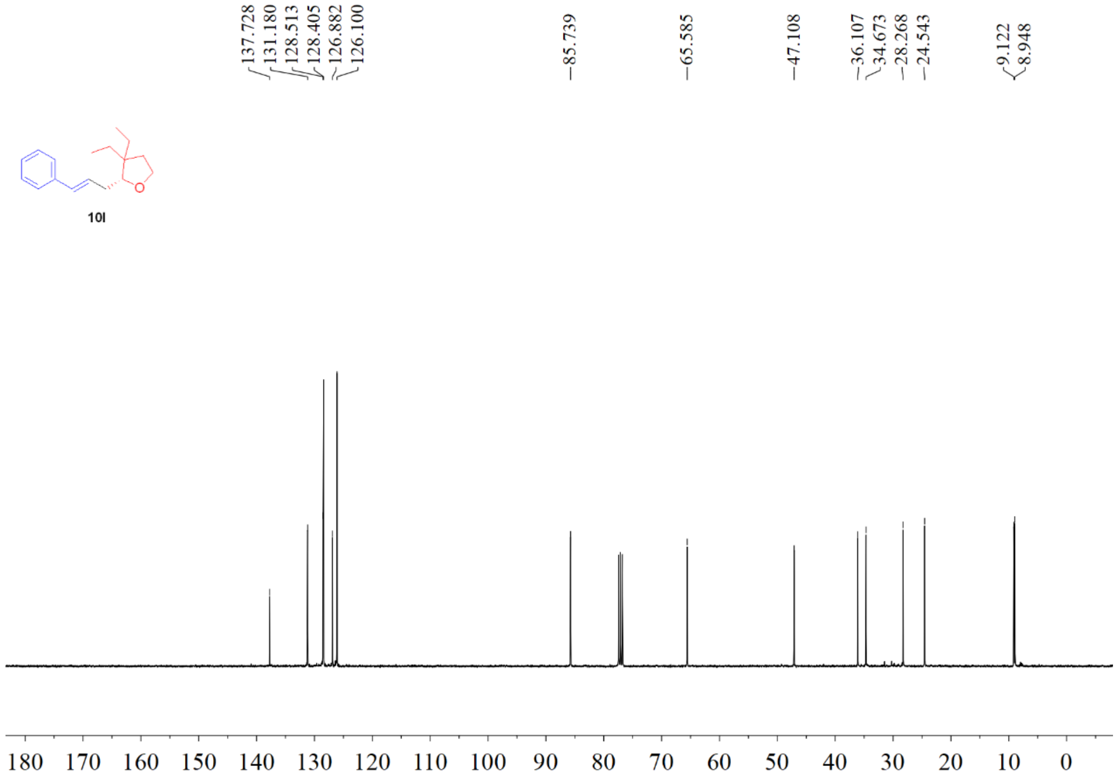
137.728
131.180
128.513
128.405
126.882
126.100
85.739
65.585
47.108
36.107
34.673
28.268
24.543
9.122
8.948
10l
180 170 160 150 140 130 120 110 100 90 80 70 60 50 40 30 20 10 0

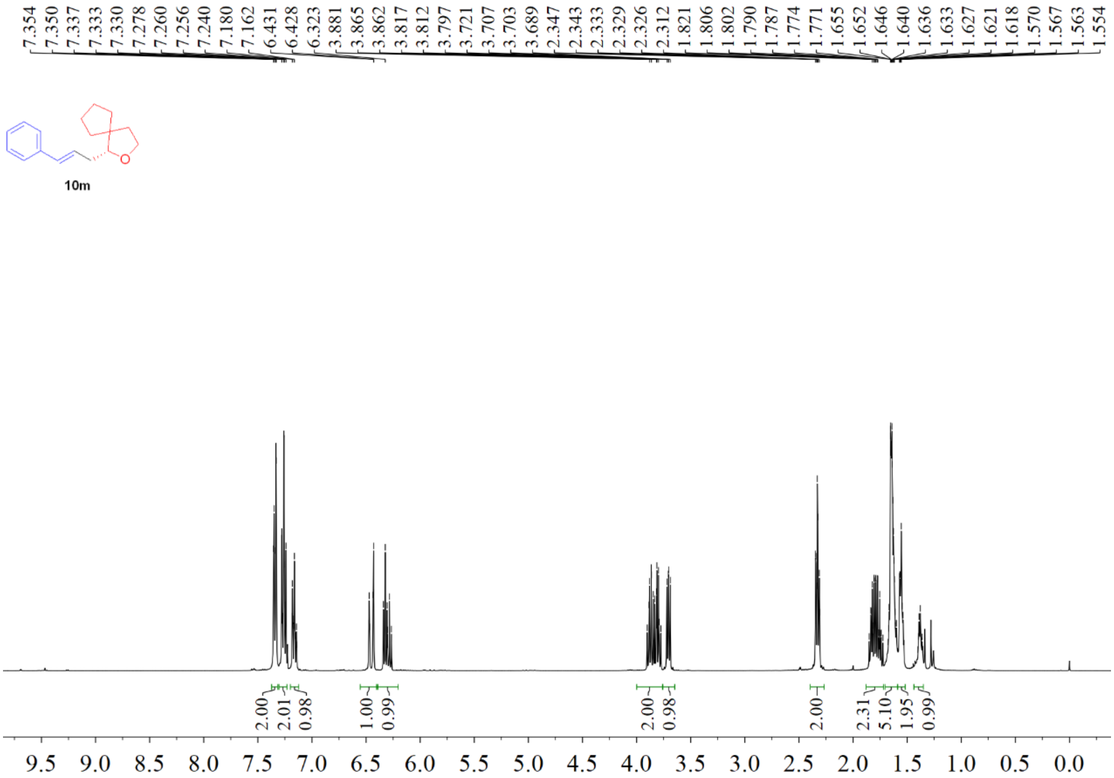
7.354 7.350 7.337 7.333 7.330 7.278 7.260 7.256 7.240 7.180 7.162 6.431 6.428 6.323 3.881 3.865 3.862 3.817 3.812 3.797 3.721 3.707 3.703 3.689 2.347 2.343 2.333 2.329 2.326 2.312 1.821 1.806 1.802 1.790 1.787 1.774 1.771 1.655 1.652 1.646 1.640 1.636 1.633 1.627 1.621 1.618 1.570 1.567 1.563 1.554
10m
2.00 2.01 0.98 1.00 0.99 2.00 0.98 2.00 2.31 5.10 1.95 0.99
9.5 9.0 8.5 8.0 7.5 7.0 6.5 6.0 5.5 5.0 4.5 4.0 3.5 3.0 2.5 2.0 1.5 1.0 0.5 0.0

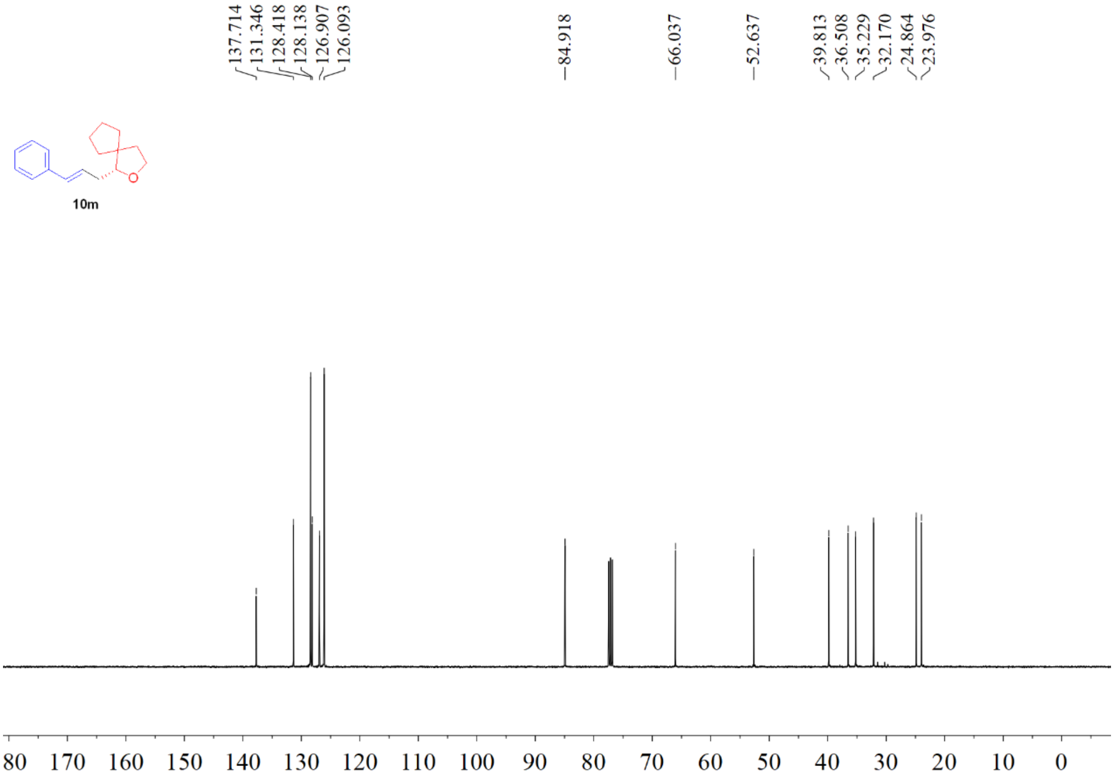
137.714 131.346 128.418 128.138 126.907 126.093
84.918
66.037
52.637
39.813 36.508 35.229 32.170 24.864 23.976
10m
80 170 160 150 140 130 120 110 100 90 80 70 60 50 40 30 20 10 0

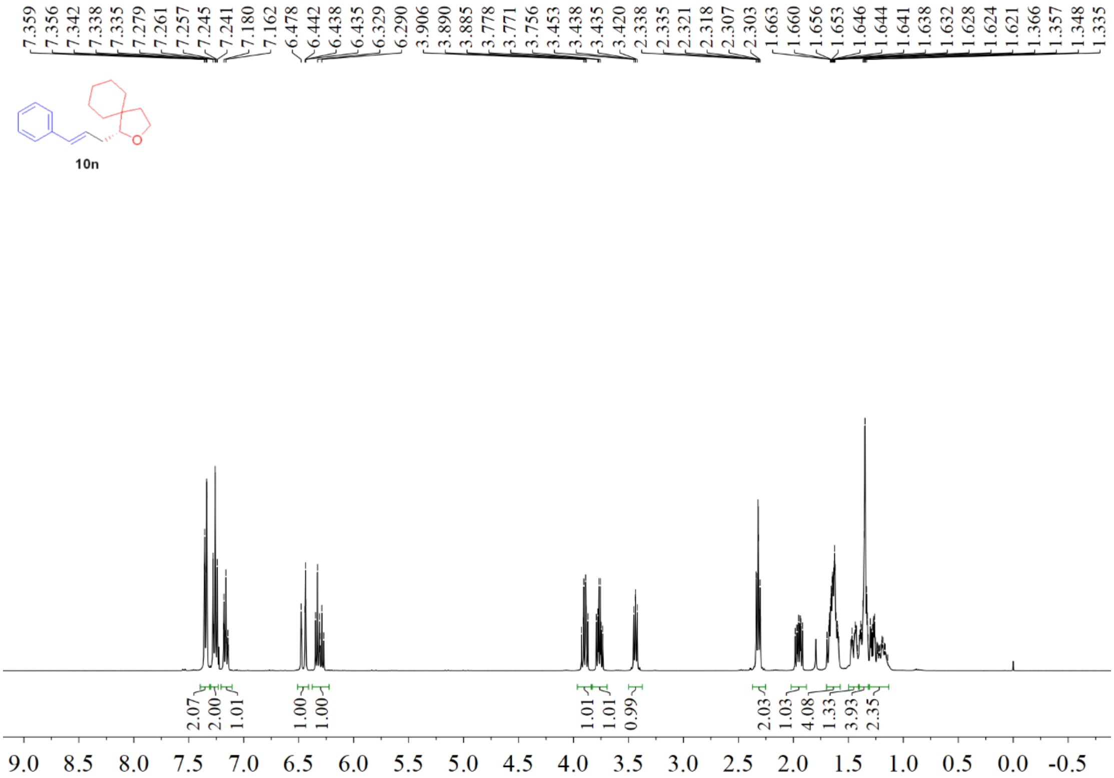
7.359
7.356
7.342
7.338
7.335
7.279
7.261
7.257
7.245
7.241
7.180
7.162
6.478
6.442
6.438
6.435
6.329
6.290
3.906
3.890
3.885
3.778
3.771
3.756
3.453
3.438
3.435
3.420
2.338
2.335
2.321
2.318
2.307
2.303
1.663
1.660
1.656
1.653
1.646
1.644
1.641
1.638
1.632
1.628
1.624
1.621
1.366
1.357
1.348
1.335
10n
2.07
2.00
1.01
1.00
1.00
1.01
1.01
0.99
2.03
1.03
4.08
1.33
3.93
2.35
9.0 8.5 8.0 7.5 7.0 6.5 6.0 5.5 5.0 4.5 4.0 3.5 3.0 2.5 2.0 1.5 1.0 0.5 0.0 -0.5

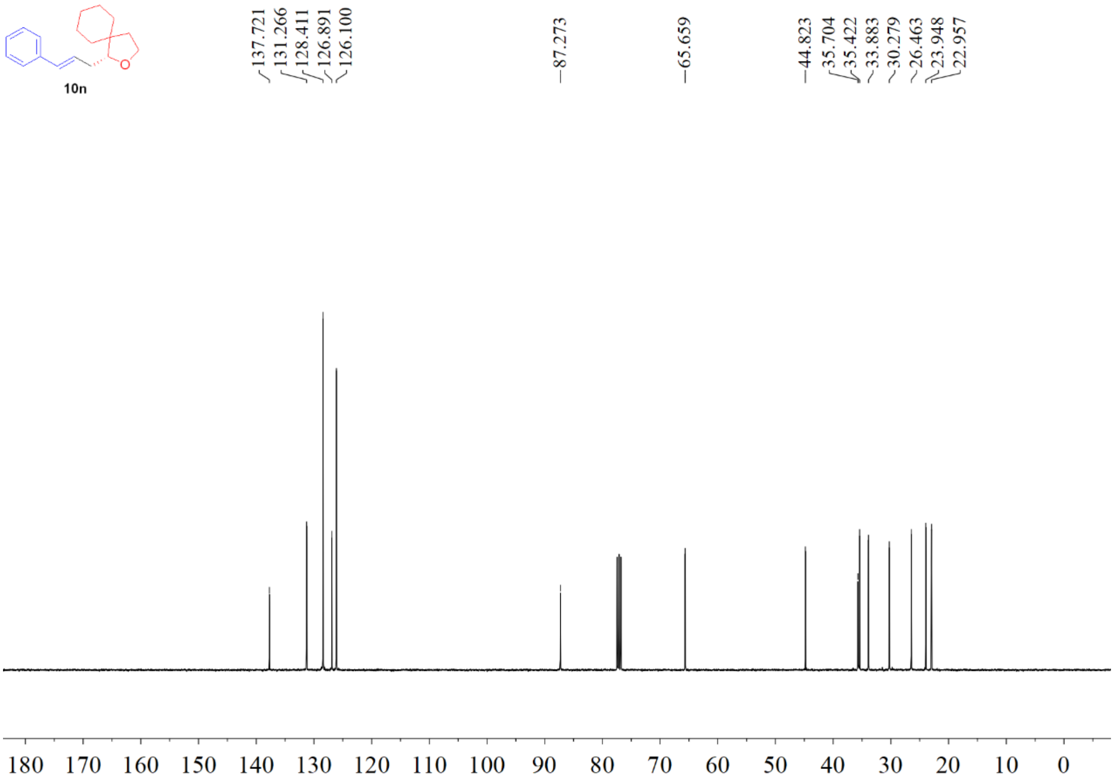
10n
137.721
131.266
128.411
126.891
126.100
87.273
65.659
44.823
35.704
35.422
33.883
30.279
26.463
23.948
22.957
180 170 160 150 140 130 120 110 100 90 80 70 60 50 40 30 20 10 0

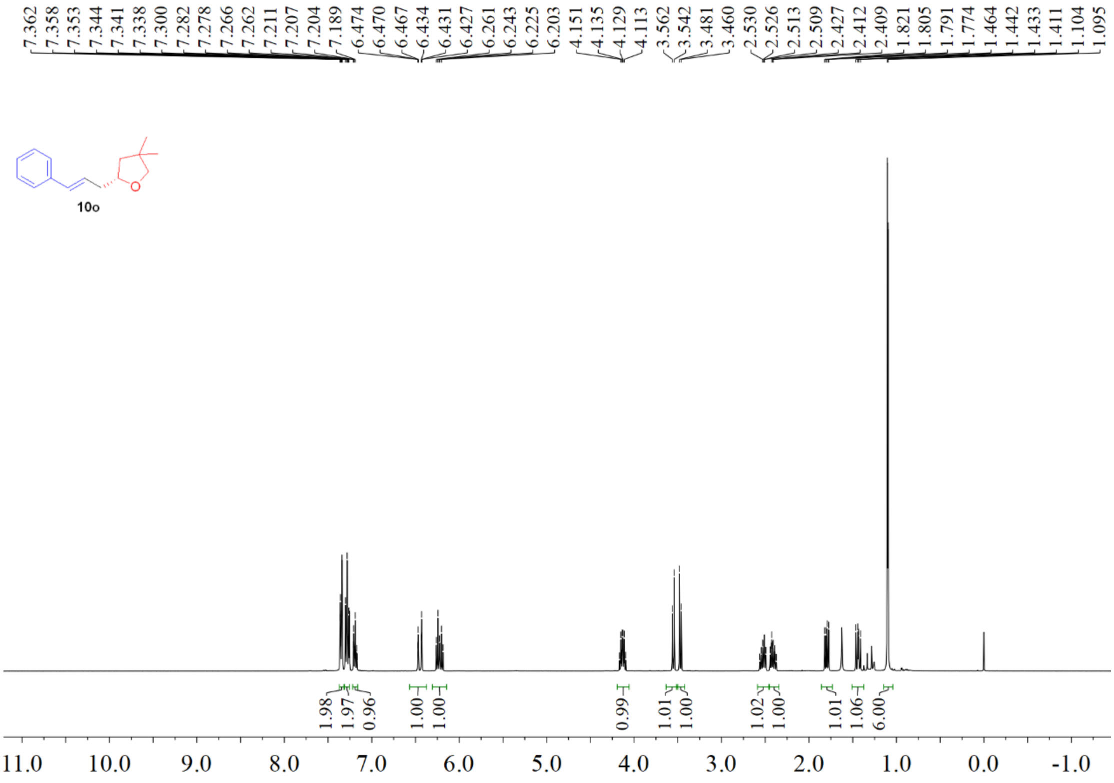
7.362
7.358
7.353
7.344
7.341
7.338
7.300
7.282
7.278
7.266
7.262
7.211
7.207
7.204
7.189
6.474
6.470
6.467
6.434
6.431
6.427
6.261
6.243
6.225
6.203
4.151
4.135
4.129
4.113
3.562
3.542
3.481
3.460
2.530
2.526
2.513
2.509
2.427
2.412
2.409
1.821
1.805
1.791
1.774
1.464
1.442
1.433
1.411
1.104
1.095
10o
1.98
1.97
0.96
1.00
1.00
0.99
1.01
1.00
1.02
1.00
1.01
1.06
6.00
11.0 10.0 9.0 8.0 7.0 6.0 5.0 4.0 3.0 2.0 1.0 0.0 -1.0
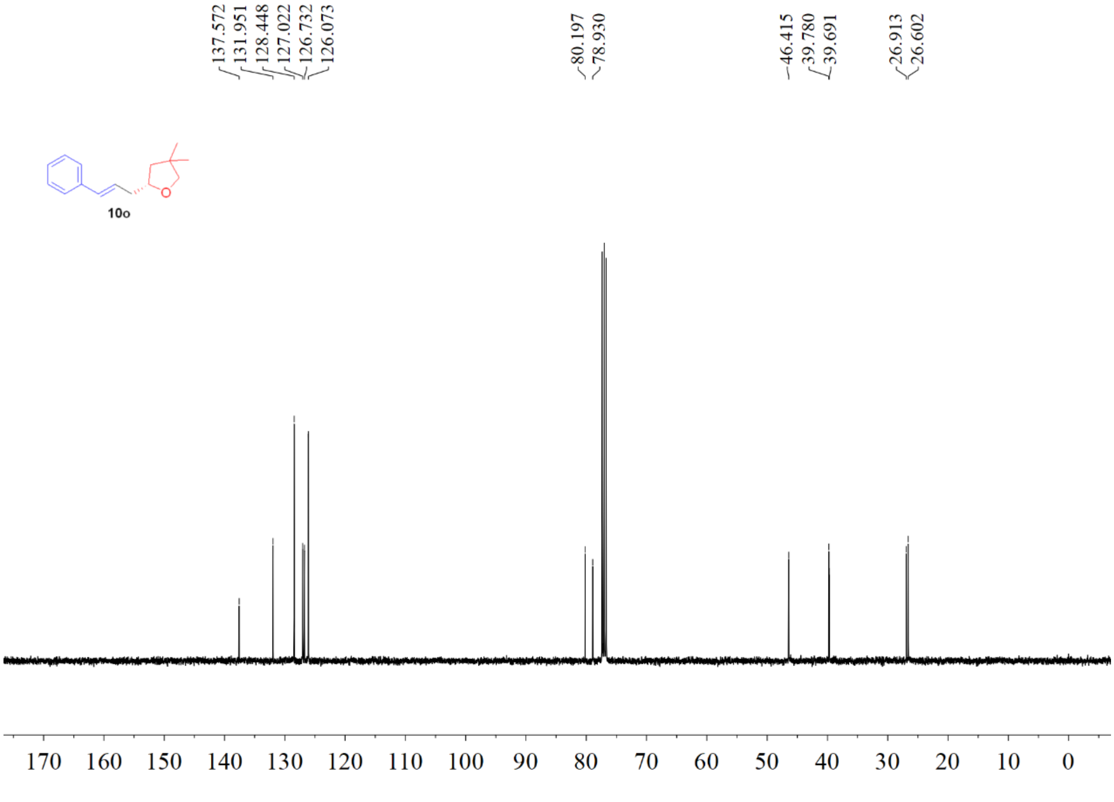
137.572
131.951
128.448
127.022
126.732
126.073
80.197
78.930
46.415
39.780
39.691
26.913
26.602
10o
170 160 150 140 130 120 110 100 90 80 70 60 50 40 30 20 10 0

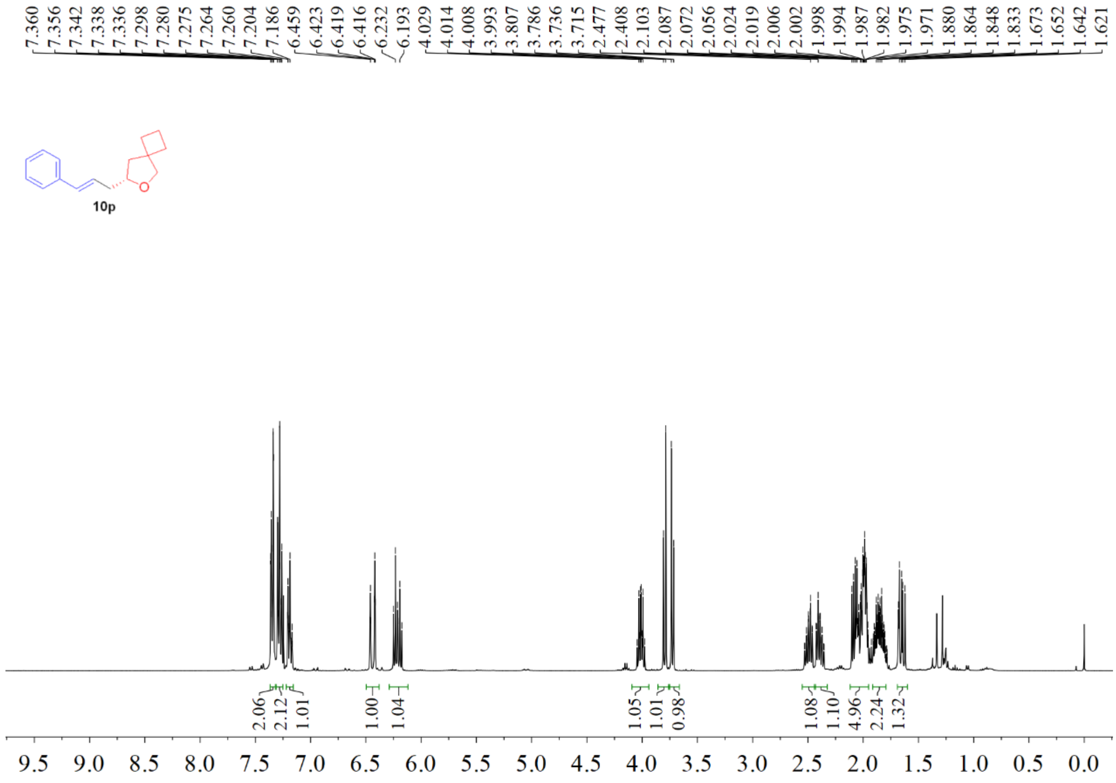
7.360 7.356 7.342 7.338 7.336 7.298 7.280 7.275 7.264 7.260 7.204 7.186 6.459 6.423 6.419 6.416 6.232 6.193 4.029 4.014 4.008 3.993 3.807 3.786 3.736 3.715 2.477 2.408 2.103 2.087 2.072 2.056 2.024 2.019 2.006 2.002 1.998 1.994 1.987 1.982 1.975 1.971 1.880 1.864 1.848 1.833 1.673 1.652 1.642 1.621
10p
2.06 2.12 1.01 1.00 1.04 1.05 1.01 0.98 1.08 1.10 4.96 2.24 1.32
9.5 9.0 8.5 8.0 7.5 7.0 6.5 6.0 5.5 5.0 4.5 4.0 3.5 3.0 2.5 2.0 1.5 1.0 0.5 0.0

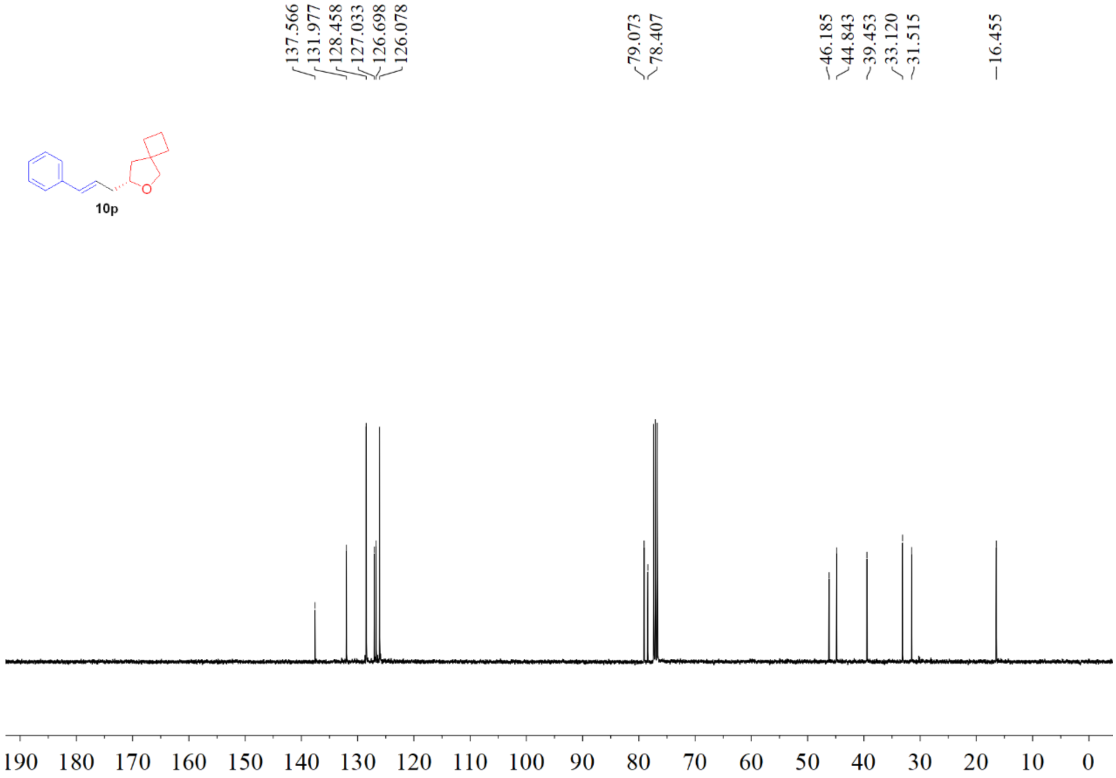
137.566 131.977 128.458 127.033 126.698 126.078 79.073 78.407 46.185 44.843 39.453 33.120 31.515 16.455
10p
190 180 170 160 150 140 130 120 110 100 90 80 70 60 50 40 30 20 10 0

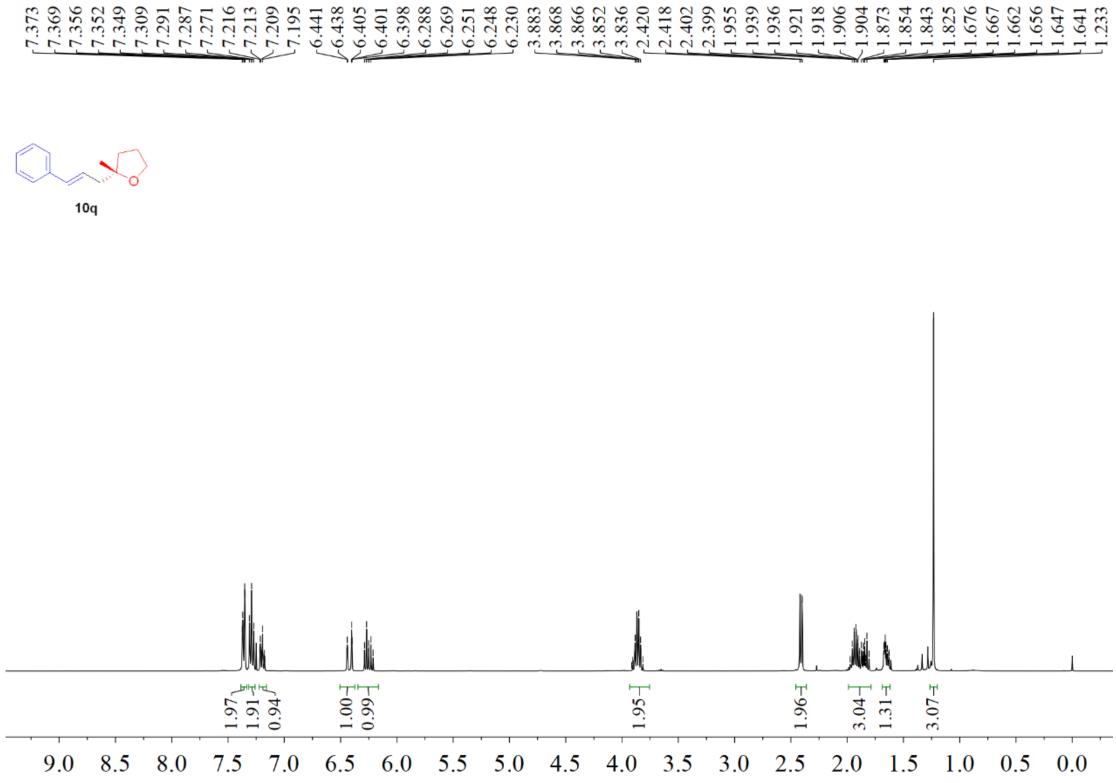
7.373
7.369
7.356
7.352
7.349
7.309
7.291
7.287
7.271
7.216
7.213
7.209
7.195
6.441
6.438
6.405
6.401
6.398
6.288
6.269
6.251
6.248
6.230
3.883
3.868
3.866
3.852
3.836
2.420
2.418
2.402
2.399
1.955
1.939
1.936
1.921
1.918
1.906
1.904
1.873
1.854
1.843
1.825
1.676
1.667
1.662
1.656
1.647
1.641
1.233
O
10q
1.97
1.91
0.94
1.00
0.99
1.95
1.96
3.04
1.31
3.07
9.0 8.5 8.0 7.5 7.0 6.5 6.0 5.5 5.0 4.5 4.0 3.5 3.0 2.5 2.0 1.5 1.0 0.5 0.0

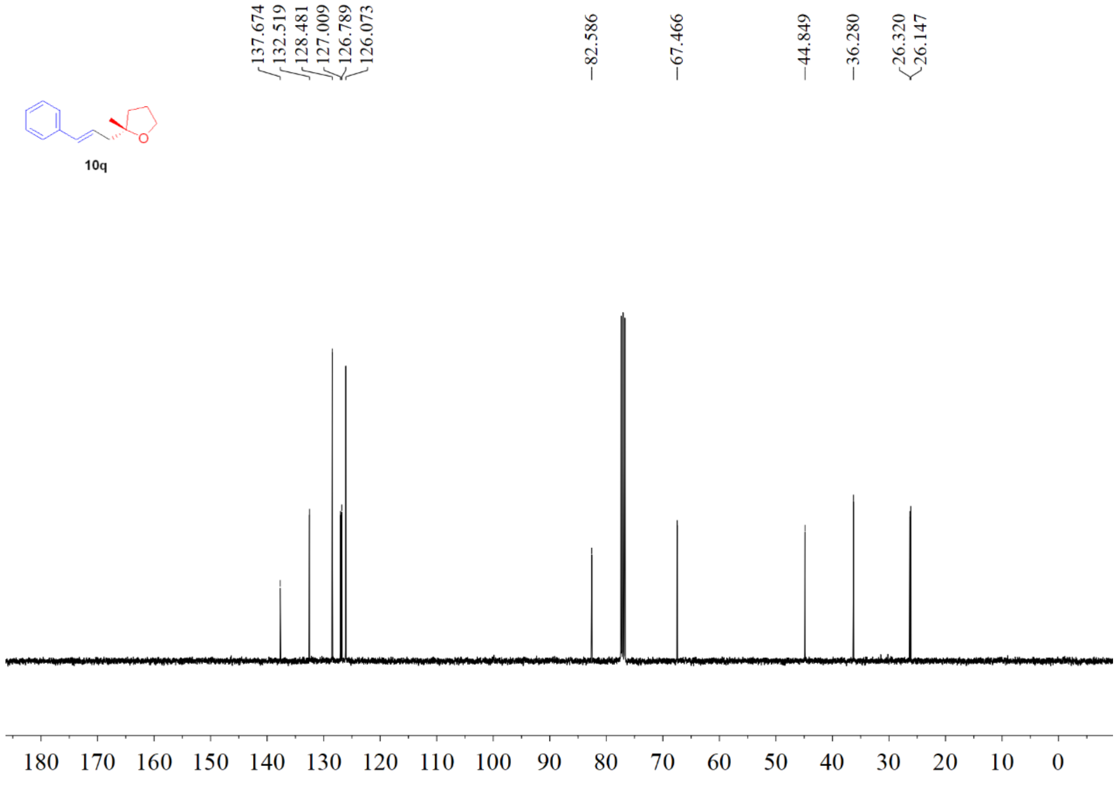
137.674
132.519
128.481
127.009
126.789
126.073
82.586
67.466
44.849
36.280
26.320
26.147
O
10q
180 170 160 150 140 130 120 110 100 90 80 70 60 50 40 30 20 10 0

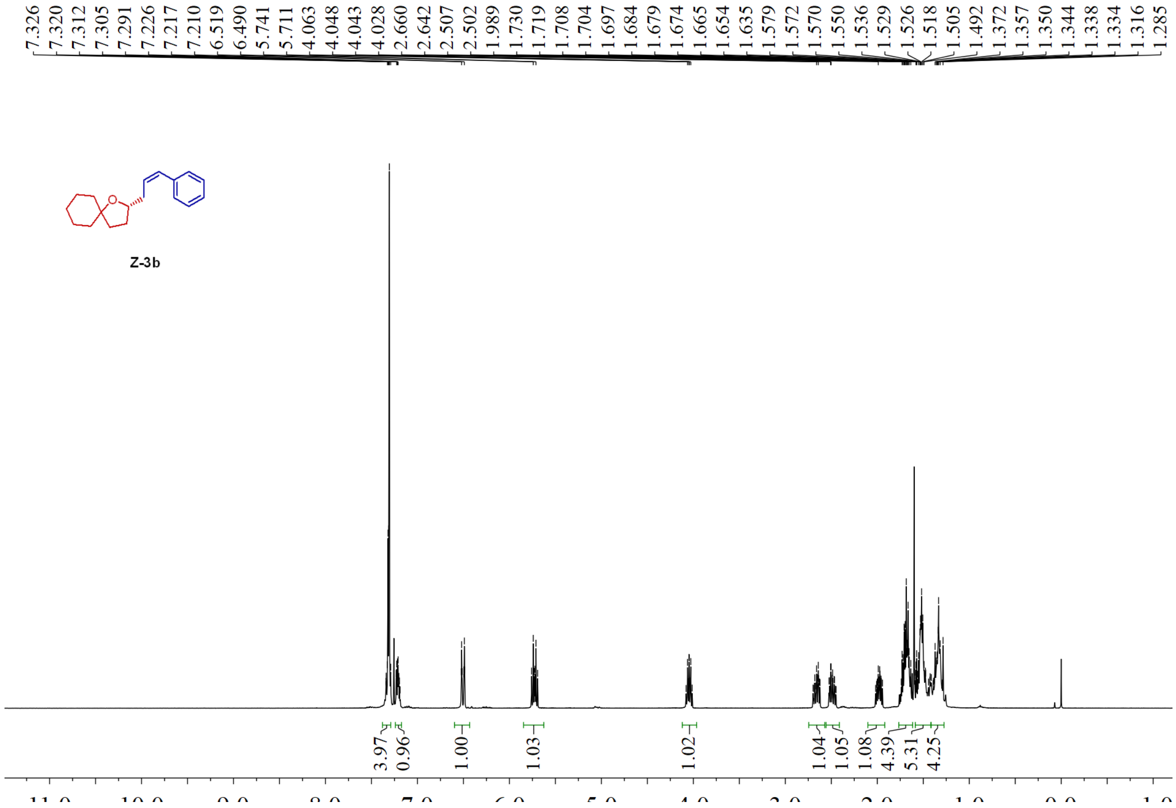
7.326
7.320
7.312
7.305
7.291
7.226
7.217
7.210
6.519
6.490
5.741
5.711
4.063
4.048
4.043
4.028
2.660
2.642
2.507
2.502
1.989
1.730
1.719
1.708
1.704
1.697
1.684
1.679
1.674
1.665
1.654
1.635
1.579
1.572
1.570
1.550
1.536
1.529
1.526
1.518
1.505
1.492
1.372
1.357
1.350
1.344
1.338
1.334
1.316
1.285
Z-3b
3.97
0.96
1.00
1.03
1.02
1.04
1.05
1.08
4.39
5.31
4.25

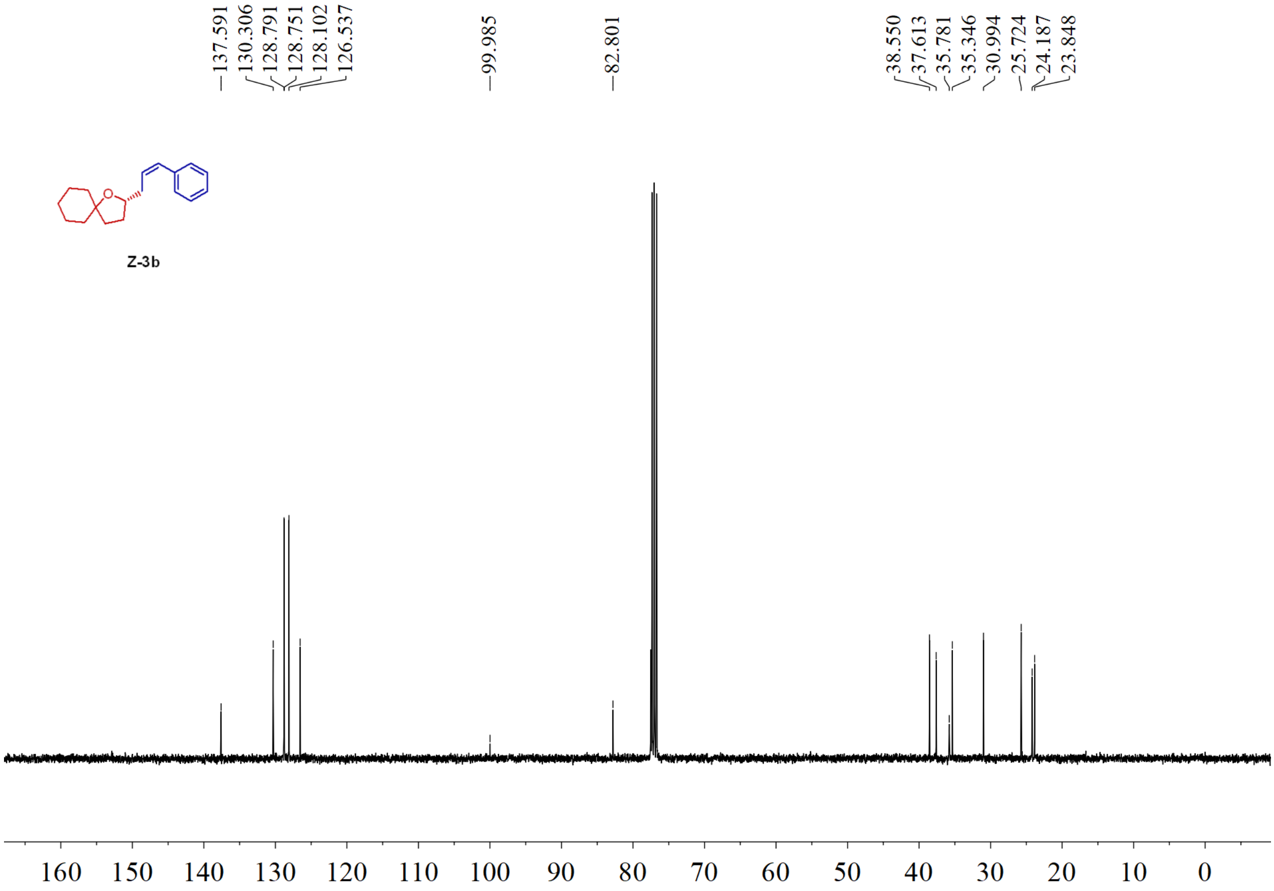
137.591
130.306
128.791
128.751
128.102
126.537
99.985
82.801
38.550
37.613
35.781
35.346
30.994
25.724
24.187
23.848
Z-3b
160 150 140 130 120 110 100 90 80 70 60 50 40 30 20 10 0

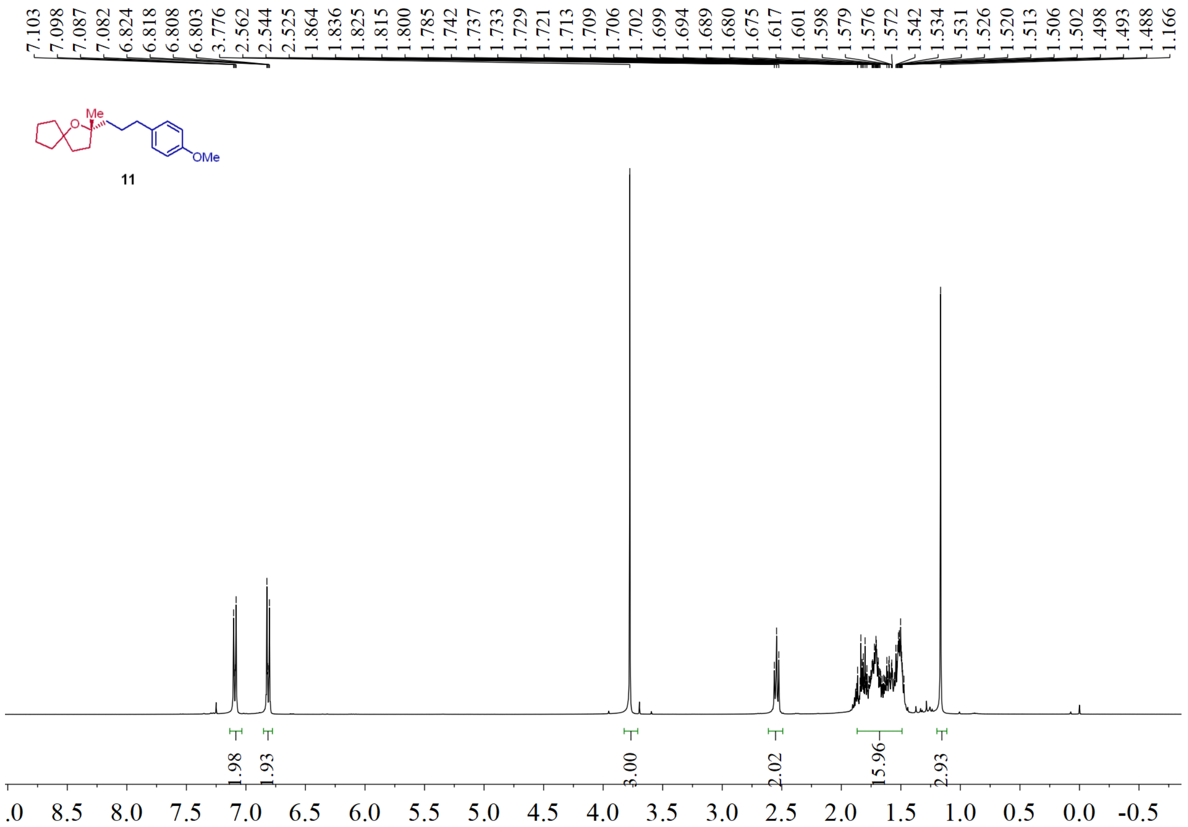
7.103
7.098
7.087
7.082
6.824
6.818
6.808
6.803
3.776
2.562
2.544
2.525
1.864
1.836
1.825
1.815
1.800
1.785
1.742
1.737
1.733
1.729
1.721
1.713
1.709
1.706
1.702
1.699
1.694
1.689
1.680
1.675
1.617
1.601
1.598
1.579
1.576
1.572
1.542
1.534
1.531
1.526
1.520
1.513
1.506
1.502
1.498
1.493
1.488
1.166
Me
O
OMe
11
1.98
1.93
3.00
2.02
15.96
2.93
.0 8.5 8.0 7.5 7.0 6.5 6.0 5.5 5.0 4.5 4.0 3.5 3.0 2.5 2.0 1.5 1.0 0.5 0.0 -0.5

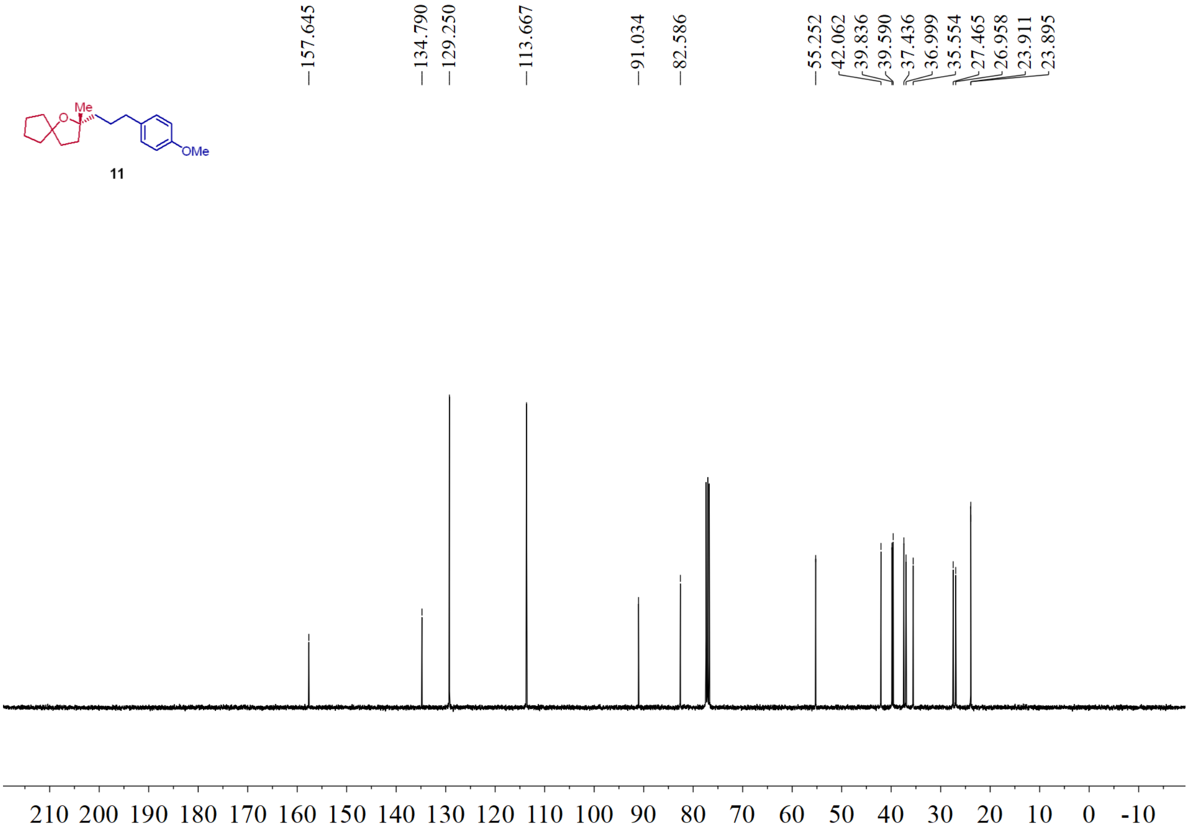
157.645
134.790
129.250
113.667
91.034
82.586
55.252
42.062
39.836
39.590
37.436
36.999
35.554
27.465
26.958
23.911
23.895
Me
O
OMe
11
210 200 190 180 170 160 150 140 130 120 110 100 90 80 70 60 50 40 30 20 10 0 -10

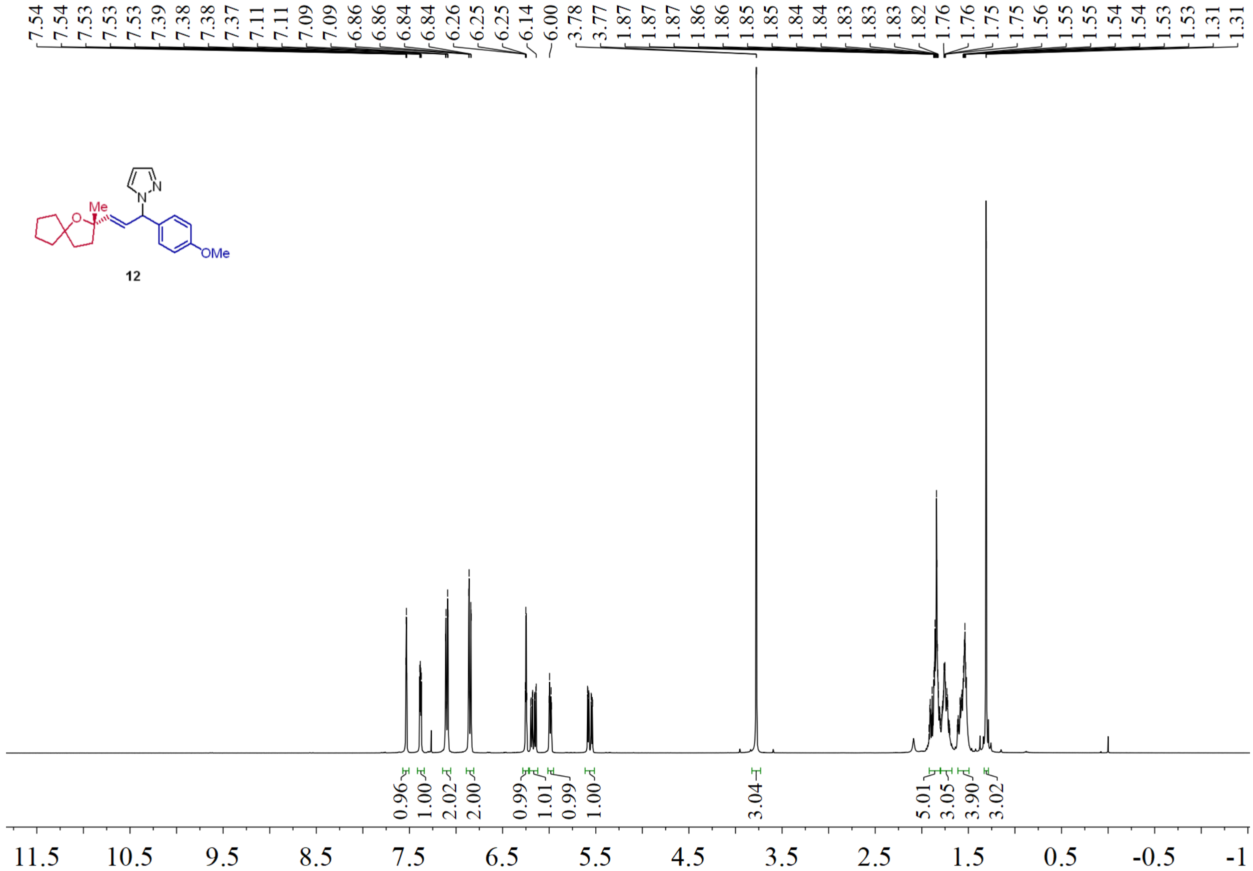

7.54
7.54
7.53
7.53
7.53
7.39
7.38
7.38
7.37
7.11
7.11
7.09
7.09
6.86
6.86
6.84
6.84
6.26
6.25
6.25
6.14
6.00
3.78
3.77
1.87
1.87
1.87
1.86
1.86
1.85
1.85
1.84
1.84
1.83
1.83
1.83
1.82
1.76
1.76
1.75
1.75
1.56
1.55
1.55
1.54
1.54
1.53
1.53
1.31
1.31
Me
O
N
N
OMe
12
0.96
1.00
2.02
2.00
0.99
1.01
0.99
1.00
3.04
5.01
3.05
3.90
3.02
11.5
10.5
9.5
8.5
7.5
6.5
5.5
4.5
3.5
2.5
1.5
0.5
-0.5
-1

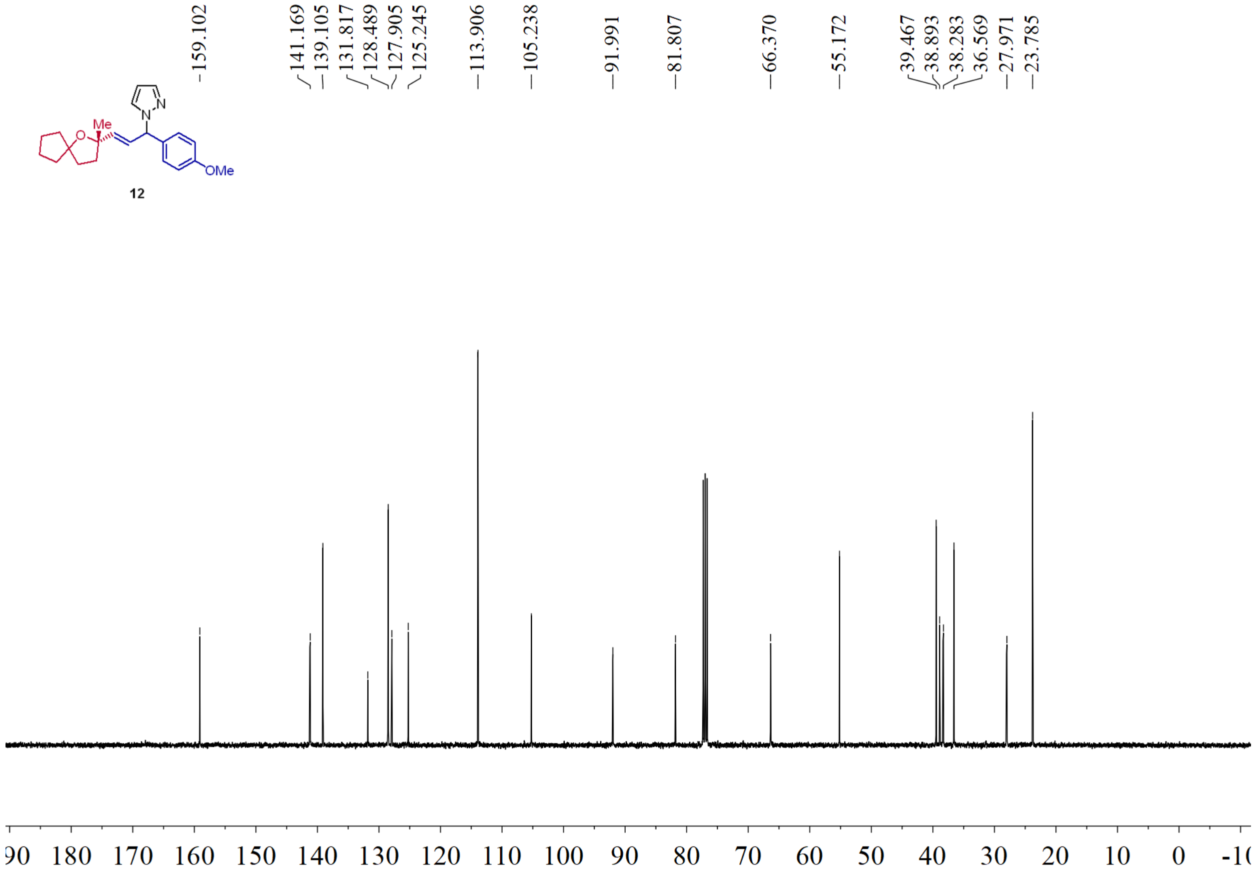

159.102
141.169
139.105
131.817
128.489
127.905
125.245
113.906
105.238
91.991
81.807
66.370
55.172
39.467
38.893
38.283
36.569
27.971
23.785
Me
O
N
N
OMe
12
180
170
160
150
140
130
120
110
100
90
80
70
60
50
40
30
20
10
0

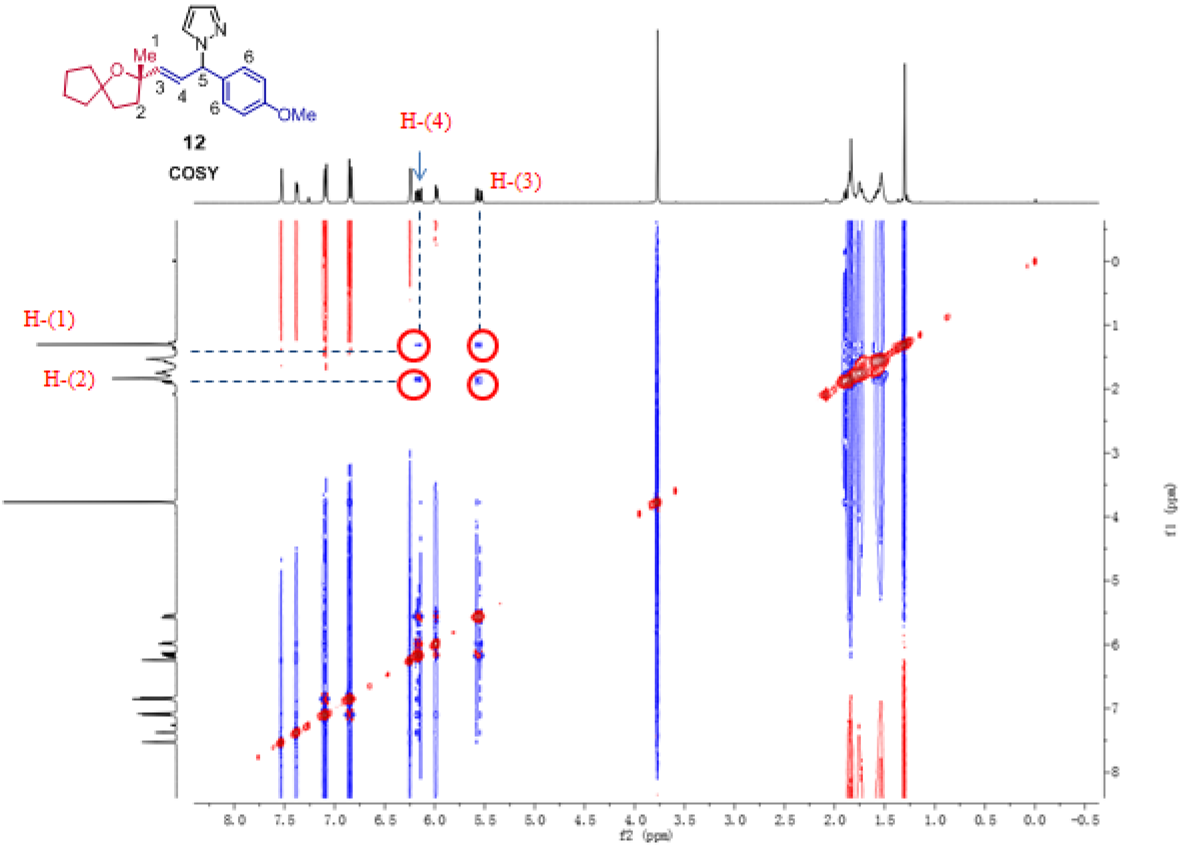

Me
1
3
4
5
2
6
6
OMe
12
COSY
H-(4)
H-(3)
H-(1)
H-(2)
f2 (ppm)
f1 (ppm)

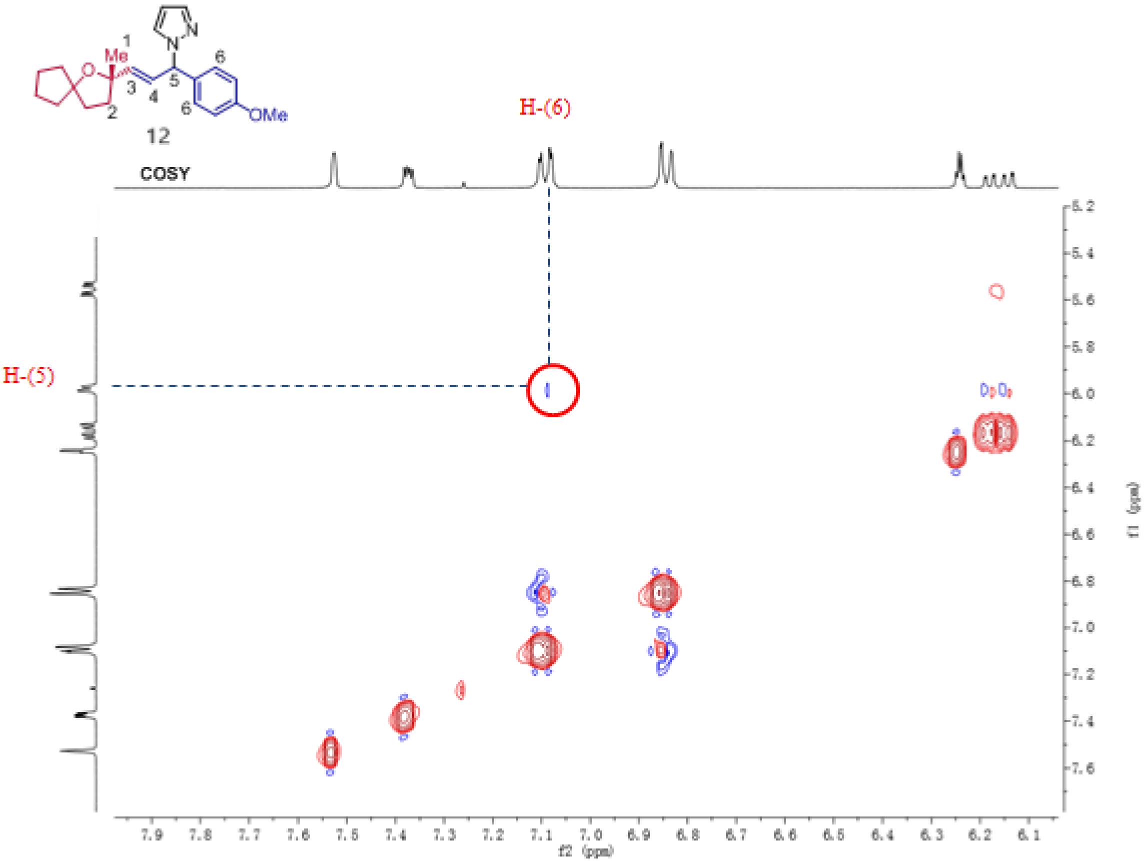

12
COSY
H-(6)
H-(5)
f2 (ppm)
f1 (ppm)

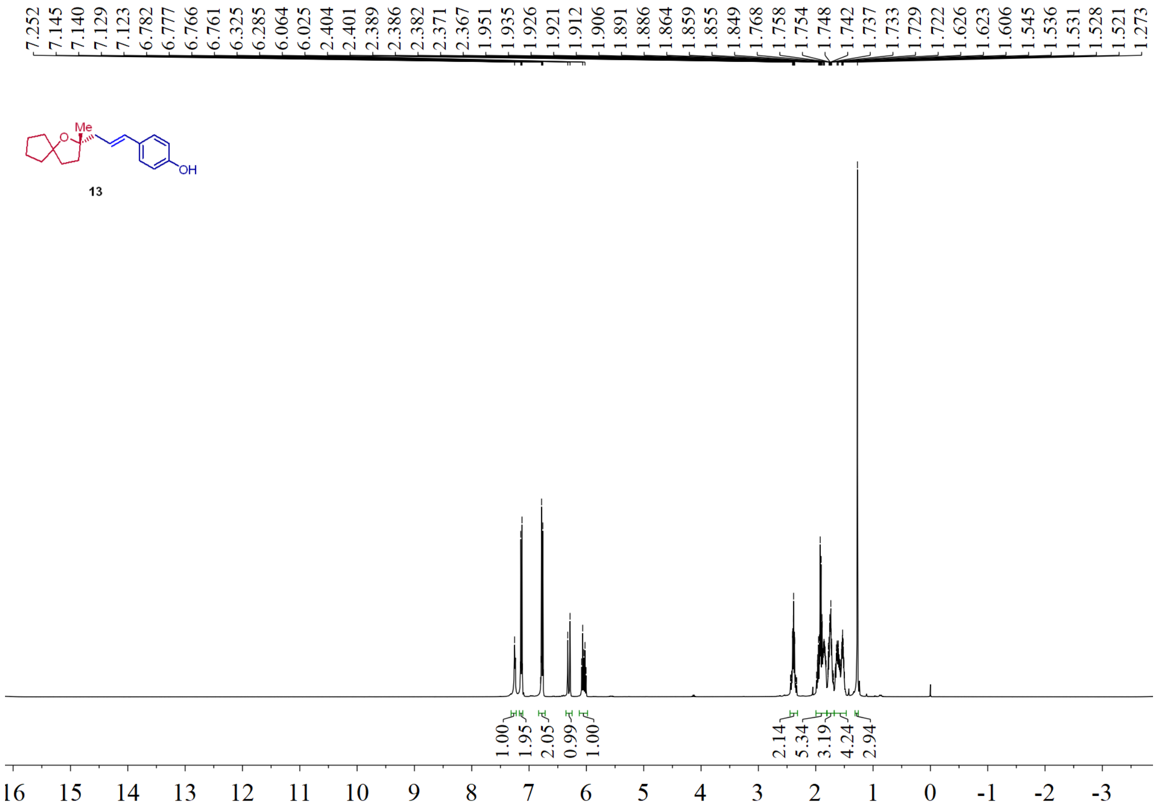

7.252
7.145
7.140
7.129
7.123
6.782
6.777
6.766
6.761
6.325
6.285
6.064
6.025
2.404
2.401
2.389
2.386
2.382
2.371
2.367
1.951
1.935
1.926
1.921
1.912
1.906
1.891
1.886
1.864
1.859
1.855
1.849
1.768
1.758
1.754
1.748
1.742
1.737
1.733
1.729
1.722
1.626
1.623
1.606
1.545
1.536
1.531
1.528
1.521
1.273
13
1.00
1.95
2.05
0.99
1.00
2.14
5.34
3.19
4.24
2.94

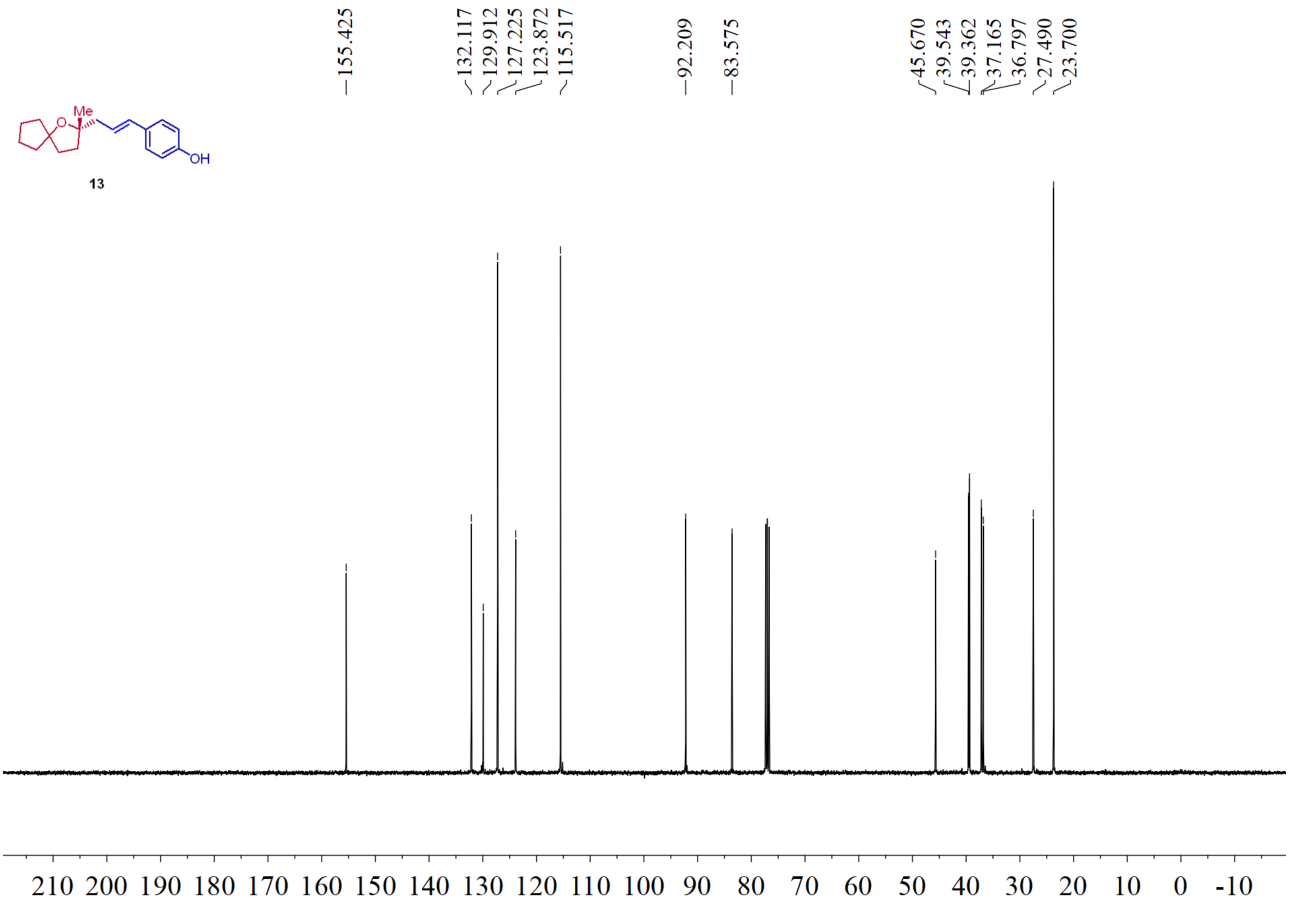

9. References

1. Zhong, M., Jiang, Y., Chen, Y., Yan, Q., Liu, J. & Di, D. *Tetrahedron: Asymmetry* **26**, 1145-1149 (2015).
2. Nishiyama, T., Woodhall, J. F., Lawson, E. N. & Kitching, W., *J. Org. Chem.* **54**, 2183-2189 (1989).
3. Miao, L., Haque, I., Manzoni, M. R., Tham, W. S. & Chemler, S. R. *Org. Lett.* **12**, 4739 (2010).
4. McCourt, R. O., Dénès, F., Sanchez-Sanz, G. & Scanlan, E. M. *Org. Lett.* **20**, 2948 (2018).
5. Bhunya, R. Jana, N., Das, T. & Nanda, S. *Synlett* **8**, 1237 (2009).
6. Escudero, J., Bellosta, V. & Cossy J. *Angew. Chem. Int. Ed.* **57**, 574 (2018).
7. Dong, Y. & Breit, B. *Org. Lett.* **23**, 6765 (2021).
8. Zhang, D. P., Fan, Y. J., Chen, H., Trpout, S. & Li, M.H. *Angew.Chem. Int. Ed.* **58**,10260 –10265 (2019).
9. Tao, Y. Q., He, F. K., Jin, K. K., Wang, J. J., Wang, Y. Q., Zhou, J. F., Sun, J. & Fang, Q. *Polym. Chem.*, **8**, 2010–2015 (2017).
10. Gaussian 09, Revision E.01, Frisch, M. J., Trucks, G. W., Schlegel, H. B., Scuseria, G. E., Robb, M. A., Cheeseman, J. R., Scalmani, G., Barone, V., Mennucci, B., Petersson, G. A., Na-katsuji, H., Caricato, M., Li, X., Hratchian, H. P., Izmaylov, A. F., Bloino, J., Zheng, G., Son-nenberg, J. L., Hada, M., Ehara, M., Toyota, K., Fukuda, R., Hasegawa, J., Ishida, M., Nakajima, T., Honda, Y., Kitao, Nakai, O.H., Vreven, T., Montgomery, J. A., Peralta, Jr., J. E.，Ogliaro, F., Bearpark, M., Heyd, J. J., Brothers, E., Kudin, K. N., Staroverov, V. N., Keith, T., Kobayashi, R., Normand, J., Raghavachari, K., Rendell, A., Burant, J. C., Iyengar, S. S., Tomasi, J., Cossi, M., Rega, N., Millam, J. M., Klene, M., Knox, J. E., Cross, J. B., Bakken, V. , Adamo, C., Jaramillo, J., Gomperts, R., Stratmann, R. E., Yazyev, O., Austin, A. J., Cammi, R., Pomelli, C., Ochterski, J. W., Martin, R. L., Morokuma, K., Zakrzewski, V. G., Voth, G. A., Salvador, P.,

Dannenberg, J. J., Dapprich, S., Daniels, A. D., Farkas, O., Foresman, J. B., Ortiz, J. V., Cioslowski, J. & Fox, D. J., Gaussian, Inc., Wallingford CT, 2013.

11. Adamo, C. & Barone, V. Toward reliable density functional methods without adjustable parameters: The PBE0 model. *J. Chem. Phys*. **110**, 6158 (1999).
12. Grimme, S., Antony, J., Ehrlich, S. & Krieg, H. A Consistent and Accurate Ab Initio Parametrization of Density Functional Dispersion Correction (DFT-D) for the 94 Elements H-Pu. *J. Chem. Phys*. **132**, 154104 (2010).
13. Grimme, S., Ehrlich, S. & Goerigk, L. Effect of the Damping Function in Dispersion Corrected Density Functional Theory. *J. Comput. Chem.* **32**, 1456–1465 (2011).
14. Dunning Jr., T. H. & Hay, P. J. in Modern Theoretical Chemistry, Ed. H. F. Schaefer III, Vol. 3 (Plenum, New York), **1977**, 1–28.
15. Hariharan, P. C. & Pople, J. A. Accuracy of AH Nequilibrium Geometries by Single Determinant Molecular Orbital Theory. *Molecular Physics.* **27**, 209–214 (1974).
16. Petersson, G. A., Bennett, A., Tensfeldt, T. G., Laham, Al, M. A., Shirley, W. A. & Mantzaris, J. A Complete Basis Set Model Chemistry. I. the Total Energies of Closed-Shell Atoms and Hydrides of the First-Row Elements. *J. Chem. Phys.* **89**, 2193–2218 (1988).
17. Gonzalez, C., Schlegel HBAn improved algorithm for reaction-path following. *J. Chem. Phys.* **90**, 2154–2161 (1989).
18. Gonzalez, C., Schlegel HB Reaction-path following in massweighted internal coordinates. *J. Org. Chem.* **94**, 5523–5527 (1990).
19. Weigend, F. & Ahlrichs, R. Balanced basis sets of split valence, triple zeta valence and quad-ruple zeta valence quality for H to Rn: Design and assessment of accuracy. *Phys. Chem. Chem. Phys.* **7**, 3297 (2005).
20. Marenich, A. V., Christopher C. J. & Truhlar, D. G. Universal Solvation Model Based on Solute Electron Density and on a Continuum Model of the Solvent Defined by the Bulk Dielectric Constant and Atomic Surface Tensions. *J. Phys. Chem. B* **113**, 6378–6396 (2009).
21. Humphrey, W., Dalke, A. & Schulten, K., `VMD – Visual Molecular Dynamics', *J. Molec. Graphics.* **14.1**, 33-38 (1996).
22. CYLview, 1.0b; Legault, C. Y., Université de Sherbrooke, 2009 (http://www.cylview.org)
23. Stefan G., Exploration of Chemical Compound, Conformer, and Reaction Space with Meta-Dynamics Simulations Based on Tight-Binding Quantum Chemical Calculations. *J. Chem. Theory Comput.* **15**, 2847-2862 (2019).
24. Tian Lu, Molclus program, Version 1.9, http://www.keinsci.com/research/molclus.html (accessed 3-24-2022).
25. Becke, A. D. *J. Chem. Phys*. **98**, 5648-5652 (1993).
26. Stephens, P. J., Devlin, F. J., Chabalowski, C. F. & Frisch, M. J. *J. Phys. Chem.* **98**, 11623-11627 (1994).
27. Lee, C., Yang, W. & Parr, R. G. *Phys. Rev. B* **37**, 785-789 (1988).
28. Lu, T. & Chen, F. *J. Comput. Chem*. **33**, 580 (2012).
29. Mackenzie W. K., Christopher R. T., Ga Young Lee, Hannah J. E., K. N. Houk, and Marcey L. Waters. More Than π–π–π Stacking: Contribution of Amide−π and CH−π Interactions to Crotonyllysine Binding by the AF9 YEATS Domain. *J. Am. Chem. Soc.* **142,** 40, 17048–17056 (2020).
